# Pf4 Phage Variant Infection Reduces Virulence-Associated Traits in *Pseudomonas aeruginosa*

**DOI:** 10.1128/spectrum.01548-22

**Published:** 2022-08-29

**Authors:** Damien Tortuel, Ali Tahrioui, Audrey David, Mélyssa Cambronel, Flore Nilly, Thomas Clamens, Olivier Maillot, Magalie Barreau, Marc G. J. Feuilloley, Olivier Lesouhaitier, Alain Filloux, Emeline Bouffartigues, Pierre Cornelis, Sylvie Chevalier

**Affiliations:** a Unité de Recherche Communication Bactérienne et Stratégies Anti-infectieuses, CBSA UR4312, Université de Rouen Normandie, Évreux, France; b Normandie Université, Fédération de Recherche SéSAD, Université de Rouen Normandie, Rouen, France; c MRC Centre for Molecular Bacteriology and Infection, Department of Life Sciences, Imperial College London, London, United Kingdom; d Laboratorium Microbiologie, Vrije Universiteit Brussel, Brussels, Belgium; Emory University School of Medicine

**Keywords:** Pf4 phage, virulence factors, *Pseudomonas aeruginosa*, RNA-seq, quorum sensing

## Abstract

Pf4 is a filamentous bacteriophage integrated as a prophage into the genome of Pseudomonas aeruginosa PAO1. Pf4 virions can be produced without killing P. aeruginosa. However, cell lysis can occur during superinfection when Pf virions successfully infect a host lysogenized by a Pf superinfective variant. We have previously shown that infection of P. aeruginosa PAO1 with a superinfective Pf4 variant abolished twitching motility and altered biofilm architecture. More precisely, most of the cells embedded into the biofilm were showing a filamentous morphology, suggesting the activation of the cell envelope stress response involving both AlgU and SigX extracytoplasmic function sigma factors. Here, we show that Pf4 variant infection results in a drastic dysregulation of 3,360 genes representing about 58% of P. aeruginosa genome; of these, 70% of the virulence factors encoding genes show a dysregulation. Accordingly, Pf4 variant infection (termed Pf4*) causes *in vivo* reduction of P. aeruginosa virulence and decreased production of *N*-acyl-homoserine lactones and 2-alkyl-4-quinolones quorum-sensing molecules and related virulence factors, such as pyocyanin, elastase, and pyoverdine. In addition, the expression of genes involved in metabolism, including energy generation and iron homeostasis, was affected, suggesting further relationships between virulence and central metabolism. Altogether, these data show that Pf4 phage variant infection results in complex network dysregulation, leading to reducing acute virulence in P. aeruginosa. This study contributes to the comprehension of the bacterial response to filamentous phage infection.

**IMPORTANCE** Filamentous bacteriophages can become superinfective and infect P. aeruginosa, even though they are inserted in the genome as lysogens. Despite this productive infection, growth of the host is only mildly affected, allowing the study of the interaction between the phage and the host, which is not possible in the case of lytic phages killing rapidly their host. Here, we demonstrate by transcriptome and phenotypic analysis that the infection by a superinfective filamentous phage variant causes a massive disruption in gene expression, including those coding for virulence factors and metabolic pathways.

## INTRODUCTION

Pseudomonas aeruginosa is a Gram-negative opportunistic pathogen that causes acute and chronic infections in immunocompromised hosts, including patients with cystic fibrosis (CF), burns, or cancers (1–3). P. aeruginosa is one of the most prevalent bacterial pathogens in the lungs of CF patients associated with poor clinical outcomes due to their problematic eradication (4, 5). This pathogen exhibits high intrinsic and acquired antibiotic resistance and is classified by the World Health Organization (February 2017) as “critical” (carbapenem resistant). Moreover, P. aeruginosa can switch from free-living (planktonic) to sessile (biofilm) lifestyles and vice versa depending on the environmental cues encountered at the infection site, causing acute and chronic infections, respectively. During acute infections, P. aeruginosa secretes virulence factors, including pyocyanin, siderophores (pyochelin and pyoverdine), and rhamnolipids, to avoid host defences and compete with host microbiota (2, 6–9). The regulation of these virulence factors is complex and multifactorial, allowing P. aeruginosa adaptation to a wide range of infection sites and environmental conditions. Most secreted virulence factors are controlled via quorum sensing (QS) (10), while exotoxin A and the siderophore pyoverdine are produced in response to iron starvation (11–13). The Las and Rhl QS systems use *N*-acyl-homoserine lactones (AHL), while the PQS system relies on 2-alkyl-4-quinolones (HAQ, PQS system) signaling molecules (14, 15). Biofilms are organized communities of microorganisms embedded into a self-produced matrix consisting of exopolysaccharides, extracellular DNA, vesicles, and proteins, which are often associated with chronic infections. These large structures protect bacteria from antimicrobials and the host immune system (16).

Inoviruses are filamentous bacteriophages that are widespread and associated with chronic lung infections (17). P. aeruginosa Pf phages can be extruded from the host cell without killing the bacterium, allowing virions to accumulate to high titers in biofilms (10^11^ mL^−1^) (18) or in the sputa of CF patients (10^7^ mL^−1^) (19–21). About 68% of chronic wounds infected by P. aeruginosa harbor Pf bacteriophages, and the presence of the phage causes a maladaptive immune response against the virus, resulting in more chronic persistence of the pathogen (22). P. aeruginosa PAO1 has a filamentous Pf4 phage integrated within its genome. Lysogenized bacteria defend against infection by the same phage through a mechanism called superinfection exclusion, which is promoted by the phage protein PfsE. This protein has been shown to bind to the bacterial PilC protein (23), thus inhibiting assembly of the type 4 pili, which serve as Pf4 cell surface receptors (24). Superinfective phage variants can, however, emerge and successfully infect and partly kill a host lysogenized by a Pf prophage. The molecular mechanism leading to the production of superinfective variants is far from clear. The accumulation of reactive oxygen species within the biofilms was shown to lead to a hypermutation of a region of Pf4 prophage genome located between PA0716 and PA0717 (18, 25). Two new genes have been described, including *xisF4* encoding an excisionase and *pf4r* encoding a repressor of *xisf4* (26). Superinfective Pf4 variants cause bacterial death within the microcolonies of old biofilms and dispersion (27, 28). Such variants have been associated with bacterial biofilm organization and maturation, stress tolerance, and virulence (28–30).

In a previous study, we identified a transposon mutant derived from P. aeruginosa H103 (dH103Pf4^+^ PAO1 strain) overproducing superinfective Pf4 phages termed Pf4* (31). Pf4* displayed characteristics of a superinfective variant, i.e., it was able to induce cell lysis on its wild-type host. Sequencing of the Pf4* prophage genomic region led to identify numerous mutations in PA0723, PA0724 and PA0725, but not in the genes (*pf4r*) or regions (PA0716-PA0717) that were previously related to the superinfective phenotype (26). We have shown that P. aeruginosa exposure to Pf4* resulted in altered biofilm architecture with increased matrix-encoded gene expression and c-di-GMP production. In addition, in flow cell dynamic conditions, numerous sessile bacteria were displaying a filamentous morphology (31). Noticeably, the cell envelope stress response (CESR) that is mediated by two extracytoplasmic function (ECF) sigma factors, AlgU and SigX, was strongly activated in response to Pf4* infection, suggesting a link between the regulation of the cell shape and the reorganization of cytoskeleton-like structures (31). Here, we conducted a transcriptome sequencing (RNA-seq)-based study to get further insights into the response of P. aeruginosa upon Pf4* phage infection.

## RESULTS AND DISCUSSION

### Pf4* infection of *P. aeruginosa* H103 leads to deep gene expression alterations.

**FIG 1:**
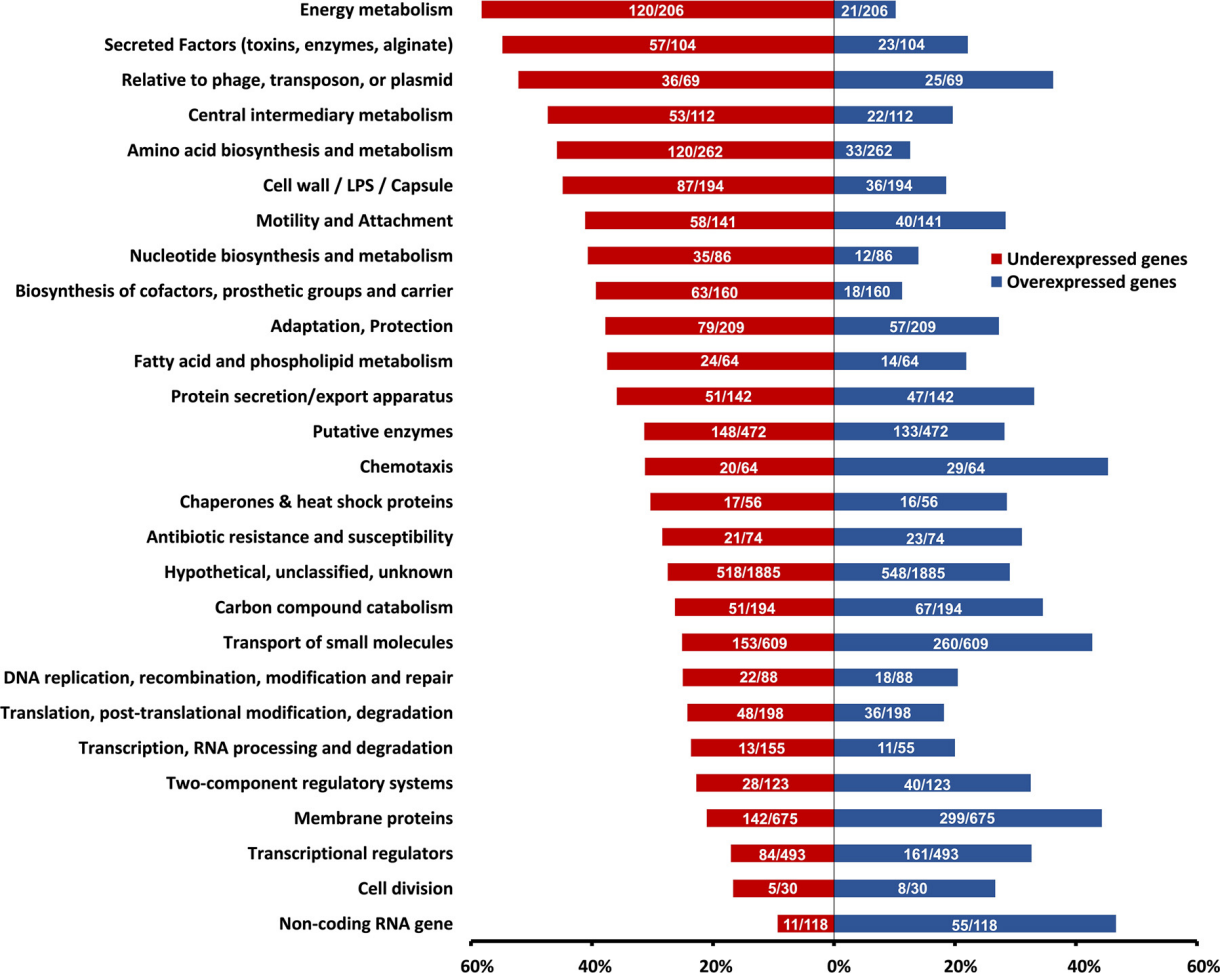
PseudoCAP analysis of RNA-seq study. Each PseudoCAP category (32) represented in the histogram is composed of the proportions of underexpressed genes (red bar) and overexpressed genes (blue bar) relative to this category. Numbers in the histogram bars represent the absolute numbers of overexpressed (blue bars) and underexpressed (red bars) genes on the total genes included in each PseudoCAP category.

P. aeruginosa H103 was infected by a Pf4 phage variant that was previously described (31) at a final titer of 1.5 × 10^3^ PFU mL^−1^. Total RNAs were extracted from planktonic cultures at an *A*_580_ of 2.8 (see Materials and Methods). A global comparative transcriptomic analysis revealed that a total of 3,360 genes (i.e., 58.9% of the bacterial genome) were differentially expressed by >2-fold (*P < *0.05 by Empirical Bayes statistical test ; see Table S1 in the supplemental material), when P. aeruginosa H103 was infected by Pf4* compared to untreated bacteria. Among these genes, 1,686 and 1,674 were down- and upregulated in Pf4*-treated bacteria, respectively. Forty-eight genes that were differentially expressed by RNA-seq analysis were selected for validation by quantitative reverse-transcription real-time PCR (RT-qPCR), and the data for both methods displayed a very good correlation (squared Pearson’s correlation coefficient of 0.9599 [see Fig. S1]). The differentially expressed genes were then classified according to their functional categories (PseudoCAP) (32). Noticeably, most of the genes belonging to “membrane proteins” (44.3%), “noncoding RNA” (46.6%), or “chemotaxis” (45.3%) functional classes were upregulated after Pf4* treatment (Fig. 1). Conversely, genes belonging to the classes “cell wall and LPS” (44.8% of the genes belonging to this specific functional class), “secreted factors” (54.8%), “metabolism” (amino acid (45.8%), central metabolism (47.3%), energy metabolism (58.3%), or “relative to phage, transposon, or plasmid” (52.2%) functional classes were mostly downregulated in response to Pf4* treatment (Fig. 1). The “relative to phage, transposon, or plasmid” functional class is separated into two groups; the first group included several pyocins (R2 and F2 filamentous pyocins and S4- and S5-type soluble pyocins) that were downregulated (36 genes out of 69), and the second group, composed of Pf4-related genes, integrases, and transposases, were upregulated (see Table S1). Genes related to Pf4 phage were the most upregulated, their fold change ranging from 4.8 (PA0728) to 11.4 (PA0718). Accordingly, the supernatant of dH103Pf4^+^ strain, from which Pf4* phage was produced (31), did not contain R2-type pyocins, since no lysis plaque were observed when performing PAK strain infection, a strain that is sensitive to this type of pyocin (31). The huge overexpression of the Pf4 gene loci was confirmed by RT-qPCR on PA0717, the expression of which showed an excellent correlation between the two techniques (RT-qPCR and RNA-sequencing; see Fig. S1) (31). Interestingly, the Pf superinfective exclusion protein encoded by the gene *pfsE* (PA0721) (23) was increased by 4.1-fold under these conditions (see Table S1), explaining at least partly the P. aeruginosa H103 resistance to Pf4* plaque formation and the absence of twitching motility upon Pf4* infection (31). Noticeably, bacteria that survive Pf superinfection were shown to transiently display these phenotypes (23). Even though Pf4* infection generates a huge gene dysregulation with almost 60% of genes differentially regulated, P. aeruginosa’s growth was not severely affected by Pf4* phage infection (31). Whereas lytic phages hijack host metabolism extremely rapidly leading to bacterial death after few minutes, filamentous phages establish a chronic infection of their hosts with limited cell lysis. Since this transcriptomic study was performed 7 h postinfection, the gene dysregulation could result from the adaptation P. aeruginosa of to Pf4* infection. Consequently, these data might reflect the establishment of a host-pathogen dynamic of chronic infection at the host gene expression level.

### Decreased P. aeruginosa virulence in response to Pf4* infection.

**FIG 2:**
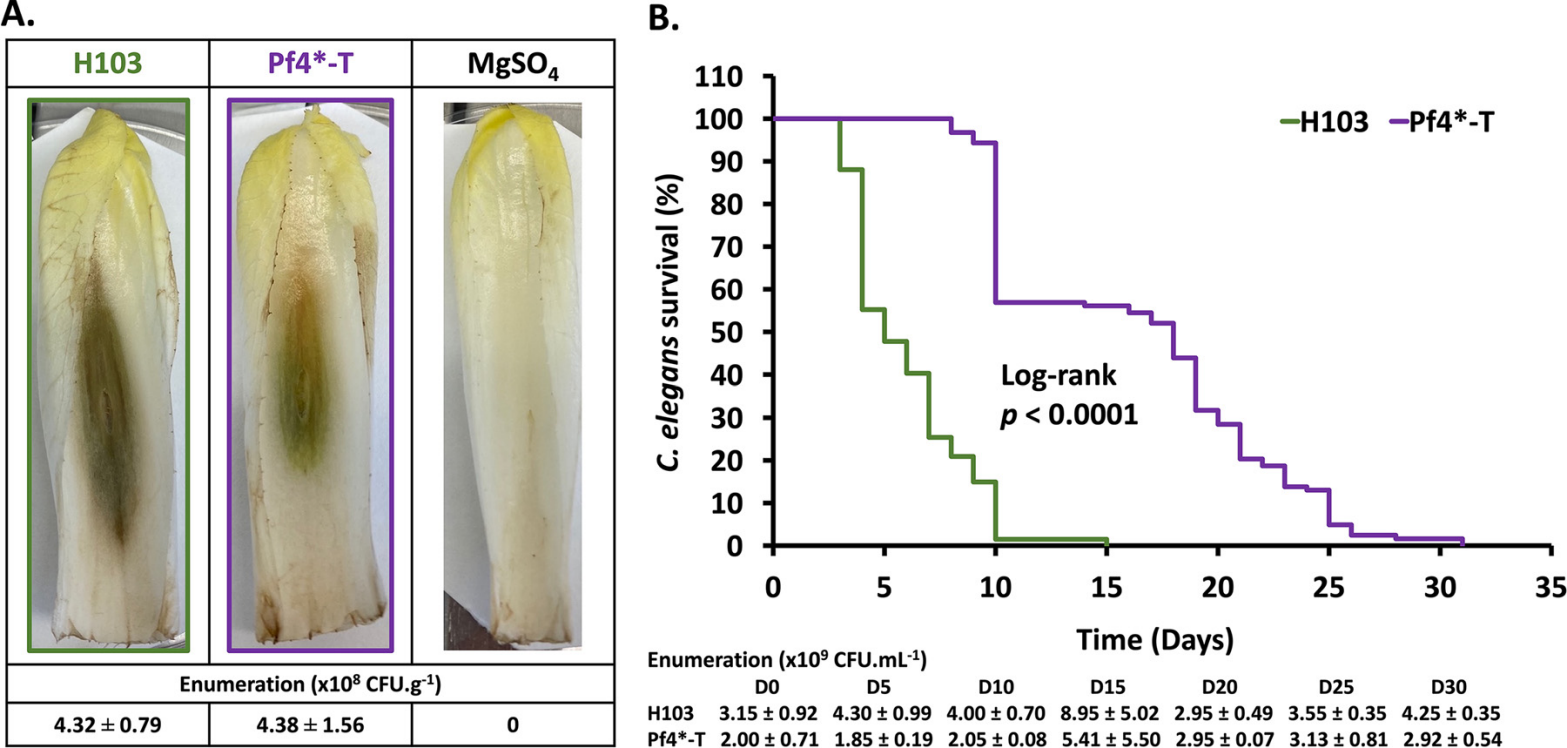
Pf4 phage variant infection leads to a decrease in P. aeruginosa virulence. (A) Representative pictures of infected leaves of Belgian endives by H103 and Pf4*-T H103 and the bacterium-free buffer (MgSO_4_ 10 mM) negative control. The mean bacterial numerations ± the SEM from rots are indicated above the pictures. (B) Kaplan-Meier survival plots of C. elegans nematodes in contact with P. aeruginosa H103 (green curve) (*n* = 67) or Pf4*-T (purple curve) (*n* = 123). Means of bacterial numerations ± the SEM determined every 5 days by scraping the entire NGM plate are indicated at the bottom of the panel. Statistics were determined by pairwise comparison (log-rank test). Each experiment was assayed at least three times independently.

The transcriptomic data analysis revealed that 257 (69.65%) of 369 genes annotated as encoding virulence factors in P. aeruginosa (32) were dysregulated upon Pf4* treatment. Strikingly, 61.48% of these genes were downregulated (see Table S1, “classified by PA numbers”). This over-representation of dysregulated virulence-related genes prompted us to investigate further virulence-related traits upon Pf4* treatment. We first investigated the virulence using two multicellular models, the Belgian endive *Cichorium intybus* var. *foliosum* L and the nematode Caenorhabditis elegans. Pf4*-treated or untreated P. aeruginosa H103 cells were inoculated within the middle vein of Belgian endives leaves, and necrosis was allowed to develop for 5 days (Fig. 2A). Inoculation of treated and untreated P. aeruginosa led to leaf necrosis that was smaller in extent when Pf4*-treated bacteria were injected. As expected, the control condition consisting of a 10 mM MgSO_4_ solution that was used to wash and resuspend the bacteria prior to infection did not produce any necrosis, suggesting that Pf4* exposure reduces P. aeruginosa virulence. To ascertain that the observed reduced virulence resulted from Pf4* exposure and not from a growth difference between treated and untreated P. aeruginosa under this condition, bacterial enumeration was performed for each leaf. As shown in Fig. 2A, a similar bacterial load was measured in each case, with means of 4.32 × 10^8^ and 4.38 × 10^8^ CFU g^−1^ of endive from leaves inoculated with H103 wild-type and Pf4*-treated samples, respectively, showing that Pf4* exposure reduced P. aeruginosa virulence without affecting *in planta* growth. We then investigated the virulence using the nematode C. elegans model. P. aeruginosa can kill C. elegans in an infection-like process, using Pf4* infected and untreated bacteria as a food supply (33). Upon Pf4* exposure, P. aeruginosa was significantly less virulent toward C. elegans since 50% of the nematode population was still alive after 17 days, whereas it was after 5 days when using untreated bacteria as the food supply (Fig. 2B, *P* < 0.0001). Accordingly, all worms were dead after 31 or 15 days with Pf4*-treated or untreated P. aeruginosa, respectively (Fig. 2B). Enumeration every 5 days showed that these bacteria were still alive for the duration of the assay, with the number of live bacteria ranging from 1.85 × 10^9^ to 8.95 × 10^9^ CFU mL^−1^ (Fig. 2B). Altogether, these data indicate that Pf4* exposure causes a reduction in P. aeruginosa virulence in line with previous data obtained using other experimental models (34). Indeed, when Pf4* was added to P. aeruginosa cultures, the bacteria showed lower cytotoxicity and virulence in mice (24), as well as reduced production of the siderophore pyoverdine (35). Interestingly, a recent study shows that Pf4 phages influence many virulence factors of newly infected P. aeruginosa strains, with the exception of swimming motility and biofilm production (36). In addition, it was recently shown that the superinfection exclusion protein PfsE binds to PilC to avoid extension of the of type IV pili, hence affecting twitching motility (23). Since type IV pili play important roles in virulence and biofilm formation (37–39), it was suggested that PfsE may be involved in the virulence of P. aeruginosa through type IV pilus activity inhibition (23). Interestingly, *pfsE* expression (PA0721) was greatly overexpressed in our study, which may be correlated with the decreased virulence observed under our conditions (see Table S1). Consistent with a role in P. aeruginosa pathogenesis, Pf4 phage has been shown to contribute to the virulence of P. aeruginosa infections in animal models of acute lung infection (29, 34). Indeed, mice infected with a P. aeruginosa strain impaired in the production of Pf4 phages survived significantly longer than those infected with an isogenic wild-type P. aeruginosa strain, suggesting that Pf4 contributes to the virulence of P. aeruginosa PAO1 (29). However, in that study (29), the virulence of a Pf phage-deficient mutant was compared to that of wild-type bacteria, where, presumably, the level of Pf phage produced by P. aeruginosa *in vivo* was probably not as high as that observed under *in vitro* conditions, where phage titers could be as high as 10^10^ PFU/mL (18). Using a different approach in which Pf4 filamentous phages at levels comparable to those achieved in biofilms were added to P. aeruginosa PAO1 culture, Secor et al. showed that Pf4-infected bacteria showed reduced cytotoxicity and virulence while promoting phenotypes associated with chronic infections in a mouse model of lung infection (34), suggesting that that Pf4 phage may contribute to the establishment of chronic infections and may help P. aeruginosa evade host defense mechanisms (34). Accordingly, Pf4 phages were shown to promote P. aeruginosa wound infection in mice and to be associated with chronic wound infections in humans (22). In addition, acute infection of P. aeruginosa by the Pf4 bacteriophage inhibited the production of the virulence factor pyoverdine (35, 40). Recently, it was shown that Pf4 phages were produced in larger amounts upon exposure to sublethal concentrations of ciprofloxacin and mitomycin C (36). Interestingly, the released Pf4 virions were able to successfully infect new strains of P. aeruginosa, establishing very complex interactions with other indigenous filamentous (pro)phages (36). Infections by these phages reduced pyocyanin and pyoverdine production of lysogenic strains, suggesting that Pf4 decreased the toxicity of P. aeruginosa strains (36). In other bacteria, such as Ralstonia solanacearum, infection by the ϕRSS1 filamentous phage increases virulence through enhancement of expression of virulence factors encoding genes (41), while ϕRSM3 filamentous phage infection leads to a decreased virulence (42), thereby confirming the relationship between virulence and filamentous phage infection.

### Decreased production and secretion of virulence factors.

**FIG 3:**
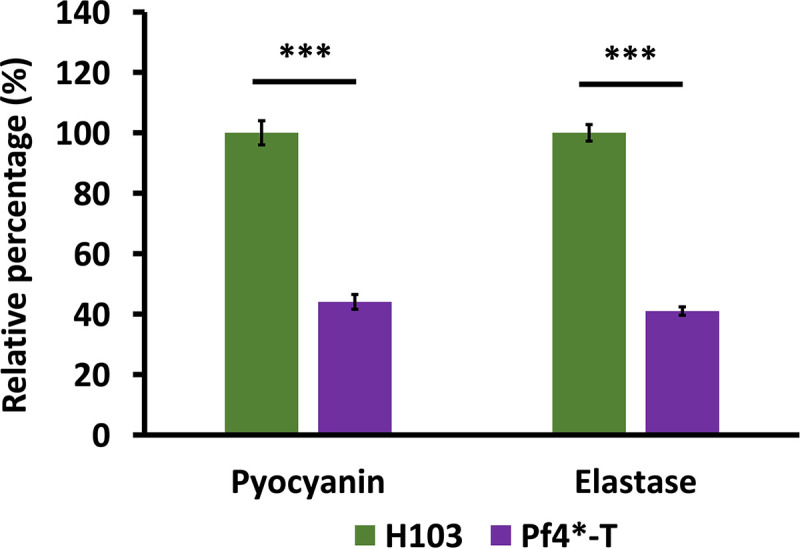
Pyocyanin and elastase activity were decreased upon Pf4 phage variant infection. The relative quantifications (± the SEM) of pyocyanin production and elastase activity, determined by absorbance measurement at 520 nm and by elastolytic activity assay, respectively, in H103 (green bars) and Pf4*-T (violet bars) condition are shown. All measures were normalized to the *A*_580_. Pyocyanin and elastase experiments were assayed four times independently. Statistics were achieved by using a paired (two sample) two-tailed *t* test (***, *P < *0.001).

Next, we addressed whether the virulence reduction upon exposure to Pf4* is due to decreased virulence factor production. Noticeably, the expression of genes encoding virulence factors and their related export systems were strongly decreased upon Pf4* infection (Table 1, asterisks). LasA and LasB are extracellular elastolytic metalloproteinases involved in tissue and epithelial junction damage (43, 44), and the phenazine pyocyanin contributes to tissue damage and neutrophil defense inactivation (9, 45–47) and to Caenorhabditis elegans killing (48). The production of elastase and pyocyanin was reduced by about 60% upon Pf4* infection (Fig. 3). The expression of *lasA* and *lasB*, as well as the two operons that are involved in phenazine biosynthesis (*phzA1-G1* and *phzA2-G2* for the biosynthesis of the phenazine 1-carboxylic [PCA]) and the *phzS* and *phzM* genes for the conversion of PCA to pyocyanin (49), was strongly decreased (Table 1). Accordingly, the genes encoding the proteins involved in the Xcp-type II secretion system (T2SS), which are involved in secretion of proteins, including the protease LasA and the elastase LasB (50), were downregulated upon Pf4* infection (Table 1). Phenazines, being small molecules, are likely to be exported via efflux systems, and the MexGHI-OpmD RND pump has been shown to be involved in the export of a precursor of pyocyanin (51). Notably, the *opmD* gene encoding the outer membrane efflux component of the pump shows a very strong downregulation (Table 1). In addition, genes encoding proteins of the type I secretion system (T1SS), and the secreted AprA protease (−44-fold), the type Va secretion system (T5aSS), and two of the three type VI secretion systems (H2 and H3-T6SS), as well as their cognate effectors, were downregulated upon Pf4* infection, especially in the case of H3-T6SS (Table 1). Noticeably, these two secretion systems have been associated with P. aeruginosa pathogenesis (52, 53). H2-T6SS and H3-T6SS have been proposed to be positively controlled by the QS regulators LasR and PqsR (52), which will be discussed below. Conversely, genes encoding the Hxc of the T2SS, the T3SS, the T5bSS, and the T5dSS, and their associated virulence factors were overexpressed (Table 1). This was particularly true for T3SS and its effectors the *exoT* and *exoS* genes, which were upregulated, as well as *exsA*, encoding the T3SS-master regulator (54). However, *exsA* transcription is regulated by the master virulence regulator Vfr, whose expression was decreased by 4.4-fold (54) (see Table S1). We assessed T3SS functionality through the production of PcrV effector and cytotoxicity. No difference was observed either in terms of the presence of PcrV in Pf4*-treated or untreated P. aeruginosa supernatants or of cytotoxicity in lung A549 cells (see Fig. S2), suggesting that the activity of T3SS was not affected by Pf4* infection. Interestingly, ExoT protected cells *in vitro* from type III machinery-dependent cytotoxicity (55), and the ExoS chaperone encoding gene (*spcS*) expression was reduced upon Pf4* infection (Table 1, −4.29-fold), suggesting that ExoS may not be functional under our conditions. Interestingly, Pf4* infection was previously shown to induce a cell envelope stress response (CESR) involving at least the two ECF sigma factors, AlgU and SigX (31). This was confirmed here by the RNA-seq data since their encoding genes and target genes were strongly increased (Table 1). Noticeably, AlgU hyperactivity was previously associated with reduced expression of numerous acute virulence factors, including LasA, RhlA, and HcnA (56–59). AlgU activates the transcription of *algR*, encoding a major repressor of Vfr and of CzcR, which represses phenazine genes transcription (60). Accordingly, *algR* transcription was increased, while that of *czcR* and *vfr* was decreased in response to Pf4* infection (Table 1).

**TABLE 1:** Virulence-related selected genes up- and downregulated upon Pf4* infection

PA no.	Gene^*a*^	Product name and/or function	Regulator(s)	Fold change	
				RNA-seq	RT-qPCR
Secretion systems					
SEC secretion system					
PA3820	*secF*	Secretion protein SecF		−3.27	
PA3821	*secD*	Secretion protein SecD		−3.52	
PA3822	*yajC*	Conserved hypothetical protein		−2.66	
PA4403	*secA*	Secretion protein SecA		−2.96	
PA4747	*secG*	Secretion protein SecG		3.28	
PA5128	*secB*	Secretion protein SecB		−3.66	
TAT secretion system					
PA5068	*tatA*	Translocation protein TatA		−2.23	
PA5069	*tatB*	Translocation protein TatB		−2.54	
PA5070	*tatC*	Transport protein TatC		−2.70	
Type 1 secretion system: APR					
PA1245	aprX*	AprX		−3.94	
PA1246	aprD*	Alkaline protease secretion protein AprD		−4.15	
PA1247	aprE*	Alkaline protease secretion protein AprE		−7.09	
PA1248	aprF*	Alkaline protease secretion OM pAprF precursor		−10.53	
PA1249	aprA*	Alkaline metalloproteinase precursor		−45.45	
PA1250	aprI*	Alkaline proteinase inhibitor AprI		−4.12	
Type 2 secretion system: HXC					
PA0677	*hxcW*	HxcW		3.89	
PA0678	*hxcU*	HxcU		2.81	
PA0679	*hxcP*	HxcP		2.60	
PA0680	*hxcV*	HxcV		5.09	
PA0681	*hxcT*	HxcT		10.55	
PA0682	*hxcX*	HxcX		25.02	
PA0683	*hxcY*	HxcY		6.09	
PA0684	*hxcZ*	HxcZ		10.33	
PA0685	*hxcQ*	HxcQ		13.41	
PA0686	*hxcR*	HxcR		11.11	
PA0687	*hxcS*	HxcS		18.82	
Type 2 secretion system: XCP					
PA3095	xcpZ*	General secretion pathway protein M		−3.27	
PA3096	xcpY*	General secretion pathway protein L		−3.13	
PA3097	xcpX*	General secretion pathway protein K		−2.00	
PA3098	xcpW*	General secretion pathway protein J		−5.35	
PA3099	xcpV*	General secretion pathway protein I		−9.17	
PA3100	xcpU*	General secretion pathway OM protein H precursor		−15.87	
PA3101	xcpT*	General secretion pathway protein G		−17.24	
PA3102	xcpS*	General secretion pathway protein F		−3.57	
PA3103	xcpR*	General secretion pathway protein E		−3.77	
PA3105	xcpQ*	General secretion pathway protein D		−2.55	
Type 2 secretion system: XCP-elated proteins					
PA1867	*xphA*	XphA		6.62	
PA1868	*xqhA*	Secretion protein XqhA		6.92	
Type 2 secretion system: XCP-dependent secreted factors					
PA0026	plcB*	Phospholipase C, PlcB		−6.06	
PA0572	impA*	Hypothetical protein		−27.03	
PA0843	*plcR*	Phospholipase accessory protein PlcR precursor		4.81	
PA0844	*plcH*	Hemolytic phospholipase C precursor		2.94	
PA0852	cbpD*	Chitin-binding protein CbpD precursor		−33.33	
PA2862	*lipA*	Lactonizing lipase precursor		2.02	
PA2939	paaP*	Probable aminopeptidase		−50.00	
PA3296	*phoA*	Alkaline phosphatase		2.95	
PA3319	*plcN*	Nonhemolytic phospholipase C precursor		8.68	
PA3910	*eddA*	Extracelullar DNA degradation protein, EddA		3.91	
PA4175	piv*	Protease IV		−29.41	
PA4813	*lipC*	Lipase LipC		6.38	
Type 3 secretion system					
PA1690	*pscU*	Translocation protein in type III secretion	ExsA	7.95	
PA1691	*pscT*	Translocation protein in type III secretion	ExsA	13.76	
PA1692	*pscS*	Translocation protein in type III secretion	ExsA	6.42	
PA1693	*pscR*	Translocation protein in type III secretion	ExsA	5.37	
PA1694	*pscQ*	Translocation protein in type III secretion	ExsA, RsmA	−2.01	
PA1696	*pscO*	Translocation protein in type III secretion	ExsA	2.59	
PA1697	*pscN*	ATP synthase in type III secretion system	ExsA	3.40	
PA1698	*popN*	Type III secretion OM protein PopN precursor	ExsA	2.14	
PA1699	*pcr1*	Pcr1	ExsA, RsmA	2.46	
PA1700	*pcr2*	Pcr2	ExsA	2.43	
PA1701	*pcr3*	Pcr3	ExsA, RsmA	5.04	
PA1702	*pcr4*	Pcr4	ExsA	3.84	
PA1703	*pcrD*	Type III secretory apparatus protein PcrD	ExsA	2.48	
PA1705	*pcrG*	Regulator in type III secretion	ExsA	4.22	
PA1713	*exsA*	Transcriptional regulator ExsA	PsrA, PtrA, PtrB, PtrC, Vfr, RsmA	2.00	4.63
PA1714	*exsD*	ExsD	ExsA, RsmA	2.84	
PA1715	*pscB*	Type III export apparatus protein	ExsA	9.61	
PA1716	*pscC*	Type III secretion OM protein PscC precursor	ExsA	9.26	
PA1717	*pscD*	Type III export protein PscD	ExsA	11.51	
PA1718	*pscE*	Type III export protein PscE	ExsA, RsmA	3.76	
PA1719	*pscF*	Type III export protein PscF	ExsA, RsmA	2.06	
PA1721	*pscH*	Type III export protein PscH	ExsA, RsmA	2.22	
PA1722	*pscI*	Type III export protein PscI	ExsA, RsmA	2.33	
PA1723	*pscJ*	Type III export protein PscJ	ExsA, RsmA	2.74	
PA1724	*pscK*	Type III export protein PscK	ExsA	2.67	
PA1725	*pscL*	Type III export protein PscL	ExsA	2.79	
Type 3 secretion system-dependent secreted factors					
PA0044	*exoT*	Exoenzyme T	RsmA	3.47	7.06
PA2191	*exoY*	Adenylate cyclase ExoY	RsmA	3.53	
PA3842	*spcS*	Specific Pseudomonas chaperone for ExoS		−4.29	
Type 3 secretion system: regulators					
PA0612	*ptrB*	Repressor, PtrB	PrtR	4.17	9.66
PA2486	*ptrC*	Pseudomonas type III repressor gene C, PtrC		5.69	
PA2808	*ptrA*	Pseudomonas type III repressor A		11.12	
PA3006	*psrA*	Transcriptional regulator PsrA		18.28	46.11
PA4916	nrtR*	Nudix-related transcriptional regulator NrtR		3.90	
PA4917	nadD2*	NadD2	NrtR	5.03	
Type 5A secretion system					
PA5112	*estA*	Esterase EstA		−2.68	
Type 5B secretion system					
PA4541	*lepA*	Large extracellular protease		3.83	
Type 5D secretion system					
PA3339	*plpD*	Patatin-like protein, PlpD		2.62	
Type 6 secretion system (H1-T6SS)					
PA0074	*ppkA*	Serine/threonine protein kinase PpkA	AmrZ, RsmA	−2.13	
PA0078	*tssL1*	TssL1	AmrZ	−2.83	
PA0079	*tssK1*	TssK1	AmrZ, RsmA	−2.42	
PA0080	*tssJ1*	TssJ1	AmrZ	−2.04	
PA0084	*tssC1*	TssC1	AmrZ, RsmA	−2.13	
Type 6 secretion system (H2-T6SS)					
PA1657	hsiB2*	HsiB2	AmrZ, CueR, Fur, RpoN	−2.29	
PA1658	hsiC2*	HsiC2	AmrZ, CueR, Fur, RpoN	−3.38	
PA1659	hsiF2*	HsiF2	AmrZ, CueR, Fur, RpoN	−3.55	
PA1660	hsiG2*	HsiG2	AmrZ, CueR, Fur, RpoN	−4.65	
PA1661	hsiH2*	HsiH2	AmrZ, CueR, Fur, RpoN	−3.30	
PA1662	clpV2*	clpV2	AmrZ, CueR, Fur, RpoN	−2.99	
PA1663	sfa2*	Sfa2	AmrZ, CueR, Fur, RpoN	−3.24	
PA1664	orfX*	OrfX	AmrZ, CueR, Fur, RpoN	−3.61	
PA1665	fha2*	Fha2	AmrZ, CueR, Fur, RpoN	−5.24	
PA1666	lip2*	Lip2	AmrZ, CueR, Fur, RpoN	−5.00	
PA1667	hsiJ2*	HsiJ2	AmrZ, CueR, Fur, RpoN	−3.85	
PA1668	dotU2*	DotU2	AmrZ, CueR, Fur, RpoN	−2.62	
Type 6 secretion system (H3-T6SS)					
PA2359	*sfnR2*	Probable transcriptional regulator	AmrZ, RpoN, Fur	2.54	
PA2360	*hsiA3*	Hypothetical protein	AmrZ, RpoN, Fur	−11.49	
PA2361	icmF3*	IcmF3	AmrZ, RpoN, Fur	−2.62	
PA2363	hsiJ3*	HsiJ3	AmrZ, RpoN, Fur	−3.85	
PA2365	hsiB3*	HsiB3	AmrZ, RpoN, Fur	−35.71	
PA2366	hsiC3*	HsiC3	AmrZ, RpoN, Fur	−47.62	
PA2367	hcp3*	Hcp3	AmrZ, RpoN, Fur	−55.56	
PA2368	hsiF3*	HsiF3	AmrZ, RpoN, Fur	−66.67	
PA2369	hsiG3*	HsiG3	AmrZ, RpoN, Fur	−32.26	
PA2370	hsiH3*	HsiH3	AmrZ, RpoN, Fur	−41.67	
PA2371	clpV3*	ClpV3	AmrZ, RpoN, Fur	−25.64	
PA2372	*	Hypothetical protein	AmrZ, RpoN, Fur	−17.24	
PA2373	vgrG3*	VgrG3	AmrZ, RpoN, Fur	−7.46	
PA2374	tseF*	TseF	AmrZ, RpoN, Fur	−7.58	
Type 6 secretion system-associated genes					
PA1512	*hcpA*	Secreted protein Hcp		−2.00	
PA1844	*tse1*	Tse1		2.06	
PA2685	*vgrG4*	VgrG4		2.63	
PA2703	*tsi2*	Tsi2		−2.79	
PA2774	*tse4*	Tse4		2.76	
PA2775	*tsi4*	Tsi4		3.38	
PA3291	*tli1*	Tli1		−2.64	
PA3294	*vgrG4a*	VgrG4a		−2.34	
PA3485	*tsi3*	Tsi3		−3.24	
PA3486	*vgrG4b*	VgrG4b		−2.28	
PA3487	*tle5*	Tle5		−3.09	
PA3488	*tli5*	Tli5		−2.40	
PA5086	*tli5b1*	Type VI secretion lipase immunity protein		4.55	
PA5088	*tli5b3*	Type VI secretion lipase immunity protein		−2.45	
PA5089	*tle5b*	Type VI secretion phospholipase D effector		−2.36	
PA5090	*vgrG5*	VgrG5		−2.04	
PA5266	*vgrG6*	VgrG6		−2.90	
PA5267	*hcpB*	Secreted protein Hcp		−3.36	
Quorum sensing					
LAS					
PA1430	lasR*	Transcriptional regulator LasR	Vfr, GacA, AlgQ, QscR, QslA, QteE, RpoN	−4.35	−1.31
PA1431	rsaL*	Regulatory protein RsaL	RsaL, MvaT, RpoN, VqsR, PprB	−5.03	
RHL					
PA3476	rhlI*	Autoinducer synthesis protein RhlI	DksA, RpoS, RpoN, PprB, AlgR	−2.13	1.068
PA3477	rhlR*	Transcriptional regulator RhlR	PhrD, Vfr, GacA, PprB, AlgQ, QteE, RpoN, BfmR	−13.33	−1.31
PQS					
PA0996	pqsA*	PqsA	Fur	−12.50	−4.61
PA0997	pqsB*	PqsB	Fur	−21.74	
PA0998	pqsC*	PqsC	Fur	−21.74	
PA0999	pqsD*	3-Oxoacyl-[acyl-carrier-protein] synthase III	Fur	−13.33	
PA1000	pqsE*	Quinolone signal response protein	Fur	−7.75	
PA1001	phnA*	Anthranilate synthase component I	Fur	−6.41	
PA1002	phnB*	Anthranilate synthase component II	Fur	−9.17	
PA1003	*mvfR* (*pqsR*)*	Transcriptional regulator MvfR (PqsR)	PvdS, OxyR, PhrS, QslA	−4.72	−1.63
PA2587	pqsH*	Probable FAD-dependent monooxygenase	CdpR	−7.04	
PA4190	pqsL*	Probable FAD-dependent monooxygenase		−9.52	
Quorum-sensing:regulators					
PA0714.1	*phrD*	PhrD		14.75	
PA1032	*quiP*	QuiP		−3.12	
PA1244	*qslA*	QslA		−5.68	
PA1898	*qscR*	Quorum-sensing control repressor	VqsR	13.46	17.70
PA2226	*qsrO*	QsrO		−9.17	
PA2227	*vqsM*	AraC-type transcriptional regulator VqsM	CdpR, QsrO	−3.76	
PA3305.1	*phrS*	PhrS	Anr	−2.72	−2.20
Virulence factors					
Phenazines					
PA0051	phzH*	Potential phenazine-modifying enzyme		−8.40	
PA1899	phzA2*	Probable phenazine biosynthesis protein		−16.39	−8.77
PA1900	phzB2*	Probable phenazine biosynthesis protein		−37.04	
PA1901	phzC2*	Phenazine biosynthesis protein PhzC		−16.39	
PA1902	phzD2*	Phenazine biosynthesis protein PhzD		−17.24	
PA1903	phzE2*	Phenazine biosynthesis protein PhzE		−19.23	
PA1904	phzF2*	Probable phenazine biosynthesis protein		−21.28	
PA1905	phzG2*	Probable pyridoxamine 5′-phosphate oxidase		−23.81	
PA4209	phzM*	Phenazine-specific methyltransferase		−2.39	
PA4210	phzA1*	Probable phenazine biosynthesis protein		−9.52	−5.52
PA4211	phzB1*	Probable phenazine biosynthesis protein		−6.41	
PA4212	phzC1*	Phenazine biosynthesis protein PhzC		−13.16	
PA4213	phzD1*	Phenazine biosynthesis protein PhzD		−19.61	
PA4214	phzE1*	Phenazine biosynthesis protein PhzE		−19.61	
PA4215	phzF1*	Probable phenazine biosynthesis protein		−21.74	
PA4216	phzG1*	Probable pyridoxamine 5′-phosphate oxidase		−23.26	
PA4217	phzS*	Flavin-containing monooxygenase		−6.67	
Elastases					
PA1871	lasA*	LasA protease precursor		−62.50	
PA3724	lasB*	Elastase LasB	AlgQ	−40.00	−14.49
Rhamnolipids					
PA1130	rhlC*	Rhamnosyltransferase 2		−10.87	
PA3478	rhlB*	Rhamnosyltransferase chain B	AlgR, RhlR	−22.22	
PA3479	rhlA*	Rhamnosyltransferase chain A	AlgR, RhlR	−15.87	−6.25
Lectins					
PA2570	lecA*	LecA	RhlR	−4.37	−2.24
PA3361	lecB*	Fucose-binding lectin PA-IIL	AlgU, RhlR	−10.53	
Hydrogen cyanide					
PA2193	hcnA*	Hydrogen cyanide synthase HcnA	RhlR, AlgR, RsmA	−8.33	
PA2194	hcnB*	Hydrogen cyanide synthase HcnB	RhlR, AlgR, RsmA	−9.35	
PA2195	hcnC*	Hydrogen cyanide synthase HcnC	RhlR, AlgR, RsmA	−12.20	
CHP/VFR pathway					
PA0413	*chpA*	Component of chemotactic signal transduction system		−2.25	
PA0414	*chpB*	Probable methylesterase		−2.82	
PA0417	*chpE*	Probable chemotaxis protein		2.84	
PA0652	*vfr*	Transcriptional regulator Vfr	Vfr, AlgR	−4.39	−1.85
PA5272	*cyaA*	Adenylate cyclase		2.12	7.79
Others					
PA0041		Probable hemagglutinin		2.92	
PA0423	*pasP*	PasP		−8.85	
PA0707	*toxR*	Transcriptional regulator ToxR	PvdS, Vfr	−3.04	
PA2258	*ptxR*	Transcriptional regulator PtxR	PvdS, Vfr	11.78	
Iron homeostasis					
Pyoverdine					
PA2385	*pvdQ*	3-Oxo-C_12_-homoserine lactone acylase PvdQ	PvdS, FpvI	−8.47	
PA2386	*pvdA*	l-Ornithine N5-oxygenase	PvdS, FpvI	−13.16	
PA2389	*pvdR*	PvdR	PvdS	−2.37	
PA2390	*pvdT*	PvdT	PvdS	−4.61	
PA2391	*opmQ*	Probable outer membrane protein precursor	PvdS	−8.70	
PA2392	*pvdP*	PvdP	PvdS	−3.26	
PA2393	*pvdM*	Putative dipeptidase	PvdS	−12.35	
PA2394	*pvdN*	PvdN	PvdS	−14.49	
PA2395	*pvdO*	PvdO	PvdS	−8.47	
PA2396	*pvdF*	Pyoverdine synthetase F	PvdS	−5.46	
PA2397	*pvdE*	Pyoverdine biosynthesis protein PvdE	PvdS	−13.51	
PA2398	*fpvA*	Ferripyoverdine receptor	FpvI, SigX	−8.13	
PA2402	*pvdI*	Probable nonribosomal peptide synthetase	PvdS, SigX, FpvI	−2.07	
PA2403	*fpvG*	FpvG	PvdS, SigX, FpvI	−2.90	
PA2404	*fpvH*	FpvH	PvdS, SigX, FpvI	−4.95	
PA2405	*fpvJ*	FpvJ	PvdS, SigX, FpvI	−5.52	
PA2406	*fpvK*	FpvK	PvdS, SigX, FpvI	−5.13	
PA2407	*fpvC*	FpvC	PvdS, SigX, FpvI	−5.71	
PA2408	*fpvD*	FpvD	PvdS, SigX, FpvI	−5.46	
PA2409	*fpvE*	FpvE	PvdS, SigX, FpvI	−5.99	
PA2410	*fpvF*	FpvF	PvdS, SigX, FpvI	−4.65	
PA2411		Probable thioesterase	PvdS	−5.10	
PA2412	*mbtH*	Conserved hypothetical protein	PvdS	−5.26	
PA2413	*pvdH*	l-2,4-Diaminobutyrate:2-ketoglutarate 4-aminotransferase, PvdH	PvdS	−3.85	
PA2424	*pvdL*	PvdL	PvdS, RpoS	−6.54	
PA2425	*pvdG*	PvdG	PvdS, RpoS	−6.67	
PA2426	*pvdS*	Sigma factor PvdS	Fur, RsmA, PvdS, OxyR	−33.33	
PA4168	*fpvB*	Second ferric pyoverdine receptor FpvB	Fur	−4.07	
Pyochelin					
PA4220	*fptB*	Hypothetical protein	Fur, RsmA, PchR	−6.99	
PA4221	*fptA*	Fe(III)-pyochelin OM receptor precursor	Fur, RsmA, PchR	−6.49	
PA4223	*pchH*	Probable ATP-binding component of ABC transporter	Fur, RsmA, PchR	−2.43	
PA4224	*pchG*	Pyochelin biosynthetic protein PchG	Fur, RsmA, PchR	−3.28	
PA4225	*pchF*	Pyochelin synthetase	Fur, RsmA, PchR	−4.07	
PA4226	*pchE*	Dihydroaeruginoic acid synthetase	Fur, RsmA, PchR	−5.71	
PA4227	*pchR*	Transcriptional regulator PchR	Fur, RsmA	−4.18	
PA4228	*pchD*	Pyochelin biosynthesis protein PchD	Fur, RsmA, PchR	−10.20	
PA4229	*pchC*	Pyochelin biosynthetic protein PchC	Fur, RsmA, PchR	−11.76	
PA4230	*pchB*	Salicylate biosynthesis protein PchB	Fur, RsmA, PchR	−12.50	
PA4231	*pchA*	Salicylate biosynthesis isochorismate synthase	Fur, RsmA, PchR	−10.53	
Virulence/biofilm switch					
GAC pathway					
PA0905	*rsmA*	RsmA	AlgU, AlgR, RsmY, RsmZ	−7.19	
PA0928	*gacS*	Sensor/response regulator hybrid		−2.24	
PA3345	*hptB*	Histidine phosphotransfer protein HptB		3.81	
PA3346	hsbR*	HptB-dependent secretion and biofilm regulator HsbR		2.34	
PA3347	hsbA*	HptB-dependent secretion and biofilm anti anti-sigma factor HsbA		2.29	
PA3621.1	*rsmZ*	Regulatory RNA RsmZ	GacA	−2.57	−5.26
Stress-related					
Stationary phase and general stress regulation					
PA3622	*rpoS*	Sigma factor RpoS		−16.13	−8.26
PPGPP metabolism					
PA5338	*spoT*	Guanosine-3′,5′-bis(diphosphate) 3′-pyrophosphohydrolase		−2.05	1.01
Envelope stress response					
PA0405	*algH*	AlgH		−2.27	
PA0762	*algU*	Sigma factor AlgU	AlgU	13.46	33.43
PA0763	*mucA*	Anti-sigma factor MucA	AlgU	9.89	
PA0764	*mucB*	Negative regulator for alginate biosynthesis MucB	AlgU	11.42	
PA0765	*mucC*	Positive regulator for alginate biosynthesis	AlgU	9.41	
PA1774	*cfrX*	CfrX protein	SigX	8.87	23.31
PA1775	*cmpX*	Cytoplasmic membrane protein, CmpX	SigX	9.91	28.06
PA1776	*sigX*	ECF sigma factor SigX	SigX	2.33	5.74
PA2895	*sbrR*	SbrR	SbrI	2.90	
PA2896	*sbrI*	SbrI ECF sigma	SbrI	5.00	13.26
PA3540	*algD*	GDP-mannose 6-dehydrogenase AlgD	AlgU, AmrZ, AlgR, RpoN, RsmA	13.26	39.34
PA3541	*alg8*	Alginate biosynthesis protein Alg8	AlgU, AmrZ AlgR, RpoN RsmA	20.92	
PA3545	*algG*	Alginate-c5-mannuronan-epimerase AlgG	AlgU, AmrZ AlgR, RpoN RsmA	2.31	
PA3546	*algX*	Alginate biosynthesis protein AlgX	AlgU, AmrZ AlgR, RpoN RsmA	2.79	
PA3550	*algF*	Alginate *O*-acetyltransferase AlgF	AlgU, AmrZ AlgR, RpoN RsmA	−2.83	
PA3551	*algA*	Phosphomannose isomerase/guanosine 5′-diphospho-d-mannose pyrophosphorylase	AlgU, AmrZ AlgR, RpoN RsmA	−2.48	
PA3649	*mucP*	MucP		2.77	
PA4033	*mucE*	MucE	AlgU	2.10	
PA5253	*algP*	Alginate regulatory protein AlgP		−9.90	
PA5255	*algQ*	Alginate regulatory protein AlgQ		−3.92	
PA5261	*algR*	Alginate biosynthesis regulatory protein AlgR	AlgU, RpoS	2.03	4.62
PA5262	*fimS*	FimS	AlgU	3.06	
PA5483	*algB*	Two-component response regulator AlgB	AlgU	3.70	

a *, Gene regulated by QS.

### Pf4* infection led to altered QS molecule production.

**FIG 4:**
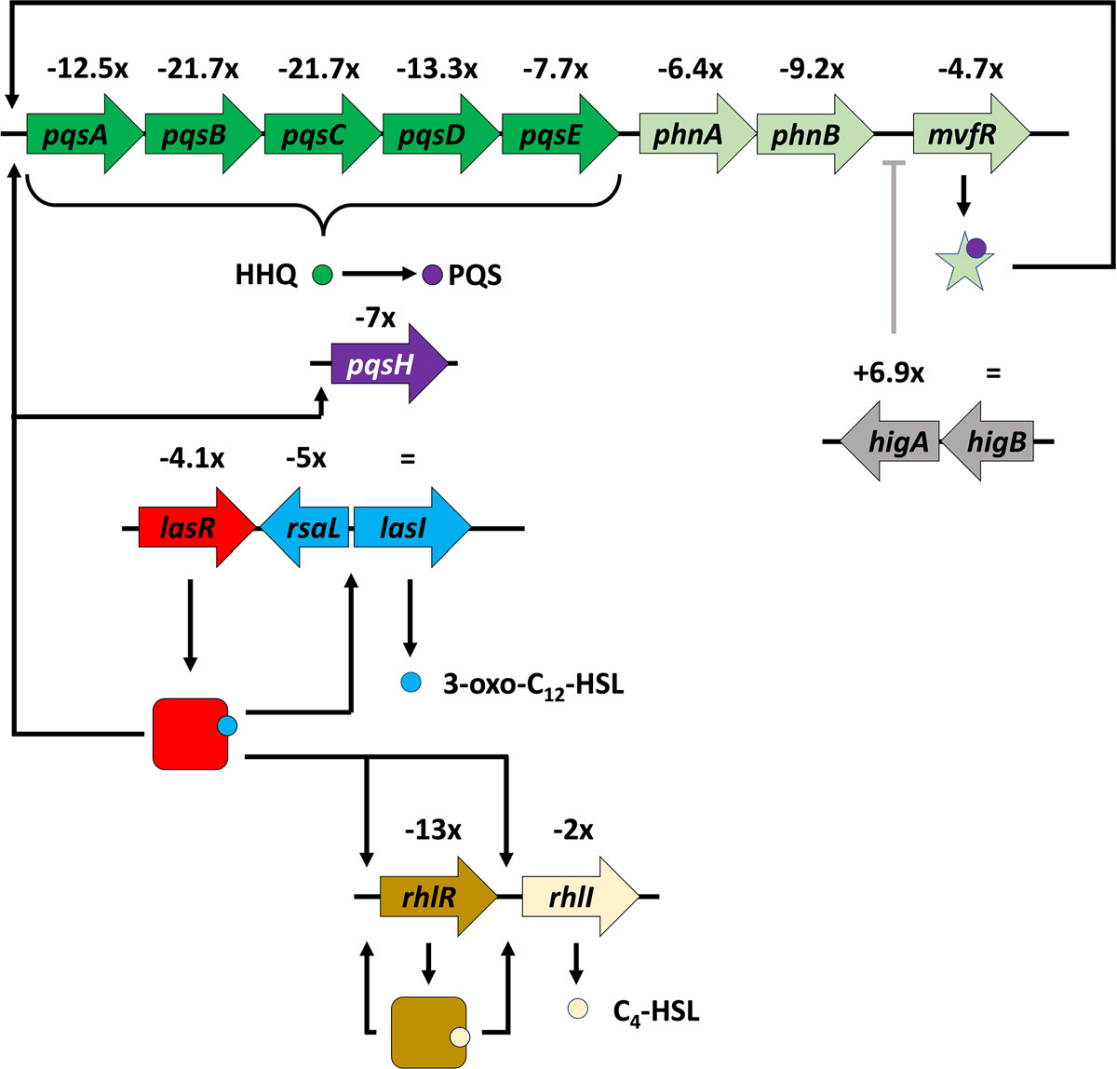
QS hierarchy. The LasI AHL synthase (blue arrow) produced the 3-oxo-C_12_-HSL (blue circle), which associated with the LasR LuxR regulator (in red arrow for *lasR* and red square for the LasR protein). LasR bound to 3-oxo-C_12_-HSL activates *rhlR* and *rhlI* (golden and beige arrows, respectively). RhlI produces C_4_-HSL (beige circle) and, after binding on the RhlR regulator (golden square), this system autoregulates itself. LasR bound to 3-oxo-C_12_-HSL activates the *pqsABCDE* operon (dark green arrows), as well as the *phnAB* genes (light green arrows). The product of these genes is HHQ (dark green circle), which is converted to PQS (violet circle) by the product of the *pqsH* gene (violet arrow), itself positively regulated by LasR. The MvfR regulator (light green star) binds PQS and activates several genes coding for virulence factors, including those for the biosynthesis of pyocyanin (not shown). Likewise, Las and Rhl contribute to the expression of virulence genes. The level of expression of each gene is indicated, and all values are negative except for *lasI* (unchanged). The HigA antitoxin gene is overexpressed in Pf4*-infected cells and has been shown to bind to the promoter region of the *mvfR* gene, inhibiting its transcription (128).

**FIG 5:**
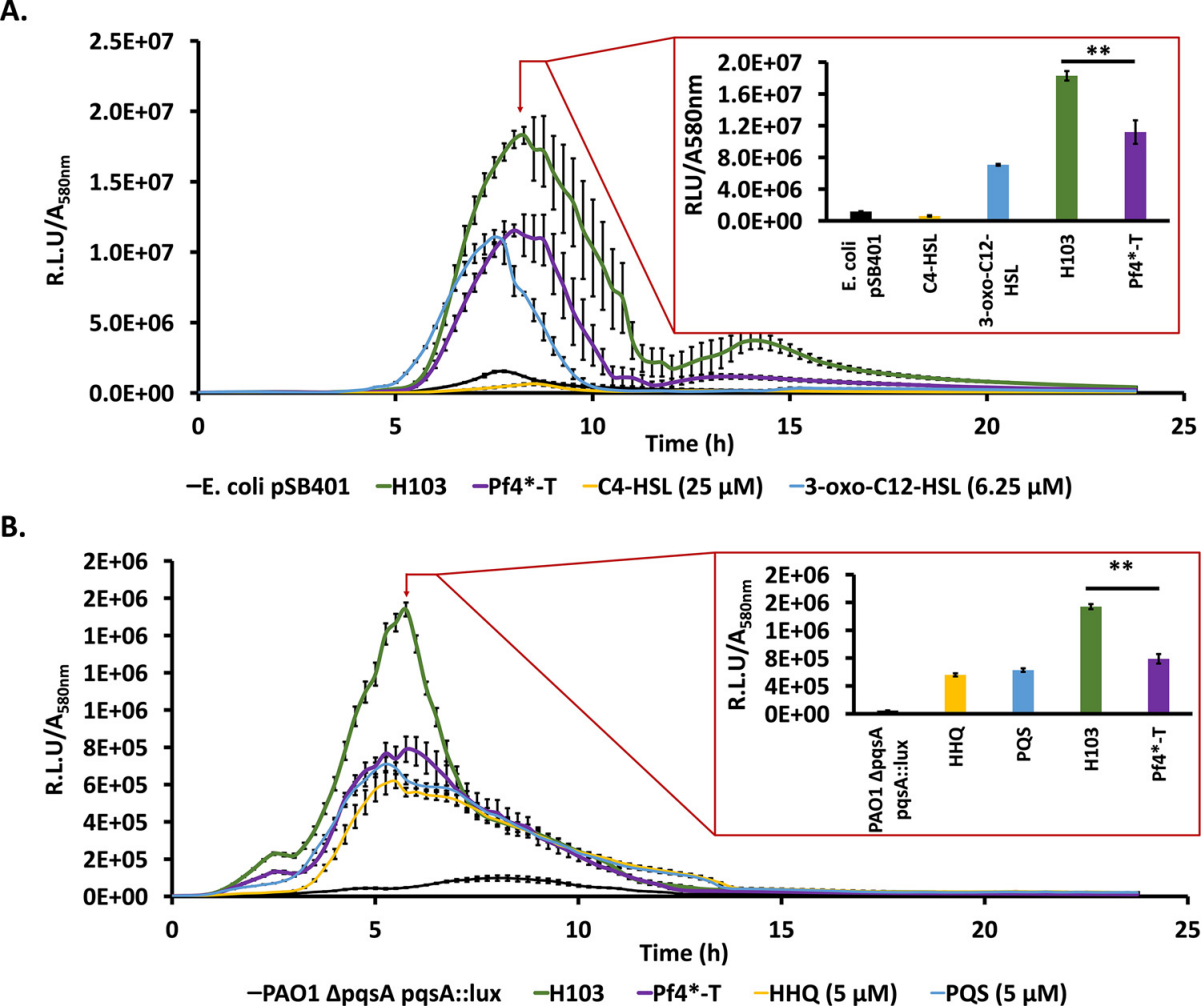
QS molecule production was altered after a Pf4 phage variant infection. (A) Bioluminescence measurements (± the SEM) normalized with *A*_580_ along the bacterial growth of the AHL bioreporter strain, E. coli pSB401, alone (negative control, black curve), in the presence of 3-oxo-C_12_-HSL (6.25 μM) or C_4_-HSL (25 μM) (positive controls, blue and yellow curves, respectively), and HSL extracts from the P. aeruginosa H103 (wild-type) condition (green curve) or the Pf4*-T condition (violet curve). The histogram represents all conditions at the peak of bioluminescence in the H103 condition (8 h, 15 min). (B) Bioluminescence measurements (± the SEM) normalized with *A*_580_ along the bacterial growth of the HAQ bioreporter strain, P. aeruginosa PAO1 Δ*pqsA pqsA*::*lux*, alone (negative control, black curve), in the presence of HHQ (5 μM) or PQS (5 μM) (positive controls, yellow and blue curves, respectively), and HAQ extracts from P. aeruginosa H103 (wild-type) condition (green curve) or Pf4*-T condition (violet curve). The histogram is a representation of all conditions at the peak of bioluminescence in the H103 condition (5 h, 45 min). Each experiment was assayed at least four times independently. Statistics were determined from values of bioluminescence peaks by a paired (two-sample) two-tailed *t* test (**, *P < *0.01).

The production of many virulence factors from P. aeruginosa depends on QS (61, 62), and numerous virulence factors encoding genes were strongly dysregulated upon Pf4* infection (see Table S1, virulence), suggesting that the QS pathways were affected. Autoinducer molecules produced by the three QS systems of P. aeruginosa accumulate depending on the cell density and associate with cognate activators to trigger the expression of virulence factor genes (15, 63). Two of the QS systems depend on *N*-acyl-homoserine lactones (AHLs) as signal molecules: 3-oxo-C_12_-HSL and C_4_-HSL for the Las and Rhl systems, respectively (15, 63). A third system relies on the production of two alkyl-quinolones, HHQ (2-heptyl-4-quinolone) and PQS (2-heptyl-3hydroxy-4-quinolone or Pseudomonas quinolone signal). The PQS system is interwoven with the Las and Rhl systems (15, 63) (Fig. 4). Looking at the transcripts from Pf4*-infected P. aeruginosa, the major QS regulator genes showed clearly decreased levels of expression, especially for *rhlR* (−13-fold) and *lasR* (−4.3-fold), although the transcription of the AHL synthase LasI is unaffected, while the level of *rhlI* is only 2-fold decreased. Production of AHL was assessed using Escherichia coli harboring the plasmid pSB401 (*luxRI′*::*luxCDABE*) biosensor strain, which is able to detect short (C_4_) and long (C_12_) HSL chains produced by P. aeruginosa either infected by Pf4* or not. As depicted in Fig. 5A, Pf4* infection reduced AHL production since a decrease of about 39% of bioluminescence was observed under this condition, showing that Pf4* infection interferes with AHL molecule production.

Many QS regulated genes are under the control of both LasR and RhlR regulators (64) (see Table S1, QS). Interestingly, the gene encoding the orphan LuxR repressor QscR, which was shown to interfere with the Rhl (>100 genes impacted) and the Las regulon (~70 genes) (65), shows a 13-fold upregulation. As previously mentioned, the ECF sigma factor AlgU was active in response to Pf4* infection and activates the transcription of *algR*, encoding a major repressor of Vfr. Accordingly, *algR* transcription was increased, while that of *vfr* was decreased in response to Pf4* infection (Table 1). Interestingly, AlgR was previously shown to repress the expression of *rhlI* and *rhlR* (66). Since Vfr was previously shown to directly activate transcription of *rhlR* (67, 68), one can assume that lower abundance levels of Vfr could also contribute to the decrease of *rhlR* transcription and of virulence on C. elegans (Fig. 2B) (69), which was observed upon Pf4* infection. In addition, the second key CESR sigma factor SigX that was highly active upon Pf4* infection was previously shown to cause an increased membrane fluidity under our conditions (31), which could possibly influence the production/diffusion of QS signal molecules. Indeed, a phospholipid *lptA* mutant induces membrane stiffness in P. aeruginosa, which results in strong and early production of the C_4_-HSL QS molecule (70). In addition, the production of the C_4_-HSL QS molecule was delayed, and the production of PQS was decreased in an *oprF* mutant, in which SigX was activated (71). It is therefore tempting to hypothesize that for the opposite situation, i.e., increased membrane fluidity due to SigX hyperactivity, the levels of C_4_-HSL would be decreased by an unknown mechanism in line with the results presented here.

HHQ and PQS are two alkyl quinolones synthesized by the *pqsABCDE* locus and the *phnAB* anthranilate synthase genes (72, 73) (Fig. 4), the expression of which was strongly decreased in Pf4*-treated bacteria (see Table S1, QS), suggesting that HHQ and PQS production may be impaired. HHQ and PQS production was assessed using the P. aeruginosa PAO1 Δ*pqsA* CTX-*lux*::*pqsA* biosensor strain, which is able to detect HAQ derivatives produced by P. aeruginosa either infected by Pf4* or not. As depicted in Fig. 5B, Pf4* infection resulted in reduced HAQ production since a decrease of about 49% of bioluminescence was measured under this condition, showing that Pf4* infection interferes with HAQ molecule production. The transcription of the *pqs* genes is under the control of the MvfR (PqsR) activator (74), whose transcription is strongly impaired under our conditions (see Table S1, QS). RhlR also binds upstream of *pqsA*, generating a longer transcript and a hairpin in the mRNA reducing *pqsABCDE* operon expression (74). Direct targets of LasR have been identified, uncluding *pqsA*, *mvfR*, and *pqsH* coding for a FAD-dependent monooxygenase responsible for the conversion of HHQ to PQS (Fig. 4, Table S1, QS) (64). The expression of *pqsH* is dependent on the neighboring AraC regulator encoding gene *cdpR*, which is also a direct target of LasR (75). Accordingly, in Pf4*-infected cells, the expression of *pqsH* and *cdpR* is decreased by 7- and 8-fold, respectively. In addition, anthranilate is the precursor in the biosynthesis of HHQ and PQS, as well a precursor of other alkyl-quinolones, and is synthesized by the PhnAB anthranilate synthase, whose genes are in the direct vicinity of the *pqsABCDE* operon (73, 76). However, anthranilate can also be provided by the catabolism of tryptophan via the kynurenine pathway (77). The KinU enzyme is responsible for the conversion of kynurenine to anthranilate (77, 78). In Pf4*-infected cells, both anthranilate biosynthesis pathways were affected with decreased *phnA* and *phnB* expression (−6.4- and −9.2-fold, respectively) and *kinU* expression (−4-fold), which should result in decreased availability of the anthranilate precursor for the synthesis of HHQ and hence PQS.

Why Pf4*-infected cells display impaired QS is not a trivial question. Interactions between phage proteins and QS systems in bacteria were recently reviewed (79), and QS may help bacteria to prevent phage predation. Indeed, it has been suggested that the induction of QS in Escherichia coli can help bacteria to defend themselves against λ phage by decreasing the adsorption of phages at the bacterial surface through lower production of the phage receptor LamB (80). Some clues suggest that PQS could be involved in the response against phage upon a bateriophage infection in P. aeruginosa (81, 82). A very recent work demonstrates that the DMS3 phage possesses a gene that encodes an anti-activator of QS in P. aeruginosa (83). This protein, named Aqs1, binds to LasR to inhibit its DNA-binding regulatory function, suggesting that DMS3 affects bacterial defense against phages through a QS-dependent mechanism (83). Interestingly, under our conditions, all genes involved in QS were underexpressed (see Table S1, QS, Fig. 4). It is tempting to hypothesize that, through a mechanism resembling that of DSM3 phage, Pf4 might encode a protein, which can interact with QS molecules and/or QS-encoded gene expression. Notably, PQS, but not HHQ, can bind Fe^3+^, causing iron limitation in cells exposed to PQS, although no siderophore activity could be demonstrated for PQS (14, 84). Because of its iron binding activity, the PQS regulon overlaps partially with the genes induced by iron scarcity (see Table S1, QS) (14, 84), suggesting that genes whose products are involved in iron capture may also be affected by Pf4* infection.

### Impact of Pf4* infection on iron uptake mechanisms.

**FIG 6:**
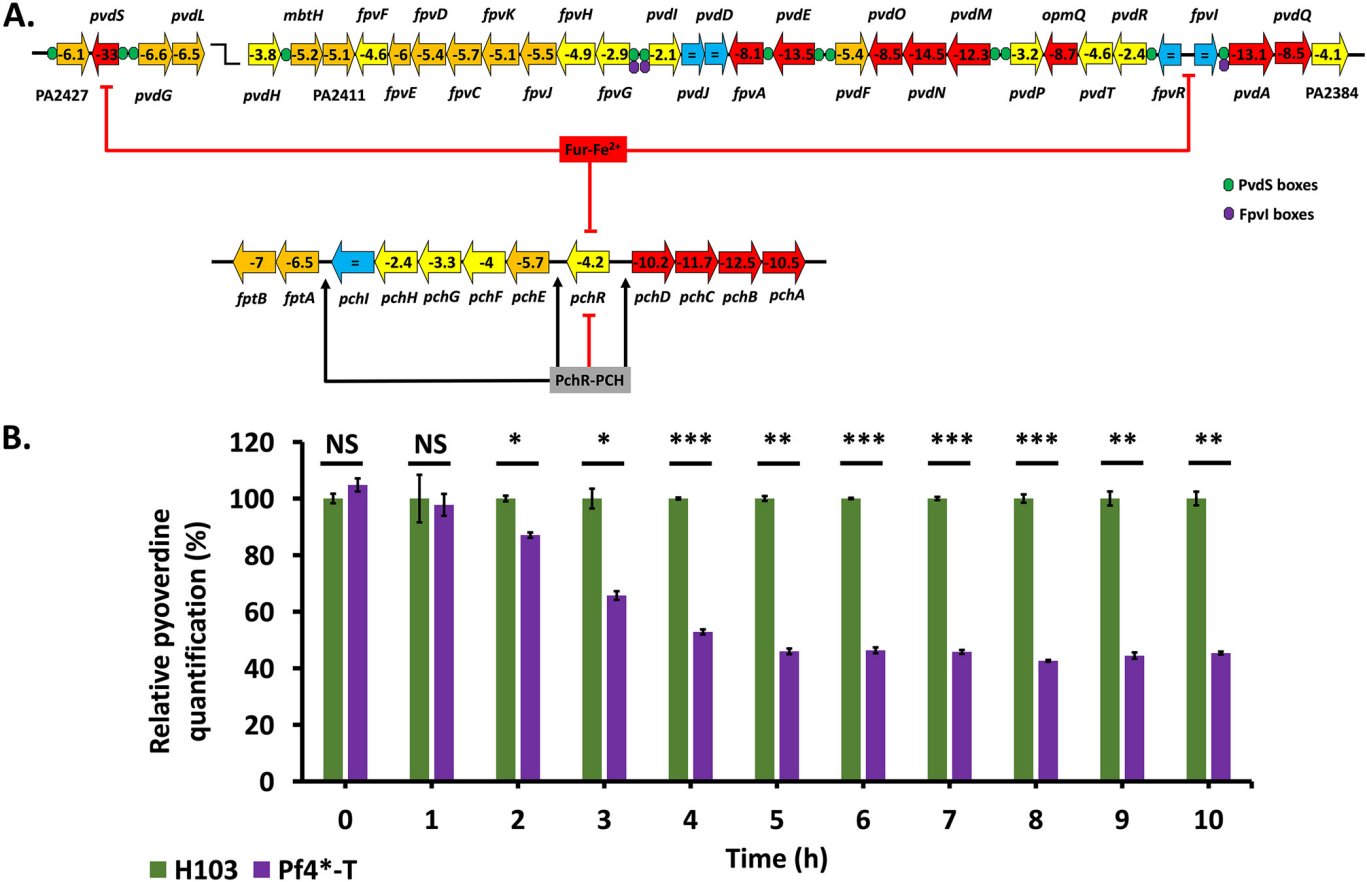
Pyoverdine production upon Pf4* infection. (A) Pyoverdine-encoding and pyochelin-encoding gene organization and regulation of the operons. The expression of each gene upon Pf4* infection is indicated inside arrows, and the colors indicate the following: red, >10-fold underexpressed; orange, downregulation between 5- and 10-fold; yellow, underexpression between 2- and 5-fold; and blue, not differentially expressed. PvdS binding sites are indicated by green circles, and FpvI binding sites are indicated by violet circles. The *pvdS*, *fpvR*, and *fpvI* genes are repressed by Fur-Fe^2+^, as well as the *pchR* gene. The product PchR, once bound to pyochelin (PCH), represses its own expression, whereas it activates the *fptAB*, *pchEFGHI*, and *pchDCBA* operons. See the text for more details. (B) Relative pyoverdine quantification (± the SEM) of the H103 (in green) and Pf4*-T (in purple) conditions in iron-poor medium (CAA). Pyoverdine quantifications were normalized with *A*_580_. Pyoverdine quantification was assayed three times independently. Statistics were determined using a paired (two-sample) two-tailed *t* test (NS, *P > *0.05; *, *P < *0.05; **, *P < *0.01; ***, *P < *0.001).

Iron is an essential element for bacteria and an important factor contributing to the virulence of bacterial pathogens since Fe is strongly sequestered by transferrin and lactoferrin in the host in a process termed “nutritional immunity” (85, 86). As in most bacteria, the expression of iron uptake systems is controlled by Fur (ferric uptake regulator). Fur exhibits regulatory activity once bound to its corepressor Fe^2+^. Under conditions of iron limitation, Fur is unable to exert its repressor activity, allowing the expression of iron uptake genes (87). Infection with Pf4* does not, however, cause a change in the level of *fur* transcripts. Under conditions of anaerobiosis, P. aeruginosa takes up the dominant and soluble form of Fe^2+^ via the Feo system combined with the redox cycling phenazines (8, 88). Pf4* infection causes a downregulation of the Fe^2+^ permease encoding *feoB* gene by a factor of 8. Under aerobic conditions and when available iron is limiting, P. aeruginosa produces and exports two siderophores, pyochelin (PCH) and pyoverdine (PVD) (89), and the genes encoding proteins of their biosynthetic pathways were strongly downregulated upon Pf4* infection (see Table S1, iron). PVD siderophore biosynthesis and uptake is indirectly regulated by Fur and directly by two extracytoplasmic sigma factors, PvdS for its biosynthesis and FpvI for the uptake of ferripyoverdine (Fe-PVD) via the TonB-dependent outer membrane transporter FpvA (90). PVD can be considered a virulence factor for two reasons: first, because it is essential to capture iron in the host (from lactoferrin and transferrin), and second, since the binding of Fe-PVD to the FpvA transporter triggers a transmembrane signaling system resulting in the production of two virulence factors, exotoxin A and PrpL (Piv) protease (90). Remarkably, the *pvdS* gene shows a 33-fold downregulation in Pf4*-infected cells with a concomitant decreased expression of all PVD biosynthesis genes (Fig. 6A). Despite the unchanged transcription level of the *fpvI* gene, the expression of *fpvA* is decreased 8.1-fold. The *fpvB* gene encoding a second Fe-PVD transporter (91) also shows a decreased transcription in Pf4*-infected cells (−4-fold). To confirm these data, PVD production was quantified under siderophore-inducing conditions, i.e., Casamino acid (CAA) medium depleted in iron. Under these conditions, P. aeruginosa produces less PVD upon Pf4* treatment all along the infected cells growth course compared to the untreated bacteria (Fig. 6B), suggesting that Pf4* infection interferes with PVD production or secretion (Table 1).

PCH is the other siderophore produced by P. aeruginosa, and its biosynthesis and uptake are regulated by PchR, an AraC regulator, which, when bound with PCH, activates the *pchDCBA* and the *pchEFGHI* operons for PCH biosynthesis (Fig. 6A) (92). PCH-Fe uptake is mediated by the FptA outer membrane TonB-dependent transporter (TBDT) and the Fpt inner membrane transporter (92). PchR-PCH acts as a repressor on its own *pchR* gene (92). During Pf4* infection, all *pch* operons and *pchR* gene expression are downregulated, with the *pchDCBA* genes showing the most significant decrease (>10-fold). Chorismate, the precursor of PCH biosynthesis, is converted to salicylate by the PchAB enzymes. As will be detailed below, chorismate is also a key precursor for the synthesis of tryptophan, and the PQS QS molecule.

Noticeably, the small noncoding RNA *prrF2* that is involved in iron metabolism was reduced in transription by >7-fold (see Table S1, iron). Since *prrF2* and the genes involved in pyochelin and pyoverdine biosynthesis are under the control of the major repressor Fur (12, 93), our data suggest that Fur is activated upon Pf4* treatment. PrrF1 and PrrF2, when expressed (under low-iron conditions), form a heteroduplex with the mRNA of the bacterioferritin gene *bfrB*, inhibiting its translation. Noticeably, *bfrB* transcripts are increased upon Pf4* infection (by a factor of 10). Interestingly, P. aeruginosa was shown to inhibit Candida albicans and Aspergillus fumigatus biofilm formation through the reduction of iron availability in the medium via the sequestration of iron by Pf4 phages (35, 40). Considering this, it is tempting to speculate that Pf4* phage may bring iron directly into the bacteria by the means of infection, thus avoiding the need for siderophore production. Another source of iron for P. aeruginosa is the heme molecule, which is present in the yeast extract from the Luria-Bertani (LB) medium. P. aeruginosa has three heme uptake systems involving TBDT: the Has, Phu, and Hxu systems (94). Of these three systems, only the *hxuA* gene encoding a TBDT for heme uptake is upregulated (5.9-fold), together with the ECF sigma factor gene *hxuI* (4.4-fold) and the gene *hxuR* coding for a transmembrane sensor (4.5-fold). Finally, an interesting link between H3-T6SS and iron has been described (95). In that study, the authors show that TseF (PA2374), an effector of H3-T6SS, binds PQS-Fe^3+^ and brings it to the FptA Fe-pyochelin transporter and to the OprF porin (95). H3-T6SS is regulated by both Fur and QS, and in Pf4*-infected cells, all H3-T6SS genes are strongly downregulated (see Table S1, virulence).

### Metabolism dysregulation could participate to the virulence-decrease after Pf4* infection.

**FIG 7:**
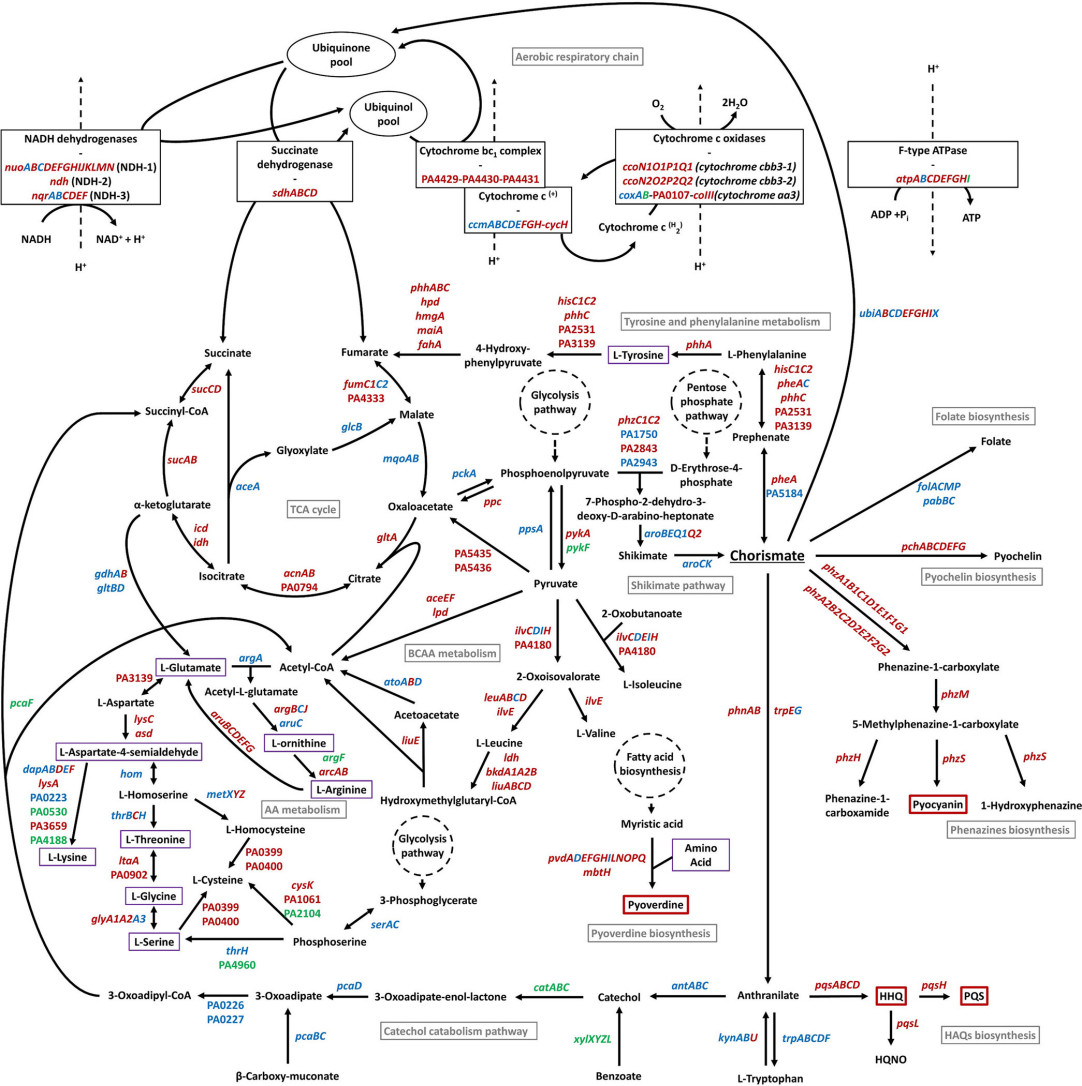
Chorismate pathway and central, energy, and amino acid metabolism are severely impacted upon Pf4* infection. Genes indicated in red, green, and blue were, respectively, downregulated, upregulated, and not differentially regulated in our transcriptomic study. Pathway names are presented in gray. Compounds surrounded by red were produced less under our conditions. Compounds surrounded in violet were involved in the biosynthesis of pyoverdine. AA, amino acid; BCAA, branched-chain amino acid; TCA, tricarboxylic acid.

As depicted in Fig. 1, several genes annotated in PseudoCAP (32) and involved in metabolism were affected. In support of the involvement of metabolism in virulence, several articles have shown that virulence factors production rely on metabolism in P. aeruginosa (96–100). The main pathways affected in response to Pf4* infection are depicted in Fig. 7.

**(i) Central metabolism.** Chorismate is a central compound involved in multiple metabolic pathways that is important to link metabolism to QS and virulence factor production and to the full virulence of P. aeruginosa (99). As depicted in Fig. 7, chorismate represents both the last product of the shikimate pathway and the precursor of several molecules belonging to (i) primary metabolism such as tyrosine, phenylalanine, and the tryptophan amino acids, folate, and ubiquinone, and (ii) secondary metabolism, as HAQs (through anthranilate), phenazines, and pyochelin. Genes involved in shikimate pathway do not seem particularly differentially expressed. Remarkably, all genes encoding enzymes involved in biosynthesis of secondary metabolism molecules from chorismate (including HAQs) were downregulated (Fig. 7; see also Table S1). Moreover, genes involved in tyrosine and phenylalanine biosynthesis from chorismate are also mostly underexpressed, as in the biosynthesis of quinones, which are important cofactors in the respiratory chain (Fig. 7; see also Table S1).

**FIG 8:**
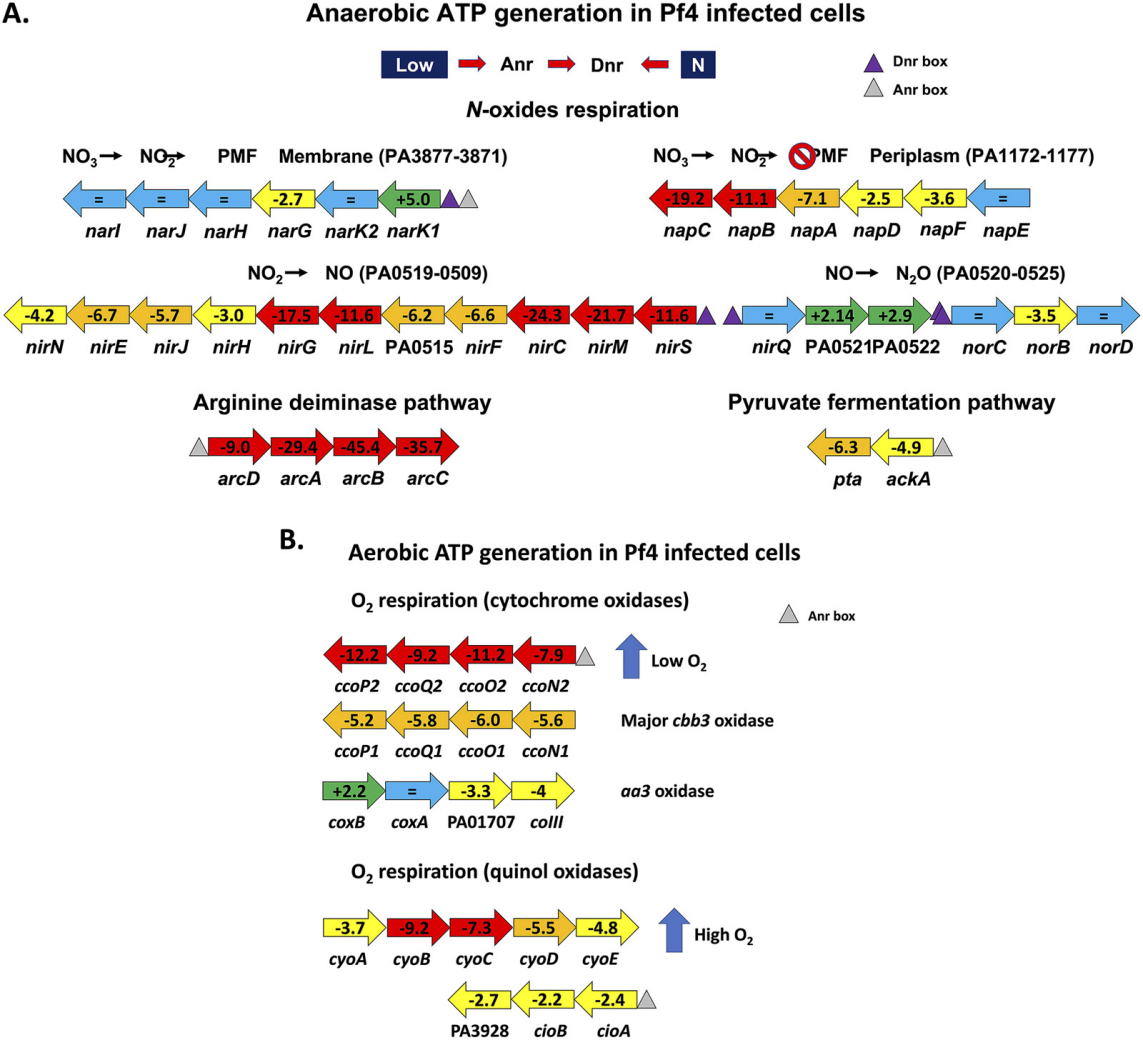
N-oxide respiration. (A) Anaerobic ATP generation in Pf4*-infected cells. The ANR and DNR regulators are shown at the top and are regulated by low O_2_ and NO, respectively. ANR and DNR binding sites are indicated as triangles. Unchanged gene transcriptions are shown as a blue arrow, increased transcription is shown as a green arrow, and decreased transcription is shown as yellow (−2 to −5×), orange (−5 to −10×), or red (>10× decreased) arrows. See the text for details. (B) Aerobic ATP generation in Pf4*-infected cells. The two *cco* low-oxygen-tension aerobic respiration operons are downregulated because of Pf4* infection with *ccoN2* operon was the most affected (the gray triangle represents an Anr regulator binding site). The *aa3* oxidase pathway (*cox* genes) is only mildly affected by the Pf4* infection. The quinol oxidase aerobic pathways represented by the *cyo* and *cio* genes and induced by high oxygen tension also show a decreased expression. See the text for details.

**(ii) Energy generation.** The PseudoCAP category with the highest proportion of downregulated genes is referring to energy metabolism (Fig. 1). The production of a high number of phage particles upon superinfection with the Pf4* variant certainly imposes a burden to the host cell, which is reflected in its way to energize the system via the generation of reductive power [NAD(P)H] and ATP. In P. aeruginosa, reductive power is generated by different types of dehydrogenases, resulting in the production of NAD(P)H and the transfer of electrons via a respiratory chain to a terminal electron acceptor: oxygen in the case of aerobic respiration or nitrate as an alternative acceptor under anaerobic conditions (101, 102). Next to the main respiratory chains involving electron transport chains, a limited fermentative capacity exists in P. aeruginosa involving pyruvate fermentation or the arginine deiminase pathway, but these alternative pathways only provide survival capacity in stationary phase (103–105). It has also been shown that phenazines (phenazine-1-carboxylic acid [PCA]) can act as electron shuttles outside the cell by being oxidized extracellularly and reused intracellularly, regenerating NAD during pyruvate and arginine fermentation (105). One of the striking consequences of the Pf4* infection is the strong downregulation of the anaerobic pathways for ATP generation in cultures infected by Pf4*, with the notable exception of the Nar dissimilatory nitrate reduction pathway (Fig. 8A). *N*-oxide respiration in P. aeruginosa involves different respiratory chains and terminal enzymes using NO_3_, NO_2_, N_2_O and NO as electron acceptors (106). Using an interactomic approach, the authors described the existence of a highly structured denitrification supercomplex termed respirasome. Figure 8A summarizes the changes in transcription levels of genes involved in N-oxides respiration following Pf4* infection. Although the membrane-bound dissimilatory nitrate reduction (*nar* genes) pathway seems relatively unaffected by the phage infection, it is interesting to note that the *narK1* gene encoding one of the two nitrite extrusion antiporter protein is upregulated, while the *nark2* gene transcription is unchanged, suggesting that the NO_2_ produced by the nitrate reductase is extruded to the periplasm. Interestingly, the *nap* genes encoding the periplasmic components of the second nitrate reductase are strongly downregulated with the exception of the *napE* gene. It is here worth noting that only the Nar system, but not the periplasmic Nap system, contributes to the energy generation via the establishment of a proton motive force (101). The anaerobic respirasome platform not only includes the proteins involved in the N-oxide respiration but also includes other components, such as the general Nuo dehydrogenases (PA2638 to PA2644) whose genes are strongly downregulated (Fig. 7; see also Table S1). A similar downregulation can be seen for more dedicated dehydrogenases encoding genes, such as the succinate dehydrogenase genes (Fig. 7; see also Table S1). More interesting still is the involvement of the interactome in the platform attachment of other proteins, such as the members of the Sec translocon (106).

Aerobic respiration is also branched in P. aeruginosa, involving five different terminal oxidases (101). Three of them are cytochrome oxidases, including two *cbb3* terminal oxidases, *ccoN1O1Q1P1* and *ccoN2O2Q2P2*, which differ in their affinity for O_2_, and one operon corresponds to an *aa3* oxidase (*cox* genes) (102) (Fig. 7 and Fig. 8B). The other two operons contain genes for cytochrome-independent quinol oxidases receiving their electrons directly from the quinone pool, *bo3* (*cyo* genes) and the cyanide-insensitive oxidase (*cio* genes) (101, 102). As can be seen from the data presented in Table S1, all aerobic respiration pathways are downregulated upon Pf4* infection, with the *aa3* oxidase being the least affected (Fig. 7 and Fig. 8B). In addition, the *atpABCDEFGHI* genes, encoding the F-type ATP synthase, and almost all genes encoding proteins involved in tricarboxylic acid (TCA) cycle were underexpressed, except those involved in the glyoxylate shunt and those encoding the malate:quinone oxidoreductases (*mqoA* and *mqoB*) (Fig. 7; see also Table S1). Two major regulators are involved in the control of the energy generation pathways: Anr and Dnr (102, 107). Anr (anaerobic regulator of arginine deiminase and nitrate reductase) is a sensor of oxygen tension via a [4Fe-4S]^2+^ cluster that binds upstream of the regulated genes such as *nar*, *arc*, and *ackA* (Fig. 8A) (107). In the presence of high O_2_ tension or NO, the iron-sulfur cluster is partly destroyed, and Anr becomes unable to activate its target genes. Anr sits upstream of *dnr* encoding a second regulator, which senses NO (107). The expression of both *anr* and *dnr* genes is lower in Pf4*-infected cells (−2.9-fold for *anr* and −7.2-fold for *dnr*). Dnr boxes are present in front of the *nar*, *nir*, and *nor* genes, while Anr boxes are found upstream of the *arc* and *ackA* genes (Fig. 8A). The Anr-regulated *oprG* gene, which encodes a small porin presumably involved in the uptake of Fe^2+^ under anaerobic conditions, also shows a strongly decreased transcription in phage-treated cells (−15.8-fold) (108) (see also the discussion of iron uptake above). Among other genes regulated by Anr are those encoding the so-called “universal stress proteins,” *uspK* (−9.7-fold in Pf4* infected cells), *uspL* (−8-fold), *uspO* (−13.8-fold), *uspM* (−3.3-fold), and *uspN* (−8.5-fold) (107). All *usp* genes are under the regulation of Anr, and their expression is downregulated in cells infected by Pf4* in line with the decreased expression of *anr* in the phage-infected cells (−2.9-fold). Of the 40 genes experimentally demonstrated to be Anr dependent (107), 28 are also underexpressed upon Pf4* infection (see Table S1).

**(iii) Amino acid metabolism.** Another PseudoCAP category with a high proportion of underexpressed genes is the amino acid metabolism category (Fig. 1). Genes involved in the metabolism of branched-chain amino acid (BCAA), including valine, leucine, and isoleucine, were largely underexpressed, as were tyrosine and phenylalanine, two aromatic amino acids derived from chorismate, as already mentioned. The same tendency has been be noted for the expression of genes coding for proteins involved in the biosynthesis pathways of threonine, glycine, serine, and cysteine (Fig. 7; see also Table S1).

Taken together, these data suggest that dysregulation at the expression level of genes whose products are involved in metabolic pathways can contribute to the global decrease of virulence observed under our conditions; this could minimize the impact that a slight upregulation of the T3SS may have. Several studies have established a link between metabolism and virulence of P. aeruginosa and led to the identification of metabolic pathways or key enzymes essential for virulence expression in this bacterium (96–100). Moreover, it is now well known that the virulence of a bacterium is dependent of the type of nutrients present in the environment (109–111). Phages rearrange the host metabolism to their own benefit (112), but the conclusions of these studies do not suggest a universal response to phage predation in bacteria at the metabolism or stress response levels. Moreover, there are a lot of studies that have been performed using virulent phages, but very few used filamentous phages, which can establish chronic and long-term infection of their hosts. Our transcriptomic study was made at 7 h postinfection, reflecting the adaptation of P. aeruginosa gene expression to Pf4* infection. This can explain the very high number of differentially expressed genes in our study compared to others (113–115). Some features in common with other studies have been observed, such as a significant underexpression of energy metabolism-encoding genes (Fig. 1, Table S1) often described after a phage infection (112, 115) or also of amino acid metabolism-encoding genes (Fig. 1; see also Table S1). In contrast, genes coding for ribosomal proteins or tRNA are overexpressed (see Table S1), as well as genes coding for proteins involved in carbon metabolism (Fig. 1). This last category of overexpressing genes could partly explain the relatively good growth of P. aeruginosa during Pf4* phage infection (31) despite the large number of underexpressed genes coding for proteins involved in metabolism. Even if alterations in gene expression can be the consequence of phage infection leading to reprogramming of the cell metabolism, we cannot exclude other explanations. Indeed, we previously demonstrated that a Pf4* infection leads to a cell wall stress response in P. aeruginosa mediated by AlgU and SigX (31). Notably, SigX increased activity led to a rise in membrane fluidity (31). Metabolic modifications were already described when membrane fluidity was altered (116, 117). Our transcriptomic study reveals many transporter-encoding genes that are differentially regulated at the expression level, as well as secretion system-encoding genes (see Table S1). Taken together, these data suggest that the significant dysregulation of metabolism at the gene expression level due (i) to the phage infection itself by reprogramming metabolism for its own benefits and (ii) to the membrane fluidity alteration via SigX activity that provoke transport alterations can participate in the decrease of P. aeruginosa virulence observed in our study. Overall, considering how many genes are changing in expression, one might think that it is the overall combinations that make the bacterium less fit and thus less virulent.

### Concluding remarks.

**FIG 9:**
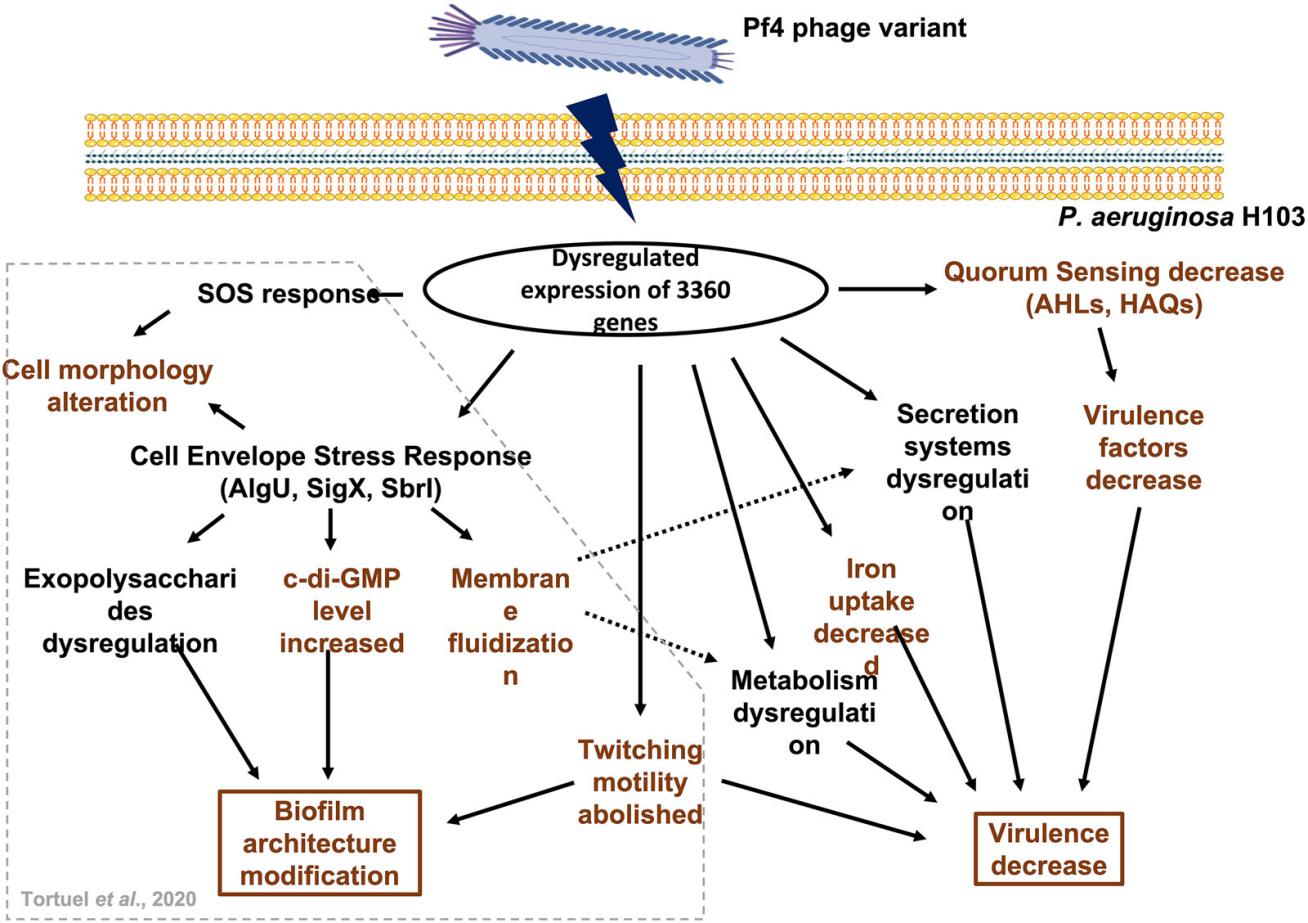
Adaptation of P. aeruginosa H103 to Pf4* infection. Broken lines arrows represent a suggested link. Color coding: brown, confirmed by experimental data; and black, suggested by the expression data. The data inside the gray box were presented previously by Tortuel et al. (31).

The behavior of P. aeruginosa facing Pf4* phage infection involves multiple alterations of regulatory and physiological circuits (Fig. 9). We previously showed that Pf4* phage infection results in an extended envelope stress response in P. aeruginosa mediated by the ECF sigma factors AlgU, SigX, and SbrI (31). This biological response leads to biofilm architecture modification through the dysregulation of exopolysaccharide-endoded gene expression and the increase of bis-(3′–5′)-cyclic dimeric GMP (31). Abolished twitching motility and cell morphology alterations (through the cell envelope stress and the SOS responses) also contribute to the modification of biofilm (31). We also described the fluidization of the membrane following Pf4* phage infection, probably through the increased activity of SigX (31). All gene expression alterations (found by RT-qPCR) seen in that earlier study were confirmed by the global transcriptomic study presented here. We also find that although planktonic growth is unaffected, the virulence of P. aeruginosa H103 is severely decreased upon Pf4* phage infection (Fig. 9). This reduction can be explained by multiple causes: (i) a decrease in virulence factors, such as elastase and pyocyanin, probably through a strong impairment of the QS regulatory network (Fig. 9); (ii) a decrease in siderophore production, as seen for pyoverdine and likely also pyochelin, suggesting that Pf4*-infected cells do not undergo iron deprivation (Fig. 9); (iii) a major metabolism modification that may be the result of phage infection by itself and an increase in membrane fluidity that can alter membrane trafficking (Fig. 9); (iv) a significant dysregulation in the expression of secretion systems (Fig. 9); and (v) an alteration in motility (Fig. 9). Considering the large number of dysregulated genes in response to Pf4* infection, it is possible that the slight upregulation of the T3SS would not be counted in terms of global virulence. Since we observed gene expression changes several hours after infection, the data presented here are the result of multiple primary and secondary effects. The original trigger is the Pf4* infection, but the changes observed in this study are the result of P. aeruginosa adaptation to this chronic infection. We provide here some clues about the adaptation of P. aeruginosa in response to a phage that establishes a dynamic chronic infection/interaction with its host, leading to dysregulation of multiple cellular processes associated with virulence and environmental fitness. Further work is needed to fully understand the interactions between bacteria and phages, especially the filamentous phages.

## MATERIALS AND METHODS

### Pf4* phage production.

Pf4* phages were obtained as previously described (31). Briefly, the screening of a transposon mutant library led to the identification of dH103Pf4^+^, a transposon mutant strain displaying a colony lysis phenotype and overproducing Pf4 phage variant (Pf4*). To obtain Pf4* phages, the dH103Pf4^+^ mutant strain was grown for 24 h at 37°C, and then 1 mL of the planktonic culture was harvested and centrifuged at 8,000 × *g* for 5 min. The supernatant was filtered (0.22-μm pore size) and stored at 4°C until use. For infection assays, Pf4* phage was added to the planktonic cultures at a final concentration of 1.5 × 10^9^ PFU mL^−1^.

### Bacterial strains, media, and growth conditions.

Bacterial strains used in this study are listed in Table S2 in the supplemental material. For planktonic cultures, P. aeruginosa H103 (117) was inoculated at an initial absorbance (*A*_580_) of 0.08 in LB medium containing 50 mM NaCl (31). Bacteria were grown at 37°C with orbital shaking at 180 r.p.m to their very early stationary phase (*A*_580_ = ~2.8), which was reached after 5 h (wild-type strain) or 7 h (Pf4*-T) (31). For pyoverdine quantification, cultures were performed in CAA medium (5 g L^−1^ Casamino Acids, 0.9 g L^−1^ KH_2_PO_4_, 0.25 g L^−1^ MgSO_4_**·**7H_2_O), followed by incubation at 37°C for 10 h at 180 rpm, and the pyoverdine concentration was measured as the *A*_405_ divided by the *A*_580_ as a measure of cell density.

### Total RNA extraction.

Total RNAs from Pf4*-treated and untreated P. aeruginosa cultures were extracted using the hot acid-phenol method (118). Genomic DNA contamination was removed using rigorous treatment with a RNA-free Turbo DNase I kit (Invitrogen, Carlsbad, CA) according to the manufacturer’s instructions. The RNA concentration was determined by using a NanoDrop ND-2000 spectrophotometer (Thermo Fisher Scientific, Waltham, MA), and quality was determined on an agarose gel (2%).

### RT-qPCR assays.

RT-qPCR experiments were performed as previously described (31). The primers used in this study are listed in Table S3.

### RNA-seq.

rRNA depletion, cDNA library preparation and Illumina sequencing were performed by ViroScan3D (Lyon, France). RNA samples were quantified using QuantiFluor RNA system (Promega, Madison, WI) and qualified using a fragment analyzer system (Agilent, Les Ulis, France). All RNA sample profiles were validated with an RNA IQ score of ≥8. Next, removal of 23S and 16S rRNAs was performed using a Ribo-Zero rRNA removal kit for Gram-negative bacteria (Illumina, San Diego, CA) according to the manufacturer’s instructions. At least 99% of the rRNA was removed from the total RNA, ensuring sufficient mRNA to be sequenced. After ribosomal depletion, libraries were generated by using a NextFlex rapid directional RNAseq kit for Illumina platforms (Perkin-Elmer, Waltham, MA). Briefly, the steps of fragmentation, first- and second-strand synthesis, adenylation, adapter ligation, and PCR amplification were performed to generate libraries for sequencing. The fragmented RNA samples were reverse transcribed to generate the first-strand synthesis. To retain the directionality, dUTP instead of dTTP was added during the second-strand synthesis. The purified second-strand synthesis DNA was 3′ adenylated, and the adapters were added and ligated the 3′ adenylated DNA. Different index primers were also used for the multiplexing step. Next, the purified adapter-ligated DNA and indexed sample were amplified by PCR to generate the libraries for sequencing. Uracil DNA glycosylase was incorporated into the PCR mixture to degrade the strand containing dUTP, allowing stranded sequencing. Library lengths were then quantified according to the Agilent HS NGS fragment kit protocol using the fragment analyzer system (Agilent). The libraries showed a mean size compatible with cluster generation of 380 bp. Thus, the validated libraries were loaded on a NextSeq High Output flowcell for cluster generation according to the standard Illumina protocol. Single-end run sequencing with a 75-bp read length was performed on a NextSeq sequencing system (Illumina, San Diego, CA) on three biological replicates of P. aeruginosa H103 cells infected or not with Pf4* phages. The main quality control parameters, including the number of reads generated, the quality of reads, the phasing/prephasing, and the error rate, passed the thresholds defined by Illumina.

### RNA-seq data analyses.

The RNA-seq data analyses started after the “base calling” step, performed during sequencing by using the NCS 1.3.0.26 and RTA 2.1.3 Illumina software suite implemented on a NextSeq Illumina sequencing machine. The format of data after this base calling is BCL (Base Call File). To get the number of raw reads per sample, the number of read passing filters (PF), the percentage of bases above Q30 (1 error out of 1,000 bases) among PF reads and the mean quality score, a demultiplexing step was assessed. This step, which consisted in attributing each read to the corresponding sample using the index sequence, was performed using bcl2fastq 2.17.1.14 from Illumina, allowing no mismatch. The format of data after demultiplexing was Fastq. Quality reports for the Fastq files were generated with the tool FastQC v0.11.8 (https://www.bioinformatics.babraham.ac.uk/projects/fastqc/). Low-quality bases and contaminant adapters were trimmed using Trimmomatic v0.38, using a minimum read length threshold of 50 bases (119, 120). The high-quality RNA-seq reads were mapped against the reference genome of P. aeruginosa PAO1 strain (GenBank assembly accession number GCA_000006765.1) using Boowtie2 alignment tool v2.3.4.1 with default parameters. Next, read mappings for each annotated coding sequence of P. aeruginosa PAO1 genome were counted using featureCounts v1.6.4 (120), and default parameters were used, except for the orientation parameter stranded, which was set to reverse. To determine the impact of Pf4* phage treatment on P. aeruginosa H103 gene expression, we compared the transcriptome of untreated versus Pf4*-treated bacteria. Analysis of differentially expressed genes (DEGs) was performed using the SARTools R package, including the DESeq2 package (121, 122). The analysis process included data normalization, graphical exploration of raw and normalized data, testing for differential expression for each feature between the conditions, and raw *P* value adjustment. Genes were considered significantly differentially expressed when the gene expression fold change (FC) was ≥2 (DEG upregulated) or was ≤−2 (DEG downregulated) and the *P* value (*P*_adj_) adjusted by the FDR (false discovery rate) is <0.05 (123). To validate the RNA-seq results, 48 DEGs were selected for expression level confirmation using RT-qPCR.

### Virulence factor quantification.

**(i) Elastase.** P. aeruginosa supernatants (50 μL) were mixed with 20 mg (±0.2) of elastase Congo red (Sigma-Aldrich, Saint-Louis, MO) and 1 mL of Tris buffer (100 mM Tris, 1 mM CaCl_2_ [pH 7.2]), followed by incubation for 18 h at 37°C with shaking. The reaction was stopped with 100 μL of EDTA, followed by centrifugation, and the absorbance of the supernatants was measured at 490 nm and normalized to the *A*_580_.

**(ii) Pyocyanin.** Pyocyanin was extracted from 1 mL of cell-free culture supernatants with 1 mL of chloroform by vortexing. The chloroform phase was extracted with 500 μL of 0.2 N HCl. The absorbance of the aqueous phase was measured at 520 nm and normalized to the *A*_580_ (49).

**(iii) Pyoverdine.** Pyoverdine was quantified by spectrophotometry from cells grown in CAA, and the results are expressed as the *A*_405_/*A*_580_ ratio (124).

### Belgian endives infection model.

The experimental procedure was performed as previously described (71), with few modifications. Leaves were infected with 10 μL of P. aeruginosa resuspended in 10 mM MgSO_4_ solution, treated or not with Pf4* (10^8^ CFU mL^−1^), and symptom development was inspected visually for 5 days. As a control, numerations were performed from the infection site of each leaf, and results were normalized to the endive weight (CFU g^−1^).

### *Caenorhabditis elegans* infection model.

Experimental procedures and data analysis were performed as previously described (33, 125). C. elegans wild-type Bristol strain N2 worms were grown at 22°C on nematode growth medium (NGM) agar plates using E. coli OP50 as the nutrient. Untreated or treated bacteria (10^9^ CFU mL^−1^) were spread onto NGM solidified agar plates before incubation at 37°C overnight. The plates were cooled to room temperature for 4 h, and 20 to 30 L4-synchronized worms were plated and incubated at 22°C in a humid environment to prevent plate drying. Worm survival was scored daily for 32 days using an Axiovert S100 optical microscope (Zeiss, Oberkochen, Germany) equipped with a digital camera (DXM 1200F; Nikon Instruments, Melville, NY). Four independent experiments per condition were performed, and all worms from each condition were used for the survival assay. The Kaplan-Meier method was used to calculate the nematode survival, and the significance of survival differences was tested using a log-rank test (Prism software, version 4.0; GraphPad Software, San Diego, CA). As a control to ascertain similar growth on NGM plates between treated and untreated bacteria, the NGM agar plates were entirely scraped every 5 days for enumeration on LB agar plates.

### Cytotoxicity assay on the A549 cell line.

The human lung A549 cells were cultured in Dulbecco modified Eagle medium (Lonza, BioWhittaker, Basel, Switzerland) supplemented with 4.5 g L^−1^ of glucose, 2 mM l-glutamine, 10% of heat-inactivated (30 min, 56°C) fetal bovine serum, and 100 U mL^−1^ of each antibiotic (penicillin and streptomycin). Cells were grown at 37°C under an atmosphere of 5% CO_2_ and 95% air with regularly medium changes until a confluent monolayer was obtained. The cytotoxicity of P. aeruginosa was assessed by a lactate dehydrogenase (LDH) assay (125), which is based on the quantification of the LDH release from damaged A549 cells. Briefly, confluent A549 monolayers were grown on 24-well tissue culture plates before being infected with treated or untreated P. aeruginosa cells (10^8^ CFU mL^−1^) for 20 h. Supernatants were then collected, and the LDH release was quantified according to the manufacturer’s instruction (Pierce LDH cytotoxicity assay kit ; Thermo Scientific, Waltham, MA) and normalized to the *A*_580_. A549 cells exposed to lysis buffer were used as a positive control for maximal LDH release (100% lysis), and the background level (0% LDH release) was determined with serum-free culture medium.

### Monitoring of T3SS activity.

Western blot analyses of T3SS α-PcrV in P. aeruginosa H103 were performed as previously described (126).

### Extraction and quantification of AHL and HAQ molecules.

AHL and HAQ extraction was performed as described previously (127). Quantification was assessed using Escherichia coli harboring plasmid pSB401 (*luxRI′*::*luxCDABE*) and P. aeruginosa PAO1 Δ*pqsA* CTX-*lux*::*pqsA* as biosensors (see Table S2), respectively, by a combined spectrophotometer/luminometer microplate assay. The biosensor strains were grown overnight, and the *A*_580_ was measured and adjusted to achieve an *A*_580_ value of 1. For each test well, 5 μL of crude extracts of QS molecules was diluted in 100 μL of LB medium before being added to 100 μL of a 1:50 dilution of the biosensor strains. Further, the bioluminescence and *A*_580_ were monitored every 15 min for 24 h at 37°C using a Spark 20M multimode microplate reader (Tecan, Männedorf, Switzerland) in white-sided and clear-bottom 96-well microtiter plates. The 3-oxo-C_12_-HSL, C_4_-HSL, HHQ, and PQS synthetic standards (Sigma-Aldrich, Saint-Louis, MO) at final concentrations of 5 μM were added to a 1:100 dilution of the biosensor strains as positive controls. The bioluminescence, recorded as relative light units (RLU), was normalized to the *A*_580_.

### Statistical analyses.

Unless indicated otherwise, data were statistically analyzed using a two-sample paired two-sided *t* test to calculate *P* values with GraphPad Prism. All values are reported and plotted as means ± the standard errors of the mean (SEM) based on at least triplicate analyses for each experimental variable (NS, *P > *0.05; *, *P < *0.05; **, *P < *0.01; ***, *P < *0.001; ****, *P < *0.0001).

### Data availability.

The RNA-seq data were deposited under GEO accession no. GSE201738.
