# Supplementary material for: Pf4 Phage Variant Infection Reduces Virulence-Associated Traits in Pseudomonas aeruginosa
Source: Microbiol Spectr. 2022 Aug 29;10(5):e01548-22. doi: 10.1128/spectrum.01548-22 (PMC9602442; doi:10.1128/spectrum.01548-22)
Supplement: Supplemental file 1 — Fig. S1 and S2 and Tables S1 to S3. Download spectrum.01548-22-s0001.pdf, PDF file, 2.5 MB [file spectrum.01548-22-s0001.pdf]

Supplemental Material

Pf4 phage variant infection reduces virulence-associated traits in *Pseudomonas aeruginosa*

Tortuel Damien[1,2], Tahrioui Ali[1,2], David Audrey[1,2], Cambronel Mélyssa[1,2], Nilly Flore[1,2], Clamens Thomas[3], Maillot Olivier[1,2], Barreau Magalie[1,2], Feuilloley Marc GJ[1,2], Lesouhaitier Olivier[1,2], Filloux Alain[3], Bouffartigues Emeline[1,2], Cornelis Pierre[1,2,4], Chevalier Sylvie[1,2*]

[1]Unité de recherche Communication Bactérienne et Stratégies Anti-infectieuses, CBSA UR4312, Université de Rouen Normandie, Évreux, France.

[2]Normandie Université, Fédération de Recherche SéSAD, Université de Rouen Normandie,

[3]MRC Centre for Molecular Bacteriology and Infection, Department of Life Sciences, Imperial College London, SW7 2AZ London, United Kingdom.

[4]Laboratorium Microbiologie, Vrije Universiteit Brussel (VUB), Belgium.

*Corresponding Author : Pr. Sylvie Chevalier, 55 rue Saint Germain, 27000 Evreux, France. Tel: +33-235140000; Fax: +33-232291550; sylvie.chevalier@univ-rouen.fr

SUPPLEMENTARY FIGURES

Supplementary Figure S1
Supplementary Figure S2

SUPPLEMENTARY TABLES

Supplementary Table S1
Supplementary Table S2
Supplementary Table S3

SUPPLEMENTARY REFERENCES

SUPPLEMENTARY FIGURES

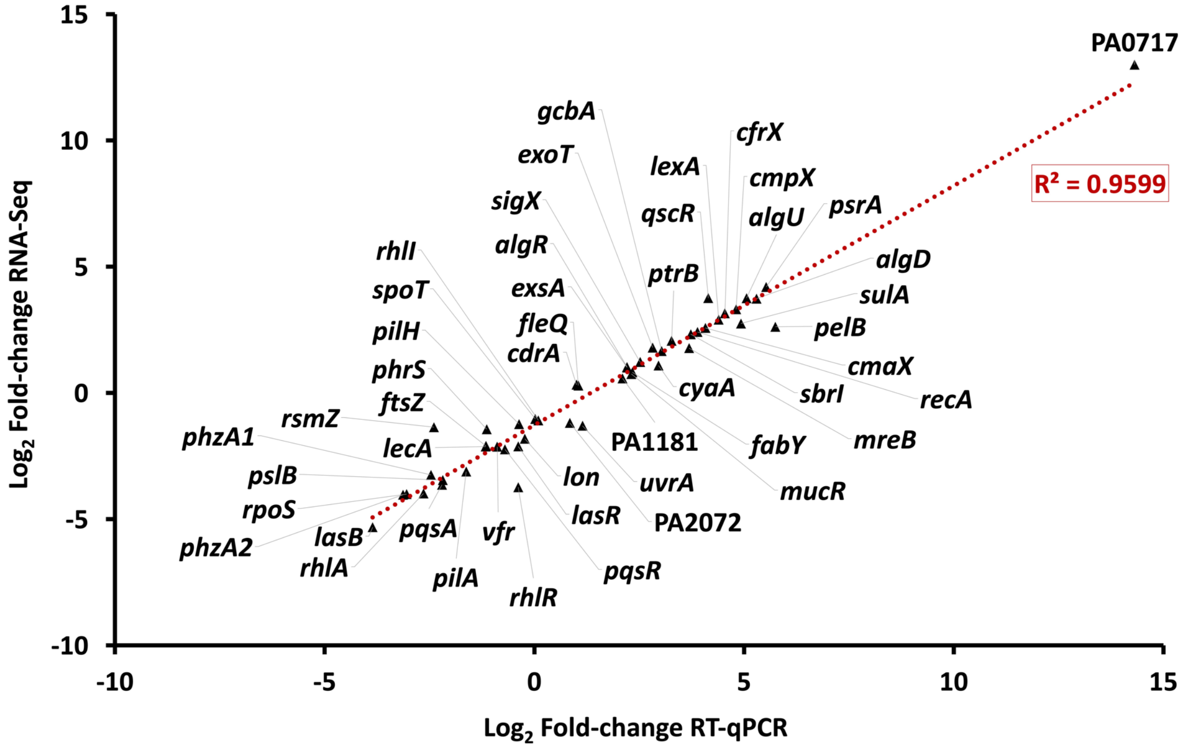

Supplementary Figure S1. Correlation between the log2 fold change of several genes in RNA-seq and RT-qPCR data.

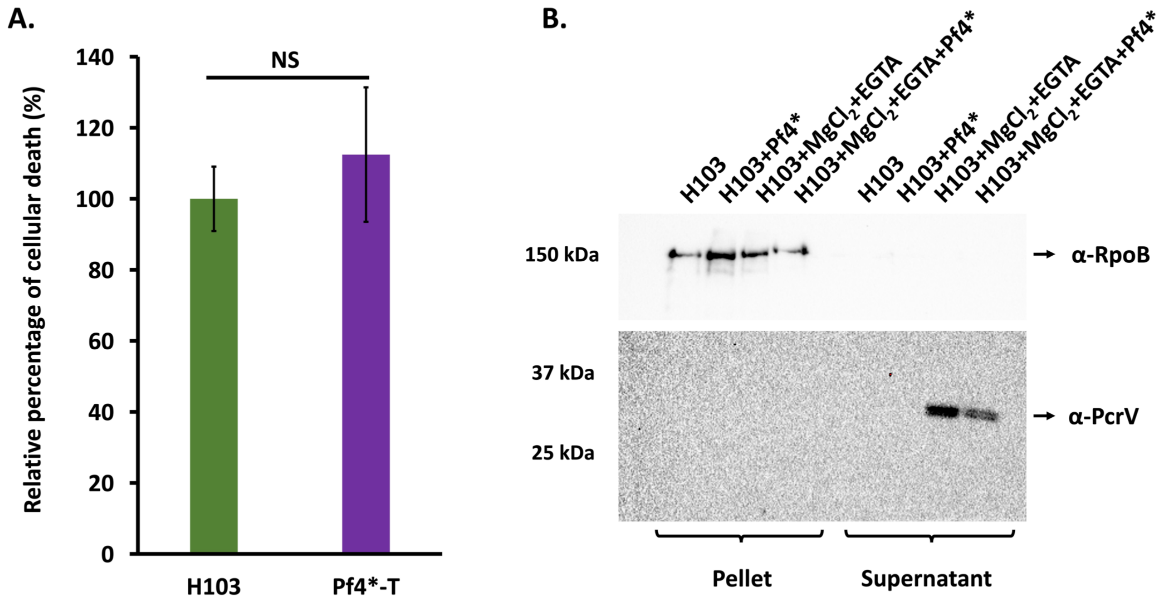

Supplementary Figure S2. Pf4*-infection did not lead to T3SS activity alterations. **A.** Cytotoxity of Pf4*-treated or untreated P. aeruginosa H103 on A549 lung cells. Statistics were achieved by paired (two samples) two-tailed t-test. ^{NS} $p > 0.05$. **B.** Functionality of the T3SS assessed by western blot analysis of pellets using **α**-RpoB antibodies to evaluate the bacterial amount, and of cell supernatants using **α**-PcrV antibodies to detect presence of the secreted **α**-PcrV protein. **α**-PcrV was detected in similar amounts in Pf4*- treated or untreated P. aeruginosa H103 when treated with $MgCl_2$ and EGTA.

SUPPLEMENTARY TABLES

Supplementary Table S1. List of genes under and overexpressed after Pf4 phage variant treatment compared to H103 (*p*-value ≤ 0.05 and fold-change ≤ -2 or ≥ 2), classified by functions, PA number, related to iron, QS or virulence factors.

Classification by functions

Functional groups	Function	PA number	Gene name	Product name	Fold-change	PseudoCAP1	PseudoCAP2	PseudoCAP3
Secretion Systems	Sec Secretion System	PA3820	*secF*	secretion protein SecF	-3.27	Protein secretion/export apparatus		
		PA3821	*secD*	secretion protein SecD	-3.52	Protein secretion/export apparatus	Membrane proteins	
		PA3822	*yajC*	conserved hypothetical protein	-2.66	Hypothetical, unclassified, unknown		
		PA4403	*secA*	secretion protein SecA	-2.96	Protein secretion/export apparatus		
		PA4747	*secG*	secretion protein SecG	3.28	Membrane proteins	Protein secretion/export apparatus	
		PA5128	*secB*	secretion protein SecB	-3.66	Protein secretion/export apparatus		
	Tat Secretion System	PA5068	*tatA*	translocation protein TatA	-2.23	Protein secretion/export apparatus		
		PA5069	*tatB*	translocation protein TatB	-2.54	Protein secretion/export apparatus		
		PA5070	*tatC*	transport protein TatC	-2.70	Membrane proteins	Transport of small molecules	
	T1SS-Apr	PA1245	*aprX*	AprX	-3.94	Secreted Factors (toxins, enzymes, alginate)	Membrane proteins	
		PA1246	*aprD*	alkaline protease secretion protein AprD	-4.15	Secreted Factors (toxins, enzymes, alginate)	Protein secretion/export apparatus	
		PA1247	*aprE*	alkaline protease secretion protein AprE	-7.09	Secreted Factors (toxins, enzymes, alginate)	Protein secretion/export apparatus	
		PA1248	*aprF*	Alkaline protease secretion outer membrane protein AprF precursor	-10.53	Secreted Factors (toxins, enzymes, alginate)	Protein secretion/export apparatus	
		PA1249	*aprA*	alkaline metalloproteinase precursor	-45.45	Secreted Factors (toxins, enzymes, alginate)		
		PA1250	*aprI*	alkaline proteinase inhibitor AprI	-4.12	Secreted Factors (toxins, enzymes, alginate)		
	T2SS-Hxc	PA0677	*hxcW*	HxcW	3.89	Protein secretion/export apparatus		
		PA0678	*hxcU*	HxcU	2.81	Protein secretion/export apparatus		
		PA0679	*hxcP*	HxcP	2.60	Hypothetical, unclassified, unknown		
		PA0680	*hxcV*	HxcV	5.09	Protein secretion/export apparatus		
		PA0681	*hxcT*	HxcT	10.55	Protein secretion/export apparatus		
		PA0682	*hxcX*	HxcX	25.02	Protein secretion/export apparatus		
		PA0683	*hxcY*	HxcY	6.09	Protein secretion/export apparatus		
		PA0684	*hxcZ*	HxcZ	10.33	Protein secretion/export apparatus		
		PA0685	*hxcQ*	HxcQ	13.41	Protein secretion/export apparatus		
		PA0686	*hxcR*	HxcR	11.11	Protein secretion/export apparatus		
		PA0687	*hxcS*	HxcS	18.82	Protein secretion/export apparatus		
	T2SS-Xcp	PA3095	*xcpZ*	general secretion pathway protein M	-3.27	Protein secretion/export apparatus		
		PA3096	*xcpY*	general secretion pathway protein L	-3.13	Protein secretion/export apparatus		
		PA3097	*xcpX*	general secretion pathway protein K	-2.00	Protein secretion/export apparatus		
		PA3098	*xcpW*	general secretion pathway protein J	-5.35	Protein secretion/export apparatus		
		PA3099	*xcpV*	general secretion pathway protein I	-9.17	Protein secretion/export apparatus		
		PA3100	*xcpU*	General secretion pathway outer membrane protein H precursor	-15.87	Protein secretion/export apparatus		
		PA3101	*xcpT*	general secretion pathway protein G	-17.24	Protein secretion/export apparatus		
		PA3102	*xcpS*	general secretion pathway protein F	-3.57	Protein secretion/export apparatus		
		PA3103	*xcpR*	general secretion pathway protein E	-3.77	Protein secretion/export apparatus		
		PA3105	*xcpQ*	general secretion pathway protein D	-2.55	Protein secretion/export apparatus		
	T2SS-Xcp-Related genes	PA1867	*xphA*	XphA	6.62	Protein secretion/export apparatus		
		PA1868	*xqhA*	secretion protein XqhA	6.92	Protein secretion/export apparatus		
	T2SS-Xcp-Secreted factors	PA0026	*plcB*	phospholipase C, PlcB	-6.06	Hypothetical, unclassified, unknown		
		PA0572	*impA*	hypothetical protein	-27.03	Hypothetical, unclassified, unknown		
		PA0843	*plcR*	phospholipase accessory protein PlcR precursor	4.81	Secreted Factors (toxins, enzymes, alginate)		
		PA0844	*plcH*	hemolytic phospholipase C precursor	2.94	Secreted Factors (toxins, enzymes, alginate)		
		PA0852	*cbpD*	chitin-binding protein CbpD precursor	-33.33	Secreted Factors (toxins, enzymes, alginate)		
		PA2862	*lipA*	lactonizing lipase precursor	2.02	Secreted Factors (toxins, enzymes, alginate)	Carbon compound catabolism	
		PA2939	*paaP*	probable aminopeptidase	-50.00	Secreted Factors (toxins, enzymes, alginate)		
		PA3296	*phoA*	alkaline phosphatase	2.95	Central intermediary metabolism		
		PA3319	*plcN*	non-hemolytic phospholipase C precursor	8.68	Secreted Factors (toxins, enzymes, alginate)		
		PA3910	*eddA*	Extracelullar DNA degradation protein, EddA	3.91	Secreted Factors (toxins, enzymes, alginate)	Nucleotide biosynthesis and metabolism	
		PA4175	*piv*	protease IV	-29.41	Secreted Factors (toxins, enzymes, alginate)		
		PA4813	*lipC*	lipase LipC	6.38	Fatty acid and phospholipid metabolism		
	T3SS	PA1690	*pscU*	translocation protein in type III secretion	7.95	Protein secretion/export apparatus		
		PA1691	*pscT*	translocation protein in type III secretion	13.76	Protein secretion/export apparatus		
		PA1692	*pscS*	probable translocation protein in type III secretion	6.42	Protein secretion/export apparatus		
		PA1693	*pscR*	translocation protein in type III secretion	5.37	Protein secretion/export apparatus		
		PA1694	*pscQ*	translocation protein in type III secretion	-2.01	Protein secretion/export apparatus		
		PA1696	*pscO*	translocation protein in type III secretion	2.59	Protein secretion/export apparatus		
		PA1697	*pscN*	ATP synthase in type III secretion system	3.40	Protein secretion/export apparatus		
		PA1698	*popN*	Type III secretion outer membrane protein PopN precursor	2.14	Protein secretion/export apparatus	Membrane proteins	
		PA1699	*pcr1*	Pcr1	2.46	Protein secretion/export apparatus	Secreted Factors (toxins, enzymes, alginate)	
		PA1700	*pcr2*	Pcr2	2.43	Protein secretion/export apparatus	Secreted Factors (toxins, enzymes, alginate)	
		PA1701	*pcr3*	Pcr3	5.04	Protein secretion/export apparatus		
		PA1702	*pcr4*	Pcr4	3.84	Protein secretion/export apparatus	Secreted Factors (toxins, enzymes, alginate)	
		PA1703	*pcrD*	type III secretory apparatus protein PcrD	2.48	Protein secretion/export apparatus		
		PA1705	*pcrG*	regulator in type III secretion	4.22	Protein secretion/export apparatus		
		PA1713	*exsA*	transcriptional regulator ExsA	2.00	Protein secretion/export apparatus	Transcriptional regulators	
		PA1714	*exsD*	ExsD	2.84	Hypothetical, unclassified, unknown		
		PA1715	*pscB*	type III export apparatus protein	9.61	Protein secretion/export apparatus		
		PA1716	*pscC*	Type III secretion outer membrane protein PscC precursor	9.26	Protein secretion/export apparatus		
		PA1717	*pscD*	type III export protein PscD	11.51	Protein secretion/export apparatus		
		PA1718	*pscE*	type III export protein PscE	3.76	Protein secretion/export apparatus	Chaperones & heat shock proteins	
		PA1719	*pscF*	type III export protein PscF	2.06	Protein secretion/export apparatus		
		PA1721	*pscH*	type III export protein PscH	2.22	Protein secretion/export apparatus		
		PA1722	*pscI*	type III export protein PscI	2.33	Protein secretion/export apparatus		
		PA1723	*pscJ*	type III export protein PscJ	2.74	Protein secretion/export apparatus		
		PA1724	*pscK*	type III export protein PscK	2.67	Protein secretion/export apparatus		
		PA1725	*pscL*	type III export protein PscL	2.79	Protein secretion/export apparatus		
	T3SS-Secreted factors	PA0044	*exoT*	exoenzyme T	3.47	Secreted Factors (toxins, enzymes, alginate)		
		PA2191	*exoY*	adenylate cyclase ExoY	3.53	Secreted Factors (toxins, enzymes, alginate)		
		PA3842	*spcS*	specific Pseudomonas chaperone for ExoS, SpcS	-4.29	Secreted Factors (toxins, enzymes, alginate)	Protein secretion/export apparatus	Chaperones & heat shock proteins
	T3SS-Regulation	PA0612	*ptrB*	repressor, PtrB	4.17	Transcriptional regulators		
		PA2486	*ptrC*	Pseudomonas type III repressor gene C, PtrC	5.69	Hypothetical, unclassified, unknown		
		PA2808	*ptrA*	Pseudomonas type III repressor A	11.12	Adaptation, Protection	Transcriptional regulators	
		PA3006	*psrA*	transcriptional regulator PsrA	18.28	Transcriptional regulators		
		PA4916	*nrtR*	Nudix-related transcriptional regulator NrtR	3.90	Hypothetical, unclassified, unknown		
		PA4917	*nadD2*	nicotinate mononucleotide adenylyltransferase NadD2	5.03	Hypothetical, unclassified, unknown		
	T5aSS	PA5112	*estA*	esterase EstA	-2.68	Fatty acid and phospholipid metabolism	Secreted Factors (toxins, enzymes, alginate)	Protein secretion/export apparatus
	T5bSS	PA4541	*lepA*	Pseudomonas aeruginosa-derived large extracellular protease, LepA	3.83	Membrane proteins	Secreted Factors (toxins, enzymes, alginate)	
	T5dSS	PA3339	*plpD*	patatin-like protein, PlpD	2.62	Membrane proteins	Secreted Factors (toxins, enzymes, alginate)	
	H1-T6SS	PA0074	*ppkA*	serine/threonine protein kinase PpkA	-2.13	Translation, post-translational modification, degradation	Adaptation, Protection	Protein secretion/export apparatus
		PA0078	*tssL1*	TssL1	-2.83	Protein secretion/export apparatus		
		PA0079	*tssK1*	TssK1	-2.42	Protein secretion/export apparatus		
		PA0080	*tssJ1*	TssJ1	-2.04	Protein secretion/export apparatus		
		PA0084	*tssC1*	TssC1	-2.13	Protein secretion/export apparatus		
	H2-T6SS	PA1657	*hsiB2*	HsiB2	-2.29	Protein secretion/export apparatus		
		PA1658	*hsiC2*	HsiC2	-3.38	Protein secretion/export apparatus		
		PA1659	*hsiF2*	HsiF2	-3.55	Protein secretion/export apparatus		
		PA1660	*hsiG2*	HsiG2	-4.65	Protein secretion/export apparatus		
		PA1661	*hsiH2*	HsiH2	-3.30	Protein secretion/export apparatus		
		PA1662	*clpV2*	clpV2	-2.99	Protein secretion/export apparatus	Putative enzymes	
		PA1663	*sfa2*	Sfa2	-3.24	Protein secretion/export apparatus	Transcriptional regulators	
		PA1664	*orfX*	OrfX	-3.61	Protein secretion/export apparatus		
		PA1665	*fha2*	Fha2	-5.24	Protein secretion/export apparatus		
		PA1666	*lip2*	Lip2	-5.00	Protein secretion/export apparatus		
		PA1667	*hsiJ2*	HsiJ2	-3.85	Protein secretion/export apparatus		
		PA1668	*dotU2*	DotU2	-2.62	Protein secretion/export apparatus		
	H3-T6SS	PA2359	*sfnR2*	probable transcriptional regulator	2.54	Transcriptional regulators		
		PA2360	*hsiA3*	hypothetical protein	-11.49	Hypothetical, unclassified, unknown		
		PA2361	*icmF3*	IcmF3	-2.62	Hypothetical, unclassified, unknown		
		PA2363	*hsiJ3*	HsiJ3	-3.85	Hypothetical, unclassified, unknown		
		PA2365	*hsiB3*	HsiB3	-35.71	Hypothetical, unclassified, unknown		
		PA2366	*hsiC3*	HsiC3	-47.62	Hypothetical, unclassified, unknown		
		PA2367	*hcp3*	Hcp3	-55.56	Hypothetical, unclassified, unknown		
		PA2368	*hsiF3*	HsiF3	-66.67	Hypothetical, unclassified, unknown		
		PA2369	*hsiG3*	HsiG3	-32.26	Membrane proteins		
		PA2370	*hsiH3*	HsiH3	-41.67	Hypothetical, unclassified, unknown		
		PA2371	*clpV3*	ClpV3	-25.64	Translation, post-translational modification, degradation		
		PA2372		hypothetical protein	-17.24	Hypothetical, unclassified, unknown		
		PA2373	*vgrG3*	VgrG3	-7.46	Hypothetical, unclassified, unknown		
		PA2374	*tseF*	TseF	-7.58	Hypothetical, unclassified, unknown		
	T6SS-Associated genes	PA1512	*hcpA*	secreted protein Hcp	-2.00	Secreted Factors (toxins, enzymes, alginate)		
		PA1844	*tse1*	Tse1	2.06	Secreted Factors (toxins, enzymes, alginate)		
		PA2685	*vgrG4*	VgrG4	2.63	Protein secretion/export apparatus		
		PA2703	*tsi2*	Tsi2	-2.79	Adaptation, Protection		
		PA2774	*tse4*	Tse4	2.76	Membrane proteins	Secreted Factors (toxins, enzymes, alginate)	
		PA2775	*tsi4*	Tsi4	3.38	Membrane proteins	Adaptation, Protection	
		PA3291	*tli1*	Tli1	-2.64	Adaptation, Protection		
		PA3294	*vgrG4a*	VgrG4a	-2.34	Hypothetical, unclassified, unknown		
		PA3485	*tsi3*	Tsi3	-3.24	Adaptation, Protection		
		PA3486	*vgrG4b*	VgrG4b	-2.28	Hypothetical, unclassified, unknown		
		PA3487	*tle5*	Tle5	-3.09	Fatty acid and phospholipid metabolism	Secreted Factors (toxins, enzymes, alginate)	
		PA3488	*tli5*	Tli5	-2.40	Adaptation, Protection		
		PA5086	*tli5b1*	type VI secretion lipase immunity protein, Tli5b1	4.55	Hypothetical, unclassified, unknown		
		PA5088	*tli5b3*	type VI secretion lipase immunity protein, Tli5b3	-2.45	Hypothetical, unclassified, unknown		
		PA5089	*tle5b*	type VI secretion phospholipase D effector Tle5b	-2.36	Secreted Factors (toxins, enzymes, alginate)		
		PA5090	*vgrG5*	VgrG5	-2.04	Hypothetical, unclassified, unknown		
		PA5266	*vgrG6*	VgrG6	-2.90	Hypothetical, unclassified, unknown		
		PA5267	*hcpB*	secreted protein Hcp	-3.36	Secreted Factors (toxins, enzymes, alginate)		
	Probable type II secretion system	PA2672		probable type II secretion system protein	10.73	Protein secretion/export apparatus		
		PA2673		probable type II secretion system protein	4.05	Protein secretion/export apparatus		
		PA2674		probable type II secretion system protein	2.81	Protein secretion/export apparatus		
		PA2675		probable type II secretion system protein	2.78	Protein secretion/export apparatus		
		PA2676		probable type II secretion system protein	2.40	Protein secretion/export apparatus		
		PA2677		probable type II secretion protein	2.21	Protein secretion/export apparatus		
	Others	PA1382		probable type II secretion system protein	3.88	Protein secretion/export apparatus		
		PA3221	*csaA*	CsaA protein	-2.49	Protein secretion/export apparatus	Chaperones & heat shock proteins	
		PA3360		probable secretion protein	8.54	Secreted Factors (toxins, enzymes, alginate)		
		PA4142		probable secretion protein	-2.60	Protein secretion/export apparatus		
		PA4143		probable toxin transporter	-2.03	Protein secretion/export apparatus	Membrane proteins	Transport of small molecules
		PA4144		probable outer membrane protein precursor	-3.88	Protein secretion/export apparatus		
		PA4974		probable outer membrane protein precursor	-3.51	Protein secretion/export apparatus		
		PA5210		probable secretion pathway ATPase	-3.68	Protein secretion/export apparatus		
Quorum Sensing	Las system	PA1430	*lasR*	transcriptional regulator LasR	-4.35	Adaptation, Protection	Transcriptional regulators	
		PA1431	*rsaL*	regulatory protein RsaL	-5.03	Adaptation, Protection	Transcriptional regulators	
	Rhl system	PA3476	*rhlI*	autoinducer synthesis protein RhlI	-2.13	Adaptation, Protection		
		PA3477	*rhlR*	transcriptional regulator RhlR	-13.33	Transcriptional regulators		
	Pqs system	PA0996	*pqsA*	PqsA	-12.50	Biosynthesis of cofactors, prosthetic groups and carrier		
		PA0997	*pqsB*	PqsB	-21.74	Biosynthesis of cofactors, prosthetic groups and carrier		
		PA0998	*pqsC*	PqsC	-21.74	Biosynthesis of cofactors, prosthetic groups and carrier		
		PA0999	*pqsD*	3-oxoacyl-[acyl-carrier-protein] synthase III	-13.33	Biosynthesis of cofactors, prosthetic groups and carrier		
		PA1000	*pqsE*	Quinolone signal response protein	-7.75	Biosynthesis of cofactors, prosthetic groups and carrier		
		PA1001	*phnA*	anthranilate synthase component I	-6.41	Adaptation, Protection		
		PA1002	*phnB*	anthranilate synthase component II	-9.17	Amino acid biosynthesis and metabolism	Adaptation, Protection	
		PA1003	*mvfR*	Transcriptional regulator MvfR	-4.72	Biosynthesis of cofactors, prosthetic groups and carrier	Transcriptional regulators	
		PA2587	*pqsH*	probable FAD-dependent monooxygenase	-7.04	Biosynthesis of cofactors, prosthetic groups and carrier		
		PA4190	*pqsL*	probable FAD-dependent monooxygenase	-9.52	Putative enzymes		
	QS regulators	PA0714.1	*phrD*	PhrD	14.75	Non-coding RNA gene		
		PA1032	*quiP*	QuiP	-3.12	Antibiotic resistance and susceptibility	Putative enzymes	Carbon compound catabolism
		PA1244	*qslA*	QslA	-5.68	Hypothetical, unclassified, unknown		
		PA1898	*qscR*	quorum-sensing control repressor	13.46	Transcriptional regulators		
		PA2226	*qsrO*	QsrO	-9.17	Hypothetical, unclassified, unknown		
		PA2227	*vqsM*	AraC-type transcriptional regulator VqsM	-3.76	Transcriptional regulators		
		PA3305.1	*phrS*	PhrS	-2.72	Non-coding RNA gene		
Virulence Factors	Rhamnolipids biosynthesis	PA1130	*rhlC*	rhamnosyltransferase 2	-10.87	Cell Wall / LPS / Capsule	Adaptation, Protection	
		PA3478	*rhlB*	rhamnosyltransferase chain B	-22.22	Secreted Factors (toxins, enzymes, alginate)		
		PA3479	*rhlA*	rhamnosyltransferase chain A	-15.87	Secreted Factors (toxins, enzymes, alginate)		
	Lectins	PA2570	*lecA*	LecA	-4.37	Cell Wall / LPS / Capsule	Adaptation, Protection	Motility and Attachment
		PA3361	*lecB*	fucose-binding lectin PA-IIL	-10.53	Motility and Attachment		
	Elastases	PA1871	*lasA*	LasA protease precursor	-62.50	Translation, post-translational modification, degradation	Secreted Factors (toxins, enzymes, alginate)	
		PA3724	*lasB*	elastase LasB	-40.00	Translation, post-translational modification, degradation	Secreted Factors (toxins, enzymes, alginate)	
	Pyocyanin biosynthesis	PA0051	*phzH*	potential phenazine-modifying enzyme	-8.40	Putative enzymes		
		PA1899	*phzA2*	probable phenazine biosynthesis protein	-16.39	Secreted Factors (toxins, enzymes, alginate)		
		PA1900	*phzB2*	probable phenazine biosynthesis protein	-37.04	Secreted Factors (toxins, enzymes, alginate)		
		PA1901	*phzC2*	phenazine biosynthesis protein PhzC	-16.39	Secreted Factors (toxins, enzymes, alginate)		
		PA1902	*phzD2*	phenazine biosynthesis protein PhzD	-17.24	Secreted Factors (toxins, enzymes, alginate)		
		PA1903	*phzE2*	phenazine biosynthesis protein PhzE	-19.23	Secreted Factors (toxins, enzymes, alginate)		
		PA1904	*phzF2*	probable phenazine biosynthesis protein	-21.28	Secreted Factors (toxins, enzymes, alginate)		
		PA1905	*phzG2*	probable pyridoxamine 5'-phosphate oxidase	-23.81	Secreted Factors (toxins, enzymes, alginate)		
		PA4209	*phzM*	probable phenazine-specific methyltransferase	-2.39	Putative enzymes		
		PA4210	*phzA1*	probable phenazine biosynthesis protein	-9.52	Secreted Factors (toxins, enzymes, alginate)		
		PA4211	*phzB1*	probable phenazine biosynthesis protein	-6.41	Secreted Factors (toxins, enzymes, alginate)		
		PA4212	*phzC1*	phenazine biosynthesis protein PhzC	-13.16	Secreted Factors (toxins, enzymes, alginate)		
		PA4213	*phzD1*	phenazine biosynthesis protein PhzD	-19.61	Secreted Factors (toxins, enzymes, alginate)		
		PA4214	*phzE1*	phenazine biosynthesis protein PhzE	-19.61	Secreted Factors (toxins, enzymes, alginate)		
		PA4215	*phzF1*	probable phenazine biosynthesis protein	-21.74	Secreted Factors (toxins, enzymes, alginate)		
		PA4216	*phzG1*	probable pyridoxamine 5'-phosphate oxidase	-23.26	Secreted Factors (toxins, enzymes, alginate)		
		PA4217	*phzS*	flavin-containing monooxygenase	-6.67	Putative enzymes		
	HCN biosynthesis	PA2193	*hcnA*	hydrogen cyanide synthase HcnA	-8.33	Central intermediary metabolism		
		PA2194	*hcnB*	hydrogen cyanide synthase HcnB	-9.35	Central intermediary metabolism		
		PA2195	*hcnC*	hydrogen cyanide synthase HcnC	-12.20	Central intermediary metabolism		
	Exotoxin A regulation	PA0707	*toxR*	transcriptional regulator ToxR	-3.04	Transcriptional regulators		
		PA2258	*ptxR*	transcriptional regulator PtxR	11.78	Transcriptional regulators		
	Others	PA0041		probable hemagglutinin	2.92	Secreted Factors (toxins, enzymes, alginate)		
		PA0122	*rahU*	rahU	-26.32	Adaptation, Protection		
		PA0253	*hudR*	HudR	2.80	Transcriptional regulators		
		PA0254	*hudA*	HudA	4.30	Hypothetical, unclassified, unknown		
		PA0423	*pasP*	PasP	-8.85	Secreted Factors (toxins, enzymes, alginate)		
		PA2432	*bexR*	bistable expression regulator, BexR	2.50	Transcriptional regulators		
		PA2899	*atvR*	atypical virulence-related response regulator AtvR	2.72	Transcriptional regulators		
		PA2931	*cifR*	CifR	3.34	Transcriptional regulators		
		PA4370	*icmP*	Insulin-cleaving metalloproteinase outer membrane protein precursor	-13.89	Membrane proteins		
		PA4487	*magF*	MagF	-4.76	Hypothetical, unclassified, unknown		
		PA4491	*magB*	MagB	-2.34	Hypothetical, unclassified, unknown		
		PA4493	*roxR*	RoxR	-4.88	Two-component regulatory systems	Transcriptional regulators	
Biofilm-Virulence switch	Chp pathway	PA0413	*chpA*	component of chemotactic signal transduction system	-2.25	Two-component regulatory systems	Motility and Attachment	Chemotaxis
		PA0414	*chpB*	probable methylesterase	-2.82	Chemotaxis		
		PA0417	*chpE*	probable chemotaxis protein	2.84	Chemotaxis		
		PA0652	*vfr*	transcriptional regulator Vfr	-4.39	Transcriptional regulators		
		PA5272	*cyaA*	adenylate cyclase	2.12	Adaptation, Protection		
	Gac pathway	PA0905	*rsmA*	RsmA	-7.19	Translation, post-translational modification, degradation	Adaptation, Protection	
		PA0928	*gacS*	sensor/response regulator hybrid	-2.24	Two-component regulatory systems		
		PA3345	*hptB*	Histidine phosphotransfer protein HptB	3.81	Two-component regulatory systems	Motility and Attachment	
		PA3346	*hsbR*	HptB-dependent secretion and biofilm regulator HsbR	2.34	Two-component regulatory systems	Transcriptional regulators	
		PA3347	*hsbA*	HptB-dependent secretion and biofilm anti anti-sigma factor HsbA	2.29	Two-component regulatory systems	Motility and Attachment	
		PA3621.1	*rsmZ*	regulatory RNA RsmZ	-2.57	Non-coding RNA gene		
Biofilm matrix Exopolysaccharides	Pel biosynthesis	PA3058	*pelG*	PelG	4.08	Cell Wall / LPS / Capsule		
		PA3059	*pelF*	PelF	2.35	Cell Wall / LPS / Capsule		
		PA3060	*pelE*	PelE	2.72	Cell Wall / LPS / Capsule		
		PA3061	*pelD*	PelD	2.67	Cell Wall / LPS / Capsule		
		PA3062	*pelC*	PelC	2.63	Cell Wall / LPS / Capsule		
		PA3063	*pelB*	PelB	6.14	Cell Wall / LPS / Capsule		
		PA3064	*pelA*	PelA	6.55	Cell Wall / LPS / Capsule		
	Psl biosynthesis	PA2232	*pslB*	PslB	-10.99	Cell Wall / LPS / Capsule		
		PA2233	*pslC*	PslC	-13.70	Cell Wall / LPS / Capsule	Putative enzymes	
		PA2234	*pslD*	PslD	-18.52	Cell Wall / LPS / Capsule	Transport of small molecules	
		PA2235	*pslE*	PslE	-18.18	Cell Wall / LPS / Capsule		
		PA2236	*pslF*	PslF	-21.74	Cell Wall / LPS / Capsule		
		PA2237	*pslG*	PslG	-12.82	Cell Wall / LPS / Capsule	Putative enzymes	
		PA2238	*pslH*	PslH	-15.38	Cell Wall / LPS / Capsule		
		PA2239	*pslI*	PslI	-10.64	Cell Wall / LPS / Capsule	Putative enzymes	
		PA2240	*pslJ*	PslJ	-7.41	Cell Wall / LPS / Capsule		
		PA2241	*pslK*	PslL	-5.52	Cell Wall / LPS / Capsule	Membrane proteins	
		PA2242	*pslL*	hypothetical protein	-15.87	Hypothetical, unclassified, unknown		
		PA2243	*pslM*	hypothetical protein	-2.28	Hypothetical, unclassified, unknown		
		PA2244	*pslN*	hypothetical protein	2.32	Hypothetical, unclassified, unknown		
		PA2245	*pslO*	hypothetical protein	2.50	Hypothetical, unclassified, unknown		
	Alginates biosynthesis	PA3540	*algD*	GDP-mannose 6-dehydrogenase AlgD	13.26	Secreted Factors (toxins, enzymes, alginate)	Cell Wall / LPS / Capsule	Adaptation, Protection
		PA3541	*alg8*	alginate biosynthesis protein Alg8	20.92	Secreted Factors (toxins, enzymes, alginate)	Cell Wall / LPS / Capsule	Adaptation, Protection
		PA3545	*algG*	alginate-c5-mannuronan-epimerase AlgG	2.31	Secreted Factors (toxins, enzymes, alginate)	Cell Wall / LPS / Capsule	Adaptation, Protection
		PA3546	*algX*	alginate biosynthesis protein AlgX	2.79	Secreted Factors (toxins, enzymes, alginate)	Cell Wall / LPS / Capsule	Adaptation, Protection
		PA3550	*algF*	alginate o-acetyltransferase AlgF	-2.83	Secreted Factors (toxins, enzymes, alginate)	Cell Wall / LPS / Capsule	Adaptation, Protection
		PA3551	*algA*	phosphomannose isomerase / guanosine 5'-diphospho-D-mannose pyrophosphorylase	-2.48	Secreted Factors (toxins, enzymes, alginate)	Cell Wall / LPS / Capsule	Adaptation, Protection
	Alginates regulation	PA0405	*algH*	AlgH	-2.27	Transcriptional regulators		
		PA0762	*algU*	sigma factor AlgU	13.46	Transcriptional regulators		
		PA0763	*mucA*	anti-sigma factor MucA	9.89	Cell Wall / LPS / Capsule	Transcriptional regulators	
		PA0764	*mucB*	negative regulator for alginate biosynthesis MucB	11.42	Cell Wall / LPS / Capsule	Transcriptional regulators	
		PA0765	*mucC*	positive regulator for alginate biosynthesis MucC	9.41	Cell Wall / LPS / Capsule	Transcriptional regulators	Secreted Factors (toxins, enzymes, alginate)
		PA3649	*mucP*	MucP	2.77	Hypothetical, unclassified, unknown		
		PA4033	*mucE*	MucE	2.10	Hypothetical, unclassified, unknown		
		PA5253	*algP*	alginate regulatory protein AlgP	-9.90	Transcriptional regulators		
		PA5255	*algQ*	Alginate regulatory protein AlgQ	-3.92	Transcriptional regulators		
		PA5261	*algR*	alginate biosynthesis regulatory protein AlgR	2.03	Two-component regulatory systems	Transcriptional regulators	Secreted Factors (toxins, enzymes, alginate)
		PA5262	*fimS*	FimS	3.06	Two-component regulatory systems	Motility and Attachment	
		PA5483	*algB*	two-component response regulator AlgB	3.70	Two-component regulatory systems	Transcriptional regulators	
	Type IVa pilus biogenesis	PA0395	*pilT*	twitching motility protein PilT	-3.17	Cell Wall / LPS / Capsule	Motility and Attachment	
		PA0396	*pilU*	twitching motility protein PilU	-3.26	Motility and Attachment		
		PA0408	*pilG*	twitching motility protein PilG	-2.00	Two-component regulatory systems	Motility and Attachment	Chemotaxis
		PA0409	*pilH*	twitching motility protein PilH	-2.36	Two-component regulatory systems	Motility and Attachment	Chemotaxis
		PA0410	*pilI*	twitching motility protein PilI	-2.86	Motility and Attachment	Chemotaxis	
		PA0411	*pilJ*	twitching motility protein PilJ	-4.08	Motility and Attachment	Chemotaxis	
		PA0412	*pilK*	methyltransferase PilK	-3.77	Motility and Attachment	Chemotaxis	
		PA1822	*fimL*	hypothetical protein	-2.33	Motility and Attachment		
		PA4525	*pilA*	type 4 fimbrial precursor PilA	-8.70	Motility and Attachment		
		PA4526	*pilB*	type 4 fimbrial biogenesis protein PilB	-5.24	Motility and Attachment		
		PA4528	*pilD*	type 4 prepilin peptidase PilD	-2.07	Secreted Factors (toxins, enzymes, alginate)	Protein secretion/export apparatus	Motility and Attachment
		PA4546	*pilS*	two-component sensor PilS	-3.44	Two-component regulatory systems		
		PA4547	*pilR*	two-component response regulator PilR	-10.31	Two-component regulatory systems	Transcriptional regulators	
		PA4549	*fimT*	type 4 fimbrial biogenesis protein FimT	5.43	Motility and Attachment		
		PA4550	*fimU*	type 4 fimbrial biogenesis protein FimU	-4.29	Motility and Attachment		
		PA4551	*pilV*	type 4 fimbrial biogenesis protein PilV	-4.63	Motility and Attachment		
		PA4552	*pilW*	type 4 fimbrial biogenesis protein PilW	-9.01	Motility and Attachment		
		PA4553	*pilX*	type 4 fimbrial biogenesis protein PilX	-17.86	Motility and Attachment		
		PA4554	*pilY1*	type 4 fimbrial biogenesis protein PilY1	-3.57	Motility and Attachment		
		PA4555	*pilY2*	type 4 fimbrial biogenesis protein PilY2	-6.33	Motility and Attachment		
		PA4556	*pilE*	type 4 fimbrial biogenesis protein PilE	-11.36	Motility and Attachment		
		PA4958	*fimW*	FimW	-7.75	Hypothetical, unclassified, unknown		
		PA5040	*pilQ*	Type 4 fimbrial biogenesis outer membrane protein PilQ precursor	-4.93	Motility and Attachment		

Motility and attachment		PA5041	*pilP*	type 4 fimbrial biogenesis protein PilP	-2.49	Motility and Attachment		
	Type IVb pilus biogenesis (Flp pilus)	PA4293	*pprA*	two-component sensor PprA	-5.03	Two-component regulatory systems		
		PA4294		hypothetical protein	-8.33	Hypothetical, unclassified, unknown		
		PA4295	*fppA*	Flp prepilin peptidase A, FppA	-2.13	Motility and Attachment		
		PA4296	*pprB*	two-component response regulator, PprB	-7.81	Two-component regulatory systems	Transcriptional regulators	
		PA4297	*tadG*	TadG	-3.13	Motility and Attachment	Membrane proteins	
		PA4299	*tadD*	TadD	-18.52	Motility and Attachment		
		PA4300	*tadC*	TadC	-6.41	Motility and Attachment	Membrane proteins	
		PA4301	*tadB*	TadB	-5.03	Motility and Attachment	Membrane proteins	
		PA4302	*tadA*	TadA ATPase	-5.00	Motility and Attachment	Protein secretion/export apparatus	
		PA4303	*tadZ*	TadZ	-3.58	Motility and Attachment		
		PA4304	*rcpA*	RcpA	-2.17	Motility and Attachment	Protein secretion/export apparatus	
		PA4305	*rcpC*	RcpC	-2.20	Motility and Attachment		
		PA4306	*flp*	Type IVb pilin, Flp	-62.50	Motility and Attachment		
	Cup fimbriae	PA0499		probable pili assembly chaperone	3.60	Motility and Attachment	Chaperones & heat shock proteins	
		PA0993	*cupC2*	chaperone CupC2	7.80	Motility and Attachment	Chaperones & heat shock proteins	
		PA0994	*cupC3*	usher CupC3	2.99	Motility and Attachment		
		PA2127	*cgrA*	cupA gene regulator A, CgrA	2.94	Transcriptional regulators		
		PA2129	*cupA2*	chaperone CupA2	3.15	Motility and Attachment	Chaperones & heat shock proteins	
		PA2130	*cupA3*	usher CupA3	4.37	Motility and Attachment		
		PA2131	*cupA4*	fimbrial subunit CupA4	3.57	Motility and Attachment		
		PA2132	*cupA5*	chaperone CupA5	3.56	Motility and Attachment	Chaperones & heat shock proteins	
		PA3044	*rocsS2*	Two-component sensor RocS2	8.51	Two-component regulatory systems		
		PA3045	*rocA2*	Two-component response regulator, RocA2	10.23	Two-component regulatory systems	Transcriptional regulators	
		PA3946	*rocS1*	two-component sensor RocS1	4.20	Two-component regulatory systems		
		PA3948	*rocA1*	Two-component response regulator RocA1	-2.74	Two-component regulatory systems	Transcriptional regulators	
		PA4081	*cupB6*	fimbrial subunit CupB6	2.81	Motility and Attachment		
		PA4083	*cupB4*	chaperone CupB4	9.00	Motility and Attachment	Chaperones & heat shock proteins	
		PA4084	*cupB3*	usher CupB3	5.86	Motility and Attachment		
		PA4085	*cupB2*	chaperone CupB2	3.47	Motility and Attachment	Chaperones & heat shock proteins	
		PA4648	*cupE1*	Pilin subunit CupE1	-28.57	Motility and Attachment	Cell Wall / LPS / Capsule	
		PA4649	*cupE2*	Pilin subunit CupE2	-25.00	Motility and Attachment	Cell Wall / LPS / Capsule	
		PA4650	*cupE3*	Pilin subunit CupE3	-17.24	Motility and Attachment	Cell Wall / LPS / Capsule	
		PA4651	*cupE4*	Pilin assembly chaperone CupE4	-52.63	Motility and Attachment	Cell Wall / LPS / Capsule	Chaperones & heat shock proteins
		PA4652	*cupE5*	Fimbrial usher protein CupE5	-9.17	Motility and Attachment	Cell Wall / LPS / Capsule	
		PA4653	*cupE6*	Adhesin-like protein CupE6	-28.57	Motility and Attachment	Cell Wall / LPS / Capsule	
	Flagellum	PA1077	*flgB*	flagellar basal-body rod protein FlgB	-2.54	Cell Wall / LPS / Capsule	Motility and Attachment	
		PA1078	*flgC*	flagellar basal-body rod protein FlgC	-2.61	Cell Wall / LPS / Capsule	Motility and Attachment	
		PA1079	*flgD*	flagellar basal-body rod modification protein FlgD	-3.62	Cell Wall / LPS / Capsule	Motility and Attachment	
		PA1080	*flgE*	flagellar hook protein FlgE	-9.01	Cell Wall / LPS / Capsule	Motility and Attachment	
		PA1085	*flgJ*	flagellar protein FlgJ	-2.88	Cell Wall / LPS / Capsule	Motility and Attachment	
		PA1086	*flgK*	flagellar hook-associated protein 1 FlgK	-10.99	Cell Wall / LPS / Capsule	Motility and Attachment	
		PA1087	*flgL*	flagellar hook-associated protein type 3 FlgL	-2.22	Cell Wall / LPS / Capsule	Motility and Attachment	
		PA1091	*fgtA*	flagellar glycosyl transferase, FgtA	-5.68	Hypothetical, unclassified, unknown		
		PA1095	*fliS*	hypothetical protein	2.94	Hypothetical, unclassified, unknown		
		PA1096	*fliT*	hypothetical protein	4.12	Hypothetical, unclassified, unknown		
		PA1098	*fleS*	two-component sensor	8.17	Two-component regulatory systems		
		PA1099	*fleR*	two-component response regulator	2.58	Two-component regulatory systems	Transcriptional regulators	Motility and Attachment
		PA1100	*fliE*	flagellar hook-basal body complex protein FliE	8.46	Cell Wall / LPS / Capsule	Motility and Attachment	
		PA1101	*fliF*	Flagella M-ring outer membrane protein precursor	7.93	Cell Wall / LPS / Capsule	Motility and Attachment	
		PA1102	*fliG*	flagellar motor switch protein FliG	3.05	Cell Wall / LPS / Capsule	Motility and Attachment	
		PA1103	*fliH*	probable flagellar assembly protein	2.75	Cell Wall / LPS / Capsule	Motility and Attachment	
		PA1104	*fliI*	flagellum-specific ATP synthase FliI	2.38	Energy metabolism	Motility and Attachment	
		PA1105	*fliJ*	flagellar protein FliJ	3.78	Cell Wall / LPS / Capsule	Motility and Attachment	
		PA1442	*fliL*	conserved hypothetical protein	4.65	Membrane proteins		
		PA1443	*fliM*	flagellar motor switch protein FliM	3.06	Adaptation, Protection	Motility and Attachment	Chemotaxis
		PA1444	*fliN*	flagellar motor switch protein FliN	2.33	Adaptation, Protection	Motility and Attachment	Chemotaxis
		PA1445	*fliO*	flagellar protein FliO	2.45	Adaptation, Protection	Motility and Attachment	Chemotaxis
		PA1446	*fliP*	flagellar biosynthetic protein FliP	3.48	Adaptation, Protection	Motility and Attachment	Chemotaxis
		PA1448	*fliR*	flagellar biosynthetic protein FliR	3.73	Adaptation, Protection	Motility and Attachment	Chemotaxis
		PA1449	*flhB*	flagellar biosynthetic protein FlhB	3.64	Adaptation, Protection	Motility and Attachment	Chemotaxis
		PA1450		conserved hypothetical protein	3.01	Hypothetical, unclassified, unknown		
		PA1452	*flhA*	flagellar biosynthesis protein FlhA	17.68	Adaptation, Protection	Motility and Attachment	Chemotaxis
		PA1453	*flhF*	flagellar biosynthesis protein FlhF	6.43	Adaptation, Protection	Motility and Attachment	Chemotaxis
		PA1454	*fleN*	flagellar synthesis regulator FleN	3.69	Motility and Attachment		
		PA1455	*fliA*	sigma factor FliA	3.13	Transcriptional regulators		
		PA1456	*cheY*	two-component response regulator CheY	2.34	Two-component regulatory systems	Transcriptional regulators	
		PA1458	*cheA*	probable two-component sensor	3.49	Two-component regulatory systems	Chemotaxis	
		PA1460	*motC*	MotC	2.15	Motility and Attachment		
		PA1461	*motD*	MotD	2.05	Motility and Attachment		
		PA1464	*cheW*	probable purine-binding chemotaxis protein	2.06	Adaptation, Protection	Chemotaxis	
		PA3349		probable chemotaxis protein	2.52	Adaptation, Protection	Chemotaxis	
		PA3350		hypothetical protein	4.31	Hypothetical, unclassified, unknown		
		PA3351	*flgM*	FlgM	8.12	Transcriptional regulators		
		PA3352		hypothetical protein	8.77	Hypothetical, unclassified, unknown		
		PA3353	*flgZ*	FlgZ	10.43	Hypothetical, unclassified, unknown		
		PA3526	*motY*	MotY	12.42	Membrane proteins		
		PA4953	*motB*	chemotaxis protein MotB	4.07	Adaptation, Protection	Chemotaxis	Membrane proteins
		PA4954	*motA*	chemotaxis protein MotA	3.88	Adaptation, Protection	Chemotaxis	
	Fap amyloids	PA1951	*fapF*	FapF	-16.13	Motility and Attachment		
		PA1952	*fapE*	FapE	3.94	Motility and Attachment		
		PA1953	*fapD*	FapD	3.34	Motility and Attachment		
		PA1955	*fapB*	FapB	13.57	Motility and Attachment		
		PA1956	*fapA*	FapA	21.97	Motility and Attachment		
Phage and pyocins	F2-R2 pyocins regulation	PA0610	*prtN*	transcriptional regulator PrtN	9.05	Transcriptional regulators		
		PA0611	*prtR*	transcriptional regulator PrtR	2.05	Transcriptional regulators		
	R2 pyocin	PA0614		hypothetical protein	-4.42	Hypothetical, unclassified, unknown		
		PA0615		hypothetical protein	-4.13	Hypothetical, unclassified, unknown		
		PA0616		hypothetical protein	-3.11	Relative to phage, transposon, or plasmid		
		PA0617		probable bacteriophage protein	-6.06	Relative to phage, transposon, or plasmid		
		PA0618		probable bacteriophage protein	-5.41	Relative to phage, transposon, or plasmid		
		PA0619		probable bacteriophage protein	-4.67	Relative to phage, transposon, or plasmid		
		PA0620		probable bacteriophage protein	-4.55	Relative to phage, transposon, or plasmid		
		PA0621		conserved hypothetical protein	-4.08	Relative to phage, transposon, or plasmid		
		PA0622		probable bacteriophage protein	-7.25	Relative to phage, transposon, or plasmid		
		PA0623		probable bacteriophage protein	-7.09	Relative to phage, transposon, or plasmid		
		PA0624		hypothetical protein	-6.90	Relative to phage, transposon, or plasmid		
		PA0625		hypothetical protein	-3.30	Relative to phage, transposon, or plasmid		
		PA0626		hypothetical protein	-2.39	Relative to phage, transposon, or plasmid		
		PA0627		conserved hypothetical protein	-2.54	Relative to phage, transposon, or plasmid		
		PA0628		conserved hypothetical protein	-2.56	Relative to phage, transposon, or plasmid		
	F2 pyocin	PA0629		conserved hypothetical protein	-3.98	Relative to phage, transposon, or plasmid		
		PA0630		hypothetical protein	-3.91	Relative to phage, transposon, or plasmid		
		PA0631		hypothetical protein	-3.62	Relative to phage, transposon, or plasmid		
		PA0632		hypothetical protein	-4.93	Relative to phage, transposon, or plasmid		
		PA0633		hypothetical protein	-7.87	Relative to phage, transposon, or plasmid		
		PA0634		hypothetical protein	-5.81	Relative to phage, transposon, or plasmid		
		PA0635		hypothetical protein	-5.68	Relative to phage, transposon, or plasmid		
		PA0636		hypothetical protein	-4.52	Relative to phage, transposon, or plasmid		
		PA0637		conserved hypothetical protein	-5.08	Relative to phage, transposon, or plasmid		
		PA0638		probable bacteriophage protein	-5.38	Relative to phage, transposon, or plasmid		
		PA0639		conserved hypothetical protein	-5.43	Relative to phage, transposon, or plasmid		
		PA0640		probable bacteriophage protein	-4.85	Relative to phage, transposon, or plasmid		
		PA0641		probable bacteriophage protein	-3.86	Relative to phage, transposon, or plasmid		
		PA0642		hypothetical protein	-3.06	Relative to phage, transposon, or plasmid		
		PA0643		hypothetical protein	-2.87	Relative to phage, transposon, or plasmid		
		PA0644		hypothetical protein	-3.86	Relative to phage, transposon, or plasmid		
		PA0645		hypothetical protein	-3.25	Relative to phage, transposon, or plasmid		
		PA0646		hypothetical protein	-5.10	Relative to phage, transposon, or plasmid		
		PA0647		hypothetical protein	-5.78	Relative to phage, transposon, or plasmid		
		PA0648		hypothetical protein	-5.88	Relative to phage, transposon, or plasmid		
	S4 pyocin	PA3866		Pyocin S4	-3.21	Adaptation, Protection	Secreted Factors (toxins, enzymes, alginate)	
	S5 pyocin	PA0984		colicin immunity protein	-2.58	Membrane proteins	Secreted Factors (toxins, enzymes, alginate)	
		PA0985	*pyoS5*	pyocin S5	-8.13	Membrane proteins	Secreted Factors (toxins, enzymes, alginate)	
	Pf4 phage	PA0715		hypothetical protein	-2.67	Relative to phage, transposon, or plasmid		
		PA0716		hypothetical protein	-3.70	Relative to phage, transposon, or plasmid		
		PA0717		hypothetical protein of bacteriophage Pf1	8,180.44	Relative to phage, transposon, or plasmid		
		PA0718		hypothetical protein of bacteriophage Pf1	11,362.12	Relative to phage, transposon, or plasmid		
		PA0719		hypothetical protein of bacteriophage Pf1	7,965.00	Relative to phage, transposon, or plasmid		
		PA0720		helix destabilizing protein of bacteriophage Pf1	6,316.21	Relative to phage, transposon, or plasmid	DNA replication, recombination, modification and repair	
		PA0721		hypothetical protein of bacteriophage Pf1	4,090.89	Relative to phage, transposon, or plasmid		
		PA0722		hypothetical protein of bacteriophage Pf1	5,689.38	Relative to phage, transposon, or plasmid		
		PA0723	*coaB*	coat protein B of bacteriophage Pf1	2,078.30	Relative to phage, transposon, or plasmid		
		PA0724		probable coat protein A of bacteriophage Pf1	3,178.13	Relative to phage, transposon, or plasmid		
		PA0725		hypothetical protein of bacteriophage Pf1	1,711.45	Relative to phage, transposon, or plasmid		
		PA0726		hypothetical protein of bacteriophage Pf1	304.06	Relative to phage, transposon, or plasmid		
		PA0727		Pf replication initiator protein	56.10	Relative to phage, transposon, or plasmid		
		PA0728		probable bacteriophage integrase	4.81	Relative to phage, transposon, or plasmid	Putative enzymes	
		PA0729		hypothetical protein	10.69	Hypothetical, unclassified, unknown		
	Integrases	PA0257		hypothetical protein	3.35	Relative to phage, transposon, or plasmid		
		PA0978		conserved hypothetical protein	4.22	Relative to phage, transposon, or plasmid		
		PA0987		conserved hypothetical protein	12.12	Relative to phage, transposon, or plasmid		
		PA1938		conserved hypothetical protein	4.33	Relative to phage, transposon, or plasmid		
		PA3142		integrase	2.34	Relative to phage, transposon, or plasmid		
	Transposases	PA0979		conserved hypothetical protein	2.67	Relative to phage, transposon, or plasmid		
		PA0986		conserved hypothetical protein	8.79	Relative to phage, transposon, or plasmid		
		PA1937		conserved hypothetical protein	3.71	Relative to phage, transposon, or plasmid		
		PA2319		probable transposase	2.23	Relative to phage, transposon, or plasmid		
		PA2690		probable transposase	2.19	Relative to phage, transposon, or plasmid		
		PA3434		probable transposase	2.06	Relative to phage, transposon, or plasmid		
		PA3993		probable transposase	2.10	Relative to phage, transposon, or plasmid		
		PA4797		probable transposase	2.05	Relative to phage, transposon, or plasmid		
	Unknown function	PA0613		hypothetical protein	4.44	Hypothetical, unclassified, unknown		
		PA2633		hypothetical protein	-4.63	Relative to phage, transposon, or plasmid		
Cellular division and shape	Cellular division	PA0857	*bolA*	morphogene protein BolA	-2.28	Cell division		
		PA2615	*ftsK*	cell division protein FtsK	2.82	Antibiotic resistance and susceptibility	Cell division	
		PA4407	*ftsZ*	cell division protein FtsZ	-4.29	Cell division		
		PA4409	*ftsQ*	cell division protein FtsQ	-2.90	Cell division		
		PA4413	*ftsW*	cell division protein FtsW	-4.74	Cell division		
		PA5563	*soj*	chromosome partitioning protein Soj	2.67	Cell division	Central intermediary metabolism	
		PA5565	*gidA*	glucose-inhibited division protein A	2.16	Cell division	Central intermediary metabolism	
	Plasmid partioning	PA1462		probable plasmid partitioning protein	3.22	Cell division		
	Cellular shape	PA4479	*mreD*	rod shape-determining protein MreD	7.16	Cell Wall / LPS / Capsule	Cell division	
		PA4480	*mreC*	rod shape-determining protein MreC	7.74	Cell Wall / LPS / Capsule	Cell division	
		PA4481	*mreB*	rod shape-determining protein MreB	3.41	Cell Wall / LPS / Capsule	Cell division	
Antibiotic resistance	Aminoglycoside resistance	PA0058	*dsbM*	DsbM	2.22	Antibiotic resistance and susceptibility		
		PA0463	*creB*	two-component response regulator CreB	8.46	Two-component regulatory systems	Transcriptional regulators	
		PA0464	*creC*	two-component sensor CreC	8.24	Two-component regulatory systems		
		PA0465	*creD*	inner membrane protein CreD	10.64	Membrane proteins		
		PA0779	*asrA*	AsrA	-2.81	Adaptation, Protection	Putative enzymes	
		PA1858	*str*	streptomycin 3''-phosphotransferase	-4.39	Antibiotic resistance and susceptibility		
		PA2018	*mexY*	Resistance-Nodulation-Cell Division (RND) multidrug efflux transporter MexY	8.33	Membrane proteins	Antibiotic resistance and susceptibility	Transport of small molecules
		PA2019	*mexX*	Resistance-Nodulation-Cell Division (RND) multidrug efflux membrane fusion protein MexX precursor	13.54	Antibiotic resistance and susceptibility	Transport of small molecules	
		PA2020	*mexZ*	MexZ	2.51	Transcriptional regulators		
		PA2818	*arr*	aminoglycoside response regulator	13.81	Antibiotic resistance and susceptibility	Nucleotide biosynthesis and metabolism	
		PA4119	*aph*	aminoglycoside 3'-phosphotransferase type IIb	3.30	Antibiotic resistance and susceptibility		
		PA5471	*armZ*	ArmZ	19.12	Antibiotic resistance and susceptibility		
	Chloramphenicol resistance	PA0706	*cat*	chloramphenicol acetyltransferase	-2.48	Antibiotic resistance and susceptibility		
		PA2047	*cmrA*	chloramphenicol resistance activator CmrA	-5.18	Transcriptional regulators		
	β-lactam resistance	PA0807	*ampDh3*	AmpDh3	-6.37	Antibiotic resistance and susceptibility		
		PA0958	*oprD*	Basic amino acid, basic peptide and imipenem outer membrane porin OprD precursor	-6.80	Transport of small molecules		
		PA4110	*ampC*	beta-lactamase precursor	-3.38	Adaptation, Protection		
		PA4218	*ampP*	AmpP	-4.93	Membrane proteins	Antibiotic resistance and susceptibility	Transport of small molecules
		PA4219	*ampO*	AmpO	-4.72	Membrane proteins	Antibiotic resistance and susceptibility	
		PA5288	*glnK*	nitrogen regulatory protein P-II 2	-4.33	Antibiotic resistance and susceptibility	Central intermediary metabolism	
		PA5514		probable beta-lactamase	4.60	Antibiotic resistance and susceptibility	Putative enzymes	
	Triclosan resistance	PA0158	*triC*	Resistance-Nodulation-Cell Division (RND) triclosan efflux transporter, TriC	-2.49	Antibiotic resistance and susceptibility	Transport of small molecules	
	Vanadium resistance	PA4206	*mexH*	probable Resistance-Nodulation-Cell Division (RND) efflux membrane fusion protein precursor	-2.17	Transport of small molecules		
		PA4207	*mexI*	probable Resistance-Nodulation-Cell Division (RND) efflux transporter	-4.37	Transport of small molecules	Membrane proteins	
		PA4208	*opmD*	probable outer membrane protein precursor	-22.73	Transport of small molecules	Membrane proteins	
	Bacitracine resistance	PA1959	*bacA*	bacitracin resistance protein	22.98	Cell Wall / LPS / Capsule	Antibiotic resistance and susceptibility	Adaptation, Protection
	Microbial peptides resistance	PA1798	*parS*	two-component sensor, ParS	-3.01	Two-component regulatory systems	Antibiotic resistance and susceptibility	
		PA1972		conserved hypothetical protein	19.89	Membrane proteins		
		PA2656	*bqsS*	two-component sensor BqsS	3.58	Two-component regulatory systems		
		PA2800	*vacJ*	VacJ	-2.65	Antibiotic resistance and susceptibility		
	Multiple resistance	PA0424	*mexR*	multidrug resistance operon repressor MexR	6.17	Transcriptional regulators		
		PA0426	*mexB*	Resistance-Nodulation-Cell Division (RND) multidrug efflux transporter MexB	-2.97	Membrane proteins	Antibiotic resistance and susceptibility	Transport of small molecules
		PA0427	*oprM*	Major intrinsic multiple antibiotic resistance efflux outer membrane protein OprM precursor	-3.45	Membrane proteins	Antibiotic resistance and susceptibility	Transport of small molecules
		PA1361	*norM*	NorM	3.61	Membrane proteins	Transport of small molecules	
		PA1874		hypothetical protein	-33.33	Antibiotic resistance and susceptibility		
		PA1875		probable outer membrane protein precursor	-15.38	Antibiotic resistance and susceptibility	Protein secretion/export apparatus	
		PA1876		probable ATP-binding/permease fusion ABC transporter	-10.87	Antibiotic resistance and susceptibility	Protein secretion/export apparatus	Secreted Factors (toxins, enzymes, alginate)
		PA1877		probable secretion protein	-12.82	Antibiotic resistance and susceptibility	Protein secretion/export apparatus	
		PA2491	*mexS*	MexS	-2.05	Transcriptional regulators	Putative enzymes	
		PA2492	*mexT*	transcriptional regulator MexT	-2.38	Transcriptional regulators		
		PA2493	*mexE*	Resistance-Nodulation-Cell Division (RND) multidrug efflux membrane fusion protein MexE precursor	7.51	Antibiotic resistance and susceptibility	Transport of small molecules	
		PA2494	*mexF*	Resistance-Nodulation-Cell Division (RND) multidrug efflux transporter MexF	9.97	Antibiotic resistance and susceptibility	Transport of small molecules	Membrane proteins
		PA2495	*oprN*	Multidrug efflux outer membrane protein OprN precursor	13.37	Antibiotic resistance and susceptibility	Transport of small molecules	Membrane proteins
		PA2523	*czcR*	CzcR	3.46	Two-component regulatory systems	Transcriptional regulators	
		PA2524	*czcS*	CzcS	4.61	Two-component regulatory systems		
		PA2527	*muxB*	MuxB	6.06	Antibiotic resistance and susceptibility	Transport of small molecules	Membrane proteins
		PA2528	*muxA*	MuxA	13.56	Antibiotic resistance and susceptibility	Transport of small molecules	
		PA3206	*cpxS*	CpxS	3.24	Two-component regulatory systems		
		PA3521	*opmE*	OpmE	3.50	Membrane proteins	Transport of small molecules	
		PA3522	*mexQ*	MexQ	5.53	Membrane proteins	Transport of small molecules	
		PA3523	*mexP*	MexP	14.31	Transport of small molecules		
		PA3719	*armR*	antirepressor for MexR, ArmR	6.20	Antibiotic resistance and susceptibility		
		PA4356	*xenB*	xenobiotic reductase	-4.65	Antibiotic resistance and susceptibility	Adaptation, Protection	
		PA4375	*mexW*	Resistance-Nodulation-Cell Division (RND) multidrug efflux transporter MexW	-2.01	Membrane proteins	Transport of small molecules	
		PA4596	*esrC*	EsrC	13.23	Transcriptional regulators		
		PA4597	*oprJ*	Multidrug efflux outer membrane protein OprJ precursor	2.73	Transport of small molecules	Antibiotic resistance and susceptibility	Membrane proteins
		PA4598	*mexD*	Resistance-Nodulation-Cell Division (RND) multidrug efflux transporter MexD	3.08	Transport of small molecules	Antibiotic resistance and susceptibility	Membrane proteins
		PA4599	*mexC*	Resistance-Nodulation-Cell Division (RND) multidrug efflux membrane fusion protein MexC precursor	10.00	Transport of small molecules	Antibiotic resistance and susceptibility	
		PA4600	*nfxB*	transcriptional regulator NfxB	-3.33	Transcriptional regulators		
	Others	PA1237		probable multidrug resistance efflux pump	12.29	Antibiotic resistance and susceptibility	Transport of small molecules	
		PA1238		probable outer membrane component of multidrug efflux pump	9.07	Antibiotic resistance and susceptibility	Transport of small molecules	
		PA3133	*sawR*	SawR	2.55	Transcriptional regulators		
Iron homeostasis	Pyoverdine biosynthesis	PA2385	*pvdQ*	3-oxo-C12-homoserine lactone acylase PvdQ	-8.47	Adaptation, Protection		
		PA2386	*pvdA*	L-ornithine N5-oxygenase	-13.16	Adaptation, Protection		
		PA2389	*pvdR*	PvdR	-2.37	Transport of small molecules		
		PA2390	*pvdT*	PvdT	-4.61	Transport of small molecules	Membrane proteins	
		PA2391	*opmQ*	probable outer membrane protein precursor	-8.70	Transport of small molecules	Membrane proteins	
		PA2392	*pvdP*	PvdP	-3.26	Adaptation, Protection		
		PA2393	*pvdM*	putative dipeptidase	-12.35	Central intermediary metabolism		
		PA2394	*pvdN*	PvdN	-14.49	Adaptation, Protection		
		PA2395	*pvdO*	PvdO	-8.47	Adaptation, Protection		
		PA2396	*pvdF*	pyoverdine synthetase F	-5.46	Adaptation, Protection	Secreted Factors (toxins, enzymes, alginate)	
		PA2397	*pvdE*	pyoverdine biosynthesis protein PvdE	-13.51	Adaptation, Protection	Membrane proteins	Transport of small molecules
		PA2398	*fpvA*	ferripyoverdine receptor	-8.13	Transport of small molecules		
		PA2402	*pvdI*	probable non-ribosomal peptide synthetase	-2.07	Putative enzymes		
		PA2403	*fpvG*	FpvG	-2.90	Membrane proteins		
		PA2404	*fpvH*	FpvH	-4.95	Membrane proteins		
		PA2405	*fpvJ*	FpvJ	-5.52	Hypothetical, unclassified, unknown		
		PA2406	*fpvK*	FpvK	-5.13	Hypothetical, unclassified, unknown		
		PA2407	*fpvC*	FpvC	-5.71	Transport of small molecules	Motility and Attachment	
		PA2408	*fpvD*	FpvD	-5.46	Transport of small molecules		
		PA2409	*fpvE*	FpvE	-5.99	Transport of small molecules	Membrane proteins	
		PA2410	*fpvF*	FpvF	-4.65	Transport of small molecules		
		PA2411		probable thioesterase	-5.10	Adaptation, Protection	Putative enzymes	
		PA2412	*mbtH*	conserved hypothetical protein	-5.26	Hypothetical, unclassified, unknown		
		PA2413	*pvdH*	L-2,4-diaminobutyrate:2-ketoglutarate 4-aminotransferase, PvdH	-3.85	Adaptation, Protection		
		PA2424	*pvdL*	PvdL	-6.54	Adaptation, Protection		
		PA2425	*pvdG*	PvdG	-6.67	Adaptation, Protection		
		PA2426	*pvdS*	sigma factor PvdS	-33.33	Transcriptional regulators		
		PA4168	*fpvB*	second ferric pyoverdine receptor FpvB	-4.07	Transport of small molecules		
	Pyochelin biosynthesis	PA4220	*fptB*	hypothetical protein	-6.99	Hypothetical, unclassified, unknown		
		PA4221	*fptA*	Fe(III)-pyochelin outer membrane receptor precursor	-6.49	Transport of small molecules		
		PA4223	*pchH*	probable ATP-binding component of ABC transporter	-2.43	Transport of small molecules	Membrane proteins	
		PA4224	*pchG*	pyochelin biosynthetic protein PchG	-3.28	Transport of small molecules	Membrane proteins	
		PA4225	*pchF*	pyochelin synthetase	-4.07	Transport of small molecules	Secreted Factors (toxins, enzymes, alginate)	
		PA4226	*pchE*	dihydroaeruginoic acid synthetase	-5.71	Transport of small molecules	Secreted Factors (toxins, enzymes, alginate)	
		PA4227	*pchR*	transcriptional regulator PchR	-4.18	Transcriptional regulators		
		PA4228	*pchD*	pyochelin biosynthesis protein PchD	-10.20	Transport of small molecules	Secreted Factors (toxins, enzymes, alginate)	
		PA4229	*pchC*	pyochelin biosynthetic protein PchC	-11.76	Transport of small molecules	Secreted Factors (toxins, enzymes, alginate)	
		PA4230	*pchB*	salicylate biosynthesis protein PchB	-12.50	Transport of small molecules	Secreted Factors (toxins, enzymes, alginate)	
		PA4231	*pchA*	salicylate biosynthesis isochorismate synthase	-10.53	Transport of small molecules	Secreted Factors (toxins, enzymes, alginate)	
	Enterobactin uptake	PA2686	*pfeR*	two-component response regulator PfeR	-3.30	Two-component regulatory systems	Transcriptional regulators	
		PA0931	*pirA*	ferric enterobactin receptor PirA	-2.92	Transport of small molecules		
		PA4158	*fepC*	ferric enterobactin transport protein FepC	2.81	Transport of small molecules		
		PA4159	*fepB*	ferrienterobactin-binding periplasmic protein precursor FepB	5.95	Transport of small molecules		
		PA4160	*fepD*	ferric enterobactin transport protein FepD	3.60	Transport of small molecules	Membrane proteins	
		PA4161	*fepG*	ferric enterobactin transport protein FepG	2.19	Transport of small molecules	Membrane proteins	
	Vibriobactin uptake	PA4156	*fvbA*	FvbA	3.89	Transport of small molecules		
	Heme uptake	PA3404		probable outer membrane protein precursor	2.49	Protein secretion/export apparatus		
		PA3407	*hasAp*	heme acquisition protein HasAp	-5.32	Transport of small molecules		
		PA4708	*phuT*	Heme-transport protein, PhuT	-2.11	Transport of small molecules		
		PA4710	*phuR*	Heme/Hemoglobin uptake outer membrane receptor PhuR precursor	-10.53	Transport of small molecules		
	Ferrioxamine uptake	PA2466	*foxA*	Ferrioxamine receptor FoxA	-3.07	Transport of small molecules		
		PA2467	*foxR*	Anti-sigma factor FoxR	-3.80	Membrane proteins	Transcriptional regulators	
		PA2468	*foxI*	ECF sigma factor FoxI	-4.35	Transcriptional regulators		
	Ferrichrome uptake	PA0470	*fiuA*	Ferrichrome receptor FiuA	-3.60	Membrane proteins	Transport of small molecules	
		PA0471	*fiuR*	FiuR	-9.35	Transcriptional regulators		
		PA0472	*fiuI*	FiuI	-8.70	Transcriptional regulators		
	Citrate-mediated iron uptake	PA3899	*fecI*	FecI	-2.25	Transcriptional regulators		
		PA3900	*fecR*	FecR	-3.85	Transcriptional regulators	Membrane proteins	
		PA3901	*fecA*	Fe(III) dicitrate transport protein FecA	-2.60	Transport of small molecules	Membrane proteins	
	Aerobactin uptake	PA4675	*chtA*	ChtA	-5.43	Transport of small molecules		

	Fe^{2+} uptake	PA4358	*feoB*	FeoB	-8.06	Membrane proteins	Transport of small molecules	
	Iron protection	PA1008	*bcp*	bacterioferritin comigratory protein	-9.52	Adaptation, Protection		
		PA3530	*bfd*	bacterioferritin-associated ferredoxin Bfd	-8.33	Hypothetical, unclassified, unknown		
		PA3531	*bfrB*	bacterioferritin	10.19	Adaptation, Protection	Transport of small molecules	
		PA4235	*ftnA*	bacterial ferritin	-6.94	Adaptation, Protection	Transport of small molecules	
	Energizing system	PA0197	*tonB2*	TonB2	9.00	Transport of small molecules		
		PA0198	*exbB1*	transport protein ExbB	3.40	Transport of small molecules		
		PA0693	*exbB2*	transport protein ExbB2	3.01	Membrane proteins	Transport of small molecules	
		PA0694	*exbD2*	transport protein ExbD	4.65	Membrane proteins	Transport of small molecules	
	Iron uptake regulation	PA4704.2	*prrF2*	regulatory RNA PrrF2	-7.75	Non-coding RNA gene		
		PA0150		anti-sigma factor	3.51	Membrane proteins	Transcriptional regulators	
		PA0151		probable TonB-dependent receptor	4.12	Transport of small molecules		
		PA1300		ECF sigma factor	4.41	Transcriptional regulators		
		PA1301		probable transmembrane sensor	4.55	Membrane proteins	Transcriptional regulators	
		PA1302		probable heme utilization protein precursor	5.94	Membrane proteins	Transport of small molecules	
		PA1363		ECF sigma factor	-3.25	Transcriptional regulators		
		PA1365		probable siderophore receptor	-2.86	Transport of small molecules		
		PA2050		probable sigma-70 factor, ECF subfamily	9.11	Transcriptional regulators		
		PA2051		probable transmembrane sensor	6.82	Membrane proteins	Transcriptional regulators	
		PA2070		hypothetical protein	8.58	Membrane proteins		
		PA2089		hypothetical protein	3.55	Hypothetical, unclassified, unknown		
		PA2094		probable transmembrane sensor	4.61	Membrane proteins	Transcriptional regulators	
	Others	PA0434		hypothetical protein	-2.24	Membrane proteins		
		PA4514		probable outer membrane receptor for iron transport	-3.17	Transport of small molecules		
	Gallium uptake	PA4687	*hitA*	ferric iron-binding periplasmic protein HitA	-8.00	Transport of small molecules		
		PA4688	*hitB*	iron (III)-transport system permease HitB	-5.56	Transport of small molecules	Membrane proteins	
		PA5217		probable binding protein component of ABC iron transporter	-9.71	Transport of small molecules		
**Oxidative phosphorylation**	NADH dehydrogenase (NDH-1)	PA1883		probable NADH-ubiquinone/plastoquinone oxidoreductase	32.13	Energy metabolism		
		PA2638	*nuoB*	NADH dehydrogenase I chain B	-3.73	Energy metabolism		
		PA2639	*nuoD*	NADH dehydrogenase I chain C,D	-7.69	Energy metabolism		
		PA2640	*nuoE*	NADH dehydrogenase I chain E	-10.53	Energy metabolism		
		PA2641	*nuoF*	NADH dehydrogenase I chain F	-9.62	Energy metabolism		
		PA2642	*nuoG*	NADH dehydrogenase I chain G	-8.70	Energy metabolism		
		PA2643	*nuoH*	NADH dehydrogenase I chain H	-8.20	Energy metabolism		
		PA2644	*nuoI*	NADH Dehydrogenase I chain I	-10.31	Energy metabolism		
		PA2645	*nuoJ*	NADH dehydrogenase I chain J	-9.90	Energy metabolism		
		PA2646	*nuoK*	NADH dehydrogenase I chain K	-9.80	Energy metabolism		
		PA2647	*nuoL*	NADH dehydrogenase I chain L	-12.50	Energy metabolism		
		PA2648	*nuoM*	NADH dehydrogenase I chain M	-10.42	Energy metabolism		
		PA2649	*nuoN*	NADH dehydrogenase I chain N	-9.17	Energy metabolism	Antibiotic resistance and susceptibility	
	NADH dehydrogenase (NDH-2)	PA4538	*ndh*	NADH dehydrogenase	-3.30	Energy metabolism		
	NQR biosynthesis (NDH-3)	PA2994	*nqrF*	Na+-translocating NADH:quinone oxidoreductase, subunit Nqr6	-3.31	Energy metabolism		
		PA2995	*nqrE*	Na+-translocating NADH:quinone oxidoreductase subunit Nqr5	-3.56	Energy metabolism		
		PA2996	*nqrD*	Na+-translocating NADH:uniquinone oxidoreductase subunit Nqr4	-3.16	Energy metabolism		
		PA2997	*nqrC*	Na+-translocating NADH:ubiquinone oxidoreductase subunit Nrq3	-2.17	Energy metabolism		
	Succinate dehydrogenase	PA1581	*sdhC*	succinate dehydrogenase (C subunit)	-2.43	Energy metabolism		
		PA1582	*sdhD*	succinate dehydrogenase (D subunit)	-4.52	Energy metabolism		
		PA1583	*sdhA*	succinate dehydrogenase (A subunit)	-6.21	Energy metabolism		
		PA1584	*sdhB*	succinate dehydrogenase (B subunit)	-6.25	Energy metabolism		
	Ubiquinone biosynthesis O2-dependent	PA0655	*ubiF*	hypothetical protein	-2.23	Hypothetical, unclassified, unknown		
		PA3171	*ubiG*	3-demethylubiquinone-9 3-methyltransferase	-2.35	Biosynthesis of cofactors, prosthetic groups and carrier	Energy metabolism	
		PA5063	*ubiE*	ubiquinone biosynthesis methyltransferase UbiE	-2.09	Energy metabolism	Biosynthesis of cofactors, prosthetic groups and carrier	
		PA5065	*ubiB*	ubiquinone biosynthetic protein UbiB	-4.83	Biosynthesis of cofactors, prosthetic groups and carrier	Putative enzymes	
		PA5221	*ubiI*	probable FAD-dependent monooxygenase	-2.84	Putative enzymes		
		PA5223	*ubiH*	ubiH protein	-4.39	Energy metabolism	Biosynthesis of cofactors, prosthetic groups and carrier	
	Ubiquinone biosynthesis O2-independent	PA3911	*ubiT*	conserved hypothetical protein	-4.42	Hypothetical, unclassified, unknown		
		PA3912	*ubiV*	conserved hypothetical protein	-4.26	Hypothetical, unclassified, unknown		
		PA3913	*UbiU*	probable protease	-3.05	Putative enzymes		
	Cytochrome c-type biogenesis	PA1480	*ccmF*	cytochrome C-type biogenesis protein CcmF	-3.79	Energy metabolism		
		PA1481	*ccmG*	cytochrome C biogenesis protein CcmG	-8.13	Translation, post-translational modification, degradation	Energy metabolism	Chaperones & heat shock proteins
		PA1482	*ccmH*	cytochrome C-type biogenesis protein CcmH	-8.85	Energy metabolism		
		PA1483	*cycH*	cytochrome c-type biogenesis protein	-6.10	Energy metabolism		
	Cytochrome bc1 complex	PA4429		probable cytochrome c1 precursor	-4.76	Energy metabolism		
		PA4430		probable cytochrome b	-3.83	Energy metabolism		
		PA4431		probable iron-sulfur protein	-2.53	Putative enzymes		
	Cytochrome aa3 oxidase	PA0105	*coxB*	cytochrome c oxidase, subunit II	2.20	Energy metabolism		
		PA0107		conserved hypothetical protein	-3.37	Energy metabolism		
		PA0108	*colII*	cytochrome c oxidase, subunit III	-4.07	Energy metabolism		
		PA0113		probable cytochrome c oxidase assembly factor	-7.09	Energy metabolism		
	Cytochrome bo3 quinol oxidase	PA1317	*cyoA*	cytochrome o ubiquinol oxidase subunit II	-3.70	Energy metabolism		
		PA1318	*cyoB*	cytochrome o ubiquinol oxidase subunit I	-9.17	Energy metabolism		
		PA1319	*cyoC*	cytochrome o ubiquinol oxidase subunit III	-7.30	Energy metabolism		
		PA1320	*cyoD*	cytochrome o ubiquinol oxidase subunit IV	-5.56	Energy metabolism		
		PA1321	*cyoE*	cytochrome o ubiquinol oxidase protein CyoE	-4.78	Energy metabolism		
	Cytochrome c oxidase ccb3-1	PA1552	*ccoP1*	Cytochrome c oxidase, cbb3-type, CcoP subunit	-5.21	Energy metabolism	Central intermediary metabolism	
		PA1552.1	*ccoQ1*	Cytochrome c oxidase, cbb3-type, CcoQ subunit	-5.85	Central intermediary metabolism		
		PA1553	*ccoO1*	Cytochrome c oxidase, cbb3-type, CcoO subunit	-6.02	Energy metabolism	Central intermediary metabolism	
		PA1554	*ccoN1*	Cytochrome c oxidase, cbb3-type, CcoN subunit	-5.62	Energy metabolism	Central intermediary metabolism	
	Cytochrome c oxidase ccb3-2	PA1555	*ccoP2*	Cytochrome c oxidase, cbb3-type, CcoP subunit	-12.20	Energy metabolism	Central intermediary metabolism	
		PA1555.1	*ccoQ2*	Cytochrome c oxidase, cbb3-type, CcoQ subunit	-9.17	Central intermediary metabolism		
		PA1556	*ccoO2*	Cytochrome c oxidase, cbb3-type, CcoO subunit	-11.24	Energy metabolism	Central intermediary metabolism	
		PA1557	*ccoN2*	Cytochrome c oxidase, cbb3-type, CcoN subunit	-7.87	Energy metabolism	Central intermediary metabolism	
	Cytochrome bd quinol oxidase	PA3929	*cioB*	cyanide insensitive terminal oxidase	-2.20	Energy metabolism		
		PA3930	*cioA*	cyanide insensitive terminal oxidase	-2.43	Energy metabolism		
	Cytochrome c oxidase ccb3-type	PA4133		cytochrome c oxidase subunit (cbb3-type)	-6.06	Adaptation, Protection	Energy metabolism	
		PA2482		probable cytochrome c	4.07	Energy metabolism		
		PA4571		probable cytochrome c	-2.65	Energy metabolism		
	Cytochrome b561	PA0918		cytochrome b561	-4.85	Energy metabolism		
	Cytochrome 551	PA4587	*ccpR*	cytochrome c551 peroxidase precursor	-2.90	Energy metabolism		
	Cytochrome P450	PA2475		probable cytochrome P450	-2.53	Adaptation, Protection	Putative enzymes	
		PA3331		cytochrome P450	-9.52	Adaptation, Protection	Carbon compound catabolism	
	Cytochrome c oxidase	PA0112		hypothetical protein	-4.85	Membrane proteins		
		PA1547		hypothetical protein	-2.33	Membrane proteins		
		PA1548		conserved hypothetical protein	-2.72	Hypothetical, unclassified, unknown		
		PA1550		hypothetical protein	-12.05	Hypothetical, unclassified, unknown		
		PA1551		probable ferredoxin	-5.85	Energy metabolism		
	F-type ATP synthase	PA5553	*atpC*	ATP synthase epsilon chain	-2.46	Energy metabolism		
		PA5554	*atpD*	ATP synthase beta chain	-2.89	Energy metabolism		
		PA5555	*atpG*	ATP synthase gamma chain	-2.88	Energy metabolism		
		PA5556	*atpA*	ATP synthase alpha chain	-2.46	Energy metabolism		
		PA5557	*atpH*	ATP synthase delta chain	-2.39	Energy metabolism		
		PA5558	*atpF*	ATP synthase B chain	-2.23	Energy metabolism		
		PA5559	*atpE*	atp synthase C chain	-2.12	Energy metabolism		
		PA5561	*atpI*	ATP synthase protein I	2.78	Energy metabolism	Membrane proteins	
	Flavoprotein	PA2951	*etfA*	electron transfer flavoprotein alpha-subunit	-2.15	Energy metabolism		
	Others	PA2714		probable molybdopterin oxidoreductase	2.48	Energy metabolism		
		PA3489		conserved hypothetical protein	3.29	Membrane proteins		
		PA4061		probable thioredoxin	-2.85	Energy metabolism		
**Carbohydrate metabolism**	Citrate cycle (TCA cycle)	PA1376	*aceK*	isocitrate dehydrogenase kinase/phosphatase	-4.57	Central intermediary metabolism		
		PA1562	*acnA*	aconitate hydratase 1	-3.12	Energy metabolism		
		PA1580	*gltA*	citrate synthase	-3.52	Energy metabolism		
		PA1585	*sucA*	2-oxoglutarate dehydrogenase (E1 subunit)	-7.19	Amino acid biosynthesis and metabolism	Energy metabolism	
		PA1586	*sucB*	dihydrolipoamide succinyltransferase (E2 subunit)	-5.85	Energy metabolism		
		PA1588	*sucC*	succinyl-CoA synthetase beta chain	-6.02	Energy metabolism		
		PA1589	*sucD*	succinyl-CoA synthetase alpha chain	-6.25	Energy metabolism		
		PA1787	*acnB*	aconitate hydratase 2	-2.77	Energy metabolism		
		PA2623	*icd*	isocitrate dehydrogenase	-5.03	Amino acid biosynthesis and metabolism	Energy metabolism	Carbon compound catabolism
		PA2624	*idh*	isocitrate dehydrogenase	-2.09	Energy metabolism		
		PA4333		probable fumarase	-2.26	Energy metabolism		
		PA4470	*fumC1*	fumarate hydratase	-23.26	Energy metabolism		
	Glycolysis/Gluconeogenesis	PA0927	*ldhA*	D-lactate dehydrogenase (fermentative)	-8.62	Energy metabolism	Carbon compound catabolism	Central intermediary metabolism
		PA1400		probable pyruvate carboxylase	5.15	Energy metabolism		
		PA1498	*pykF*	pyruvate kinase I	4.82	Energy metabolism	Carbon compound catabolism	
		PA1587	*lpd*	dihydrolipoamide dehydrogenase Lpd	-6.94	Amino acid biosynthesis and metabolism	Energy metabolism	
		PA2275		probable alcohol dehydrogenase (Zn-dependent)	8.43	Putative enzymes		
		PA2323	*gapN*	GapN	-3.25	Carbon compound catabolism		
		PA3001		probable glyceraldehyde-3-phosphate dehydrogenase	-2.51	Putative enzymes		
		PA3415		probable dihydrolipoamide acetyltransferase	-8.00	Energy metabolism		
		PA3416		probable pyruvate dehydrogenase E1 component, beta chain	-6.10	Energy metabolism		
		PA3417		probable pyruvate dehydrogenase E1 component, alpha subunit	-5.52	Energy metabolism		
		PA3629	*adhC*	alcohol dehydrogenase class III	-3.27	Central intermediary metabolism		
		PA3635	*eno*	enolase	-3.30	Translation, post-translational modification, degradation	Energy metabolism	Carbon compound catabolism
		PA3687	*ppc*	phosphoenolpyruvate carboxylase	-2.05	Energy metabolism	Central intermediary metabolism	
		PA4022	*hdhA*	hydrazone dehydrogenase, HdhA	-2.27	Putative enzymes		
		PA4329	*pykA*	pyruvate kinase II	-3.36	Energy metabolism	Carbon compound catabolism	
		PA4732	*pgi*	glucose-6-phosphate isomerase	-3.29	Energy metabolism	Carbon compound catabolism	
		PA4748	*tpiA*	triosephosphate isomerase	5.11	Energy metabolism	Central intermediary metabolism	
		PA5015	*aceE*	pyruvate dehydrogenase	-5.49	Energy metabolism	Amino acid biosynthesis and metabolism	
		PA5016	*aceF*	dihydrolipoamide acetyltransferase	-4.27	Energy metabolism	Carbon compound catabolism	
		PA5427	*adhA*	alcohol dehydrogenase	-15.38	Energy metabolism	Carbon compound catabolism	
	Pentose phosphate pathway	PA0548	*tktA*	transketolase	-3.80	Energy metabolism		
		PA2263	*kguD*	probable 2-hydroxyacid dehydrogenase	-2.52	Putative enzymes		
		PA2265	*gad*	gluconate dehydrogenase	-3.26	Carbon compound catabolism		
		PA2266		probable cytochrome c precursor	-5.32	Energy metabolism	Carbon compound catabolism	
		PA2290	*gcd*	glucose dehydrogenase	-5.43	Energy metabolism	Carbon compound catabolism	
		PA2321	*gntK*	GntK	8.63	Energy metabolism	Carbon compound catabolism	
		PA2796	*tal*	transaldolase	-2.97	Energy metabolism	Carbon compound catabolism	
		PA3181	*eda*	2-keto-3-deoxy-6-phosphogluconate aldolase	2.35	Carbon compound catabolism	Central intermediary metabolism	
		PA3182	*pgl*	6-phosphogluconolactonase	2.51	Central intermediary metabolism		
		PA3183	*zwf*	glucose-6-phosphate 1-dehydrogenase	4.08	Energy metabolism	Carbon compound catabolism	
		PA3896		probable 2-hydroxyacid dehydrogenase	-2.12	Putative enzymes		
	Ethanol oxydation	PA1978	*erbR*	response regulator ErbR	4.36	Transcriptional regulators		
		PA1979	*eraS*	sensor kinase, EraS	2.06	Two-component regulatory systems		
		PA1980	*eraR*	response regulator EraR	2.82	Two-component regulatory systems	Transcriptional regulators	
		PA1982	*exaA*	quinoprotein ethanol dehydrogenase	4.95	Carbon compound catabolism		
		PA1983	*exaB*	cytochrome c550	5.28	Energy metabolism	Carbon compound catabolism	
		PA1984	*exaC*	NAD+ dependent aldehyde dehydrogenase ExaC	-2.20	Putative enzymes		
	Cathecol degradation	PA2507	*catA*	catechol 1,2-dioxygenase	21.12	Carbon compound catabolism		
		PA2508	*catC*	muconolactone delta-isomerase	25.61	Carbon compound catabolism		
		PA2509	*catB*	muconate cycloisomerase I	42.83	Carbon compound catabolism		
		PA2510	*catR*	transcriptional regulator CatR	2.86	Transcriptional regulators	Carbon compound catabolism	
	Benzoate degradation	PA0247	*pobA*	p-hydroxybenzoate hydroxylase	5.81	Carbon compound catabolism		
		PA2515	*xylL*	cis-1,2-dihydroxycyclohexa-3,4-diene carboxylate dehydrogenase	2.45	Carbon compound catabolism		
		PA2516	*xylZ*	toluate 1,2-dioxygenase electron transfer component	3.55	Energy metabolism	Carbon compound catabolism	
		PA2517	*xylY*	toluate 1,2-dioxygenase beta subunit	9.20	Carbon compound catabolism		
		PA2518	*xylX*	toluate 1,2-dioxygenase alpha subunit	5.33	Carbon compound catabolism		
		PA2519	*xylS*	transcriptional regulator XylS	3.34	Transcriptional regulators		
	Protocatechuate degradation	PA0153	*pcaH*	protocatechuate 3,4-dioxygenase, beta subunit	2.58	Carbon compound catabolism		
		PA0154	*pcaG*	protocatechuate 3,4-dioxygenase, alpha subunit	2.72	Carbon compound catabolism		
		PA0155	*pcaR*	transcriptional regulator PcaR	2.12	Transcriptional regulators	Carbon compound catabolism	
		PA0228	*pcaF*	beta-ketoadipyl CoA thiolase PcaF	14.38	Fatty acid and phospholipid metabolism	Carbon compound catabolism	
		PA0229	*pcaT*	dicarboxylic acid transporter PcaT	3.82	Membrane proteins	Transport of small molecules	
		PA0235	*pcaK*	4-hydroxybenzoate transporter PcaK	15.07	Membrane proteins	Transport of small molecules	Carbon compound catabolism
	Carbon metabolism regulation	PA4726.11	*crcZ*	CrcZ	-2.92	Non-coding RNA gene	Transcriptional regulators	
		PA5332	*crc*	catabolite repression control protein	2.78	Energy metabolism	Carbon compound catabolism	
	Malonate degradation	PA0211	*mdcD*	malonate decarboxylase beta subunit	-2.13	Carbon compound catabolism		
		PA0212	*mdcE*	malonate decarboxylase gamma subunit	-3.60	Carbon compound catabolism		
		PA0213		hypothetical protein	-4.41	Carbon compound catabolism		
		PA0214		probable acyl transferase	-5.46	Carbon compound catabolism		
	Malonate transport	PA0215		malonate transporter MadL	-3.64	Membrane proteins	Transport of small molecules	
		PA0216		malonate transporter MadM	4.42	Membrane proteins	Transport of small molecules	
	Acetyl-CoA synthetase	PA0887	*acsA*	acetyl-coenzyme A synthetase	6.54	Carbon compound catabolism	Central intermediary metabolism	
	Urea metabolism	PA4893	*ureG*	urease accessory protein UreG	-4.02	Biosynthesis of cofactors, prosthetic groups and carrier		
		PA4864	*ureD*	urease accessory protein	-5.46	Central intermediary metabolism		
		PA4865	*ureA*	urease gamma subunit	-10.42	Central intermediary metabolism		
		PA4867	*ureB*	urease beta subunit	-13.89	Central intermediary metabolism		
		PA4868	*ureC*	urease alpha subunit	-16.67	Central intermediary metabolism		
	Urea transport	PA1497		probable transporter	2.94	Membrane proteins	Transport of small molecules	
	Butanoate metabolism	PA0226		probable CoA transferase, subunit A	25.63	Carbon compound catabolism		
		PA0227		probable CoA transferase, subunit B	25.37	Carbon compound catabolism		
		PA1146		probable iron-containing alcohol dehydrogenase	4.20	Carbon compound catabolism		
		PA1999	*dhcA*	dehydrocarnitine CoA transferase, subunit A	-6.29	Amino acid biosynthesis and metabolism	Carbon compound catabolism	
		PA2000	*dhcB*	dehydrocarnitine CoA transferase, subunit B	-5.62	Amino acid biosynthesis and metabolism	Carbon compound catabolism	
		PA2003	*bdhA*	3-hydroxybutyrate dehydrogenase	-10.31	Carbon compound catabolism		
		PA4148		probable short-chain dehydrogenase	8.53	Putative enzymes		
	Propanoate metabolism	PA0792	*prpD*	propionate catabolic protein PrpD	-11.76	Carbon compound catabolism		
		PA0794		probable aconitate hydratase	-3.68	Energy metabolism		
		PA0795	*prpC*	citrate synthase 2	-5.38	Carbon compound catabolism	Central intermediary metabolism	
		PA0796	*prpB*	carboxyphosphonoenolpyruvate phosphonomutase	-3.94	Fatty acid and phospholipid metabolism	Carbon compound catabolism	Central intermediary metabolism
		PA0835	*pta*	phosphate acetyltransferase	-6.29	Carbon compound catabolism		
	Galactose metabolism	PA1384	*galE*	UDP-glucose 4-epimerase	5.76	Carbon compound catabolism	Nucleotide biosynthesis and metabolism	Central intermediary metabolism
		PA4068		probable epimerase	-2.03	Central intermediary metabolism		
	Long-chain alcohol degradation	PA0364	*laoA*	LaoA	-2.90	Putative enzymes		
		PA0365	*laoB*	LaoB	-3.15	Membrane proteins		
		PA0366	*laoC*	LaoC	-2.10	Putative enzymes		
	Central metabolism	PA1726	*bglX*	periplasmic beta-glucosidase	-2.16	Carbon compound catabolism		
		PA1748		probable enoyl-CoA hydratase/isomerase	-2.15	Putative enzymes		
		PA1821		probable enoyl-CoA hydratase/isomerase	-2.18	Putative enzymes		
		PA2552		probable acyl-CoA dehydrogenase	-4.98	Putative enzymes		
		PA2553		probable acyl-CoA thiolase	-3.24	Putative enzymes		
		PA2940		probable acyl-CoA thiolase	4.21	Putative enzymes		
		PA3426		probable enoyl CoA-hydratase/isomerase	-4.85	Putative enzymes		
		PA3588		probable porin	3.78	Carbon compound catabolism	Membrane proteins	Transport of small molecules
		PA3589		probable acyl-CoA thiolase	5.41	Carbon compound catabolism		
		PA3590		probable hydroxyacyl-CoA dehydrogenase	3.26	Carbon compound catabolism		
		PA3591		probable enoyl-CoA hydratase/isomerase	6.37	Carbon compound catabolism		
		PA3592		conserved hypothetical protein	5.04	Hypothetical, unclassified, unknown		
		PA3593		probable acyl-CoA dehydrogenase	5.41	Putative enzymes		
		PA3925		probable acyl-CoA thiolase	2.27	Putative enzymes		
		PA4147	*acoR*	transcriptional regulator AcoR	2.56	Transcriptional regulators		
		PA4150		probable dehydrogenase E1 component	4.59	Carbon compound catabolism		
		PA4151	*acoB*	acetoin catabolism protein AcoB	7.44	Carbon compound catabolism		
		PA4152		probable hydrolase	3.84	Carbon compound catabolism		
		PA4785		probable acyl-CoA thiolase	5.40	Putative enzymes		
	Glyoxylate and dicarboxylate metabolism	PA1502	*gcl*	glyoxylate carboligase	4.25	Central intermediary metabolism		
		PA5353	*glcF*	glycolate oxidase subunit GlcF	5.56	Carbon compound catabolism	Central intermediary metabolism	
		PA5354	*glcE*	glycolate oxidase subunit GlcE	6.12	Carbon compound catabolism	Central intermediary metabolism	
		PA5355	*glcD*	glycolate oxidase subunit GlcD	4.47	Carbon compound catabolism	Central intermediary metabolism	
	Pentose and glucuronate interconversions	PA2022		probable nucleotide sugar dehydrogenase	18.44	Putative enzymes		
		PA2343	*mtlY*	xylulose kinase	2.41	Carbon compound catabolism		
	Fructose metabolism	PA3561	*fruK*	1-phosphofructokinase	2.19	Transport of small molecules	Central intermediary metabolism	
		PA3562	*fruI*	phosphotransferase system transporter enzyme I, FruI	2.75	Transport of small molecules	Central intermediary metabolism	
	Glycogen metabolism	PA2144	*glgP*	glycogen phosphorylase	-2.14	Cell Wall / LPS / Capsule		
		PA2160		probable glycosyl hydrolase	-2.65	Putative enzymes		
		PA2162		probable glycosyl hydrolase	-2.58	Putative enzymes		
		PA2163		hypothetical protein	-3.97	Hypothetical, unclassified, unknown		
		PA2164		probable glycosyl hydrolase	-2.60	Putative enzymes		
	Pyruvate metabolism	PA0836	*ackA*	acetate kinase	-4.95	Putative enzymes		
		PA1813		probable hydroxyacylglutathione hydrolase	-4.55	Adaptation, Protection	Putative enzymes	
		PA3471		probable malic enzyme	-3.83	Central intermediary metabolism		
		PA5435		probable transcarboxylase subunit	-3.14	Central intermediary metabolism		
		PA5436		probable biotin carboxylase subunit of a transcarboxylase	-2.03	Central intermediary metabolism		
	Glycerol metabolism and transport	PA3581	*glpF*	glycerol uptake facilitator protein	6.47	Transport of small molecules		
		PA3582	*glpK*	glycerol kinase	-2.66	Central intermediary metabolism		
		PA3583	*glpR*	glycerol-3-phosphate regulon repressor	-2.22	Transcriptional regulators		
		PA3584	*glpD*	glycerol-3-phosphate dehydrogenase	9.56	Energy metabolism	Central intermediary metabolism	
		PA3585	*glpM*	membrane protein GlpM	4.15	Membrane proteins	Transport of small molecules	
		PA5235	*glpT*	glycerol-3-phosphate transporter	6.04	Membrane proteins	Transport of small molecules	
	Aromatic compound degradation	PA2085		probable ring-hydroxylating dioxygenase small subunit	5.01	Carbon compound catabolism		
		PA2086		probable epoxide hydrolase	2.49	Carbon compound catabolism		
		PA4901	*mdlC*	benzoylformate decarboxylase	5.34	Carbon compound catabolism		
		PA4904	*vanA*	vanillate O-demethylase oxygenase subunit	5.10	Carbon compound catabolism		
	Cis-muconate transport	PA1019	*mucK*	cis,cis-muconate transporter MucK	13.12	Membrane proteins	Transport of small molecules	
	Chitin degradation	PA2300	*chiC*	chitinase	-18.87	Carbon compound catabolism		
	Carnitine metabolism	PA5384	*hocS*	HocS	4.25	Carbon compound catabolism	Central intermediary metabolism	
		PA5385	*cdhB*	CdhB, Carnitine dehydrogenase-related gene B	3.75	Carbon compound catabolism		
		PA5386	*cdhA*	CdhA, Carnitine dehydrogenase	6.44	Carbon compound catabolism	Putative enzymes	
		PA5387	*cdhC*	CdhC, Carnitine dehydrogenase-related gene C	7.58	Carbon compound catabolism		
		PA5388	*caiX*	CaiX	8.12	Carbon compound catabolism	Transport of small molecules	
	Acetamide metabolism	PA3363	*amiR*	aliphatic amidase regulator	-3.69	Transcriptional regulators		
		PA3364	*amiC*	aliphatic amidase expression-regulating protein	-2.35	Transcriptional regulators		
		PA3366.1	*amiL*	AmiL	24.51	Non-coding RNA gene		
	Terpenoid backbone biosynthesis	PA3803	*gcpE*	probable isoprenoid biosynthetic protein GcpE	-3.18	Putative enzymes		
		PA4557	*lytB*	LytB protein	-2.42	Adaptation, Protection		
	Citronellol catabolism	PA2886	*atuA*	expressed protein with apparent function in citronellol catabolism	6.07	Hypothetical, unclassified, unknown		
		PA2887	*atuB*	putative dehydrogenase involved in catabolism of citronellol	3.72	Putative enzymes		
	L-lyxonate metabolism	PA2214		putative L-lyxonate transporter	8.14	Membrane proteins	Transport of small molecules	
		PA2215	*lyxD*	l-lyxonate dehydratase LyxD	2.35	Hypothetical, unclassified, unknown		
		PA2216		2-keto-3-deoxy-D-arabinonate dehydratase	2.94	Hypothetical, unclassified, unknown		
	C4-dicarboxylate transport	PA0119		probable dicarboxylate transporter	11.25	Transport of small molecules		
		PA0884		probable C4-dicarboxylate-binding periplasmic protein	5.75	Transport of small molecules		
		PA0885		probable C4-dicarboxylate transporter	16.00	Transport of small molecules		
		PA0886		probable C4-dicarboxylate transporter	2.92	Transport of small molecules		
		PA1183	*dctA*	C4-dicarboxylate transport protein	2.77	Transport of small molecules		
		PA5166	*dctD*	DctD	-3.18	Two-component regulatory systems	Transcriptional regulators	Transport of small molecules
	C5-dicarboxylate transport	PA5530		C5-dicarboxylate transporter	16.56	Membrane proteins	Transport of small molecules	
		PA5512	*mifS*	MifS	3.88	Two-component regulatory systems	Cell Wall / LPS / Capsule	
	Glucose/Mannose transport	PA3187		probable ATP-binding component of ABC transporter	3.38	Transport of small molecules		
		PA3188		probable permease of ABC sugar transporter	4.47	Transport of small molecules		
		PA3189		probable permease of ABC sugar transporter	5.10	Transport of small molecules		
		PA3190		probable binding protein component of ABC sugar transporter	2.51	Transport of small molecules		
		PA3191	*gtrS*	glucose transport sensor, GtrS	2.56	Two-component regulatory systems		
	Ribose transport	PA1946	*rbsB*	binding protein component precursor of ABC ribose transporter	6.61	Transport of small molecules		
		PA1947	*rbsA*	ribose transport protein RbsA	10.85	Transport of small molecules		
		PA1948	*rbsC*	membrane protein component of ABC ribose transporter	7.49	Transport of small molecules		
		PA1949	*rbsR*	ribose operon repressor RbsR	2.75	Transcriptional regulators	Carbon compound catabolism	
	Citrate transport	PA5468		probable citrate transporter	5.68	Transport of small molecules		

	Gluconate transport	PA1051		probable transporter	3.53	Membrane proteins	Transport of small molecules	
		PA2322	*gntP*	GntP	6.01	Transport of small molecules		
	Gluconate metabolism	PA2320	*gntR*	transcriptional regulator GntR	2.08	Transcriptional regulators		
		PA4204	*ppgL*	periplasmic gluconolactonase, PpgL	6.20	Hypothetical, unclassified, unknown		
	2-ketogluconate transport	PA2262		probable 2-ketogluconate transporter	2.73	Membrane proteins	Transport of small molecules	
	L-lactate transport	PA4770	*lldP*	L-lactate permease	11.30	Transport of small molecules		
	Cis-aconitate transport	PA0755	*opdH*	cis-aconitate porin OpdH	7.10	Membrane proteins	Transport of small molecules	
	Polyhydroxyalkanoate biosynthesis	PA5056	*phaC1*	poly(3-hydroxyalkanoic acid) synthase 1	-6.37	Central intermediary metabolism		
		PA5057	*phaD*	poly(3-hydroxyalkanoic acid) depolymerase	-8.00	Carbon compound catabolism		
		PA5058	*phaC2*	poly(3-hydroxyalkanoic acid) synthase 2	-29.41	Central intermediary metabolism		
	Ornithine regulation	PA0831	*oruR*	transcriptional regulator OruR	2.43	Transcriptional regulators		
	Others	PA2414		L-sorbosone dehydrogenase	2.13	Carbon compound catabolism		
		PA2717	*cpo*	chloroperoxidase precursor	-8.00	Central intermediary metabolism		
		PA2726		probable radical activating enzyme	-2.86	Central intermediary metabolism		
		PA2787	*cpg2*	carboxypeptidase G2 precursor	-4.27	Central intermediary metabolism		
**Nitrogen metabolism**	Dissimilatory nitrate reduction	PA1172	*napC*	cytochrome c-type protein NapC	-19.23	Energy metabolism		
		PA1173	*napB*	cytochrome c-type protein NapB precursor	-11.11	Energy metabolism		
		PA1174	*napA*	periplasmic nitrate reductase protein NapA	-7.09	Energy metabolism		
		PA1175	*napD*	NapD protein of periplasmic nitrate reductase	-2.56	Energy metabolism		
		PA1176	*napF*	ferredoxin protein NapF	-3.62	Energy metabolism		
		PA1781	*nirB*	assimilatory nitrite reductase large subunit	2.19	Central intermediary metabolism		
		PA3875	*narG*	respiratory nitrate reductase alpha chain	-2.78	Energy metabolism		
		PA4922	*azu*	azurin precursor	-12.35	Energy metabolism		
	Denitrification	PA0509	*nirN*	NirN	-4.17	Biosynthesis of cofactors, prosthetic groups and carrier	Energy metabolism	
		PA0510	*nirE*	NirE	-6.71	Biosynthesis of cofactors, prosthetic groups and carrier	Energy metabolism	
		PA0511	*nirJ*	heme d1 biosynthesis protein NirJ	-5.75	Biosynthesis of cofactors, prosthetic groups and carrier	Energy metabolism	
		PA0512	*nirH*	NirH	-3.02	Biosynthesis of cofactors, prosthetic groups and carrier	Energy metabolism	
		PA0513	*nirG*	NirG	-17.54	Biosynthesis of cofactors, prosthetic groups and carrier	Energy metabolism	Transcriptional regulators
		PA0514	*nirL*	heme d1 biosynthesis protein NirL	-11.63	Biosynthesis of cofactors, prosthetic groups and carrier	Energy metabolism	
		PA0515		probable transcriptional regulator	-6.25	Biosynthesis of cofactors, prosthetic groups and carrier	Energy metabolism	Transcriptional regulators
		PA0516	*nirF*	heme d1 biosynthesis protein NirF	-6.62	Biosynthesis of cofactors, prosthetic groups and carrier	Energy metabolism	
		PA0517	*nirC*	probable c-type cytochrome precursor	-24.39	Biosynthesis of cofactors, prosthetic groups and carrier	Energy metabolism	
		PA0518	*nirM*	cytochrome c-551 precursor	-21.74	Biosynthesis of cofactors, prosthetic groups and carrier	Energy metabolism	
		PA0519	*nirS*	nitrite reductase precursor	-11.63	Energy metabolism		
		PA0524	*norB*	nitric-oxide reductase subunit B	-3.48	Energy metabolism		
		PA2611	*cysG*	siroheme synthase	-2.54	Biosynthesis of cofactors, prosthetic groups and carrier		
	Assimilatory nitrate reduction	PA1779		assimilatory nitrate reductase	2.65	Central intermediary metabolism		
	Transporters	PA0291	*oprE*	Anaerobically-induced outer membrane porin OprE precursor	-2.58	Membrane proteins	Transport of small molecules	
		PA1783	*nasA*	nitrate transporter	3.24	Membrane proteins	Transport of small molecules	
		PA3877	*narK1*	nitrite extrusion protein 1	5.03	Membrane proteins	Transport of small molecules	
	Nitrate regulation	PA0527	*dnr*	transcriptional regulator Dnr	-7.25	Transcriptional regulators		
		PA1544	*anr*	transcriptional regulator Anr	-2.94	Transcriptional regulators		
		PA1781.1	*nalA*	nitrogen assimilation leader A (NalA)	9.79	Non-coding RNA gene		
		PA1786	*nasS*	NasS	2.03	Hypothetical, unclassified, unknown		
	Nitrous oxyde regulation	PA3391	*nosR*	regulatory protein NosR	7.29	Membrane proteins	Energy metabolism	
	Unknown	PA0660		NAD(P)H:quinone reductase	-6.58	Hypothetical, unclassified, unknown		
		PA3658	*glnD*	protein-PII uridylyltransferase	-2.21	Amino acid biosynthesis and metabolism		
		PA4464	*ptsN*	nitrogen regulatory IIA protein	-9.26	Transport of small molecules		
	Anaerobic respiration	PA0521		probable cytochrome c oxidase subunit	2.14	Energy metabolism		
		PA0522		hypothetical protein	2.93	Hypothetical, unclassified, unknown		
		PA3032	*snr1*	cytochrome c Snr1	-3.68	Energy metabolism		
		PA3621	*fdxA*	ferredoxin I	2.26	Energy metabolism		
		PA4809	*fdhE*	FdhE protein	-5.38	Energy metabolism		
		PA4810	*fdnI*	nitrate-inducible formate dehydrogenase, gamma subunit	-3.30	Energy metabolism		
		PA4811	*fdnH*	nitrate-inducible formate dehydrogenase, beta subunit	-3.98	Energy metabolism		
		PA4812	*fdnG*	formate dehydrogenase-O, major subunit	-2.63	Energy metabolism		
	Cyanate metabolism	PA2052	*cynS*	cyanate lyase	-14.29	Central intermediary metabolism		
		PA2053	*cynT*	carbonate dehydratase	-2.04	Adaptation, Protection	Central intermediary metabolism	
	Formate metabolism	PA3628		putative esterase	-4.27	Putative enzymes		
	Ammonium transporter	PA3039		probable transporter	6.77	Membrane proteins	Transport of small molecules	
	Nitrogen utilization regulation	PA5125	*ntrC*	two-component response regulator NtrC	-4.88	Two-component regulatory systems	Transcriptional regulators	
**Sulfur metabolism**	Sulfate transport	PA0029		probable sulfate transporter	2.71	Membrane proteins	Transport of small molecules	
		PA0280	*cysA*	sulfate transport protein CysA	-7.19	Transport of small molecules		
		PA0281	*cysW*	sulfate transport protein CysW	-2.49	Membrane proteins	Transport of small molecules	
		PA0846		probable sulfate uptake protein	3.34	Transport of small molecules		
		PA1493	*cysP*	sulfate-binding protein of ABC transporter	-4.08	Transport of small molecules		
		PA2563		probable sulfate transporter	7.86	Membrane proteins	Transport of small molecules	
	Taurine transport	PA3936		probable permease of ABC taurine transporter	4.49	Transport of small molecules	Membrane proteins	
		PA3937		probable ATP-binding component of ABC taurine transporter	5.05	Transport of small molecules		
		PA3938		probable periplasmic taurine-binding protein precursor	6.09	Transport of small molecules		
	Alkane sulfonate transport	PA0184		probable ATP-binding component of ABC transporter	2.36	Transport of small molecules		
		PA0185		probable permease of ABC transporter	6.44	Membrane proteins	Transport of small molecules	
		PA0186		probable binding protein component of ABC transporter	7.85	Transport of small molecules		
		PA2594		conserved hypothetical protein	2.05	Hypothetical, unclassified, unknown		
		PA2595		conserved hypothetical protein	3.34	Hypothetical, unclassified, unknown		
		PA2596		conserved hypothetical protein	2.17	Hypothetical, unclassified, unknown		
		PA3442		probable ATP-binding component of ABC transporter	3.33	Transport of small molecules		
		PA3443		probable permease of ABC transporter	4.30	Membrane proteins	Transport of small molecules	
		PA3445		conserved hypothetical protein	5.10	Hypothetical, unclassified, unknown		
		PA3447		probable ATP-binding component of ABC transporter	6.45	Transport of small molecules		
		PA3448		probable permease of ABC transporter	4.21	Membrane proteins	Transport of small molecules	
		PA3449		conserved hypothetical protein	5.50	Hypothetical, unclassified, unknown		
	Assimilatory sulfate reduction	PA5175	*cysQ*	3,5-bisphosphate nucleotidase CysQ	3.91	Putative enzymes		
	Sulfite metabolism	PA0589		conserved hypothetical protein	-2.71	Energy metabolism		
		PA1838	*cysI*	sulfite reductase	-2.37	Central intermediary metabolism		
		PA2310		hypothetical protein	4.75	Putative enzymes		
		PA2345		conserved hypothetical protein	-6.21	Hypothetical, unclassified, unknown		
		PA2354	*sfnR1*	SfnR1	3.83	Transcriptional regulators		
		PA2356	*msuD*	methanesulfonate sulfonatase MsuD	7.66	Carbon compound catabolism	Central intermediary metabolism	
		PA2357	*msuE*	NADH-dependent FMN reductase MsuE	5.70	Carbon compound catabolism	Central intermediary metabolism	
		PA2566		conserved hypothetical protein	-4.12	Hypothetical, unclassified, unknown		
		PA2600		hypothetical protein	12.37	Hypothetical, unclassified, unknown		
		PA3444		conserved hypothetical protein	3.69	Putative enzymes		
		PA3446		conserved hypothetical protein	4.92	Hypothetical, unclassified, unknown		
**Phosphate metabolism**	Phosphate limitation	PA0674	*vreA*	VreA	7.06	Transcriptional regulators		
		PA0675	*vreI*	ECF sigma factor, VreI	12.05	Transcriptional regulators		
		PA0676	*vreR*	sigma factor regulator, VreR	4.01	Membrane proteins	Transcriptional regulators	
		PA0690	*pdtA*	phosphate depletion regulated TPS partner A, PdtA	6.13	Hypothetical, unclassified, unknown		
		PA0692	*pdtB*	phosphate depletion regulated TPS partner B, PdtB	13.59	Hypothetical, unclassified, unknown		
		PA0450		probable phosphate transporter	18.76	Membrane proteins	Transport of small molecules	
		PA3279	*oprP*	Phosphate-specific outer membrane porin OprP precursor	5.07	Transport of small molecules		
		PA3280	*oprO*	Pyrophosphate-specific outer membrane porin OprO precursor	10.50	Transport of small molecules		
		PA5368	*pstC*	membrane protein component of ABC phosphate transporter	2.58	Transport of small molecules	Membrane proteins	
	Phosphate metabolism	PA0141		conserved hypothetical protein	-7.46	Hypothetical, unclassified, unknown		
		PA4031	*ppa*	inorganic pyrophosphatase	-2.32	Central intermediary metabolism		
		PA5242	*ppk*	polyphosphate kinase	-2.69	Nucleotide biosynthesis and metabolism	Adaptation, Protection	
	Phosphonate transport	PA3382	*phnE*	phosphonate transport protein PhnE	2.40	Transport of small molecules	Membrane proteins	
		PA3384	*phnC*	ATP-binding component of ABC phosphonate transporter	2.41	Transport of small molecules		
**Nucleotide metabolism**	Purine metabolism	PA0134		probable guanine deaminase	5.13	Nucleotide biosynthesis and metabolism		
		PA0143	*nuh*	purine nucleosidase Nuh	-5.95	Nucleotide biosynthesis and metabolism		
		PA0148		adenine deaminase	-2.38	Nucleotide biosynthesis and metabolism		
		PA0387		conserved hypothetical protein	-9.62	Hypothetical, unclassified, unknown		
		PA0590	*apaH*	bis(5'-nucleosyl)-tetraphosphatase	-2.70	Nucleotide biosynthesis and metabolism		
		PA1013	*purC*	phosphoribosylaminoimidazole-succinocarboxamide synthase	-4.44	Nucleotide biosynthesis and metabolism		
		PA1124	*dgt*	deoxyguanosinetriphosphate triphosphohydrolase	-2.73	Nucleotide biosynthesis and metabolism		
		PA1140		conserved hypothetical protein	-2.18	Hypothetical, unclassified, unknown		
		PA1393	*cysC*	adenosine 5'-phosphosulfate (APS) kinase	2.24	Amino acid biosynthesis and metabolism	Nucleotide biosynthesis and metabolism	Central intermediary metabolism
		PA1514		ureidoglycolate hydrolaseYbbT	-4.78	Hypothetical, unclassified, unknown		
		PA1515	*alc*	allantoicase	-2.26	Carbon compound catabolism		
		PA1521		probable guanine deaminase	-2.50	Nucleotide biosynthesis and metabolism		
		PA1523	*xdhB*	xanthine dehydrogenase	-2.67	Nucleotide biosynthesis and metabolism		
		PA1543	*apt*	adenine phosphoribosyltransferase	-2.09	Nucleotide biosynthesis and metabolism		
		PA1931		probable ferredoxin	-2.95	Energy metabolism	Carbon compound catabolism	
		PA3043		conserved hypothetical protein	-3.09	Hypothetical, unclassified, unknown		
		PA3686	*adk*	adenylate kinase	-3.38	Nucleotide biosynthesis and metabolism		
		PA3751	*purT*	phosphoribosylglycinamide formyltransferase 2	-2.84	Amino acid biosynthesis and metabolism	Nucleotide biosynthesis and metabolism	
		PA3769	*guaA*	GMP synthase	-2.19	Amino acid biosynthesis and metabolism	Nucleotide biosynthesis and metabolism	
		PA3970	*amn*	AMP nucleosidase	-2.39	Nucleotide biosynthesis and metabolism		
		PA4855	*purD*	phosphoribosylamine--glycine ligase	-2.02	Nucleotide biosynthesis and metabolism		
		PA4971	*aspP*	adenosine diphosphate sugar pyrophosphatase	4.50	Energy metabolism		
		PA5420	*purU2*	formyltetrahydrofolate deformylase	-2.31	Nucleotide biosynthesis and metabolism		
		PA5426	*purE*	phosphoribosylaminoimidazole carboxylase, catalytic subunit	2.43	Nucleotide biosynthesis and metabolism		
	Pyrimidine metabolism	PA0401		noncatalytic dihydroorotase-like protein	-4.88	Nucleotide biosynthesis and metabolism		
		PA0402	*pyrB*	aspartate carbamoyltransferase	-4.29	Amino acid biosynthesis and metabolism	Nucleotide biosynthesis and metabolism	
		PA0403	*pyrR*	transcriptional regulator PyrR	-3.95	Transcriptional regulators	Nucleotide biosynthesis and metabolism	
		PA0437	*codA*	cytosine deaminase	-5.81	Nucleotide biosynthesis and metabolism		
		PA0439		probable oxidoreductase	2.14	Putative enzymes		
		PA0440		probable oxidoreductase	2.44	Putative enzymes		
		PA0441	*dht*	dihydropyrimidinase	8.79	Nucleotide biosynthesis and metabolism		
		PA0444		N-carbamoyl-beta-alanine amidohydrolase	4.72	Nucleotide biosynthesis and metabolism		
		PA3163	*cmk*	cytidylate kinase	-6.90	Nucleotide biosynthesis and metabolism		
		PA3480		probable deoxycytidine triphosphate deaminase	-2.18	Nucleotide biosynthesis and metabolism		
		PA3527	*pyrC*	dihydroorotase	-3.16	Nucleotide biosynthesis and metabolism		
		PA3637	*pyrG*	CTP synthase	-2.61	Nucleotide biosynthesis and metabolism		
		PA5321	*dut*	deoxyuridine 5'-triphosphate nucleotidohydrolase	-6.54	Nucleotide biosynthesis and metabolism		
		PA5541	*pyrQ*	dihydroorotase	4.32	Nucleotide biosynthesis and metabolism		
	Purine/Pyrimidine metabolism	PA1155	*nrdB*	NrdB, tyrosyl radical-harboring component of class Ia ribonucleotide reductase	-10.20	Nucleotide biosynthesis and metabolism		
		PA1156	*nrdA*	NrdA, catalytic component of class Ia ribonucleotide reductase	-5.92	Nucleotide biosynthesis and metabolism		
		PA1920	*nrdD*	class III (anaerobic) ribonucleoside-triphosphate reductase subunit, NrdD	3.86	Nucleotide biosynthesis and metabolism		
		PA4740	*pnp*	polyribonucleotide nucleotidyltransferase	-4.65	Transcription, RNA processing and degradation		
		PA5496	*nrdJb*	class II (cobalamin-dependent) ribonucleotide-diphosphate reductase subunit, NrdJb	-13.51	Nucleotide biosynthesis and metabolism		
		PA5497	*nrdJa*	class II (cobalamin-dependent) ribonucleotide-diphosphate reductase subunit, NrdJa	-4.93	Nucleotide biosynthesis and metabolism		
	ppGpp metabolism	PA5338	*spoT*	guanosine-3',5'-bis(diphosphate) 3'-pyrophosphohydrolase	-2.05	Adaptation, Protection	Nucleotide biosynthesis and metabolism	
	N-Acetylglucosamine catabolism	PA3758		probable N-acetylglucosamine-6-phosphate deacetylase	-2.99	Central intermediary metabolism		
		PA3759		probable aminotransferase	-3.27	Putative enzymes		
		PA3760		N-Acetyl-D-Glucosamine phosphotransferase system transporter	-2.10	Transport of small molecules		
	Uracil transport	PA4647	*uraA*	uracil permease	-2.16	Transport of small molecules	Membrane proteins	
	Nucleobases transport	PA0166		probable transporter	15.91	Membrane proteins	Transport of small molecules	
		PA0352		probable transporter	3.74	Membrane proteins	Transport of small molecules	
		PA0443		probable transporter	27.75	Membrane proteins	Transport of small molecules	
		PA1507		probable transporter	5.65	Membrane proteins	Transport of small molecules	
		PA1519		probable transporter	3.13	Membrane proteins	Transport of small molecules	
		PA2938		probable transporter	8.47	Membrane proteins	Transport of small molecules	
	Queuosine transport	PA0013		conserved hypothetical protein	5.44	Membrane proteins		
**c-di-GMP metabolism**	GGDEF domain	PA1851		hypothetical protein	3.70	Membrane proteins		
		PA2870		diguanylate cyclase	2.96	Nucleotide biosynthesis and metabolism		
		PA3177		diguanylate cyclase	11.63	Hypothetical, unclassified, unknown		
		PA3343	*hsbD*	diguanylate cyclase HsbD	2.01	Motility and Attachment		
		PA3702	*wspR*	WspR	-2.58	Motility and Attachment	Chemotaxis	Nucleotide biosynthesis and metabolism
		PA4843	*gcbA*	GcbA	3.15	Two-component regulatory systems	Transcriptional regulators	
		PA4929		hypothetical protein	-4.41	Membrane proteins		
	EAL domain	PA2133		Cyclic-guanylate-specific phosphodiesterase	2.32	Hypothetical, unclassified, unknown		
		PA3947	*rocR*	RocR	4.46	Nucleotide biosynthesis and metabolism		
	HD-GYP domain	PA2572		probable two-component response regulator	-13.33	Two-component regulatory systems	Transcriptional regulators	
		PA4108		cyclic di-GMP phosphodiesterase	-3.17	Cell Wall / LPS / Capsule	Motility and Attachment	
		PA4781		cyclic di-GMP phosphodiesterase	-3.36	Cell Wall / LPS / Capsule	Transcriptional regulators	Motility and Attachment
	GGDEF+EAL domains	PA2072		conserved hypothetical protein	-2.28	Membrane proteins		
		PA3258		hypothetical protein	2.30	Hypothetical, unclassified, unknown		
		PA3311	*nbdA*	NbdA	-4.88	Membrane proteins		
		PA4601	*morA*	motility regulator	-5.56	Membrane proteins		
		PA4959	*fimX*	FimX	-3.80	Motility and Attachment		
		PA5017	*dipA*	DipA	2.39	Hypothetical, unclassified, unknown		
		PA5442		conserved hypothetical protein	7.21	Membrane proteins		
	Others	PA2771		diguanylate cyclase with a self-blocked I-site, Dcsbis	-27.03	Motility and Attachment		
	Valine, leucine and isoleucine degradation	PA2011	*liuE*	3-hydroxy-3-methylglutaryl-CoA lyase	-6.13	Carbon compound catabolism		
		PA2012	*liuD*	methylcrotonyl-CoA carboxylase, alpha-subunit (biotin-containing)	-7.04	Carbon compound catabolism		
		PA2013	*liuC*	putative 3-methylglutaconyl-CoA hydratase	-5.71	Carbon compound catabolism		
		PA2014	*liuB*	methylcrotonyl-CoA carboxylase, beta-subunit	-4.12	Carbon compound catabolism		
		PA2015	*liuA*	putative isovaleryl-CoA dehydrogenase	-3.12	Carbon compound catabolism		
		PA2016	*liuR*	regulator of liu genes	-2.92	Transcriptional regulators		
		PA2247	*bkdA1*	2-oxoisovalerate dehydrogenase (alpha subunit)	-3.68	Amino acid biosynthesis and metabolism		
		PA2248	*bkdA2*	2-oxoisovalerate dehydrogenase (beta subunit)	-5.24	Amino acid biosynthesis and metabolism		
		PA2249	*bkdB*	branched-chain alpha-keto acid dehydrogenase (lipoamide component)	-4.63	Amino acid biosynthesis and metabolism		
		PA2250	*lpdV*	lipoamide dehydrogenase-Val	-4.69	Amino acid biosynthesis and metabolism	Energy metabolism	
		PA3418	*ldh*	leucine dehydrogenase	-5.13	Amino acid biosynthesis and metabolism		
		PA3570	*mmsA*	methylmalonate-semialdehyde dehydrogenase	-3.89	Amino acid biosynthesis and metabolism	Carbon compound catabolism	
		PA3569	*mmsB*	3-hydroxyisobutyrate dehydrogenase	-5.03	Carbon compound catabolism		
		PA5013	*ilvE*	branched-chain amino acid transferase	-3.45	Amino acid biosynthesis and metabolism		
		PA5308	*lrp*	leucine-responsive regulatory protein	-2.96	Transcriptional regulators	Central intermediary metabolism	
	Valine, leucine and isoleucine biosynthesis	PA0851		hypothetical protein	-7.87	Hypothetical, unclassified, unknown		
		PA1217		probable 2-isopropylmalate synthase	-35.71	Amino acid biosynthesis and metabolism		
		PA3118	*leuB*	3-isopropylmalate dehydrogenase	-3.66	Amino acid biosynthesis and metabolism		
		PA3120	*leuD*	3-isopropylmalate dehydratase small subunit	-2.46	Amino acid biosynthesis and metabolism		
		PA3792	*leuA*	2-isopropylmalate synthase	-7.46	Amino acid biosynthesis and metabolism		
		PA4180		probable acetolactate synthase large subunit	-2.84	Amino acid biosynthesis and metabolism		
		PA4694	*ilvC*	ketol-acid reductoisomerase	-3.40	Amino acid biosynthesis and metabolism	Biosynthesis of cofactors, prosthetic groups and carrier	
		PA4695	*ilvH*	acetolactate synthase isozyme III small subunit	-2.21	Amino acid biosynthesis and metabolism	Biosynthesis of cofactors, prosthetic groups and carrier	
	Alanine, aspartate and glutamate metabolism	PA0132	*bauA*	Beta-alanine:pyruvate transaminase	3.30	Carbon compound catabolism	Amino acid biosynthesis and metabolism	
		PA0133	*bauR*	BauR	8.90	Transcriptional regulators	Carbon compound catabolism	
		PA0600	*agtS*	two-component sensor, AgtS	3.38	Two-component regulatory systems		
		PA0904	*lysC*	aspartate kinase alpha and beta chain	-2.20	Amino acid biosynthesis and metabolism		
		PA1337	*ansB*	glutaminase-asparaginase	-11.11	Amino acid biosynthesis and metabolism		
		PA2113	*opdO*	pyroglutamate porin OpdO	-19.61	Membrane proteins	Transport of small molecules	
		PA2253	*ansA*	L-asparaginase I	-2.93	Amino acid biosynthesis and metabolism		
		PA3068	*gdhB*	NAD-dependent glutamate dehydrogenase	-8.20	Amino acid biosynthesis and metabolism		
		PA3117	*asd*	aspartate semialdehyde dehydrogenase	-2.01	Amino acid biosynthesis and metabolism		
		PA3517		probable lyase	3.21	Carbon compound catabolism		
		PA3602		conserved hypothetical protein	-6.06	Membrane proteins		
		PA3797		conserved hypothetical protein	-2.82	Hypothetical, unclassified, unknown		
		PA4731	*panD*	aspartate 1-decarboxylase precursor	-2.02	Amino acid biosynthesis and metabolism	Biosynthesis of cofactors, prosthetic groups and carrier	
		PA4756	*carB*	carbamoylphosphate synthetase large subunit	-5.24	Amino acid biosynthesis and metabolism	Nucleotide biosynthesis and metabolism	
		PA4758	*carA*	carbamoyl-phosphate synthase small chain	-2.38	Amino acid biosynthesis and metabolism	Nucleotide biosynthesis and metabolism	
		PA4930	*alr*	biosynthetic alanine racemase	3.79	Amino acid biosynthesis and metabolism		
		PA5119	*glnA*	glutamine synthetase	-5.85	Amino acid biosynthesis and metabolism		
		PA5203	*gshA*	glutamate--cysteine ligase	-6.10	Amino acid biosynthesis and metabolism	Biosynthesis of cofactors, prosthetic groups and carrier	
		PA5302	*dadX*	catabolic alanine racemase	-4.95	Amino acid biosynthesis and metabolism		
		PA5549	*glmS*	glucosamine--fructose-6-phosphate aminotransferase	-6.58	Central intermediary metabolism		
	Glycine, serine and threonine metabolism	PA0421		hypothetical protein	-2.18	Putative enzymes		
		PA0743		probable 3-hydroxyisobutyrate dehydrogenase	-13.33	Carbon compound catabolism		
		PA0902		hypothetical protein	-2.11	Hypothetical, unclassified, unknown		
		PA2442	*gcvT2*	glycine cleavage system protein T2	-6.80	Amino acid biosynthesis and metabolism	Central intermediary metabolism	
		PA2443	*sdaA*	L-serine dehydratase	-5.92	Amino acid biosynthesis and metabolism		
		PA2444	*glyA2*	serine hydroxymethyltransferase	-7.19	Amino acid biosynthesis and metabolism		
		PA2445	*gcvP2*	glycine cleavage system protein P2	-6.33	Amino acid biosynthesis and metabolism	Central intermediary metabolism	
		PA2446	*gcvH2*	glycine cleavage system protein H2	-3.76	Amino acid biosynthesis and metabolism		
		PA2449	*gcsR*	GcsR	3.02	Transcriptional regulators		
		PA3357	*dsdA*	D-serine dehydratase	2.14	Amino acid biosynthesis and metabolism		
		PA3735	*thrC*	threonine synthase	-3.21	Amino acid biosynthesis and metabolism	Biosynthesis of cofactors, prosthetic groups and carrier	
		PA4960		probable phosphoserine phosphatase	2.77	Amino acid biosynthesis and metabolism		
		PA5213	*gcvP1*	glycine cleavage system protein P1	-4.83	Amino acid biosynthesis and metabolism	Central intermediary metabolism	
		PA5214	*gcvH1*	glycine cleavage system protein H1	-2.44	Amino acid biosynthesis and metabolism	Central intermediary metabolism	
		PA5379	*sdaB*	L-serine dehydratase	3.72	Amino acid biosynthesis and metabolism		
		PA5413	*ltaA*	low specificity l-threonine aldolase	-7.04	Amino acid biosynthesis and metabolism		
		PA5415	*glyA1*	serine hydroxymethyltransferase	-7.04	Amino acid biosynthesis and metabolism		
		PA5416	*soxB*	sarcosine oxidase beta subunit	-2.26	Amino acid biosynthesis and metabolism		
		PA5418	*soxA*	sarcosine oxidase alpha subunit	-2.81	Carbon compound catabolism		
		PA5419	*soxG*	sarcosine oxidase gamma subunit	-2.09	Amino acid biosynthesis and metabolism		
	Cysteine and methionine metabolism	PA0399		cystathionine beta-synthase	-10.10	Amino acid biosynthesis and metabolism		
		PA0400		probable cystathionine gamma-lyase	-12.82	Amino acid biosynthesis and metabolism		
		PA0407	*gshB*	glutathione synthetase	-5.29	Amino acid biosynthesis and metabolism	Biosynthesis of cofactors, prosthetic groups and carrier	
		PA0432	*sahH*	S-adenosyl-L-homocysteine hydrolase	-2.21	Amino acid biosynthesis and metabolism		
		PA0546	*metK*	methionine adenosyltransferase	-13.70	Amino acid biosynthesis and metabolism	Central intermediary metabolism	
		PA1061		conserved hypothetical protein	-7.87	Putative enzymes		
		PA1292		probable 3-mercaptopyruvate sulfurtransferase	-5.59	Putative enzymes		
		PA1683		probable sugar aldolase	-4.52	Putative enzymes		
		PA1684		1,2-dihydroxy-3-keto-5-methylthiopentene dioxygenase MtnD	-2.34	Amino acid biosynthesis and metabolism		
		PA1685	*masA*	enolase-phosphatase E-1	-4.42	Amino acid biosynthesis and metabolism		
		PA1843	*metH*	methionine synthase	-3.45	Amino acid biosynthesis and metabolism		
		PA2104		probable cysteine synthase	3.71	Amino acid biosynthesis and metabolism		
		PA2105		probable acetyltransferase	6.14	Putative enzymes		
		PA2602		3-mercaptopropionate dioxygenase	-2.50	Hypothetical, unclassified, unknown		
		PA2709	*cysK*	cysteine synthase A	-5.18	Amino acid biosynthesis and metabolism		
		PA3107	*metZ*	o-succinylhomoserine sulfhydrylase	-2.04	Amino acid biosynthesis and metabolism		
		PA3816	*cysE*	O-acetylserine synthase	5.49	Biosynthesis of cofactors, prosthetic groups and carrier	Amino acid biosynthesis and metabolism	
		PA5025	*metY*	homocysteine synthase	-6.10	Amino acid biosynthesis and metabolism		
		PA0430	*metF*	5,10-methylenetetrahydrofolate reductase	-3.91	Amino acid biosynthesis and metabolism	Central intermediary metabolism	
	Lysine metabolism	PA0447	*gcdH*	glutaryl-CoA dehydrogenase	2.16	Fatty acid and phospholipid metabolism	Amino acid biosynthesis and metabolism	Carbon compound catabolism
		PA0448	*gcdR*	transcriptional regulator, GcdR	2.38	Transcriptional regulators		
		PA0530		probable class III pyridoxal phosphate-dependent aminotransferase	3.24	Putative enzymes		
		PA1818	*cadA*	lysine decarboxylase	-3.72	Amino acid biosynthesis and metabolism		
		PA3659		probable aminotransferase	-5.95	Putative enzymes		
		PA3666	*dapD*	tetrahydrodipicolinate succinylase	-2.99	Cell Wall / LPS / Capsule		
		PA4188		conserved hypothetical protein	3.09	Hypothetical, unclassified, unknown		
		PA4914	*amaR*	transcriptional regulator, AmaR	-2.93	Transcriptional regulators		
		PA5277	*lysA*	diaminopimelate decarboxylase	-3.10	Amino acid biosynthesis and metabolism		
		PA5278	*dapF*	diaminopimelate epimerase	-4.05	Amino acid biosynthesis and metabolism		
	Arginine biosynthesis	PA1195	*ddaH*	dimethylarginine dimethylaminohydrolase DdaH	12.24	Amino acid biosynthesis and metabolism		
		PA1196	*ddaR*	transcriptional regulator DdaR	3.23	Transcriptional regulators		
		PA3525	*argG*	argininosuccinate synthase	3.12	Amino acid biosynthesis and metabolism		
		PA3537	*argF*	ornithine carbamoyltransferase, anabolic	2.35	Amino acid biosynthesis and metabolism		
		PA4402	*argJ*	glutamate N-acetyltransferase	-6.58	Amino acid biosynthesis and metabolism		
		PA5170	*arcD*	arginine/ornithine antiporter	-9.01	Amino acid biosynthesis and metabolism	Membrane proteins	Transport of small molecules
		PA5171	*arcA*	arginine deiminase	-29.41	Amino acid biosynthesis and metabolism		
		PA5172	*arcB*	ornithine carbamoyltransferase, catabolic	-45.45	Amino acid biosynthesis and metabolism		
		PA5173	*arcC*	carbamate kinase	-35.71	Amino acid biosynthesis and metabolism		
		PA5206	*argE*	acetylornithine deacetylase	-2.03	Amino acid biosynthesis and metabolism		
		PA5323	*argB*	acetylglutamate kinase	-2.19	Amino acid biosynthesis and metabolism		

		PA5390		probable peptidic bond hydrolase	2.02	Putative enzymes		
Aminoacid metabolism	Arginine catabolism	PA0287	gpuP	3-guanidinopropionate transport protein	8.04	Transport of small molecules		
		PA0289	gpuR	transcriptional activator GpuR	-2.32	Transcriptional regulators		
		PA0294	aguR	transcriptional regulator AguR	-2.30	Transcriptional regulators		
		PA1422	gbuR	GbuR	3.15	Transcriptional regulators		
	Arginine and proline metabolism	PA0782	putA	proline dehydrogenase PutA	8.27	Amino acid biosynthesis and metabolism		
		PA0893	argR	transcriptional regulator ArgR	-4.98	Amino acid biosynthesis and metabolism	Transcriptional regulators	
		PA0895	aruC	N2-Succinylornithine 5-aminotransferase (SOAT) = N2-acetylornithine 5-aminotransferase (ACOAT)	-3.56	Amino acid biosynthesis and metabolism		
		PA0896	aruF	subunit I of arginine N2-succinyltransferase = ornithine N2-succinyltransferase	-2.80	Amino acid biosynthesis and metabolism		
		PA0897	aruG	subunit II of arginine N2-succinyltransferase = ornithine N2-succinyltransferase	-3.33	Amino acid biosynthesis and metabolism		
		PA0898	aruD	N-Succinylglutamate 5-semialdehyde dehydrogenase	-4.59	Amino acid biosynthesis and metabolism		
		PA0899	aruB	N2-Succinylarginine dihydrolase	-8.06	Amino acid biosynthesis and metabolism		
		PA0901	aruE	N-Succinylglutamate desuccinylase	-4.85	Amino acid biosynthesis and metabolism		
		PA1253	lhpG	alpha-ketoglutaric semialdehyde dehydrogenase, LhpG	4.63	Amino acid biosynthesis and metabolism	Putative enzymes	
		PA1254	lhpC	delta1-pyrroline-4-hydroxy-2-carboxylate deaminase, LphC	5.02	Amino acid biosynthesis and metabolism	Putative enzymes	
		PA1258	lhpM	Permease of ABC transporter, LhpM	6.17	Amino acid biosynthesis and metabolism	Membrane proteins	Transport of small molecules
		PA1259	lhpH	LhpH	9.73	Amino acid biosynthesis and metabolism		
		PA1260	lhpP	ABC transporter periplasmic-binding protein, LhpP	10.74	Amino acid biosynthesis and metabolism	Transport of small molecules	
		PA1266	lhpE	D-hydroxyproline dehydrogenase alpha-subunit, LphE	2.69	Amino acid biosynthesis and metabolism	Putative enzymes	
		PA1267	lhpB	D-hydroxyproline dehydrogenase beta-subunit, LphB	2.31	Amino acid biosynthesis and metabolism		
		PA1268	lhpA	Hydroxyproline 2-epimerase, LhpA	3.49	Amino acid biosynthesis and metabolism		
		PA3863	dauA	FAD-dependent catabolic D-arginine dehydrogenase, DauA	-2.84	Amino acid biosynthesis and metabolism		
		PA4565	proB	glutamate 5-kinase	8.30	Amino acid biosynthesis and metabolism		
		PA4908		hypothetical protein	2.24	Hypothetical, unclassified, unknown		
		PA4976	aruH	Arginine:Pyruvate Transaminas, AruH	-3.22	Amino acid biosynthesis and metabolism		
		PA4977	aruI	2-ketoarginine decarboxylase, AruI	2.24	Amino acid biosynthesis and metabolism		
	Histidine metabolism	PA2531		probable aminotransferase	-10.00	Amino acid biosynthesis and metabolism		
		PA3151	hisF2	imidazoleglycerol-phosphate synthase, cyclase subunit	-6.45	Amino acid biosynthesis and metabolism		
		PA3152	hisH2	glutamine amidotransferase	-4.31	Amino acid biosynthesis and metabolism		
		PA3165	hisC2	histidinol-phosphate aminotransferase	-3.22	Amino acid biosynthesis and metabolism		
		PA4447	hisC1	histidinol-phosphate aminotransferase	-4.07	Amino acid biosynthesis and metabolism		
		PA4939		conserved hypothetical protein	2.84	Hypothetical, unclassified, unknown		
		PA5091	hutG	N-formylglutamate amidohydrolase	-3.66	Amino acid biosynthesis and metabolism		
		PA5092	hutI	imidazolone-5-propionate hydrolase HutI	-2.40	Amino acid biosynthesis and metabolism		
		PA5105	hutC	histidine utilization repressor HutC	2.36	Transcriptional regulators		
		PA5140	hisF1	imidazoleglycerol-phosphate synthase, cyclase subunit	-3.65	Amino acid biosynthesis and metabolism		
		PA5141	hisA	phosphoribosylformimino-5-aminoimidazole carboxamide	-3.08	Amino acid biosynthesis and metabolism		
		PA5142	hisH1	glutamine amidotransferase	-2.74	Amino acid biosynthesis and metabolism		
	Tyrosine metabolism	PA0242		hypothetical protein	9.35	Putative enzymes		
		PA0865	hpd	4-hydroxyphenylpyruvate dioxygenase	-4.13	Amino acid biosynthesis and metabolism		
		PA1966		hypothetical protein	4.69	Putative enzymes		
		PA2007	maiA	maleylacetoacetate isomerase	-8.33	Carbon compound catabolism		
		PA2008	fahA	fumarylacetoacetase	-5.65	Carbon compound catabolism		
		PA2009	hmgA	homogentisate 1,2-dioxygenase	-3.51	Carbon compound catabolism		
		PA2470	gtdA	gentisate 1,2-dioxygenase	4.08	Carbon compound catabolism		
		PA2471		conserved hypothetical protein	-2.98	Hypothetical, unclassified, unknown		
		PA3139		probable amino acid aminotransferase	-2.48	Amino acid biosynthesis and metabolism	Putative enzymes	
		PA4125	hpcD	5-carboxymethyl-2-hydroxymuconate isomerase	-3.33	Carbon compound catabolism		
	Phenylalanine metabolism	PA0704		probable amidase	-5.99	Putative enzymes		
		PA0870	phhC	aromatic amino acid aminotransferase	-5.41	Amino acid biosynthesis and metabolism		
		PA0871	phhB	pterin-4-alpha-carbinolamine dehydratase	-4.95	Amino acid biosynthesis and metabolism		
		PA0872	phhA	phenylalanine-4-hydroxylase	-5.56	Amino acid biosynthesis and metabolism		
		PA4073		probable aldehyde dehydrogenase	3.31	Putative enzymes		
		PA4342		probable amidase	2.99	Putative enzymes		
		PA4980		probable enoyl-CoA hydratase/isomerase	2.09	Putative enzymes		
	Tryptophan metabolism	PA2080	kynU	kynureninase KynU	-5.43	Amino acid biosynthesis and metabolism		
		PA0037	trpI	transcriptional regulator TrpI	6.09	Amino acid biosynthesis and metabolism	Transcriptional regulators	
	Phenylalanine , tyrosine and tryptophan biosynthesis	PA0244		hypothetical protein	17.27	Putative enzymes		
		PA0245	aroQ2	3-dehydroquinate dehydratase	15.67	Amino acid biosynthesis and metabolism		
		PA0609	trpE	anthranilate synthetase component I	-2.37	Amino acid biosynthesis and metabolism	Energy metabolism	
		PA2843		probable aldolase	-2.26	Putative enzymes		
		PA3166	pheA	chorismate mutase	-4.50	Amino acid biosynthesis and metabolism		
	D-glutamate catabolism	PA5082	dguC	DguC	2.17	Transport of small molecules		
		PA5083	dguB	Rid2 subfamily protein	5.18	Hypothetical, unclassified, unknown		
		PA5084	dguA	DguA	11.27	Putative enzymes		
		PA5085	dguR	DguR	2.53	Transcriptional regulators		
	Glutathione metabolism	PA0164		probable gamma-glutamyltranspeptidase	3.59	Amino acid biosynthesis and metabolism		
		PA1338	ggt	gamma-glutamyltranspeptidase precursor	-4.05	Amino acid biosynthesis and metabolism	Adaptation, Protection	Central intermediary metabolism
		PA2025	gor	glutathione reductase	-3.42	Amino acid biosynthesis and metabolism	Biosynthesis of cofactors, prosthetic groups and carrier	
	Paerucumarin biosynthesis	PA2254	pvcA	paerucumarin biosynthesis protein PvcA	-4.83	Amino acid biosynthesis and metabolism	Secreted Factors (toxins, enzymes, alginate)	
		PA2255	pvcB	paerucumarin biosynthesis protein PvcB	-3.86	Amino acid biosynthesis and metabolism	Secreted Factors (toxins, enzymes, alginate)	
		PA2256	pvcC	paerucumarin biosynthesis protein PvcC	-2.62	Amino acid biosynthesis and metabolism	Secreted Factors (toxins, enzymes, alginate)	
	Amino acid transporter	PA0136		probable ATP-binding component of ABC transporter	17.29	Transport of small molecules		
		PA0137		probable permease of ABC transporter	21.39	Membrane proteins	Transport of small molecules	
		PA0138		probable permease of ABC transporter	5.04	Membrane proteins	Transport of small molecules	
		PA0162	opdC	histidine porin OpdC	3.04	Membrane proteins	Transport of small molecules	
		PA0220		amino acid APC family transporter	7.22	Transport of small molecules		
		PA0313		L-cysteine transporter of ABC system YecS	-2.26	Membrane proteins	Transport of small molecules	
		PA0314		L-cysteine transporter of ABC system FliY	-4.27	Transport of small molecules		
		PA0322		probable transporter	2.49	Membrane proteins	Transport of small molecules	
		PA0328	aaaA	arginine-specific autotransporter of Pseudomonas aeruginosa, AaaA	7.75	Amino acid biosynthesis and metabolism		
		PA0783	putP	sodium/proline symporter PutP	3.04	Membrane proteins	Transport of small molecules	
		PA0789		probable amino acid permease	2.33	Membrane proteins	Transport of small molecules	
		PA0866	aroP2	aromatic amino acid transport protein AroP2	3.29	Transport of small molecules		
		PA0890	aotM	arginine/ornithine transport protein AotM	-2.36	Membrane proteins	Transport of small molecules	
		PA0892	aotP	arginine/ornithine transport protein AotP	-6.06	Transport of small molecules		
		PA1070	braG	branched-chain amino acid transport protein BraG	-5.13	Transport of small molecules		
		PA1071	braF	branched-chain amino acid transport protein BraF	-6.41	Transport of small molecules		
		PA1072	braE	branched-chain amino acid transport protein BraE	-4.20	Membrane proteins	Transport of small molecules	
		PA1147		probable amino acid permease	6.45	Membrane proteins	Transport of small molecules	
		PA1194		probable amino acid permease	18.00	Membrane proteins	Transport of small molecules	
		PA1335	aauR	AauR	-3.66	Two-component regulatory systems	Transcriptional regulators	
		PA1339	aatP	AatP	-4.26	Amino acid biosynthesis and metabolism	Transport of small molecules	
		PA1340	aatM	AatM	-5.71	Amino acid biosynthesis and metabolism	Transport of small molecules	
		PA1341	aatQ	AatQ	-4.72	Membrane proteins	Transport of small molecules	
		PA1342	aatJ	AatJ	-5.26	Amino acid biosynthesis and metabolism	Transport of small molecules	
		PA1485		probable amino acid permease	15.70	Membrane proteins	Transport of small molecules	
		PA1590	braB	branched chain amino acid transporter	-4.08	Transport of small molecules		
		PA1620		hypothetical protein	3.00	Membrane proteins		
		PA1807	nppD	NppD	-10.42	Transport of small molecules		
		PA1808	nppC	NppC	-12.05	Transport of small molecules		
		PA1809	nppB	NppB	-4.44	Transport of small molecules		
		PA1810	nppA2	NppA2	-4.13	Transport of small molecules		
		PA1916		probable amino acid permease	3.25	Transport of small molecules		
		PA1971	braZ	branched chain amino acid transporter BraZ	9.61	Transport of small molecules		
		PA2041		Amino acid permease	2.70	Transport of small molecules	Carbon compound catabolism	
		PA2079		probable amino acid permease	-3.39	Transport of small molecules		
		PA2505	opdT	tyrosine porin OpdT	7.39	Membrane proteins	Transport of small molecules	
		PA2916		hypothetical protein	4.08	Membrane proteins		
		PA2923	hisJ	periplasmic histidine-binding protein HisJ	6.04	Amino acid biosynthesis and metabolism	Transport of small molecules	
		PA2929		hypothetical protein	6.77	Membrane proteins		
		PA3176	gltS	glutamate/sodium ion symporter, GltS	2.71	Transport of small molecules		
		PA3597		probable amino acid permease	12.79	Membrane proteins	Transport of small molecules	
		PA3641		probable amino acid permease	2.46	Transport of small molecules		
		PA3766		probable aromatic amino acid transporter	35.23	Transport of small molecules		
		PA3858		probable amino acid-binding protein	-6.41	Transport of small molecules		
		PA4072		probable amino acid permease	7.23	Transport of small molecules		
		PA4365	lysE	Lysine efflux permease	2.78	Membrane proteins	Transport of small molecules	
		PA4496	dppA1	probable binding protein component of ABC transporter	-9.26	Transport of small molecules		
		PA4497	dppA2	probable binding protein component of ABC transporter	-12.50	Transport of small molecules		
		PA4502	dppA4	probable binding protein component of ABC transporter	-2.90	Transport of small molecules		
		PA4504	dppC	dipeptide ABC transporter permease DppC	-2.23	Transport of small molecules	Membrane proteins	
		PA4505	dppD	dipeptide ABC transporter ATP-binding protein DppD	-4.24	Transport of small molecules		
		PA4506	dppF	dipeptide ABC transporter ATP-binding protein DppF	-5.43	Transport of small molecules		
		PA4628	lysP	lysine-specific permease	-2.35	Transport of small molecules	Membrane proteins	
		PA4757		conserved hypothetical protein	-3.77	Membrane proteins		
		PA4898	opdK	histidine porin OpdK	8.42	Membrane proteins	Transport of small molecules	
		PA4911		probable permease of ABC branched-chain amino acid transporter	3.38	Transport of small molecules	Membrane proteins	
		PA4912		branched chain amino acid ABC transporter membrane protein	6.79	Transport of small molecules	Membrane proteins	
		PA4981	lysP	lysine-specific permease	2.23	Membrane proteins	Transport of small molecules	
		PA5153		amino acid (lysine/arginine/ornithine/histidine/octopine) ABC transporter periplasmic binding protein	-3.72	Transport of small molecules		
		PA5154		probable permease of ABC transporter	-2.16	Transport of small molecules	Membrane proteins	
		PA5155		amino acid (lysine/arginine/ornithine/histidine/octopine) ABC transporter membrane protein	-2.08	Transport of small molecules	Membrane proteins	
		PA5317	dppA5	probable binding protein component of ABC dipeptide transporter	-6.49	Transport of small molecules		
		PA5504		D-methionine ABC transporter membrane protein	4.51	Transport of small molecules	Membrane proteins	
	Polyamine biosynthesis	PA0296	spuI	Glutamylpolyamine synthetase	-2.28	Putative enzymes	Carbon compound catabolism	
		PA0297	spuA	probable glutamine amidotransferase	6.03	Carbon compound catabolism	Amino acid biosynthesis and metabolism	
		PA0534	pauB1	FAD-dependent oxidoreductase	2.96	Putative enzymes	Carbon compound catabolism	
		PA1409	aphA	acetylpolyamine aminohydrolase	3.76	Carbon compound catabolism		
		PA1565	pauB2	FAD-dependent oxidoreductase	2.90	Putative enzymes	Carbon compound catabolism	
		PA2776	pauB3	FAD-dependent oxidoreductase	4.13	Carbon compound catabolism		
		PA3356	pauA5	Glutamylpolyamine synthetase	-2.12	Carbon compound catabolism		
		PA5313	gabT2	Transaminase	2.45	Carbon compound catabolism	Putative enzymes	
		PA5522	pauA6	Glutamylpolyamine synthetase	6.36	Putative enzymes	Carbon compound catabolism	
	Polyamine transport	PA0295		probable periplasmic polyamine binding protein	-5.99	Transport of small molecules		
		PA0300	spuD	polyamine transport protein	-5.88	Transport of small molecules		
		PA0301	spuE	polyamine transport protein	-5.75	Transport of small molecules		
		PA0302	spuF	polyamine transport protein PotG	-5.41	Transport of small molecules		
		PA0303	spuG	polyamine transport protein PotH	-6.29	Membrane proteins	Transport of small molecules	
		PA0304	spuH	polyamine transport protein PotI	-3.30	Membrane proteins	Transport of small molecules	
		PA1410		probable periplasmic spermidine/putrescine-binding protein	2.75	Transport of small molecules		
		PA1540		conserved hypothetical protein	81.33	Membrane proteins		
		PA1541		probable drug efflux transporter	89.36	Membrane proteins	Transport of small molecules	
		PA2592		probable periplasmic spermidine/putrescine-binding protein	-3.33	Transport of small molecules		
		PA3607	potA	polyamine transport protein PotA	14.04	Transport of small molecules		
		PA3608	potB	polyamine transport protein PotB	9.79	Membrane proteins	Transport of small molecules	
		PA3609	potC	polyamine transport protein PotC	2.57	Membrane proteins	Transport of small molecules	
	Phosphonate and phosphinate metabolism	PA1310	phnW	2-aminoethylphosphonate:pyruvate aminotransferase	5.20	Carbon compound catabolism		
	Aeruginaldehyde biosynthesis	PA2302	ambE	AmbE	-14.49	Secreted Factors (toxins, enzymes, alginate)	Putative enzymes	
		PA2303	ambD	AmbD	-16.13	Secreted Factors (toxins, enzymes, alginate)		
		PA2304	ambC	AmbC	-11.24	Secreted Factors (toxins, enzymes, alginate)		
		PA2305	ambB	AmbB	-3.89	Secreted Factors (toxins, enzymes, alginate)	Putative enzymes	
		PA2306	ambA	AmbA	-6.41	Secreted Factors (toxins, enzymes, alginate)	Membrane proteins	
Fatty acid and phospholipid metabolism	Fatty acid degradation	PA0879		probable acyl-CoA dehydrogenase	2.11	Putative enzymes		
		PA1187		probable acyl-CoA dehydrogenase	2.98	Putative enzymes		
		PA1525	alkB2	alkane-1-monooxygenase 2	2.61	Carbon compound catabolism		
		PA1535		citronellyl-CoA dehydrogenase	6.55	Putative enzymes		
		PA2001	atoB	acetyl-CoA acetyltransferase	-5.46	Fatty acid and phospholipid metabolism	Central intermediary metabolism	
		PA2574	alkB1	alkane-1-monooxygenase	5.22	Carbon compound catabolism		
		PA2815		probable acyl-CoA dehydrogenase	-3.66	Putative enzymes		
		PA3013	faoB	fatty-acid oxidation complex beta-subunit	-3.05	Fatty acid and phospholipid metabolism	Amino acid biosynthesis and metabolism	
		PA3014	faoA	fatty-acid oxidation complex alpha-subunit	-2.02	Fatty acid and phospholipid metabolism	Amino acid biosynthesis and metabolism	
		PA3299	fadD1	long-chain-fatty-acid--CoA ligase	-2.70	Fatty acid and phospholipid metabolism		
		PA4435		probable acyl-CoA dehydrogenase	2.71	Putative enzymes		
		PA4994		probable acyl-CoA dehydrogenase	5.95	Putative enzymes		
		PA5349	rubB	rubredoxin reductase	-2.70	Carbon compound catabolism		
		PA5350	rubA2	Rubredoxin 2	-2.16	Carbon compound catabolism		
	Fatty acid biosynthesis	PA1609	fabB	beta-ketoacyl-ACP synthase I	-5.41	Fatty acid and phospholipid metabolism		
		PA1806	fabI	NADH-dependent enoyl-ACP reductase	-7.81	Fatty acid and phospholipid metabolism		
		PA2949		esterase	-3.07	Fatty acid and phospholipid metabolism	Putative enzymes	
		PA2965	fabF1	beta-ketoacyl-acyl carrier protein synthase II	-2.03	Fatty acid and phospholipid metabolism		
		PA3092	fadH1	2,4-dienoyl-CoA reductase FadH1	6.35	Fatty acid and phospholipid metabolism		
		PA3112	accD	acetyl-CoA carboxylase beta subunit	2.12	Fatty acid and phospholipid metabolism		
		PA3333	fabH2	3-oxoacyl-[acyl-carrier-protein] synthase III	-8.40	Fatty acid and phospholipid metabolism		
		PA3924		probable medium-chain acyl-CoA ligase	-4.48	Fatty acid and phospholipid metabolism		
		PA4339		probable phospholipase	2.22	Fatty acid and phospholipid metabolism		
		PA4814	fadH2	2,4-dienoyl-CoA reductase FadH2	13.21	Fatty acid and phospholipid metabolism		
	Glycerophospholipid metabolism	PA0005	lptA	lysophosphatidic acid acyltransferase, LptA	3.67	Fatty acid and phospholipid metabolism		
		PA0581		conserved hypothetical protein	2.32	Hypothetical, unclassified, unknown		
		PA0798	pmtA	phospholipid methyltransferase	-2.47	Fatty acid and phospholipid metabolism		
		PA1614	gpsA	glycerol-3-phosphate dehydrogenase, biosynthetic	2.75	Fatty acid and phospholipid metabolism	Central intermediary metabolism	
		PA2536		probable phosphatidate cytidylyltransferase	-4.85	Fatty acid and phospholipid metabolism		
		PA2537		probable acyltransferase	-3.94	Fatty acid and phospholipid metabolism		
		PA2541		probable CDP-alcohol phosphatidyltransferase	2.48	Putative enzymes		
		PA2990		probable phosphodiesterase	-5.03	Central intermediary metabolism		
		PA3603	dgkA	diacylglycerol kinase	2.40	Fatty acid and phospholipid metabolism		
		PA3857	pcs	phosphatidylcholine synthase	-2.64	Fatty acid and phospholipid metabolism		
		PA4024	eutB	ethanolamine-ammonia lyase, large subunit, EutB	-5.62	Central intermediary metabolism		
		PA4025	eutC	ethanolamine-ammonia lyase, small subunit, EutC	-5.41	Carbon compound catabolism		
		PA4350	olsB	OlsB	5.87	Hypothetical, unclassified, unknown		
		PA4351	olsA	OlsA	10.42	Fatty acid and phospholipid metabolism		
		PA4693	pssA	phosphatidylserine synthase	-3.14	Fatty acid and phospholipid metabolism		
		PA4957	psd	phosphatidylserine decarboxylase	-3.97	Fatty acid and phospholipid metabolism	Amino acid biosynthesis and metabolism	
		PA5310		conserved hypothetical protein	-2.79	Hypothetical, unclassified, unknown		
		PA5394	cls	cardiolipin synthase	2.08	Fatty acid and phospholipid metabolism		
	Sphingolipid metabolism	PA0845	cerN	CerN	3.60	Hypothetical, unclassified, unknown		
	Fatty acid desaturase	PA0286	desA	delta-9 fatty acid desaturase, DesA	-2.88	Fatty acid and phospholipid metabolism		
		PA4889		probable oxidoreductase	2.38	Putative enzymes		
		PA5102		hypothetical protein	3.55	Membrane proteins		
	Acyl-carrier protein	PA1869	acp1	Acp1	-6.45	Fatty acid and phospholipid metabolism		
		PA3334	acp3	Acp3	-6.17	Fatty acid and phospholipid metabolism		
	Long-chain fatty acid transport	PA1288		probable outer membrane protein precursor	-2.34	Membrane proteins	Transport of small molecules	
	Short fatty acid chain transport	PA2002		conserved hypothetical protein	-2.13	Membrane proteins		
	Lipids transport	PA5107	blc	outer membrane lipoprotein Blc	6.97	Membrane proteins		
	Ethanolamine transport	PA1491		probable transporter	4.12	Membrane proteins	Transport of small molecules	
		PA4023	eat	ethanolamine transporter, Eat	2.10	Transport of small molecules		
	Lipoprotein biosynthesis and transport	PA0341	lgt	prolipoprotein diacylglyceryl transferase	-3.86	Fatty acid and phospholipid metabolism	Translation, post-translational modification, degradation	
		PA2988	lolE	lipoprotein localization protein LolE	14.72	Membrane proteins		
		PA3984	lnt	apolipoprotein N-acyltransferase	-2.25	Translation, post-translational modification, degradation	Cell Wall / LPS / Capsule	
		PA4668	lolB	lipoprotein localization protein LolB	-6.80	Cell Wall / LPS / Capsule		
	Unknow function	PA3387	rhlG	beta-ketoacyl reductase	33.58	Fatty acid and phospholipid metabolism	Cell Wall / LPS / Capsule	
Prosthetic group biosynthesis	Heme biosynthesis	PA0023	qor	quinone oxidoreductase	-5.85	Energy metabolism		
		PA1546	hemN	oxygen-independent coproporphyrinogen III oxidase	-17.86	Biosynthesis of cofactors, prosthetic groups and carrier		
		PA3977	hemL	glutamate-1-semialdehyde 2,1-aminomutase	-6.76	Biosynthesis of cofactors, prosthetic groups and carrier		
		PA4655	hemH	ferrochelatase	2.17	Biosynthesis of cofactors, prosthetic groups and carrier		
		PA5243	hemB	delta-aminolevulinic acid dehydratase	-3.02	Biosynthesis of cofactors, prosthetic groups and carrier		
		PA5259	hemD	uroporphyrinogen-III synthetase	4.29	Biosynthesis of cofactors, prosthetic groups and carrier		
		PA5260	hemC	porphobilinogen deaminase	5.30	Biosynthesis of cofactors, prosthetic groups and carrier		
		PA0672	hemO	heme oxygenase	-2.18	Biosynthesis of cofactors, prosthetic groups and carrier		
	Heme transporter	PA1475	ccmA	heme exporter protein CcmA	4.99	Transport of small molecules		
		PA1476	ccmB	heme exporter protein CcmB	3.25	Membrane proteins	Transport of small molecules	
	Pyrroloquinoline quinone biosynthesis	PA1985	pqqA	pyrroloquinoline quinone biosynthesis protein A	-16.39	Biosynthesis of cofactors, prosthetic groups and carrier		
		PA1986	pqqB	pyrroloquinoline quinone biosynthesis protein B	-6.25	Biosynthesis of cofactors, prosthetic groups and carrier		
		PA1987	pqqC	pyrroloquinoline quinone biosynthesis protein C	-5.49	Biosynthesis of cofactors, prosthetic groups and carrier		
		PA1988	pqqD	pyrroloquinoline quinone biosynthesis protein D	-3.51	Biosynthesis of cofactors, prosthetic groups and carrier		
		PA1989	pqqE	pyrroloquinoline quinone biosynthesis protein E	-6.33	Biosynthesis of cofactors, prosthetic groups and carrier		
		PA1990	pqqH	PqqH	-8.33	Putative enzymes		
	Vitamin B12 coenzyme biosynthesis	PA1923		hypothetical protein	4.08	Biosynthesis of cofactors, prosthetic groups and carrier		
		PA2944	cobN	cobalamin biosynthetic protein CobN	3.61	Biosynthesis of cofactors, prosthetic groups and carrier		
		PA2945		conserved hypothetical protein	2.05	Biosynthesis of cofactors, prosthetic groups and carrier		
		PA2948	cobM	precorrin-3 methylase	-2.32	Biosynthesis of cofactors, prosthetic groups and carrier		
	Nitric oxide protection	PA2664	fhp	flavohemoprotein	2.22	Energy metabolism		
		PA2665	fhpR	Transcriptional activator of P. aeruginosa flavohemoglobin, FhpR	3.59	Adaptation, Protection	Transcriptional regulators	
	Sphingosine detection and catabolism	PA5324	sphR	Sphingosine-responsive Regulator, SphR	4.86	Adaptation, Protection	Transcriptional regulators	
		PA5325	sphA	SphA	5.93	Adaptation, Protection		
		PA5326	sphD	SphD	2.36	Hypothetical, unclassified, unknown		
		PA5327	sphC	SphC	30.77	Putative enzymes		
		PA5328	sphB	SphB	44.12	Energy metabolism		
	Vitamin B6 metabolism	PA0593	pdxA	pyridoxal phosphate biosynthetic protein PdxA	-7.19	Biosynthesis of cofactors, prosthetic groups and carrier		
		PA0773	pdxJ	pyridoxal phosphate biosynthetic protein PdxJ	-3.09	Biosynthesis of cofactors, prosthetic groups and carrier		
		PA1049	pdxH	pyridoxine 5'-phosphate oxidase	-4.48	Biosynthesis of cofactors, prosthetic groups and carrier		
	Nicotinate and nicotinamide metabolism	PA0196	pntB	pyridine nucleotide transhydrogenase, beta subunit	-2.59	Transport of small molecules	Energy metabolism	
		PA1004	nadA	quinolinate synthetase A	3.24	Amino acid biosynthesis and metabolism	Biosynthesis of cofactors, prosthetic groups and carrier	
		PA1125		probable cobalamin biosynthetic protein	-2.45	Biosynthesis of cofactors, prosthetic groups and carrier		
		PA1823	nudC	NudC	-2.92	Hypothetical, unclassified, unknown		
		PA1197		hypothetical protein	-2.21	Hypothetical, unclassified, unknown		
		PA4376	pncB2	nicotinate phosphoribosyltransferase	-2.72	Biosynthesis of cofactors, prosthetic groups and carrier		
		PA4918	pcnA	nicotinamidase, PcnA	55.24	Hypothetical, unclassified, unknown		
		PA4919	pncB1	nicotinate phosphoribosyltransferase	26.99	Biosynthesis of cofactors, prosthetic groups and carrier		
		PA4920	nadE	NH3-dependent NAD synthetase	3.00	Amino acid biosynthesis and metabolism	Biosynthesis of cofactors, prosthetic groups and carrier	
	Biotine metabolism	PA0420	bioA	adenosylmethionine-8-amino-7-oxononanoate aminotransferase	-2.05	Biosynthesis of cofactors, prosthetic groups and carrier		
		PA0501	bioF	8-amino-7-oxononanoate synthase	3.50	Biosynthesis of cofactors, prosthetic groups and carrier		
		PA1017	pauA	pimeloyl-CoA synthetase	8.73	Biosynthesis of cofactors, prosthetic groups and carrier		
		PA4280	birA	BirA bifunctional protein	3.26	Biosynthesis of cofactors, prosthetic groups and carrier		
	Folate biosynthesis	PA0582	folB	dihydroneopterin aldolase	5.32	Biosynthesis of cofactors, prosthetic groups and carrier		
		PA0583		hypothetical protein	5.30	Biosynthesis of cofactors, prosthetic groups and carrier		
		PA3028	moeA2	molybdenum cofactor biosynthesis protein A2	-3.83	Biosynthesis of cofactors, prosthetic groups and carrier		
		PA3029	moaB2	molybdopterin biosynthetic protein B2	-3.94	Biosynthesis of cofactors, prosthetic groups and carrier		
		PA3437	folM	dihydromonapterin reductase, FolM	-2.02	Biosynthesis of cofactors, prosthetic groups and carrier	Putative enzymes	
		PA3438	folE1	GTP cyclohydrolase I precursor	-3.79	Biosynthesis of cofactors, prosthetic groups and carrier		
		PA3439	folX	d-erythro-7,8-dihydroneopterin triphosphate epimerase	-3.56	Biosynthesis of cofactors, prosthetic groups and carrier		
		PA3870	moaA1	molybdopterin biosynthetic protein A1	2.31	Biosynthesis of cofactors, prosthetic groups and carrier		
		PA3917	moaD	molybdopterin converting factor, small subunit	-2.17	Biosynthesis of cofactors, prosthetic groups and carrier		
		PA4663	moeB	molybdopterin biosynthesis MoeB protein	-2.99	Biosynthesis of cofactors, prosthetic groups and carrier		
	Thiamine biosynthesis	PA3976	thiE	thiamin-phosphate pyrophosphorylase	-2.34	Biosynthesis of cofactors, prosthetic groups and carrier		
		PA5118	thiI	thiazole biosynthesis protein ThiI	6.19	Biosynthesis of cofactors, prosthetic groups and carrier		
	Riboflavin metabolism	PA4047	ribA	GTP cyclohydrolase II	2.73	Biosynthesis of cofactors, prosthetic groups and carrier		
		PA4054	ribB	GTP cyclohydrolase II / 3,4-dihydroxy-2-butanone 4-phosphate synthase	-2.71	Biosynthesis of cofactors, prosthetic groups and carrier		
	Pantothenate biosynthesis	PA4397	panE	ketopantoate reductase	-2.27	Biosynthesis of cofactors, prosthetic groups and carrier	Nucleotide biosynthesis and metabolism	
		PA4529	coaE	dephosphocoenzyme A kinase	-2.70	Biosynthesis of cofactors, prosthetic groups and carrier		
		PA4729	panB	3-methyl-2-oxobutanoate hydroxymethyltransferase	-2.51	Biosynthesis of cofactors, prosthetic groups and carrier		
		PA4730	panC	pantoate--beta-alanine ligase	-3.68	Biosynthesis of cofactors, prosthetic groups and carrier		
		PA5320	coaC	Phosphopantothenoylcysteine synthase/(R)-4'-phospho-N-pantothenoylcysteine decarboxylase	-3.64	DNA replication, recombination, modification and repair	Biosynthesis of cofactors, prosthetic groups and carrier	
	Methyltransferase	PA1772		probable methyltransferase	-3.53	Biosynthesis of cofactors, prosthetic groups and carrier		
		PA4664	prmC	S-adenosylmethionine-dependent methyltransferase, PrmC	-2.26	Biosynthesis of cofactors, prosthetic groups and carrier		
	Lipoic acid metabolism	PA3997	lipB	lipoate-protein ligase B	-2.69	Amino acid biosynthesis and metabolism	Biosynthesis of cofactors, prosthetic groups and carrier	
		PA0936	lpxO2	lipopolysaccharide biosynthetic protein LpxO2	-2.15	Cell Wall / LPS / Capsule	Putative enzymes	

LPS	LPS biosynthesis	PA3141	*wbpM*	nucleotide sugar epimerase/dehydratase WbpM	2.72	Membrane proteins	Cell Wall / LPS / Capsule	Putative enzymes
		PA3145	*wbpL*	glycosyltransferase WbpL	-2.00	Cell Wall / LPS / Capsule	Membrane proteins	
		PA3147	*wbpJ*	probable glycosyl transferase WbpJ	-2.11	Cell Wall / LPS / Capsule	Putative enzymes	
		PA3148	*wbpI*	UDP-N-acetylglucosamine 2-epimerase WbpI	-7.69	Cell Wall / LPS / Capsule	Putative enzymes	
		PA3149	*wbpH*	probable glycosyltransferase WbpH	-10.87	Cell Wall / LPS / Capsule	Putative enzymes	
		PA3150	*wbpG*	LPS biosynthesis protein WbpG	-12.50	Cell Wall / LPS / Capsule		
		PA3153	*wzx*	O-antigen translocase	-6.25	Cell Wall / LPS / Capsule	Membrane proteins	Transport of small molecules
		PA3154	*wzy*	B-band O-antigen polymerase	-7.63	Cell Wall / LPS / Capsule	Membrane proteins	
		PA3155	*wbpE*	UDP-2-acetamido-2-dideoxy-d-ribo-hex-3-uluronic acid transaminase, wbpE	-7.63	Cell Wall / LPS / Capsule	Putative enzymes	
		PA3156	*wbpD*	UDP-2-acetamido-3-amino-2,3-dideoxy-d-glucuronic acid N-acetyltransferase, WbpD	-4.44	Cell Wall / LPS / Capsule	Putative enzymes	
		PA3157	*wbpC*	probable acetyltransferase	-2.36	Cell Wall / LPS / Capsule	Putative enzymes	
		PA3159	*wbpA*	UDP-N-acetyl-d-glucosamine 6-Dehydrogenase	2.42	Cell Wall / LPS / Capsule	Putative enzymes	
		PA3160	*wzz*	O-antigen chain length regulator	7.71	Cell Wall / LPS / Capsule		
		PA3242	*htrB2*	temperature-regulated acyltransferase HtrB1	4.22	Cell Wall / LPS / Capsule		
		PA4425	*gmhA*	sedoheptulose 7-phosphate isomerase GmhA	-4.67	Putative enzymes		
		PA4457	*kdsD*	arabinose-5-phosphate isomerase KdsD	3.13	Secreted Factors (toxins, enzymes, alginate)		
		PA4661	*pagL*	Lipid A 3-O-deacylase	-3.45	Hypothetical, unclassified, unknown		
		PA4999	*waaL*	O-antigen ligase, WaaL	-2.14	Membrane proteins		
		PA5001	*ssg*	cell surface-sugar biosynthetic glycosyltransferase, Ssg	-2.44	Hypothetical, unclassified, unknown		
		PA5007	*wapG*	WapG	-2.96	Putative enzymes		
		PA5008	*wapP*	WapP	-3.06	Putative enzymes		
		PA5009	*waaP*	lipopolysaccharide kinase WaaP	-2.32	Cell Wall / LPS / Capsule		
		PA5161	*rmlB*	dTDP-D-glucose 4,6-dehydratase	-2.35	Cell Wall / LPS / Capsule		
		PA5162	*rmlD*	dTDP-4-dehydrorhamnose reductase	-5.13	Cell Wall / LPS / Capsule		
		PA5163	*rmlA*	glucose-1-phosphate thymidylyltransferase	-9.35	Cell Wall / LPS / Capsule		
		PA5164	*rmlC*	dTDP-4-dehydrorhamnose 3,5-epimerase	-12.82	Cell Wall / LPS / Capsule		
		PA5238		probable O-antigen acetylase	3.81	Membrane proteins	Cell Wall / LPS / Capsule	Putative enzymes
		PA5447	*wbpZ*	glycosyltransferase WbpZ	-2.50	Cell Wall / LPS / Capsule		
		PA5448	*wbpY*	glycosyltransferase WbpY	-3.46	Cell Wall / LPS / Capsule		
		PA5449	*wbpX*	glycosyltransferase WbpX	-3.39	Cell Wall / LPS / Capsule		
		PA5450	*wzt*	ABC subunit of A-band LPS efflux transporter	-2.77	Cell Wall / LPS / Capsule	Transport of small molecules	
		PA5452	*wbpW*	phosphomannose isomerase/GDP-mannose WbpW	-2.02	Cell Wall / LPS / Capsule		
		PA5453	*gmd*	GDP-mannose 4,6-dehydratase	-2.56	Cell Wall / LPS / Capsule		
		PA5455		putative glycosyltransferase	-2.42	Cell Wall / LPS / Capsule		
		PA5456		putative glycosyltransferase	-6.33	Cell Wall / LPS / Capsule		
		PA5457		methyltransferase	-4.81	Cell Wall / LPS / Capsule		
		PA5458		putative acetyltransferase	-2.90	Cell Wall / LPS / Capsule	Membrane proteins	
		PA5459		putative methyltransferase	-9.43	Cell Wall / LPS / Capsule		
	LPS export	PA0595	*lptD*	LPS-assembly protein LptD	-3.36	Adaptation, Protection		
		PA3828	*lptF*	Lipopolysaccharide export system permease protein LptF	5.47	Membrane proteins		
		PA3988	*lptE*	LPS-assembly lipoprotein LptE	-2.14	Hypothetical, unclassified, unknown		
		PA4461	*lptB*	Lipopolysaccharide export system ATP-binding protein LptB	-2.30	Transport of small molecules		
	LPS regulation	PA4378	*warB*	WarB	25.88	Adaptation, Protection		
		PA4379	*warA*	WarA	31.00	Hypothetical, unclassified, unknown		
Peptidoglycan	Peptidoglycan biosynthesis	PA0378		probable transglycosylase	3.82	Cell Wall / LPS / Capsule		
		PA1812	*mltD*	membrane-bound lytic murein transglycosylase D precursor	-6.76	Amino acid biosynthesis and metabolism	Cell Wall / LPS / Capsule	
		PA3020		probable soluble lytic transglycosylase	-4.88	Cell Wall / LPS / Capsule		
		PA3047		probable D-alanyl-D-alanine carboxypeptidase	-3.75	Cell Wall / LPS / Capsule		
		PA3636	*kdsA*	2-dehydro-3-deoxyphosphooctonate aldolase	-5.81	Cell Wall / LPS / Capsule	Carbon compound catabolism	
		PA3644	*lpxA*	UDP-N-acetylglucosamine acyltransferase	-2.87	Cell Wall / LPS / Capsule		
		PA3645	*fabZ*	(3R)-hydroxymyristoyl-[acyl carrier protein] dehydratase	-2.70	Cell Wall / LPS / Capsule	Fatty acid and phospholipid metabolism	
		PA3646	*lpxD*	UDP-3-O-[3-hydroxylauroyl] glucosamine N-acyltransferase	-5.56	Cell Wall / LPS / Capsule		
		PA3999	*dacC*	D-ala-D-ala-carboxypeptidase	-3.00	Cell Wall / LPS / Capsule		
		PA4001	*sltB1*	soluble lytic transglycosylase B	2.25	Cell Wall / LPS / Capsule		
		PA4003	*pbpA*	penicillin-binding protein 2	2.98	Cell Wall / LPS / Capsule		
		PA4201	*ddlA*	D-alanine-D-alanine ligase A	-3.05	Cell Wall / LPS / Capsule		
		PA4406	*lpxC*	UDP-3-O-acyl-N-acetylglucosamine deacetylase	-2.10	Cell Wall / LPS / Capsule		
		PA4410	*ddlB*	D-alanine--D-alanine ligase	-6.71	Cell Wall / LPS / Capsule		
		PA4411	*murC*	UDP-N-acetylmuramate--alanine ligase	-6.33	Cell Wall / LPS / Capsule		
		PA4412	*murG*	UDP-N-acetylglucosamine--N-acetylmuramyl-(pentapeptide) pyrophosphoryl-undecaprenol N-acetylglucosamine transferase	-6.33	Cell Wall / LPS / Capsule	Carbon compound catabolism	
		PA4414	*murD*	UDP-N-acetylmuramoylalanine--D-glutamate ligase	-6.71	Cell Wall / LPS / Capsule		
		PA4415	*mraY*	phospho-N-acetylmuramoyl-pentapeptide-transferase	-2.07	Cell Wall / LPS / Capsule		
		PA4416	*murF*	UDP-N-acetylmuramoylalanyl-D-glutamyl-2, 6-diaminopimelate--D-alanyl-D-alanyl ligase	-2.60	Cell Wall / LPS / Capsule		
		PA4417	*murE*	UDP-N-acetylmuramoylalanyl-D-glutamate-2, 6-diaminopimelate ligase	-2.23	Cell Wall / LPS / Capsule		
		PA4444	*mltB1*	soluble and membrane-bound lytic transglycosylases	-5.62	Cell Wall / LPS / Capsule		
		PA4662	*murI*	glutamate racemase	-2.03	Cell Wall / LPS / Capsule		
		PA4700	*mrcB*	penicillin-binding protein 1B	8.71	Cell Wall / LPS / Capsule		
		PA5538	*amiA*	N-acetylmuramoyl-L-alanine amidase	30.15	Cell Wall / LPS / Capsule		
		PA5552	*glmU*	glucosamine-1-phosphate acetyltransferase/N-acetylglucosamine-1-phosphate uridyltransferase	-2.54	Cell Wall / LPS / Capsule		
Adaptation, protection, chemotaxis	Mechanosensitive channels	PA1775	*cmpX*	conserved cytoplasmic membrane protein, CmpX protein	9.91	Membrane proteins		
		PA4394		conserved hypothetical protein	2.12	Hypothetical, unclassified, unknown		
		PA4614	*mscL*	conductance mechanosensitive channel	-2.56	Adaptation, Protection	Transport of small molecules	Membrane proteins
		PA4925		conserved hypothetical protein	-11.63	Hypothetical, unclassified, unknown		
		PA5022		conserved hypothetical protein	-3.02	Hypothetical, unclassified, unknown		
		PA5121		hypothetical protein	4.03	Membrane proteins		
	Glycine-Betaine catabolism	PA5411	*gbcB*	GbcB	2.86	Amino acid biosynthesis and metabolism		
	Osmoprotectant transport	PA3236	*betX*	BetX	4.70	Adaptation, Protection	Transport of small molecules	
		PA3888	*opuCD*	OpuC ABC transporter, permease protein, OpuCD	2.82	Transport of small molecules	Membrane proteins	
		PA3891	*opuCA*	OpuC ABC transporter, ATP-binding protein, OpuCA	-2.68	Transport of small molecules		
		PA3933	*betT3*	BetT3	-5.88	Membrane proteins	Transport of small molecules	
		PA5291	*betT2*	BetT2	-4.15	Membrane proteins	Transport of small molecules	
		PA5375	*betT1*	BetT1	4.03	Transport of small molecules	Membrane proteins	
		PA5376	*cbcV*	CbcV	-4.65	Transport of small molecules		
		PA5377	*cbcW*	CbcW	-3.53	Transport of small molecules	Membrane proteins	
	Oxygen protection	PA0059	*osmC*	osmotically inducible protein OsmC	-2.62	Adaptation, Protection		
		PA0140	*ahpF*	alkyl hydroperoxide reductase subunit F	4.39	Adaptation, Protection		
		PA1204		NAD(P)H quinone oxidoreductase	-3.85	Hypothetical, unclassified, unknown		
		PA2185	*katN*	non-heme catalase KatN	3.24	Adaptation, Protection		
		PA2850	*ohr*	organic hydroperoxide resistance protein	5.61	Adaptation, Protection		
		PA3529		alkylhydroperoxide reductase C	-2.38	Adaptation, Protection	Putative enzymes	
		PA3815	*iscR*	IscR	5.49	Adaptation, Protection		
		PA4236	*katA*	catalase	-5.62	Adaptation, Protection		
		PA4366	*sodB*	superoxide dismutase	-4.69	Adaptation, Protection		
		PA4468	*sodM*	superoxide dismutase	-22.73	Adaptation, Protection		
		PA4615	*fprB*	FprB	-2.58	Putative enzymes		
		PA4876	*osmE*	osmotically inducible lipoprotein OsmE	-2.00	Membrane proteins	Adaptation, Protection	
		PA4975		NAD(P)H quinone oxidoreductase	-3.48	Energy metabolism		
		PA5344	*oxyR*	OxyR	-3.80	Transcriptional regulators		
	HCN protection	PA4129		hypothetical protein	-5.56	Adaptation, Protection		
		PA4130		probable sulfite or nitrite reductase	-3.12	Adaptation, Protection	Central intermediary metabolism	
		PA4131		probable iron-sulfur protein	-3.37	Adaptation, Protection	Putative enzymes	
		PA4132		conserved hypothetical protein	-5.78	Adaptation, Protection		
		PA4134		hypothetical protein	-7.81	Adaptation, Protection		
	Peroxyde reduction	PA2532	*tpx*	thiol peroxidase	-3.73	Adaptation, Protection		
		PA2616	*trxB1*	thioredoxin reductase 1	-2.91	Nucleotide biosynthesis and metabolism		
		PA5240	*trxA*	thioredoxin	-3.26	Nucleotide biosynthesis and metabolism	Energy metabolism	nslation, post-translational modification, degradat
	Glutathione metabolism	PA1287		probable glutathione peroxidase	-2.95	Putative enzymes		
		PA2821		probable glutathione S-transferase	-2.76	Central intermediary metabolism		
		PA2826		probable glutathione peroxidase	2.00	Adaptation, Protection		
	Lysozyme protection	PA0867	*mliC*	membrane-bound lysozyme inhibitor of c-type lysozyme MliC	-5.10	Adaptation, Protection		
	DNA protection protein	PA0021		conserved hypothetical protein	3.97	Membrane proteins		
	Extracellular DNA release	PA4101	*bfmR*	BfmR	-4.65	Two-component regulatory systems	Transcriptional regulators	
		PA4102	*bfmS*	BfmS	-5.21	Two-component regulatory systems	Cell Wall / LPS / Capsule	Adaptation, Protection
	Lysis protection	PA0691	*phdA*	prevent-host-death protein A	9.09	Hypothetical, unclassified, unknown		
	Extracellular proteins processing	PA2782	*bamI*	biofilm-associated metzincin Inhibitor, BamI	3.86	Hypothetical, unclassified, unknown		
		PA2783	*mep72*	Mep72	3.19	Hypothetical, unclassified, unknown		
	Arsenate resistance	PA0950		probable arsenate reductase	2.88	Adaptation, Protection	Transport of small molecules	
		PA2277	*arsR*	ArsR protein	5.25	Transcriptional regulators		
		PA2278	*arsB*	ArsB protein	5.64	Membrane proteins	Transport of small molecules	
		PA2280		oxidoreductase	-5.95	Hypothetical, unclassified, unknown		
	Oxidoreductases	PA1127		probable oxidoreductase	-3.48	Adaptation, Protection	Putative enzymes	
	Cold-shock proteins	PA0961		probable cold-shock protein	-2.11	Transcriptional regulators		
		PA1159		probable cold-shock protein	-4.44	Adaptation, Protection	Transcriptional regulators	
		PA2622	*cspD*	cold-shock protein CspD	-10.99	Adaptation, Protection	Transcriptional regulators	
		PA3266	*capB*	cold acclimation protein B	3.63	Adaptation, Protection	Transcriptional regulators	
	Heat-shock proteins	PA0376	*rpoH*	sigma factor RpoH	4.03	Transcriptional regulators		
		PA1068		probable heat shock protein (hsp90 family)	2.63	Chaperones & heat shock proteins		
		PA3126	*ibpA*	heat-shock protein IbpA	2.76	Chaperones & heat shock proteins		
		PA4762	*grpE*	heat shock protein GrpE	2.10	DNA replication, recombination, modification and repair	Chaperones & heat shock proteins	
		PA4873		probable heat-shock protein	10.26	Chaperones & heat shock proteins		
		PA5195		probable heat shock protein	2.11	Chaperones & heat shock proteins		
	Copper resistance	PA2064	*pcoB*	copper resistance protein B precursor	-2.16	Adaptation, Protection		
		PA2065	*pcoA*	copper resistance protein A precursor	-3.46	Adaptation, Protection		
	Amino acid starvation response	PA4427	*sspB*	stringent starvation protein B	-3.47	Adaptation, Protection		
		PA4428	*sspA*	stringent starvation protein A	-3.58	Adaptation, Protection		
	Persistance regulation	PA4566	*obg*	GTP-binding protein Obg	12.48	Adaptation, Protection		
	Zinc limitation response	PA5536	*dksA2*	DksA2	26.94	Adaptation, Protection	Transcriptional regulators	
	Formaldehyde resistance	PA3630	*gfnR*	glutathione-dependent formaldehyde neutralization regulator GfnR	3.65	Transcriptional regulators		
	Photosensing	PA4117	*bphP*	bacterial phytochrome, BphP	-5.03	Two-component regulatory systems		
	Chaperones	PA4385	*groEL*	GroEL protein	-2.21	Chaperones & heat shock proteins		
		PA4386	*groES*	GroES protein	-2.89	Chaperones & heat shock proteins		
	High calcium concentration response	PA4107	*efhP*	EfhP	10.83	Hypothetical, unclassified, unknown		
	Cell redox homeostasis	PA2477		probable thiol:disulfide interchange protein	2.63	Membrane proteins	Chaperones & heat shock proteins	Putative enzymes
		PA2478		probable thiol:disulfide interchange protein	2.48	Membrane proteins	Chaperones & heat shock proteins	Putative enzymes
	Envelope stress response	PA1776	*sigX*	ECF sigma factor SigX	2.33	Transcriptional regulators		
		PA2895	*sbrR*	SbrR	2.90	Transcriptional regulators		
		PA2896	*sbrI*	SbrI	5.00	Transcriptional regulators		
	Stress-related proteins	PA1801	*clpP*	ClpP	-2.98	Cell Wall / LPS / Capsule	Antibiotic resistance and susceptibility	Motility and Attachment
		PA1802	*clpX*	ClpX	-4.20	Cell Wall / LPS / Capsule	Chaperones & heat shock proteins	
	SOS Response	PA1803	*lon*	Lon protease	-3.53	Translation, post-translational modification, degradation	Adaptation, Protection	
		PA3007	*lexA*	repressor protein LexA	7.46	Translation, post-translational modification, degradation	Adaptation, Protection	
		PA3008	*sulA*	SulA	6.71	Hypothetical, unclassified, unknown		
		PA3617	*recA*	RecA protein	5.32	DNA replication, recombination, modification and repair		
		PA4234	*uvrA*	excinuclease ABC subunit A	-2.47	DNA replication, recombination, modification and repair		
	Programmed cell death	PA0907	*alpA*	lysis phenotype activator, AlpA	8.00	Hypothetical, unclassified, unknown		
		PA0908	*alpB*	AlpB	3.18	Antibiotic resistance and susceptibility		
	Toxin/antitoxin systems	PA0124		ParE toxin	7.08	Hypothetical, unclassified, unknown		
		PA0125		ParD antitoxin	6.77	Hypothetical, unclassified, unknown		
	Chemotaxis	PA0173		probable methylesterase	-33.33	Adaptation, Protection	Chemotaxis	
		PA0174		conserved hypothetical protein	-19.23	Hypothetical, unclassified, unknown		
		PA0175	*cheR2*	CheR2	-43.48	Adaptation, Protection	Transcriptional regulators	Chemotaxis
		PA0176	*aer2*	aerotaxis transducer Aer2	-38.46	Adaptation, Protection	Chemotaxis	
		PA0177		probable purine-binding chemotaxis protein	-21.28	Adaptation, Protection	Chemotaxis	
		PA0178		probable two-component sensor	-10.31	Two-component regulatory systems	Adaptation, Protection	Chemotaxis
		PA0179		probable two-component response regulator	-6.41	Two-component regulatory systems	Adaptation, Protection	Chemotaxis
		PA1251		probable chemotaxis transducer	2.02	Adaptation, Protection	Chemotaxis	
		PA1423	*bdlA*	BdlA	2.84	Cell Wall / LPS / Capsule	Chemotaxis	
		PA1463		hypothetical protein	3.77	Hypothetical, unclassified, unknown		
		PA1608		probable chemotaxis transducer	8.27	Adaptation, Protection	Chemotaxis	
		PA1646		probable chemotaxis transducer	3.98	Adaptation, Protection	Chemotaxis	
		PA1930		probable chemotaxis transducer	-14.49	Adaptation, Protection	Chemotaxis	
		PA2561	*ctpH*	CtpH	10.68	Adaptation, Protection	Chemotaxis	
		PA2573		probable chemotaxis transducer	-4.15	Adaptation, Protection	Chemotaxis	
		PA2652		methyl-accepting chemotaxis protein	2.17	Adaptation, Protection	Chemotaxis	
		PA2654	*tlpQ*	TlpQ	7.24	Adaptation, Protection	Chemotaxis	
		PA2788		probable chemotaxis transducer	-2.08	Adaptation, Protection	Chemotaxis	
		PA2867		probable chemotaxis transducer	2.01	Adaptation, Protection	Chemotaxis	
		PA2920		probable chemotaxis transducer	3.07	Adaptation, Protection	Chemotaxis	
		PA3348	*cheR1*	CheR1	3.66	Adaptation, Protection	Chemotaxis	
		PA3703	*wspF*	probable methylesterase	-2.74	Motility and Attachment	Chemotaxis	Transcriptional regulators
		PA3704	*wspE*	probable chemotaxis sensor/effector fusion protein	-3.39	Two-component regulatory systems	Chemotaxis	Motility and Attachment
		PA4290		probable chemotaxis transducer	11.15	Adaptation, Protection	Chemotaxis	
		PA4520		probable chemotaxis transducer	22.29	Adaptation, Protection	Chemotaxis	
		PA4608	*mapZ*	MapZ	-2.71	Hypothetical, unclassified, unknown		
		PA4633		probable chemotaxis transducer	2.44	Adaptation, Protection	Chemotaxis	
		PA4844	*ctpL*	CtpL	5.21	Adaptation, Protection	Chemotaxis	
		PA4915		probable chemotaxis transducer	-4.63	Adaptation, Protection	Chemotaxis	
		PA5072	*mcpK*	McpK	27.03	Adaptation, Protection	Chemotaxis	
	Others	PA1782		probable serine/threonine-protein kinase	4.78	Adaptation, Protection		
		PA3109		hypothetical protein	4.03	Adaptation, Protection		
		PA3327		probable non-ribosomal peptide synthetase	-4.13	Adaptation, Protection		
		PA4078		probable nonribosomal peptide synthetase	-19.23	Adaptation, Protection		
Replication, DNA processing	DNA polymerase	PA0002	*dnaN*	DNA polymerase III, beta chain	-2.04	DNA replication, recombination, modification and repair		
		PA0923	*dinB*	DNA Polymerase IV, DinB	12.74	DNA replication, recombination, modification and repair	Adaptation, Protection	
		PA1532	*dnaX*	DNA polymerase subunits gamma and tau	2.57	DNA replication, recombination, modification and repair		
		PA1886	*polB*	DNA polymerase II	2.04	DNA replication, recombination, modification and repair		
		PA3832	*holC*	DNA polymerase III, chi subunit	-2.67	DNA replication, recombination, modification and repair		
		PA3989	*holA*	DNA polymerase III, delta subunit	-4.12	DNA replication, recombination, modification and repair		
	DNA topoisomerase	PA3011	*topA*	DNA topoisomerase I	-3.05	DNA replication, recombination, modification and repair		
		PA4964	*parC*	topoisomerase IV subunit A	-3.38	DNA replication, recombination, modification and repair		
		PA4967	*parE*	topoisomerase IV subunit B	-3.21	DNA replication, recombination, modification and repair		
	Primase	PA0577	*dnaG*	DNA primase	14.04	DNA replication, recombination, modification and repair		
	Helicase	PA0967	*ruvB*	Holliday junction DNA helicase RuvB	3.68	DNA replication, recombination, modification and repair		
		PA1405		probable helicase	4.37	DNA replication, recombination, modification and repair		
		PA3344	*recQ*	ATP-dependent DNA helicase RecQ	2.49	DNA replication, recombination, modification and repair		
		PA4931	*dnaB*	replicative DNA helicase	3.87	DNA replication, recombination, modification and repair		
		PA5296	*rep*	ATP-dependent DNA helicase Rep	3.20	DNA replication, recombination, modification and repair		
		PA5345	*recG*	ATP-dependent DNA helicase RecG	-5.99	DNA replication, recombination, modification and repair	Transcriptional regulators	
	Nuclease	PA2545	*xthA*	exodeoxyribonuclease III	-2.47	DNA replication, recombination, modification and repair		
		PA2749	*endA*	DNA-specific endonuclease I	5.52	DNA replication, recombination, modification and repair		
		PA3495	*nth*	endonuclease III	-6.02	DNA replication, recombination, modification and repair		
		PA3528	*rnt*	ribonuclease T	-7.25	DNA replication, recombination, modification and repair	Transcription, RNA processing and degradation	
		PA3642	*rnhB*	ribonuclease HII	-2.38	DNA replication, recombination, modification and repair		
		PA4283	*recD*	exodeoxyribonuclease V alpha chain	-2.63	DNA replication, recombination, modification and repair		
		PA4284	*recB*	exodeoxyribonuclease V beta chain	-2.39	DNA replication, recombination, modification and repair		
	DNA repair	PA0382	*micA*	DNA mismatch repair protein MicA	-2.51	DNA replication, recombination, modification and repair		
		PA2118	*ada*	O6-methylguanine-DNA methyltransferase	-2.36	DNA replication, recombination, modification and repair	Transcriptional regulators	
		PA4232	*ssb*	single-stranded DNA-binding protein	-2.00	DNA replication, recombination, modification and repair		
		PA4660	*phr*	deoxyribodipyrimidine photolyase	-2.06	DNA replication, recombination, modification and repair		
		PA4763	*recN*	DNA repair protein RecN	7.31	DNA replication, recombination, modification and repair		
		PA4946	*mutL*	DNA mismatch repair protein MutL	-3.03	DNA replication, recombination, modification and repair		
		PA5147	*mutY*	A / G specific adenine glycosylase	-2.31	DNA replication, recombination, modification and repair		
	Ligase	PA2138	*ligD*	Multifunctional non-homologous end joining protein LigD	3.93	DNA replication, recombination, modification and repair		
	Replication regulation	PA4363	*iciA*	inhibitor of chromosome initiation IciA	2.21	DNA replication, recombination, modification and repair		
		PA4853	*fis*	DNA-binding protein Fis	2.89	DNA replication, recombination, modification and repair	Transcriptional regulators	Transcription, RNA processing and degradation
	Unknown	PA3596		probable methylated-DNA--protein-cysteine methyltransferase	-2.72	DNA replication, recombination, modification and repair		
Transcription, RNA processing	Helicase	PA0428		probable ATP-dependent RNA helicase	-2.17	Transcription, RNA processing and degradation		
		PA2840		probable ATP-dependent RNA helicase	32.64	Transcription, RNA processing and degradation		
		PA3861	*rhl*	ATP-dependent RNA helicase RhlB	-7.52	Transcription, RNA processing and degradation		
	Nuclease	PA0770	*rnc*	ribonuclease III	-2.07	Transcription, RNA processing and degradation		
		PA2976	*rne*	ribonuclease E	-2.79	Transcription, RNA processing and degradation		
		PA4477	*cafA*	cytoplasmic axial filament protein (Rnase G)	2.09	Cell division		
	Transcription elongation	PA2859	*greB*	transcription elongation factor GreB	-2.04	Transcription, RNA processing and degradation		
		PA4745	*nusA*	N utilization substance protein A	2.76	Transcription, RNA processing and degradation		
		PA4755	*greA*	transcription elongation factor GreA	-2.95	Transcription, RNA processing and degradation		
	Ligase	PA2861	*ligT*	2'-5' RNA ligase	3.88	Transcription, RNA processing and degradation		
	Pseudouridine synthase	PA2975	*rluC*	ribosomal large subunit pseudouridine synthase C	4.37	Transcription, RNA processing and degradation		
		PA3246	*rluA*	pseudouridine synthase RluA	2.18	Transcription, RNA processing and degradation		
		PA4544	*rluD*	pseudouridine synthase	2.60	Transcription, RNA processing and degradation		
		PA4742	*truB*	tRNA pseudouridine 55 synthase	3.64	Translation, post-translational modification, degradation	Transcription, RNA processing and degradation	
	rRNA processing	PA0017		conserved hypothetical protein	-3.94	Hypothetical, unclassified, unknown		
		PA1161	*rrmA*	rRNA methyltransferase	-3.47	Transcription, RNA processing and degradation		
		PA4936		probable rRNA methylase	-2.20	Transcription, RNA processing and degradation		
	tRNA processing	PA0054		conserved hypothetical protein	-6.25	Hypothetical, unclassified, unknown		
		PA2795	*dusA*	dihydrouridine synthase A	3.39	Hypothetical, unclassified, unknown		
		PA3685	*yeaZ*	YeaZ	3.11	Hypothetical, unclassified, unknown		
		PA3743	*trmD*	tRNA (guanine-N1)-methyltransferase	7.64	Transcription, RNA processing and degradation		
		PA4720	*trmA*	tRNA (uracil-5-)-methyltransferase	-2.90	Transcription, RNA processing and degradation		
	RNA processing	PA4581	*rtcR*	transcriptional regulator RtcR	3.90	Transcriptional regulators		
		PA4585	*rtcA*	RNA 3'-terminal phosphate cyclase	18.18	Transcription, RNA processing and degradation		
ing	tRNA synthase	PA0008	*glyS*	glycyl-tRNA synthetase beta chain	-2.58	Translation, post-translational modification, degradation	Amino acid biosynthesis and metabolism	
		PA0018	*fmt*	methionyl-tRNA formyltransferase	-2.55	Translation, post-translational modification, degradation	Amino acid biosynthesis and metabolism	
		PA0956	*proS*	prolyl-tRNA synthetase	-2.42	Translation, post-translational modification, degradation	Amino acid biosynthesis and metabolism	
		PA1794	*glnS*	glutaminyl-tRNA synthetase	-2.51	Translation, post-translational modification, degradation	Amino acid biosynthesis and metabolism	
		PA1795	*cysS*	cysteinyl-tRNA synthetase	-2.75	Translation, post-translational modification, degradation	Amino acid biosynthesis and metabolism	
		PA2612	*serS*	seryl-tRNA synthetase	-2.25	Translation, post-translational modification, degradation	Amino acid biosynthesis and metabolism	
		PA3482	*metG*	methionyl-tRNA synthetase	-3.41	Translation, post-translational modification, degradation	Amino acid biosynthesis and metabolism	
		PA3802	*hisS*	histidyl-tRNA synthetase	-3.38	Translation, post-translational modification, degradation		
		PA3834	*valS*	valyl-tRNA synthetase	-3.38	Translation, post-translational modification, degradation	Amino acid biosynthesis and metabolism	
		PA4138	*tyrS*	tyrosyl-tRNA synthetase	5.46	Translation, post-translational modification, degradation	Amino acid biosynthesis and metabolism	
		PA4439	*trpS*	tryptophanyl-tRNA synthetase	-3.72	Translation, post-translational modification, degradation	Amino acid biosynthesis and metabolism	
		PA4482	*gatC*	Glu-tRNA(Gln) amidotransferase subunit C	-2.46	Translation, post-translational modification, degradation		
		PA4483	*gatA*	Glu-tRNA(Gln) amidotransferase subunit A	-3.88	Translation, post-translational modification, degradation		
		PA4484	*gatB*	Glu-tRNA(Gln) amidotransferase subunit B	-4.27	Translation, post-translational modification, degradation		
		PA0263.1		tRNA-Arg	12.71	Non-coding RNA gene		
		PA0668.2		tRNA-Ile	18.76	Non-coding RNA gene		
		PA0668.3		tRNA-Ala	8.52	Non-coding RNA gene		
		PA0905.1		tRNA-Ser	-3.89	Non-coding RNA gene		
		PA0905.3		tRNA-Arg	2.81	Non-coding RNA gene		
		PA0922.1		tRNA-Met	24.25	Non-coding RNA gene		
		PA0976.1		tRNA-Lys	9.99	Non-coding RNA gene		
		PA1013.1		tRNA-Ser	-4.10	Non-coding RNA gene		
		PA1796.1		tRNA-Arg	3.90	Non-coding RNA gene		
		PA1796.2		tRNA-His	2.46	Non-coding RNA gene		
		PA1796.3		tRNA-Leu	12.00	Non-coding RNA gene		
		PA1796.4		tRNA-His	29.67	Non-coding RNA gene		
		PA1804.1		tRNA-Asp	-2.50	Non-coding RNA gene		
		PA2570.1		tRNA-Leu	6.59	Non-coding RNA gene		
		PA2581.1		tRNA-Cys	5.32	Non-coding RNA gene		
		PA2583.1		tRNA-Gly	3.27	Non-coding RNA gene		
		PA2603.1		tRNA-Ser	22.60	Non-coding RNA gene		
		PA2775.1		tRNA-Val	22.38	Non-coding RNA gene		
		PA2819.1		tRNA-Gly	3.24	Non-coding RNA gene		
		PA2819.2		tRNA-Gly	3.40	Non-coding RNA gene		
		PA2819.3		tRNA-Glu	3.70	Non-coding RNA gene		
		PA2852.1		tRNA-Ser	9.54	Non-coding RNA gene		
		PA3031.1		tRNA-Pro	6.82	Non-coding RNA gene		

Translation, protein process	tRNA	PA3094.1		tRNA-Asp	6.23	Non-coding RNA gene		
		PA3094.2		tRNA-Asp	7.53	Non-coding RNA gene		
		PA3133.1		tRNA-Glu	4.40	Non-coding RNA gene		
		PA3133.4		tRNA-Ala	2.37	Non-coding RNA gene		
		PA3139.1		tRNA-Asn	-2.91	Non-coding RNA gene		
		PA3262.1		tRNA-Asp	20.07	Non-coding RNA gene		
		PA3262.2		tRNA-Val	3.30	Non-coding RNA gene		
		PA3368.1		tRNA-Arg	28.52	Non-coding RNA gene		
		PA3824.1		tRNA-Leu	22.87	Non-coding RNA gene		
		PA4280.3		tRNA-Ala	8.10	Non-coding RNA gene		
		PA4280.4		tRNA-Ile	11.06	Non-coding RNA gene		
		PA4524.1		tRNA-Thr	-8.20	Non-coding RNA gene		
		PA4541.2		tRNA-Pro	4.11	Non-coding RNA gene		
		PA4541.1		tRNA-Lys	4.51	Non-coding RNA gene		
		PA4541.3		tRNA-Asn	3.95	Non-coding RNA gene		
		PA4581.1		tRNA-Arg	7.71	Non-coding RNA gene		
		PA4669.1		tRNA-Gln	2.12	Non-coding RNA gene		
		PA4673.1		tRNA-Met	2.93	Non-coding RNA gene		
		PA4690.3		tRNA-Ala	7.83	Non-coding RNA gene		
		PA4690.4		tRNA-Ile	11.06	Non-coding RNA gene		
		PA4937.1		tRNA-Leu	2.89	Non-coding RNA gene		
		PA5149.1		tRNA-Phe	4.16	Non-coding RNA gene		
		PA5369.3		tRNA-Ala	8.57	Non-coding RNA gene		
		PA5369.4		tRNA-Ile	12.28	Non-coding RNA gene		
	Ribosomal proteins	PA0668.5		5S ribosomal RNA	6.26	Non-coding RNA gene		
		PA1530.1	*ffs*	4.5S RNA	21.22	Non-coding RNA gene		
		PA2741	*rplT*	50S ribosomal protein L20	3.07	Translation, post-translational modification, degradation		
		PA2742	*rpmI*	50S ribosomal protein L35	2.94	Translation, post-translational modification, degradation		
		PA3742	*rplS*	50S ribosomal protein L19	8.20	Translation, post-translational modification, degradation		
		PA3744	*rimM*	16S rRNA processing protein	4.17	Transcription, RNA processing and degradation		
		PA3745	*rpsP*	30S ribosomal protein S16	2.51	DNA replication, recombination, modification and repair	Translation, post-translational modification, degradation	
		PA4271	*rplL*	50S ribosomal protein L7 / L12	4.27	Translation, post-translational modification, degradation		
		PA4272	*rplJ*	50S ribosomal protein L10	4.98	Translation, post-translational modification, degradation		
		PA4273	*rplA*	50S ribosomal protein L1	2.16	Translation, post-translational modification, degradation		
		PA4433	*rplM*	50S ribosomal protein L13	2.02	Translation, post-translational modification, degradation		
		PA4567	*rpmA*	50S ribosomal protein L27	3.99	Translation, post-translational modification, degradation		
		PA4568	*rplU*	50S ribosomal protein L21	5.00	Translation, post-translational modification, degradation		
		PA4741	*rpsO*	30S ribosomal protein S15	3.04	Translation, post-translational modification, degradation		
		PA4932	*rplI*	50S ribosomal protein L9	9.27	Translation, post-translational modification, degradation		
		PA4934	*rpsR*	30S ribosomal protein S18	8.60	Translation, post-translational modification, degradation		
		PA4935	*rpsF*	30S ribosomal protein S6	8.34	Translation, post-translational modification, degradation		
		PA5049	*rpmE*	50S ribosomal protein L31	3.86	Translation, post-translational modification, degradation		
		PA5315	*rpmG*	50S ribosomal protein L33	7.46	Translation, post-translational modification, degradation		
		PA5316	*rpmB*	50S ribosomal protein L28	8.85	Translation, post-translational modification, degradation		
	Ribosome processing	PA0771	*era*	GTP-binding protein Era	-2.24	Translation, post-translational modification, degradation	Cell division	
		PA1928	*rimJ*	ribosomal protein alanine acetyltransferase	2.00	Translation, post-translational modification, degradation		
		PA3049	*rmf*	ribosome modulation factor	-200.00	Translation, post-translational modification, degradation		
		PA4743	*rbfA*	ribosome-binding factor A	5.41	Translation, post-translational modification, degradation	Adaptation, Protection	
		PA5197	*rimK*	ribosomal protein S6 modification protein	-2.30	Translation, post-translational modification, degradation	Transcription, RNA processing and degradation	
	Trans-translation tmRNA	PA0826.2	*ssrA*	tmRNA	-2.11	Amino acid biosynthesis and metabolism		
	Protease/peptidase	PA0067	*prlC*	oligopeptidase A	-3.50	Translation, post-translational modification, degradation		
		PA0459		probable ClpA/B protease ATP binding subunit	-12.99	Translation, post-translational modification, degradation		
		PA0580	*gcp*	O-sialoglycoprotein endopeptidase	5.34	Translation, post-translational modification, degradation		
		PA1242	*sprP*	SprP	8.42	Translation, post-translational modification, degradation	Motility and Attachment	
		PA2748		probable methionine aminopeptidase	4.72	Translation, post-translational modification, degradation		
		PA2973		probable peptidase	3.28	Translation, post-translational modification, degradation		
		PA3083	*pepN*	aminopeptidase N	-3.68	Translation, post-translational modification, degradation		
		PA3326	*clpP2*	ClpP2	-5.78	Translation, post-translational modification, degradation	Cell Wall / LPS / Capsule	
		PA3831	*pepA*	leucine aminopeptidase	-2.27	Translation, post-translational modification, degradation	Transcription, RNA processing and degradation	Secreted Factors (toxins, enzymes, alginate)
		PA4542	*clpB*	ClpB protein	3.10	Translation, post-translational modification, degradation		
		PA5224	*pepP*	aminopeptidase P	-3.33	Translation, post-translational modification, degradation		
	Protease inhibitor	PA2755	*eco*	ecotin precursor	2.40	Translation, post-translational modification, degradation		
	Translation initation	PA2743	*infC*	translation initiation factor IF-3	2.08	Translation, post-translational modification, degradation		
		PA4744	*infB*	translation initiation factor IF-2	2.13	Translation, post-translational modification, degradation		
	Translation elongation	PA2071	*fusA2*	elongation factor G	-2.51	Translation, post-translational modification, degradation		
		PA4807	*selB*	selenocysteine-specific elongation factor	-2.15	Translation, post-translational modification, degradation		
	Translation termination	PA3903	*prfC*	peptide chain release factor 3	-2.08	Translation, post-translational modification, degradation		
		PA5470		probable peptide chain release factor	28.78	Translation, post-translational modification, degradation		
	Chaperone	PA0538	*dsbB*	disulfide bond formation protein	-2.77	Translation, post-translational modification, degradation	Chaperones & heat shock proteins	
		PA0594	*surA*	peptidyl-prolyl cis-trans isomerase SurA	-6.25	Translation, post-translational modification, degradation	Adaptation, Protection	Chaperones & heat shock proteins
		PA2476	*dsbG*	thiol:disulfide interchange protein DsbG	-2.74	Translation, post-translational modification, degradation	Chaperones & heat shock proteins	
		PA3737	*dsbC*	thiol:disulfide interchange protein DsbC	-2.27	Translation, post-translational modification, degradation	Chaperones & heat shock proteins	
		PA4845	*dipZ*	thiol:disulfide interchange protein DipZ	3.33	Translation, post-translational modification, degradation	Chaperones & heat shock proteins	
	Peptidyl-prolyl cis/trans-isomerase	PA0837	*slyD*	peptidyl-prolyl cis-trans isomerase SlyD	-2.30	Translation, post-translational modification, degradation	Chaperones & heat shock proteins	
		PA1793	*ppiB*	peptidyl-prolyl cis-trans isomerase B	-3.94	Translation, post-translational modification, degradation	Chaperones & heat shock proteins	
		PA3227	*ppiA*	peptidyl-prolyl cis-trans isomerase A	-7.25	Translation, post-translational modification, degradation	Chaperones & heat shock proteins	
		PA3717		probable peptidyl-prolyl cis-trans isomerase, FkbP-type	2.85	Translation, post-translational modification, degradation	Chaperones & heat shock proteins	
		PA4176	*ppiC2*	peptidyl-prolyl cis-trans isomerase C2	-3.65	Translation, post-translational modification, degradation	Chaperones & heat shock proteins	
		PA4558		probable peptidyl-prolyl cis-trans isomerase, FkbP-type	-2.57	Translation, post-translational modification, degradation	Chaperones & heat shock proteins	
	Post-translational modification	PA2617	*aat*	leucyl/phenylalanyl-tRNA-protein transferase	-3.55	Translation, post-translational modification, degradation		
		PA2618		hypothetical protein	-5.00	Translation, post-translational modification, degradation		
		PA3818		extragenic suppressor protein SuhB	2.19	Translation, post-translational modification, degradation	Adaptation, Protection	
		PA3824	*queA*	S-adenosylmethionine:trna ribosyltransferase-isomerase	3.82	Translation, post-translational modification, degradation		
		PA4945	*miaA*	delta 2-isopentenylpyrophosphate transferase	2.01	Translation, post-translational modification, degradation	Adaptation, Protection	
	Protein repair	PA5018	*msrA*	peptide methionine sulfoxide reductase	-4.85	Translation, post-translational modification, degradation		
Bacterial transport systems	Potassium transport	PA0016	*trkA*	potassium uptake protein TrkA	-3.88	Transport of small molecules		
		PA1207	*kefB*	glutathione-regulated potassium-efflux system protein KefB	2.61	Transport of small molecules		
		PA1632	*kdpF*	KdpF protein	40.07	Transport of small molecules		
		PA1633	*kdpA*	potassium-transporting ATPase, A chain	5.06	Transport of small molecules		
		PA1634	*kdpB*	potassium-transporting ATPase, B chain	3.49	Transport of small molecules		
		PA1635	*kdpC*	potassium-transporting ATPase, C chain	2.41	Transport of small molecules		
		PA3210	*trkH*	potassium uptake protein TrkH	2.14	Membrane proteins	Transport of small molecules	
		PA5518		probable potassium efflux transporter	6.06	Transport of small molecules	Membrane proteins	
	Magnesium transport	PA0913	*mgtE*	MgtE	12.97	Transport of small molecules		
		PA4822		hypothetical protein	9.30	Membrane proteins		
		PA4823		hypothetical protein	14.66	Hypothetical, unclassified, unknown		
		PA4824		hypothetical protein	13.55	Hypothetical, unclassified, unknown		
		PA4825	*mgtA*	Mg(2+) transport ATPase, P-type 2	7.10	Transport of small molecules		
		PA4826		hypothetical protein	4.66	Hypothetical, unclassified, unknown		
	Zinc transport	PA1922		probable TonB-dependent receptor	2.66	Transport of small molecules		
		PA2911		probable TonB-dependent receptor	7.95	Membrane proteins	Transport of small molecules	
		PA2913		hypothetical protein	2.53	Transport of small molecules		
		PA2914		probable permease of ABC transporter	5.32	Membrane proteins	Transport of small molecules	
		PA4063		hypothetical protein	-4.22	Hypothetical, unclassified, unknown		
		PA4064		probable ATP-binding component of ABC transporter	-5.56	Transport of small molecules		
		PA4065		hypothetical protein	-4.03	Membrane proteins		
		PA4066		hypothetical protein	-2.24	Hypothetical, unclassified, unknown		
		PA4834	*cntI*	CntI	2.45	Membrane proteins		
		PA4835	*cntM*	CntM	2.96	Membrane proteins		
		PA4837	*cntO*	CntO	2.17	Membrane proteins	Transport of small molecules	
	Na+/H+ antiport	PA1055	*shaB*	ShaB	-3.01	Membrane proteins	Transport of small molecules	
		PA1056	*shaC*	ShaC	-2.23	Membrane proteins	Transport of small molecules	Putative enzymes
		PA1057	*shaD*	ShaD	-2.84	Membrane proteins	Transport of small molecules	
		PA1058	*shaE*	ShaE	-3.06	Membrane proteins	Transport of small molecules	
		PA1059	*shaF*	ShaF	-5.68	Membrane proteins	Transport of small molecules	
		PA2135		probable transporter	2.75	Membrane proteins	Transport of small molecules	
		PA3739		probable sodium/hydrogen antiporter	-2.38	Membrane proteins	Transport of small molecules	
		PA5021		probable sodium/hydrogen antiporter	2.18	Membrane proteins	Transport of small molecules	
	Copper transport	PA0114	*senC*	SenC	-6.90	Hypothetical, unclassified, unknown		
		PA1549	*copA2*	CopA2	-8.33	Membrane proteins	Transport of small molecules	
		PA3790	*oprC*	Putative copper transport outer membrane porin OprC precursor	-19.23	Transport of small molecules		
		PA3920	*copA1*	CopA1	-3.26	Membrane proteins	Transport of small molecules	
	Divalent metal cation/H+ antiporter	PA2520	*czcA*	Resistance-Nodulation-Cell Division (RND) divalent metal cation efflux transporter CzcA	13.93	Membrane proteins	Transport of small molecules	
		PA2521	*czcB*	Resistance-Nodulation-Cell Division (RND) divalent metal cation efflux membrane fusion protein CzcB precursor	22.24	Membrane proteins	Transport of small molecules	
		PA2522	*czcC*	outer membrane protein precursor CzcC	28.97	Membrane proteins	Transport of small molecules	
	Na+/solute symport	PA1418		probable sodium:solute symport protein	3.80	Transport of small molecules		
		PA3234		probable sodium:solute symporter	4.76	Membrane proteins	Transport of small molecules	
	Manganese transport	PA2910		conserved hypothetical protein	11.58	Membrane proteins		
	Ions transport	PA0809		probable transporter	8.81	Membrane proteins	Transport of small molecules	
		PA1297		probable metal transporter	7.59	Membrane proteins	Transport of small molecules	
		PA1773	*cmaX*	CmaX protein	5.94	Membrane proteins		
		PA1873		hypothetical protein	2.68	Membrane proteins		
		PA3963		probable transporter	2.48	Membrane proteins	Transport of small molecules	
		PA4467		hypothetical protein	-9.62	Membrane proteins		
	Unknow function	PA2057	*sppR*	TonB-dependent receptor, SppR	7.31	Hypothetical, unclassified, unknown		
		PA2058	*sppA*	ABC transporter substrate-binding protein, SppA	13.08	Transport of small molecules		
		PA2059	*sppB*	ABC transporter permease, SppB	23.43	Membrane proteins	Transport of small molecules	
		PA2060	*sppC*	ABC transporter permease, SppC	8.45	Transport of small molecules		
		PA2061	*sppD*	ABC transporter ATP-binding protein, SppD	3.11	Transport of small molecules		
		PA4590	*pra*	protein activator	-13.70	Transport of small molecules	Carbon compound catabolism	
	Putative transporters	PA0040		conserved hypothetical protein	11.81	Hypothetical, unclassified, unknown		
		PA0189		probable porin	8.90	Membrane proteins	Transport of small molecules	
		PA0192		probable TonB-dependent receptor	6.90	Membrane proteins	Transport of small molecules	
		PA0204		probable permease of ABC transporter	14.10	Membrane proteins	Transport of small molecules	
		PA0205		probable permease of ABC transporter	4.91	Membrane proteins	Transport of small molecules	
		PA0206		probable ATP-binding component of ABC transporter	5.03	Transport of small molecules		
		PA0239		hypothetical protein	2.10	Membrane proteins		
		PA0240		probable porin	3.50	Membrane proteins	Transport of small molecules	
		PA0241		probable major facilitator superfamily (MFS) transporter	4.81	Membrane proteins	Transport of small molecules	
		PA0246		probable major facilitator superfamily (MFS) transporter	7.08	Membrane proteins	Transport of small molecules	
		PA0273		probable major facilitator superfamily (MFS) transporter	6.99	Membrane proteins	Transport of small molecules	
		PA0324		probable permease of ABC transporter	6.84	Membrane proteins	Transport of small molecules	
		PA0325		probable permease of ABC transporter	11.70	Membrane proteins	Transport of small molecules	
		PA0326		probable ATP-binding component of ABC transporter	15.30	Transport of small molecules		
		PA0476		probable permease	11.12	Transport of small molecules		
		PA0786		probable transporter	2.48	Membrane proteins	Transport of small molecules	
		PA0790		hypothetical protein	3.01	Membrane proteins		
		PA0860		probable ATP-binding/permease fusion ABC transporter	3.71	Membrane proteins	Transport of small molecules	
		PA1025		probable porin	2.04	Membrane proteins	Transport of small molecules	
		PA1041		probable outer membrane protein precursor	-15.63	Membrane proteins	Transport of small molecules	
		PA1048		probable outer membrane protein precursor	-8.13	Membrane proteins	Transport of small molecules	
		PA1108		probable major facilitator superfamily (MFS) transporter	10.54	Membrane proteins	Transport of small molecules	
		PA1144		probable major facilitator superfamily (MFS) transporter	6.61	Membrane proteins	Transport of small molecules	
		PA1212		probable major facilitator superfamily (MFS) transporter	-19.61	Membrane proteins	Transport of small molecules	
		PA1230		hypothetical protein	25.64	Membrane proteins		
		PA1231		conserved hypothetical protein	8.50	Membrane proteins		
		PA1232		hypothetical protein	4.61	Membrane proteins		
		PA1236		probable major facilitator superfamily (MFS) transporter	9.05	Membrane proteins	Transport of small molecules	
		PA1262		probable major facilitator superfamily (MFS) transporter	7.26	Membrane proteins	Transport of small molecules	
		PA1271		probable tonB-dependent receptor	4.95	Transport of small molecules		
		PA1282		probable major facilitator superfamily (MFS) transporter	5.60	Membrane proteins	Transport of small molecules	
		PA1286		probable major facilitator superfamily (MFS) transporter	5.74	Membrane proteins	Transport of small molecules	
		PA1313		probable major facilitator superfamily (MFS) transporter	4.65	Membrane proteins	Transport of small molecules	
		PA1386		probable ATP-binding component of ABC transporter	2.74	Transport of small molecules		
		PA1419		probable transporter	12.10	Membrane proteins	Transport of small molecules	
		PA1425		probable ATP-binding component of ABC transporter	3.95	Transport of small molecules		
		PA1435		probable Resistance-Nodulation-Cell Division (RND) efflux membrane fusion protein precursor	37.56	Transport of small molecules		
		PA1436		probable Resistance-Nodulation-Cell Division (RND) efflux transporter	42.51	Membrane proteins	Transport of small molecules	
		PA1569		probable major facilitator superfamily (MFS) transporter	3.57	Membrane proteins	Transport of small molecules	
		PA1735		hypothetical protein	3.52	Membrane proteins		
		PA1848		probable major facilitator superfamily (MFS) transporter	3.77	Membrane proteins	Transport of small molecules	
		PA1882		probable transporter	9.83	Membrane proteins	Transport of small molecules	
		PA1964		probable ATP-binding component of ABC transporter	2.63	Transport of small molecules		
		PA1993		probable major facilitator superfamily (MFS) transporter	3.98	Membrane proteins	Transport of small molecules	
		PA2006		probable major facilitator superfamily (MFS) transporter	-5.46	Membrane proteins	Transport of small molecules	
		PA2042		probable transporter (membrane subunit)	-2.65	Transport of small molecules		
		PA2055		probable major facilitator superfamily (MFS) transporter	3.51	Membrane proteins	Antibiotic resistance and susceptibility	Transport of small molecules
		PA2068		probable major facilitator superfamily (MFS) transporter	-33.33	Membrane proteins	Transport of small molecules	
		PA2073		probable transporter (membrane subunit)	3.56	Transport of small molecules		
		PA2092		probable major facilitator superfamily (MFS) transporter	2.30	Membrane proteins	Transport of small molecules	
		PA2114		probable major facilitator superfamily (MFS) transporter	-8.00	Membrane proteins	Transport of small molecules	
		PA2210		probable major facilitator superfamily (MFS) transporter	14.37	Membrane proteins	Transport of small molecules	
		PA2213		probable porin	7.31	Membrane proteins	Transport of small molecules	
		PA2219	*opdE*	membrane protein OpdE	2.64	Membrane proteins	Transport of small molecules	
		PA2269		conserved hypothetical protein	2.49	Membrane proteins		
		PA2294		probable ATP-binding component of ABC transporter	2.05	Transport of small molecules		
		PA2295		probable permease of ABC transporter	2.44	Membrane proteins	Transport of small molecules	
		PA2307		probable permease of ABC transporter	4.81	Membrane proteins	Transport of small molecules	
		PA2308		probable ATP-binding component of ABC transporter	3.24	Transport of small molecules		
		PA2314		probable major facilitator superfamily (MFS) transporter	6.57	Membrane proteins	Transport of small molecules	
		PA2327		probable permease of ABC transporter	-3.08	Membrane proteins	Transport of small molecules	
		PA2329		probable ATP-binding component of ABC transporter	-9.90	Transport of small molecules		
		PA2335		probable TonB-dependent receptor	14.53	Transport of small molecules		
		PA2339		probable binding-protein-dependent maltose/mannitol transport protein	2.26	Transport of small molecules		
		PA2350		probable ATP-binding component of ABC transporter	2.06	Transport of small molecules		
		PA2351		probable permease of ABC transporter	3.32	Membrane proteins	Transport of small molecules	
		PA2420		probable porin	3.23	Membrane proteins	Transport of small molecules	
		PA2472		probable major facilitator superfamily (MFS) transporter	12.29	Membrane proteins	Transport of small molecules	
		PA2558		probable transport protein	2.97	Membrane proteins	Transport of small molecules	
		PA2589		hypothetical protein	10.24	Membrane proteins		
		PA2653		probable transporter	6.74	Membrane proteins	Transport of small molecules	
		PA2678		probable permease of ABC-2 transporter	19.56	Membrane proteins	Transport of small molecules	
		PA2701		probable major facilitator superfamily (MFS) transporter	-2.75	Membrane proteins	Transport of small molecules	
		PA2710		hypothetical protein	-5.95	Membrane proteins		
		PA2760	*oprQ*	OprQ	-3.33	Antibiotic resistance and susceptibility	Transport of small molecules	Motility and Attachment
		PA2777		conserved hypothetical protein	17.79	Membrane proteins		
		PA2811		probable permease of ABC-2 transporter	-4.17	Membrane proteins	Transport of small molecules	
		PA2812		probable ATP-binding component of ABC transporter	-3.33	Transport of small molecules		
		PA2900		probable outer membrane protein precursor	2.95	Membrane proteins	Transport of small molecules	
		PA2933		probable major facilitator superfamily (MFS) transporter	3.10	Membrane proteins	Transport of small molecules	
		PA3019		probable ATP-binding component of ABC transporter	2.68	Transport of small molecules		
		PA3136		probable secretion protein	2.69	Transport of small molecules		
		PA3137		probable major facilitator superfamily (MFS) transporter	2.51	Membrane proteins	Transport of small molecules	
		PA3228		probable ATP-binding/permease fusion ABC transporter	-5.05	Membrane proteins	Transport of small molecules	
		PA3252		probable permease of ABC transporter	-3.82	Membrane proteins	Transport of small molecules	
		PA3253		probable permease of ABC transporter	-2.56	Membrane proteins	Transport of small molecules	
		PA3254		probable ATP-binding component of ABC transporter	-5.29	Transport of small molecules		
		PA3265		probable transporter	3.48	Membrane proteins	Transport of small molecules	
		PA3267		hypothetical protein	2.26	Membrane proteins		
		PA3305		hypothetical protein	2.60	Membrane proteins		
		PA3313		hypothetical protein	-8.33	Transport of small molecules		
		PA3314		probable ATP-binding component of ABC transporter	-3.51	Transport of small molecules		
		PA3315		probable permease of ABC transporter	-2.35	Membrane proteins	Transport of small molecules	
		PA3316		probable permease of ABC transporter	-2.42	Membrane proteins	Transport of small molecules	
		PA3336		probable major facilitator superfamily (MFS) transporter	-3.55	Membrane proteins	Transport of small molecules	
		PA3467		probable major facilitator superfamily (MFS) transporter	3.98	Membrane proteins	Transport of small molecules	
		PA3512		probable permease of ABC transporter	4.92	Membrane proteins	Transport of small molecules	
		PA3514		probable ATP-binding component of ABC transporter	8.45	Transport of small molecules		
		PA3532		hypothetical protein	3.38	Membrane proteins		
		PA3573		probable major facilitator superfamily (MFS) transporter	16.81	Adaptation, Protection	Transport of small molecules	
		PA3595		probable major facilitator superfamily (MFS) transporter	4.00	Membrane proteins	Transport of small molecules	
		PA3647		probable outer membrane protein precursor	-5.15	Membrane proteins	Transport of small molecules	
		PA3671		probable permease of ABC transporter	-2.53	Membrane proteins	Transport of small molecules	
		PA3692	*lptF*	Lipotoxin F, LptF	-2.18	Membrane proteins	Transport of small molecules	
		PA3718		probable major facilitator superfamily (MFS) transporter	5.73	Membrane proteins	Transport of small molecules	
		PA3749		probable major facilitator superfamily (MFS) transporter	4.94	Membrane proteins	Transport of small molecules	
		PA3753		conserved hypothetical protein	-2.26	Transport of small molecules		
		PA3773		hypothetical protein	7.06	Membrane proteins		
		PA3781		probable transporter	2.10	Membrane proteins	Transport of small molecules	
		PA3838		probable ATP-binding component of ABC transporter	-2.68	Transport of small molecules		
		PA3893		conserved hypothetical protein	2.37	Membrane proteins		
		PA3894		probable outer membrane protein precursor	3.47	Membrane proteins		
		PA3926		probable major facilitator superfamily (MFS) transporter	2.07	Membrane proteins	Transport of small molecules	
		PA3934		conserved hypothetical protein	-4.44	Membrane proteins		
		PA4067	*oprG*	Outer membrane protein OprG precursor	-15.87	Membrane proteins		
		PA4096		probable major facilitator superfamily (MFS) transporter	17.36	Membrane proteins	Transport of small molecules	
		PA4113		probable major facilitator superfamily (MFS) transporter	5.33	Membrane proteins	Transport of small molecules	
		PA4136		probable major facilitator superfamily (MFS) transporter	4.06	Transport of small molecules	Membrane proteins	
		PA4137		probable porin	2.42	Transport of small molecules		
		PA4179		probable porin	14.65	Membrane proteins	Transport of small molecules	
		PA4187		probable major facilitator superfamily (MFS) transporter	7.52	Membrane proteins	Transport of small molecules	
		PA4192		probable ATP-binding component of ABC transporter	2.64	Transport of small molecules		
		PA4193		probable permease of ABC transporter	8.30	Transport of small molecules	Membrane proteins	
		PA4194		probable permease of ABC transporter	5.26	Transport of small molecules	Membrane proteins	
		PA4195		probable binding protein component of ABC transporter	4.25	Transport of small molecules		
		PA4343		probable major facilitator superfamily (MFS) transporter	16.80	Membrane proteins	Transport of small molecules	
		PA4355	*pyeM*	PyeM	4.70	Membrane proteins	Transport of small molecules	Transcriptional regulators
		PA4456		probable ATP-binding component of ABC transporter	2.99	Transport of small molecules		
		PA4589		probable outer membrane protein precursor	-3.73	Membrane proteins	Transport of small molecules	
		PA4595		probable ATP-binding component of ABC transporter	-3.41	Transport of small molecules		
		PA4654		probable major facilitator superfamily (MFS) transporter	10.58	Membrane proteins	Transport of small molecules	
		PA4804		POTRA-like domain-containing usher, CupB3	6.82	Membrane proteins	Transport of small molecules	Protein secretion/export apparatus
		PA4821		probable transporter	3.81	Membrane proteins	Transport of small molecules	
		PA4859		probable permease of ABC transporter	6.53	Transport of small molecules	Membrane proteins	
		PA4860		probable permease of ABC transporter	5.04	Transport of small molecules	Membrane proteins	
		PA4861		probable ATP-binding component of ABC transporter	2.17	Transport of small molecules		
		PA4887		probable major facilitator superfamily (MFS) transporter	3.92	Membrane proteins	Transport of small molecules	
		PA4900		probable major facilitator superfamily (MFS) transporter	5.30	Membrane proteins	Transport of small molecules	
		PA4903		probable major facilitator superfamily (MFS) transporter	6.34	Membrane proteins	Transport of small molecules	
		PA4990		SMR multidrug efflux transporter	4.09	Membrane proteins	Antibiotic resistance and susceptibility	Transport of small molecules
		PA5074		probable ATP-binding component of ABC transporter	-3.38	Transport of small molecules		
		PA5075		probable permease of ABC transporter	-2.56	Transport of small molecules	Membrane proteins	
		PA5094		probable ATP-binding component of ABC transporter	-2.07	Transport of small molecules		
		PA5099		probable transporter	2.04	Transport of small molecules	Membrane proteins	
		PA5159		multidrug resistance protein	-3.10	Transport of small molecules	Antibiotic resistance and susceptibility	
		PA5230		probable permease of ABC transporter	-6.94	Membrane proteins	Transport of small molecules	
		PA5231		probable ATP-binding/permease fusion ABC transporter	-4.63	Membrane proteins	Transport of small molecules	

		PA5282		probable major facilitator superfamily (MFS) transporter	7.37	Membrane proteins	Transport of small molecules
		PA5311		probable major facilitator superfamily (MFS) transporter	16.34	Membrane proteins	Transport of small molecules
		PA5370		probable major facilitator superfamily (MFS) transporter	2.74	Transport of small molecules	Membrane proteins
		PA5503		probable ATP-binding component of ABC transporter	6.72	Transport of small molecules	
		PA5505		probable TonB-dependent receptor	-2.21	Transport of small molecules	Membrane proteins
		PA5548		probable major facilitator superfamily (MFS) transporter	3.01	Transport of small molecules	Membrane proteins
Membrane proteins	Unknown function	PA0007		hypothetical protein	-2.20	Membrane proteins	
		PA0043		hypothetical protein	2.18	Membrane proteins	
		PA0109		hypothetical protein	-2.44	Membrane proteins	
		PA0276		hypothetical protein	4.07	Membrane proteins	
		PA0349		hypothetical protein	14.65	Membrane proteins	
		PA0452		probable stomatin-like protein	5.84	Membrane proteins	
		PA0539		hypothetical protein	20.88	Membrane proteins	
		PA0659		hypothetical protein	-2.95	Membrane proteins	
		PA0661		conserved hypothetical protein	-2.66	Membrane proteins	
		PA0736		hypothetical protein	-2.27	Membrane proteins	
		PA0738		conserved hypothetical protein	11.84	Membrane proteins	
		PA0751		conserved hypothetical protein	2.29	Membrane proteins	
		PA0801		hypothetical protein	-3.30	Membrane proteins	
		PA0802		hypothetical protein	-4.31	Membrane proteins	
		PA0833		hypothetical protein	5.92	Membrane proteins	
		PA0924		hypothetical protein	2.02	Membrane proteins	
		PA1007		conserved hypothetical protein	9.00	Membrane proteins	
		PA1042		conserved hypothetical protein	-2.39	Membrane proteins	
		PA1044		hypothetical protein	2.97	Membrane proteins	
		PA1060		hypothetical protein	-2.79	Membrane proteins	
		PA1115		hypothetical protein	2.38	Membrane proteins	
		PA1170		conserved hypothetical protein	12.73	Membrane proteins	
		PA1265		hypothetical protein	21.37	Membrane proteins	
		PA1305		hypothetical protein	2.30	Membrane proteins	
		PA1352		conserved hypothetical protein	4.66	Membrane proteins	
		PA1360		conserved hypothetical protein	9.79	Membrane proteins	
		PA1513		hypothetical protein	-4.26	Membrane proteins	
		PA1591		hypothetical protein	8.65	Membrane proteins	
		PA1625		conserved hypothetical protein	5.63	Membrane proteins	
		PA1676		hypothetical protein	4.09	Membrane proteins	
		PA1767		hypothetical protein	-2.23	Membrane proteins	
		PA1824		conserved hypothetical protein	3.99	Membrane proteins	
		PA1891		hypothetical protein	-6.80	Membrane proteins	
		PA1895		hypothetical protein	-16.13	Membrane proteins	
		PA1897		hypothetical protein	-10.53	Membrane proteins	
		PA1960		hypothetical protein	17.56	Membrane proteins	
		PA1977		hypothetical protein	8.32	Membrane proteins	
		PA2004		conserved hypothetical protein	-2.86	Membrane proteins	
		PA2148		conserved hypothetical protein	2.48	Membrane proteins	
		PA2313		hypothetical protein	2.95	Membrane proteins	
		PA2331		hypothetical protein	-10.87	Membrane proteins	
		PA2375		hypothetical protein	-2.20	Membrane proteins	
		PA2415		hypothetical protein	4.78	Membrane proteins	
		PA2439		hypothetical protein	2.89	Membrane proteins	
		PA2465		hypothetical protein	2.20	Membrane proteins	
		PA2549		conserved hypothetical protein	2.57	Membrane proteins	
		PA2568		hypothetical protein	8.19	Membrane proteins	
		PA2576		hypothetical protein	5.29	Membrane proteins	
		PA2746		hypothetical protein	-3.79	Membrane proteins	
		PA2752		conserved hypothetical protein	4.21	Membrane proteins	
		PA2757		hypothetical protein	2.23	Membrane proteins	
		PA2761		hypothetical protein	-4.17	Membrane proteins	
		PA2763		hypothetical protein	2.52	Membrane proteins	
		PA2768		hypothetical protein	4.87	Membrane proteins	
		PA2773		hypothetical protein	8.96	Membrane proteins	
		PA2853	*oprI*	Outer membrane lipoprotein OprI precursor	-2.87	Membrane proteins	
		PA2858		conserved hypothetical protein	-2.06	Membrane proteins	
		PA2868		hypothetical protein	2.94	Membrane proteins	
		PA2936		hypothetical protein	2.44	Membrane proteins	
		PA2985		hypothetical protein	2.74	Membrane proteins	
		PA3041		hypothetical protein	-5.85	Membrane proteins	
		PA3079		hypothetical protein	3.06	Membrane proteins	
		PA3086		hypothetical protein	-2.27	Membrane proteins	
		PA3216		hypothetical protein	-2.42	Membrane proteins	
		PA3222		hypothetical protein	-4.90	Membrane proteins	
		PA3235		conserved hypothetical protein	11.00	Membrane proteins	
		PA3281		hypothetical protein	3.19	Membrane proteins	
		PA3320		hypothetical protein	19.66	Membrane proteins	
		PA3358		hypothetical protein	3.39	Membrane proteins	
		PA3359		hypothetical protein	7.52	Membrane proteins	
		PA3362		hypothetical protein	-2.20	Membrane proteins	
		PA3369		hypothetical protein	4.78	Membrane proteins	
		PA3370		hypothetical protein	4.79	Membrane proteins	
		PA3403		hypothetical protein	15.68	Membrane proteins	
		PA3432		hypothetical protein	2.22	Membrane proteins	
		PA3465		conserved hypothetical protein	-3.50	Membrane proteins	
		PA3473		hypothetical protein	3.31	Membrane proteins	
		PA3474		conserved hypothetical protein	3.96	Membrane proteins	
		PA3730		hypothetical protein	5.39	Membrane proteins	
		PA3748		conserved hypothetical protein	-3.33	Membrane proteins	
		PA3775		hypothetical protein	4.55	Membrane proteins	
		PA3789		hypothetical protein	-6.58	Membrane proteins	
		PA3794		hypothetical protein	5.25	Membrane proteins	
		PA3819		conserved hypothetical protein	6.43	Membrane proteins	
		PA3826		hypothetical protein	4.03	Membrane proteins	
		PA3897		hypothetical protein	54.53	Membrane proteins	
		PA3966		hypothetical protein	3.03	Membrane proteins	
		PA4008		probable hydrolase	12.76	Membrane proteins	Putative enzymes
		PA4013		conserved hypothetical protein	3.03	Membrane proteins	
		PA4014		hypothetical protein	3.03	Membrane proteins	
		PA4046		hypothetical protein	3.63	Membrane proteins	
		PA4287		hypothetical protein	11.55	Membrane proteins	
		PA4383		conserved hypothetical protein	2.93	Membrane proteins	
		PA4586		hypothetical protein	72.26	Membrane proteins	
		PA4754		hypothetical protein	-2.00	Membrane proteins	
		PA4779		hypothetical protein	2.22	Membrane proteins	
		PA4783		conserved hypothetical protein	8.87	Membrane proteins	
		PA4815		hypothetical protein	2.45	Membrane proteins	
		PA4818		conserved hypothetical protein	5.75	Membrane proteins	
		PA4849		hypothetical protein	2.87	Membrane proteins	
		PA4894		hypothetical protein	-6.76	Membrane proteins	
		PA4924		conserved hypothetical protein	4.62	Membrane proteins	
		PA4933		hypothetical protein	9.20	Membrane proteins	
		PA4940		conserved hypothetical protein	3.96	Membrane proteins	
		PA4961		hypothetical protein	-2.10	Membrane proteins	
		PA5114		hypothetical protein	4.13	Membrane proteins	
		PA5151		hypothetical protein	9.38	Membrane proteins	
		PA5205		conserved hypothetical protein	-2.43	Membrane proteins	
		PA5264		hypothetical protein	2.98	Membrane proteins	
		PA5469		conserved hypothetical protein	13.16	Membrane proteins	
		PA5473		conserved hypothetical protein	10.74	Membrane proteins	
		PA5477		hypothetical protein	3.38	Membrane proteins	
		PA5478		conserved hypothetical protein	2.37	Membrane proteins	
Signal transduction	Signalling systems	PA0172	*siaA*	SiaA	6.09	Motility and Attachment	
		PA0991	*hptA*	Histidine phosphotransfer protein HptA	-2.02	Two-component regulatory systems	
		PA1119	*yfiB*	YfiB	-12.05	Membrane proteins	Cell Wall / LPS / Capsule
		PA5346	*sadB*	SadB	-4.69	Cell Wall / LPS / Capsule	
	Copper sensing	PA4778	*cueR*	CueR	-3.18	Transcriptional regulators	
Regulation	General regulation	PA4315	*mvaT*	transcriptional regulator MvaT, P16 subunit	-2.39	Transcriptional regulators	
		PA0576	*rpoD*	sigma factor RpoD	2.11	Transcriptional regulators	
	Stationnary phase regulation	PA3622	*rpoS*	sigma factor RpoS	-16.13	Transcriptional regulators	
	Non-coding RNA	PA0836.1	*P5*	P5	108.54	Non-coding RNA gene	
		PA1030.1	*P8*	P8	17.50	Non-coding RNA gene	
		PA1324.1	*P9*	P9	-8.13	Non-coding RNA gene	
		PA2744.1	*P13*	P13	65.26	Non-coding RNA gene	
		PA2942.1	*P15*	P15	2.08	Non-coding RNA gene	
		PA3304.1	*P18*	P18	3.21	Non-coding RNA gene	
		PA4270.1	*P26*	P26	3.22	Non-coding RNA gene	
		PA4406.1			-5.26	Non-coding RNA gene	
		PA5181.1	*P34*	P34	29.05	Non-coding RNA gene	
		PA5316.1			3.28	Non-coding RNA gene	
	Putative regulators	PA0032		probable transcriptional regulator	4.78	Transcriptional regulators	
		PA0034		probable two-component response regulator	2.81	Two-component regulatory systems	Transcriptional regulators
		PA0048		probable transcriptional regulator	-33.33	Transcriptional regulators	
		PA0056		probable transcriptional regulator	4.36	Transcriptional regulators	
		PA0123		probable transcriptional regulator	6.76	Transcriptional regulators	
		PA0159		probable transcriptional regulator	-2.16	Transcriptional regulators	
		PA0167		probable transcriptional regulator	-3.12	Transcriptional regulators	
		PA0191		probable transcriptional regulator	4.33	Transcriptional regulators	
		PA0207		probable transcriptional regulator	3.22	Transcriptional regulators	
		PA0218		probable transcriptional regulator	2.12	Transcriptional regulators	
		PA0248		probable transcriptional regulator	2.10	Transcriptional regulators	
		PA0436		probable transcriptional regulator	-2.34	Transcriptional regulators	
		PA0528		probable transcriptional regulator	3.35	Transcriptional regulators	
		PA0535		probable transcriptional regulator	3.10	Transcriptional regulators	
		PA0547		probable transcriptional regulator	-5.21	Transcriptional regulators	
		PA0756		probable two-component response regulator	2.94	Two-component regulatory systems	Transcriptional regulators
		PA0757		probable two-component sensor	3.10	Two-component regulatory systems	
		PA0784		probable transcriptional regulator	-4.20	Transcriptional regulators	
		PA0797		probable transcriptional regulator	2.22	Transcriptional regulators	
		PA0816		probable transcriptional regulator	2.75	Transcriptional regulators	
		PA0828		probable transcriptional regulator	2.84	Transcriptional regulators	
		PA0839		probable transcriptional regulator	6.12	Transcriptional regulators	
		PA0877		probable transcriptional regulator	2.26	Transcriptional regulators	
		PA0942		probable transcriptional regulator	3.15	Transcriptional regulators	
		PA1067		probable transcriptional regulator	2.63	Transcriptional regulators	
		PA1109		probable transcriptional regulator	7.03	Transcriptional regulators	
		PA1136		probable transcriptional regulator	3.87	Transcriptional regulators	
		PA1138		probable transcriptional regulator	3.57	Transcriptional regulators	
		PA1157		probable two-component response regulator	2.65	Two-component regulatory systems	Transcriptional regulators
		PA1158		probable two-component sensor	4.65	Two-component regulatory systems	
		PA1229		probable transcriptional regulator	3.24	Transcriptional regulators	
		PA1241		probable transcriptional regulator	4.20	Transcriptional regulators	
		PA1243		probable sensor/response regulator hybrid	-2.04	Two-component regulatory systems	
		PA1290		probable transcriptional regulator	4.47	Transcriptional regulators	
		PA1347		probable transcriptional regulator	5.06	Transcriptional regulators	
		PA1359		probable transcriptional regulator	5.90	Transcriptional regulators	
		PA1380		probable transcriptional regulator	2.08	Transcriptional regulators	
		PA1397		probable two-component response regulator	2.02	Two-component regulatory systems	Transcriptional regulators
		PA1403		probable transcriptional regulator	2.58	Transcriptional regulators	
		PA1437		probable two-component response regulator	3.28	Two-component regulatory systems	Transcriptional regulators
		PA1438		probable two-component sensor	2.75	Two-component regulatory systems	
		PA1570		probable transcriptional regulator	2.49	Transcriptional regulators	
		PA1627		probable transcriptional regulator	2.05	Transcriptional regulators	
		PA1630		probable transcriptional regulator	2.47	Transcriptional regulators	
		PA1653		probable transcriptional regulator	3.99	Transcriptional regulators	
		PA1760		probable transcriptional regulator	-2.29	Transcriptional regulators	
		PA1826		probable transcriptional regulator	-2.86	Transcriptional regulators	
		PA1836		probable transcriptional regulator	2.57	Transcriptional regulators	
		PA1850		probable transcriptional regulator	4.45	Transcriptional regulators	
		PA1853		probable transcriptional regulator	7.70	Transcriptional regulators	
		PA1864		probable transcriptional regulator	2.29	Transcriptional regulators	
		PA1945		probable transcriptional regulator	2.62	Transcriptional regulators	
		PA2056		probable transcriptional regulator	2.17	Transcriptional regulators	
		PA2123		probable transcriptional regulator	-3.11	Transcriptional regulators	
		PA2276		probable transcriptional regulator	2.63	Transcriptional regulators	
		PA2281		probable transcriptional regulator	4.17	Transcriptional regulators	
		PA2312		probable transcriptional regulator	7.28	Transcriptional regulators	
		PA2334		probable transcriptional regulator	7.91	Transcriptional regulators	
		PA2376		probable transcriptional regulator	6.43	Transcriptional regulators	
		PA2417		probable transcriptional regulator	9.15	Transcriptional regulators	
		PA2447		probable transcriptional regulator	3.18	Transcriptional regulators	
		PA2469		probable transcriptional regulator	2.08	Transcriptional regulators	
		PA2488		probable transcriptional regulator	3.84	Transcriptional regulators	
		PA2571		probable two-component sensor	2.68	Two-component regulatory systems	
		PA2577		probable transcriptional regulator	-2.71	Transcriptional regulators	
		PA2583		probable sensor/response regulator hybrid	16.25	Two-component regulatory systems	Transcriptional regulators
		PA2588		probable transcriptional regulator	-8.33	Transcriptional regulators	
		PA2601		probable transcriptional regulator	6.64	Transcriptional regulators	
		PA2692		probable transcriptional regulator	2.46	Transcriptional regulators	
		PA2704		probable transcriptional regulator	2.76	Transcriptional regulators	
		PA2758		probable transcriptional regulator	4.52	Transcriptional regulators	
		PA2766		probable transcriptional regulator	4.01	Transcriptional regulators	
		PA2838		probable transcriptional regulator	2.11	Transcriptional regulators	
		PA2846		probable transcriptional regulator	-2.10	Transcriptional regulators	
		PA2881		probable two-component response regulator	2.19	Two-component regulatory systems	Transcriptional regulators
		PA2882		probable two-component sensor	19.46	Two-component regulatory systems	
		PA2917		probable transcriptional regulator	2.14	Transcriptional regulators	
		PA2921		probable transcriptional regulator	3.85	Transcriptional regulators	
		PA2930		probable transcriptional regulator	3.77	Transcriptional regulators	
		PA3122		probable transcriptional regulator	-2.14	Transcriptional regulators	
		PA3215		probable transcriptional regulator	2.07	Transcriptional regulators	
		PA3249		probable transcriptional regulator	-2.14	Transcriptional regulators	
		PA3260		probable transcriptional regulator	2.87	Transcriptional regulators	
		PA3269		probable transcriptional regulator	4.58	Transcriptional regulators	
		PA3271		probable two-component sensor	2.01	Two-component regulatory systems	
		PA3381		probable transcriptional regulator	3.85	Transcriptional regulators	
		PA3420		probable transcriptional regulator	4.81	Transcriptional regulators	
		PA3462		probable sensor/response regulator hybrid	-2.28	Two-component regulatory systems	
		PA3565		probable transcriptional regulator	2.86	Transcriptional regulators	
		PA3697		hypothetical protein	-3.19	Transcriptional regulators	
		PA3699		probable transcriptional regulator	-2.10	Transcriptional regulators	
		PA3830		probable transcriptional regulator	2.19	Transcriptional regulators	
		PA3921		probable transcriptional regulator	-6.45	Transcriptional regulators	
		PA3927		probable transcriptional regulator	6.45	Transcriptional regulators	
		PA3965		probable transcriptional regulator	-2.50	Transcriptional regulators	
		PA4036		probable two-component sensor	-3.08	Two-component regulatory systems	
		PA4077		probable transcriptional regulator	-3.30	Transcriptional regulators	
		PA4094		probable transcriptional regulator	2.64	Transcriptional regulators	
		PA4120		probable transcriptional regulator	2.32	Transcriptional regulators	
		PA4135		probable transcriptional regulator	-3.60	Transcriptional regulators	
		PA4185		probable transcriptional regulator	-2.43	Transcriptional regulators	
		PA4341		probable transcriptional regulator	4.62	Transcriptional regulators	
		PA4354		conserved hypothetical protein	5.22	Transcriptional regulators	
		PA4398		two-component sensor	-2.90	Two-component regulatory systems	
		PA4436		probable transcriptional regulator	2.52	Transcriptional regulators	
		PA4769		probable transcriptional regulator	2.06	Transcriptional regulators	
		PA4806		probable transcriptional regulator	3.15	Transcriptional regulators	
		PA4982		probable two-component sensor	7.62	Two-component regulatory systems	
		PA4983		probable two-component response regulator	2.99	Two-component regulatory systems	Transcriptional regulators
		PA4984		probable transcriptional regulator	2.27	Transcriptional regulators	
		PA4989		probable transcriptional regulator	5.61	Transcriptional regulators	
		PA5029		probable transcriptional regulator	2.25	Transcriptional regulators	
		PA5032		probable transcriptional regulator	6.73	Transcriptional regulators	
		PA5059		probable transcriptional regulator	-41.67	Transcriptional regulators	
		PA5157		probable transcriptional regulator	2.58	Transcriptional regulators	
		PA5189		probable transcriptional regulator	2.34	Transcriptional regulators	
		PA5293		probable transcriptional regulator	3.83	Transcriptional regulators	
		PA5364		probable two-component response regulator	-2.24	Two-component regulatory systems	Transcriptional regulators
		PA5403		probable transcriptional regulator	2.67	Transcriptional regulators	
		PA5428		probable transcriptional regulator	2.06	Transcriptional regulators	
		PA5437		probable transcriptional regulator	3.25	Transcriptional regulators	
	Azoreductase	PA0785	*azoR1*	FMN-dependent NADH-azoreductase 1, AzoR1	2.62	Putative enzymes	
		PA1962	*azoR2*	FMN-dependent NADH-azoreductase 2, AzoR2	20.49	Putative enzymes	
		PA3223	*azoR3*	AzoR3, azoreductase 3	2.95	Fatty acid and phospholipid metabolism	
	Sulfatase	PA0740	*sdsA1*	SDS hydrolase SdsA1	26.34	Putative enzymes	
	Enoyl-coA hydratase	PA0745	*dspI*	DspI	-8.20	Putative enzymes	
	Cholinesterase	PA4921	*choE*	cholinesterase, ChoE	6.02	Hypothetical, unclassified, unknown	
		PA0117		probable short chain dehydrogenase	3.36	Putative enzymes	
		PA0142		hypothetical protein	2.02	Putative enzymes	
		PA0182		probable short-chain dehydrogenase	6.64	Putative enzymes	
		PA0190		probable acid phosphatase	2.44	Putative enzymes	
		PA0193		hypothetical protein	-2.65	Putative enzymes	
		PA0194		hypothetical protein	-2.03	Putative enzymes	
		PA0219		probable aldehyde dehydrogenase	4.15	Putative enzymes	
		PA0237		probable oxidoreductase	2.51	Putative enzymes	
		PA0318		conserved hypothetical protein	-2.08	Putative enzymes	
		PA0333		hypothetical protein	5.46	Putative enzymes	
		PA0473		probable glutathione S-transferase	-2.30	Putative enzymes	
		PA0483		probable acetyltransferase	-4.12	Putative enzymes	
		PA0489		probable phosphoribosyl transferase	27.39	Putative enzymes	
		PA0493		probable biotin-requiring enzyme	3.69	Putative enzymes	
		PA0506		probable acyl-CoA dehydrogenase	2.20	Putative enzymes	
		PA0508		probable acyl-CoA dehydrogenase	3.14	Putative enzymes	
		PA0531		probable glutamine amidotransferase	4.50	Putative enzymes	
		PA0545		hypothetical protein	7.63	Putative enzymes	
		PA0562		probable hydrolase	2.07	Putative enzymes	
		PA0656		probable HIT family protein	-4.41	Putative enzymes	
		PA0658		probable short-chain dehydrogenase	2.47	Putative enzymes	
		PA0730		probable transferase	-5.52	Putative enzymes	
		PA0744		probable enoyl-CoA hydratase/isomerase	-9.52	Putative enzymes	

**Putative enzymes**		PA0746		probable acyl-CoA dehydrogenase	-7.87	Putative enzymes
		PA0747		probable aldehyde dehydrogenase	-4.00	Putative enzymes
		PA0799		probable helicase	2.36	Putative enzymes
		PA0804		probable oxidoreductase	-4.93	Putative enzymes
		PA0817		probable ring-cleaving dioxygenase	-2.36	Putative enzymes
		PA0829		probable hydrolase	4.45	Putative enzymes
		PA0840		probable oxidoreductase	2.18	Putative enzymes
		PA0842		probable glycosyl transferase	33.31	Putative enzymes
		PA0863		probable oxidoreductase	-4.46	Putative enzymes
		PA0882		hypothetical protein	3.53	Putative enzymes
		PA0883		probable acyl-CoA lyase beta chain	3.23	Putative enzymes
		PA0975		probable radical activating enzyme	24.88	Putative enzymes
		PA1023		probable short-chain dehydrogenase	-4.78	Putative enzymes
		PA1033		probable glutathione S-transferase	-4.93	Putative enzymes
		PA1047		probable esterase	-4.65	Putative enzymes
		PA1062		hypothetical protein	-7.63	Putative enzymes
		PA1066		probable short-chain dehydrogenase	-2.14	Putative enzymes
		PA1202		probable hydrolase	-7.52	Putative enzymes
		PA1224		probable NAD(P)H dehydrogenase	3.40	Putative enzymes
		PA1240		probable enoyl-CoA hydratase/isomerase	-2.75	Putative enzymes
		PA1296		probable 2-hydroxyacid dehydrogenase	20.47	Putative enzymes
		PA1303		signal peptidase	-2.17	Putative enzymes
		PA1304		probable oligopeptidase	-2.40	Putative enzymes
		PA1306		probable HIT family protein	2.05	Putative enzymes
		PA1327		probable protease	-2.95	Putative enzymes
		PA1344		probable short-chain dehydrogenase	-4.61	Putative enzymes
		PA1379		probable short-chain dehydrogenase	3.14	Putative enzymes
		PA1470		probable short-chain dehydrogenase	3.62	Putative enzymes
		PA1538		probable flavin-containing monooxygenase	4.91	Putative enzymes
		PA1576		probable 3-hydroxyisobutyrate dehydrogenase	-2.10	Putative enzymes
		PA1617		probable AMP-binding enzyme	2.87	Putative enzymes
		PA1631		probable acyl-CoA dehydrogenase	3.27	Putative enzymes
		PA1648		probable oxidoreductase	-3.57	Putative enzymes
		PA1649		probable short-chain dehydrogenase	3.54	Putative enzymes
		PA1655		probable glutathione S-transferase	-2.72	Putative enzymes
		PA1739		probable oxidoreductase	6.68	Putative enzymes
		PA1771	*estX*	EstX	2.70	Putative enzymes
		PA1827		probable short-chain dehydrogenase	9.22	Putative enzymes
		PA1828		probable short-chain dehydrogenase	-5.81	Putative enzymes
		PA1829		hypothetical protein	-4.90	Putative enzymes
		PA1833		probable oxidoreductase	-2.68	Putative enzymes
		PA1880		probable oxidoreductase	-10.87	Putative enzymes
		PA1881		probable oxidoreductase	-6.25	Putative enzymes
		PA1890		probable glutathione S-transferase	-5.24	Putative enzymes
		PA1893		hypothetical protein	-12.20	Putative enzymes
		PA1914		conserved hypothetical protein	-333.33	Putative enzymes
		PA1918		hypothetical protein	3.18	Putative enzymes
		PA2024		probable ring-cleaving dioxygenase	-2.60	Putative enzymes
		PA2067		probable hydrolase	-25.64	Putative enzymes
		PA2069		probable carbamoyl transferase	-38.46	Putative enzymes
		PA2108		probable decarboxylase	11.04	Putative enzymes
		PA2119		alcohol dehydrogenase (Zn-dependent)	-7.30	Putative enzymes
		PA2124		probable dehydrogenase	2.54	Putative enzymes
		PA2188		probable alcohol dehydrogenase (Zn-dependent)	3.78	Putative enzymes
		PA2197		conserved hypothetical protein	-2.91	Putative enzymes
		PA2199		probable dehydrogenase	-2.91	Putative enzymes
		PA2324		hypothetical protein	2.62	Putative enzymes
	Putative enzymes	PA2326		hypothetical protein	4.60	Putative enzymes
		PA2346		conserved hypothetical protein	7.66	Putative enzymes
		PA2347		hypothetical protein	6.32	Putative enzymes
		PA2348		conserved hypothetical protein	9.49	Putative enzymes
		PA2355		probable FMNH2-dependent monooxygenase	4.66	Putative enzymes
		PA2419		probable hydrolase	-3.79	Putative enzymes
		PA2499		probable deaminase	-2.05	Putative enzymes
		PA2546		probable ring-cleaving dioxygenase	2.87	Putative enzymes
		PA2578		probable acetyltransferase	-2.34	Putative enzymes
		PA2631		probable acetyl transferase	-2.11	Putative enzymes
		PA2698		probable hydrolase	-2.07	Putative enzymes
		PA2745		probable hydrolase	19.93	Putative enzymes
		PA2767		probable enoyl-CoA hydratase/isomerase	6.17	Putative enzymes
		PA2841		probable enoyl-CoA hydratase/isomerase	-5.24	Putative enzymes
		PA2865		probable glycosylase	7.33	Putative enzymes
		PA2906		probable oxidoreductase	3.87	Putative enzymes
		PA2918		probable short-chain dehydrogenase	-2.18	Putative enzymes
		PA2922		probable hydrolase	3.37	Putative enzymes
		PA2974		probable hydrolase	3.87	Putative enzymes
		PA3053		probable hydrolytic enzyme	-2.27	Putative enzymes
		PA3128		probable short-chain dehydrogenase	-3.38	Putative enzymes
		PA3132		probable hydrolase	2.30	Putative enzymes
		PA3172		probable hydrolase	-4.31	Putative enzymes
		PA3173		probable short-chain dehydrogenase	-6.99	Putative enzymes
		PA3226		probable hydrolase	-2.48	Putative enzymes
		PA3256		probable oxidoreductase	-2.25	Putative enzymes
		PA3295		probable HIT family protein	-6.13	Putative enzymes
		PA3302		conserved hypothetical protein	-7.30	Putative enzymes
		PA3324		probable short-chain dehydrogenase	-2.26	Putative enzymes
		PA3328		probable FAD-dependent monooxygenase	-3.48	Putative enzymes
		PA3330		probable short chain dehydrogenase	-8.77	Putative enzymes
		PA3368		probable acetyltransferase	3.65	Putative enzymes
		PA3389		probable ring-cleaving dioxygenase	22.67	Putative enzymes
		PA3429		probable epoxide hydrolase	-2.15	Putative enzymes
		PA3460		probable acetyltransferase	-2.05	Putative enzymes
		PA3498		probable oxidoreductase	4.30	Putative enzymes
		PA3511		probable short-chain dehydrogenase	3.97	Putative enzymes
		PA3534		probable oxidoreductase	2.79	Putative enzymes
		PA3535		probable serine protease	-5.32	Putative enzymes
		PA3567		probable oxidoreductase	-3.95	Putative enzymes
		PA3586		probable hydrolase	6.48	Putative enzymes
		PA3667		probable pyridoxal-phosphate dependent enzyme	-2.99	Putative enzymes
		PA3723		probable FMN oxidoreductase	-12.82	Putative enzymes
		PA3798		probable aminotransferase	-2.23	Putative enzymes
		PA3817		probable methyltransferase	14.17	Putative enzymes
		PA3859		carboxylesterase	-3.09	Putative enzymes
		PA3860		probable AMP-binding enzyme	2.03	Putative enzymes
		PA3968		probable pseudouridine synthase	3.22	Putative enzymes
		PA3994		probable epoxide hydrolase	12.02	Putative enzymes
		PA4026		probable acetyltransferase	-5.29	Putative enzymes
		PA4041		hypothetical protein	2.66	Putative enzymes
		PA4079		NAD(P)H-dependent carbonyl reductase	-2.75	Putative enzymes
		PA4088		glutamate-1-semialdehyde aminomutase	2.72	Putative enzymes
		PA4098		probable short-chain dehydrogenase	4.63	Putative enzymes
		PA4167		probable oxidoreductase	8.50	Putative enzymes
		PA4189		probable aldehyde dehydrogenase	2.53	Putative enzymes
		PA4198		probable AMP-binding enzyme	2.90	Putative enzymes
		PA4199		probable acyl-CoA dehydrogenase	-3.21	Putative enzymes
		PA4344		probable hydrolase	6.53	Putative enzymes
		PA4401		probable glutathione S-transferase	-3.48	Putative enzymes
		PA4434		probable oxidoreductase	-4.74	Putative enzymes
		PA4576		probable ATP-dependent protease	-2.44	Putative enzymes
		PA4621		probable oxidoreductase	3.51	Putative enzymes
		PA4715		probable aminotransferase	-2.46	Putative enzymes
		PA4724		probable aminoacyl-transfer RNA synthetase (class I)	5.78	Putative enzymes
		PA4786		probable short-chain dehydrogenase	-2.10	Putative enzymes
		PA4819		probable glycosyl transferase	5.93	Putative enzymes
		PA4907		probable short-chain dehydrogenase	-7.09	Putative enzymes
		PA4943		probable GTP-binding protein	2.28	Putative enzymes
		PA4978		hypothetical protein	3.67	Putative enzymes
		PA4979		probable acyl-CoA dehydrogenase	2.53	Putative enzymes
		PA4986		probable oxidoreductase	2.29	Putative enzymes
		PA4995		probable acyl-CoA dehydrogenase	8.11	Putative enzymes
		PA5031		probable short chain dehydrogenase	10.05	Putative enzymes
		PA5048		probable nuclease	6.08	Putative enzymes
		PA5281		probable hydrolase	-3.07	Putative enzymes
		PA5432		probable acetyltransferase	2.26	Putative enzymes
		PA5445		probable coenzyme A transferase	7.73	Putative enzymes
		PA5523		probable aminotransferase	14.01	Putative enzymes
		PA5524		probable short-chain dehydrogenase	2.38	Putative enzymes
		PA5546		conserved hypothetical protein	-2.31	Putative enzymes
		PA0014		hypothetical protein	2.33	Hypothetical, unclassified, unknown
		PA0027		hypothetical protein	-7.52	Hypothetical, unclassified, unknown
		PA0028		hypothetical protein	-5.68	Hypothetical, unclassified, unknown
		PA0039		hypothetical protein	-4.00	Hypothetical, unclassified, unknown
		PA0042		hypothetical protein	2.42	Hypothetical, unclassified, unknown
		PA0045		hypothetical protein	2.40	Hypothetical, unclassified, unknown
		PA0046		hypothetical protein	2.58	Hypothetical, unclassified, unknown
		PA0047		hypothetical protein	3.04	Hypothetical, unclassified, unknown
		PA0049		hypothetical protein	-52.63	Hypothetical, unclassified, unknown
		PA0050		hypothetical protein	-10.31	Hypothetical, unclassified, unknown
		PA0052		hypothetical protein	-6.80	Hypothetical, unclassified, unknown
		PA0053		hypothetical protein	2.11	Hypothetical, unclassified, unknown
		PA0055		hypothetical protein	-5.75	Hypothetical, unclassified, unknown
		PA0057		hypothetical protein	4.56	Hypothetical, unclassified, unknown
		PA0062		hypothetical protein	2.12	Hypothetical, unclassified, unknown
		PA0063		hypothetical protein	3.09	Hypothetical, unclassified, unknown
		PA0068		hypothetical protein	-5.21	Hypothetical, unclassified, unknown
		PA0111		hypothetical protein	-5.24	Hypothetical, unclassified, unknown
		PA0115		conserved hypothetical protein	-2.20	Hypothetical, unclassified, unknown
		PA0128		conserved hypothetical protein	2.35	Hypothetical, unclassified, unknown
		PA0135		hypothetical protein	11.10	Hypothetical, unclassified, unknown
		PA0145		hypothetical protein	2.43	Hypothetical, unclassified, unknown
		PA0146		conserved hypothetical protein	11.97	Hypothetical, unclassified, unknown
		PA0160		hypothetical protein	2.21	Hypothetical, unclassified, unknown
		PA0161		hypothetical protein	4.58	Hypothetical, unclassified, unknown
		PA0165		hypothetical protein	5.26	Hypothetical, unclassified, unknown
		PA0168		conserved hypothetical protein	-3.56	Hypothetical, unclassified, unknown
		PA0187		hypothetical protein	-2.19	Hypothetical, unclassified, unknown
		PA0200		hypothetical protein	-3.82	Hypothetical, unclassified, unknown
		PA0201		hypothetical protein	4.45	Hypothetical, unclassified, unknown
		PA0222		hypothetical protein	2.60	Hypothetical, unclassified, unknown
		PA0234		hypothetical protein	2.58	Hypothetical, unclassified, unknown
		PA0250		conserved hypothetical protein	-2.74	Hypothetical, unclassified, unknown
		PA0251		hypothetical protein	7.02	Hypothetical, unclassified, unknown
		PA0252		hypothetical protein	9.99	Hypothetical, unclassified, unknown
		PA0264		hypothetical protein	14.99	Hypothetical, unclassified, unknown
		PA0270		hypothetical protein	-2.39	Hypothetical, unclassified, unknown
		PA0271		hypothetical protein	-2.06	Hypothetical, unclassified, unknown
		PA0277		conserved hypothetical protein	9.79	Hypothetical, unclassified, unknown
		PA0284		hypothetical protein	2.28	Hypothetical, unclassified, unknown
		PA0307		hypothetical protein	2.60	Hypothetical, unclassified, unknown
		PA0309		hypothetical protein	2.66	Hypothetical, unclassified, unknown
		PA0310		hypothetical protein	4.07	Hypothetical, unclassified, unknown
		PA0311		hypothetical protein	3.91	Hypothetical, unclassified, unknown
		PA0332		hypothetical protein	2.02	Hypothetical, unclassified, unknown
		PA0335		hypothetical protein	-3.28	Hypothetical, unclassified, unknown
		PA0346		hypothetical protein	6.25	Hypothetical, unclassified, unknown
		PA0348		hypothetical protein	9.33	Hypothetical, unclassified, unknown
		PA0351		conserved hypothetical protein	2.57	Hypothetical, unclassified, unknown
		PA0358		hypothetical protein	8.24	Hypothetical, unclassified, unknown
		PA0369		Uncharacterized protein	-4.69	Hypothetical, unclassified, unknown
		PA0370		conserved hypothetical protein	-5.88	Hypothetical, unclassified, unknown
		PA0371		hypothetical protein	-2.87	Hypothetical, unclassified, unknown
		PA0377		hypothetical protein	2.01	Hypothetical, unclassified, unknown
		PA0384		hypothetical protein	-3.91	Hypothetical, unclassified, unknown
		PA0385		hypothetical protein	-2.00	Hypothetical, unclassified, unknown
		PA0388		hypothetical protein	-9.09	Hypothetical, unclassified, unknown
		PA0389		hypothetical protein	-5.15	Hypothetical, unclassified, unknown
		PA0391		hypothetical protein	-6.99	Hypothetical, unclassified, unknown
		PA0392		conserved hypothetical protein	-3.18	Hypothetical, unclassified, unknown
		PA0398		hypothetical protein	-2.13	Hypothetical, unclassified, unknown
		PA0404		conserved hypothetical protein	-2.08	Hypothetical, unclassified, unknown
		PA0429		hypothetical protein	-5.21	Hypothetical, unclassified, unknown
		PA0431		hypothetical protein	-3.17	Hypothetical, unclassified, unknown
		PA0453		hypothetical protein	8.08	Hypothetical, unclassified, unknown
		PA0457.1		hypothetical membrane protein	-2.14	Hypothetical, unclassified, unknown
		PA0462		hypothetical protein	5.93	Hypothetical, unclassified, unknown
		PA0466		hypothetical protein	5.14	Hypothetical, unclassified, unknown
		PA0467		conserved hypothetical protein	-3.73	Hypothetical, unclassified, unknown
		PA0468		hypothetical protein	-5.75	Hypothetical, unclassified, unknown
		PA0469		hypothetical protein	2.78	Hypothetical, unclassified, unknown
		PA0474		hypothetical protein	3.72	Hypothetical, unclassified, unknown
		PA0484		conserved hypothetical protein	-7.30	Hypothetical, unclassified, unknown
		PA0488		conserved hypothetical protein	5.95	Hypothetical, unclassified, unknown
		PA0490		hypothetical protein	3.54	Hypothetical, unclassified, unknown
		PA0492		conserved hypothetical protein	5.40	Hypothetical, unclassified, unknown
		PA0497		hypothetical protein	2.83	Hypothetical, unclassified, unknown
		PA0526		hypothetical protein	-6.54	Hypothetical, unclassified, unknown
		PA0529		conserved hypothetical protein	3.15	Hypothetical, unclassified, unknown
		PA0532		hypothetical protein	10.60	Hypothetical, unclassified, unknown
		PA0542		conserved hypothetical protein	-2.02	Hypothetical, unclassified, unknown
		PA0543		hypothetical protein	5.91	Hypothetical, unclassified, unknown
		PA0549		hypothetical protein	-2.46	Hypothetical, unclassified, unknown
		PA0550		conserved hypothetical protein	-5.43	Hypothetical, unclassified, unknown
		PA0563		conserved hypothetical protein	2.09	Hypothetical, unclassified, unknown
		PA0565		conserved hypothetical protein	-6.45	Hypothetical, unclassified, unknown
		PA0568		hypothetical protein	-2.97	Hypothetical, unclassified, unknown
		PA0569		hypothetical protein	-3.70	Hypothetical, unclassified, unknown
		PA0570		hypothetical protein	-2.19	Hypothetical, unclassified, unknown
		PA0571		hypothetical protein	-3.52	Hypothetical, unclassified, unknown
		PA0573		hypothetical protein	4.37	Hypothetical, unclassified, unknown
		PA0574		hypothetical protein	2.04	Hypothetical, unclassified, unknown
		PA0578		conserved hypothetical protein	8.74	Hypothetical, unclassified, unknown
		PA0585		hypothetical protein	-2.26	Hypothetical, unclassified, unknown
		PA0586		conserved hypothetical protein	-28.57	Hypothetical, unclassified, unknown
		PA0587		conserved hypothetical protein	-19.23	Hypothetical, unclassified, unknown
		PA0588		conserved hypothetical protein	-10.87	Hypothetical, unclassified, unknown
		PA0596		hypothetical protein	2.96	Hypothetical, unclassified, unknown
		PA0599		hypothetical protein	-2.19	Hypothetical, unclassified, unknown
		PA0665		conserved hypothetical protein	2.55	Hypothetical, unclassified, unknown
		PA0670		hypothetical protein	2.23	Hypothetical, unclassified, unknown
		PA0671		hypothetical protein	4.29	Hypothetical, unclassified, unknown
		PA0673		hypothetical protein	-3.46	Hypothetical, unclassified, unknown
		PA0695		hypothetical protein	4.88	Hypothetical, unclassified, unknown
		PA0711		hypothetical protein	7.49	Hypothetical, unclassified, unknown
		PA0712		hypothetical protein	4.95	Hypothetical, unclassified, unknown
		PA0713		hypothetical protein	-4.37	Hypothetical, unclassified, unknown
		PA0731		hypothetical protein	-4.74	Hypothetical, unclassified, unknown
		PA0732		hypothetical protein	-2.25	Hypothetical, unclassified, unknown
		PA0735		hypothetical protein	-2.58	Hypothetical, unclassified, unknown
		PA0737		hypothetical protein	11.17	Hypothetical, unclassified, unknown
		PA0741		conserved hypothetical protein	-2.30	Hypothetical, unclassified, unknown
		PA0749		hypothetical protein	2.33	Hypothetical, unclassified, unknown
		PA0754		hypothetical protein	-2.03	Hypothetical, unclassified, unknown
		PA0758		hypothetical protein	-3.25	Hypothetical, unclassified, unknown
		PA0759		conserved hypothetical protein	-2.74	Hypothetical, unclassified, unknown
		PA0760		conserved hypothetical protein	2.14	Hypothetical, unclassified, unknown
		PA0775		conserved hypothetical protein	2.46	Hypothetical, unclassified, unknown
		PA0776		hypothetical protein	-4.13	Hypothetical, unclassified, unknown
		PA0778	*icp*	inhibitor of cysteine peptidase	-3.10	Hypothetical, unclassified, unknown
		PA0788		hypothetical protein	-2.68	Hypothetical, unclassified, unknown
		PA0793		hypothetical protein	-5.62	Hypothetical, unclassified, unknown
		PA0803		hypothetical protein	-5.00	Hypothetical, unclassified, unknown
		PA0805		hypothetical protein	46.99	Hypothetical, unclassified, unknown
		PA0808		hypothetical protein	-3.51	Hypothetical, unclassified, unknown
		PA0812		hypothetical protein	2.52	Hypothetical, unclassified, unknown
		PA0818		hypothetical protein	2.60	Hypothetical, unclassified, unknown
		PA0820		hypothetical protein	-2.13	Hypothetical, unclassified, unknown
		PA0821		hypothetical protein	-2.61	Hypothetical, unclassified, unknown
		PA0823		hypothetical protein	6.93	Hypothetical, unclassified, unknown
		PA0824		hypothetical protein	5.51	Hypothetical, unclassified, unknown
		PA0825		hypothetical protein	3.02	Hypothetical, unclassified, unknown
		PA0832		conserved hypothetical protein	-2.24	Hypothetical, unclassified, unknown
		PA0850		hypothetical protein	-2.58	Hypothetical, unclassified, unknown
		PA0856		hypothetical protein	-2.29	Hypothetical, unclassified, unknown
		PA0858		conserved hypothetical protein	2.38	Hypothetical, unclassified, unknown
		PA0862		hypothetical protein	-4.10	Hypothetical, unclassified, unknown
		PA0874		hypothetical protein	17.72	Hypothetical, unclassified, unknown
		PA0878		hypothetical protein	5.67	Hypothetical, unclassified, unknown
		PA0881		hypothetical protein	2.03	Hypothetical, unclassified, unknown
		PA0891		hypothetical protein	-5.00	Hypothetical, unclassified, unknown
		PA0894		hypothetical protein	2.40	Hypothetical, unclassified, unknown
		PA0900		hypothetical protein	-5.00	Hypothetical, unclassified, unknown
		PA0912		hypothetical protein	2.95	Hypothetical, unclassified, unknown
		PA0914		hypothetical protein	2.62	Hypothetical, unclassified, unknown
		PA0919		alanyl-phosphatidylglycerol hydrolase	-4.05	Hypothetical, unclassified, unknown
		PA0922		hypothetical protein	11.12	Hypothetical, unclassified, unknown
		PA0926		hypothetical protein	-2.54	Hypothetical, unclassified, unknown
		PA0939		hypothetical protein	4.36	Hypothetical, unclassified, unknown

PA0941		hypothetical protein	2.42	Hypothetical, unclassified, unknown
PA0946		hypothetical protein	-2.33	Hypothetical, unclassified, unknown
PA0947		conserved hypothetical protein	3.07	Hypothetical, unclassified, unknown
PA0955		hypothetical protein	-3.18	Hypothetical, unclassified, unknown
PA0957		hypothetical protein	2.61	Hypothetical, unclassified, unknown
PA0976		conserved hypothetical protein	10.75	Hypothetical, unclassified, unknown
PA0977		hypothetical protein	5.29	Hypothetical, unclassified, unknown
PA0980		hypothetical protein	32.21	Hypothetical, unclassified, unknown
PA0981		hypothetical protein	4.36	Hypothetical, unclassified, unknown
PA0983		conserved hypothetical protein	2.14	Hypothetical, unclassified, unknown
PA0988		hypothetical protein	-2.43	Hypothetical, unclassified, unknown
PA0990		conserved hypothetical protein	3.98	Hypothetical, unclassified, unknown
PA1005		conserved hypothetical protein	-4.61	Hypothetical, unclassified, unknown
PA1006		Protein PA1006	6.69	Hypothetical, unclassified, unknown
PA1009		hypothetical protein	-4.22	Hypothetical, unclassified, unknown
PA1011		hypothetical protein	-3.29	Hypothetical, unclassified, unknown
PA1016		hypothetical protein	9.62	Hypothetical, unclassified, unknown
PA1018		hypothetical protein	8.05	Hypothetical, unclassified, unknown
PA1024		NADH:quinone reductase	-4.27	Hypothetical, unclassified, unknown
PA1029		hypothetical protein	8.62	Hypothetical, unclassified, unknown
PA1030		hypothetical protein	9.87	Hypothetical, unclassified, unknown
PA1031		conserved hypothetical protein	6.73	Hypothetical, unclassified, unknown
PA1034		hypothetical protein	-2.48	Hypothetical, unclassified, unknown
PA1035		hypothetical protein	-2.32	Hypothetical, unclassified, unknown
PA1037		conserved hypothetical protein	2.81	Hypothetical, unclassified, unknown
PA1038		hypothetical protein	-5.38	Hypothetical, unclassified, unknown
PA1039		conserved hypothetical protein	-5.62	Hypothetical, unclassified, unknown
PA1040		hypothetical protein	-3.51	Hypothetical, unclassified, unknown
PA1050		conserved hypothetical protein	-3.29	Hypothetical, unclassified, unknown
PA1063		hypothetical protein	-6.41	Hypothetical, unclassified, unknown
PA1064		hypothetical protein	-2.39	Hypothetical, unclassified, unknown
PA1076		hypothetical protein	-3.45	Hypothetical, unclassified, unknown
PA1088		hypothetical protein	-3.56	Hypothetical, unclassified, unknown
PA1089		conserved hypothetical protein	-6.67	Hypothetical, unclassified, unknown
PA1090		hypothetical protein	-5.03	Hypothetical, unclassified, unknown
PA1106		hypothetical protein	-2.56	Hypothetical, unclassified, unknown
PA1111		hypothetical protein	12.19	Hypothetical, unclassified, unknown
PA1114		hypothetical protein	6.07	Hypothetical, unclassified, unknown
PA1116		hypothetical protein	3.68	Hypothetical, unclassified, unknown
PA1123		hypothetical protein	-6.99	Hypothetical, unclassified, unknown
PA1132		hypothetical protein	6.28	Hypothetical, unclassified, unknown
PA1134		hypothetical protein	-4.63	Hypothetical, unclassified, unknown
PA1135		conserved hypothetical protein	-7.41	Hypothetical, unclassified, unknown
PA1139		hypothetical protein	6.63	Hypothetical, unclassified, unknown
PA1152		hypothetical protein	2.61	Hypothetical, unclassified, unknown
PA1154		conserved hypothetical protein	3.98	Hypothetical, unclassified, unknown
PA1160		hypothetical protein	-3.06	Hypothetical, unclassified, unknown
PA1164		conserved hypothetical protein	6.73	Hypothetical, unclassified, unknown
PA1166		hypothetical protein	-2.36	Hypothetical, unclassified, unknown
PA1167		hypothetical protein	-5.38	Hypothetical, unclassified, unknown
PA1168		hypothetical protein	-2.10	Hypothetical, unclassified, unknown
PA1203		hypothetical protein	-3.80	Hypothetical, unclassified, unknown
PA1205		conserved hypothetical protein	-4.83	Hypothetical, unclassified, unknown
PA1206		hypothetical protein	2.36	Hypothetical, unclassified, unknown
PA1208		conserved hypothetical protein	2.03	Hypothetical, unclassified, unknown
PA1211		hypothetical protein	-7.69	Hypothetical, unclassified, unknown
PA1213		hypothetical protein	-29.41	Hypothetical, unclassified, unknown
PA1214		hypothetical protein	-15.38	Hypothetical, unclassified, unknown
PA1215		hypothetical protein	-20.00	Hypothetical, unclassified, unknown
PA1216		hypothetical protein	-41.67	Hypothetical, unclassified, unknown
PA1218		hypothetical protein	-34.48	Hypothetical, unclassified, unknown
PA1219		hypothetical protein	-7.94	Hypothetical, unclassified, unknown
PA1220		hypothetical protein	-8.70	Hypothetical, unclassified, unknown
PA1221		hypothetical protein	-7.69	Hypothetical, unclassified, unknown
PA1228		hypothetical protein	6.22	Hypothetical, unclassified, unknown
PA1234		hypothetical protein	-2.13	Hypothetical, unclassified, unknown
PA1263		hypothetical protein	2.72	Hypothetical, unclassified, unknown
PA1270		hypothetical protein	11.27	Hypothetical, unclassified, unknown
PA1291		hypothetical protein	7.42	Hypothetical, unclassified, unknown
PA1293		hypothetical protein	-3.83	Hypothetical, unclassified, unknown
PA1298		conserved hypothetical protein	16.32	Hypothetical, unclassified, unknown
PA1299		conserved hypothetical protein	13.00	Hypothetical, unclassified, unknown
PA1323		hypothetical protein	-4.42	Hypothetical, unclassified, unknown
PA1324		hypothetical protein	-6.67	Hypothetical, unclassified, unknown
PA1325		conserved hypothetical protein	2.23	Hypothetical, unclassified, unknown
PA1329		conserved hypothetical protein	6.57	Hypothetical, unclassified, unknown
PA1332		hypothetical protein	35.07	Hypothetical, unclassified, unknown
PA1333		hypothetical protein	15.90	Hypothetical, unclassified, unknown
PA1343		hypothetical protein	4.36	Hypothetical, unclassified, unknown
PA1353		hypothetical protein	-4.72	Hypothetical, unclassified, unknown
PA1354		hypothetical protein	-2.15	Hypothetical, unclassified, unknown
PA1356		hypothetical protein	-3.69	Hypothetical, unclassified, unknown
PA1357		conserved hypothetical protein	-2.99	Hypothetical, unclassified, unknown
PA1362		hypothetical protein	-3.11	Hypothetical, unclassified, unknown
PA1369		hypothetical protein	-3.10	Hypothetical, unclassified, unknown
PA1370		hypothetical protein	-2.50	Hypothetical, unclassified, unknown
PA1371		hypothetical protein	-7.94	Hypothetical, unclassified, unknown
PA1372		hypothetical protein	-5.03	Hypothetical, unclassified, unknown
PA1374		hypothetical protein	7.48	Hypothetical, unclassified, unknown
PA1381		hypothetical protein	3.45	Hypothetical, unclassified, unknown
PA1394		hypothetical protein	5.92	Hypothetical, unclassified, unknown
PA1395		hypothetical protein	8.79	Hypothetical, unclassified, unknown
PA1401		hypothetical protein	-2.11	Hypothetical, unclassified, unknown
PA1412		hypothetical protein	4.06	Hypothetical, unclassified, unknown
PA1414		hypothetical protein	-2.15	Hypothetical, unclassified, unknown
PA1415		hypothetical protein	-3.18	Hypothetical, unclassified, unknown
PA1420		hypothetical protein	3.27	Hypothetical, unclassified, unknown
PA1424		hypothetical protein	6.28	Hypothetical, unclassified, unknown
PA1426		hypothetical protein	5.32	Hypothetical, unclassified, unknown
PA1427		hypothetical protein	13.51	Hypothetical, unclassified, unknown
PA1428		conserved hypothetical protein	3.98	Hypothetical, unclassified, unknown
PA1439		conserved hypothetical protein	6.31	Hypothetical, unclassified, unknown
PA1465		hypothetical protein	2.40	Hypothetical, unclassified, unknown
PA1466		hypothetical protein	2.05	Hypothetical, unclassified, unknown
PA1467		hypothetical protein	14.05	Hypothetical, unclassified, unknown
PA1468		hypothetical protein	18.92	Hypothetical, unclassified, unknown
PA1469		hypothetical protein	5.49	Hypothetical, unclassified, unknown
PA1471		hypothetical protein	2.26	Hypothetical, unclassified, unknown
PA1472		conserved hypothetical protein	6.33	Hypothetical, unclassified, unknown
PA1473		hypothetical protein	6.42	Hypothetical, unclassified, unknown
PA1474		hypothetical protein	9.84	Hypothetical, unclassified, unknown
PA1489		hypothetical protein	2.00	Hypothetical, unclassified, unknown
PA1495		hypothetical protein	-3.82	Hypothetical, unclassified, unknown
PA1503		hypothetical protein	3.59	Hypothetical, unclassified, unknown
PA1516		hypothetical protein	-2.16	Hypothetical, unclassified, unknown
PA1531		hypothetical protein	-3.72	Hypothetical, unclassified, unknown
PA1539		hypothetical protein	3.48	Hypothetical, unclassified, unknown
PA1542		hypothetical protein	6.52	Hypothetical, unclassified, unknown
PA1545		hypothetical protein	6.33	Hypothetical, unclassified, unknown
PA1558		hypothetical protein	3.51	Hypothetical, unclassified, unknown
PA1559		hypothetical protein	4.00	Hypothetical, unclassified, unknown
PA1560		hypothetical protein	2.66	Hypothetical, unclassified, unknown
PA1567		conserved hypothetical protein	2.30	Hypothetical, unclassified, unknown
PA1571		hypothetical protein	-3.98	Hypothetical, unclassified, unknown
PA1575		hypothetical protein	-3.13	Hypothetical, unclassified, unknown
PA1592		hypothetical protein	2.24	Hypothetical, unclassified, unknown
PA1605		hypothetical protein	3.56	Hypothetical, unclassified, unknown
PA1607		conserved hypothetical protein	2.45	Hypothetical, unclassified, unknown
PA1623		conserved hypothetical protein	-4.10	Hypothetical, unclassified, unknown
PA1624		hypothetical protein	-5.32	Hypothetical, unclassified, unknown
PA1641		hypothetical protein	3.20	Hypothetical, unclassified, unknown
PA1672		hypothetical protein	2.21	Hypothetical, unclassified, unknown
PA1675		conserved hypothetical protein	5.79	Hypothetical, unclassified, unknown
PA1679		hypothetical protein	7.67	Hypothetical, unclassified, unknown
PA1680		hypothetical protein	9.55	Hypothetical, unclassified, unknown
PA1689		conserved hypothetical protein	-3.48	Hypothetical, unclassified, unknown
PA1728		hypothetical protein	-2.19	Hypothetical, unclassified, unknown
PA1732		conserved hypothetical protein	-6.33	Hypothetical, unclassified, unknown
PA1733		conserved hypothetical protein	-9.90	Hypothetical, unclassified, unknown
PA1734		hypothetical protein	-2.32	Hypothetical, unclassified, unknown
PA1740		hypothetical protein	3.67	Hypothetical, unclassified, unknown
PA1743		hypothetical protein	4.28	Hypothetical, unclassified, unknown
PA1745		hypothetical protein	-3.10	Hypothetical, unclassified, unknown
PA1746		hypothetical protein	-18.52	Hypothetical, unclassified, unknown
PA1762		hypothetical protein	-2.16	Hypothetical, unclassified, unknown
PA1764		hypothetical protein	4.78	Hypothetical, unclassified, unknown
PA1766		hypothetical protein	-3.11	Hypothetical, unclassified, unknown
PA1769		conserved hypothetical protein	-3.17	Hypothetical, unclassified, unknown
PA1774	*crfX*	CrfX protein	8.87	Hypothetical, unclassified, unknown
PA1784		hypothetical protein	-29.41	Hypothetical, unclassified, unknown
PA1789		hypothetical protein	-8.00	Hypothetical, unclassified, unknown
PA1790		hypothetical protein	3.99	Hypothetical, unclassified, unknown
PA1791		hypothetical protein	2.30	Hypothetical, unclassified, unknown
PA1817		hypothetical protein	-2.18	Hypothetical, unclassified, unknown
PA1830		hypothetical protein	-4.98	Hypothetical, unclassified, unknown
PA1835		hypothetical protein	-2.09	Hypothetical, unclassified, unknown
PA1837		hypothetical protein	-5.92	Hypothetical, unclassified, unknown
PA1839		hypothetical protein	4.69	Hypothetical, unclassified, unknown
PA1842		hypothetical protein	-9.71	Hypothetical, unclassified, unknown
PA1849		conserved hypothetical protein	4.44	Hypothetical, unclassified, unknown
PA1852		hypothetical protein	2.12	Hypothetical, unclassified, unknown
PA1854		conserved hypothetical protein	4.55	Hypothetical, unclassified, unknown
PA1855		hypothetical protein	4.45	Hypothetical, unclassified, unknown
PA1865		hypothetical protein	2.48	Hypothetical, unclassified, unknown
PA1866		hypothetical protein	4.01	Hypothetical, unclassified, unknown
PA1870		hypothetical protein	-2.02	Hypothetical, unclassified, unknown
PA1872		hypothetical protein	-2.56	Hypothetical, unclassified, unknown
PA1887		hypothetical protein	-24.39	Hypothetical, unclassified, unknown
PA1888		hypothetical protein	-12.82	Hypothetical, unclassified, unknown
PA1889		hypothetical protein	9.07	Hypothetical, unclassified, unknown
PA1892		hypothetical protein	-11.24	Hypothetical, unclassified, unknown
PA1894		hypothetical protein	-16.95	Hypothetical, unclassified, unknown
PA1896		hypothetical protein	-11.63	Hypothetical, unclassified, unknown
PA1907		hypothetical protein	3.06	Hypothetical, unclassified, unknown
PA1909		hypothetical protein	-3.72	Hypothetical, unclassified, unknown
PA1915		hypothetical protein	-2.22	Hypothetical, unclassified, unknown
PA1921		hypothetical protein	2.98	Hypothetical, unclassified, unknown
PA1929		hypothetical protein	3.27	Hypothetical, unclassified, unknown
PA1935		hypothetical protein	2.23	Hypothetical, unclassified, unknown
PA1936		hypothetical protein	2.71	Hypothetical, unclassified, unknown
PA1939		hypothetical protein	-8.00	Hypothetical, unclassified, unknown
PA1940		hypothetical protein	-7.75	Hypothetical, unclassified, unknown
PA1942		hypothetical protein	18.51	Hypothetical, unclassified, unknown
PA1943		hypothetical protein	-2.53	Hypothetical, unclassified, unknown
PA1965		hypothetical protein	2.43	Hypothetical, unclassified, unknown
PA1967		hypothetical protein	-6.90	Hypothetical, unclassified, unknown
PA1970		hypothetical protein	6.56	Hypothetical, unclassified, unknown
PA1974		hypothetical protein	7.94	Hypothetical, unclassified, unknown
PA1975		hypothetical protein	4.82	Hypothetical, unclassified, unknown
PA1981		hypothetical protein	2.57	Hypothetical, unclassified, unknown
PA1994		hypothetical protein	-2.76	Hypothetical, unclassified, unknown
PA1995		hypothetical protein	-2.36	Hypothetical, unclassified, unknown
PA2017		hypothetical protein	4.59	Hypothetical, unclassified, unknown
PA2021		hypothetical protein	2.25	Hypothetical, unclassified, unknown
PA2029		hypothetical protein	-2.72	Hypothetical, unclassified, unknown
PA2030		hypothetical protein	-10.64	Hypothetical, unclassified, unknown
PA2031		hypothetical protein	-8.47	Hypothetical, unclassified, unknown
PA2033		hypothetical protein	-2.23	Hypothetical, unclassified, unknown
PA2034		hypothetical protein	-2.28	Hypothetical, unclassified, unknown
PA2036		hypothetical protein	2.66	Hypothetical, unclassified, unknown
PA2037		hypothetical protein	2.36	Hypothetical, unclassified, unknown
PA2038		hypothetical protein	-5.59	Hypothetical, unclassified, unknown
PA2044		hypothetical protein	-5.03	Hypothetical, unclassified, unknown
PA2046		hypothetical protein	3.61	Hypothetical, unclassified, unknown
PA2049		hypothetical protein	-2.79	Hypothetical, unclassified, unknown
PA2066		hypothetical protein	-21.28	Hypothetical, unclassified, unknown
PA2087		hypothetical protein	2.44	Hypothetical, unclassified, unknown
PA2106		hypothetical protein	6.20	Hypothetical, unclassified, unknown
PA2107		hypothetical protein	2.95	Hypothetical, unclassified, unknown
PA2109		hypothetical protein	-18.87	Hypothetical, unclassified, unknown
PA2110		hypothetical protein	-18.87	Hypothetical, unclassified, unknown
PA2111		hypothetical protein	-16.39	Hypothetical, unclassified, unknown
PA2112		conserved hypothetical protein	-23.26	Hypothetical, unclassified, unknown
PA2116		conserved hypothetical protein	-29.41	Hypothetical, unclassified, unknown
PA2122		hypothetical protein	-3.25	Hypothetical, unclassified, unknown
PA2136		hypothetical protein	8.34	Hypothetical, unclassified, unknown
PA2137		hypothetical protein	6.64	Hypothetical, unclassified, unknown
PA2139		hypothetical protein	8.31	Hypothetical, unclassified, unknown
PA2141		hypothetical protein	2.36	Hypothetical, unclassified, unknown
PA2145		hypothetical protein	5.71	Hypothetical, unclassified, unknown
PA2150		conserved hypothetical protein	2.12	Hypothetical, unclassified, unknown
PA2156		conserved hypothetical protein	2.05	Hypothetical, unclassified, unknown
PA2161		hypothetical protein	-5.99	Hypothetical, unclassified, unknown
PA2166		hypothetical protein	-2.68	Hypothetical, unclassified, unknown
PA2171		hypothetical protein	-2.26	Hypothetical, unclassified, unknown
PA2173		hypothetical protein	-2.50	Hypothetical, unclassified, unknown
PA2174		hypothetical protein	-3.98	Hypothetical, unclassified, unknown
PA2181		hypothetical protein	14.88	Hypothetical, unclassified, unknown
PA2186		hypothetical protein	5.43	Hypothetical, unclassified, unknown
PA2187		hypothetical protein	2.77	Hypothetical, unclassified, unknown
PA2189		hypothetical protein	4.81	Hypothetical, unclassified, unknown
PA2198		hypothetical protein	-2.26	Hypothetical, unclassified, unknown
PA2208		hypothetical protein	3.12	Hypothetical, unclassified, unknown
PA2209		hypothetical protein	5.80	Hypothetical, unclassified, unknown
PA2211		conserved hypothetical protein	7.21	Hypothetical, unclassified, unknown
PA2222		hypothetical protein	-9.90	Hypothetical, unclassified, unknown
PA2223		hypothetical protein	-11.90	Hypothetical, unclassified, unknown
PA2224		hypothetical protein	-2.40	Hypothetical, unclassified, unknown
PA2225		hypothetical protein	-3.36	Hypothetical, unclassified, unknown
PA2229		conserved hypothetical protein	-2.58	Hypothetical, unclassified, unknown
PA2251		hypothetical protein	3.63	Hypothetical, unclassified, unknown
PA2282		hypothetical protein	2.09	Hypothetical, unclassified, unknown
PA2283		hypothetical protein	5.65	Hypothetical, unclassified, unknown
PA2284		hypothetical protein	3.70	Hypothetical, unclassified, unknown
PA2285		hypothetical protein	-2.13	Hypothetical, unclassified, unknown
PA2287		hypothetical protein	2.87	Hypothetical, unclassified, unknown
PA2288		hypothetical protein	24.81	Hypothetical, unclassified, unknown
PA2289		conserved hypothetical protein	-6.41	Hypothetical, unclassified, unknown
PA2301		hypothetical protein	-10.42	Hypothetical, unclassified, unknown
PA2309		hypothetical protein	3.81	Hypothetical, unclassified, unknown
PA2311		hypothetical protein	9.55	Hypothetical, unclassified, unknown
PA2315		hypothetical protein	15.99	Hypothetical, unclassified, unknown
PA2318		hypothetical protein	-5.10	Hypothetical, unclassified, unknown
PA2328		hypothetical protein	-13.16	Hypothetical, unclassified, unknown
PA2330		hypothetical protein	-10.42	Hypothetical, unclassified, unknown
PA2377		hypothetical protein	7.68	Hypothetical, unclassified, unknown
PA2381		hypothetical protein	-37.04	Hypothetical, unclassified, unknown
PA2384		hypothetical protein	-4.13	Hypothetical, unclassified, unknown
PA2422		hypothetical protein	-2.62	Hypothetical, unclassified, unknown
PA2423		hypothetical protein	-4.52	Hypothetical, unclassified, unknown
PA2427		hypothetical protein	-6.13	Hypothetical, unclassified, unknown
PA2429		hypothetical protein	2.76	Hypothetical, unclassified, unknown
PA2430		conserved hypothetical protein	-2.81	Hypothetical, unclassified, unknown
PA2434		hypothetical protein	2.38	Hypothetical, unclassified, unknown
PA2440		hypothetical protein	3.57	Hypothetical, unclassified, unknown
PA2441		hypothetical protein	2.13	Hypothetical, unclassified, unknown
PA2448		hypothetical protein	-10.87	Hypothetical, unclassified, unknown
PA2450		hypothetical protein	4.15	Hypothetical, unclassified, unknown
PA2455		hypothetical protein	-2.44	Hypothetical, unclassified, unknown
PA2456		hypothetical protein	-3.21	Hypothetical, unclassified, unknown
PA2457		hypothetical protein	2.03	Hypothetical, unclassified, unknown
PA2460		hypothetical protein	2.63	Hypothetical, unclassified, unknown
PA2462		hypothetical protein	2.62	Hypothetical, unclassified, unknown
PA2463		hypothetical protein	7.31	Hypothetical, unclassified, unknown
PA2481		hypothetical protein	5.29	Hypothetical, unclassified, unknown
PA2485		hypothetical protein	13.74	Hypothetical, unclassified, unknown
PA2487		hypothetical protein	4.40	Hypothetical, unclassified, unknown
PA2496		hypothetical protein	2.22	Hypothetical, unclassified, unknown
PA2503		hypothetical protein	2.99	Hypothetical, unclassified, unknown
PA2504		hypothetical protein	-8.26	Hypothetical, unclassified, unknown
PA2506		hypothetical protein	4.78	Hypothetical, unclassified, unknown
PA2529		hypothetical protein	-3.91	Hypothetical, unclassified, unknown
PA2530		hypothetical protein	-5.35	Hypothetical, unclassified, unknown
PA2540		conserved hypothetical protein	2.18	Hypothetical, unclassified, unknown
PA2542		conserved hypothetical protein	2.50	Hypothetical, unclassified, unknown
PA2543		conserved hypothetical protein	2.17	Hypothetical, unclassified, unknown
PA2544		hypothetical protein	-2.85	Hypothetical, unclassified, unknown

Unknow, hypothetical protein

Hypothetical proteins

PA2548	hypothetical protein	3.03	Hypothetical, unclassified, unknown
PA2559	hypothetical protein	2.07	Hypothetical, unclassified, unknown
PA2560	hypothetical protein	3.69	Hypothetical, unclassified, unknown
PA2564	hypothetical protein	-2.66	Hypothetical, unclassified, unknown
PA2565	hypothetical protein	-2.52	Hypothetical, unclassified, unknown
PA2569	hypothetical protein	12.97	Hypothetical, unclassified, unknown
PA2575	hypothetical protein	-8.20	Hypothetical, unclassified, unknown
PA2590	hypothetical protein	20.07	Hypothetical, unclassified, unknown
PA2599	conserved hypothetical protein	4.99	Hypothetical, unclassified, unknown
PA2604	conserved hypothetical protein	-3.28	Hypothetical, unclassified, unknown
PA2605	conserved hypothetical protein	-4.81	Hypothetical, unclassified, unknown
PA2606	conserved hypothetical protein	-6.85	Hypothetical, unclassified, unknown
PA2607	conserved hypothetical protein	-8.62	Hypothetical, unclassified, unknown
PA2608	conserved hypothetical protein	-11.36	Hypothetical, unclassified, unknown
PA2609	hypothetical protein	-14.71	Hypothetical, unclassified, unknown
PA2610	conserved hypothetical protein	-2.15	Hypothetical, unclassified, unknown
PA2625	conserved hypothetical protein	-3.72	Hypothetical, unclassified, unknown
PA2630	conserved hypothetical protein	-2.79	Hypothetical, unclassified, unknown
PA2635	hypothetical protein	3.16	Hypothetical, unclassified, unknown
PA2636	hypothetical protein	3.99	Hypothetical, unclassified, unknown
PA2650	conserved hypothetical protein	-4.35	Hypothetical, unclassified, unknown
PA2651	conserved hypothetical protein	2.08	Hypothetical, unclassified, unknown
PA2655	hypothetical protein	7.28	Hypothetical, unclassified, unknown
PA2659	hypothetical protein	-2.31	Hypothetical, unclassified, unknown
PA2668	hypothetical protein	-2.72	Hypothetical, unclassified, unknown
PA2669	hypothetical protein	3.40	Hypothetical, unclassified, unknown
PA2670	hypothetical protein	3.40	Hypothetical, unclassified, unknown
PA2671	hypothetical protein	10.96	Hypothetical, unclassified, unknown
PA2679	hypothetical protein	5.47	Hypothetical, unclassified, unknown
PA2693	conserved hypothetical protein	5.85	Hypothetical, unclassified, unknown
PA2699	hypothetical protein	-12.50	Hypothetical, unclassified, unknown
PA2707	hypothetical protein	-2.49	Hypothetical, unclassified, unknown
PA2708	hypothetical protein	4.19	Hypothetical, unclassified, unknown
PA2721	hypothetical protein	-2.62	Hypothetical, unclassified, unknown
PA2722	hypothetical protein	-4.50	Hypothetical, unclassified, unknown
PA2727	hypothetical protein	-4.95	Hypothetical, unclassified, unknown
PA2728	hypothetical protein	2.48	Hypothetical, unclassified, unknown
PA2729	hypothetical protein	-2.25	Hypothetical, unclassified, unknown
PA2730	hypothetical protein	-6.94	Hypothetical, unclassified, unknown
PA2731	Uncharacterized protein	-4.57	Hypothetical, unclassified, unknown
PA2732	hypothetical protein	-2.67	Hypothetical, unclassified, unknown
PA2733	conserved hypothetical protein	-2.72	Hypothetical, unclassified, unknown
PA2734	hypothetical protein	-3.38	Hypothetical, unclassified, unknown
PA2753	hypothetical protein	-2.45	Hypothetical, unclassified, unknown
PA2754	conserved hypothetical protein	-2.16	Hypothetical, unclassified, unknown
PA2756	hypothetical protein	-2.07	Hypothetical, unclassified, unknown
PA2762	hypothetical protein	3.39	Hypothetical, unclassified, unknown
PA2764	hypothetical protein	-4.29	Hypothetical, unclassified, unknown
PA2769	hypothetical protein	2.95	Hypothetical, unclassified, unknown
PA2770	hypothetical protein	2.05	Hypothetical, unclassified, unknown
PA2772	hypothetical protein	4.10	Hypothetical, unclassified, unknown
PA2778	hypothetical protein	8.53	Hypothetical, unclassified, unknown
PA2779	hypothetical protein	4.86	Hypothetical, unclassified, unknown
PA2789	hypothetical protein	6.72	Hypothetical, unclassified, unknown
PA2791	hypothetical protein	7.43	Hypothetical, unclassified, unknown
PA2792	hypothetical protein	2.01	Hypothetical, unclassified, unknown
PA2803	hypothetical protein	10.07	Hypothetical, unclassified, unknown
PA2804	hypothetical protein	2.36	Hypothetical, unclassified, unknown
PA2805	hypothetical protein	-5.10	Hypothetical, unclassified, unknown
PA2807	hypothetical protein	2.13	Hypothetical, unclassified, unknown
PA2814	hypothetical protein	10.37	Hypothetical, unclassified, unknown
PA2816	hypothetical protein	-3.39	Hypothetical, unclassified, unknown
PA2819	hypothetical protein	7.40	Hypothetical, unclassified, unknown
PA2829	hypothetical protein	-2.70	Hypothetical, unclassified, unknown
PA2831	conserved hypothetical protein	-2.99	Hypothetical, unclassified, unknown
PA2845	hypothetical protein	15.43	Hypothetical, unclassified, unknown
PA2847	conserved hypothetical protein	9.29	Hypothetical, unclassified, unknown
PA2855	hypothetical protein	3.47	Hypothetical, unclassified, unknown
PA2860	hypothetical protein	5.39	Hypothetical, unclassified, unknown
PA2864	conserved hypothetical protein	-4.98	Hypothetical, unclassified, unknown
PA2869	hypothetical protein	6.92	Hypothetical, unclassified, unknown
PA2872	hypothetical protein	-2.13	Hypothetical, unclassified, unknown
PA2894	hypothetical protein	2.07	Hypothetical, unclassified, unknown
PA2898	hypothetical protein	8.43	Hypothetical, unclassified, unknown
PA2901	hypothetical protein	2.52	Hypothetical, unclassified, unknown
PA2915	hypothetical protein	-2.62	Hypothetical, unclassified, unknown
PA2927	hypothetical protein	-3.70	Hypothetical, unclassified, unknown
PA2928	hypothetical protein	3.89	Hypothetical, unclassified, unknown
PA2935	hypothetical protein	11.66	Hypothetical, unclassified, unknown
PA2937	hypothetical protein	-5.99	Hypothetical, unclassified, unknown
PA2941	hypothetical protein	2.12	Hypothetical, unclassified, unknown
PA2954	hypothetical protein	3.90	Hypothetical, unclassified, unknown
PA2955	hypothetical protein	4.51	Hypothetical, unclassified, unknown
PA2956	conserved hypothetical protein	2.38	Hypothetical, unclassified, unknown
PA2963	conserved hypothetical protein	3.32	Hypothetical, unclassified, unknown
PA2971	conserved hypothetical protein	4.66	Hypothetical, unclassified, unknown
PA2992	hypothetical protein	-2.34	Hypothetical, unclassified, unknown
PA3010	hypothetical protein	-4.85	Hypothetical, unclassified, unknown
PA3015	hypothetical protein	2.52	Hypothetical, unclassified, unknown
PA3016	hypothetical protein	4.94	Hypothetical, unclassified, unknown
PA3018	hypothetical protein	2.84	Hypothetical, unclassified, unknown
PA3021	hypothetical protein	-5.35	Hypothetical, unclassified, unknown
PA3022	hypothetical protein	-2.81	Hypothetical, unclassified, unknown
PA3031	hypothetical protein	-4.13	Hypothetical, unclassified, unknown
PA3037	hypothetical protein	-3.14	Hypothetical, unclassified, unknown
PA3040	conserved hypothetical protein	-7.14	Hypothetical, unclassified, unknown
PA3042	hypothetical protein	-6.29	Hypothetical, unclassified, unknown
PA3048	conserved hypothetical protein	4.30	Hypothetical, unclassified, unknown
PA3051	hypothetical protein	3.65	Hypothetical, unclassified, unknown
PA3052	hypothetical protein	-2.78	Hypothetical, unclassified, unknown
PA3054	hypothetical protein	-6.54	Hypothetical, unclassified, unknown
PA3069	hypothetical protein	10.53	Hypothetical, unclassified, unknown
PA3075	hypothetical protein	-3.85	Hypothetical, unclassified, unknown
PA3081	conserved hypothetical protein	-2.53	Hypothetical, unclassified, unknown
PA3087	hypothetical protein	-2.65	Hypothetical, unclassified, unknown
PA3090	hypothetical protein	5.88	Hypothetical, unclassified, unknown
PA3091	hypothetical protein	-2.79	Hypothetical, unclassified, unknown
PA3119	conserved hypothetical protein	-2.09	Hypothetical, unclassified, unknown
PA3123	RidA subfamily protein	-4.39	Hypothetical, unclassified, unknown
PA3127	hypothetical protein	-2.58	Hypothetical, unclassified, unknown
PA3129	conserved hypothetical protein	5.75	Hypothetical, unclassified, unknown
PA3140	hypothetical protein	6.01	Hypothetical, unclassified, unknown
PA3179	conserved hypothetical protein	7.07	Hypothetical, unclassified, unknown
PA3198	conserved hypothetical protein	-4.63	Hypothetical, unclassified, unknown
PA3199	conserved hypothetical protein	-3.76	Hypothetical, unclassified, unknown
PA3202	conserved hypothetical protein	-3.79	Hypothetical, unclassified, unknown
PA3203	hypothetical protein	-2.88	Hypothetical, unclassified, unknown
PA3205	hypothetical protein	6.74	Hypothetical, unclassified, unknown
PA3209	conserved hypothetical protein	7.09	Hypothetical, unclassified, unknown
PA3213	hypothetical protein	-2.20	Hypothetical, unclassified, unknown
PA3214	hypothetical protein	-5.15	Hypothetical, unclassified, unknown
PA3218	hypothetical protein	15.99	Hypothetical, unclassified, unknown
PA3219	hypothetical protein	15.57	Hypothetical, unclassified, unknown
PA3224	hypothetical protein	-4.27	Hypothetical, unclassified, unknown
PA3237	hypothetical protein	10.32	Hypothetical, unclassified, unknown
PA3239	conserved hypothetical protein	-2.11	Hypothetical, unclassified, unknown
PA3250	hypothetical protein	-5.99	Hypothetical, unclassified, unknown
PA3251	hypothetical protein	-4.29	Hypothetical, unclassified, unknown
PA3255	hypothetical protein	-4.07	Hypothetical, unclassified, unknown
PA3261	hypothetical protein	-2.30	Hypothetical, unclassified, unknown
PA3270	hypothetical protein	2.18	Hypothetical, unclassified, unknown
PA3273	hypothetical protein	3.18	Hypothetical, unclassified, unknown
PA3276	hypothetical protein	2.78	Hypothetical, unclassified, unknown
PA3282	hypothetical protein	11.44	Hypothetical, unclassified, unknown
PA3283	conserved hypothetical protein	8.09	Hypothetical, unclassified, unknown
PA3284	hypothetical protein	5.78	Hypothetical, unclassified, unknown
PA3298	hypothetical protein	2.41	Hypothetical, unclassified, unknown
PA3306	hypothetical protein	9.01	Hypothetical, unclassified, unknown
PA3307	hypothetical protein	4.15	Hypothetical, unclassified, unknown
PA3309	conserved hypothetical protein	-9.71	Hypothetical, unclassified, unknown
PA3322	hypothetical protein	2.90	Hypothetical, unclassified, unknown
PA3329	hypothetical protein	-6.06	Hypothetical, unclassified, unknown
PA3332	conserved hypothetical protein	-9.90	Hypothetical, unclassified, unknown
PA3335	hypothetical protein	-4.42	Hypothetical, unclassified, unknown
PA3338	hypothetical protein	2.28	Hypothetical, unclassified, unknown
PA3354	hypothetical protein	-3.53	Hypothetical, unclassified, unknown
PA3371	hypothetical protein	4.99	Hypothetical, unclassified, unknown
PA3386	conserved hypothetical protein	8.34	Hypothetical, unclassified, unknown
PA3388	conserved hypothetical protein	7.38	Hypothetical, unclassified, unknown
PA3390	hypothetical protein	18.24	Hypothetical, unclassified, unknown
PA3399	hypothetical protein	-2.74	Hypothetical, unclassified, unknown
PA3402	hypothetical protein	2.14	Hypothetical, unclassified, unknown
PA3411	hypothetical protein	7.14	Hypothetical, unclassified, unknown
PA3412	hypothetical protein	2.04	Hypothetical, unclassified, unknown
PA3413	conserved hypothetical protein	5.29	Hypothetical, unclassified, unknown
PA3414	hypothetical protein	9.51	Hypothetical, unclassified, unknown
PA3422	hypothetical protein	4.48	Hypothetical, unclassified, unknown
PA3425	hypothetical protein	-3.55	Hypothetical, unclassified, unknown
PA3436	hypothetical protein	2.22	Hypothetical, unclassified, unknown
PA3440	conserved hypothetical protein	-2.76	Hypothetical, unclassified, unknown
PA3451	hypothetical protein	-9.17	Hypothetical, unclassified, unknown
PA3455	conserved hypothetical protein	-4.81	Hypothetical, unclassified, unknown
PA3461	conserved hypothetical protein	-9.35	Hypothetical, unclassified, unknown
PA3464	hypothetical protein	6.27	Hypothetical, unclassified, unknown
PA3481	conserved hypothetical protein	-2.25	Hypothetical, unclassified, unknown
PA3493	conserved hypothetical protein	-2.98	Hypothetical, unclassified, unknown
PA3494	conserved hypothetical protein	-3.00	Hypothetical, unclassified, unknown
PA3496	hypothetical protein	-2.01	Hypothetical, unclassified, unknown
PA3497	hypothetical protein	8.48	Hypothetical, unclassified, unknown
PA3513	hypothetical protein	10.02	Hypothetical, unclassified, unknown
PA3518	hypothetical protein	2.70	Hypothetical, unclassified, unknown
PA3519	hypothetical protein	2.63	Hypothetical, unclassified, unknown
PA3520	hypothetical protein	-5.35	Hypothetical, unclassified, unknown
PA3539	conserved hypothetical protein	-2.76	Hypothetical, unclassified, unknown
PA3564	conserved hypothetical protein	-2.05	Hypothetical, unclassified, unknown
PA3566	conserved hypothetical protein	-2.53	Hypothetical, unclassified, unknown
PA3572	hypothetical protein	-7.94	Hypothetical, unclassified, unknown
PA3577	hypothetical protein	3.21	Hypothetical, unclassified, unknown
PA3580	conserved hypothetical protein	2.05	Hypothetical, unclassified, unknown
PA3598	conserved hypothetical protein	14.03	Hypothetical, unclassified, unknown
PA3606	conserved hypothetical protein	4.50	Hypothetical, unclassified, unknown
PA3614	hypothetical protein	-11.36	Hypothetical, unclassified, unknown
PA3615	hypothetical protein	-7.75	Hypothetical, unclassified, unknown
PA3616	conserved hypothetical protein	9.25	Hypothetical, unclassified, unknown
PA3623	conserved hypothetical protein	-3.29	Hypothetical, unclassified, unknown
PA3638	conserved hypothetical protein	4.86	Hypothetical, unclassified, unknown
PA3661	hypothetical protein	4.35	Hypothetical, unclassified, unknown
PA3662	hypothetical protein	5.95	Hypothetical, unclassified, unknown
PA3664	conserved hypothetical protein	3.61	Hypothetical, unclassified, unknown
PA3668	conserved hypothetical protein	-2.66	Hypothetical, unclassified, unknown
PA3670	hypothetical protein	-2.17	Hypothetical, unclassified, unknown
PA3674	hypothetical protein	2.25	Hypothetical, unclassified, unknown
PA3679	hypothetical protein	2.61	Hypothetical, unclassified, unknown
PA3680	conserved hypothetical protein	2.13	Hypothetical, unclassified, unknown
PA3684	hypothetical protein	2.01	Hypothetical, unclassified, unknown
PA3688	hypothetical protein	-7.19	Hypothetical, unclassified, unknown
PA3693	conserved hypothetical protein	-3.28	Hypothetical, unclassified, unknown
PA3694	hypothetical protein	-3.66	Hypothetical, unclassified, unknown
PA3695	hypothetical protein	-6.33	Hypothetical, unclassified, unknown
PA3696	conserved hypothetical protein	-8.47	Hypothetical, unclassified, unknown
PA3720	hypothetical protein	7.51	Hypothetical, unclassified, unknown
PA3722	hypothetical protein	3.55	Hypothetical, unclassified, unknown
PA3728	hypothetical protein	2.43	Hypothetical, unclassified, unknown
PA3729	conserved hypothetical protein	2.61	Hypothetical, unclassified, unknown
PA3731	conserved hypothetical protein	3.76	Hypothetical, unclassified, unknown
PA3732	Uncharacterized protein	4.32	Hypothetical, unclassified, unknown
PA3733	hypothetical protein	-3.56	Hypothetical, unclassified, unknown
PA3734	hypothetical protein	-7.87	Hypothetical, unclassified, unknown
PA3741	hypothetical protein	27.01	Hypothetical, unclassified, unknown
PA3750	hypothetical protein	-2.08	Hypothetical, unclassified, unknown
PA3752	hypothetical protein	-2.30	Hypothetical, unclassified, unknown
PA3762	hypothetical protein	4.31	Hypothetical, unclassified, unknown
PA3765	hypothetical protein	3.52	Hypothetical, unclassified, unknown
PA3767	conserved hypothetical protein	-2.35	Hypothetical, unclassified, unknown
PA3784	hypothetical protein	-34.48	Hypothetical, unclassified, unknown
PA3785	conserved hypothetical protein	-55.56	Hypothetical, unclassified, unknown
PA3786	hypothetical protein	-2.28	Hypothetical, unclassified, unknown
PA3793	hypothetical protein	2.75	Hypothetical, unclassified, unknown
PA3796	hypothetical protein	2.06	Hypothetical, unclassified, unknown
PA3801	conserved hypothetical protein	-2.39	Hypothetical, unclassified, unknown
PA3808	conserved hypothetical protein	-2.02	Hypothetical, unclassified, unknown
PA3829	hypothetical protein	2.98	Hypothetical, unclassified, unknown
PA3833	hypothetical protein	-3.45	Hypothetical, unclassified, unknown
PA3835	hypothetical protein	2.19	Hypothetical, unclassified, unknown
PA3836	hypothetical protein	-2.52	Hypothetical, unclassified, unknown
PA3843	hypothetical protein	-2.82	Hypothetical, unclassified, unknown
PA3846	hypothetical protein	-2.32	Hypothetical, unclassified, unknown
PA3847	conserved hypothetical protein	-2.34	Hypothetical, unclassified, unknown
PA3848	hypothetical protein	-8.77	Hypothetical, unclassified, unknown
PA3851	hypothetical protein	-2.03	Hypothetical, unclassified, unknown
PA3855	hypothetical protein	-2.02	Hypothetical, unclassified, unknown
PA3856	hypothetical protein	-4.24	Hypothetical, unclassified, unknown
PA3868	hypothetical protein	3.15	Hypothetical, unclassified, unknown
PA3869	hypothetical protein	6.33	Hypothetical, unclassified, unknown
PA3882	hypothetical protein	-2.60	Hypothetical, unclassified, unknown
PA3886	hypothetical protein	-2.39	Hypothetical, unclassified, unknown
PA3902	hypothetical protein	7.27	Hypothetical, unclassified, unknown
PA3919	conserved hypothetical protein	-15.38	Hypothetical, unclassified, unknown
PA3922	conserved hypothetical protein	-5.29	Hypothetical, unclassified, unknown
PA3923	hypothetical protein	-4.42	Hypothetical, unclassified, unknown
PA3928	hypothetical protein	-2.70	Hypothetical, unclassified, unknown
PA3931	conserved hypothetical protein	2.42	Hypothetical, unclassified, unknown
PA3939	hypothetical protein	4.25	Hypothetical, unclassified, unknown
PA3951	conserved hypothetical protein	7.58	Hypothetical, unclassified, unknown
PA3952	hypothetical protein	6.51	Hypothetical, unclassified, unknown
PA3956	hypothetical protein	-3.72	Hypothetical, unclassified, unknown
PA3959	hypothetical protein	2.39	Hypothetical, unclassified, unknown
PA3960	hypothetical protein	2.56	Hypothetical, unclassified, unknown
PA3962	hypothetical protein	2.47	Hypothetical, unclassified, unknown
PA3967	hypothetical protein	4.43	Hypothetical, unclassified, unknown
PA3979	hypothetical protein	11.14	Hypothetical, unclassified, unknown
PA3980	conserved hypothetical protein	2.20	Hypothetical, unclassified, unknown
PA3981	conserved hypothetical protein	-3.95	Hypothetical, unclassified, unknown
PA3982	conserved hypothetical protein	-4.81	Hypothetical, unclassified, unknown
PA3983	conserved hypothetical protein	-6.80	Hypothetical, unclassified, unknown
PA3986	hypothetical protein	-2.34	Hypothetical, unclassified, unknown
PA3990	conserved hypothetical protein	4.66	Hypothetical, unclassified, unknown
PA3991	hypothetical protein	26.40	Hypothetical, unclassified, unknown
PA3998	conserved hypothetical protein	-4.37	Hypothetical, unclassified, unknown
PA4009	hypothetical protein	7.85	Hypothetical, unclassified, unknown
PA4015	conserved hypothetical protein	-14.93	Hypothetical, unclassified, unknown
PA4027	hypothetical protein	-4.27	Hypothetical, unclassified, unknown
PA4035	hypothetical protein	-3.02	Hypothetical, unclassified, unknown
PA4039	hypothetical protein	2.63	Hypothetical, unclassified, unknown
PA4045	conserved hypothetical protein	4.25	Hypothetical, unclassified, unknown
PA4059	hypothetical protein	-2.19	Hypothetical, unclassified, unknown
PA4071	hypothetical protein	3.23	Hypothetical, unclassified, unknown
PA4076	hypothetical protein	-2.51	Hypothetical, unclassified, unknown
PA4087	conserved hypothetical protein	11.22	Hypothetical, unclassified, unknown
PA4090	hypothetical protein	-8.47	Hypothetical, unclassified, unknown
PA4095	hypothetical protein	19.88	Hypothetical, unclassified, unknown
PA4099	hypothetical protein	6.16	Hypothetical, unclassified, unknown
PA4105	hypothetical protein	2.70	Hypothetical, unclassified, unknown
PA4106	conserved hypothetical protein	4.93	Hypothetical, unclassified, unknown
PA4139	hypothetical protein	-2.69	Hypothetical, unclassified, unknown
PA4141	hypothetical protein	-12.66	Hypothetical, unclassified, unknown
PA4146	hypothetical protein	3.39	Hypothetical, unclassified, unknown
PA4149	conserved hypothetical protein	12.36	Hypothetical, unclassified, unknown
PA4154	conserved hypothetical protein	7.98	Hypothetical, unclassified, unknown
PA4169	conserved hypothetical protein	2.02	Hypothetical, unclassified, unknown
PA4173	conserved hypothetical protein	10.55	Hypothetical, unclassified, unknown
PA4200	hypothetical protein	-6.25	Hypothetical, unclassified, unknown
PA4278	hypothetical protein	3.46	Hypothetical, unclassified, unknown
PA4279	hypothetical protein	2.55	Hypothetical, unclassified, unknown
PA4298	hypothetical protein	-12.05	Hypothetical, unclassified, unknown
PA4322	conserved hypothetical protein	-6.17	Hypothetical, unclassified, unknown
PA4323	hypothetical protein	-4.39	Hypothetical, unclassified, unknown

PA4324	hypothetical protein	2.55	Hypothetical, unclassified, unknown
PA4325	hypothetical protein	4.34	Hypothetical, unclassified, unknown
PA4326	hypothetical protein	4.71	Hypothetical, unclassified, unknown
PA4328	hypothetical protein	-3.28	Hypothetical, unclassified, unknown
PA4335	hypothetical protein	-2.05	Hypothetical, unclassified, unknown
PA4336	conserved hypothetical protein	-2.73	Hypothetical, unclassified, unknown
PA4340	hypothetical protein	2.21	Hypothetical, unclassified, unknown
PA4345	hypothetical protein	2.10	Hypothetical, unclassified, unknown
PA4348	conserved hypothetical protein	-2.54	Hypothetical, unclassified, unknown
PA4352	conserved hypothetical protein	-8.55	Hypothetical, unclassified, unknown
PA4353	conserved hypothetical protein	2.18	Hypothetical, unclassified, unknown
PA4357	conserved hypothetical protein	-12.99	Hypothetical, unclassified, unknown
PA4359	conserved hypothetical protein	-3.46	Hypothetical, unclassified, unknown
PA4360	hypothetical protein	2.71	Hypothetical, unclassified, unknown
PA4362	hypothetical protein	-4.57	Hypothetical, unclassified, unknown
PA4364	hypothetical protein	4.20	Hypothetical, unclassified, unknown
PA4368	hypothetical protein	2.02	Hypothetical, unclassified, unknown
PA4371	hypothetical protein	-3.98	Hypothetical, unclassified, unknown
PA4372	hypothetical protein	-9.71	Hypothetical, unclassified, unknown
PA4373	hypothetical protein	-9.01	Hypothetical, unclassified, unknown
PA4377	hypothetical protein	2.14	Hypothetical, unclassified, unknown
PA4382	hypothetical protein	7.23	Hypothetical, unclassified, unknown
PA4384	hypothetical protein	-10.87	Hypothetical, unclassified, unknown
PA4390	hypothetical protein	5.08	Hypothetical, unclassified, unknown
PA4391	hypothetical protein	4.08	Hypothetical, unclassified, unknown
PA4392	conserved hypothetical protein	2.05	Hypothetical, unclassified, unknown
PA4399	conserved hypothetical protein	-2.05	Hypothetical, unclassified, unknown
PA4424	conserved hypothetical protein	-5.18	Hypothetical, unclassified, unknown
PA4426	conserved hypothetical protein	-6.94	Hypothetical, unclassified, unknown
PA4437	hypothetical protein	2.82	Hypothetical, unclassified, unknown
PA4438	conserved hypothetical protein	2.11	Hypothetical, unclassified, unknown
PA4453	conserved hypothetical protein	-2.18	Hypothetical, unclassified, unknown
PA4463	conserved hypothetical protein	-14.93	Hypothetical, unclassified, unknown
PA4465	conserved hypothetical protein	-5.29	Hypothetical, unclassified, unknown
PA4469	hypothetical protein	-26.32	Hypothetical, unclassified, unknown
PA4476	hypothetical protein	3.72	Hypothetical, unclassified, unknown
PA4478	conserved hypothetical protein	2.45	Hypothetical, unclassified, unknown
PA4495	hypothetical protein	-5.95	Hypothetical, unclassified, unknown
PA4515	conserved hypothetical protein	-4.31	Hypothetical, unclassified, unknown
PA4516	hypothetical protein	-9.71	Hypothetical, unclassified, unknown
PA4517	conserved hypothetical protein	-2.05	Hypothetical, unclassified, unknown
PA4523	hypothetical protein	-2.13	Hypothetical, unclassified, unknown
PA4530	conserved hypothetical protein	-4.74	Hypothetical, unclassified, unknown
PA4535	hypothetical protein	-2.30	Hypothetical, unclassified, unknown
PA4536	hypothetical protein	-4.37	Hypothetical, unclassified, unknown
PA4537	hypothetical protein	-3.15	Hypothetical, unclassified, unknown
PA4539	hypothetical protein	3.28	Hypothetical, unclassified, unknown
PA4540	hypothetical protein	2.79	Hypothetical, unclassified, unknown
PA4543	conserved hypothetical protein	2.07	Hypothetical, unclassified, unknown
PA4562	conserved hypothetical protein	2.96	Hypothetical, unclassified, unknown
PA4564	conserved hypothetical protein	5.23	Hypothetical, unclassified, unknown
PA4570	hypothetical protein	-8.13	Hypothetical, unclassified, unknown
PA4574	conserved hypothetical protein	4.63	Hypothetical, unclassified, unknown
PA4575	hypothetical protein	2.67	Hypothetical, unclassified, unknown
PA4578	hypothetical protein	-5.10	Hypothetical, unclassified, unknown
PA4579	hypothetical protein	-3.61	Hypothetical, unclassified, unknown
PA4580	conserved hypothetical protein	3.43	Hypothetical, unclassified, unknown
PA4582	conserved hypothetical protein	66.98	Hypothetical, unclassified, unknown
PA4583	conserved hypothetical protein	31.43	Hypothetical, unclassified, unknown
PA4584	conserved hypothetical protein	22.78	Hypothetical, unclassified, unknown
PA4604	conserved hypothetical protein	-2.20	Hypothetical, unclassified, unknown
PA4605	conserved hypothetical protein	-2.11	Hypothetical, unclassified, unknown
PA4607	hypothetical protein	-29.41	Hypothetical, unclassified, unknown
PA4610	hypothetical protein	4.56	Hypothetical, unclassified, unknown
PA4611	hypothetical protein	-10.10	Hypothetical, unclassified, unknown
PA4623	hypothetical protein	5.19	Hypothetical, unclassified, unknown
PA4629	hypothetical protein	18.80	Hypothetical, unclassified, unknown
PA4630	hypothetical protein	5.87	Hypothetical, unclassified, unknown
PA4632	hypothetical protein	-2.35	Hypothetical, unclassified, unknown
PA4634	hypothetical protein	6.34	Hypothetical, unclassified, unknown
PA4635	conserved hypothetical protein	18.75	Hypothetical, unclassified, unknown
PA4637	hypothetical protein	11.08	Hypothetical, unclassified, unknown
PA4638	hypothetical protein	8.45	Hypothetical, unclassified, unknown
PA4639	hypothetical protein	2.18	Hypothetical, unclassified, unknown
PA4644	hypothetical protein	-2.30	Hypothetical, unclassified, unknown
PA4656	conserved hypothetical protein	3.20	Hypothetical, unclassified, unknown
PA4657	hypothetical protein	-2.46	Hypothetical, unclassified, unknown
PA4667	hypothetical protein	-3.05	Hypothetical, unclassified, unknown
PA4674	Antitoxin HigA	6.87	Hypothetical, unclassified, unknown
PA4686	hypothetical protein	-3.82	Hypothetical, unclassified, unknown
PA4697	hypothetical protein	-2.81	Hypothetical, unclassified, unknown
PA4701	conserved hypothetical protein	-5.21	Hypothetical, unclassified, unknown
PA4702	hypothetical protein	-7.94	Hypothetical, unclassified, unknown
PA4703	hypothetical protein	-4.33	Hypothetical, unclassified, unknown
PA4712	hypothetical protein	3.10	Hypothetical, unclassified, unknown
PA4713	hypothetical protein	4.72	Hypothetical, unclassified, unknown
PA4714	conserved hypothetical protein	-2.72	Hypothetical, unclassified, unknown
PA4716	hypothetical protein	3.25	Hypothetical, unclassified, unknown
PA4717	conserved hypothetical protein	3.37	Hypothetical, unclassified, unknown
PA4718	hypothetical protein	4.78	Hypothetical, unclassified, unknown
PA4724.1	conserved hypothetical protein	6.85	Hypothetical, unclassified, unknown
PA4736	hypothetical protein	-2.11	Hypothetical, unclassified, unknown
PA4746	conserved hypothetical protein	5.66	Hypothetical, unclassified, unknown
PA4766	conserved hypothetical protein	3.18	Hypothetical, unclassified, unknown
PA4782	hypothetical protein	4.72	Hypothetical, unclassified, unknown
PA4793	hypothetical protein	-3.51	Hypothetical, unclassified, unknown
PA4794	hypothetical protein	-3.46	Hypothetical, unclassified, unknown
PA4795	hypothetical protein	-6.06	Hypothetical, unclassified, unknown
PA4796	hypothetical protein	-7.09	Hypothetical, unclassified, unknown
PA4798	hypothetical protein	-4.83	Hypothetical, unclassified, unknown
PA4799	hypothetical protein	6.33	Hypothetical, unclassified, unknown
PA4800	hypothetical protein	6.87	Hypothetical, unclassified, unknown
PA4801	hypothetical protein	2.51	Hypothetical, unclassified, unknown
PA4816	hypothetical protein	11.05	Hypothetical, unclassified, unknown
PA4817	hypothetical protein	15.53	Hypothetical, unclassified, unknown
PA4820	hypothetical protein	12.02	Hypothetical, unclassified, unknown
PA4852	conserved hypothetical protein	4.49	Hypothetical, unclassified, unknown
PA4858	conserved hypothetical protein	6.94	Hypothetical, unclassified, unknown
PA4863	hypothetical protein	-2.98	Hypothetical, unclassified, unknown
PA4866	conserved hypothetical protein	-10.99	Hypothetical, unclassified, unknown
PA4869	hypothetical protein	-3.38	Hypothetical, unclassified, unknown
PA4870	conserved hypothetical protein	4.50	Hypothetical, unclassified, unknown
PA4874	conserved hypothetical protein	-3.37	Hypothetical, unclassified, unknown
PA4875	hypothetical protein	-2.99	Hypothetical, unclassified, unknown
PA4877	hypothetical protein	2.54	Hypothetical, unclassified, unknown
PA4879	conserved hypothetical protein	3.57	Hypothetical, unclassified, unknown
PA4881	hypothetical protein	24.55	Hypothetical, unclassified, unknown
PA4882	hypothetical protein	5.02	Hypothetical, unclassified, unknown
PA4883	hypothetical protein	10.06	Hypothetical, unclassified, unknown
PA4884	hypothetical protein	15.58	Hypothetical, unclassified, unknown
PA4926	conserved hypothetical protein	-3.39	Hypothetical, unclassified, unknown
PA4928	conserved hypothetical protein	2.63	Hypothetical, unclassified, unknown
PA4948	conserved hypothetical protein	4.75	Hypothetical, unclassified, unknown
PA4949	conserved hypothetical protein	3.05	Hypothetical, unclassified, unknown
PA4966	hypothetical protein	-2.29	Hypothetical, unclassified, unknown
PA4970	conserved hypothetical protein	4.14	Hypothetical, unclassified, unknown
PA4972	hypothetical protein	2.36	Hypothetical, unclassified, unknown
PA4985	Uncharacterized protein	10.89	Hypothetical, unclassified, unknown
PA5019	conserved hypothetical protein	4.03	Hypothetical, unclassified, unknown
PA5023	conserved hypothetical protein	-2.05	Hypothetical, unclassified, unknown
PA5026	hypothetical protein	-2.07	Hypothetical, unclassified, unknown
PA5027	hypothetical protein	-13.89	Hypothetical, unclassified, unknown
PA5028	conserved hypothetical protein	-2.15	Hypothetical, unclassified, unknown
PA5033	hypothetical protein	-2.87	Hypothetical, unclassified, unknown
PA5047	hypothetical protein	4.88	Hypothetical, unclassified, unknown
PA5052	hypothetical protein	-2.10	Hypothetical, unclassified, unknown
PA5064	hypothetical protein	-2.87	Hypothetical, unclassified, unknown
PA5073	hypothetical protein	3.75	Hypothetical, unclassified, unknown
PA5079	conserved hypothetical protein	-2.18	Hypothetical, unclassified, unknown
PA5101	hypothetical protein	-4.33	Hypothetical, unclassified, unknown
PA5108	hypothetical protein	7.73	Hypothetical, unclassified, unknown
PA5109	hypothetical protein	-2.07	Hypothetical, unclassified, unknown
PA5122	hypothetical protein	-2.02	Hypothetical, unclassified, unknown
PA5123	hypothetical protein	-2.62	Hypothetical, unclassified, unknown
PA5144	hypothetical protein	2.04	Hypothetical, unclassified, unknown
PA5148	conserved hypothetical protein	-2.28	Hypothetical, unclassified, unknown
PA5156	hypothetical protein	7.42	Hypothetical, unclassified, unknown
PA5176	conserved hypothetical protein	3.74	Hypothetical, unclassified, unknown
PA5191	hypothetical protein	-2.65	Hypothetical, unclassified, unknown
PA5196	hypothetical protein	-2.44	Hypothetical, unclassified, unknown
PA5220	hypothetical protein	-10.10	Hypothetical, unclassified, unknown
PA5222	hypothetical protein	-2.82	Hypothetical, unclassified, unknown
PA5228	conserved hypothetical protein	2.74	Hypothetical, unclassified, unknown
PA5232	conserved hypothetical protein	-3.13	Hypothetical, unclassified, unknown
PA5233	hypothetical protein	-2.13	Hypothetical, unclassified, unknown
PA5245	conserved hypothetical protein	-2.79	Hypothetical, unclassified, unknown
PA5246	conserved hypothetical protein	2.37	Hypothetical, unclassified, unknown
PA5270	hypothetical protein	-2.18	Hypothetical, unclassified, unknown
PA5271	hypothetical protein	-14.93	Hypothetical, unclassified, unknown
PA5284	hypothetical protein	2.64	Hypothetical, unclassified, unknown
PA5290	conserved hypothetical protein	2.11	Hypothetical, unclassified, unknown
PA5294	putative multidrug efflux pump	5.20	Hypothetical, unclassified, unknown
PA5299	hypothetical protein	2.97	Hypothetical, unclassified, unknown
PA5303	conserved hypothetical protein	-3.95	Hypothetical, unclassified, unknown
PA5305	conserved hypothetical protein	-2.40	Hypothetical, unclassified, unknown
PA5307	hypothetical protein	2.69	Hypothetical, unclassified, unknown
PA5314	hypothetical protein	2.17	Hypothetical, unclassified, unknown
PA5318	hypothetical protein	-7.52	Hypothetical, unclassified, unknown
PA5329	conserved hypothetical protein	-5.68	Hypothetical, unclassified, unknown
PA5335	conserved hypothetical protein	3.33	Hypothetical, unclassified, unknown
PA5339	conserved hypothetical protein	-6.45	Hypothetical, unclassified, unknown
PA5340	hypothetical protein	-5.78	Hypothetical, unclassified, unknown
PA5341	hypothetical protein	3.59	Hypothetical, unclassified, unknown
PA5352	conserved hypothetical protein	4.20	Hypothetical, unclassified, unknown
PA5359	hypothetical protein	-5.08	Hypothetical, unclassified, unknown
PA5362	conserved hypothetical protein	-4.57	Hypothetical, unclassified, unknown
PA5393	conserved hypothetical protein	4.00	Hypothetical, unclassified, unknown
PA5395	conserved hypothetical protein	-4.00	Hypothetical, unclassified, unknown
PA5406	hypothetical protein	3.08	Hypothetical, unclassified, unknown
PA5407	hypothetical protein	4.97	Hypothetical, unclassified, unknown
PA5408	hypothetical protein	-2.89	Hypothetical, unclassified, unknown
PA5412	hypothetical protein	2.80	Hypothetical, unclassified, unknown
PA5423	hypothetical protein	7.52	Hypothetical, unclassified, unknown
PA5446	hypothetical protein	-5.85	Hypothetical, unclassified, unknown
PA5460	hypothetical protein	-8.70	Hypothetical, unclassified, unknown
PA5461	hypothetical protein	-7.63	Hypothetical, unclassified, unknown
PA5464	hypothetical protein	2.44	Hypothetical, unclassified, unknown
PA5465	hypothetical protein	16.32	Hypothetical, unclassified, unknown
PA5471.1	PA5471 leader peptide	4.53	Hypothetical, unclassified, unknown
PA5475	hypothetical protein	-12.99	Hypothetical, unclassified, unknown
PA5480	hypothetical protein	7.26	Hypothetical, unclassified, unknown
PA5486	conserved hypothetical protein	5.73	Hypothetical, unclassified, unknown
PA5506	hypothetical protein	2.57	Hypothetical, unclassified, unknown
PA5509	hypothetical protein	2.85	Hypothetical, unclassified, unknown
PA5517	conserved hypothetical protein	3.85	Hypothetical, unclassified, unknown
PA5519	conserved hypothetical protein	2.42	Hypothetical, unclassified, unknown
PA5520	hypothetical protein	-2.02	Hypothetical, unclassified, unknown
PA5526	hypothetical protein	6.29	Hypothetical, unclassified, unknown
PA5533	hypothetical protein	3.10	Hypothetical, unclassified, unknown
PA5534	hypothetical protein	4.34	Hypothetical, unclassified, unknown
PA5535	conserved hypothetical protein	3.22	Hypothetical, unclassified, unknown
PA5539	RidA subfamily protein	6.32	Hypothetical, unclassified, unknown
PA5540	hypothetical protein	4.49	Hypothetical, unclassified, unknown
PA5566	hypothetical protein	4.92	Hypothetical, unclassified, unknown

Classification by PA number

PA number	Gene name	Product name	Fold-change	PseudoCAP1	PseudoCAP2	PseudoCAP3	Annotated as encoding virulence factors (Winsor et al., 2016)
PA0002	*dnaN*	DNA polymerase III, beta chain	-2,04	DNA replication, recombination, modification and repair			
PA0005	*lptA*	lysophosphatidic acid acyltransferase, LptA	3,67	Fatty acid and phospholipid metabolism			
PA0007		hypothetical protein	-2,20	Membrane proteins			
PA0008	*glyS*	glycyl-tRNA synthetase beta chain	-2,58	Translation, post-translational modification, degradation	Amino acid biosynthesis and metabolism		
PA0013		conserved hypothetical protein	5,44	Membrane proteins			
PA0014		hypothetical protein	2,33	Hypothetical, unclassified, unknown			
PA0016	*trkA*	potassium uptake protein TrkA	-3,88	Transport of small molecules			
PA0017		conserved hypothetical protein	-3,94	Hypothetical, unclassified, unknown			
PA0018	*fmt*	methionyl-tRNA formyltransferase	-2,55	Translation, post-translational modification, degradation	Amino acid biosynthesis and metabolism		
PA0021		conserved hypothetical protein	3,97	Membrane proteins			
PA0023	*qor*	quinone oxidoreductase	-5,85	Energy metabolism			
PA0026	*plcB*	phospholipase C, PlcB	-6,06	Hypothetical, unclassified, unknown			
PA0027		hypothetical protein	-7,52	Hypothetical, unclassified, unknown			
PA0028		Lipoprotein	-5,68	Hypothetical, unclassified, unknown			
PA0029		probable sulfate transporter	2,71	Membrane proteins	Transport of small molecules		
PA0032		probable transcriptional regulator	4,78	Transcriptional regulators			
PA0034		probable two-component response regulator	2,81	Two-component regulatory systems	Transcriptional regulators		
PA0037	*trpI*	transcriptional regulator TrpI	6,09	Amino acid biosynthesis and metabolism	Transcriptional regulators		
PA0039		hypothetical protein	-4,00	Hypothetical, unclassified, unknown			
PA0040		conserved hypothetical protein	11,81	Hypothetical, unclassified, unknown			
PA0041		probable hemagglutinin	2,92	Secreted Factors (toxins, enzymes, alginate)			x
PA0042		Lipoprotein	2,42	Hypothetical, unclassified, unknown			
PA0043		hypothetical protein	2,18	Membrane proteins			
PA0044	*exoT*	exoenzyme T	3,47	Secreted Factors (toxins, enzymes, alginate)			x
PA0045		Lipoprotein	2,40	Hypothetical, unclassified, unknown			
PA0046		Lipoprotein	2,58	Hypothetical, unclassified, unknown			
PA0047		hypothetical protein	3,04	Hypothetical, unclassified, unknown			
PA0048		probable transcriptional regulator	-33,33	Transcriptional regulators			
PA0049		hypothetical protein	-52,63	Hypothetical, unclassified, unknown			
PA0050		hypothetical protein	-10,31	Hypothetical, unclassified, unknown			
PA0051	*phzH*	potential phenazine-modifying enzyme	-8,40	Putative enzymes			x
PA0052		hypothetical protein	-6,80	Hypothetical, unclassified, unknown			
PA0053		hypothetical protein	2,11	Hypothetical, unclassified, unknown			
PA0054		conserved hypothetical protein	-6,25	Hypothetical, unclassified, unknown			
PA0055		hypothetical protein	-5,75	Hypothetical, unclassified, unknown			
PA0056		probable transcriptional regulator	4,36	Transcriptional regulators			
PA0057		hypothetical protein	4,56	Hypothetical, unclassified, unknown			
PA0058	*dsbM*	DsbM	2,22	Antibiotic resistance and susceptibility			
PA0059	*osmC*	osmotically inducible protein OsmC	-2,62	Adaptation, Protection			
PA0062		Lipoprotein	2,12	Hypothetical, unclassified, unknown			
PA0063		hypothetical protein	3,09	Hypothetical, unclassified, unknown			
PA0067	*prlC*	oligopeptidase A	-3,50	Translation, post-translational modification, degradation			
PA0068		hypothetical protein	-5,21	Hypothetical, unclassified, unknown			
PA0074	*ppkA*	serine/threonine protein kinase PpkA	-2,13	Translation, post-translational modification, degradation	Adaptation, Protection	Protein secretion/export apparatus	x
PA0078	*tssL1*	TssL1	-2,83	Protein secretion/export apparatus			x
PA0079	*tssK1*	TssK1	-2,42	Protein secretion/export apparatus			x
PA0080	*tssJ1*	TssJ1	-2,04	Protein secretion/export apparatus			x
PA0084	*tssC1*	TssC1	-2,13	Protein secretion/export apparatus			x
PA0105	*coxB*	cytochrome c oxidase, subunit II	2,20	Energy metabolism			
PA0107		conserved hypothetical protein	-3,37	Energy metabolism			
PA0108	*coIII*	cytochrome c oxidase, subunit III	-4,07	Energy metabolism			
PA0109		hypothetical protein	-2,44	Membrane proteins			
PA0111		hypothetical protein	-5,24	Hypothetical, unclassified, unknown			
PA0112		hypothetical protein	-4,85	Membrane proteins			
PA0113		probable cytochrome c oxidase assembly factor	-7,09	Energy metabolism			
PA0114	*senC*	SenC	-6,90	Hypothetical, unclassified, unknown			
PA0115		conserved hypothetical protein	-2,20	Hypothetical, unclassified, unknown			
PA0117		probable short chain dehydrogenase	3,36	Putative enzymes			
PA0119		probable dicarboxylate transporter	11,25	Transport of small molecules			
PA0122	*rahU*	rahU	-26,32	Adaptation, Protection			
PA0123		probable transcriptional regulator	6,76	Transcriptional regulators			
PA0124		ParE toxin	7,08	Hypothetical, unclassified, unknown			
PA0125		ParD antitoxin	6,77	Hypothetical, unclassified, unknown			
PA0128		conserved hypothetical protein	2,35	Hypothetical, unclassified, unknown			
PA0132	*bauA*	Beta-alanine:pyruvate transaminase	3,30	Carbon compound catabolism	Amino acid biosynthesis and metabolism		
PA0133	*bauR*	BauR	8,90	Transcriptional regulators	Carbon compound catabolism		
PA0134		probable guanine deaminase	5,13	Nucleotide biosynthesis and metabolism			
PA0135		hypothetical protein	11,10	Hypothetical, unclassified, unknown			
PA0136		probable ATP-binding component of ABC transporter	17,29	Transport of small molecules			
PA0137		probable permease of ABC transporter	21,39	Membrane proteins	Transport of small molecules		
PA0138		probable permease of ABC transporter	5,04	Membrane proteins	Transport of small molecules		
PA0140	*ahpF*	alkyl hydroperoxide reductase subunit F	4,39	Adaptation, Protection			
PA0141		conserved hypothetical protein	-7,46	Hypothetical, unclassified, unknown			
PA0142		hypothetical protein	2,02	Putative enzymes			
PA0143	*nuh*	purine nucleosidase Nuh	-5,95	Nucleotide biosynthesis and metabolism			
PA0145		hypothetical protein	2,43	Hypothetical, unclassified, unknown			
PA0146		conserved hypothetical protein	11,97	Hypothetical, unclassified, unknown			
PA0148		adenine deaminase	-2,38	Nucleotide biosynthesis and metabolism			
PA0150		anti-sigma factor	3,51	Membrane proteins	Transcriptional regulators		
PA0151		probable TonB-dependent receptor	4,12	Transport of small molecules			x
PA0153	*pcaH*	protocatechuate 3,4-dioxygenase, beta subunit	2,58	Carbon compound catabolism			
PA0154	*pcaG*	protocatechuate 3,4-dioxygenase, alpha subunit	2,72	Carbon compound catabolism			
PA0155	*pcaR*	transcriptional regulator PcaR	2,12	Transcriptional regulators	Carbon compound catabolism		
PA0158	*triC*	Resistance-Nodulation-Cell Division (RND) triclosan efflux transporter, TriC	-2,49	Antibiotic resistance and susceptibility	Transport of small molecules		x
PA0159		probable transcriptional regulator	-2,16	Transcriptional regulators			
PA0160		hypothetical protein	2,21	Hypothetical, unclassified, unknown			
PA0161		hypothetical protein	4,58	Hypothetical, unclassified, unknown			
PA0162	*opdC*	histidine porin OpdC	3,04	Membrane proteins	Transport of small molecules		
PA0164		probable gamma-glutamyltranspeptidase	3,59	Amino acid biosynthesis and metabolism			
PA0165		hypothetical protein	5,26	Hypothetical, unclassified, unknown			
PA0166		probable transporter	15,91	Membrane proteins	Transport of small molecules		
PA0167		probable transcriptional regulator	-3,12	Transcriptional regulators			
PA0168		conserved hypothetical protein	-3,56	Hypothetical, unclassified, unknown			
PA0172	*siaA*	SiaA	6,09	Motility and Attachment			
PA0173		probable methylesterase	-33,33	Adaptation, Protection	Chemotaxis		
PA0174		conserved hypothetical protein	-19,23	Hypothetical, unclassified, unknown			
PA0175	*cheR2*	CheR2	-43,48	Adaptation, Protection	Transcriptional regulators	Chemotaxis	
PA0176	*aer2*	aerotaxis transducer Aer2	-38,46	Adaptation, Protection	Chemotaxis		
PA0177		probable purine-binding chemotaxis protein	-21,28	Adaptation, Protection	Chemotaxis		
PA0178		probable two-component sensor	-10,31	Two-component regulatory systems	Adaptation, Protection	Chemotaxis	
PA0179		probable two-component response regulator	-6,41	Two-component regulatory systems	Adaptation, Protection	Chemotaxis	
PA0182		probable short-chain dehydrogenase	6,64	Putative enzymes			
PA0184		probable ATP-binding component of ABC transporter	2,36	Transport of small molecules			
PA0185		probable permease of ABC transporter	6,44	Membrane proteins	Transport of small molecules		
PA0186		probable binding protein component of ABC transporter	7,85	Transport of small molecules			
PA0187		hypothetical protein	-2,19	Hypothetical, unclassified, unknown			
PA0189	*opdI*	probable porin	8,90	Membrane proteins	Transport of small molecules		
PA0190		probable acid phosphatase	2,44	Putative enzymes			
PA0191		probable transcriptional regulator	4,33	Transcriptional regulators			
PA0192		probable TonB-dependent receptor	6,90	Membrane proteins	Transport of small molecules		
PA0193		hypothetical protein	-2,65	Putative enzymes			
PA0194		hypothetical protein	-2,03	Putative enzymes			
PA0196	*pntB*	pyridine nucleotide transhydrogenase, beta subunit	-2,59	Transport of small molecules	Energy metabolism		
PA0197	*tonB2*	TonB2	9,00	Transport of small molecules			
PA0198	*exbB1*	transport protein ExbB	3,40	Transport of small molecules			
PA0200		hypothetical protein	-3,82	Hypothetical, unclassified, unknown			
PA0201		hypothetical protein	4,45	Hypothetical, unclassified, unknown			
PA0204		probable permease of ABC transporter	14,10	Membrane proteins	Transport of small molecules		
PA0205		probable permease of ABC transporter	4,91	Membrane proteins	Transport of small molecules		
PA0206		probable ATP-binding component of ABC transporter	5,03	Transport of small molecules			
PA0207		probable transcriptional regulator	3,22	Transcriptional regulators			
PA0211	*mdcD*	malonate decarboxylase beta subunit	-2,13	Carbon compound catabolism			
PA0212	*mdcE*	malonate decarboxylase gamma subunit	-3,60	Carbon compound catabolism			
PA0213		hypothetical protein	-4,41	Carbon compound catabolism			
PA0214		probable acyl transferase	-5,46	Carbon compound catabolism			
PA0215		malonate transporter MadL	-3,64	Membrane proteins	Transport of small molecules		
PA0216		malonate transporter MadM	4,42	Membrane proteins	Transport of small molecules		
PA0218		probable transcriptional regulator	2,12	Transcriptional regulators			
PA0219		probable aldehyde dehydrogenase	4,15	Putative enzymes			
PA0220		amino acid APC family transporter	7,22	Transport of small molecules			
PA0222		hypothetical protein	2,60	Hypothetical, unclassified, unknown			
PA0226		probable CoA transferase, subunit A	25,63	Carbon compound catabolism			
PA0227		probable CoA transferase, subunit B	25,37	Carbon compound catabolism			
PA0228	*pcaF*	beta-ketoadipyl CoA thiolase PcaF	14,38	Fatty acid and phospholipid metabolism	Carbon compound catabolism		
PA0229	*pcaT*	dicarboxylic acid transporter PcaT	3,82	Membrane proteins	Transport of small molecules		
PA0234		hypothetical protein	2,58	Hypothetical, unclassified, unknown			
PA0235	*pcaK*	4-hydroxybenzoate transporter PcaK	15,07	Membrane proteins	Transport of small molecules	Carbon compound catabolism	
PA0237		probable oxidoreductase	2,51	Putative enzymes			
PA0239		hypothetical protein	2,10	Membrane proteins			
PA0240	*opdF*	probable porin	3,50	Membrane proteins	Transport of small molecules		
PA0241		probable major facilitator superfamily (MFS) transporter	4,81	Membrane proteins	Transport of small molecules		
PA0242		hypothetical protein	9,35	Putative enzymes			
PA0244		hypothetical protein	17,27	Putative enzymes			
PA0245	*aroQ2*	3-dehydroquinate dehydratase	15,67	Amino acid biosynthesis and metabolism			
PA0246		probable major facilitator superfamily (MFS) transporter	7,08	Membrane proteins	Transport of small molecules		
PA0247	*pobA*	p-hydroxybenzoate hydroxylase	5,81	Carbon compound catabolism			
PA0248		probable transcriptional regulator	2,10	Transcriptional regulators			
PA0250		conserved hypothetical protein	-2,74	Hypothetical, unclassified, unknown			
PA0251		hypothetical protein	7,02	Hypothetical, unclassified, unknown			
PA0252		hypothetical protein	9,99	Hypothetical, unclassified, unknown			
PA0253	*hudR*	HudR	2,80	Transcriptional regulators			
PA0254	*hudA*	HudA	4,30	Hypothetical, unclassified, unknown			
PA0257		hypothetical protein	3,35	Relative to phage, transposon, or plasmid			
PA0263.1		tRNA-Arg	12,71	Non-coding RNA gene			
PA0264		hypothetical protein	14,99	Hypothetical, unclassified, unknown			
PA0270		hypothetical protein	-2,39	Hypothetical, unclassified, unknown			
PA0271		hypothetical protein	-2,06	Hypothetical, unclassified, unknown			
PA0273		probable major facilitator superfamily (MFS) transporter	6,99	Membrane proteins	Transport of small molecules		
PA0276		hypothetical protein	4,07	Membrane proteins			
PA0277		Lipoprotein	9,79	Hypothetical, unclassified, unknown			
PA0280	*cysA*	sulfate transport protein CysA	-7,19	Transport of small molecules			
PA0281	*cysW*	sulfate transport protein CysW	-2,49	Membrane proteins	Transport of small molecules		
PA0284		hypothetical protein	2,28	Hypothetical, unclassified, unknown			
PA0286	*desA*	delta-9 fatty acid desaturase, DesA	-2,88	Fatty acid and phospholipid metabolism			
PA0287	*gpuP*	3-guanidinopropionate transport protein	8,04	Transport of small molecules			
PA0289	*gpuR*	transcriptional activator GpuR	-2,32	Transcriptional regulators			
PA0291	*oprE*	Anaerobically-induced outer membrane porin OprE precursor	-2,58	Membrane proteins	Transport of small molecules		
PA0294	*aguR*	transcriptional regulator AguR	-2,30	Transcriptional regulators			
PA0295		probable periplasmic polyamine binding protein	-5,99	Transport of small molecules			
PA0296	*spuI*	Glutamylpolyamine synthetase	-2,28	Putative enzymes	Carbon compound catabolism		
PA0297	*spuA*	probable glutamine amidotransferase	6,03	Carbon compound catabolism	Amino acid biosynthesis and metabolism		
PA0300	*spuD*	polyamine transport protein	-5,88	Transport of small molecules			
PA0301	*spuE*	polyamine transport protein	-5,75	Transport of small molecules			
PA0302	*spuF*	polyamine transport protein PotG	-5,41	Transport of small molecules			
PA0303	*spuG*	polyamine transport protein PotH	-6,29	Membrane proteins	Transport of small molecules		
PA0304	*spuH*	polyamine transport protein PotI	-3,30	Membrane proteins	Transport of small molecules		
PA0307		hypothetical protein	2,60	Hypothetical, unclassified, unknown			
PA0309		hypothetical protein	2,66	Hypothetical, unclassified, unknown			
PA0310		hypothetical protein	4,07	Hypothetical, unclassified, unknown			
PA0311		hypothetical protein	3,91	Hypothetical, unclassified, unknown			
PA0313		L-cysteine transporter of ABC system YecS	-2,26	Membrane proteins	Transport of small molecules		
PA0314		L-cysteine transporter of ABC system FliY	-4,27	Transport of small molecules			
PA0318		conserved hypothetical protein	-2,08	Putative enzymes			
PA0322		probable transporter	2,49	Membrane proteins	Transport of small molecules		
PA0324		probable permease of ABC transporter	6,84	Membrane proteins	Transport of small molecules		
PA0325		probable permease of ABC transporter	11,70	Membrane proteins	Transport of small molecules		
PA0326		probable ATP-binding component of ABC transporter	15,30	Transport of small molecules			
PA0328	*aaaA*	arginine-specific autotransporter of Pseudomonas aeruginosa, AaaA	7,75	Amino acid biosynthesis and metabolism			x
PA0332		hypothetical protein	2,02	Hypothetical, unclassified, unknown			
PA0333		hypothetical protein	5,46	Putative enzymes			
PA0335		hypothetical protein	-3,28	Hypothetical, unclassified, unknown			
PA0341	*lgt*	prolipoprotein diacylglyceryl transferase	-3,86	Fatty acid and phospholipid metabolism	Translation, post-translational modification, degradation		
PA0346		hypothetical protein	6,25	Hypothetical, unclassified, unknown			
PA0348		hypothetical protein	9,33	Hypothetical, unclassified, unknown			
PA0349		hypothetical protein	14,65	Membrane proteins			
PA0351		conserved hypothetical protein	2,57	Hypothetical, unclassified, unknown			
PA0352		probable transporter	3,74	Membrane proteins	Transport of small molecules		
PA0358		hypothetical protein	8,24	Hypothetical, unclassified, unknown			
PA0364	*laoA*	LaoA	-2,90	Putative enzymes			
PA0365	*laoB*	LaoB	-3,15	Membrane proteins			
PA0366	*laoC*	LaoC	-2,10	Putative enzymes			
PA0369		Uncharacterized protein	-4,69	Hypothetical, unclassified, unknown			
PA0370		conserved hypothetical protein	-5,88	Hypothetical, unclassified, unknown			
PA0371		hypothetical protein	-2,87	Hypothetical, unclassified, unknown			
PA0376	*rpoH*	sigma factor RpoH	4,03	Transcriptional regulators			
PA0377		hypothetical protein	2,01	Hypothetical, unclassified, unknown			
PA0378		probable transglycosylase	3,82	Cell Wall / LPS / Capsule			
PA0382	*micA*	DNA mismatch repair protein MicA	-2,51	DNA replication, recombination, modification and repair			
PA0384		hypothetical protein	-3,91	Hypothetical, unclassified, unknown			
PA0385		hypothetical protein	-2,00	Hypothetical, unclassified, unknown			
PA0387		conserved hypothetical protein	-9,62	Hypothetical, unclassified, unknown			
PA0388		hypothetical protein	-9,09	Hypothetical, unclassified, unknown			
PA0389		hypothetical protein	-5,15	Hypothetical, unclassified, unknown			
PA0391		hypothetical protein	-6,99	Hypothetical, unclassified, unknown			
PA0392		conserved hypothetical protein	-3,18	Hypothetical, unclassified, unknown			
PA0395	*pilT*	twitching motility protein PilT	-3,17	Cell Wall / LPS / Capsule	Motility and Attachment		x
PA0396	*pilU*	twitching motility protein PilU	-3,26	Motility and Attachment			x
PA0398		hypothetical protein	-2,13	Hypothetical, unclassified, unknown			
PA0399		cystathionine beta-synthase	-10,10	Amino acid biosynthesis and metabolism			
PA0400		probable cystathionine gamma-lyase	-12,82	Amino acid biosynthesis and metabolism			
PA0401		noncatalytic dihydroorotase-like protein	-4,88	Nucleotide biosynthesis and metabolism			
PA0402	*pyrB*	aspartate carbamoyltransferase	-4,29	Amino acid biosynthesis and metabolism	Nucleotide biosynthesis and metabolism		
PA0403	*pyrR*	transcriptional regulator PyrR	-3,95	Transcriptional regulators	Nucleotide biosynthesis and metabolism		
PA0404		conserved hypothetical protein	-2,08	Hypothetical, unclassified, unknown			
PA0405	*algH*	AlgH	-2,27	Transcriptional regulators			
PA0407	*gshB*	glutathione synthetase	-5,29	Amino acid biosynthesis and metabolism	Biosynthesis of cofactors, prosthetic groups and carrier		
PA0408	*pilG*	twitching motility protein PilG	-2,00	Two-component regulatory systems	Motility and Attachment	Chemotaxis	x
PA0409	*pilH*	twitching motility protein PilH	-2,36	Two-component regulatory systems	Motility and Attachment	Chemotaxis	x
PA0410	*pilI*	twitching motility protein PilI	-2,86	Motility and Attachment	Chemotaxis		x
PA0411	*pilJ*	twitching motility protein PilJ	-4,08	Motility and Attachment	Chemotaxis		x
PA0412	*pilK*	methyltransferase PilK	-3,77	Motility and Attachment	Chemotaxis		x
PA0413	*chpA*	component of chemotactic signal transduction system	-2,25	Two-component regulatory systems	Motility and Attachment	Chemotaxis	x
PA0414	*chpB*	probable methylesterase	-2,82	Chemotaxis			x
PA0417	*chpE*	probable chemotaxis protein	2,84	Chemotaxis			x
PA0420	*bioA*	adenosylmethionine-8-amino-7-oxononanoate aminotransferase	-2,05	Biosynthesis of cofactors, prosthetic groups and carrier			
PA0421		hypothetical protein	-2,18	Putative enzymes			
PA0423	*pasP*	PasP	-8,85	Secreted Factors (toxins, enzymes, alginate)			x
PA0424	*mexR*	multidrug resistance operon repressor MexR	6,17	Transcriptional regulators			
PA0426	*mexB*	Resistance-Nodulation-Cell Division (RND) multidrug efflux transporter MexB	-2,97	Membrane proteins	Antibiotic resistance and susceptibility	Transport of small molecules	
PA0427	*oprM*	Major intrinsic multiple antibiotic resistance efflux outer membrane protein OprM pre	-3,45	Membrane proteins	Antibiotic resistance and susceptibility	Transport of small molecules	
PA0428		probable ATP-dependent RNA helicase	-2,17	Transcription, RNA processing and degradation			
PA0429		hypothetical protein	-5,21	Hypothetical, unclassified, unknown			
PA0430	*metF*	5,10-methylenetetrahydrofolate reductase	-3,91	Amino acid biosynthesis and metabolism	Central intermediary metabolism		
PA0431		hypothetical protein	-3,17	Hypothetical, unclassified, unknown			
PA0432	*sahH*	S-adenosyl-L-homocysteine hydrolase	-2,21	Amino acid biosynthesis and metabolism			
PA0434		hypothetical protein	-2,24	Membrane proteins			
PA0436		probable transcriptional regulator	-2,34	Transcriptional regulators			
PA0437	*codA*	cytosine deaminase	-5,81	Nucleotide biosynthesis and metabolism			
PA0439		probable oxidoreductase	2,14	Putative enzymes			
PA0440		probable oxidoreductase	2,44	Putative enzymes			
PA0441	*dht*	dihydropyrimidinase	8,79	Nucleotide biosynthesis and metabolism			
PA0443		probable transporter	27,75	Membrane proteins	Transport of small molecules		
PA0444		N-carbamoyl-beta-alanine amidohydrolase	4,72	Nucleotide biosynthesis and metabolism			
PA0447	*gcdH*	glutaryl-CoA dehydrogenase	2,16	Fatty acid and phospholipid metabolism	Amino acid biosynthesis and metabolism	Carbon compound catabolism	
PA0448	*gcdR*	transcriptional regulator, GcdR	2,38	Transcriptional regulators			
PA0450		probable phosphate transporter	18,76	Membrane proteins	Transport of small molecules		
PA0452		probable stomatin-like protein	5,84	Membrane proteins			
PA0453		hypothetical protein	8,08	Hypothetical, unclassified, unknown			
PA0457.1		hypothetical membrane protein	-2,14	Hypothetical, unclassified, unknown			
PA0459		probable ClpA/B protease ATP binding subunit	-12,99	Translation, post-translational modification, degradation			
PA0462		hypothetical protein	5,93	Hypothetical, unclassified, unknown			
PA0463	*creB*	two-component response regulator CreB	8,46	Two-component regulatory systems	Transcriptional regulators		
PA0464	*creC*	two-component sensor CreC	8,24	Two-component regulatory systems			
PA0465	*creD*	inner membrane protein CreD	10,64	Membrane proteins			
PA0466		hypothetical protein	5,14	Hypothetical, unclassified, unknown			
PA0467		conserved hypothetical protein	-3,73	Hypothetical, unclassified, unknown			
PA0468		hypothetical protein	-5,75	Hypothetical, unclassified, unknown			
PA0469		hypothetical protein	2,78	Hypothetical, unclassified, unknown			
PA0470	*fiuA*	Ferrichrome receptor FiuA	-3,60	Membrane proteins	Transport of small molecules		
PA0471	*fiuR*	FiuR	-9,35	Transcriptional regulators			
PA0472	*fiuI*	FiuI	-8,70	Transcriptional regulators			
PA0473		probable glutathione S-transferase	-2,30	Putative enzymes			
PA0474		hypothetical protein	3,72	Hypothetical, unclassified, unknown			
PA0476		probable permease	11,12	Transport of small molecules			
PA0483		probable acetyltransferase	-4,12	Putative enzymes			
PA0484		conserved hypothetical protein	-7,30	Hypothetical, unclassified, unknown			
PA0488		Lipoprotein	5,95	Hypothetical, unclassified, unknown			
PA0489		probable phosphoribosyl transferase	27,39	Putative enzymes			
PA0490		hypothetical protein	3,54	Hypothetical, unclassified, unknown			
PA0492		conserved hypothetical protein	5,40	Hypothetical, unclassified, unknown			
PA0493		probable biotin-requiring enzyme	3,69	Putative enzymes			
PA0497		hypothetical protein	2,83	Hypothetical, unclassified, unknown			
PA0499		probable pili assembly chaperone	3,60	Motility and Attachment	Chaperones & heat shock proteins		
PA0501	*bioF*	8-amino-7-oxononanoate synthase	3,50	Biosynthesis of cofactors, prosthetic groups and carrier			
PA0506		probable acyl-CoA dehydrogenase	2,20	Putative enzymes			
PA0508		probable acyl-CoA dehydrogenase	3,14	Putative enzymes			

PA0509	*nirN*	NirN	-4,17	Biosynthesis of cofactors, prosthetic groups and carrier	Energy metabolism		
PA0510	*nirE*	NirE	-6,71	Biosynthesis of cofactors, prosthetic groups and carrier	Energy metabolism		
PA0511	*nirJ*	heme d1 biosynthesis protein NirJ	-5,75	Biosynthesis of cofactors, prosthetic groups and carrier	Energy metabolism		
PA0512	*nirH*	NirH	-3,02	Biosynthesis of cofactors, prosthetic groups and carrier	Energy metabolism		
PA0513	*nirG*	NirG	-17,54	Biosynthesis of cofactors, prosthetic groups and carrier	Energy metabolism	Transcriptional regulators	
PA0514	*nirL*	heme d1 biosynthesis protein NirL	-11,63	Biosynthesis of cofactors, prosthetic groups and carrier	Energy metabolism		
PA0515		probable transcriptional regulator	-6,25	Biosynthesis of cofactors, prosthetic groups and carrier	Energy metabolism	Transcriptional regulators	
PA0516	*nirF*	heme d1 biosynthesis protein NirF	-6,62	Biosynthesis of cofactors, prosthetic groups and carrier	Energy metabolism		
PA0517	*nirC*	probable c-type cytochrome precursor	-24,39	Biosynthesis of cofactors, prosthetic groups and carrier	Energy metabolism		
PA0518	*nirM*	cytochrome c-551 precursor	-21,74	Biosynthesis of cofactors, prosthetic groups and carrier	Energy metabolism		
PA0519	*nirS*	nitrite reductase precursor	-11,63	Energy metabolism			
PA0521		probable cytochrome c oxidase subunit	2,14	Energy metabolism			
PA0522		hypothetical protein	2,93	Hypothetical, unclassified, unknown			
PA0524	*norB*	nitric-oxide reductase subunit B	-3,48	Energy metabolism			
PA0526		hypothetical protein	-6,54	Hypothetical, unclassified, unknown			
PA0527	*dnr*	transcriptional regulator Dnr	-7,25	Transcriptional regulators			
PA0528		probable transcriptional regulator	3,35	Transcriptional regulators			
PA0529		conserved hypothetical protein	3,15	Hypothetical, unclassified, unknown			
PA0530		probable class III pyridoxal phosphate-dependent aminotransferase	3,24	Putative enzymes			
PA0531		probable glutamine amidotransferase	4,50	Putative enzymes			
PA0532		hypothetical protein	10,60	Hypothetical, unclassified, unknown			
PA0534	*pauB1*	FAD-dependent oxidoreductase	2,96	Putative enzymes	Carbon compound catabolism		
PA0535		probable transcriptional regulator	3,10	Transcriptional regulators			
PA0538	*dsbB*	disulfide bond formation protein	-2,77	Translation, post-translational modification, degradation	Chaperones & heat shock proteins		
PA0539		hypothetical protein	20,88	Membrane proteins			
PA0542		conserved hypothetical protein	-2,02	Hypothetical, unclassified, unknown			
PA0543		hypothetical protein	5,91	Hypothetical, unclassified, unknown			
PA0545		hypothetical protein	7,63	Putative enzymes			
PA0546	*metK*	methionine adenosyltransferase	-13,70	Amino acid biosynthesis and metabolism	Central intermediary metabolism		
PA0547		probable transcriptional regulator	-5,21	Transcriptional regulators			
PA0548	*tktA*	transketolase	-3,80	Energy metabolism			
PA0549		hypothetical protein	-2,46	Hypothetical, unclassified, unknown			
PA0550		conserved hypothetical protein	-5,43	Hypothetical, unclassified, unknown			
PA0562		probable hydrolase	2,07	Putative enzymes			
PA0563		conserved hypothetical protein	2,09	Hypothetical, unclassified, unknown			
PA0565		conserved hypothetical protein	-6,45	Hypothetical, unclassified, unknown			
PA0568		hypothetical protein	-2,97	Hypothetical, unclassified, unknown			
PA0569		hypothetical protein	-3,70	Hypothetical, unclassified, unknown			
PA0570		hypothetical protein	-2,19	Hypothetical, unclassified, unknown			
PA0571		hypothetical protein	-3,52	Hypothetical, unclassified, unknown			
PA0572	*impA*	hypothetical protein	-27,03	Hypothetical, unclassified, unknown			
PA0573		hypothetical protein	4,37	Hypothetical, unclassified, unknown			
PA0574		hypothetical protein	2,04	Hypothetical, unclassified, unknown			
PA0576	*rpoD*	sigma factor RpoD	2,11	Transcriptional regulators			
PA0577	*dnaG*	DNA primase	14,04	DNA replication, recombination, modification and repair			
PA0578		conserved hypothetical protein	8,74	Hypothetical, unclassified, unknown			
PA0580	*gcp*	O-sialoglycoprotein endopeptidase	5,34	Translation, post-translational modification, degradation			
PA0581		conserved hypothetical protein	2,32	Hypothetical, unclassified, unknown			
PA0582	*folB*	dihydroneopterin aldolase	5,32	Biosynthesis of cofactors, prosthetic groups and carrier			
PA0583		hypothetical protein	5,30	Biosynthesis of cofactors, prosthetic groups and carrier			
PA0585		Lipoprotein	-2,26	Hypothetical, unclassified, unknown			
PA0586		conserved hypothetical protein	-28,57	Hypothetical, unclassified, unknown			
PA0587		conserved hypothetical protein	-19,23	Hypothetical, unclassified, unknown			
PA0588		conserved hypothetical protein	-10,87	Hypothetical, unclassified, unknown			
PA0589		conserved hypothetical protein	-2,71	Energy metabolism			
PA0590	*apaH*	bis(5'-nucleosyl)-tetraphosphatase	-2,70	Nucleotide biosynthesis and metabolism			
PA0593	*pdxA*	pyridoxal phosphate biosynthetic protein PdxA	-7,19	Biosynthesis of cofactors, prosthetic groups and carrier			
PA0594	*surA*	peptidyl-prolyl cis-trans isomerase SurA	-6,25	Translation, post-translational modification, degradation	Adaptation, Protection	Chaperones & heat shock proteins	
PA0595	*lptD*	LPS-assembly protein LptD	-3,36	Adaptation, Protection			
PA0596		hypothetical protein	2,96	Hypothetical, unclassified, unknown			
PA0599		hypothetical protein	-2,19	Hypothetical, unclassified, unknown			
PA0600	*agtS*	two-component sensor, AgtS	3,38	Two-component regulatory systems			
PA0609	*trpE*	anthranilate synthetase component I	-2,37	Amino acid biosynthesis and metabolism	Energy metabolism		
PA0610	*prtN*	transcriptional regulator PrtN	9,05	Transcriptional regulators			
PA0611	*prtR*	transcriptional regulator PrtR	2,05	Transcriptional regulators			
PA0612	*ptrB*	repressor, PtrB	4,17	Transcriptional regulators			
PA0613		hypothetical protein	4,44	Hypothetical, unclassified, unknown			
PA0614		hypothetical protein	-4,42	Hypothetical, unclassified, unknown			
PA0615		hypothetical protein	-4,13	Hypothetical, unclassified, unknown			
PA0616		hypothetical protein	-3,11	Relative to phage, transposon, or plasmid			
PA0617		probable bacteriophage protein	-6,06	Relative to phage, transposon, or plasmid			
PA0618		probable bacteriophage protein	-5,41	Relative to phage, transposon, or plasmid			
PA0619		probable bacteriophage protein	-4,67	Relative to phage, transposon, or plasmid			
PA0620		probable bacteriophage protein	-4,55	Relative to phage, transposon, or plasmid			
PA0621		conserved hypothetical protein	-4,08	Relative to phage, transposon, or plasmid			
PA0622		probable bacteriophage protein	-7,25	Relative to phage, transposon, or plasmid			
PA0623		probable bacteriophage protein	-7,09	Relative to phage, transposon, or plasmid			
PA0624		hypothetical protein	-6,90	Relative to phage, transposon, or plasmid			
PA0625		hypothetical protein	-3,30	Relative to phage, transposon, or plasmid			
PA0626		hypothetical protein	-2,39	Relative to phage, transposon, or plasmid			
PA0627		conserved hypothetical protein	-2,54	Relative to phage, transposon, or plasmid			
PA0628		conserved hypothetical protein	-2,56	Relative to phage, transposon, or plasmid			
PA0629		conserved hypothetical protein	-3,98	Relative to phage, transposon, or plasmid			
PA0630		hypothetical protein	-3,91	Relative to phage, transposon, or plasmid			
PA0631		Lipoprotein	-3,62	Relative to phage, transposon, or plasmid			
PA0632		hypothetical protein	-4,93	Relative to phage, transposon, or plasmid			
PA0633		hypothetical protein	-7,87	Relative to phage, transposon, or plasmid			
PA0634		hypothetical protein	-5,81	Relative to phage, transposon, or plasmid			
PA0635		hypothetical protein	-5,68	Relative to phage, transposon, or plasmid			
PA0636		hypothetical protein	-4,52	Relative to phage, transposon, or plasmid			
PA0637		conserved hypothetical protein	-5,08	Relative to phage, transposon, or plasmid			
PA0638		probable bacteriophage protein	-5,38	Relative to phage, transposon, or plasmid			
PA0639		conserved hypothetical protein	-5,43	Relative to phage, transposon, or plasmid			
PA0640		probable bacteriophage protein	-4,85	Relative to phage, transposon, or plasmid			
PA0641		probable bacteriophage protein	-3,86	Relative to phage, transposon, or plasmid			
PA0642		hypothetical protein	-3,06	Relative to phage, transposon, or plasmid			
PA0643		hypothetical protein	-2,87	Relative to phage, transposon, or plasmid			
PA0644		hypothetical protein	-3,86	Relative to phage, transposon, or plasmid			
PA0645		hypothetical protein	-3,25	Relative to phage, transposon, or plasmid			
PA0646		hypothetical protein	-5,10	Relative to phage, transposon, or plasmid			
PA0647		hypothetical protein	-5,78	Relative to phage, transposon, or plasmid			
PA0648		hypothetical protein	-5,88	Relative to phage, transposon, or plasmid			
PA0652	*vfr*	transcriptional regulator Vfr	-4,39	Transcriptional regulators			x
PA0655	*ubiF*	hypothetical protein	-2,23	Hypothetical, unclassified, unknown			
PA0656		probable HIT family protein	-4,41	Putative enzymes			
PA0658		probable short-chain dehydrogenase	2,47	Putative enzymes			
PA0659		hypothetical protein	-2,95	Membrane proteins			
PA0660		NAD(P)H:quinone reductase	-6,58	Hypothetical, unclassified, unknown			
PA0661		conserved hypothetical protein	-2,66	Membrane proteins			
PA0665		conserved hypothetical protein	2,55	Hypothetical, unclassified, unknown			
PA0668.2		tRNA-Ile	18,76	Non-coding RNA gene			
PA0668.3		tRNA-Ala	8,52	Non-coding RNA gene			
PA0668.5		5S ribosomal RNA	6,26	Non-coding RNA gene			
PA0670		hypothetical protein	2,23	Hypothetical, unclassified, unknown			
PA0671		hypothetical protein	4,29	Hypothetical, unclassified, unknown			
PA0672	*hemO*	heme oxygenase	-2,18	Biosynthesis of cofactors, prosthetic groups and carrier			
PA0673		hypothetical protein	-3,46	Hypothetical, unclassified, unknown			
PA0674	*vreA*	VreA	7,06	Transcriptional regulators			
PA0675	*vreI*	ECF sigma factor, VreI	12,05	Transcriptional regulators			
PA0676	*vreR*	sigma factor regulator, VreR	4,01	Membrane proteins	Transcriptional regulators		
PA0677	*hxcW*	HxcW	3,89	Protein secretion/export apparatus			
PA0678	*hxcU*	HxcU	2,81	Protein secretion/export apparatus			
PA0679	*hxcP*	HxcP	2,60	Hypothetical, unclassified, unknown			
PA0680	*hxcV*	HxcV	5,09	Protein secretion/export apparatus			
PA0681	*hxcT*	HxcT	10,55	Protein secretion/export apparatus			
PA0682	*hxcX*	HxcX	25,02	Protein secretion/export apparatus			
PA0683	*hxcY*	HxcY	6,09	Protein secretion/export apparatus			
PA0684	*hxcZ*	HxcZ	10,33	Protein secretion/export apparatus			
PA0685	*hxcQ*	HxcQ secretin liporotein	13,41	Protein secretion/export apparatus			
PA0686	*hxcR*	HxcR	11,11	Protein secretion/export apparatus			
PA0687	*hxcS*	HxcS	18,82	Protein secretion/export apparatus			
PA0690	*pdtA*	phosphate depletion regulated TPS partner A, PdtA	6,13	Hypothetical, unclassified, unknown			x
PA0691	*phdA*	prevent-host-death protein A	9,09	Hypothetical, unclassified, unknown			
PA0692	*pdtB*	phosphate depletion regulated TPS partner B, PdtB	13,59	Hypothetical, unclassified, unknown			
PA0693	*exbB2*	transport protein ExbB2	3,01	Membrane proteins	Transport of small molecules		
PA0694	*exbD2*	transport protein ExbD	4,65	Membrane proteins	Transport of small molecules		
PA0695		hypothetical protein	4,88	Hypothetical, unclassified, unknown			
PA0704		probable amidase	-5,99	Putative enzymes			
PA0706	*cat*	chloramphenicol acetyltransferase	-2,48	Antibiotic resistance and susceptibility			
PA0707	*toxR*	transcriptional regulator ToxR	-3,04	Transcriptional regulators			
PA0711		hypothetical protein	7,49	Hypothetical, unclassified, unknown			
PA0712		hypothetical protein	4,95	Hypothetical, unclassified, unknown			
PA0713		hypothetical protein	-4,37	Hypothetical, unclassified, unknown			
PA0714.1	*phrD*	PhrD	14,75	Non-coding RNA gene			
PA0715		hypothetical protein	-2,67	Relative to phage, transposon, or plasmid			
PA0716		hypothetical protein	-3,70	Relative to phage, transposon, or plasmid			
PA0717		hypothetical protein of bacteriophage Pf1	8 180,44	Relative to phage, transposon, or plasmid			
PA0718		hypothetical protein of bacteriophage Pf1	11 362,12	Relative to phage, transposon, or plasmid			
PA0719		hypothetical protein of bacteriophage Pf1	7 965,00	Relative to phage, transposon, or plasmid			
PA0720		helix destabilizing protein of bacteriophage Pf1	6 316,21	Relative to phage, transposon, or plasmid	DNA replication, recombination, modification and repair		
PA0721		hypothetical protein of bacteriophage Pf1	4 090,89	Relative to phage, transposon, or plasmid			
PA0722		hypothetical protein of bacteriophage Pf1	5 689,38	Relative to phage, transposon, or plasmid			
PA0723	*coaB*	coat protein B of bacteriophage Pf1	2 078,30	Relative to phage, transposon, or plasmid			
PA0724		probable coat protein A of bacteriophage Pf1	3 178,13	Relative to phage, transposon, or plasmid			
PA0725		hypothetical protein of bacteriophage Pf1	1 711,45	Relative to phage, transposon, or plasmid			
PA0726		hypothetical protein of bacteriophage Pf1	304,06	Relative to phage, transposon, or plasmid			
PA0727		Pf replication initiator protein	56,10	Relative to phage, transposon, or plasmid			
PA0728		probable bacteriophage integrase	4,81	Relative to phage, transposon, or plasmid	Putative enzymes		
PA0729		hypothetical protein	10,69	Hypothetical, unclassified, unknown			
PA0730		probable transferase	-5,52	Putative enzymes			
PA0731		hypothetical protein	-4,74	Hypothetical, unclassified, unknown			
PA0732		hypothetical protein	-2,25	Hypothetical, unclassified, unknown			
PA0735		hypothetical protein	-2,58	Hypothetical, unclassified, unknown			
PA0736		hypothetical protein	-2,27	Membrane proteins			
PA0737		Lipoprotein	11,17	Hypothetical, unclassified, unknown			
PA0738		conserved hypothetical protein	11,84	Membrane proteins			
PA0740	*sdsA1*	SDS hydrolase SdsA1	26,34	Putative enzymes			
PA0741		conserved hypothetical protein	-2,30	Hypothetical, unclassified, unknown			
PA0743		probable 3-hydroxyisobutyrate dehydrogenase	-13,33	Carbon compound catabolism			
PA0744		probable enoyl-CoA hydratase/isomerase	-9,52	Putative enzymes			
PA0745	*dspI*	DspI	-8,20	Putative enzymes			
PA0746		probable acyl-CoA dehydrogenase	-7,87	Putative enzymes			
PA0747		probable aldehyde dehydrogenase	-4,00	Putative enzymes			
PA0749		hypothetical protein	2,33	Hypothetical, unclassified, unknown			
PA0751		conserved hypothetical protein	2,29	Membrane proteins			
PA0754		hypothetical protein	-2,03	Hypothetical, unclassified, unknown			
PA0755	*opdH*	cis-aconitate porin OpdH	7,10	Membrane proteins	Transport of small molecules		
PA0756		probable two-component response regulator	2,94	Two-component regulatory systems	Transcriptional regulators		
PA0757		probable two-component sensor	3,10	Two-component regulatory systems			
PA0758		hypothetical protein	-3,25	Hypothetical, unclassified, unknown			
PA0759		conserved hypothetical protein	-2,74	Hypothetical, unclassified, unknown			
PA0760		conserved hypothetical protein	2,14	Hypothetical, unclassified, unknown			
PA0762	*algU*	sigma factor AlgU	13,46	Transcriptional regulators			x
PA0763	*mucA*	anti-sigma factor MucA	9,89	Cell Wall / LPS / Capsule	Transcriptional regulators		x
PA0764	*mucB*	negative regulator for alginate biosynthesis MucB	11,42	Cell Wall / LPS / Capsule	Transcriptional regulators		x
PA0765	*mucC*	positive regulator for alginate biosynthesis MucC	9,41	Cell Wall / LPS / Capsule	Transcriptional regulators	Secreted Factors (toxins, enzymes, alginate)	x
PA0770	*rnc*	ribonuclease III	-2,07	Transcription, RNA processing and degradation			
PA0771	*era*	GTP-binding protein Era	-2,24	Translation, post-translational modification, degradation	Cell division		
PA0773	*pdxJ*	pyridoxal phosphate biosynthetic protein PdxJ	-3,09	Biosynthesis of cofactors, prosthetic groups and carrier			
PA0775		conserved hypothetical protein	2,46	Hypothetical, unclassified, unknown			
PA0776		Lipoprotein	-4,13	Hypothetical, unclassified, unknown			
PA0778	*icp*	inhibitor of cysteine peptidase	-3,10	Hypothetical, unclassified, unknown			
PA0779	*asrA*	AsrA	-2,81	Adaptation, Protection	Putative enzymes		
PA0782	*putA*	proline dehydrogenase PutA	8,27	Amino acid biosynthesis and metabolism			
PA0783	*putP*	sodium/proline symporter PutP	3,04	Membrane proteins	Transport of small molecules		
PA0784		probable transcriptional regulator	-4,20	Transcriptional regulators			
PA0785	*azoR1*	FMN-dependent NADH-azoreductase 1, AzoR1	2,62	Putative enzymes			
PA0786		probable transporter	2,48	Membrane proteins	Transport of small molecules		
PA0788		hypothetical protein	-2,68	Hypothetical, unclassified, unknown			
PA0789		probable amino acid permease	2,33	Membrane proteins	Transport of small molecules		
PA0790		hypothetical protein	3,01	Membrane proteins			
PA0792	*prpD*	propionate catabolic protein PrpD	-11,76	Carbon compound catabolism			
PA0793		hypothetical protein	-5,62	Hypothetical, unclassified, unknown			
PA0794		probable aconitate hydratase	-3,68	Energy metabolism			
PA0795	*prpC*	citrate synthase 2	-5,38	Carbon compound catabolism	Central intermediary metabolism		
PA0796	*prpB*	carboxyphosphonoenolpyruvate phosphonomutase	-3,94	Fatty acid and phospholipid metabolism	Carbon compound catabolism	Central intermediary metabolism	
PA0797		probable transcriptional regulator	2,22	Transcriptional regulators			
PA0798	*pmtA*	phospholipid methyltransferase	-2,47	Fatty acid and phospholipid metabolism			
PA0799		probable helicase	2,36	Putative enzymes			
PA0801		hypothetical protein	-3,30	Membrane proteins			
PA0802		hypothetical protein	-4,31	Membrane proteins			
PA0803		hypothetical protein	-5,00	Hypothetical, unclassified, unknown			
PA0804		probable oxidoreductase	-4,93	Putative enzymes			
PA0805		hypothetical protein	46,99	Hypothetical, unclassified, unknown			
PA0807	*ampDh3*	AmpDh3	-6,37	Antibiotic resistance and susceptibility			
PA0808		hypothetical protein	-3,51	Hypothetical, unclassified, unknown			
PA0809		probable transporter	8,81	Membrane proteins	Transport of small molecules		
PA0812		hypothetical protein	2,52	Hypothetical, unclassified, unknown			
PA0816		probable transcriptional regulator	2,75	Transcriptional regulators			
PA0817		probable ring-cleaving dioxygenase	-2,36	Putative enzymes			
PA0818		hypothetical protein	2,60	Hypothetical, unclassified, unknown			
PA0820		hypothetical protein	-2,13	Hypothetical, unclassified, unknown			
PA0821		hypothetical protein	-2,61	Hypothetical, unclassified, unknown			
PA0823		hypothetical protein	6,93	Hypothetical, unclassified, unknown			
PA0824		hypothetical protein	5,51	Hypothetical, unclassified, unknown			
PA0825		hypothetical protein	3,02	Hypothetical, unclassified, unknown			
PA0826.2	*ssrA*	tmRNA	-2,11	Amino acid biosynthesis and metabolism			
PA0828		probable transcriptional regulator	2,84	Transcriptional regulators			
PA0829		probable hydrolase	4,45	Putative enzymes			
PA0831	*oruR*	transcriptional regulator OruR	2,43	Transcriptional regulators			
PA0832		conserved hypothetical protein	-2,24	Hypothetical, unclassified, unknown			
PA0833		Lipoprotein	5,92	Membrane proteins			
PA0835	*pta*	phosphate acetyltransferase	-6,29	Carbon compound catabolism			
PA0836	*ackA*	acetate kinase	-4,95	Putative enzymes			
PA0836.1	*P5*	P5	108,54	Non-coding RNA gene			
PA0837	*slyD*	peptidyl-prolyl cis-trans isomerase SlyD	-2,30	Translation, post-translational modification, degradation	Chaperones & heat shock proteins		
PA0839		probable transcriptional regulator	6,12	Transcriptional regulators			
PA0840		probable oxidoreductase	2,18	Putative enzymes			
PA0842		probable glycosyl transferase	33,31	Putative enzymes			
PA0843	*plcR*	phospholipase accessory protein PlcR precursor	4,81	Secreted Factors (toxins, enzymes, alginate)			
PA0844	*plcH*	hemolytic phospholipase C precursor	2,94	Secreted Factors (toxins, enzymes, alginate)			x
PA0845	*cerN*	CerN	3,60	Hypothetical, unclassified, unknown			
PA0846		probable sulfate uptake protein	3,34	Transport of small molecules			
PA0850		hypothetical protein	-2,58	Hypothetical, unclassified, unknown			
PA0851		hypothetical protein	-7,87	Hypothetical, unclassified, unknown			
PA0852	*cbpD*	chitin-binding protein CbpD precursor	-33,33	Secreted Factors (toxins, enzymes, alginate)			
PA0856		hypothetical protein	-2,29	Hypothetical, unclassified, unknown			
PA0857	*bolA*	morphogene protein BolA	-2,28	Cell division			
PA0858		conserved hypothetical protein	2,38	Hypothetical, unclassified, unknown			
PA0860		probable ATP-binding/permease fusion ABC transporter	3,71	Membrane proteins	Transport of small molecules		
PA0862		hypothetical protein	-4,10	Hypothetical, unclassified, unknown			
PA0863		probable oxidoreductase	-4,46	Putative enzymes			
PA0865	*hpd*	4-hydroxyphenylpyruvate dioxygenase	-4,13	Amino acid biosynthesis and metabolism			
PA0866	*aroP2*	aromatic amino acid transport protein AroP2	3,29	Transport of small molecules			
PA0867	*mliC*	membrane-bound lysozyme inhibitor of c-type lysozyme MliC	-5,10	Adaptation, Protection			
PA0870	*phhC*	aromatic amino acid aminotransferase	-5,41	Amino acid biosynthesis and metabolism			
PA0871	*phhB*	pterin-4-alpha-carbinolamine dehydratase	-4,95	Amino acid biosynthesis and metabolism			
PA0872	*phhA*	phenylalanine-4-hydroxylase	-5,56	Amino acid biosynthesis and metabolism			
PA0874		hypothetical protein	17,72	Hypothetical, unclassified, unknown			
PA0877		probable transcriptional regulator	2,26	Transcriptional regulators			
PA0878		hypothetical protein	5,67	Hypothetical, unclassified, unknown			
PA0879		probable acyl-CoA dehydrogenase	2,11	Putative enzymes			
PA0881		hypothetical protein	2,03	Hypothetical, unclassified, unknown			
PA0882		hypothetical protein	3,53	Putative enzymes			
PA0883		probable acyl-CoA lyase beta chain	3,23	Putative enzymes			
PA0884		probable C4-dicarboxylate-binding periplasmic protein	5,75	Transport of small molecules			
PA0885		probable C4-dicarboxylate transporter	16,00	Transport of small molecules			
PA0886		probable C4-dicarboxylate transporter	2,92	Transport of small molecules			
PA0887	*acsA*	acetyl-coenzyme A synthetase	6,54	Carbon compound catabolism	Central intermediary metabolism		
PA0890	*aotM*	arginine/ornithine transport protein AotM	-2,36	Membrane proteins	Transport of small molecules		
PA0891		hypothetical protein	-5,00	Hypothetical, unclassified, unknown			
PA0892	*aotP*	arginine/ornithine transport protein AotP	-6,06	Transport of small molecules			
PA0893	*argR*	transcriptional regulator ArgR	-4,98	Amino acid biosynthesis and metabolism	Transcriptional regulators		
PA0894		hypothetical protein	2,40	Hypothetical, unclassified, unknown			
PA0895	*aruC*	N2-Succinylornithine 5-aminotransferase (SOAT) = N2-acetylornithine 5-aminotransfera	-3,56	Amino acid biosynthesis and metabolism			
PA0896	*aruF*	subunit I of arginine N2-succinyltransferase = ornithine N2-succinyltransferase	-2,80	Amino acid biosynthesis and metabolism			
PA0897	*aruG*	subunit II of arginine N2-succinyltransferase = ornithine N2-succinyltransferase	-3,33	Amino acid biosynthesis and metabolism			
PA0898	*aruD*	N-Succinylglutamate 5-semialdehyde dehydrogenase	-4,59	Amino acid biosynthesis and metabolism			
PA0899	*aruB*	N2-Succinylarginine dihydrolase	-8,06	Amino acid biosynthesis and metabolism			
PA0900		hypothetical protein	-5,00	Hypothetical, unclassified, unknown			
PA0901	*aruE*	N-Succinylglutamate desuccinylase	-4,85	Amino acid biosynthesis and metabolism			
PA0902		hypothetical protein	-2,11	Hypothetical, unclassified, unknown			
PA0904	*lysC*	aspartate kinase alpha and beta chain	-2,20	Amino acid biosynthesis and metabolism			
PA0905	*rsmA*	RsmA	-7,19	Translation, post-translational modification, degradation	Adaptation, Protection		x
PA0905.1		tRNA-Ser	-3,89	Non-coding RNA gene			
PA0905.3		tRNA-Arg	2,81	Non-coding RNA gene			
PA0907	*alpA*	lysis phenotype activator, AlpA	8,00	Hypothetical, unclassified, unknown			
PA0908	*alpB*	AlpB	3,18	Antibiotic resistance and susceptibility			
PA0912		hypothetical protein	2,95	Hypothetical, unclassified, unknown			
PA0913	*mgtE*	MgtE	12,97	Transport of small molecules			
PA0914		hypothetical protein	2,62	Hypothetical, unclassified, unknown			

PA0918		cytochrome b561	-4,85	Energy metabolism			
PA0919		alanyl-phosphatidylglycerol hydrolase	-4,05	Hypothetical, unclassified, unknown			
PA0922		hypothetical protein	11,12	Hypothetical, unclassified, unknown			
PA0922.1		tRNA-Met	24,25	Non-coding RNA gene			
PA0923	dinB	DNA Polymerase IV, DinB	12,74	DNA replication, recombination, modification and repair	Adaptation, Protection		
PA0924		hypothetical protein	2,02	Membrane proteins			
PA0926		hypothetical protein	-2,54	Hypothetical, unclassified, unknown			
PA0927	ldhA	D-lactate dehydrogenase (fermentative)	-8,62	Energy metabolism	Carbon compound catabolism	Central intermediary metabolism	
PA0928	gacS	sensor/response regulator hybrid	-2,24	Two-component regulatory systems			x
PA0931	pirA	ferric enterobactin receptor PirA	-2,92	Transport of small molecules			
PA0936	lpxO2	lipopolysaccharide biosynthetic protein LpxO2	-2,15	Cell Wall / LPS / Capsule	Putative enzymes		
PA0939		hypothetical protein	4,36	Hypothetical, unclassified, unknown			
PA0941		hypothetical protein	2,42	Hypothetical, unclassified, unknown			
PA0942		probable transcriptional regulator	3,15	Transcriptional regulators			
PA0946		hypothetical protein	-2,33	Hypothetical, unclassified, unknown			
PA0947		conserved hypothetical protein	3,07	Hypothetical, unclassified, unknown			
PA0950		probable arsenate reductase	2,88	Adaptation, Protection	Transport of small molecules		
PA0955		Lipoprotein	-3,18	Hypothetical, unclassified, unknown			
PA0956	proS	prolyl-tRNA synthetase	-2,42	Translation, post-translational modification, degradation	Amino acid biosynthesis and metabolism		
PA0957		hypothetical protein	2,61	Hypothetical, unclassified, unknown			
PA0958	oprD	Basic amino acid, basic peptide and imipenem outer membrane porin OprD precursor	-6,80	Transport of small molecules			
PA0961		probable cold-shock protein	-2,11	Transcriptional regulators			
PA0967	ruvB	Holliday junction DNA helicase RuvB	3,68	DNA replication, recombination, modification and repair			
PA0975		probable radical activating enzyme	24,88	Putative enzymes			
PA0976		conserved hypothetical protein	10,75	Hypothetical, unclassified, unknown			
PA0976.1		tRNA-Lys	9,99	Non-coding RNA gene			
PA0977		hypothetical protein	5,29	Hypothetical, unclassified, unknown			
PA0978		conserved hypothetical protein	4,22	Relative to phage, transposon, or plasmid			
PA0979		conserved hypothetical protein	2,67	Relative to phage, transposon, or plasmid			
PA0980		hypothetical protein	32,21	Hypothetical, unclassified, unknown			
PA0981		hypothetical protein	4,36	Hypothetical, unclassified, unknown			
PA0983		conserved hypothetical protein	2,14	Hypothetical, unclassified, unknown			
PA0984		colicin immunity protein	-2,58	Membrane proteins	Secreted Factors (toxins, enzymes, alginate)		
PA0985	pyoS5	pyocin S5	-8,13	Membrane proteins	Secreted Factors (toxins, enzymes, alginate)		
PA0986		conserved hypothetical protein	8,79	Relative to phage, transposon, or plasmid			
PA0987		conserved hypothetical protein	12,12	Relative to phage, transposon, or plasmid			
PA0988		hypothetical protein	-2,43	Hypothetical, unclassified, unknown			
PA0990		conserved hypothetical protein	3,98	Hypothetical, unclassified, unknown			
PA0991	hptA	Histidine phosphotransfer protein HptA	-2,02	Two-component regulatory systems			
PA0993	cupC2	chaperone CupC2	7,80	Motility and Attachment	Chaperones & heat shock proteins		
PA0994	cupC3	usher CupC3	2,99	Motility and Attachment			
PA0996	pqsA	PqsA	-12,50	Biosynthesis of cofactors, prosthetic groups and carrier			x
PA0997	pqsB	PqsB	-21,74	Biosynthesis of cofactors, prosthetic groups and carrier			
PA0998	pqsC	PqsC	-21,74	Biosynthesis of cofactors, prosthetic groups and carrier			
PA0999	pqsD	3-oxoacyl-[acyl-carrier-protein] synthase III	-13,33	Biosynthesis of cofactors, prosthetic groups and carrier			
PA1000	pqsE	Quinolone signal response protein	-7,75	Biosynthesis of cofactors, prosthetic groups and carrier			
PA1001	phnA	anthranilate synthase component I	-6,41	Adaptation, Protection			
PA1002	phnB	anthranilate synthase component II	-9,17	Amino acid biosynthesis and metabolism	Adaptation, Protection		
PA1003	mvfR	Transcriptional regulator MvfR	-4,72	Biosynthesis of cofactors, prosthetic groups and carrier	Transcriptional regulators		
PA1004	nadA		3,24	Amino acid biosynthesis and metabolism	Biosynthesis of cofactors, prosthetic groups and carrier		
PA1005		conserved hypothetical protein	-4,61	Hypothetical, unclassified, unknown			
PA1006		Protein PA1006	6,69	Hypothetical, unclassified, unknown			x
PA1007		conserved hypothetical protein	9,00	Membrane proteins			
PA1008	bcp	bacterioferritin comigratory protein	-9,52	Adaptation, Protection			
PA1009		hypothetical protein	-4,22	Hypothetical, unclassified, unknown			x
PA1011		Lipoprotein	-3,29	Hypothetical, unclassified, unknown			
PA1013	purC	phosphoribosylaminoimidazole-succinocarboxamide synthase	-4,44	Nucleotide biosynthesis and metabolism			
PA1013.1		tRNA-Ser	-4,10	Non-coding RNA gene			
PA1016		hypothetical protein	9,62	Hypothetical, unclassified, unknown			
PA1017	pauA	pimeloyl-CoA synthetase	8,73	Biosynthesis of cofactors, prosthetic groups and carrier			
PA1018		hypothetical protein	8,05	Hypothetical, unclassified, unknown			
PA1019	mucK	cis,cis-muconate transporter MucK	13,12	Membrane proteins	Transport of small molecules		
PA1023		probable short-chain dehydrogenase	-4,78	Putative enzymes			
PA1024		NADH:quinone reductase	-4,27	Hypothetical, unclassified, unknown			
PA1025	opdD	probable porin	2,04	Membrane proteins	Transport of small molecules		
PA1029		hypothetical protein	8,62	Hypothetical, unclassified, unknown			
PA1030		hypothetical protein	9,87	Hypothetical, unclassified, unknown			
PA1030.1	P8	P8	17,50	Non-coding RNA gene			
PA1031		conserved hypothetical protein	6,73	Hypothetical, unclassified, unknown			
PA1032	quiP	QuiP lipoprotein	-3,12	Antibiotic resistance and susceptibility	Putative enzymes	Carbon compound catabolism	
PA1033		probable glutathione S-transferase	-4,93	Putative enzymes			
PA1034		hypothetical protein	-2,48	Hypothetical, unclassified, unknown			
PA1035		Lipoprotein	-2,32	Hypothetical, unclassified, unknown			
PA1037		conserved hypothetical protein	2,81	Hypothetical, unclassified, unknown			
PA1038		hypothetical protein	-5,38	Hypothetical, unclassified, unknown			
PA1039		conserved hypothetical protein	-5,62	Hypothetical, unclassified, unknown			
PA1040		hypothetical protein	-3,51	Hypothetical, unclassified, unknown			
PA1041		Lipoprotein	-15,63	Membrane proteins	Transport of small molecules		
PA1042		conserved hypothetical protein	-2,39	Membrane proteins			
PA1044		hypothetical protein	2,97	Membrane proteins			
PA1047		probable esterase	-4,65	Putative enzymes			
PA1048		probable outer membrane lipoprotein precursor	-8,13	Membrane proteins	Transport of small molecules		
PA1049	pdxH	pyridoxine 5'-phosphate oxidase	-4,48	Biosynthesis of cofactors, prosthetic groups and carrier			
PA1050		conserved hypothetical protein	-3,29	Hypothetical, unclassified, unknown			
PA1051		probable transporter	3,53	Membrane proteins	Transport of small molecules		
PA1055	shaB	ShaB	-3,01	Membrane proteins	Transport of small molecules		
PA1056	shaC	ShaC	-2,23	Membrane proteins	Transport of small molecules	Putative enzymes	
PA1057	shaD	ShaD	-2,84	Membrane proteins	Transport of small molecules		
PA1058	shaE	ShaE	-3,06	Membrane proteins	Transport of small molecules		
PA1059	shaF	ShaF	-5,68	Membrane proteins	Transport of small molecules		
PA1060		hypothetical protein	-2,79	Membrane proteins			
PA1061		conserved hypothetical protein	-7,87	Putative enzymes			
PA1062		hypothetical protein	-7,63	Putative enzymes			
PA1063		hypothetical protein	-6,41	Hypothetical, unclassified, unknown			
PA1064		Lipoprotein	-2,39	Hypothetical, unclassified, unknown			
PA1066		probable short-chain dehydrogenase	-2,14	Putative enzymes			
PA1067		probable transcriptional regulator	2,63	Transcriptional regulators			
PA1068		probable heat shock protein (hsp90 family)	2,63	Chaperones & heat shock proteins			
PA1070	braG	branched-chain amino acid transport protein BraG	-5,13	Transport of small molecules			
PA1071	braF	branched-chain amino acid transport protein BraF	-6,41	Transport of small molecules			
PA1072	braE	branched-chain amino acid transport protein BraE	-4,20	Membrane proteins	Transport of small molecules		
PA1076		hypothetical protein	-3,45	Hypothetical, unclassified, unknown			
PA1077	flgB	flagellar basal-body rod protein FlgB	-2,54	Cell Wall / LPS / Capsule	Motility and Attachment		x
PA1078	flgC	flagellar basal-body rod protein FlgC	-2,61	Cell Wall / LPS / Capsule	Motility and Attachment		x
PA1079	flgD	flagellar basal-body rod modification protein FlgD	-3,62	Cell Wall / LPS / Capsule	Motility and Attachment		x
PA1080	flgE	flagellar hook protein FlgE	-9,01	Cell Wall / LPS / Capsule	Motility and Attachment		x
PA1085	flgJ	flagellar protein FlgJ	-2,88	Cell Wall / LPS / Capsule	Motility and Attachment		x
PA1086	flgK	flagellar hook-associated protein 1 FlgK	-10,99	Cell Wall / LPS / Capsule	Motility and Attachment		x
PA1087	flgL	flagellar hook-associated protein type 3 FlgL	-2,22	Cell Wall / LPS / Capsule	Motility and Attachment		x
PA1088		hypothetical protein	-3,56	Hypothetical, unclassified, unknown			
PA1089		conserved hypothetical protein	-6,67	Hypothetical, unclassified, unknown			
PA1090		hypothetical protein	-5,03	Hypothetical, unclassified, unknown			
PA1091	fgtA	flagellar glycosyl transferase, FgtA	-5,68	Hypothetical, unclassified, unknown			
PA1095	fliS	hypothetical protein	2,94	Hypothetical, unclassified, unknown			x
PA1096	fliT	hypothetical protein	4,12	Hypothetical, unclassified, unknown			x
PA1098	fleS	two-component sensor	8,17	Two-component regulatory systems			x
PA1099	fleR	two-component response regulator	2,58	Two-component regulatory systems	Transcriptional regulators	Motility and Attachment	x
PA1100	fliE	flagellar hook-basal body complex protein FliE	8,46	Cell Wall / LPS / Capsule	Motility and Attachment		x
PA1101	fliF	Flagella M-ring outer membrane protein precursor	7,93	Cell Wall / LPS / Capsule	Motility and Attachment		x
PA1102	fliG	flagellar motor switch protein FliG	3,05	Cell Wall / LPS / Capsule	Motility and Attachment		x
PA1103	fliH	probable flagellar assembly protein	2,75	Cell Wall / LPS / Capsule	Motility and Attachment		x
PA1104	fliI	flagellum-specific ATP synthase FliI	2,38	Energy metabolism	Motility and Attachment		x
PA1105	fliJ	flagellar protein FliJ	3,78	Cell Wall / LPS / Capsule	Motility and Attachment		x
PA1106		hypothetical protein	-2,56	Hypothetical, unclassified, unknown			
PA1108		probable major facilitator superfamily (MFS) transporter	10,54	Membrane proteins	Transport of small molecules		
PA1109		probable transcriptional regulator	7,03	Transcriptional regulators			
PA1111		hypothetical protein	12,19	Hypothetical, unclassified, unknown			
PA1114		hypothetical protein	6,07	Hypothetical, unclassified, unknown			
PA1115		hypothetical protein	2,38	Membrane proteins			
PA1116		hypothetical protein	3,68	Hypothetical, unclassified, unknown			
PA1119	yfiB	YfiB lipoprotein	-12,05	Membrane proteins	Cell Wall / LPS / Capsule		
PA1123		hypothetical protein	-6,99	Hypothetical, unclassified, unknown			
PA1124	dgt	deoxyguanosinetriphosphate triphosphohydrolase	-2,73	Nucleotide biosynthesis and metabolism			
PA1125		probable cobalamin biosynthetic protein	-2,45	Biosynthesis of cofactors, prosthetic groups and carrier			
PA1127		probable oxidoreductase	-3,48	Adaptation, Protection	Putative enzymes		
PA1130	rhlC	rhamnosyltransferase 2	-10,87	Cell Wall / LPS / Capsule	Adaptation, Protection		x
PA1132		hypothetical protein	6,28	Hypothetical, unclassified, unknown			
PA1134		hypothetical protein	-4,63	Hypothetical, unclassified, unknown			
PA1135		conserved hypothetical protein	-7,41	Hypothetical, unclassified, unknown			
PA1136		probable transcriptional regulator	3,87	Transcriptional regulators			
PA1138		probable transcriptional regulator	3,57	Transcriptional regulators			
PA1139		hypothetical protein	6,63	Hypothetical, unclassified, unknown			
PA1140		conserved hypothetical protein	-2,18	Hypothetical, unclassified, unknown			
PA1144		probable major facilitator superfamily (MFS) transporter	6,61	Membrane proteins	Transport of small molecules		
PA1146		probable iron-containing alcohol dehydrogenase	4,20	Carbon compound catabolism			
PA1147		probable amino acid permease	6,45	Membrane proteins	Transport of small molecules		
PA1152		hypothetical protein	2,61	Hypothetical, unclassified, unknown			
PA1154		conserved hypothetical protein	3,98	Hypothetical, unclassified, unknown			
PA1155	nrdB	NrdB, tyrosyl radical-harboring component of class Ia ribonucleotide reductase	-10,20	Nucleotide biosynthesis and metabolism			
PA1156	nrdA	NrdA, catalytic component of class Ia ribonucleotide reductase	-5,92	Nucleotide biosynthesis and metabolism			
PA1157		probable two-component response regulator	2,65	Two-component regulatory systems	Transcriptional regulators		x
PA1158		probable two-component sensor	4,65	Two-component regulatory systems			
PA1159		probable cold-shock protein	-4,44	Adaptation, Protection	Transcriptional regulators		
PA1160		hypothetical protein	-3,06	Hypothetical, unclassified, unknown			
PA1161	rrmA	rRNA methyltransferase	-3,47	Transcription, RNA processing and degradation			
PA1164		conserved hypothetical protein	6,73	Hypothetical, unclassified, unknown			
PA1166		hypothetical protein	-2,36	Hypothetical, unclassified, unknown			
PA1167		hypothetical protein	-5,38	Hypothetical, unclassified, unknown			
PA1168		hypothetical protein	-2,10	Hypothetical, unclassified, unknown			
PA1170		conserved hypothetical protein	12,73	Membrane proteins			
PA1172	napC	cytochrome c-type protein NapC	-19,23	Energy metabolism			
PA1173	napB	cytochrome c-type protein NapB precursor	-11,11	Energy metabolism			
PA1174	napA	periplasmic nitrate reductase protein NapA	-7,09	Energy metabolism			
PA1175	napD	NapD protein of periplasmic nitrate reductase	-2,56	Energy metabolism			
PA1176	napF	ferredoxin protein NapF	-3,62	Energy metabolism			
PA1183	dctA	C4-dicarboxylate transport protein	2,77	Transport of small molecules			
PA1187		probable acyl-CoA dehydrogenase	2,98	Putative enzymes			
PA1194		probable amino acid permease	18,00	Membrane proteins	Transport of small molecules		
PA1195	ddaH	dimethylarginine dimethylaminohydrolase DdaH	12,24	Amino acid biosynthesis and metabolism			
PA1196	ddaR	transcriptional regulator DdaR	3,23	Transcriptional regulators			
PA1197		hypothetical protein	-2,21	Hypothetical, unclassified, unknown			
PA1202		probable hydrolase	-7,52	Putative enzymes			
PA1203		hypothetical protein	-3,80	Hypothetical, unclassified, unknown			
PA1204		NAD(P)H quinone oxidoreductase	-3,85	Hypothetical, unclassified, unknown			
PA1205		conserved hypothetical protein	-4,83	Hypothetical, unclassified, unknown			
PA1206		hypothetical protein	2,36	Hypothetical, unclassified, unknown			
PA1207	kefB	glutathione-regulated potassium-efflux system protein KefB	2,61	Transport of small molecules			
PA1208		conserved hypothetical protein	2,03	Hypothetical, unclassified, unknown			
PA1211		hypothetical protein	-7,69	Hypothetical, unclassified, unknown			
PA1212		probable major facilitator superfamily (MFS) transporter	-19,61	Membrane proteins	Transport of small molecules		
PA1213		hypothetical protein	-29,41	Hypothetical, unclassified, unknown			
PA1214		hypothetical protein	-15,38	Hypothetical, unclassified, unknown			
PA1215		hypothetical protein	-20,00	Hypothetical, unclassified, unknown			
PA1216		hypothetical protein	-41,67	Hypothetical, unclassified, unknown			
PA1217		probable 2-isopropylmalate synthase	-35,71	Amino acid biosynthesis and metabolism			
PA1218		hypothetical protein	-34,48	Hypothetical, unclassified, unknown			
PA1219		hypothetical protein	-7,94	Hypothetical, unclassified, unknown			
PA1220		hypothetical protein	-8,70	Hypothetical, unclassified, unknown			
PA1221		hypothetical protein	-7,69	Hypothetical, unclassified, unknown			
PA1224		probable NAD(P)H dehydrogenase	3,40	Putative enzymes			
PA1228		hypothetical protein	6,22	Hypothetical, unclassified, unknown			
PA1229		probable transcriptional regulator	3,24	Transcriptional regulators			
PA1230		hypothetical protein	25,64	Membrane proteins			
PA1231	hlyD	Lipoprotein	8,50	Membrane proteins			
PA1232		hypothetical protein	4,61	Membrane proteins			
PA1234		hypothetical protein	-2,13	Hypothetical, unclassified, unknown			
PA1236		probable major facilitator superfamily (MFS) transporter	9,05	Membrane proteins	Transport of small molecules		
PA1237		probable multidrug resistance efflux pump	12,29	Antibiotic resistance and susceptibility	Transport of small molecules		
PA1238	opmJ	probable outer membrane component of multidrug efflux pump	9,07	Antibiotic resistance and susceptibility	Transport of small molecules		
PA1240		probable enoyl-CoA hydratase/isomerase	-2,75	Putative enzymes			
PA1241		probable transcriptional regulator	4,20	Transcriptional regulators			
PA1242	sprP	SprP	8,42	Translation, post-translational modification, degradation	Motility and Attachment		
PA1243		probable sensor/response regulator hybrid	-2,04	Two-component regulatory systems			
PA1244	qslA	QslA	-5,68	Hypothetical, unclassified, unknown			
PA1245	aprX	AprX	-3,94	Secreted Factors (toxins, enzymes, alginate)	Membrane proteins		
PA1246	aprD	alkaline protease secretion protein AprD	-4,15	Secreted Factors (toxins, enzymes, alginate)	Protein secretion/export apparatus		
PA1247	aprE	alkaline protease secretion protein AprE	-7,09	Secreted Factors (toxins, enzymes, alginate)	Protein secretion/export apparatus		
PA1248	aprF	Alkaline protease secretion outer membrane protein AprF precursor	-10,53	Secreted Factors (toxins, enzymes, alginate)	Protein secretion/export apparatus		x
PA1249	aprA	alkaline metalloproteinase precursor	-45,45	Secreted Factors (toxins, enzymes, alginate)			x
PA1250	aprI	alkaline proteinase inhibitor AprI	-4,12	Secreted Factors (toxins, enzymes, alginate)			
PA1251		probable chemotaxis transducer	2,02	Adaptation, Protection	Chemotaxis		
PA1253	lhpG	alpha-ketoglutaric semialdehyde dehydrogenase, LhpG	4,63	Amino acid biosynthesis and metabolism	Putative enzymes		
PA1254	lhpC	delta1-pyrroline-4-hydroxy-2-carboxylate deaminase, LphC	5,02	Amino acid biosynthesis and metabolism	Putative enzymes		
PA1258	lhpM	Permease of ABC transporter, LhpM	6,17	Amino acid biosynthesis and metabolism	Membrane proteins	Transport of small molecules	
PA1259	lhpH	LhpH	9,73	Amino acid biosynthesis and metabolism			
PA1260	lhpP	ABC transporter periplasmic-binding protein, LhpP	10,74	Amino acid biosynthesis and metabolism	Transport of small molecules		
PA1262		probable major facilitator superfamily (MFS) transporter	7,26	Membrane proteins	Transport of small molecules		
PA1263		hypothetical protein	2,72	Hypothetical, unclassified, unknown			
PA1265		hypothetical protein	21,37	Membrane proteins			
PA1266	lhpE	D-hydroxyproline dehydrogenase alpha-subunit, LphE	2,69	Amino acid biosynthesis and metabolism	Putative enzymes		
PA1267	lhpB	D-hydroxyproline dehydrogenase beta-subunit, LphB	2,31	Amino acid biosynthesis and metabolism			
PA1268	lhpA	Hydroxyproline 2-epimerase, LhpA	3,49	Amino acid biosynthesis and metabolism			
PA1270		hypothetical protein	11,27	Hypothetical, unclassified, unknown			
PA1271		probable tonB-dependent receptor	4,95	Transport of small molecules			
PA1282		probable major facilitator superfamily (MFS) transporter	5,60	Membrane proteins	Transport of small molecules		
PA1286		probable major facilitator superfamily (MFS) transporter	5,74	Membrane proteins	Transport of small molecules		
PA1287		probable glutathione peroxidase	-2,95	Putative enzymes			
PA1288		probable outer membrane protein precursor	-2,34	Membrane proteins	Transport of small molecules		
PA1290		probable transcriptional regulator	4,47	Transcriptional regulators			
PA1291		hypothetical protein	7,42	Hypothetical, unclassified, unknown			
PA1292		probable 3-mercaptopyruvate sulfurtransferase	-5,59	Putative enzymes			
PA1293		hypothetical protein	-3,83	Hypothetical, unclassified, unknown			
PA1296		probable 2-hydroxyacid dehydrogenase	20,47	Putative enzymes			
PA1297		probable metal transporter	7,59	Membrane proteins	Transport of small molecules		
PA1298		conserved hypothetical protein	16,32	Hypothetical, unclassified, unknown			
PA1299		conserved hypothetical protein	13,00	Hypothetical, unclassified, unknown			
PA1300		ECF sigma factor	4,41	Transcriptional regulators			
PA1301		probable transmembrane sensor	4,55	Membrane proteins	Transcriptional regulators		
PA1302		probable heme utilization protein precursor	5,94	Membrane proteins	Transport of small molecules		
PA1303		signal peptidase	-2,17	Putative enzymes			
PA1304		probable oligopeptidase	-2,40	Putative enzymes			
PA1305		hypothetical protein	2,30	Membrane proteins			
PA1306		probable HIT family protein	2,05	Putative enzymes			
PA1310	phnW	2-aminoethylphosphonate:pyruvate aminotransferase	5,20	Carbon compound catabolism			
PA1313		probable major facilitator superfamily (MFS) transporter	4,65	Membrane proteins	Transport of small molecules		
PA1317	cyoA	cytochrome o ubiquinol oxidase subunit II	-3,70	Energy metabolism			
PA1318	cyoB	cytochrome o ubiquinol oxidase subunit I	-9,17	Energy metabolism			
PA1319	cyoC	cytochrome o ubiquinol oxidase subunit III	-7,30	Energy metabolism			
PA1320	cyoD	cytochrome o ubiquinol oxidase subunit IV	-5,56	Energy metabolism			
PA1321	cyoE	cytochrome o ubiquinol oxidase protein CyoE	-4,78	Energy metabolism			
PA1323		hypothetical protein	-4,42	Hypothetical, unclassified, unknown			
PA1324		Lipoprotein	-6,67	Hypothetical, unclassified, unknown			
PA1324.1	P9	P9	-8,13	Non-coding RNA gene			
PA1325		conserved hypothetical protein	2,23	Hypothetical, unclassified, unknown			
PA1327		probable protease	-2,95	Putative enzymes			
PA1329		conserved hypothetical protein	6,57	Hypothetical, unclassified, unknown			
PA1332		hypothetical protein	35,07	Hypothetical, unclassified, unknown			
PA1333		Lipoprotein	15,90	Hypothetical, unclassified, unknown			
PA1335	aauR	AauR	-3,66	Two-component regulatory systems	Transcriptional regulators		
PA1337	ansB	glutaminase-asparaginase	-11,11	Amino acid biosynthesis and metabolism			
PA1338	ggt	gamma-glutamyltranspeptidase precursor	-4,05	Amino acid biosynthesis and metabolism	Adaptation, Protection	Central intermediary metabolism	
PA1339	aatP	AatP	-4,26	Amino acid biosynthesis and metabolism	Transport of small molecules		
PA1340	aatM	AatM	-5,71	Amino acid biosynthesis and metabolism	Transport of small molecules		
PA1341	aatQ	AatQ	-4,72	Membrane proteins	Transport of small molecules		
PA1342	aatJ	AatJ	-5,26	Amino acid biosynthesis and metabolism	Transport of small molecules		
PA1343		hypothetical protein	4,36	Hypothetical, unclassified, unknown			
PA1344		probable short-chain dehydrogenase	-4,61	Putative enzymes			
PA1347		probable transcriptional regulator	5,06	Transcriptional regulators			
PA1352		conserved hypothetical protein	4,66	Membrane proteins			
PA1353		hypothetical protein	-4,72	Hypothetical, unclassified, unknown			
PA1354		hypothetical protein	-2,15	Hypothetical, unclassified, unknown			
PA1356		hypothetical protein	-3,69	Hypothetical, unclassified, unknown			
PA1357		conserved hypothetical protein	-2,99	Hypothetical, unclassified, unknown			
PA1359		probable transcriptional regulator	5,90	Transcriptional regulators			
PA1360		conserved hypothetical protein	9,79	Membrane proteins			
PA1361	norM	NorM	3,61	Membrane proteins	Transport of small molecules		
PA1362		hypothetical protein	-3,11	Hypothetical, unclassified, unknown			
PA1363		ECF sigma factor	-3,25	Transcriptional regulators			
PA1365		probable siderophore receptor	-2,86	Transport of small molecules			
PA1369		hypothetical protein	-3,10	Hypothetical, unclassified, unknown			
PA1370		hypothetical protein	-2,50	Hypothetical, unclassified, unknown			
PA1371		hypothetical protein	-7,94	Hypothetical, unclassified, unknown			
PA1372		hypothetical protein	-5,03	Hypothetical, unclassified, unknown			
PA1374		hypothetical protein	7,48	Hypothetical, unclassified, unknown			
PA1376	aceK	isocitrate dehydrogenase kinase/phosphatase	-4,57	Central intermediary metabolism			
PA1379		probable short-chain dehydrogenase	3,14	Putative enzymes			
PA1380		probable transcriptional regulator	2,08	Transcriptional regulators			
PA1381		hypothetical protein	3,45	Hypothetical, unclassified, unknown			
PA1382		probable type II secretion system protein	3,88	Protein secretion/export apparatus			
PA1384	galE	UDP-glucose 4-epimerase	5,76	Carbon compound catabolism	Nucleotide biosynthesis and metabolism	Central intermediary metabolism	
PA1386		probable ATP-binding component of ABC transporter	2,74	Transport of small molecules			

PA1393	*cysC*	adenosine 5'-phosphosulfate (APS) kinase	2,24	Amino acid biosynthesis and metabolism	Nucleotide biosynthesis and metabolism	Central intermediary metabolism	
PA1394		hypothetical protein	5,92	Hypothetical, unclassified, unknown			
PA1395		hypothetical protein	8,79	Hypothetical, unclassified, unknown			
PA1397		probable two-component response regulator	2,02	Two-component regulatory systems	Transcriptional regulators		
PA1400		probable pyruvate carboxylase	5,15	Energy metabolism			
PA1401		hypothetical protein	-2,11	Hypothetical, unclassified, unknown			
PA1403		probable transcriptional regulator	2,58	Transcriptional regulators			
PA1405		probable helicase	4,37	DNA replication, recombination, modification and repair			
PA1409	*aphA*	acetylpolyamine aminohydrolase	3,76	Carbon compound catabolism			
PA1410		probable periplasmic spermidine/putrescine-binding protein	2,75	Transport of small molecules			
PA1412		hypothetical protein	4,06	Hypothetical, unclassified, unknown			
PA1414		hypothetical protein	-2,15	Hypothetical, unclassified, unknown			
PA1415		hypothetical protein	-3,18	Hypothetical, unclassified, unknown			
PA1418		probable sodium:solute symport protein	3,80	Transport of small molecules			
PA1419		probable transporter	12,10	Membrane proteins	Transport of small molecules		
PA1420		hypothetical protein	3,27	Hypothetical, unclassified, unknown			
PA1422	*gbuR*	GbuR	3,15	Transcriptional regulators			
PA1423	*bdlA*	BdlA	2,84	Cell Wall / LPS / Capsule	Chemotaxis		x
PA1424		hypothetical protein	6,28	Hypothetical, unclassified, unknown			
PA1425		probable ATP-binding component of ABC transporter	3,95	Transport of small molecules			
PA1426		hypothetical protein	5,32	Hypothetical, unclassified, unknown			
PA1427		hypothetical protein	13,51	Hypothetical, unclassified, unknown			
PA1428		conserved hypothetical protein	3,98	Hypothetical, unclassified, unknown			
PA1430	*lasR*	transcriptional regulator LasR	-4,35	Adaptation, Protection	Transcriptional regulators		x
PA1431	*rsaL*	regulatory protein RsaL	-5,03	Adaptation, Protection	Transcriptional regulators		
PA1435	*mexM*	probable Resistance-Nodulation-Cell Division (RND) efflux membrane fusion protein pr	37,56	Transport of small molecules			
PA1436	*mexN*	probable Resistance-Nodulation-Cell Division (RND) efflux transporter	42,51	Membrane proteins	Transport of small molecules		
PA1437	*mmmR*	probable two-component response regulator	3,28	Two-component regulatory systems	Transcriptional regulators		
PA1438		probable two-component sensor	2,75	Two-component regulatory systems			
PA1439		conserved hypothetical protein	6,31	Hypothetical, unclassified, unknown			
PA1442	*fliL*	conserved hypothetical protein	4,65	Membrane proteins			x
PA1443	*fliM*	flagellar motor switch protein FliM	3,06	Adaptation, Protection	Motility and Attachment	Chemotaxis	x
PA1444	*fliN*	flagellar motor switch protein FliN	2,33	Adaptation, Protection	Motility and Attachment	Chemotaxis	x
PA1445	*fliO*	flagellar protein FliO	2,45	Adaptation, Protection	Motility and Attachment	Chemotaxis	x
PA1446	*fliP*	flagellar biosynthetic protein FliP	3,48	Adaptation, Protection	Motility and Attachment	Chemotaxis	x
PA1448	*fliR*	flagellar biosynthetic protein FliR	3,73	Adaptation, Protection	Motility and Attachment	Chemotaxis	x
PA1449	*flhB*	flagellar biosynthetic protein FlhB	3,64	Adaptation, Protection	Motility and Attachment	Chemotaxis	x
PA1450		conserved hypothetical protein	3,01	Hypothetical, unclassified, unknown			
PA1452	*flhA*	flagellar biosynthesis protein FlhA	17,68	Adaptation, Protection	Motility and Attachment	Chemotaxis	x
PA1453	*flhF*	flagellar biosynthesis protein FlhF	6,43	Adaptation, Protection	Motility and Attachment	Chemotaxis	x
PA1454	*fleN*	flagellar synthesis regulator FleN	3,69	Motility and Attachment			x
PA1455	*fliA*	sigma factor FliA	3,13	Transcriptional regulators			x
PA1456	*cheY*	two-component response regulator CheY	2,34	Two-component regulatory systems	Transcriptional regulators		
PA1458	*cheA*	probable two-component sensor	3,49	Two-component regulatory systems	Chemotaxis		
PA1460	*motC*	MotC	2,15	Motility and Attachment			x
PA1461	*motD*	MotD	2,05	Motility and Attachment			x
PA1462		probable plasmid partitioning protein	3,22	Cell division			
PA1463		hypothetical protein	3,77	Hypothetical, unclassified, unknown			
PA1464	*cheW*	probable purine-binding chemotaxis protein	2,06	Adaptation, Protection	Chemotaxis		
PA1465		hypothetical protein	2,40	Hypothetical, unclassified, unknown			
PA1466		hypothetical protein	2,05	Hypothetical, unclassified, unknown			
PA1467		hypothetical protein	14,05	Hypothetical, unclassified, unknown			
PA1468		hypothetical protein	18,92	Hypothetical, unclassified, unknown			
PA1469		hypothetical protein	5,49	Hypothetical, unclassified, unknown			
PA1470		probable short-chain dehydrogenase	3,62	Putative enzymes			
PA1471		hypothetical protein	2,26	Hypothetical, unclassified, unknown			
PA1472		conserved hypothetical protein	6,33	Hypothetical, unclassified, unknown			
PA1473		hypothetical protein	6,42	Hypothetical, unclassified, unknown			
PA1474		hypothetical protein	9,84	Hypothetical, unclassified, unknown			
PA1475	*ccmA*	heme exporter protein CcmA	4,99	Transport of small molecules			
PA1476	*ccmB*	heme exporter protein CcmB	3,25	Membrane proteins	Transport of small molecules		
PA1480	*ccmF*	cytochrome C-type biogenesis protein CcmF	-3,79	Energy metabolism			
PA1481	*ccmG*	cytochrome C biogenesis protein CcmG	-8,13	Translation, post-translational modification, degradation	Energy metabolism	Chaperones & heat shock proteins	
PA1482	*ccmH*	cytochrome C-type biogenesis protein CcmH	-8,85	Energy metabolism			
PA1483	*cycH*	cytochrome c-type biogenesis protein	-6,10	Energy metabolism			
PA1485		probable amino acid permease	15,70	Membrane proteins	Transport of small molecules		
PA1489		hypothetical protein	2,00	Hypothetical, unclassified, unknown			
PA1491		probable transporter	4,12	Membrane proteins	Transport of small molecules		
PA1493	*cysP*	sulfate-binding protein of ABC transporter	-4,08	Transport of small molecules			
PA1495		hypothetical protein	-3,82	Hypothetical, unclassified, unknown			
PA1497		probable transporter	2,94	Membrane proteins	Transport of small molecules		
PA1498	*pykF*	pyruvate kinase I	4,82	Energy metabolism	Carbon compound catabolism		
PA1502	*gcl*	glyoxylate carboligase	4,25	Central intermediary metabolism			
PA1503		hypothetical protein	3,59	Hypothetical, unclassified, unknown			
PA1507		probable transporter	5,65	Membrane proteins	Transport of small molecules		
PA1512	*hcpA*	secreted protein Hcp	-2,00	Secreted Factors (toxins, enzymes, alginate)			
PA1513		hypothetical protein	-4,26	Membrane proteins			
PA1514		ureidoglycolate hydrolaseYbbT	-4,78	Hypothetical, unclassified, unknown			
PA1515	*alc*	allantoicase	-2,26	Carbon compound catabolism			
PA1516		hypothetical protein	-2,16	Hypothetical, unclassified, unknown			
PA1519		probable transporter	3,13	Membrane proteins	Transport of small molecules		
PA1521		probable guanine deaminase	-2,50	Nucleotide biosynthesis and metabolism			
PA1523	*xdhB*	xanthine dehydrogenase	-2,67	Nucleotide biosynthesis and metabolism			
PA1525	*alkB2*	alkane-1-monooxygenase 2	2,61	Carbon compound catabolism			
PA1530.1	*ffs*	4.5S RNA	21,22	Non-coding RNA gene			
PA1531		hypothetical protein	-3,72	Hypothetical, unclassified, unknown			
PA1532	*dnaX*	DNA polymerase subunits gamma and tau	2,57	DNA replication, recombination, modification and repair			
PA1535		citronellyl-CoA dehydrogenase	6,55	Putative enzymes			
PA1538		probable flavin-containing monooxygenase	4,91	Putative enzymes			
PA1539		hypothetical protein	3,48	Hypothetical, unclassified, unknown			
PA1540		conserved hypothetical protein	81,33	Membrane proteins			
PA1541		probable drug efflux transporter	89,36	Membrane proteins	Transport of small molecules		
PA1542		hypothetical protein	6,52	Hypothetical, unclassified, unknown			
PA1543	*apt*	adenine phosphoribosyltransferase	-2,09	Nucleotide biosynthesis and metabolism			
PA1544	*anr*	transcriptional regulator Anr	-2,94	Transcriptional regulators			
PA1545		hypothetical protein	6,33	Hypothetical, unclassified, unknown			
PA1546	*hemN*	oxygen-independent coproporphyrinogen III oxidase	-17,86	Biosynthesis of cofactors, prosthetic groups and carrier			
PA1547		hypothetical protein	-2,33	Membrane proteins			
PA1548		conserved hypothetical protein	-2,72	Hypothetical, unclassified, unknown			
PA1549	*copA2*	CopA2	-8,33	Membrane proteins	Transport of small molecules		
PA1550		hypothetical protein	-12,05	Hypothetical, unclassified, unknown			
PA1551		probable ferredoxin	-5,85	Energy metabolism			
PA1552	*ccoP1*	Cytochrome c oxidase, cbb3-type, CcoP subunit	-5,21	Energy metabolism	Central intermediary metabolism		
PA1552.1	*ccoQ1*	Cytochrome c oxidase, cbb3-type, CcoQ subunit	-5,85	Central intermediary metabolism			
PA1553	*ccoO1*	Cytochrome c oxidase, cbb3-type, CcoO subunit	-6,02	Energy metabolism	Central intermediary metabolism		
PA1554	*ccoN1*	Cytochrome c oxidase, cbb3-type, CcoN subunit	-5,62	Energy metabolism	Central intermediary metabolism		
PA1555	*ccoP2*	Cytochrome c oxidase, cbb3-type, CcoP subunit	-12,20	Energy metabolism	Central intermediary metabolism		
PA1555.1	*ccoQ2*	Cytochrome c oxidase, cbb3-type, CcoQ subunit	-9,17	Central intermediary metabolism			
PA1556	*ccoO2*	Cytochrome c oxidase, cbb3-type, CcoO subunit	-11,24	Energy metabolism	Central intermediary metabolism		
PA1557	*ccoN2*	Cytochrome c oxidase, cbb3-type, CcoN subunit	-7,87	Energy metabolism	Central intermediary metabolism		
PA1558		hypothetical protein	3,51	Hypothetical, unclassified, unknown			
PA1559		hypothetical protein	4,00	Hypothetical, unclassified, unknown			
PA1560		hypothetical protein	2,66	Hypothetical, unclassified, unknown			
PA1562	*acnA*	aconitate hydratase 1	-3,12	Energy metabolism			
PA1565	*pauB2*	FAD-dependent oxidoreductase	2,90	Putative enzymes	Carbon compound catabolism		
PA1567		conserved hypothetical protein	2,30	Hypothetical, unclassified, unknown			
PA1569		probable major facilitator superfamily (MFS) transporter	3,57	Membrane proteins	Transport of small molecules		
PA1570		probable transcriptional regulator	2,49	Transcriptional regulators			
PA1571		hypothetical protein	-3,98	Hypothetical, unclassified, unknown			
PA1575		hypothetical protein	-3,13	Hypothetical, unclassified, unknown			
PA1576		probable 3-hydroxyisobutyrate dehydrogenase	-2,10	Putative enzymes			
PA1580	*gltA*	citrate synthase	-3,52	Energy metabolism			
PA1581	*sdhC*	succinate dehydrogenase (C subunit)	-2,43	Energy metabolism			
PA1582	*sdhD*	succinate dehydrogenase (D subunit)	-4,52	Energy metabolism			
PA1583	*sdhA*	succinate dehydrogenase (A subunit)	-6,21	Energy metabolism			
PA1584	*sdhB*	succinate dehydrogenase (B subunit)	-6,25	Energy metabolism			
PA1585	*sucA*	2-oxoglutarate dehydrogenase (E1 subunit)	-7,19	Amino acid biosynthesis and metabolism	Energy metabolism		
PA1586	*sucB*	dihydrolipoamide succinyltransferase (E2 subunit)	-5,85	Energy metabolism			
PA1587	*lpd*	dihydrolipoamide dehydrogenase Lpd	-6,94	Amino acid biosynthesis and metabolism	Energy metabolism		x
PA1588	*sucC*	succinyl-CoA synthetase beta chain	-6,02	Energy metabolism			
PA1589	*sucD*	succinyl-CoA synthetase alpha chain	-6,25	Energy metabolism			
PA1590	*braB*	branched chain amino acid transporter	-4,08	Transport of small molecules			
PA1591		hypothetical protein	8,65	Membrane proteins			
PA1592		Lipoprotein	2,24	Hypothetical, unclassified, unknown			
PA1605		hypothetical protein	3,56	Hypothetical, unclassified, unknown			
PA1607		conserved hypothetical protein	2,45	Hypothetical, unclassified, unknown			
PA1608		probable chemotaxis transducer	8,27	Adaptation, Protection	Chemotaxis		
PA1609	*fabB*	beta-ketoacyl-ACP synthase I	-5,41	Fatty acid and phospholipid metabolism			
PA1614	*gpsA*	glycerol-3-phosphate dehydrogenase, biosynthetic	2,75	Fatty acid and phospholipid metabolism	Central intermediary metabolism		
PA1617		probable AMP-binding enzyme	2,87	Putative enzymes			
PA1620		hypothetical protein	3,00	Membrane proteins			
PA1623		conserved hypothetical protein	-4,10	Hypothetical, unclassified, unknown			
PA1624		hypothetical protein	-5,32	Hypothetical, unclassified, unknown			
PA1625		conserved hypothetical protein	5,63	Membrane proteins			
PA1627		probable transcriptional regulator	2,05	Transcriptional regulators			
PA1630		probable transcriptional regulator	2,47	Transcriptional regulators			
PA1631		probable acyl-CoA dehydrogenase	3,27	Putative enzymes			
PA1632	*kdpF*	KdpF protein	40,07	Transport of small molecules			
PA1633	*kdpA*	potassium-transporting ATPase, A chain	5,06	Transport of small molecules			
PA1634	*kdpB*	potassium-transporting ATPase, B chain	3,49	Transport of small molecules			
PA1635	*kdpC*	potassium-transporting ATPase, C chain	2,41	Transport of small molecules			
PA1641		Lipoprotein	3,20	Hypothetical, unclassified, unknown			
PA1646		probable chemotaxis transducer	3,98	Adaptation, Protection	Chemotaxis		
PA1648		probable oxidoreductase	-3,57	Putative enzymes			
PA1649		probable short-chain dehydrogenase	3,54	Putative enzymes			
PA1653		probable transcriptional regulator	3,99	Transcriptional regulators			
PA1655		probable glutathione S-transferase	-2,72	Putative enzymes			
PA1657	*hsiB2*	HsiB2	-2,29	Protein secretion/export apparatus			x
PA1658	*hsiC2*	HsiC2	-3,38	Protein secretion/export apparatus			x
PA1659	*hsiF2*	HsiF2	-3,55	Protein secretion/export apparatus			x
PA1660	*hsiG2*	HsiG2	-4,65	Protein secretion/export apparatus			x
PA1661	*hsiH2*	HsiH2	-3,30	Protein secretion/export apparatus			x
PA1662	*clpV2*	clpV2	-2,99	Protein secretion/export apparatus	Putative enzymes		x
PA1663	*sfa2*	Sfa2	-3,24	Protein secretion/export apparatus	Transcriptional regulators		x
PA1664	*orfX*	OrfX	-3,61	Protein secretion/export apparatus			x
PA1665	*fha2*	Fha2	-5,24	Protein secretion/export apparatus			x
PA1666	*lip2*	Lip2	-5,00	Protein secretion/export apparatus			x
PA1667	*hsiJ2*	HsiJ2	-3,85	Protein secretion/export apparatus			x
PA1668	*dotU2*	DotU2	-2,62	Protein secretion/export apparatus			
PA1672		hypothetical protein	2,21	Hypothetical, unclassified, unknown			
PA1675		conserved hypothetical protein	5,79	Hypothetical, unclassified, unknown			
PA1676		hypothetical protein	4,09	Membrane proteins			
PA1679		hypothetical protein	7,67	Hypothetical, unclassified, unknown			
PA1680		hypothetical protein	9,55	Hypothetical, unclassified, unknown			
PA1683		probable sugar aldolase	-4,52	Putative enzymes			
PA1684		1,2-dihydroxy-3-keto-5-methylthiopentene dioxygenase MtnD	-2,34	Amino acid biosynthesis and metabolism			
PA1685	*masA*	enolase-phosphatase E-1	-4,42	Amino acid biosynthesis and metabolism			
PA1689		conserved hypothetical protein	-3,48	Hypothetical, unclassified, unknown			
PA1690	*pscU*	translocation protein in type III secretion	7,95	Protein secretion/export apparatus			x
PA1691	*pscT*	translocation protein in type III secretion	13,76	Protein secretion/export apparatus			x
PA1692	*pscS*	probable translocation protein in type III secretion	6,42	Protein secretion/export apparatus			x
PA1693	*pscR*	translocation protein in type III secretion	5,37	Protein secretion/export apparatus			x
PA1694	*pscQ*	translocation protein in type III secretion	-2,01	Protein secretion/export apparatus			x
PA1696	*pscO*	translocation protein in type III secretion	2,59	Protein secretion/export apparatus			x
PA1697	*pscN*	ATP synthase in type III secretion system	3,40	Protein secretion/export apparatus			x
PA1698	*popN*	Type III secretion outer membrane protein PopN precursor	2,14	Protein secretion/export apparatus	Membrane proteins		x
PA1699	*pcr1*	Pcr1	2,46	Protein secretion/export apparatus	Secreted Factors (toxins, enzymes, alginate)		x
PA1700	*pcr2*	Pcr2	2,43	Protein secretion/export apparatus	Secreted Factors (toxins, enzymes, alginate)		x
PA1701	*pcr3*	Pcr3	5,04	Protein secretion/export apparatus			x
PA1702	*pcr4*	Pcr4	3,84	Protein secretion/export apparatus	Secreted Factors (toxins, enzymes, alginate)		x
PA1703	*pcrD*	type III secretory apparatus protein PcrD	2,48	Protein secretion/export apparatus			x
PA1705	*pcrG*	regulator in type III secretion	4,22	Protein secretion/export apparatus			x
PA1713	*exsA*	transcriptional regulator ExsA	2,00	Protein secretion/export apparatus	Transcriptional regulators		x
PA1714	*exsD*	ExsD	2,84	Hypothetical, unclassified, unknown			x
PA1715	*pscB*	type III export apparatus protein	9,61	Protein secretion/export apparatus			x
PA1716	*pscC*	Type III secretion outer membrane protein PscC precursor	9,26	Protein secretion/export apparatus			x
PA1717	*pscD*	type III export protein PscD	11,51	Protein secretion/export apparatus			x
PA1718	*pscE*	type III export protein PscE	3,76	Protein secretion/export apparatus	Chaperones & heat shock proteins		x
PA1719	*pscF*	type III export protein PscF	2,06	Protein secretion/export apparatus			x
PA1721	*pscH*	type III export protein PscH	2,22	Protein secretion/export apparatus			x
PA1722	*pscI*	type III export protein PscI	2,33	Protein secretion/export apparatus			x
PA1723	*pscJ*	type III export protein PscJ	2,74	Protein secretion/export apparatus			x
PA1724	*pscK*	type III export protein PscK	2,67	Protein secretion/export apparatus			x
PA1725	*pscL*	type III export protein PscL	2,79	Protein secretion/export apparatus			x
PA1726	*bglX*	periplasmic beta-glucosidase	-2,16	Carbon compound catabolism			
PA1728		hypothetical protein	-2,19	Hypothetical, unclassified, unknown			
PA1732		conserved hypothetical protein	-6,33	Hypothetical, unclassified, unknown			
PA1733		conserved hypothetical protein	-9,90	Hypothetical, unclassified, unknown			
PA1734		hypothetical protein	-2,32	Hypothetical, unclassified, unknown			
PA1735		hypothetical protein	3,52	Membrane proteins			
PA1739		probable oxidoreductase	6,68	Putative enzymes			
PA1740		hypothetical protein	3,67	Hypothetical, unclassified, unknown			
PA1743		hypothetical protein	4,28	Hypothetical, unclassified, unknown			
PA1745		Lipoprotein	-3,10	Hypothetical, unclassified, unknown			
PA1746		hypothetical protein	-18,52	Hypothetical, unclassified, unknown			
PA1748		probable enoyl-CoA hydratase/isomerase	-2,15	Putative enzymes			
PA1760		probable transcriptional regulator	-2,29	Transcriptional regulators			
PA1762		hypothetical protein	-2,16	Hypothetical, unclassified, unknown			
PA1764		hypothetical protein	4,78	Hypothetical, unclassified, unknown			
PA1766		hypothetical protein	-3,11	Hypothetical, unclassified, unknown			
PA1767		hypothetical protein	-2,23	Membrane proteins			
PA1769		conserved hypothetical protein	-3,17	Hypothetical, unclassified, unknown			
PA1771	*estX*	EstX	2,70	Putative enzymes			
PA1772		probable methyltransferase	-3,53	Biosynthesis of cofactors, prosthetic groups and carrier			
PA1773	*cmaX*	CmaX protein	5,94	Membrane proteins			
PA1774	*crfX*	CrfX protein	8,87	Hypothetical, unclassified, unknown			
PA1775	*cmpX*	conserved cytoplasmic membrane protein, CmpX protein	9,91	Membrane proteins			
PA1776	*sigX*	ECF sigma factor SigX	2,33	Transcriptional regulators			
PA1779		assimilatory nitrate reductase	2,65	Central intermediary metabolism			
PA1781	*nirB*	assimilatory nitrite reductase large subunit	2,19	Central intermediary metabolism			
PA1781.1	*nalA*	nitrogen assimilation leader A (NalA)	9,79	Non-coding RNA gene			
PA1782		probable serine/threonine-protein kinase	4,78	Adaptation, Protection			
PA1783	*nasA*	nitrate transporter	3,24	Membrane proteins	Transport of small molecules		
PA1784		hypothetical protein	-29,41	Hypothetical, unclassified, unknown			
PA1786	*nasS*	NasS	2,03	Hypothetical, unclassified, unknown			
PA1787	*acnB*	aconitate hydratase 2	-2,77	Energy metabolism			
PA1789	*uspL*	Universal stress protein	-8,00	Hypothetical, unclassified, unknown			
PA1790		hypothetical protein	3,99	Hypothetical, unclassified, unknown			
PA1791		hypothetical protein	2,30	Hypothetical, unclassified, unknown			
PA1793	*ppiB*	peptidyl-prolyl cis-trans isomerase B	-3,94	Translation, post-translational modification, degradation	Chaperones & heat shock proteins		
PA1794	*glnS*	glutaminyl-tRNA synthetase	-2,51	Translation, post-translational modification, degradation	Amino acid biosynthesis and metabolism		
PA1795	*cysS*	cysteinyl-tRNA synthetase	-2,75	Translation, post-translational modification, degradation	Amino acid biosynthesis and metabolism		
PA1796.1		tRNA-Arg	3,90	Non-coding RNA gene			
PA1796.2		tRNA-His	2,46	Non-coding RNA gene			
PA1796.3		tRNA-Leu	12,00	Non-coding RNA gene			
PA1796.4		tRNA-His	29,67	Non-coding RNA gene			
PA1798	*parS*	two-component sensor, ParS	-3,01	Two-component regulatory systems	Antibiotic resistance and susceptibility		
PA1801	*clpP*	ClpP	-2,98	Cell Wall / LPS / Capsule	Antibiotic resistance and susceptibility	Motility and Attachment	
PA1802	*clpX*	ClpX	-4,20	Cell Wall / LPS / Capsule	Chaperones & heat shock proteins		
PA1803	*lon*	Lon protease	-3,53	Translation, post-translational modification, degradation	Adaptation, Protection		x
PA1804.1		tRNA-Asp	-2,50	Non-coding RNA gene			
PA1806	*fabI*	NADH-dependent enoyl-ACP reductase	-7,81	Fatty acid and phospholipid metabolism			
PA1807	*nppD*	NppD	-10,42	Transport of small molecules			
PA1808	*nppC*	NppC	-12,05	Transport of small molecules			
PA1809	*nppB*	NppB	-4,44	Transport of small molecules			
PA1810	*nppA2*	NppA2	-4,13	Transport of small molecules			
PA1812	*mltD*	membrane-bound lytic murein transglycosylase D precursor	-6,76	Amino acid biosynthesis and metabolism	Cell Wall / LPS / Capsule		
PA1813		probable hydroxyacylglutathione hydrolase	-4,55	Adaptation, Protection	Putative enzymes		
PA1817		hypothetical protein	-2,18	Hypothetical, unclassified, unknown			
PA1818	*cadA*	lysine decarboxylase	-3,72	Amino acid biosynthesis and metabolism			
PA1821		probable enoyl-CoA hydratase/isomerase	-2,18	Putative enzymes			
PA1822	*fimL*	hypothetical protein	-2,33	Motility and Attachment			
PA1823	*nudC*	NudC	-2,92	Hypothetical, unclassified, unknown			
PA1824		conserved hypothetical protein	3,99	Membrane proteins			
PA1826		probable transcriptional regulator	-2,86	Transcriptional regulators			
PA1827		probable short-chain dehydrogenase	9,22	Putative enzymes			
PA1828		probable short-chain dehydrogenase	-5,81	Putative enzymes			
PA1829		hypothetical protein	-4,90	Putative enzymes			
PA1830		hypothetical protein	-4,98	Hypothetical, unclassified, unknown			
PA1833		probable oxidoreductase	-2,68	Putative enzymes			
PA1835		hypothetical protein	-2,09	Hypothetical, unclassified, unknown			
PA1836		probable transcriptional regulator	2,57	Transcriptional regulators			
PA1837		hypothetical protein	-5,92	Hypothetical, unclassified, unknown			
PA1838	*cysI*	sulfite reductase	-2,37	Central intermediary metabolism			
PA1839		hypothetical protein	4,69	Hypothetical, unclassified, unknown			
PA1842		hypothetical protein	-9,71	Hypothetical, unclassified, unknown			
PA1843	*metH*	methionine synthase	-3,45	Amino acid biosynthesis and metabolism			
PA1844	*tse1*	Tse1	2,06	Secreted Factors (toxins, enzymes, alginate)			
PA1848		probable major facilitator superfamily (MFS) transporter	3,77	Membrane proteins	Transport of small molecules		
PA1849		conserved hypothetical protein	4,44	Hypothetical, unclassified, unknown			
PA1850		probable transcriptional regulator	4,45	Transcriptional regulators			
PA1851		hypothetical protein	3,70	Membrane proteins			
PA1852		hypothetical protein	2,12	Hypothetical, unclassified, unknown			
PA1853		probable transcriptional regulator	7,70	Transcriptional regulators			
PA1854		conserved hypothetical protein	4,55	Hypothetical, unclassified, unknown			
PA1855		hypothetical protein	4,45	Hypothetical, unclassified, unknown			
PA1858	*str*	streptomycin 3''-phosphotransferase	-4,39	Antibiotic resistance and susceptibility			
PA1864		probable transcriptional regulator	2,29	Transcriptional regulators			
PA1865		hypothetical protein	2,48	Hypothetical, unclassified, unknown			
PA1866		hypothetical protein	4,01	Hypothetical, unclassified, unknown			
PA1867	*xphA*	XphA	6,62	Protein secretion/export apparatus			

PA1868	*xqhA*	secretion protein XqhA	6,92	Protein secretion/export apparatus			
PA1869	*acp1*	Acp1	-6,45	Fatty acid and phospholipid metabolism			
PA1870		hypothetical protein	-2,02	Hypothetical, unclassified, unknown			
PA1871	*lasA*	LasA protease precursor	-62,50	Translation, post-translational modification, degradation	Secreted Factors (toxins, enzymes, alginate)		x
PA1872		hypothetical protein	-2,56	Hypothetical, unclassified, unknown			
PA1873		hypothetical protein	2,68	Membrane proteins			
PA1874		hypothetical protein	-33,33	Antibiotic resistance and susceptibility			
PA1875		probable outer membrane protein precursor	-15,38	Antibiotic resistance and susceptibility	Protein secretion/export apparatus		
PA1876		probable ATP-binding/permease fusion ABC transporter	-10,87	Antibiotic resistance and susceptibility	Protein secretion/export apparatus	Secreted Factors (toxins, enzymes, alginate)	
PA1877		probable secretion protein	-12,82	Antibiotic resistance and susceptibility	Protein secretion/export apparatus		
PA1880		probable oxidoreductase	-10,87	Putative enzymes			
PA1881		probable oxidoreductase	-6,25	Putative enzymes			
PA1882		probable transporter	9,83	Membrane proteins	Transport of small molecules		
PA1883		probable NADH-ubiquinone/plastoquinone oxidoreductase	32,13	Energy metabolism			
PA1886	*polB*	DNA polymerase II	2,04	DNA replication, recombination, modification and repair			
PA1887		hypothetical protein	-24,39	Hypothetical, unclassified, unknown			
PA1888		Lipoprotein	-12,82	Hypothetical, unclassified, unknown			
PA1889		hypothetical protein	9,07	Hypothetical, unclassified, unknown			
PA1890		probable glutathione S-transferase	-5,24	Putative enzymes			
PA1891		hypothetical protein	-6,80	Membrane proteins			
PA1892		hypothetical protein	-11,24	Hypothetical, unclassified, unknown			
PA1893		hypothetical protein	-12,20	Putative enzymes			
PA1894		hypothetical protein	-16,95	Hypothetical, unclassified, unknown			
PA1895		hypothetical protein	-16,13	Membrane proteins			
PA1896		hypothetical protein	-11,63	Hypothetical, unclassified, unknown			
PA1897		hypothetical protein	-10,53	Membrane proteins			
PA1898	*qscR*	quorum-sensing control repressor	13,46	Transcriptional regulators			
PA1899	*phzA2*	probable phenazine biosynthesis protein	-16,39	Secreted Factors (toxins, enzymes, alginate)			x
PA1900	*phzB2*	probable phenazine biosynthesis protein	-37,04	Secreted Factors (toxins, enzymes, alginate)			x
PA1901	*phzC2*	phenazine biosynthesis protein PhzC	-16,39	Secreted Factors (toxins, enzymes, alginate)			x
PA1902	*phzD2*	phenazine biosynthesis protein PhzD	-17,24	Secreted Factors (toxins, enzymes, alginate)			x
PA1903	*phzE2*	phenazine biosynthesis protein PhzE	-19,23	Secreted Factors (toxins, enzymes, alginate)			x
PA1904	*phzF2*	probable phenazine biosynthesis protein	-21,28	Secreted Factors (toxins, enzymes, alginate)			x
PA1905	*phzG2*	probable pyridoxamine 5'-phosphate oxidase	-23,81	Secreted Factors (toxins, enzymes, alginate)			x
PA1907		hypothetical protein	3,06	Hypothetical, unclassified, unknown			
PA1909		hypothetical protein	-3,72	Hypothetical, unclassified, unknown			
PA1914		conserved hypothetical protein	-333,33	Putative enzymes			
PA1915		hypothetical protein	-2,22	Hypothetical, unclassified, unknown			
PA1916		probable amino acid permease	3,25	Transport of small molecules			
PA1918		hypothetical protein	3,18	Putative enzymes			
PA1920	*nrdD*	class III (anaerobic) ribonucleoside-triphosphate reductase subunit, NrdD	3,86	Nucleotide biosynthesis and metabolism			
PA1921		hypothetical protein	2,98	Hypothetical, unclassified, unknown			
PA1922		probable TonB-dependent receptor	2,66	Transport of small molecules			
PA1923		hypothetical protein	4,08	Biosynthesis of cofactors, prosthetic groups and carrier			
PA1928	*rimJ*	ribosomal protein alanine acetyltransferase	2,00	Translation, post-translational modification, degradation			
PA1929		hypothetical protein	3,27	Hypothetical, unclassified, unknown			
PA1930		probable chemotaxis transducer	-14,49	Adaptation, Protection	Chemotaxis		
PA1931		probable ferredoxin	-2,95	Energy metabolism	Carbon compound catabolism		
PA1935		hypothetical protein	2,23	Hypothetical, unclassified, unknown			
PA1936		hypothetical protein	2,71	Hypothetical, unclassified, unknown			
PA1937		conserved hypothetical protein	3,71	Relative to phage, transposon, or plasmid			
PA1938		conserved hypothetical protein	4,33	Relative to phage, transposon, or plasmid			
PA1939		hypothetical protein	-8,00	Hypothetical, unclassified, unknown			
PA1940		hypothetical protein	-7,75	Hypothetical, unclassified, unknown			
PA1942		hypothetical protein	18,51	Hypothetical, unclassified, unknown			
PA1943		hypothetical protein	-2,53	Hypothetical, unclassified, unknown			
PA1945		probable transcriptional regulator	2,62	Transcriptional regulators			
PA1946	*rbsB*	binding protein component precursor of ABC ribose transporter	6,61	Transport of small molecules			
PA1947	*rbsA*	ribose transport protein RbsA	10,85	Transport of small molecules			
PA1948	*rbsC*	membrane protein component of ABC ribose transporter	7,49	Transport of small molecules			
PA1949	*rbsR*	ribose operon repressor RbsR	2,75	Transcriptional regulators	Carbon compound catabolism		
PA1951	*fapF*	FapF	-16,13	Motility and Attachment			
PA1952	*fapE*	FapE	3,94	Motility and Attachment			
PA1953	*fapD*	FapD	3,34	Motility and Attachment			
PA1955	*fapB*	FapB	13,57	Motility and Attachment			
PA1956	*fapA*	FapA	21,97	Motility and Attachment			
PA1959	*bacA*	bacitracin resistance protein	22,98	Cell Wall / LPS / Capsule	Antibiotic resistance and susceptibility	Adaptation, Protection	
PA1960		hypothetical protein	17,56	Membrane proteins			
PA1962	*azoR2*	FMN-dependent NADH-azoreductase 2, AzoR2	20,49	Putative enzymes			
PA1964		probable ATP-binding component of ABC transporter	2,63	Transport of small molecules			
PA1965		Lipoprotein	2,43	Hypothetical, unclassified, unknown			
PA1966		hypothetical protein	4,69	Putative enzymes			
PA1967		hypothetical protein	-6,90	Hypothetical, unclassified, unknown			
PA1970		Lipoprotein	6,56	Hypothetical, unclassified, unknown			
PA1971	*braZ*	branched chain amino acid transporter BraZ	9,61	Transport of small molecules			
PA1972		conserved hypothetical protein	19,89	Membrane proteins			
PA1974		hypothetical protein	7,94	Hypothetical, unclassified, unknown			
PA1975		hypothetical protein	4,82	Hypothetical, unclassified, unknown			
PA1977		hypothetical protein	8,32	Membrane proteins			
PA1978	*erbR*	response regulator ErbR	4,36	Transcriptional regulators			
PA1979	*eraS*	sensor kinase, EraS	2,06	Two-component regulatory systems			
PA1980	*eraR*	response regulator EraR	2,82	Two-component regulatory systems	Transcriptional regulators		
PA1981		hypothetical protein	2,57	Hypothetical, unclassified, unknown			
PA1982	*exaA*	quinoprotein ethanol dehydrogenase	4,95	Carbon compound catabolism			
PA1983	*exaB*	cytochrome c550	5,28	Energy metabolism	Carbon compound catabolism		
PA1984	*exaC*	NAD+ dependent aldehyde dehydrogenase ExaC	-2,20	Putative enzymes			
PA1985	*pqqA*	pyrroloquinoline quinone biosynthesis protein A	-16,39	Biosynthesis of cofactors, prosthetic groups and carrier			
PA1986	*pqqB*	pyrroloquinoline quinone biosynthesis protein B	-6,25	Biosynthesis of cofactors, prosthetic groups and carrier			
PA1987	*pqqC*	pyrroloquinoline quinone biosynthesis protein C	-5,49	Biosynthesis of cofactors, prosthetic groups and carrier			
PA1988	*pqqD*	pyrroloquinoline quinone biosynthesis protein D	-3,51	Biosynthesis of cofactors, prosthetic groups and carrier			
PA1989	*pqqE*	pyrroloquinoline quinone biosynthesis protein E	-6,33	Biosynthesis of cofactors, prosthetic groups and carrier			
PA1990	*pqqH*	PqqH	-8,33	Putative enzymes			
PA1993		probable major facilitator superfamily (MFS) transporter	3,98	Membrane proteins	Transport of small molecules		
PA1994		hypothetical protein	-2,76	Hypothetical, unclassified, unknown			
PA1995		hypothetical protein	-2,36	Hypothetical, unclassified, unknown			
PA1999	*dhcA*	dehydrocarnitine CoA transferase, subunit A	-6,29	Amino acid biosynthesis and metabolism	Carbon compound catabolism		
PA2000	*dhcB*	dehydrocarnitine CoA transferase, subunit B	-5,62	Amino acid biosynthesis and metabolism	Carbon compound catabolism		
PA2001	*atoB*	acetyl-CoA acetyltransferase	-5,46	Fatty acid and phospholipid metabolism	Central intermediary metabolism		
PA2002		conserved hypothetical protein	-2,13	Membrane proteins			
PA2003	*bdhA*	3-hydroxybutyrate dehydrogenase	-10,31	Carbon compound catabolism			
PA2004		conserved hypothetical protein	-2,86	Membrane proteins			
PA2006		probable major facilitator superfamily (MFS) transporter	-5,46	Membrane proteins	Transport of small molecules		
PA2007	*maiA*	maleylacetoacetate isomerase	-8,33	Carbon compound catabolism			
PA2008	*fahA*	fumarylacetoacetase	-5,65	Carbon compound catabolism			
PA2009	*hmgA*	homogentisate 1,2-dioxygenase	-3,51	Carbon compound catabolism			
PA2011	*liuE*	3-hydroxy-3-methylglutaryl-CoA lyase	-6,13	Carbon compound catabolism			
PA2012	*liuD*	methylcrotonyl-CoA carboxylase, alpha-subunit (biotin-containing)	-7,04	Carbon compound catabolism			
PA2013	*liuC*	putative 3-methylglutaconyl-CoA hydratase	-5,71	Carbon compound catabolism			
PA2014	*liuB*	methylcrotonyl-CoA carboxylase, beta-subunit	-4,12	Carbon compound catabolism			
PA2015	*liuA*	putative isovaleryl-CoA dehydrogenase	-3,12	Carbon compound catabolism			
PA2016	*liuR*	regulator of liu genes	-2,92	Transcriptional regulators			
PA2017		hypothetical protein	4,59	Hypothetical, unclassified, unknown			
PA2018	*mexY*	Resistance-Nodulation-Cell Division (RND) multidrug efflux transporter MexY	8,33	Membrane proteins	Antibiotic resistance and susceptibility	Transport of small molecules	
PA2019	*mexX*	Resistance-Nodulation-Cell Division (RND) multidrug efflux membrane fusion protein M	13,54	Antibiotic resistance and susceptibility	Transport of small molecules		
PA2020	*mexZ*	MexZ	2,51	Transcriptional regulators			
PA2021		hypothetical protein	2,25	Hypothetical, unclassified, unknown			
PA2022		probable nucleotide sugar dehydrogenase	18,44	Putative enzymes			
PA2024		probable ring-cleaving dioxygenase	-2,60	Putative enzymes			
PA2025	*gor*	glutathione reductase	-3,42	Amino acid biosynthesis and metabolism	Biosynthesis of cofactors, prosthetic groups and carrier		
PA2029		hypothetical protein	-2,72	Hypothetical, unclassified, unknown			
PA2030		hypothetical protein	-10,64	Hypothetical, unclassified, unknown			
PA2031		hypothetical protein	-8,47	Hypothetical, unclassified, unknown			
PA2033		hypothetical protein	-2,23	Hypothetical, unclassified, unknown			
PA2034		hypothetical protein	-2,28	Hypothetical, unclassified, unknown			
PA2036		hypothetical protein	2,66	Hypothetical, unclassified, unknown			
PA2037		hypothetical protein	2,36	Hypothetical, unclassified, unknown			
PA2038		hypothetical protein	-5,59	Hypothetical, unclassified, unknown			
PA2041		Amino acid permease	2,70	Transport of small molecules	Carbon compound catabolism		
PA2042		probable transporter (membrane subunit)	-2,65	Transport of small molecules			
PA2044		hypothetical protein	-5,03	Hypothetical, unclassified, unknown			
PA2046		hypothetical protein	3,61	Hypothetical, unclassified, unknown			
PA2047	*cmrA*	chloramphenicol resistance activator CmrA	-5,18	Transcriptional regulators			
PA2049		hypothetical protein	-2,79	Hypothetical, unclassified, unknown			
PA2050		probable sigma-70 factor, ECF subfamily	9,11	Transcriptional regulators			
PA2051		probable transmembrane sensor	6,82	Membrane proteins	Transcriptional regulators		
PA2052	*cynS*	cyanate lyase	-14,29	Central intermediary metabolism			
PA2053	*cynT*	carbonate dehydratase	-2,04	Adaptation, Protection	Central intermediary metabolism		
PA2055		probable major facilitator superfamily (MFS) transporter	3,51	Membrane proteins	Antibiotic resistance and susceptibility	Transport of small molecules	
PA2056		probable transcriptional regulator	2,17	Transcriptional regulators			
PA2057	*sppR*	TonB-dependent receptor, SppR	7,31	Hypothetical, unclassified, unknown			
PA2058	*sppA*	ABC transporter substrate-binding protein, SppA	13,08	Transport of small molecules			
PA2059	*sppB*	ABC transporter permease, SppB	23,43	Membrane proteins	Transport of small molecules		
PA2060	*sppC*	ABC transporter permease, SppC	8,45	Transport of small molecules			
PA2061	*sppD*	ABC transporter ATP-binding protein, SppD	3,11	Transport of small molecules			
PA2064	*pcoB*	copper resistance protein B precursor	-2,16	Adaptation, Protection			
PA2065	*pcoA*	copper resistance protein A precursor	-3,46	Adaptation, Protection			
PA2066		hypothetical protein	-21,28	Hypothetical, unclassified, unknown			
PA2067		probable hydrolase	-25,64	Putative enzymes			
PA2068		probable major facilitator superfamily (MFS) transporter	-33,33	Membrane proteins	Transport of small molecules		
PA2069		probable carbamoyl transferase	-38,46	Putative enzymes			
PA2070		hypothetical protein	8,58	Membrane proteins			
PA2071	*fusA2*	elongation factor G	-2,51	Translation, post-translational modification, degradation			
PA2072		conserved hypothetical protein	-2,28	Membrane proteins			
PA2073		probable transporter (membrane subunit)	3,56	Transport of small molecules			
PA2079		probable amino acid permease	-3,39	Transport of small molecules			
PA2080	*kynU*	kynureninase KynU	-5,43	Amino acid biosynthesis and metabolism			
PA2085		probable ring-hydroxylating dioxygenase small subunit	5,01	Carbon compound catabolism			
PA2086		probable epoxide hydrolase	2,49	Carbon compound catabolism			
PA2087		hypothetical protein	2,44	Hypothetical, unclassified, unknown			
PA2089		hypothetical protein	3,55	Hypothetical, unclassified, unknown			
PA2092		probable major facilitator superfamily (MFS) transporter	2,30	Membrane proteins	Transport of small molecules		
PA2094		probable transmembrane sensor	4,61	Membrane proteins	Transcriptional regulators		
PA2104		probable cysteine synthase	3,71	Amino acid biosynthesis and metabolism			
PA2105		probable acetyltransferase	6,14	Putative enzymes			
PA2106		hypothetical protein	6,20	Hypothetical, unclassified, unknown			
PA2107		hypothetical protein	2,95	Hypothetical, unclassified, unknown			
PA2108		probable decarboxylase	11,04	Putative enzymes			
PA2109		hypothetical protein	-18,87	Hypothetical, unclassified, unknown			
PA2110		hypothetical protein	-18,87	Hypothetical, unclassified, unknown			
PA2111		hypothetical protein	-16,39	Hypothetical, unclassified, unknown			
PA2112		conserved hypothetical protein	-23,26	Hypothetical, unclassified, unknown			
PA2113	*opdO*	pyroglutamate porin OpdO	-19,61	Membrane proteins	Transport of small molecules		
PA2114		probable major facilitator superfamily (MFS) transporter	-8,00	Membrane proteins	Transport of small molecules		
PA2116		conserved hypothetical protein	-29,41	Hypothetical, unclassified, unknown			
PA2118	*ada*	O6-methylguanine-DNA methyltransferase	-2,36	DNA replication, recombination, modification and repair	Transcriptional regulators		
PA2119		alcohol dehydrogenase (Zn-dependent)	-7,30	Putative enzymes			
PA2122		hypothetical protein	-3,25	Hypothetical, unclassified, unknown			
PA2123		probable transcriptional regulator	-3,11	Transcriptional regulators			
PA2124		probable dehydrogenase	2,54	Putative enzymes			
PA2127	*cgrA*	cupA gene regulator A, CgrA	2,94	Transcriptional regulators			
PA2129	*cupA2*	chaperone CupA2	3,15	Motility and Attachment	Chaperones & heat shock proteins		
PA2130	*cupA3*	usher CupA3	4,37	Motility and Attachment			
PA2131	*cupA4*	fimbrial subunit CupA4	3,57	Motility and Attachment			
PA2132	*cupA5*	chaperone CupA5	3,56	Motility and Attachment	Chaperones & heat shock proteins		
PA2133		Cyclic-guanylate-specific phosphodiesterase	2,32	Hypothetical, unclassified, unknown			
PA2135		probable transporter	2,75	Membrane proteins	Transport of small molecules		
PA2136		hypothetical protein	8,34	Hypothetical, unclassified, unknown			
PA2137		hypothetical protein	6,64	Hypothetical, unclassified, unknown			
PA2138	*ligD*	Multifunctional non-homologous end joining protein LigD	3,93	DNA replication, recombination, modification and repair			
PA2139		hypothetical protein	8,31	Hypothetical, unclassified, unknown			
PA2141		hypothetical protein	2,36	Hypothetical, unclassified, unknown			
PA2144	*glgP*	glycogen phosphorylase	-2,14	Cell Wall / LPS / Capsule			
PA2145		hypothetical protein	5,71	Hypothetical, unclassified, unknown			
PA2148		conserved hypothetical protein	2,48	Membrane proteins			
PA2150		conserved hypothetical protein	2,12	Hypothetical, unclassified, unknown			
PA2156		conserved hypothetical protein	2,05	Hypothetical, unclassified, unknown			
PA2160		probable glycosyl hydrolase	-2,65	Putative enzymes			
PA2161		hypothetical protein	-5,99	Hypothetical, unclassified, unknown			
PA2162		probable glycosyl hydrolase	-2,58	Putative enzymes			
PA2163		hypothetical protein	-3,97	Hypothetical, unclassified, unknown			
PA2164		probable glycosyl hydrolase	-2,60	Putative enzymes			
PA2166		hypothetical protein	-2,68	Hypothetical, unclassified, unknown			
PA2171		hypothetical protein	-2,26	Hypothetical, unclassified, unknown			
PA2173		hypothetical protein	-2,50	Hypothetical, unclassified, unknown			
PA2174		hypothetical protein	-3,98	Hypothetical, unclassified, unknown			
PA2181		hypothetical protein	14,88	Hypothetical, unclassified, unknown			
PA2185	*katN*	non-heme catalase KatN	3,24	Adaptation, Protection			
PA2186		hypothetical protein	5,43	Hypothetical, unclassified, unknown			
PA2187		hypothetical protein	2,77	Hypothetical, unclassified, unknown			
PA2188		probable alcohol dehydrogenase (Zn-dependent)	3,78	Putative enzymes			
PA2189		hypothetical protein	4,81	Hypothetical, unclassified, unknown			
PA2191	*exoY*	adenylate cyclase ExoY	3,53	Secreted Factors (toxins, enzymes, alginate)			x
PA2193	*hcnA*	hydrogen cyanide synthase HcnA	-8,33	Central intermediary metabolism			x
PA2194	*hcnB*	hydrogen cyanide synthase HcnB	-9,35	Central intermediary metabolism			x
PA2195	*hcnC*	hydrogen cyanide synthase HcnC	-12,20	Central intermediary metabolism			x
PA2197		conserved hypothetical protein	-2,91	Putative enzymes			
PA2198		hypothetical protein	-2,26	Hypothetical, unclassified, unknown			
PA2199		probable dehydrogenase	-2,91	Putative enzymes			
PA2208		hypothetical protein	3,12	Hypothetical, unclassified, unknown			
PA2209		hypothetical protein	5,80	Hypothetical, unclassified, unknown			
PA2210		probable major facilitator superfamily (MFS) transporter	14,37	Membrane proteins	Transport of small molecules		
PA2211		conserved hypothetical protein	7,21	Hypothetical, unclassified, unknown			
PA2213	*opdG*	probable porin	7,31	Membrane proteins	Transport of small molecules		
PA2214		putative L-lyxonate transporter	8,14	Membrane proteins	Transport of small molecules		
PA2215	*lyxD*	l-lyxonate dehydratase LyxD	2,35	Hypothetical, unclassified, unknown			
PA2216		2-keto-3-deoxy-D-arabinonate dehydratase	2,94	Hypothetical, unclassified, unknown			
PA2219	*opdE*	membrane protein OpdE	2,64	Membrane proteins	Transport of small molecules		
PA2222		hypothetical protein	-9,90	Hypothetical, unclassified, unknown			
PA2223		hypothetical protein	-11,90	Hypothetical, unclassified, unknown			
PA2224		hypothetical protein	-2,40	Hypothetical, unclassified, unknown			
PA2225		Lipoprotein	-3,36	Hypothetical, unclassified, unknown			
PA2226	*qsrO*	QsrO	-9,17	Hypothetical, unclassified, unknown			
PA2227	*vqsM*	AraC-type transcriptional regulator VqsM	-3,76	Transcriptional regulators			
PA2229		conserved hypothetical protein	-2,58	Hypothetical, unclassified, unknown			
PA2232	*pslB*	PslB	-10,99	Cell Wall / LPS / Capsule			
PA2233	*pslC*	PslC	-13,70	Cell Wall / LPS / Capsule	Putative enzymes		
PA2234	*pslD*	PslD	-18,52	Cell Wall / LPS / Capsule	Transport of small molecules		
PA2235	*pslE*	PslE	-18,18	Cell Wall / LPS / Capsule			
PA2236	*pslF*	PslF	-21,74	Cell Wall / LPS / Capsule			
PA2237	*pslG*	PslG	-12,82	Cell Wall / LPS / Capsule	Putative enzymes		
PA2238	*pslH*	PslH	-15,38	Cell Wall / LPS / Capsule			
PA2239	*pslI*	PslI	-10,64	Cell Wall / LPS / Capsule	Putative enzymes		
PA2240	*pslJ*	PslJ	-7,41	Cell Wall / LPS / Capsule			
PA2241	*pslK*	PslL	-5,52	Cell Wall / LPS / Capsule	Membrane proteins		
PA2242	*pslL*	hypothetical protein	-15,87	Hypothetical, unclassified, unknown			
PA2243	*pslM*	hypothetical protein	-2,28	Hypothetical, unclassified, unknown			
PA2244	*pslN*	hypothetical protein	2,32	Hypothetical, unclassified, unknown			
PA2245	*pslO*	hypothetical protein	2,50	Hypothetical, unclassified, unknown			
PA2247	*bkdA1*	2-oxoisovalerate dehydrogenase (alpha subunit)	-3,68	Amino acid biosynthesis and metabolism			
PA2248	*bkdA2*	2-oxoisovalerate dehydrogenase (beta subunit)	-5,24	Amino acid biosynthesis and metabolism			
PA2249	*bkdB*	branched-chain alpha-keto acid dehydrogenase (lipoamide component)	-4,63	Amino acid biosynthesis and metabolism			
PA2250	*lpdV*	lipoamide dehydrogenase-Val	-4,69	Amino acid biosynthesis and metabolism	Energy metabolism		
PA2251		hypothetical protein	3,63	Hypothetical, unclassified, unknown			
PA2253	*ansA*	L-asparaginase I	-2,93	Amino acid biosynthesis and metabolism			
PA2254	*pvcA*	paerucumarin biosynthesis protein PvcA	-4,83	Amino acid biosynthesis and metabolism	Secreted Factors (toxins, enzymes, alginate)		
PA2255	*pvcB*	paerucumarin biosynthesis protein PvcB	-3,86	Amino acid biosynthesis and metabolism	Secreted Factors (toxins, enzymes, alginate)		
PA2256	*pvcC*	paerucumarin biosynthesis protein PvcC	-2,62	Amino acid biosynthesis and metabolism	Secreted Factors (toxins, enzymes, alginate)		
PA2258	*ptxR*	transcriptional regulator PtxR	11,78	Transcriptional regulators			
PA2262		probable 2-ketogluconate transporter	2,73	Membrane proteins	Transport of small molecules		
PA2263	*kguD*	probable 2-hydroxyacid dehydrogenase	-2,52	Putative enzymes			
PA2265	*gad*	gluconate dehydrogenase	-3,26	Carbon compound catabolism			
PA2266		probable cytochrome c precursor	-5,32	Energy metabolism	Carbon compound catabolism		
PA2269		conserved hypothetical protein	2,49	Membrane proteins			
PA2275		probable alcohol dehydrogenase (Zn-dependent)	8,43	Putative enzymes			
PA2276		probable transcriptional regulator	2,63	Transcriptional regulators			
PA2277	*arsR*	ArsR protein	5,25	Transcriptional regulators			
PA2278	*arsB*	ArsB protein	5,64	Membrane proteins	Transport of small molecules		
PA2280		oxidoreductase	-5,95	Hypothetical, unclassified, unknown			
PA2281		probable transcriptional regulator	4,17	Transcriptional regulators			
PA2282		hypothetical protein	2,09	Hypothetical, unclassified, unknown			
PA2283		hypothetical protein	5,65	Hypothetical, unclassified, unknown			
PA2284		hypothetical protein	3,70	Hypothetical, unclassified, unknown			
PA2285		hypothetical protein	-2,13	Hypothetical, unclassified, unknown			
PA2287		hypothetical protein	2,87	Hypothetical, unclassified, unknown			
PA2288		hypothetical protein	24,81	Hypothetical, unclassified, unknown			
PA2289		conserved hypothetical protein	-6,41	Hypothetical, unclassified, unknown			
PA2290	*gcd*	glucose dehydrogenase	-5,43	Energy metabolism	Carbon compound catabolism		
PA2294		probable ATP-binding component of ABC transporter	2,05	Transport of small molecules			
PA2295		probable permease of ABC transporter	2,44	Membrane proteins	Transport of small molecules		
PA2300	*chiC*	chitinase	-18,87	Carbon compound catabolism			
PA2301		hypothetical protein	-10,42	Hypothetical, unclassified, unknown			
PA2302	*ambE*	AmbE	-14,49	Secreted Factors (toxins, enzymes, alginate)	Putative enzymes		
PA2303	*ambD*	AmbD	-16,13	Secreted Factors (toxins, enzymes, alginate)			
PA2304	*ambC*	AmbC	-11,24	Secreted Factors (toxins, enzymes, alginate)			
PA2305	*ambB*	AmbB	-3,89	Secreted Factors (toxins, enzymes, alginate)	Putative enzymes		
PA2306	*ambA*	AmbA	-6,41	Secreted Factors (toxins, enzymes, alginate)	Membrane proteins		
PA2307		probable permease of ABC transporter	4,81	Membrane proteins	Transport of small molecules		
PA2308		probable ATP-binding component of ABC transporter	3,24	Transport of small molecules			
PA2309		hypothetical protein	3,81	Hypothetical, unclassified, unknown			
PA2310		hypothetical protein	4,75	Putative enzymes			
PA2311		hypothetical protein	9,55	Hypothetical, unclassified, unknown			

PA2312		probable transcriptional regulator	7,28	Transcriptional regulators			
PA2313		hypothetical protein	2,95	Membrane proteins			
PA2314		probable major facilitator superfamily (MFS) transporter	6,57	Membrane proteins	Transport of small molecules		
PA2315		hypothetical protein	15,99	Hypothetical, unclassified, unknown			
PA2318		hypothetical protein	-5,10	Hypothetical, unclassified, unknown			
PA2319		probable transposase	2,23	Relative to phage, transposon, or plasmid			
PA2320	*gntR*	transcriptional regulator GntR	2,08	Transcriptional regulators			
PA2321	*gntK*	GntK	8,63	Energy metabolism	Carbon compound catabolism		
PA2322	*gntP*	GntP	6,01	Transport of small molecules			
PA2323	*gapN*	GapN	-3,25	Carbon compound catabolism			
PA2324		hypothetical protein	2,62	Putative enzymes			
PA2326		hypothetical protein	4,60	Putative enzymes			
PA2327		probable permease of ABC transporter	-3,08	Membrane proteins	Transport of small molecules		
PA2328		hypothetical protein	-13,16	Hypothetical, unclassified, unknown			
PA2329		probable ATP-binding component of ABC transporter	-9,90	Transport of small molecules			
PA2330		hypothetical protein	-10,42	Hypothetical, unclassified, unknown			
PA2331		hypothetical protein	-10,87	Membrane proteins			
PA2334		probable transcriptional regulator	7,91	Transcriptional regulators			
PA2335		probable TonB-dependent receptor	14,53	Transport of small molecules			
PA2339		probable binding-protein-dependent maltose/mannitol transport protein	2,26	Transport of small molecules			
PA2343	*mtlY*	xylulose kinase	2,41	Carbon compound catabolism			
PA2345		conserved hypothetical protein	-6,21	Hypothetical, unclassified, unknown			
PA2346		conserved hypothetical protein	7,66	Putative enzymes			
PA2347		hypothetical protein	6,32	Putative enzymes			
PA2348		conserved hypothetical protein	9,49	Putative enzymes			
PA2350		probable ATP-binding component of ABC transporter	2,06	Transport of small molecules			
PA2351		probable permease of ABC transporter	3,32	Membrane proteins	Transport of small molecules		
PA2354	*sfnR1*	SfnR1	3,83	Transcriptional regulators			
PA2355		probable FMNH2-dependent monooxygenase	4,66	Putative enzymes			
PA2356	*msuD*	methanesulfonate sulfonatase MsuD	7,66	Carbon compound catabolism	Central intermediary metabolism		
PA2357	*msuE*	NADH-dependent FMN reductase MsuE	5,70	Carbon compound catabolism	Central intermediary metabolism		
PA2359	*sfnR2*	probable transcriptional regulator	2,54	Transcriptional regulators			x
PA2360	*hsiA3*	hypothetical protein	-11,49	Hypothetical, unclassified, unknown			x
PA2361	*icmF3*	IcmF3	-2,62	Hypothetical, unclassified, unknown			x
PA2363	*hsiJ3*	HsiJ3	-3,85	Hypothetical, unclassified, unknown			x
PA2365	*hsiB3*	HsiB3	-35,71	Hypothetical, unclassified, unknown			x
PA2366	*hsiC3*	HsiC3	-47,62	Hypothetical, unclassified, unknown			x
PA2367	*hcp3*	Hcp3	-55,56	Hypothetical, unclassified, unknown			x
PA2368	*hsiF3*	HsiF3	-66,67	Hypothetical, unclassified, unknown			x
PA2369	*hsiG3*	HsiG3	-32,26	Membrane proteins			x
PA2370	*hsiH3*	HsiH3	-41,67	Hypothetical, unclassified, unknown			x
PA2371	*clpV3*	ClpV3	-25,64	Translation, post-translational modification, degradation			x
PA2372		hypothetical protein	-17,24	Hypothetical, unclassified, unknown			x
PA2373	*vgrG3*	VgrG3	-7,46	Hypothetical, unclassified, unknown			x
PA2374	*tseF*	TseF	-7,58	Hypothetical, unclassified, unknown			
PA2375		hypothetical protein	-2,20	Membrane proteins			
PA2376		probable transcriptional regulator	6,43	Transcriptional regulators			
PA2377		hypothetical protein	7,68	Hypothetical, unclassified, unknown			
PA2381		hypothetical protein	-37,04	Hypothetical, unclassified, unknown			
PA2384		hypothetical protein	-4,13	Hypothetical, unclassified, unknown			
PA2385	*pvdQ*	3-oxo-C12-homoserine lactone acylase PvdQ	-8,47	Adaptation, Protection			x
PA2386	*pvdA*	L-ornithine N5-oxygenase	-13,16	Adaptation, Protection			x
PA2389	*pvdR*	PvdR	-2,37	Transport of small molecules			
PA2390	*pvdT*	PvdT	-4,61	Transport of small molecules	Membrane proteins		
PA2391	*opmQ*	probable outer membrane protein precursor	-8,70	Transport of small molecules	Membrane proteins		
PA2392	*pvdP*	PvdP	-3,26	Adaptation, Protection			x
PA2393	*pvdM*	putative dipeptidase	-12,35	Central intermediary metabolism			x
PA2394	*pvdN*	PvdN	-14,49	Adaptation, Protection			x
PA2395	*pvdO*	PvdO	-8,47	Adaptation, Protection			x
PA2396	*pvdF*	pyoverdine synthetase F	-5,46	Adaptation, Protection	Secreted Factors (toxins, enzymes, alginate)		x
PA2397	*pvdE*	pyoverdine biosynthesis protein PvdE	-13,51	Adaptation, Protection	Membrane proteins	Transport of small molecules	x
PA2398	*fpvA*	ferripyoverdine receptor	-8,13	Transport of small molecules			x
PA2402	*pvdI*	probable non-ribosomal peptide synthetase	-2,07	Putative enzymes			x
PA2403	*fpvG*	FpvG	-2,90	Membrane proteins			
PA2404	*fpvH*	FpvH	-4,95	Membrane proteins			
PA2405	*fpvJ*	FpvJ	-5,52	Hypothetical, unclassified, unknown			
PA2406	*fpvK*	FpvK	-5,13	Hypothetical, unclassified, unknown			
PA2407	*fpvC*	FpvC	-5,71	Transport of small molecules	Motility and Attachment		
PA2408	*fpvD*	FpvD	-5,46	Transport of small molecules			
PA2409	*fpvE*	FpvE	-5,99	Transport of small molecules	Membrane proteins		
PA2410	*fpvF*	FpvF	-4,65	Transport of small molecules			
PA2411		probable thioesterase	-5,10	Adaptation, Protection	Putative enzymes		
PA2412	*mbtH*	conserved hypothetical protein	-5,26	Hypothetical, unclassified, unknown			
PA2413	*pvdH*	L-2,4-diaminobutyrate:2-ketoglutarate 4-aminotransferase, PvdH	-3,85	Adaptation, Protection			x
PA2414		L-sorbosone dehydrogenase	2,13	Carbon compound catabolism			
PA2415		hypothetical protein	4,78	Membrane proteins			
PA2417		probable transcriptional regulator	9,15	Transcriptional regulators			
PA2419		probable hydrolase	-3,79	Putative enzymes			
PA2420	*opdJ*	probable porin	3,23	Membrane proteins	Transport of small molecules		
PA2422		hypothetical protein	-2,62	Hypothetical, unclassified, unknown			
PA2423		hypothetical protein	-4,52	Hypothetical, unclassified, unknown			
PA2424	*pvdL*	PvdL	-6,54	Adaptation, Protection			x
PA2425	*pvdG*	PvdG	-6,67	Adaptation, Protection			x
PA2426	*pvdS*	sigma factor PvdS	-33,33	Transcriptional regulators			x
PA2427		hypothetical protein	-6,13	Hypothetical, unclassified, unknown			x
PA2429		hypothetical protein	2,76	Hypothetical, unclassified, unknown			
PA2430		conserved hypothetical protein	-2,81	Hypothetical, unclassified, unknown			
PA2432	*bexR*	bistable expression regulator, BexR	2,50	Transcriptional regulators			
PA2434		hypothetical protein	2,38	Hypothetical, unclassified, unknown			
PA2439		hypothetical protein	2,89	Membrane proteins			
PA2440		hypothetical protein	3,57	Hypothetical, unclassified, unknown			
PA2441		hypothetical protein	2,13	Hypothetical, unclassified, unknown			
PA2442	*gcvT2*	glycine cleavage system protein T2	-6,80	Amino acid biosynthesis and metabolism	Central intermediary metabolism		
PA2443	*sdaA*	L-serine dehydratase	-5,92	Amino acid biosynthesis and metabolism			
PA2444	*glyA2*	serine hydroxymethyltransferase	-7,19	Amino acid biosynthesis and metabolism			
PA2445	*gcvP2*	glycine cleavage system protein P2	-6,33	Amino acid biosynthesis and metabolism	Central intermediary metabolism		
PA2446	*gcvH2*	glycine cleavage system protein H2	-3,76	Amino acid biosynthesis and metabolism			
PA2447		probable transcriptional regulator	3,18	Transcriptional regulators			
PA2448		hypothetical protein	-10,87	Hypothetical, unclassified, unknown			
PA2449	*gcsR*	GcsR	3,02	Transcriptional regulators			
PA2450		hypothetical protein	4,15	Hypothetical, unclassified, unknown			
PA2455		hypothetical protein	-2,44	Hypothetical, unclassified, unknown			
PA2456		hypothetical protein	-3,21	Hypothetical, unclassified, unknown			
PA2457		hypothetical protein	2,03	Hypothetical, unclassified, unknown			
PA2460		hypothetical protein	2,63	Hypothetical, unclassified, unknown			
PA2462		hypothetical protein	2,62	Hypothetical, unclassified, unknown			x
PA2463		hypothetical protein	7,31	Hypothetical, unclassified, unknown			
PA2465		hypothetical protein	2,20	Membrane proteins			
PA2466	*foxA*	Ferrioxamine receptor FoxA	-3,07	Transport of small molecules			
PA2467	*foxR*	Anti-sigma factor FoxR	-3,80	Membrane proteins	Transcriptional regulators		
PA2468	*foxI*	ECF sigma factor FoxI	-4,35	Transcriptional regulators			
PA2469		probable transcriptional regulator	2,08	Transcriptional regulators			
PA2470	*gtdA*	gentisate 1,2-dioxygenase	4,08	Carbon compound catabolism			
PA2471		conserved hypothetical protein	-2,98	Hypothetical, unclassified, unknown			
PA2472		probable major facilitator superfamily (MFS) transporter	12,29	Membrane proteins	Transport of small molecules		
PA2475		probable cytochrome P450	-2,53	Adaptation, Protection	Putative enzymes		
PA2476	*dsbG*	thiol:disulfide interchange protein DsbG	-2,74	Translation, post-translational modification, degradation	Chaperones & heat shock proteins		
PA2477		probable thiol:disulfide interchange protein	2,63	Membrane proteins	Chaperones & heat shock proteins	Putative enzymes	
PA2478		probable thiol:disulfide interchange protein	2,48	Membrane proteins	Chaperones & heat shock proteins	Putative enzymes	
PA2481		hypothetical protein	5,29	Hypothetical, unclassified, unknown			
PA2482		probable cytochrome c	4,07	Energy metabolism			
PA2485		hypothetical protein	13,74	Hypothetical, unclassified, unknown			
PA2486	*ptrC*	Pseudomonas type III repressor gene C, PtrC	5,69	Hypothetical, unclassified, unknown			
PA2487		hypothetical protein	4,40	Hypothetical, unclassified, unknown			
PA2488		probable transcriptional regulator	3,84	Transcriptional regulators			
PA2491	*mexS*	MexS	-2,05	Transcriptional regulators	Putative enzymes		
PA2492	*mexT*	transcriptional regulator MexT	-2,38	Transcriptional regulators			
PA2493	*mexE*	Resistance-Nodulation-Cell Division (RND) multidrug efflux membrane fusion protein M	7,51	Antibiotic resistance and susceptibility	Transport of small molecules		
PA2494	*mexF*	Resistance-Nodulation-Cell Division (RND) multidrug efflux transporter MexF	9,97	Antibiotic resistance and susceptibility	Transport of small molecules	Membrane proteins	
PA2495	*oprN*	Multidrug efflux outer membrane protein OprN precursor	13,37	Antibiotic resistance and susceptibility	Transport of small molecules	Membrane proteins	
PA2496		hypothetical protein	2,22	Hypothetical, unclassified, unknown			
PA2499		probable deaminase	-2,05	Putative enzymes			
PA2503		hypothetical protein	2,99	Hypothetical, unclassified, unknown			
PA2504		hypothetical protein	-8,26	Hypothetical, unclassified, unknown			
PA2505	*opdT*	tyrosine porin OpdT	7,39	Membrane proteins	Transport of small molecules		
PA2506		hypothetical protein	4,78	Hypothetical, unclassified, unknown			
PA2507	*catA*	catechol 1,2-dioxygenase	21,12	Carbon compound catabolism			
PA2508	*catC*	muconolactone delta-isomerase	25,61	Carbon compound catabolism			
PA2509	*catB*	muconate cycloisomerase I	42,83	Carbon compound catabolism			
PA2510	*catR*	transcriptional regulator CatR	2,86	Transcriptional regulators	Carbon compound catabolism		
PA2515	*xylL*	cis-1,2-dihydroxycyclohexa-3,4-diene carboxylate dehydrogenase	2,45	Carbon compound catabolism			
PA2516	*xylZ*	toluate 1,2-dioxygenase electron transfer component	3,55	Energy metabolism	Carbon compound catabolism		
PA2517	*xylY*	toluate 1,2-dioxygenase beta subunit	9,20	Carbon compound catabolism			
PA2518	*xylX*	toluate 1,2-dioxygenase alpha subunit	5,33	Carbon compound catabolism			
PA2519	*xylS*	transcriptional regulator XylS	3,34	Transcriptional regulators			
PA2520	*czcA*	Resistance-Nodulation-Cell Division (RND) divalent metal cation efflux transporter CzcA	13,93	Membrane proteins	Transport of small molecules		
PA2521	*czcB*	Resistance-Nodulation-Cell Division (RND) divalent metal cation efflux membrane fusio	22,24	Membrane proteins	Transport of small molecules		
PA2522	*czcC*	outer membrane protein precursor CzcC	28,97	Membrane proteins	Transport of small molecules		
PA2523	*czcR*	CzcR	3,46	Two-component regulatory systems	Transcriptional regulators		x
PA2524	*czcS*	CzcS	4,61	Two-component regulatory systems			
PA2527	*muxB*	MuxB	6,06	Antibiotic resistance and susceptibility	Transport of small molecules	Membrane proteins	
PA2528	*muxA*	MuxA	13,56	Antibiotic resistance and susceptibility	Transport of small molecules		x
PA2529		hypothetical protein	-3,91	Hypothetical, unclassified, unknown			
PA2530		hypothetical protein	-5,35	Hypothetical, unclassified, unknown			
PA2531		probable aminotransferase	-10,00	Amino acid biosynthesis and metabolism			
PA2532	*tpx*	thiol peroxidase	-3,73	Adaptation, Protection			
PA2536		probable phosphatidate cytidylyltransferase	-4,85	Fatty acid and phospholipid metabolism			
PA2537		probable acyltransferase	-3,94	Fatty acid and phospholipid metabolism			
PA2540		conserved hypothetical protein	2,18	Hypothetical, unclassified, unknown			
PA2541		probable CDP-alcohol phosphatidyltransferase	2,48	Putative enzymes			
PA2542		conserved hypothetical protein	2,50	Hypothetical, unclassified, unknown			
PA2543		conserved hypothetical protein	2,17	Hypothetical, unclassified, unknown			
PA2544		hypothetical protein	-2,85	Hypothetical, unclassified, unknown			
PA2545	*xthA*	exodeoxyribonuclease III	-2,47	DNA replication, recombination, modification and repair			
PA2546		probable ring-cleaving dioxygenase	2,87	Putative enzymes			
PA2548		hypothetical protein	3,03	Hypothetical, unclassified, unknown			
PA2549		conserved hypothetical protein	2,57	Membrane proteins			
PA2552		probable acyl-CoA dehydrogenase	-4,98	Putative enzymes			
PA2553		probable acyl-CoA thiolase	-3,24	Putative enzymes			
PA2558		probable transport protein	2,97	Membrane proteins	Transport of small molecules		
PA2559		Lipoprotein	2,07	Hypothetical, unclassified, unknown			
PA2560		hypothetical protein	3,69	Hypothetical, unclassified, unknown			
PA2561	*ctpH*	CtpH	10,68	Adaptation, Protection	Chemotaxis		
PA2563		probable sulfate transporter	7,86	Membrane proteins	Transport of small molecules		
PA2564		hypothetical protein	-2,66	Hypothetical, unclassified, unknown			
PA2565		hypothetical protein	-2,52	Hypothetical, unclassified, unknown			
PA2566		conserved hypothetical protein	-4,12	Hypothetical, unclassified, unknown			
PA2568		hypothetical protein	8,19	Membrane proteins			
PA2569		hypothetical protein	12,97	Hypothetical, unclassified, unknown			
PA2570	*lecA*	LecA	-4,37	Cell Wall / LPS / Capsule	Adaptation, Protection	Motility and Attachment	
PA2570.1		tRNA-Leu	6,59	Non-coding RNA gene			
PA2571		probable two-component sensor	2,68	Two-component regulatory systems			
PA2572		probable two-component response regulator	-13,33	Two-component regulatory systems	Transcriptional regulators		x
PA2573		probable chemotaxis transducer	-4,15	Adaptation, Protection	Chemotaxis		x
PA2574	*alkB1*	alkane-1-monooxygenase	5,22	Carbon compound catabolism			
PA2575		hypothetical protein	-8,20	Hypothetical, unclassified, unknown			
PA2576		hypothetical protein	5,29	Membrane proteins			
PA2577		probable transcriptional regulator	-2,71	Transcriptional regulators			
PA2578		probable acetyltransferase	-2,34	Putative enzymes			
PA2581.1		tRNA-Cys	5,32	Non-coding RNA gene			
PA2583		probable sensor/response regulator hybrid	16,25	Two-component regulatory systems	Transcriptional regulators		
PA2583.1		tRNA-Gly	3,27	Non-coding RNA gene			
PA2587	*pqsH*	probable FAD-dependent monooxygenase	-7,04	Biosynthesis of cofactors, prosthetic groups and carrier			
PA2588		probable transcriptional regulator	-8,33	Transcriptional regulators			
PA2589		hypothetical protein	10,24	Membrane proteins			
PA2590		hypothetical protein	20,07	Hypothetical, unclassified, unknown			
PA2592		probable periplasmic spermidine/putrescine-binding protein	-3,33	Transport of small molecules			
PA2594		conserved hypothetical protein	2,05	Hypothetical, unclassified, unknown			
PA2595		conserved hypothetical protein	3,34	Hypothetical, unclassified, unknown			
PA2596		conserved hypothetical protein	2,17	Hypothetical, unclassified, unknown			
PA2599		conserved hypothetical protein	4,99	Hypothetical, unclassified, unknown			
PA2600		hypothetical protein	12,37	Hypothetical, unclassified, unknown			
PA2601		probable transcriptional regulator	6,64	Transcriptional regulators			
PA2602		3-mercaptopropionate dioxygenase	-2,50	Hypothetical, unclassified, unknown			
PA2603.1		tRNA-Ser	22,60	Non-coding RNA gene			
PA2604		conserved hypothetical protein	-3,28	Hypothetical, unclassified, unknown			
PA2605		conserved hypothetical protein	-4,81	Hypothetical, unclassified, unknown			
PA2606		conserved hypothetical protein	-6,85	Hypothetical, unclassified, unknown			
PA2607		conserved hypothetical protein	-8,62	Hypothetical, unclassified, unknown			
PA2608		conserved hypothetical protein	-11,36	Hypothetical, unclassified, unknown			
PA2609		hypothetical protein	-14,71	Hypothetical, unclassified, unknown			
PA2610		conserved hypothetical protein	-2,15	Hypothetical, unclassified, unknown			
PA2611	*cysG*	siroheme synthase	-2,54	Biosynthesis of cofactors, prosthetic groups and carrier			
PA2612	*serS*	seryl-tRNA synthetase	-2,25	Translation, post-translational modification, degradation	Amino acid biosynthesis and metabolism		
PA2615	*ftsK*	cell division protein FtsK	2,82	Antibiotic resistance and susceptibility	Cell division		
PA2616	*trxB1*	thioredoxin reductase 1	-2,91	Nucleotide biosynthesis and metabolism			
PA2617	*aat*	leucyl/phenylalanyl-tRNA-protein transferase	-3,55	Translation, post-translational modification, degradation			
PA2618		hypothetical protein	-5,00	Translation, post-translational modification, degradation			
PA2622	*cspD*	cold-shock protein CspD	-10,99	Adaptation, Protection	Transcriptional regulators		
PA2623	*icd*	isocitrate dehydrogenase	-5,03	Amino acid biosynthesis and metabolism	Energy metabolism	Carbon compound catabolism	
PA2624	*idh*	isocitrate dehydrogenase	-2,09	Energy metabolism			
PA2625		conserved hypothetical protein	-3,72	Hypothetical, unclassified, unknown			
PA2630		conserved hypothetical protein	-2,79	Hypothetical, unclassified, unknown			
PA2631		probable acetyl transferase	-2,11	Putative enzymes			
PA2633		hypothetical protein	-4,63	Relative to phage, transposon, or plasmid			
PA2635		hypothetical protein	3,16	Hypothetical, unclassified, unknown			
PA2636		hypothetical protein	3,99	Hypothetical, unclassified, unknown			
PA2638	*nuoB*	NADH dehydrogenase I chain B	-3,73	Energy metabolism			
PA2639	*nuoD*	NADH dehydrogenase I chain C,D	-7,69	Energy metabolism			x
PA2640	*nuoE*	NADH dehydrogenase I chain E	-10,53	Energy metabolism			
PA2641	*nuoF*	NADH dehydrogenase I chain F	-9,62	Energy metabolism			
PA2642	*nuoG*	NADH dehydrogenase I chain G	-8,70	Energy metabolism			
PA2643	*nuoH*	NADH dehydrogenase I chain H	-8,20	Energy metabolism			
PA2644	*nuoI*	NADH Dehydrogenase I chain I	-10,31	Energy metabolism			
PA2645	*nuoJ*	NADH dehydrogenase I chain J	-9,90	Energy metabolism			
PA2646	*nuoK*	NADH dehydrogenase I chain K	-9,80	Energy metabolism			
PA2647	*nuoL*	NADH dehydrogenase I chain L	-12,50	Energy metabolism			
PA2648	*nuoM*	NADH dehydrogenase I chain M	-10,42	Energy metabolism			
PA2649	*nuoN*	NADH dehydrogenase I chain N	-9,17	Energy metabolism	Antibiotic resistance and susceptibility		
PA2650		conserved hypothetical protein	-4,35	Hypothetical, unclassified, unknown			
PA2651		conserved hypothetical protein	2,08	Hypothetical, unclassified, unknown			
PA2652		methyl-accepting chemotaxis protein	2,17	Adaptation, Protection	Chemotaxis		
PA2653		probable transporter	6,74	Membrane proteins	Transport of small molecules		
PA2654	*tlpQ*	TlpQ	7,24	Adaptation, Protection	Chemotaxis		
PA2655		hypothetical protein	7,28	Hypothetical, unclassified, unknown			
PA2656	*bqsS*	two-component sensor BqsS	3,58	Two-component regulatory systems			
PA2659		hypothetical protein	-2,31	Hypothetical, unclassified, unknown			
PA2664	*fhp*	flavohemoprotein	2,22	Energy metabolism			
PA2665	*fhpR*	Transcriptional activator of P. aeruginosa flavohemoglobin, FhpR	3,59	Adaptation, Protection	Transcriptional regulators		
PA2668		hypothetical protein	-2,72	Hypothetical, unclassified, unknown			
PA2669		hypothetical protein	3,40	Hypothetical, unclassified, unknown			
PA2670		hypothetical protein	3,40	Hypothetical, unclassified, unknown			
PA2671		hypothetical protein	10,96	Hypothetical, unclassified, unknown			
PA2672		probable type II secretion system protein	10,73	Protein secretion/export apparatus			
PA2673		probable type II secretion system protein	4,05	Protein secretion/export apparatus			
PA2674		probable type II secretion system protein	2,81	Protein secretion/export apparatus			
PA2675		probable type II secretion system protein	2,78	Protein secretion/export apparatus			
PA2676		probable type II secretion system protein	2,40	Protein secretion/export apparatus			
PA2677		probable type II secretion protein	2,21	Protein secretion/export apparatus			
PA2678		probable permease of ABC-2 transporter	19,56	Membrane proteins	Transport of small molecules		
PA2679		hypothetical protein	5,47	Hypothetical, unclassified, unknown			
PA2685	*vgrG4*	VgrG4	2,63	Protein secretion/export apparatus			
PA2686	*pfeR*	two-component response regulator PfeR	-3,30	Two-component regulatory systems	Transcriptional regulators		
PA2690		probable transposase	2,19	Relative to phage, transposon, or plasmid			
PA2692		probable transcriptional regulator	2,46	Transcriptional regulators			
PA2693		conserved hypothetical protein	5,85	Hypothetical, unclassified, unknown			
PA2698		probable hydrolase	-2,07	Putative enzymes			
PA2699		hypothetical protein	-12,50	Hypothetical, unclassified, unknown			
PA2701		probable major facilitator superfamily (MFS) transporter	-2,75	Membrane proteins	Transport of small molecules		
PA2703	*tsi2*	Tsi2	-2,79	Adaptation, Protection			
PA2704		probable transcriptional regulator	2,76	Transcriptional regulators			
PA2707		hypothetical protein	-2,49	Hypothetical, unclassified, unknown			
PA2708		hypothetical protein	4,19	Hypothetical, unclassified, unknown			
PA2709	*cysK*	cysteine synthase A	-5,18	Amino acid biosynthesis and metabolism			
PA2710		hypothetical protein	-5,95	Membrane proteins			
PA2714		probable molybdopterin oxidoreductase	2,48	Energy metabolism			
PA2717	*cpo*	chloroperoxidase precursor	-8,00	Central intermediary metabolism			
PA2721		hypothetical protein	-2,62	Hypothetical, unclassified, unknown			
PA2722		hypothetical protein	-4,50	Hypothetical, unclassified, unknown			
PA2726		probable radical activating enzyme	-2,86	Central intermediary metabolism			
PA2727		hypothetical protein	-4,95	Hypothetical, unclassified, unknown			
PA2728		hypothetical protein	2,48	Hypothetical, unclassified, unknown			
PA2729		hypothetical protein	-2,25	Hypothetical, unclassified, unknown			
PA2730		hypothetical protein	-6,94	Hypothetical, unclassified, unknown			
PA2731		Uncharacterized protein	-4,57	Hypothetical, unclassified, unknown			
PA2732		hypothetical protein	-2,67	Hypothetical, unclassified, unknown			
PA2733		conserved hypothetical protein	-2,72	Hypothetical, unclassified, unknown			
PA2734		hypothetical protein	-3,38	Hypothetical, unclassified, unknown			
PA2741	*rplT*	50S ribosomal protein L20	3,07	Translation, post-translational modification, degradation			
PA2742	*rpmI*	50S ribosomal protein L35	2,94	Translation, post-translational modification, degradation			
PA2743	*infC*	translation initiation factor IF-3	2,08	Translation, post-translational modification, degradation			

PA2744.1	*P13*	P13	65,26	Non-coding RNA gene			
PA2745		probable hydrolase	19,93	Putative enzymes			
PA2746		hypothetical protein	-3,79	Membrane proteins			
PA2748		probable methionine aminopeptidase	4,72	Translation, post-translational modification, degradation			
PA2749	*endA*	DNA-specific endonuclease I	5,52	DNA replication, recombination, modification and repair			
PA2752		conserved hypothetical protein	4,21	Membrane proteins			
PA2753		hypothetical protein	-2,45	Hypothetical, unclassified, unknown			
PA2754		conserved hypothetical protein	-2,16	Hypothetical, unclassified, unknown			
PA2755	*eco*	ecotin precursor	2,40	Translation, post-translational modification, degradation			
PA2756		hypothetical protein	-2,07	Hypothetical, unclassified, unknown			
PA2757		hypothetical protein	2,23	Membrane proteins			
PA2758		probable transcriptional regulator	4,52	Transcriptional regulators			
PA2760	*oprQ*	OprQ	-3,33	Antibiotic resistance and susceptibility	Transport of small molecules	Motility and Attachment	
PA2761		hypothetical protein	-4,17	Membrane proteins			
PA2762		hypothetical protein	3,39	Hypothetical, unclassified, unknown			
PA2763		hypothetical protein	2,52	Membrane proteins			
PA2764		hypothetical protein	-4,29	Hypothetical, unclassified, unknown			
PA2766		probable transcriptional regulator	4,01	Transcriptional regulators			
PA2767		probable enoyl-CoA hydratase/isomerase	6,17	Putative enzymes			
PA2768		hypothetical protein	4,87	Membrane proteins			
PA2769		hypothetical protein	2,95	Hypothetical, unclassified, unknown			
PA2770		hypothetical protein	2,05	Hypothetical, unclassified, unknown			
PA2771		diguanylate cyclase with a self-blocked I-site, Dcsbis	-27,03	Motility and Attachment			
PA2772		hypothetical protein	4,10	Hypothetical, unclassified, unknown			
PA2773		hypothetical protein	8,96	Membrane proteins			
PA2774	*tse4*	Tse4	2,76	Membrane proteins	Secreted Factors (toxins, enzymes, alginate)		
PA2775	*tsi4*	Tsi4	3,38	Membrane proteins	Adaptation, Protection		
PA2775.1		tRNA-Val	22,38	Non-coding RNA gene			
PA2776	*pauB3*	FAD-dependent oxidoreductase	4,13	Carbon compound catabolism			
PA2777		conserved hypothetical protein	17,79	Membrane proteins			
PA2778		hypothetical protein	8,53	Hypothetical, unclassified, unknown			
PA2779		hypothetical protein	4,86	Hypothetical, unclassified, unknown			
PA2782	*bamI*	biofilm-associated metzincin Inhibitor, BamI	3,86	Hypothetical, unclassified, unknown			
PA2783	*mep72*	Mep72	3,19	Hypothetical, unclassified, unknown			
PA2787	*cpg2*	carboxypeptidase G2 precursor	-4,27	Central intermediary metabolism			
PA2788		probable chemotaxis transducer	-2,08	Adaptation, Protection	Chemotaxis		
PA2789		hypothetical protein	6,72	Hypothetical, unclassified, unknown			
PA2791		hypothetical protein	7,43	Hypothetical, unclassified, unknown			
PA2792		hypothetical protein	2,01	Hypothetical, unclassified, unknown			
PA2795	*dusA*	dihydrouridine synthase A	3,39	Hypothetical, unclassified, unknown			
PA2796	*tal*	transaldolase	-2,97	Energy metabolism	Carbon compound catabolism		
PA2800	*vacJ*	VacJ	-2,65	Antibiotic resistance and susceptibility			
PA2803		hypothetical protein	10,07	Hypothetical, unclassified, unknown			
PA2804		hypothetical protein	2,36	Hypothetical, unclassified, unknown			
PA2805		hypothetical protein	-5,10	Hypothetical, unclassified, unknown			
PA2807		hypothetical protein	2,13	Hypothetical, unclassified, unknown			
PA2808	*ptrA*	Pseudomonas type III repressor A	11,12	Adaptation, Protection	Transcriptional regulators		
PA2811		probable permease of ABC-2 transporter	-4,17	Membrane proteins	Transport of small molecules		
PA2812		probable ATP-binding component of ABC transporter	-3,33	Transport of small molecules			
PA2814		hypothetical protein	10,37	Hypothetical, unclassified, unknown			
PA2815		probable acyl-CoA dehydrogenase	-3,66	Putative enzymes			
PA2816		hypothetical protein	-3,39	Hypothetical, unclassified, unknown			
PA2818	*arr*	aminoglycoside response regulator	13,81	Antibiotic resistance and susceptibility	Nucleotide biosynthesis and metabolism		
PA2819		hypothetical protein	7,40	Hypothetical, unclassified, unknown			
PA2819.1		tRNA-Gly	3,24	Non-coding RNA gene			
PA2819.2		tRNA-Gly	3,40	Non-coding RNA gene			
PA2819.3		tRNA-Glu	3,70	Non-coding RNA gene			
PA2821		probable glutathione S-transferase	-2,76	Central intermediary metabolism			
PA2826		probable glutathione peroxidase	2,00	Adaptation, Protection			
PA2829		hypothetical protein	-2,70	Hypothetical, unclassified, unknown			
PA2831		conserved hypothetical protein	-2,99	Hypothetical, unclassified, unknown			
PA2838		probable transcriptional regulator	2,11	Transcriptional regulators			
PA2840		probable ATP-dependent RNA helicase	32,64	Transcription, RNA processing and degradation			
PA2841		probable enoyl-CoA hydratase/isomerase	-5,24	Putative enzymes			
PA2843		probable aldolase	-2,26	Putative enzymes			
PA2845		hypothetical protein	15,43	Hypothetical, unclassified, unknown			
PA2846		probable transcriptional regulator	-2,10	Transcriptional regulators			
PA2847		conserved hypothetical protein	9,29	Hypothetical, unclassified, unknown			
PA2850	*ohr*	organic hydroperoxide resistance protein	5,61	Adaptation, Protection			
PA2852.1		tRNA-Ser	9,54	Non-coding RNA gene			
PA2853	*oprI*	Outer membrane lipoprotein OprI precursor	-2,87	Membrane proteins			
PA2855		hypothetical protein	3,47	Hypothetical, unclassified, unknown			
PA2858		conserved hypothetical protein	-2,06	Membrane proteins			
PA2859	*greB*	transcription elongation factor GreB	-2,04	Transcription, RNA processing and degradation			
PA2860		hypothetical protein	5,39	Hypothetical, unclassified, unknown			
PA2861	*ligT*	2'-5' RNA ligase	3,88	Transcription, RNA processing and degradation			
PA2862	*lipA*	lactonizing lipase precursor	2,02	Secreted Factors (toxins, enzymes, alginate)	Carbon compound catabolism		
PA2864		conserved hypothetical protein	-4,98	Hypothetical, unclassified, unknown			
PA2865		probable glycosylase	7,33	Putative enzymes			
PA2867		probable chemotaxis transducer	2,01	Adaptation, Protection	Chemotaxis		
PA2868		hypothetical protein	2,94	Membrane proteins			
PA2869		hypothetical protein	6,92	Hypothetical, unclassified, unknown			
PA2870		diguanylate cyclase	2,96	Nucleotide biosynthesis and metabolism			
PA2872		hypothetical protein	-2,13	Hypothetical, unclassified, unknown			
PA2881		probable two-component response regulator	2,19	Two-component regulatory systems	Transcriptional regulators		
PA2882		probable two-component sensor	19,46	Two-component regulatory systems			
PA2886	*atuA*	expressed protein with apparent function in citronellol catabolism	6,07	Hypothetical, unclassified, unknown			
PA2887	*atuB*	putative dehydrogenase involved in catabolism of citronellol	3,72	Putative enzymes			
PA2894		hypothetical protein	2,07	Hypothetical, unclassified, unknown			
PA2895	*sbrR*	SbrR	2,90	Transcriptional regulators			x
PA2896	*sbrI*	SbrI	5,00	Transcriptional regulators			
PA2898		hypothetical protein	8,43	Hypothetical, unclassified, unknown			
PA2899	*atvR*	atypical virulence-related response regulator AtvR	2,72	Transcriptional regulators			
PA2900		probable outer membrane lipoprotein precursor	2,95	Membrane proteins	Transport of small molecules		
PA2901		lipoprotein	2,52	Hypothetical, unclassified, unknown			
PA2906		probable oxidoreductase	3,87	Putative enzymes			
PA2910		conserved hypothetical protein	11,58	Membrane proteins			
PA2911		probable TonB-dependent receptor	7,95	Membrane proteins	Transport of small molecules		
PA2913		hypothetical protein	2,53	Transport of small molecules			
PA2914		probable permease of ABC transporter	5,32	Membrane proteins	Transport of small molecules		
PA2915		hypothetical protein	-2,62	Hypothetical, unclassified, unknown			
PA2916		hypothetical protein	4,08	Membrane proteins			
PA2917		probable transcriptional regulator	2,14	Transcriptional regulators			
PA2918		probable short-chain dehydrogenase	-2,18	Putative enzymes			
PA2920		probable chemotaxis transducer	3,07	Adaptation, Protection	Chemotaxis		
PA2921		probable transcriptional regulator	3,85	Transcriptional regulators			
PA2922		probable hydrolase	3,37	Putative enzymes			
PA2923	*hisJ*	periplasmic histidine-binding protein HisJ	6,04	Amino acid biosynthesis and metabolism	Transport of small molecules		
PA2927		hypothetical protein	-3,70	Hypothetical, unclassified, unknown			
PA2928		hypothetical protein	3,89	Hypothetical, unclassified, unknown			
PA2929		hypothetical protein	6,77	Membrane proteins			
PA2930		probable transcriptional regulator	3,77	Transcriptional regulators			
PA2931	*cifR*	CifR	3,34	Transcriptional regulators			
PA2933		probable major facilitator superfamily (MFS) transporter	3,10	Membrane proteins	Transport of small molecules		
PA2935		hypothetical protein	11,66	Hypothetical, unclassified, unknown			
PA2936		hypothetical protein	2,44	Membrane proteins			
PA2937		hypothetical protein	-5,99	Hypothetical, unclassified, unknown			
PA2938		probable transporter	8,47	Membrane proteins	Transport of small molecules		
PA2939	*paaP*	probable aminopeptidase	-50,00	Secreted Factors (toxins, enzymes, alginate)			
PA2940		probable acyl-CoA thiolase	4,21	Putative enzymes			
PA2941		hypothetical protein	2,12	Hypothetical, unclassified, unknown			
PA2942.1	*P15*	P15	2,08	Non-coding RNA gene			
PA2944	*cobN*	cobalamin biosynthetic protein CobN	3,61	Biosynthesis of cofactors, prosthetic groups and carrier			
PA2945		conserved hypothetical protein	2,05	Biosynthesis of cofactors, prosthetic groups and carrier			
PA2948	*cobM*	precorrin-3 methylase	-2,32	Biosynthesis of cofactors, prosthetic groups and carrier			
PA2949		esterase	-3,07	Fatty acid and phospholipid metabolism	Putative enzymes		
PA2951	*etfA*	electron transfer flavoprotein alpha-subunit	-2,15	Energy metabolism			
PA2954		hypothetical protein	3,90	Hypothetical, unclassified, unknown			
PA2955		Lipoprotein	4,51	Hypothetical, unclassified, unknown			
PA2956		conserved hypothetical protein	2,38	Hypothetical, unclassified, unknown			
PA2963		conserved hypothetical protein	3,32	Hypothetical, unclassified, unknown			
PA2965	*fabF1*	beta-ketoacyl-acyl carrier protein synthase II	-2,03	Fatty acid and phospholipid metabolism			
PA2971		conserved hypothetical protein	4,66	Hypothetical, unclassified, unknown			
PA2973		probable peptidase	3,28	Translation, post-translational modification, degradation			
PA2974		probable hydrolase	3,87	Putative enzymes			
PA2975	*rluC*	ribosomal large subunit pseudouridine synthase C	4,37	Transcription, RNA processing and degradation			
PA2976	*rne*	ribonuclease E	-2,79	Transcription, RNA processing and degradation			
PA2985		hypothetical protein	2,74	Membrane proteins			
PA2988	*lolE*	lipoprotein localization protein LolE	14,72	Membrane proteins			
PA2990		probable phosphodiesterase	-5,03	Central intermediary metabolism			
PA2992		hypothetical protein	-2,34	Hypothetical, unclassified, unknown			
PA2994	*nqrF*	Na+-translocating NADH:quinone oxidoreductase, subunit Nqr6	-3,31	Energy metabolism			
PA2995	*nqrE*	Na+-translocating NADH:quinone oxidoreductase subunit Nqr5	-3,56	Energy metabolism			
PA2996	*nqrD*	Na+-translocating NADH:uniquinone oxidoreductase subunit Nqr4	-3,16	Energy metabolism			
PA2997	*nqrC*	Na+-translocating NADH:ubiquinone oxidoreductase subunit Nrq3	-2,17	Energy metabolism			
PA3001		probable glyceraldehyde-3-phosphate dehydrogenase	-2,51	Putative enzymes			x
PA3006	*psrA*	transcriptional regulator PsrA	18,28	Transcriptional regulators			
PA3007	*lexA*	repressor protein LexA	7,46	Translation, post-translational modification, degradation	Adaptation, Protection		
PA3008	*sulA*	SulA	6,71	Hypothetical, unclassified, unknown			
PA3010		hypothetical protein	-4,85	Hypothetical, unclassified, unknown			
PA3011	*topA*	DNA topoisomerase I	-3,05	DNA replication, recombination, modification and repair			
PA3013	*faoB*	fatty-acid oxidation complex beta-subunit	-3,05	Fatty acid and phospholipid metabolism	Amino acid biosynthesis and metabolism		
PA3014	*faoA*	fatty-acid oxidation complex alpha-subunit	-2,02	Fatty acid and phospholipid metabolism	Amino acid biosynthesis and metabolism		
PA3015		hypothetical protein	2,52	Hypothetical, unclassified, unknown			
PA3016		hypothetical protein	4,94	Hypothetical, unclassified, unknown			
PA3018		hypothetical protein	2,84	Hypothetical, unclassified, unknown			
PA3019		probable ATP-binding component of ABC transporter	2,68	Transport of small molecules			
PA3020		probable soluble lytic transglycosylase	-4,88	Cell Wall / LPS / Capsule			
PA3021		hypothetical protein	-5,35	Hypothetical, unclassified, unknown			
PA3022		hypothetical protein	-2,81	Hypothetical, unclassified, unknown			
PA3028	*moeA2*	molybdenum cofactor biosynthesis protein A2	-3,83	Biosynthesis of cofactors, prosthetic groups and carrier			
PA3029	*moaB2*	molybdopterin biosynthetic protein B2	-3,94	Biosynthesis of cofactors, prosthetic groups and carrier			
PA3031		Lipoprotein	-4,13	Hypothetical, unclassified, unknown			
PA3031.1		tRNA-Pro	6,82	Non-coding RNA gene			
PA3032	*snr1*	cytochrome c Snr1	-3,68	Energy metabolism			
PA3037		hypothetical protein	-3,14	Hypothetical, unclassified, unknown			
PA3039		probable transporter	6,77	Membrane proteins	Transport of small molecules		
PA3040		conserved hypothetical protein	-7,14	Hypothetical, unclassified, unknown			
PA3041		hypothetical protein	-5,85	Membrane proteins			
PA3042		hypothetical protein	-6,29	Hypothetical, unclassified, unknown			
PA3043		conserved hypothetical protein	-3,09	Hypothetical, unclassified, unknown			
PA3044	*rocsS2*	Two-component sensor RocS2	8,51	Two-component regulatory systems			
PA3045	*rocA2*	Two-component response regulator, RocA2	10,23	Two-component regulatory systems	Transcriptional regulators		
PA3047		probable D-alanyl-D-alanine carboxypeptidase	-3,75	Cell Wall / LPS / Capsule			
PA3048		conserved hypothetical protein	4,30	Hypothetical, unclassified, unknown			
PA3049	*rmf*	ribosome modulation factor	-200,00	Translation, post-translational modification, degradation			
PA3051		hypothetical protein	3,65	Hypothetical, unclassified, unknown			
PA3052		hypothetical protein	-2,78	Hypothetical, unclassified, unknown			
PA3053		probable hydrolytic enzyme	-2,27	Putative enzymes			
PA3054		hypothetical protein	-6,54	Hypothetical, unclassified, unknown			
PA3058	*pelG*	PelG	4,08	Cell Wall / LPS / Capsule			
PA3059	*pelF*	PelF	2,35	Cell Wall / LPS / Capsule			
PA3060	*pelE*	PelE	2,72	Cell Wall / LPS / Capsule			
PA3061	*pelD*	PelD	2,67	Cell Wall / LPS / Capsule			
PA3062	*pelC*	PelC	2,63	Cell Wall / LPS / Capsule			
PA3063	*pelB*	PelB	6,14	Cell Wall / LPS / Capsule			
PA3064	*pelA*	PelA	6,55	Cell Wall / LPS / Capsule			
PA3068	*gdhB*	NAD-dependent glutamate dehydrogenase	-8,20	Amino acid biosynthesis and metabolism			
PA3069		hypothetical protein	10,53	Hypothetical, unclassified, unknown			
PA3075		hypothetical protein	-3,85	Hypothetical, unclassified, unknown			
PA3079		hypothetical protein	3,06	Membrane proteins			
PA3081		conserved hypothetical protein	-2,53	Hypothetical, unclassified, unknown			
PA3083	*pepN*	aminopeptidase N	-3,68	Translation, post-translational modification, degradation			
PA3086		hypothetical protein	-2,27	Membrane proteins			
PA3087		hypothetical protein	-2,65	Hypothetical, unclassified, unknown			
PA3090		hypothetical protein	5,88	Hypothetical, unclassified, unknown			
PA3091		hypothetical protein	-2,79	Hypothetical, unclassified, unknown			
PA3092	*fadH1*	2,4-dienoyl-CoA reductase FadH1	6,35	Fatty acid and phospholipid metabolism			
PA3094.1		tRNA-Asp	6,23	Non-coding RNA gene			
PA3094.2		tRNA-Asp	7,53	Non-coding RNA gene			
PA3095	*xcpZ*	general secretion pathway protein M	-3,27	Protein secretion/export apparatus			
PA3096	*xcpY*	general secretion pathway protein L	-3,13	Protein secretion/export apparatus			
PA3097	*xcpX*	general secretion pathway protein K	-2,00	Protein secretion/export apparatus			
PA3098	*xcpW*	general secretion pathway protein J	-5,35	Protein secretion/export apparatus			
PA3099	*xcpV*	general secretion pathway protein I	-9,17	Protein secretion/export apparatus			
PA3100	*xcpU*	General secretion pathway outer membrane protein H precursor	-15,87	Protein secretion/export apparatus			
PA3101	*xcpT*	general secretion pathway protein G	-17,24	Protein secretion/export apparatus			
PA3102	*xcpS*	general secretion pathway protein F	-3,57	Protein secretion/export apparatus			
PA3103	*xcpR*	general secretion pathway protein E	-3,77	Protein secretion/export apparatus			
PA3105	*xcpQ*	general secretion pathway protein D	-2,55	Protein secretion/export apparatus			
PA3107	*metZ*	o-succinylhomoserine sulfhydrylase	-2,04	Amino acid biosynthesis and metabolism			
PA3109		hypothetical protein	4,03	Adaptation, Protection			
PA3112	*accD*	acetyl-CoA carboxylase beta subunit	2,12	Fatty acid and phospholipid metabolism			
PA3117	*asd*	aspartate semialdehyde dehydrogenase	-2,01	Amino acid biosynthesis and metabolism			
PA3118	*leuB*	3-isopropylmalate dehydrogenase	-3,66	Amino acid biosynthesis and metabolism			
PA3119		conserved hypothetical protein	-2,09	Hypothetical, unclassified, unknown			
PA3120	*leuD*	3-isopropylmalate dehydratase small subunit	-2,46	Amino acid biosynthesis and metabolism			
PA3122		probable transcriptional regulator	-2,14	Transcriptional regulators			
PA3123		RidA subfamily protein	-4,39	Hypothetical, unclassified, unknown			
PA3126	*ibpA*	heat-shock protein IbpA	2,76	Chaperones & heat shock proteins			
PA3127		hypothetical protein	-2,58	Hypothetical, unclassified, unknown			
PA3128		probable short-chain dehydrogenase	-3,38	Putative enzymes			
PA3129		conserved hypothetical protein	5,75	Hypothetical, unclassified, unknown			
PA3132		probable hydrolase	2,30	Putative enzymes			
PA3133	*sawR*	SawR	2,55	Transcriptional regulators			
PA3133.1		tRNA-Glu	4,40	Non-coding RNA gene			
PA3133.4		tRNA-Ala	2,37	Non-coding RNA gene			
PA3136		probable secretion protein	2,69	Transport of small molecules			
PA3137		probable major facilitator superfamily (MFS) transporter	2,51	Membrane proteins	Transport of small molecules		
PA3139		probable amino acid aminotransferase	-2,48	Amino acid biosynthesis and metabolism	Putative enzymes		
PA3139.1		tRNA-Asn	-2,91	Non-coding RNA gene			
PA3140		hypothetical protein	6,01	Hypothetical, unclassified, unknown			
PA3141	*wbpM*	nucleotide sugar epimerase/dehydratase WbpM	2,72	Membrane proteins	Cell Wall / LPS / Capsule	Putative enzymes	x
PA3142		integrase	2,34	Relative to phage, transposon, or plasmid			x
PA3145	*wbpL*	glycosyltransferase WbpL	-2,00	Cell Wall / LPS / Capsule	Membrane proteins		x
PA3147	*wbpJ*	probable glycosyl transferase WbpJ	-2,11	Cell Wall / LPS / Capsule	Putative enzymes		x
PA3148	*wbpI*	UDP-N-acetylglucosamine 2-epimerase WbpI	-7,69	Cell Wall / LPS / Capsule	Putative enzymes		x
PA3149	*wbpH*	probable glycosyltransferase WbpH	-10,87	Cell Wall / LPS / Capsule	Putative enzymes		x
PA3150	*wbpG*	LPS biosynthesis protein WbpG	-12,50	Cell Wall / LPS / Capsule			x
PA3151	*hisF2*	imidazoleglycerol-phosphate synthase, cyclase subunit	-6,45	Amino acid biosynthesis and metabolism			x
PA3152	*hisH2*	glutamine amidotransferase	-4,31	Amino acid biosynthesis and metabolism			x
PA3153	*wzx*	O-antigen translocase	-6,25	Cell Wall / LPS / Capsule	Membrane proteins	Transport of small molecules	x
PA3154	*wzy*	B-band O-antigen polymerase	-7,63	Cell Wall / LPS / Capsule	Membrane proteins		x
PA3155	*wbpE*	UDP-2-acetamido-2-dideoxy-d-ribo-hex-3-uluronic acid transaminase, wbpE	-7,63	Cell Wall / LPS / Capsule	Putative enzymes		x
PA3156	*wbpD*	UDP-2-acetamido-3-amino-2,3-dideoxy-d-glucuronic acid N-acetyltransferase, WbpD	-4,44	Cell Wall / LPS / Capsule	Putative enzymes		x
PA3157	*wbpC*	probable acetyltransferase	-2,36	Cell Wall / LPS / Capsule	Putative enzymes		x
PA3159	*wbpA*	UDP-N-acetyl-d-glucosamine 6-Dehydrogenase	2,42	Cell Wall / LPS / Capsule	Putative enzymes		x
PA3160	*wzz*	O-antigen chain length regulator	7,71	Cell Wall / LPS / Capsule			x
PA3163	*cmk*	cytidylate kinase	-6,90	Nucleotide biosynthesis and metabolism			
PA3165	*hisC2*	histidinol-phosphate aminotransferase	-3,22	Amino acid biosynthesis and metabolism			
PA3166	*pheA*	chorismate mutase	-4,50	Amino acid biosynthesis and metabolism			
PA3171	*ubiG*	3-demethylubiquinone-9 3-methyltransferase	-2,35	Biosynthesis of cofactors, prosthetic groups and carrier	Energy metabolism		
PA3172		probable hydrolase	-4,31	Putative enzymes			
PA3173		probable short-chain dehydrogenase	-6,99	Putative enzymes			x
PA3176	*gltS*	glutamate/sodium ion symporter, GltS	2,71	Transport of small molecules			
PA3177		diguanylate cyclase	11,63	Hypothetical, unclassified, unknown			
PA3179		conserved hypothetical protein	7,07	Hypothetical, unclassified, unknown			
PA3181	*eda*	2-keto-3-deoxy-6-phosphogluconate aldolase	2,35	Carbon compound catabolism	Central intermediary metabolism		
PA3182	*pgl*	6-phosphogluconolactonase	2,51	Central intermediary metabolism			
PA3183	*zwf*	glucose-6-phosphate 1-dehydrogenase	4,08	Energy metabolism	Carbon compound catabolism		
PA3187		probable ATP-binding component of ABC transporter	3,38	Transport of small molecules			
PA3188		probable permease of ABC sugar transporter	4,47	Transport of small molecules			
PA3189		probable permease of ABC sugar transporter	5,10	Transport of small molecules			
PA3190		probable binding protein component of ABC sugar transporter	2,51	Transport of small molecules			
PA3191	*gtrS*	glucose transport sensor, GtrS	2,56	Two-component regulatory systems			x
PA3198		conserved hypothetical protein	-4,63	Hypothetical, unclassified, unknown			
PA3199		conserved hypothetical protein	-3,76	Hypothetical, unclassified, unknown			
PA3202		conserved hypothetical protein	-3,79	Hypothetical, unclassified, unknown			
PA3203		hypothetical protein	-2,88	Hypothetical, unclassified, unknown			
PA3205		hypothetical protein	6,74	Hypothetical, unclassified, unknown			
PA3206	*cpxS*	CpxS	3,24	Two-component regulatory systems			
PA3209		conserved hypothetical protein	7,09	Hypothetical, unclassified, unknown			
PA3210	*trkH*	potassium uptake protein TrkH	2,14	Membrane proteins	Transport of small molecules		
PA3213		hypothetical protein	-2,20	Hypothetical, unclassified, unknown			
PA3214		Lipoprotein	-5,15	Hypothetical, unclassified, unknown			
PA3215		probable transcriptional regulator	2,07	Transcriptional regulators			
PA3216		hypothetical protein	-2,42	Membrane proteins			
PA3218		hypothetical protein	15,99	Hypothetical, unclassified, unknown			
PA3219		hypothetical protein	15,57	Hypothetical, unclassified, unknown			
PA3221	*csaA*	CsaA protein	-2,49	Protein secretion/export apparatus	Chaperones & heat shock proteins		
PA3222		hypothetical protein	-4,90	Membrane proteins			
PA3223	*azoR3*	AzoR3, azoreductase 3	2,95	Fatty acid and phospholipid metabolism			
PA3224		hypothetical protein	-4,27	Hypothetical, unclassified, unknown			
PA3226		probable hydrolase	-2,48	Putative enzymes			
PA3227	*ppiA*	peptidyl-prolyl cis-trans isomerase A	-7,25	Translation, post-translational modification, degradation	Chaperones & heat shock proteins		
PA3228		probable ATP-binding/permease fusion ABC transporter	-5,05	Membrane proteins	Transport of small molecules		
PA3234		probable sodium:solute symporter	4,76	Membrane proteins	Transport of small molecules		
PA3235		conserved hypothetical protein	11,00	Membrane proteins			
PA3236	*betX*	BetX	4,70	Adaptation, Protection	Transport of small molecules		
PA3237		hypothetical protein	10,32	Hypothetical, unclassified, unknown			
PA3239		conserved hypothetical protein	-2,11	Hypothetical, unclassified, unknown			

PA3242	htrB2	temperature-regulated acyltransferase HtrB1	4,22	Cell Wall / LPS / Capsule			
PA3246	rluA	pseudouridine synthase RluA	2,18	Transcription, RNA processing and degradation			
PA3249		probable transcriptional regulator	-2,14	Transcriptional regulators			
PA3250		hypothetical protein	-5,99	Hypothetical, unclassified, unknown			
PA3251		hypothetical protein	-4,29	Hypothetical, unclassified, unknown			
PA3252		probable permease of ABC transporter	-3,82	Membrane proteins	Transport of small molecules		
PA3253		probable permease of ABC transporter	-2,56	Membrane proteins	Transport of small molecules		
PA3254		probable ATP-binding component of ABC transporter	-5,29	Transport of small molecules			
PA3255		hypothetical protein	-4,07	Hypothetical, unclassified, unknown			
PA3256		probable oxidoreductase	-2,25	Putative enzymes			
PA3258		hypothetical protein	2,30	Hypothetical, unclassified, unknown			
PA3260		probable transcriptional regulator	2,87	Transcriptional regulators			
PA3261		hypothetical protein	-2,30	Hypothetical, unclassified, unknown			
PA3262.1		tRNA-Asp	20,07	Non-coding RNA gene			
PA3262.2		tRNA-Val	3,30	Non-coding RNA gene			
PA3265		probable transporter	3,48	Membrane proteins	Transport of small molecules		
PA3266	capB	cold acclimation protein B	3,63	Adaptation, Protection	Transcriptional regulators		
PA3267		hypothetical protein	2,26	Membrane proteins			
PA3269		probable transcriptional regulator	4,58	Transcriptional regulators			
PA3270		hypothetical protein	2,18	Hypothetical, unclassified, unknown			
PA3271		probable two-component sensor	2,01	Two-component regulatory systems			
PA3273		hypothetical protein	3,18	Hypothetical, unclassified, unknown			
PA3276		hypothetical protein	2,78	Hypothetical, unclassified, unknown			
PA3279	oprP	Phosphate-specific outer membrane porin OprP precursor	5,07	Transport of small molecules			
PA3280	oprO	Pyrophosphate-specific outer membrane porin OprO precursor	10,50	Transport of small molecules			
PA3281		hypothetical protein	3,19	Membrane proteins			
PA3282		hypothetical protein	11,44	Hypothetical, unclassified, unknown			
PA3283		conserved hypothetical protein	8,09	Hypothetical, unclassified, unknown			
PA3284		hypothetical protein	5,78	Hypothetical, unclassified, unknown			
PA3291	tli1	Tli1	-2,64	Adaptation, Protection			
PA3294	vgrG4a	VgrG4a	-2,34	Hypothetical, unclassified, unknown			
PA3295		probable HIT family protein	-6,13	Putative enzymes			
PA3296	phoA	alkaline phosphatase	2,95	Central intermediary metabolism			
PA3298		hypothetical protein	2,41	Hypothetical, unclassified, unknown			
PA3299	fadD1	long-chain-fatty-acid--CoA ligase	-2,70	Fatty acid and phospholipid metabolism			
PA3302		conserved hypothetical protein	-7,30	Putative enzymes			
PA3304.1	P18	P18	3,21	Non-coding RNA gene			
PA3305		hypothetical protein	2,60	Membrane proteins			
PA3305.1	phrS	PhrS	-2,72	Non-coding RNA gene			
PA3306		hypothetical protein	9,01	Hypothetical, unclassified, unknown			
PA3307		hypothetical protein	4,15	Hypothetical, unclassified, unknown			
PA3309	uspK	Universal stress protein	-9,71	Hypothetical, unclassified, unknown			
PA3311	nbdA	NbdA	-4,88	Membrane proteins			
PA3313		hypothetical protein	-8,33	Transport of small molecules			
PA3314		probable ATP-binding component of ABC transporter	-3,51	Transport of small molecules			
PA3315		probable permease of ABC transporter	-2,35	Membrane proteins	Transport of small molecules		
PA3316		probable permease of ABC transporter	-2,42	Membrane proteins	Transport of small molecules		
PA3319	plcN	non-hemolytic phospholipase C precursor	8,68	Secreted Factors (toxins, enzymes, alginate)			x
PA3320		hypothetical protein	19,66	Membrane proteins			
PA3322		hypothetical protein	2,90	Hypothetical, unclassified, unknown			
PA3324		probable short-chain dehydrogenase	-2,26	Putative enzymes			
PA3326	clpP2	ClpP2	-5,78	Translation, post-translational modification, degradation	Cell Wall / LPS / Capsule		
PA3327		probable non-ribosomal peptide synthetase	-4,13	Adaptation, Protection			
PA3328		probable FAD-dependent monooxygenase	-3,48	Putative enzymes			
PA3329		hypothetical protein	-6,06	Hypothetical, unclassified, unknown			
PA3330		probable short chain dehydrogenase	-8,77	Putative enzymes			
PA3331		cytochrome P450	-9,52	Adaptation, Protection	Carbon compound catabolism		
PA3332		conserved hypothetical protein	-9,90	Hypothetical, unclassified, unknown			
PA3333	fabH2	3-oxoacyl-[acyl-carrier-protein] synthase III	-8,40	Fatty acid and phospholipid metabolism			
PA3334	acp3	Acp3	-6,17	Fatty acid and phospholipid metabolism			
PA3335		hypothetical protein	-4,42	Hypothetical, unclassified, unknown			
PA3336		probable major facilitator superfamily (MFS) transporter	-3,55	Membrane proteins	Transport of small molecules		
PA3338		hypothetical protein	2,28	Hypothetical, unclassified, unknown			
PA3339	plpD	patatin-like protein, PlpD	2,62	Membrane proteins	Secreted Factors (toxins, enzymes, alginate)		
PA3343	hsbD	diguanylate cyclase HsbD	2,01	Motility and Attachment			
PA3344	recQ	ATP-dependent DNA helicase RecQ	2,49	DNA replication, recombination, modification and repair			
PA3345	hptB	Histidine phosphotransfer protein HptB	3,81	Two-component regulatory systems	Motility and Attachment		
PA3346	hsbR	HptB-dependent secretion and biofilm regulator HsbR	2,34	Two-component regulatory systems	Transcriptional regulators		
PA3347	hsbA	HptB-dependent secretion and biofilm anti anti-sigma factor HsbA	2,29	Two-component regulatory systems	Motility and Attachment		
PA3348	cheR1	CheR1	3,66	Adaptation, Protection	Chemotaxis		
PA3349		probable chemotaxis protein	2,52	Adaptation, Protection	Chemotaxis		
PA3350		hypothetical protein	4,31	Hypothetical, unclassified, unknown			x
PA3351	flgM	FlgM	8,12	Transcriptional regulators			x
PA3352		hypothetical protein	8,77	Hypothetical, unclassified, unknown			x
PA3353	flgZ	FlgZ	10,43	Hypothetical, unclassified, unknown			
PA3354		hypothetical protein	-3,53	Hypothetical, unclassified, unknown			
PA3356	pauA5	Glutamylpolyamine synthetase	-2,12	Carbon compound catabolism			
PA3357	dsdA	D-serine dehydratase	2,14	Amino acid biosynthesis and metabolism			
PA3358		hypothetical protein	3,39	Membrane proteins			
PA3359		hypothetical protein	7,52	Membrane proteins			
PA3360		probable secretion protein	8,54	Secreted Factors (toxins, enzymes, alginate)			
PA3361	lecB	fucose-binding lectin PA-IIL	-10,53	Motility and Attachment			
PA3362		hypothetical protein	-2,20	Membrane proteins			
PA3363	amiR	aliphatic amidase regulator	-3,69	Transcriptional regulators			
PA3364	amiC	aliphatic amidase expression-regulating protein	-2,35	Transcriptional regulators			
PA3366.1	amiL	AmiL	24,51	Non-coding RNA gene			
PA3368		probable acetyltransferase	3,65	Putative enzymes			
PA3368.1		tRNA-Arg	28,52	Non-coding RNA gene			
PA3369		hypothetical protein	4,78	Membrane proteins			
PA3370		hypothetical protein	4,79	Membrane proteins			
PA3371		hypothetical protein	4,99	Hypothetical, unclassified, unknown			
PA3381		probable transcriptional regulator	3,85	Transcriptional regulators			
PA3382	phnE	phosphonate transport protein PhnE	2,40	Transport of small molecules	Membrane proteins		
PA3384	phnC	ATP-binding component of ABC phosphonate transporter	2,41	Transport of small molecules			
PA3386		conserved hypothetical protein	8,34	Hypothetical, unclassified, unknown			
PA3387	rhlG	beta-ketoacyl reductase	33,58	Fatty acid and phospholipid metabolism	Cell Wall / LPS / Capsule		
PA3388		conserved hypothetical protein	7,38	Hypothetical, unclassified, unknown			
PA3389		probable ring-cleaving dioxygenase	22,67	Putative enzymes			
PA3390		hypothetical protein	18,24	Hypothetical, unclassified, unknown			
PA3391	nosR	regulatory protein NosR	7,29	Membrane proteins	Energy metabolism		
PA3399		Lipoprotein	-2,74	Hypothetical, unclassified, unknown			
PA3402		hypothetical protein	2,14	Hypothetical, unclassified, unknown			
PA3403		hypothetical protein	15,68	Membrane proteins			
PA3404		probable outer membrane protein precursor	2,49	Protein secretion/export apparatus			
PA3407	hasAp	heme acquisition protein HasAp	-5,32	Transport of small molecules			
PA3411		hypothetical protein	7,14	Hypothetical, unclassified, unknown			
PA3412		hypothetical protein	2,04	Hypothetical, unclassified, unknown			
PA3413		conserved hypothetical protein	5,29	Hypothetical, unclassified, unknown			
PA3414		hypothetical protein	9,51	Hypothetical, unclassified, unknown			
PA3415		probable dihydrolipoamide acetyltransferase	-8,00	Energy metabolism			
PA3416		probable pyruvate dehydrogenase E1 component, beta chain	-6,10	Energy metabolism			
PA3417		probable pyruvate dehydrogenase E1 component, alpha subunit	-5,52	Energy metabolism			
PA3418	ldh	leucine dehydrogenase	-5,13	Amino acid biosynthesis and metabolism			
PA3420		probable transcriptional regulator	4,81	Transcriptional regulators			
PA3422		Lipoprotein	4,48	Hypothetical, unclassified, unknown			
PA3425		hypothetical protein	-3,55	Hypothetical, unclassified, unknown			
PA3426		probable enoyl CoA-hydratase/isomerase	-4,85	Putative enzymes			
PA3429		probable epoxide hydrolase	-2,15	Putative enzymes			
PA3432		hypothetical protein	2,22	Membrane proteins			
PA3434		probable transposase	2,06	Relative to phage, transposon, or plasmid			
PA3436		hypothetical protein	2,22	Hypothetical, unclassified, unknown			
PA3437	folM	dihydromonapterin reductase, FolM	-2,02	Biosynthesis of cofactors, prosthetic groups and carrier	Putative enzymes		
PA3438	folE1	GTP cyclohydrolase I precursor	-3,79	Biosynthesis of cofactors, prosthetic groups and carrier			
PA3439	folX	d-erythro-7,8-dihydroneopterin triphosphate epimerase	-3,56	Biosynthesis of cofactors, prosthetic groups and carrier			
PA3440		conserved hypothetical protein	-2,76	Hypothetical, unclassified, unknown			
PA3442		probable ATP-binding component of ABC transporter	3,33	Transport of small molecules			
PA3443		probable permease of ABC transporter	4,30	Membrane proteins	Transport of small molecules		
PA3444		conserved hypothetical protein	3,69	Putative enzymes			
PA3445		conserved hypothetical protein	5,10	Hypothetical, unclassified, unknown			
PA3446		conserved hypothetical protein	4,92	Hypothetical, unclassified, unknown			
PA3447		probable ATP-binding component of ABC transporter	6,45	Transport of small molecules			
PA3448		probable permease of ABC transporter	4,21	Membrane proteins	Transport of small molecules		
PA3449		conserved hypothetical protein	5,50	Hypothetical, unclassified, unknown			
PA3451		hypothetical protein	-9,17	Hypothetical, unclassified, unknown			
PA3455		conserved hypothetical protein	-4,81	Hypothetical, unclassified, unknown			
PA3460		probable acetyltransferase	-2,05	Putative enzymes			
PA3461		conserved hypothetical protein	-9,35	Hypothetical, unclassified, unknown			
PA3462		probable sensor/response regulator hybrid	-2,28	Two-component regulatory systems			
PA3464		hypothetical protein	6,27	Hypothetical, unclassified, unknown			
PA3465		conserved hypothetical protein	-3,50	Membrane proteins			
PA3467		probable major facilitator superfamily (MFS) transporter	3,98	Membrane proteins	Transport of small molecules		
PA3471		probable malic enzyme	-3,83	Central intermediary metabolism			
PA3473		hypothetical protein	3,31	Membrane proteins			
PA3474		conserved hypothetical protein	3,96	Membrane proteins			
PA3476	rhlI	autoinducer synthesis protein RhlI	-2,13	Adaptation, Protection			x
PA3477	rhlR	transcriptional regulator RhlR	-13,33	Transcriptional regulators			x
PA3478	rhlB	rhamnosyltransferase chain B	-22,22	Secreted Factors (toxins, enzymes, alginate)			x
PA3479	rhlA	rhamnosyltransferase chain A	-15,87	Secreted Factors (toxins, enzymes, alginate)			x
PA3480		probable deoxycytidine triphosphate deaminase	-2,18	Nucleotide biosynthesis and metabolism			
PA3481		conserved hypothetical protein	-2,25	Hypothetical, unclassified, unknown			
PA3482	metG	methionyl-tRNA synthetase	-3,41	Translation, post-translational modification, degradation	Amino acid biosynthesis and metabolism		
PA3485	tsi3	Tsi3	-3,24	Adaptation, Protection			
PA3486	vgrG4b	VgrG4b	-2,28	Hypothetical, unclassified, unknown			
PA3487	tle5	Tle5	-3,09	Fatty acid and phospholipid metabolism	Secreted Factors (toxins, enzymes, alginate)		x
PA3488	tli5	Tli5	-2,40	Adaptation, Protection			
PA3489		conserved hypothetical protein	3,29	Membrane proteins			
PA3493		conserved hypothetical protein	-2,98	Hypothetical, unclassified, unknown			
PA3494		conserved hypothetical protein	-3,00	Hypothetical, unclassified, unknown			
PA3495	nth	endonuclease III	-6,02	DNA replication, recombination, modification and repair			
PA3496		hypothetical protein	-2,01	Hypothetical, unclassified, unknown			
PA3497		hypothetical protein	8,48	Hypothetical, unclassified, unknown			
PA3498		probable oxidoreductase	4,30	Putative enzymes			x
PA3511		probable short-chain dehydrogenase	3,97	Putative enzymes			
PA3512		probable permease of ABC transporter	4,92	Membrane proteins	Transport of small molecules		
PA3513		hypothetical protein	10,02	Hypothetical, unclassified, unknown			
PA3514		probable ATP-binding component of ABC transporter	8,45	Transport of small molecules			
PA3517		probable lyase	3,21	Carbon compound catabolism			
PA3518		hypothetical protein	2,70	Hypothetical, unclassified, unknown			
PA3519		hypothetical protein	2,63	Hypothetical, unclassified, unknown			
PA3520		hypothetical protein	-5,35	Hypothetical, unclassified, unknown			
PA3521	opmE	OpmE	3,50	Membrane proteins	Transport of small molecules		
PA3522	mexQ	MexQ	5,53	Membrane proteins	Transport of small molecules		
PA3523	mexP	MexP	14,31	Transport of small molecules			
PA3525	argG	argininosuccinate synthase	3,12	Amino acid biosynthesis and metabolism			
PA3526	motY	MotY	12,42	Membrane proteins			x
PA3527	pyrC	dihydroorotase	-3,16	Nucleotide biosynthesis and metabolism			
PA3528	rnt	ribonuclease T	-7,25	DNA replication, recombination, modification and repair	Transcription, RNA processing and degradation		
PA3529		alkylhydroperoxide reductase C	-2,38	Adaptation, Protection	Putative enzymes		
PA3530	bfd	bacterioferritin-associated ferredoxin Bfd	-8,33	Hypothetical, unclassified, unknown			
PA3531	bfrB	bacterioferritin	10,19	Adaptation, Protection	Transport of small molecules		
PA3532		hypothetical protein	3,38	Membrane proteins			
PA3534		probable oxidoreductase	2,79	Putative enzymes			
PA3535		probable serine protease	-5,32	Putative enzymes			
PA3537	argF	ornithine carbamoyltransferase, anabolic	2,35	Amino acid biosynthesis and metabolism			
PA3539		conserved hypothetical protein	-2,76	Hypothetical, unclassified, unknown			
PA3540	algD	GDP-mannose 6-dehydrogenase AlgD	13,26	Secreted Factors (toxins, enzymes, alginate)	Cell Wall / LPS / Capsule	Adaptation, Protection	x
PA3541	alg8	alginate biosynthesis protein Alg8	20,92	Secreted Factors (toxins, enzymes, alginate)	Cell Wall / LPS / Capsule	Adaptation, Protection	x
PA3545	algG	alginate-c5-mannuronan-epimerase AlgG	2,31	Secreted Factors (toxins, enzymes, alginate)	Cell Wall / LPS / Capsule	Adaptation, Protection	x
PA3546	algX	alginate biosynthesis protein AlgX	2,79	Secreted Factors (toxins, enzymes, alginate)	Cell Wall / LPS / Capsule	Adaptation, Protection	x
PA3550	algF	alginate o-acetyltransferase AlgF	-2,83	Secreted Factors (toxins, enzymes, alginate)	Cell Wall / LPS / Capsule	Adaptation, Protection	x
PA3551	algA	phosphomannose isomerase / guanosine 5'-diphospho-D-mannose pyrophosphorylase	-2,48	Secreted Factors (toxins, enzymes, alginate)	Cell Wall / LPS / Capsule	Adaptation, Protection	x
PA3561	fruK	1-phosphofructokinase	2,19	Transport of small molecules	Central intermediary metabolism		
PA3562	fruI	phosphotransferase system transporter enzyme I, FruI	2,75	Transport of small molecules	Central intermediary metabolism		
PA3564		conserved hypothetical protein	-2,05	Hypothetical, unclassified, unknown			
PA3565		probable transcriptional regulator	2,86	Transcriptional regulators			
PA3566		conserved hypothetical protein	-2,53	Hypothetical, unclassified, unknown			
PA3567		probable oxidoreductase	-3,95	Putative enzymes			
PA3569	mmsB	3-hydroxyisobutyrate dehydrogenase	-5,03	Carbon compound catabolism			
PA3570	mmsA	methylmalonate-semialdehyde dehydrogenase	-3,89	Amino acid biosynthesis and metabolism	Carbon compound catabolism		
PA3572		hypothetical protein	-7,94	Hypothetical, unclassified, unknown			
PA3573		probable major facilitator superfamily (MFS) transporter	16,81	Adaptation, Protection	Transport of small molecules		
PA3577		hypothetical protein	3,21	Hypothetical, unclassified, unknown			
PA3580		conserved hypothetical protein	2,05	Hypothetical, unclassified, unknown			
PA3581	glpF	glycerol uptake facilitator protein	6,47	Transport of small molecules			
PA3582	glpK	glycerol kinase	-2,66	Central intermediary metabolism			
PA3583	glpR	glycerol-3-phosphate regulon repressor	-2,22	Transcriptional regulators			
PA3584	glpD	glycerol-3-phosphate dehydrogenase	9,56	Energy metabolism	Central intermediary metabolism		
PA3585	glpM	membrane protein GlpM	4,15	Membrane proteins	Transport of small molecules		
PA3586		probable hydrolase	6,48	Putative enzymes			
PA3588	opdR	probable porin	3,78	Carbon compound catabolism	Membrane proteins	Transport of small molecules	
PA3589		probable acyl-CoA thiolase	5,41	Carbon compound catabolism			
PA3590		probable hydroxyacyl-CoA dehydrogenase	3,26	Carbon compound catabolism			
PA3591		probable enoyl-CoA hydratase/isomerase	6,37	Carbon compound catabolism			
PA3592		conserved hypothetical protein	5,04	Hypothetical, unclassified, unknown			
PA3593		probable acyl-CoA dehydrogenase	5,41	Putative enzymes			
PA3595		probable major facilitator superfamily (MFS) transporter	4,00	Membrane proteins	Transport of small molecules		
PA3596		probable methylated-DNA--protein-cysteine methyltransferase	-2,72	DNA replication, recombination, modification and repair			
PA3597		probable amino acid permease	12,79	Membrane proteins	Transport of small molecules		
PA3598		conserved hypothetical protein	14,03	Hypothetical, unclassified, unknown			
PA3602		conserved hypothetical protein	-6,06	Membrane proteins			
PA3603	dgkA	diacylglycerol kinase	2,40	Fatty acid and phospholipid metabolism			
PA3606		conserved hypothetical protein	4,50	Hypothetical, unclassified, unknown			
PA3607	potA	polyamine transport protein PotA	14,04	Transport of small molecules			
PA3608	potB	polyamine transport protein PotB	9,79	Membrane proteins	Transport of small molecules		
PA3609	potC	polyamine transport protein PotC	2,57	Membrane proteins	Transport of small molecules		
PA3614		hypothetical protein	-11,36	Hypothetical, unclassified, unknown			
PA3615		hypothetical protein	-7,75	Hypothetical, unclassified, unknown			
PA3616		conserved hypothetical protein	9,25	Hypothetical, unclassified, unknown			
PA3617	recA	RecA protein	5,32	DNA replication, recombination, modification and repair			
PA3621	fdxA	ferredoxin I	2,26	Energy metabolism			
PA3621.1	rsmZ	regulatory RNA RsmZ	-2,57	Non-coding RNA gene			
PA3622	rpoS	sigma factor RpoS	-16,13	Transcriptional regulators			
PA3623		conserved hypothetical protein	-3,29	Hypothetical, unclassified, unknown			
PA3628		putative esterase	-4,27	Putative enzymes			
PA3629	adhC	alcohol dehydrogenase class III	-3,27	Central intermediary metabolism			
PA3630	gfnR	glutathione-dependent formaldehyde neutralization regulator GfnR	3,65	Transcriptional regulators			
PA3635	eno	enolase	-3,30	Translation, post-translational modification, degradation	Energy metabolism	Carbon compound catabolism	
PA3636	kdsA	2-dehydro-3-deoxyphosphooctonate aldolase	-5,81	Cell Wall / LPS / Capsule	Carbon compound catabolism		
PA3637	pyrG	CTP synthase	-2,61	Nucleotide biosynthesis and metabolism			
PA3638		conserved hypothetical protein	4,86	Hypothetical, unclassified, unknown			
PA3641		probable amino acid permease	2,46	Transport of small molecules			
PA3642	rnhB	ribonuclease HII	-2,38	DNA replication, recombination, modification and repair			
PA3644	lpxA	UDP-N-acetylglucosamine acyltransferase	-2,87	Cell Wall / LPS / Capsule			
PA3645	fabZ	(3R)-hydroxymyristoyl-[acyl carrier protein] dehydratase	-2,70	Cell Wall / LPS / Capsule	Fatty acid and phospholipid metabolism		
PA3646	lpxD	UDP-3-O-[3-hydroxylauroyl] glucosamine N-acyltransferase	-5,56	Cell Wall / LPS / Capsule			
PA3647		probable outer membrane protein precursor	-5,15	Membrane proteins	Transport of small molecules		
PA3649	mucP	MucP	2,77	Hypothetical, unclassified, unknown			x
PA3658	glnD	protein-PII uridylyltransferase	-2,21	Amino acid biosynthesis and metabolism			
PA3659		probable aminotransferase	-5,95	Putative enzymes			
PA3661		Lipoprotein	4,35	Hypothetical, unclassified, unknown			
PA3662		hypothetical protein	5,95	Hypothetical, unclassified, unknown			
PA3664		conserved hypothetical protein	3,61	Hypothetical, unclassified, unknown			
PA3666	dapD	tetrahydrodipicolinate succinylase	-2,99	Cell Wall / LPS / Capsule			
PA3667		probable pyridoxal-phosphate dependent enzyme	-2,99	Putative enzymes			
PA3668		conserved hypothetical protein	-2,66	Hypothetical, unclassified, unknown			
PA3670		hypothetical protein	-2,17	Hypothetical, unclassified, unknown			
PA3671		probable permease of ABC transporter	-2,53	Membrane proteins	Transport of small molecules		
PA3674		Lipoprotein	2,25	Hypothetical, unclassified, unknown			
PA3679		hypothetical protein	2,61	Hypothetical, unclassified, unknown			
PA3680		conserved hypothetical protein	2,13	Hypothetical, unclassified, unknown			
PA3684		hypothetical protein	2,01	Hypothetical, unclassified, unknown			
PA3685	yeaZ	YeaZ	3,11	Hypothetical, unclassified, unknown			
PA3686	adk	adenylate kinase	-3,38	Nucleotide biosynthesis and metabolism			
PA3687	ppc	phosphoenolpyruvate carboxylase	-2,05	Energy metabolism	Central intermediary metabolism		
PA3688		hypothetical protein	-7,19	Hypothetical, unclassified, unknown			
PA3692	lptF	Lipotoxin F, LptF	-2,18	Membrane proteins	Transport of small molecules		
PA3693		conserved hypothetical protein	-3,28	Hypothetical, unclassified, unknown			
PA3694		hypothetical protein	-3,66	Hypothetical, unclassified, unknown			
PA3695		Lipoprotein	-6,33	Hypothetical, unclassified, unknown			
PA3696		conserved hypothetical protein	-8,47	Hypothetical, unclassified, unknown			
PA3697		hypothetical protein	-3,19	Transcriptional regulators			
PA3699		probable transcriptional regulator	-2,10	Transcriptional regulators			
PA3702	wspR	WspR	-2,58	Motility and Attachment	Chemotaxis	Nucleotide biosynthesis and metabolism	
PA3703	wspF	probable methylesterase	-2,74	Motility and Attachment	Chemotaxis	Transcriptional regulators	
PA3704	wspE	probable chemotaxis sensor/effector fusion protein	-3,39	Two-component regulatory systems	Chemotaxis	Motility and Attachment	
PA3717		probable peptidyl-prolyl cis-trans isomerase, FkbP-type	2,85	Translation, post-translational modification, degradation	Chaperones & heat shock proteins		
PA3718		probable major facilitator superfamily (MFS) transporter	5,73	Membrane proteins	Transport of small molecules		
PA3719	armR	antirepressor for MexR, ArmR	6,20	Antibiotic resistance and susceptibility			
PA3720		hypothetical protein	7,51	Hypothetical, unclassified, unknown			
PA3722		hypothetical protein	3,55	Hypothetical, unclassified, unknown			
PA3723		probable FMN oxidoreductase	-12,82	Putative enzymes			
PA3724	lasB	elastase LasB	-40,00	Translation, post-translational modification, degradation	Secreted Factors (toxins, enzymes, alginate)		x
PA3728		hypothetical protein	2,43	Hypothetical, unclassified, unknown			
PA3729		conserved hypothetical protein	2,61	Hypothetical, unclassified, unknown			
PA3730		hypothetical protein	5,39	Membrane proteins			
PA3731		conserved hypothetical protein	3,76	Hypothetical, unclassified, unknown			
PA3732		Uncharacterized protein	4,32	Hypothetical, unclassified, unknown			
PA3733		hypothetical protein	-3,56	Hypothetical, unclassified, unknown			
PA3734		hypothetical protein	-7,87	Hypothetical, unclassified, unknown			
PA3735	thrC	threonine synthase	-3,21	Amino acid biosynthesis and metabolism	Biosynthesis of cofactors, prosthetic groups and carrier		x
PA3737	dsbC	thiol:disulfide interchange protein DsbC	-2,27	Translation, post-translational modification, degradation	Chaperones & heat shock proteins		

PA3739		probable sodium/hydrogen antiporter	-2,38	Membrane proteins	Transport of small molecules		
PA3741		hypothetical protein	27,01	Hypothetical, unclassified, unknown			
PA3742	*rplS*	50S ribosomal protein L19	8,20	Translation, post-translational modification, degradation			
PA3743	*trmD*	tRNA (guanine-N1)-methyltransferase	7,64	Transcription, RNA processing and degradation			
PA3744	*rimM*	16S rRNA processing protein	4,17	Transcription, RNA processing and degradation			
PA3745	*rpsP*	30S ribosomal protein S16	2,51	DNA replication, recombination, modification and repair	Translation, post-translational modification, degradation		
PA3748		conserved hypothetical protein	-3,33	Membrane proteins			
PA3749		probable major facilitator superfamily (MFS) transporter	4,94	Membrane proteins	Transport of small molecules		
PA3750		hypothetical protein	-2,08	Hypothetical, unclassified, unknown			
PA3751	*purT*	phosphoribosylglycinamide formyltransferase 2	-2,84	Amino acid biosynthesis and metabolism	Nucleotide biosynthesis and metabolism		
PA3752		hypothetical protein	-2,30	Hypothetical, unclassified, unknown			
PA3753		conserved hypothetical protein	-2,26	Transport of small molecules			
PA3758		probable N-acetylglucosamine-6-phosphate deacetylase	-2,99	Central intermediary metabolism			
PA3759		probable aminotransferase	-3,27	Putative enzymes			
PA3760		N-Acetyl-D-Glucosamine phosphotransferase system transporter	-2,10	Transport of small molecules			
PA3762		hypothetical protein	4,31	Hypothetical, unclassified, unknown			
PA3765		hypothetical protein	3,52	Hypothetical, unclassified, unknown			
PA3766		probable aromatic amino acid transporter	35,23	Transport of small molecules			
PA3767		conserved hypothetical protein	-2,35	Hypothetical, unclassified, unknown			
PA3769	*guaA*	GMP synthase	-2,19	Amino acid biosynthesis and metabolism	Nucleotide biosynthesis and metabolism		
PA3773		hypothetical protein	7,06	Membrane proteins			
PA3775		hypothetical protein	4,55	Membrane proteins			
PA3781		probable transporter	2,10	Membrane proteins	Transport of small molecules		
PA3784		hypothetical protein	-34,48	Hypothetical, unclassified, unknown			
PA3785		conserved hypothetical protein	-55,56	Hypothetical, unclassified, unknown			
PA3786		hypothetical protein	-2,28	Hypothetical, unclassified, unknown			
PA3789		hypothetical protein	-6,58	Membrane proteins			
PA3790	*oprC*	Putative copper transport outer membrane porin OprC precursor	-19,23	Transport of small molecules			
PA3792	*leuA*	2-isopropylmalate synthase	-7,46	Amino acid biosynthesis and metabolism			
PA3793		hypothetical protein	2,75	Hypothetical, unclassified, unknown			
PA3794		hypothetical protein	5,25	Membrane proteins			
PA3796		hypothetical protein	2,06	Hypothetical, unclassified, unknown			
PA3797		conserved hypothetical protein	-2,82	Hypothetical, unclassified, unknown			
PA3798		probable aminotransferase	-2,23	Putative enzymes			
PA3801		conserved hypothetical protein	-2,39	Hypothetical, unclassified, unknown			
PA3802	*hisS*	histidyl-tRNA synthetase	-3,38	Translation, post-translational modification, degradation			
PA3803	*gcpE*	probable isoprenoid biosynthetic protein GcpE	-3,18	Putative enzymes			
PA3808		conserved hypothetical protein	-2,02	Hypothetical, unclassified, unknown			
PA3815	*iscR*	IscR	5,49	Adaptation, Protection			
PA3816	*cysE*	O-acetylserine synthase	5,49	Biosynthesis of cofactors, prosthetic groups and carrier	Amino acid biosynthesis and metabolism		
PA3817		probable methyltransferase	14,17	Putative enzymes			
PA3818		extragenic suppressor protein SuhB	2,19	Translation, post-translational modification, degradation	Adaptation, Protection		
PA3819		conserved hypothetical protein	6,43	Membrane proteins			
PA3820	*secF*	secretion protein SecF	-3,27	Protein secretion/export apparatus			
PA3821	*secD*	secretion protein SecD	-3,52	Protein secretion/export apparatus	Membrane proteins		
PA3822	*yajC*	conserved hypothetical protein	-2,66	Hypothetical, unclassified, unknown			
PA3824	*queA*	S-adenosylmethionine:trna ribosyltransferase-isomerase	3,82	Translation, post-translational modification, degradation			
PA3824.1		tRNA-Leu	22,87	Non-coding RNA gene			
PA3826		hypothetical protein	4,03	Membrane proteins			x
PA3828	*lptF*	Lipopolysaccharide export system permease protein LptF	5,47	Membrane proteins			
PA3829		hypothetical protein	2,98	Hypothetical, unclassified, unknown			
PA3830		probable transcriptional regulator	2,19	Transcriptional regulators			
PA3831	*pepA*	leucine aminopeptidase	-2,27	Translation, post-translational modification, degradation	Transcription, RNA processing and degradation	Secreted Factors (toxins, enzymes, alginate)	x
PA3832	*holC*	DNA polymerase III, chi subunit	-2,67	DNA replication, recombination, modification and repair			
PA3833		hypothetical protein	-3,45	Hypothetical, unclassified, unknown			
PA3834	*valS*	valyl-tRNA synthetase	-3,38	Translation, post-translational modification, degradation	Amino acid biosynthesis and metabolism		
PA3835		hypothetical protein	2,19	Hypothetical, unclassified, unknown			
PA3836		hypothetical protein	-2,52	Hypothetical, unclassified, unknown			
PA3838		probable ATP-binding component of ABC transporter	-2,68	Transport of small molecules			
PA3842	*spcS*	specific Pseudomonas chaperone for ExoS, SpcS	-4,29	Secreted Factors (toxins, enzymes, alginate)	Protein secretion/export apparatus	Chaperones & heat shock proteins	
PA3843		hypothetical protein	-2,82	Hypothetical, unclassified, unknown			
PA3846		hypothetical protein	-2,32	Hypothetical, unclassified, unknown			
PA3847		conserved hypothetical protein	-2,34	Hypothetical, unclassified, unknown			
PA3848		hypothetical protein	-8,77	Hypothetical, unclassified, unknown			
PA3851		hypothetical protein	-2,03	Hypothetical, unclassified, unknown			
PA3855		hypothetical protein	-2,02	Hypothetical, unclassified, unknown			
PA3856		hypothetical protein	-4,24	Hypothetical, unclassified, unknown			
PA3857	*pcs*	phosphatidylcholine synthase	-2,64	Fatty acid and phospholipid metabolism			
PA3858		probable amino acid-binding protein	-6,41	Transport of small molecules			
PA3859		carboxylesterase	-3,09	Putative enzymes			
PA3860		probable AMP-binding enzyme	2,03	Putative enzymes			
PA3861	*rhl*	ATP-dependent RNA helicase RhlB	-7,52	Transcription, RNA processing and degradation			
PA3863	*dauA*	FAD-dependent catabolic D-arginine dehydrogenase, DauA	-2,84	Amino acid biosynthesis and metabolism			
PA3866		Pyocin S4	-3,21	Adaptation, Protection	Secreted Factors (toxins, enzymes, alginate)		
PA3868		hypothetical protein	3,15	Hypothetical, unclassified, unknown			
PA3869		hypothetical protein	6,33	Hypothetical, unclassified, unknown			
PA3870	*moaA1*	molybdopterin biosynthetic protein A1	2,31	Biosynthesis of cofactors, prosthetic groups and carrier			
PA3875	*narG*	respiratory nitrate reductase alpha chain	-2,78	Energy metabolism			
PA3877	*narK1*	nitrite extrusion protein 1	5,03	Membrane proteins	Transport of small molecules		
PA3882		hypothetical protein	-2,60	Hypothetical, unclassified, unknown			
PA3886		hypothetical protein	-2,39	Hypothetical, unclassified, unknown			
PA3888	*opuCD*	OpuC ABC transporter, permease protein, OpuCD	2,82	Transport of small molecules	Membrane proteins		
PA3891	*opuCA*	OpuC ABC transporter, ATP-binding protein, OpuCA	-2,68	Transport of small molecules			
PA3893		conserved hypothetical protein	2,37	Membrane proteins			
PA3894		probable outer membrane protein precursor	3,47	Membrane proteins			
PA3896		probable 2-hydroxyacid dehydrogenase	-2,12	Putative enzymes			
PA3897		hypothetical protein	54,53	Membrane proteins			
PA3899	*fecI*	FecI	-2,25	Transcriptional regulators			
PA3900	*fecR*	FecR	-3,85	Transcriptional regulators	Membrane proteins		
PA3901	*fecA*	Fe(III) dicitrate transport protein FecA	-2,60	Transport of small molecules	Membrane proteins		
PA3902		hypothetical protein	7,27	Hypothetical, unclassified, unknown			
PA3903	*prfC*	peptide chain release factor 3	-2,08	Translation, post-translational modification, degradation			
PA3910	*eddA*	Extracelullar DNA degradation protein, EddA	3,91	Secreted Factors (toxins, enzymes, alginate)	Nucleotide biosynthesis and metabolism		
PA3911	*ubiT*	conserved hypothetical protein	-4,42	Hypothetical, unclassified, unknown			
PA3912	*ubiV*	conserved hypothetical protein	-4,26	Hypothetical, unclassified, unknown			
PA3913	*UbiU*	probable protease	-3,05	Putative enzymes			
PA3917	*moaD*	molybdopterin converting factor, small subunit	-2,17	Biosynthesis of cofactors, prosthetic groups and carrier			
PA3919		conserved hypothetical protein	-15,38	Hypothetical, unclassified, unknown			
PA3920	*copA1*	CopA1	-3,26	Membrane proteins	Transport of small molecules		
PA3921		probable transcriptional regulator	-6,45	Transcriptional regulators			
PA3922		conserved hypothetical protein	-5,29	Hypothetical, unclassified, unknown			
PA3923		hypothetical protein	-4,42	Hypothetical, unclassified, unknown			
PA3924		probable medium-chain acyl-CoA ligase	-4,48	Fatty acid and phospholipid metabolism			
PA3925		probable acyl-CoA thiolase	2,27	Putative enzymes			
PA3926		probable major facilitator superfamily (MFS) transporter	2,07	Membrane proteins	Transport of small molecules		
PA3927		probable transcriptional regulator	6,45	Transcriptional regulators			
PA3928		hypothetical protein	-2,70	Hypothetical, unclassified, unknown			
PA3929	*cioB*	cyanide insensitive terminal oxidase	-2,20	Energy metabolism			
PA3930	*cioA*	cyanide insensitive terminal oxidase	-2,43	Energy metabolism			
PA3931		conserved hypothetical protein	2,42	Hypothetical, unclassified, unknown			
PA3933	*betT3*	BetT3	-5,88	Membrane proteins	Transport of small molecules		
PA3934		conserved hypothetical protein	-4,44	Membrane proteins			
PA3936		probable permease of ABC taurine transporter	4,49	Transport of small molecules	Membrane proteins		
PA3937		probable ATP-binding component of ABC taurine transporter	5,05	Transport of small molecules			
PA3938		probable periplasmic taurine-binding protein precursor	6,09	Transport of small molecules			
PA3939		hypothetical protein	4,25	Hypothetical, unclassified, unknown			
PA3946	*rocS1*	two-component sensor RocS1	4,20	Two-component regulatory systems			
PA3947	*rocR*	RocR	4,46	Nucleotide biosynthesis and metabolism			
PA3948	*rocA1*	Two-component response regulator RocA1	-2,74	Two-component regulatory systems	Transcriptional regulators		
PA3951		conserved hypothetical protein	7,58	Hypothetical, unclassified, unknown			
PA3952		hypothetical protein	6,51	Hypothetical, unclassified, unknown			
PA3956		hypothetical protein	-3,72	Hypothetical, unclassified, unknown			
PA3959		hypothetical protein	2,39	Hypothetical, unclassified, unknown			
PA3960		hypothetical protein	2,56	Hypothetical, unclassified, unknown			
PA3962		Lipoprotein	2,47	Hypothetical, unclassified, unknown			
PA3963		probable transporter	2,48	Membrane proteins	Transport of small molecules		
PA3965		probable transcriptional regulator	-2,50	Transcriptional regulators			
PA3966		hypothetical protein	3,03	Membrane proteins			
PA3967		hypothetical protein	4,43	Hypothetical, unclassified, unknown			
PA3968		probable pseudouridine synthase	3,22	Putative enzymes			
PA3970	*amn*	AMP nucleosidase	-2,39	Nucleotide biosynthesis and metabolism			
PA3976	*thiE*	thiamin-phosphate pyrophosphorylase	-2,34	Biosynthesis of cofactors, prosthetic groups and carrier			
PA3977	*hemL*	glutamate-1-semialdehyde 2,1-aminomutase	-6,76	Biosynthesis of cofactors, prosthetic groups and carrier			
PA3979		hypothetical protein	11,14	Hypothetical, unclassified, unknown			
PA3980		conserved hypothetical protein	2,20	Hypothetical, unclassified, unknown			
PA3981		conserved hypothetical protein	-3,95	Hypothetical, unclassified, unknown			
PA3982		conserved hypothetical protein	-4,81	Hypothetical, unclassified, unknown			
PA3983		conserved hypothetical protein	-6,80	Hypothetical, unclassified, unknown			
PA3984	*lnt*	apolipoprotein N-acyltransferase	-2,25	Translation, post-translational modification, degradation	Cell Wall / LPS / Capsule		
PA3986		hypothetical protein	-2,34	Hypothetical, unclassified, unknown			
PA3988	*lptE*	LPS-assembly lipoprotein LptE	-2,14	Hypothetical, unclassified, unknown			
PA3989	*holA*	DNA polymerase III, delta subunit	-4,12	DNA replication, recombination, modification and repair			
PA3990		conserved hypothetical protein	4,66	Hypothetical, unclassified, unknown			
PA3991		hypothetical protein	26,40	Hypothetical, unclassified, unknown			
PA3993		probable transposase	2,10	Relative to phage, transposon, or plasmid			
PA3994		probable epoxide hydrolase	12,02	Putative enzymes			
PA3997	*lipB*	lipoate-protein ligase B	-2,69	Amino acid biosynthesis and metabolism	Biosynthesis of cofactors, prosthetic groups and carrier		
PA3998		conserved hypothetical protein	-4,37	Hypothetical, unclassified, unknown			
PA3999	*dacC*	D-ala-D-ala-carboxypeptidase	-3,00	Cell Wall / LPS / Capsule			
PA4001	*sltB1*	soluble lytic transglycosylase B	2,25	Cell Wall / LPS / Capsule			
PA4003	*pbpA*	penicillin-binding protein 2	2,98	Cell Wall / LPS / Capsule			
PA4008		probable hydrolase	12,76	Membrane proteins	Putative enzymes		
PA4009		hypothetical protein	7,85	Hypothetical, unclassified, unknown			
PA4013		conserved hypothetical protein	3,03	Membrane proteins			
PA4014		hypothetical protein	3,03	Membrane proteins			
PA4015		conserved hypothetical protein	-14,93	Hypothetical, unclassified, unknown			
PA4022	*hdhA*	hydrazone dehydrogenase, HdhA	-2,27	Putative enzymes			
PA4023	*eat*	ethanolamine transporter, Eat	2,10	Transport of small molecules			
PA4024	*eutB*	ethanolamine-ammonia lyase, large subunit, EutB	-5,62	Central intermediary metabolism			
PA4025	*eutC*	ethanolamine-ammonia lyase, small subunit, EutC	-5,41	Carbon compound catabolism			
PA4026		probable acetyltransferase	-5,29	Putative enzymes			
PA4027		hypothetical protein	-4,27	Hypothetical, unclassified, unknown			
PA4031	*ppa*	inorganic pyrophosphatase	-2,32	Central intermediary metabolism			
PA4033	*mucE*	MucE	2,10	Hypothetical, unclassified, unknown			x
PA4035		hypothetical protein	-3,02	Hypothetical, unclassified, unknown			
PA4036		probable two-component sensor	-3,08	Two-component regulatory systems			
PA4039		hypothetical protein	2,63	Hypothetical, unclassified, unknown			
PA4041		hypothetical protein	2,66	Putative enzymes			
PA4045		conserved hypothetical protein	4,25	Hypothetical, unclassified, unknown			
PA4046		hypothetical protein	3,63	Membrane proteins			
PA4047	*ribA*	GTP cyclohydrolase II	2,73	Biosynthesis of cofactors, prosthetic groups and carrier			
PA4054	*ribB*	GTP cyclohydrolase II / 3,4-dihydroxy-2-butanone 4-phosphate synthase	-2,71	Biosynthesis of cofactors, prosthetic groups and carrier			
PA4059		hypothetical protein	-2,19	Hypothetical, unclassified, unknown			
PA4061		probable thioredoxin	-2,85	Energy metabolism			
PA4063		hypothetical protein	-4,22	Hypothetical, unclassified, unknown			
PA4064		probable ATP-binding component of ABC transporter	-5,56	Transport of small molecules			
PA4065		Lipoprotein	-4,03	Membrane proteins			
PA4066		hypothetical protein	-2,24	Hypothetical, unclassified, unknown			
PA4067	*oprG*	Outer membrane protein OprG precursor	-15,87	Membrane proteins			
PA4068		probable epimerase	-2,03	Central intermediary metabolism			
PA4071		hypothetical protein	3,23	Hypothetical, unclassified, unknown			
PA4072		probable amino acid permease	7,23	Transport of small molecules			
PA4073		probable aldehyde dehydrogenase	3,31	Putative enzymes			
PA4076		hypothetical protein	-2,51	Hypothetical, unclassified, unknown			
PA4077		probable transcriptional regulator	-3,30	Transcriptional regulators			
PA4078		probable nonribosomal peptide synthetase	-19,23	Adaptation, Protection			
PA4079		NAD(P)H-dependent carbonyl reductase	-2,75	Putative enzymes			
PA4081	*cupB6*	fimbrial subunit CupB6	2,81	Motility and Attachment			
PA4083	*cupB4*	chaperone CupB4	9,00	Motility and Attachment	Chaperones & heat shock proteins		
PA4084	*cupB3*	usher CupB3	5,86	Motility and Attachment			
PA4085	*cupB2*	chaperone CupB2	3,47	Motility and Attachment	Chaperones & heat shock proteins		
PA4087		conserved hypothetical protein	11,22	Hypothetical, unclassified, unknown			
PA4088		glutamate-1-semialdehyde aminomutase	2,72	Putative enzymes			
PA4090		hypothetical protein	-8,47	Hypothetical, unclassified, unknown			
PA4094		probable transcriptional regulator	2,64	Transcriptional regulators			
PA4095		hypothetical protein	19,88	Hypothetical, unclassified, unknown			
PA4096		probable major facilitator superfamily (MFS) transporter	17,36	Membrane proteins	Transport of small molecules		
PA4098		probable short-chain dehydrogenase	4,63	Putative enzymes			
PA4099		hypothetical protein	6,16	Hypothetical, unclassified, unknown			
PA4101	*bfmR*	BfmR	-4,65	Two-component regulatory systems	Transcriptional regulators		
PA4102	*bfmS*	BfmS	-5,21	Two-component regulatory systems	Cell Wall / LPS / Capsule	Adaptation, Protection	
PA4105		hypothetical protein	2,70	Hypothetical, unclassified, unknown			
PA4106		conserved hypothetical protein	4,93	Hypothetical, unclassified, unknown			
PA4107	*efhP*	EfhP	10,83	Hypothetical, unclassified, unknown			
PA4108		cyclic di-GMP phosphodiesterase	-3,17	Cell Wall / LPS / Capsule	Motility and Attachment		x
PA4110	*ampC*	beta-lactamase precursor	-3,38	Adaptation, Protection			
PA4113		probable major facilitator superfamily (MFS) transporter	5,33	Membrane proteins	Transport of small molecules		
PA4117	*bphP*	bacterial phytochrome, BphP	-5,03	Two-component regulatory systems			
PA4119	*aph*	aminoglycoside 3'-phosphotransferase type IIb	3,30	Antibiotic resistance and susceptibility			
PA4120		probable transcriptional regulator	2,32	Transcriptional regulators			
PA4125	*hpcD*	5-carboxymethyl-2-hydroxymuconate isomerase	-3,33	Carbon compound catabolism			
PA4129		hypothetical protein	-5,56	Adaptation, Protection			
PA4130		probable sulfite or nitrite reductase	-3,12	Adaptation, Protection	Central intermediary metabolism		
PA4131		probable iron-sulfur protein	-3,37	Adaptation, Protection	Putative enzymes		
PA4132		conserved hypothetical protein	-5,78	Adaptation, Protection			
PA4133		cytochrome c oxidase subunit (cbb3-type)	-6,06	Adaptation, Protection	Energy metabolism		
PA4134		hypothetical protein	-7,81	Adaptation, Protection			
PA4135		probable transcriptional regulator	-3,60	Transcriptional regulators			
PA4136		probable major facilitator superfamily (MFS) transporter	4,06	Transport of small molecules	Membrane proteins		
PA4137	*opdL*	probable porin	2,42	Transport of small molecules			
PA4138	*tyrS*	tyrosyl-tRNA synthetase	5,46	Translation, post-translational modification, degradation	Amino acid biosynthesis and metabolism		
PA4139		hypothetical protein	-2,69	Hypothetical, unclassified, unknown			
PA4141		hypothetical protein	-12,66	Hypothetical, unclassified, unknown			
PA4142		probable secretion protein	-2,60	Protein secretion/export apparatus			
PA4143		probable toxin transporter	-2,03	Protein secretion/export apparatus	Membrane proteins	Transport of small molecules	
PA4144		probable outer membrane protein precursor	-3,88	Protein secretion/export apparatus			
PA4146		hypothetical protein	3,39	Hypothetical, unclassified, unknown			
PA4147	*acoR*	transcriptional regulator AcoR	2,56	Transcriptional regulators			
PA4148		probable short-chain dehydrogenase	8,53	Putative enzymes			
PA4149		conserved hypothetical protein	12,36	Hypothetical, unclassified, unknown			
PA4150		probable dehydrogenase E1 component	4,59	Carbon compound catabolism			x
PA4151	*acoB*	acetoin catabolism protein AcoB	7,44	Carbon compound catabolism			
PA4152		probable hydrolase	3,84	Carbon compound catabolism			
PA4154		conserved hypothetical protein	7,98	Hypothetical, unclassified, unknown			
PA4156	*fvbA*	FvbA	3,89	Transport of small molecules			
PA4158	*fepC*	ferric enterobactin transport protein FepC	2,81	Transport of small molecules			
PA4159	*fepB*	ferrienterobactin-binding periplasmic protein precursor FepB	5,95	Transport of small molecules			
PA4160	*fepD*	ferric enterobactin transport protein FepD	3,60	Transport of small molecules	Membrane proteins		
PA4161	*fepG*	ferric enterobactin transport protein FepG	2,19	Transport of small molecules	Membrane proteins		
PA4167		probable oxidoreductase	8,50	Putative enzymes			
PA4168	*fpvB*	second ferric pyoverdine receptor FpvB	-4,07	Transport of small molecules			
PA4169		conserved hypothetical protein	2,02	Hypothetical, unclassified, unknown			
PA4173		conserved hypothetical protein	10,55	Hypothetical, unclassified, unknown			
PA4175	*piv*	protease IV	-29,41	Secreted Factors (toxins, enzymes, alginate)			x
PA4176	*ppiC2*	peptidyl-prolyl cis-trans isomerase C2	-3,65	Translation, post-translational modification, degradation	Chaperones & heat shock proteins		
PA4179	*opdN*	probable porin	14,65	Membrane proteins	Transport of small molecules		
PA4180		probable acetolactate synthase large subunit	-2,84	Amino acid biosynthesis and metabolism			
PA4185		probable transcriptional regulator	-2,43	Transcriptional regulators			
PA4187		probable major facilitator superfamily (MFS) transporter	7,52	Membrane proteins	Transport of small molecules		
PA4188		conserved hypothetical protein	3,09	Hypothetical, unclassified, unknown			
PA4189		probable aldehyde dehydrogenase	2,53	Putative enzymes			
PA4190	*pqsL*	probable FAD-dependent monooxygenase	-9,52	Putative enzymes			
PA4192		probable ATP-binding component of ABC transporter	2,64	Transport of small molecules			
PA4193		probable permease of ABC transporter	8,30	Transport of small molecules	Membrane proteins		
PA4194		probable permease of ABC transporter	5,26	Transport of small molecules	Membrane proteins		
PA4195		probable binding protein component of ABC transporter	4,25	Transport of small molecules			
PA4198		probable AMP-binding enzyme	2,90	Putative enzymes			
PA4199		probable acyl-CoA dehydrogenase	-3,21	Putative enzymes			
PA4200		hypothetical protein	-6,25	Hypothetical, unclassified, unknown			
PA4201	*ddlA*	D-alanine-D-alanine ligase A	-3,05	Cell Wall / LPS / Capsule			
PA4204	*ppgL*	periplasmic gluconolactonase, PpgL	6,20	Hypothetical, unclassified, unknown			
PA4206	*mexH*	probable Resistance-Nodulation-Cell Division (RND) efflux membrane fusion protein pr	-2,17	Transport of small molecules			
PA4207	*mexI*	probable Resistance-Nodulation-Cell Division (RND) efflux transporter	-4,37	Transport of small molecules	Membrane proteins		
PA4208	*opmD*	probable outer membrane protein precursor	-22,73	Transport of small molecules	Membrane proteins		
PA4209	*phzM*	probable phenazine-specific methyltransferase	-2,39	Putative enzymes			x
PA4210	*phzA1*	probable phenazine biosynthesis protein	-9,52	Secreted Factors (toxins, enzymes, alginate)			x
PA4211	*phzB1*	probable phenazine biosynthesis protein	-6,41	Secreted Factors (toxins, enzymes, alginate)			x
PA4212	*phzC1*	phenazine biosynthesis protein PhzC	-13,16	Secreted Factors (toxins, enzymes, alginate)			x
PA4213	*phzD1*	phenazine biosynthesis protein PhzD	-19,61	Secreted Factors (toxins, enzymes, alginate)			x
PA4214	*phzE1*	phenazine biosynthesis protein PhzE	-19,61	Secreted Factors (toxins, enzymes, alginate)			x
PA4215	*phzF1*	probable phenazine biosynthesis protein	-21,74	Secreted Factors (toxins, enzymes, alginate)			x
PA4216	*phzG1*	probable pyridoxamine 5'-phosphate oxidase	-23,26	Secreted Factors (toxins, enzymes, alginate)			x
PA4217	*phzS*	flavin-containing monooxygenase	-6,67	Putative enzymes			x
PA4218	*ampP*	AmpP	-4,93	Membrane proteins	Antibiotic resistance and susceptibility	Transport of small molecules	
PA4219	*ampO*	AmpO	-4,72	Membrane proteins	Antibiotic resistance and susceptibility		
PA4220	*fptB*	hypothetical protein	-6,99	Hypothetical, unclassified, unknown			
PA4221	*fptA*	Fe(III)-pyochelin outer membrane receptor precursor	-6,49	Transport of small molecules			x
PA4223	*pchH*	probable ATP-binding component of ABC transporter	-2,43	Transport of small molecules	Membrane proteins		x
PA4224	*pchG*	pyochelin biosynthetic protein PchG	-3,28	Transport of small molecules	Membrane proteins		x
PA4225	*pchF*	pyochelin synthetase	-4,07	Transport of small molecules	Secreted Factors (toxins, enzymes, alginate)		x
PA4226	*pchE*	dihydroaeruginoic acid synthetase	-5,71	Transport of small molecules	Secreted Factors (toxins, enzymes, alginate)		x
PA4227	*pchR*	transcriptional regulator PchR	-4,18	Transcriptional regulators			x
PA4228	*pchD*	pyochelin biosynthesis protein PchD	-10,20	Transport of small molecules	Secreted Factors (toxins, enzymes, alginate)		x
PA4229	*pchC*	pyochelin biosynthetic protein PchC	-11,76	Transport of small molecules	Secreted Factors (toxins, enzymes, alginate)		x
PA4230	*pchB*	salicylate biosynthesis protein PchB	-12,50	Transport of small molecules	Secreted Factors (toxins, enzymes, alginate)		x
PA4231	*pchA*	salicylate biosynthesis isochorismate synthase	-10,53	Transport of small molecules	Secreted Factors (toxins, enzymes, alginate)		x
PA4232	*ssb*	single-stranded DNA-binding protein	-2,00	DNA replication, recombination, modification and repair			
PA4234	*uvrA*	excinuclease ABC subunit A	-2,47	DNA replication, recombination, modification and repair			
PA4235	*ftnA*	bacterial ferritin	-6,94	Adaptation, Protection	Transport of small molecules		
PA4236	*katA*	catalase	-5,62	Adaptation, Protection			
PA4270.1	*P26*	P26	3,22	Non-coding RNA gene			

PA4271	*rplL*	50S ribosomal protein L7 / L12	4,27	Translation, post-translational modification, degradation			
PA4272	*rplJ*	50S ribosomal protein L10	4,98	Translation, post-translational modification, degradation			
PA4273	*rplA*	50S ribosomal protein L1	2,16	Translation, post-translational modification, degradation			
PA4278		hypothetical protein	3,46	Hypothetical, unclassified, unknown			
PA4279		hypothetical protein	2,55	Hypothetical, unclassified, unknown			
PA4280	*birA*	BirA bifunctional protein	3,26	Biosynthesis of cofactors, prosthetic groups and carrier			
PA4280.3		tRNA-Ala	8,10	Non-coding RNA gene			
PA4280.4		tRNA-Ile	11,06	Non-coding RNA gene			
PA4283	*recD*	exodeoxyribonuclease V alpha chain	-2,63	DNA replication, recombination, modification and repair			
PA4284	*recB*	exodeoxyribonuclease V beta chain	-2,39	DNA replication, recombination, modification and repair			
PA4287		hypothetical protein	11,55	Membrane proteins			
PA4290		probable chemotaxis transducer	11,15	Adaptation, Protection	Chemotaxis		
PA4293	*pprA*	two-component sensor PprA	-5,03	Two-component regulatory systems			
PA4294		hypothetical protein	-8,33	Hypothetical, unclassified, unknown			
PA4295	*fppA*	Flp prepilin peptidase A, FppA	-2,13	Motility and Attachment			x
PA4296	*pprB*	two-component response regulator, PprB	-7,81	Two-component regulatory systems	Transcriptional regulators		
PA4297	*tadG*	TadG	-3,13	Motility and Attachment	Membrane proteins		
PA4298		hypothetical protein	-12,05	Hypothetical, unclassified, unknown			
PA4299	*tadD*	TadD	-18,52	Motility and Attachment			
PA4300	*tadC*	TadC	-6,41	Motility and Attachment	Membrane proteins		
PA4301	*tadB*	TadB	-5,03	Motility and Attachment	Membrane proteins		
PA4302	*tadA*	TadA ATPase	-5,00	Motility and Attachment	Protein secretion/export apparatus		
PA4303	*tadZ*	TadZ	-3,58	Motility and Attachment			
PA4304	*rcpA*	RcpA	-2,17	Motility and Attachment	Protein secretion/export apparatus		
PA4305	*rcpC*	RcpC	-2,20	Motility and Attachment			
PA4306	*flp*	Type IVb pilin, Flp	-62,50	Motility and Attachment			
PA4315	*mvaT*	transcriptional regulator MvaT, P16 subunit	-2,39	Transcriptional regulators			
PA4322		conserved hypothetical protein	-6,17	Hypothetical, unclassified, unknown			
PA4323		hypothetical protein	-4,39	Hypothetical, unclassified, unknown			
PA4324		hypothetical protein	2,55	Hypothetical, unclassified, unknown			
PA4325		hypothetical protein	4,34	Hypothetical, unclassified, unknown			
PA4326		Lipoprotein	4,71	Hypothetical, unclassified, unknown			
PA4328	*uspM*	Universal stress protein	-3,28	Hypothetical, unclassified, unknown			
PA4329	*pykA*	pyruvate kinase II	-3,36	Energy metabolism	Carbon compound catabolism		
PA4333		probable fumarase	-2,26	Energy metabolism			
PA4335		hypothetical protein	-2,05	Hypothetical, unclassified, unknown			
PA4336		conserved hypothetical protein	-2,73	Hypothetical, unclassified, unknown			
PA4339		probable phospholipase	2,22	Fatty acid and phospholipid metabolism			
PA4340		hypothetical protein	2,21	Hypothetical, unclassified, unknown			
PA4341		probable transcriptional regulator	4,62	Transcriptional regulators			
PA4342		probable amidase	2,99	Putative enzymes			
PA4343		probable major facilitator superfamily (MFS) transporter	16,80	Membrane proteins	Transport of small molecules		
PA4344		probable hydrolase	6,53	Putative enzymes			
PA4345		hypothetical protein	2,10	Hypothetical, unclassified, unknown			
PA4348		conserved hypothetical protein	-2,54	Hypothetical, unclassified, unknown			
PA4350	*olsB*	OlsB	5,87	Hypothetical, unclassified, unknown			
PA4351	*olsA*	OlsA	10,42	Fatty acid and phospholipid metabolism			
PA4352	*uspN*	Universal stress protein	-8,55	Hypothetical, unclassified, unknown			
PA4353		conserved hypothetical protein	2,18	Hypothetical, unclassified, unknown			
PA4354		conserved hypothetical protein	5,22	Transcriptional regulators			
PA4355	*pyeM*	PyeM	4,70	Membrane proteins	Transport of small molecules	Transcriptional regulators	
PA4356	*xenB*	xenobiotic reductase	-4,65	Antibiotic resistance and susceptibility	Adaptation, Protection		
PA4357	*feoA*	conserved hypothetical protein	-12,99	Hypothetical, unclassified, unknown			
PA4358	*feoB*	FeoB	-8,06	Membrane proteins	Transport of small molecules		
PA4359		conserved hypothetical protein	-3,46	Hypothetical, unclassified, unknown			
PA4360		hypothetical protein	2,71	Hypothetical, unclassified, unknown			
PA4362		hypothetical protein	-4,57	Hypothetical, unclassified, unknown			
PA4363	*iciA*	inhibitor of chromosome initiation IciA	2,21	DNA replication, recombination, modification and repair			
PA4364		hypothetical protein	4,20	Hypothetical, unclassified, unknown			
PA4365	*lysE*	Lysine efflux permease	2,78	Membrane proteins	Transport of small molecules		
PA4366	*sodB*	superoxide dismutase	-4,69	Adaptation, Protection			
PA4368		hypothetical protein	2,02	Hypothetical, unclassified, unknown			
PA4370	*icmP*	Insulin-cleaving metalloproteinase outer membrane protein precursor	-13,89	Membrane proteins			
PA4371		hypothetical protein	-3,98	Hypothetical, unclassified, unknown			
PA4372		Lipoprotein	-9,71	Hypothetical, unclassified, unknown			
PA4373		hypothetical protein	-9,01	Hypothetical, unclassified, unknown			
PA4375	*mexW*	Resistance-Nodulation-Cell Division (RND) multidrug efflux transporter MexW	-2,01	Membrane proteins	Transport of small molecules		
PA4376	*pncB2*	nicotinate phosphoribosyltransferase	-2,72	Biosynthesis of cofactors, prosthetic groups and carrier			
PA4377		hypothetical protein	2,14	Hypothetical, unclassified, unknown			
PA4378	*warB*	WarB	25,88	Adaptation, Protection			
PA4379	*warA*	WarA	31,00	Hypothetical, unclassified, unknown			
PA4382		hypothetical protein	7,23	Hypothetical, unclassified, unknown			
PA4383		conserved hypothetical protein	2,93	Membrane proteins			
PA4384		hypothetical protein	-10,87	Hypothetical, unclassified, unknown			
PA4385	*groEL*	GroEL protein	-2,21	Chaperones & heat shock proteins			
PA4386	*groES*	GroES protein	-2,89	Chaperones & heat shock proteins			
PA4390		hypothetical protein	5,08	Hypothetical, unclassified, unknown			
PA4391		hypothetical protein	4,08	Hypothetical, unclassified, unknown			
PA4392		conserved hypothetical protein	2,05	Hypothetical, unclassified, unknown			
PA4394		conserved hypothetical protein	2,12	Hypothetical, unclassified, unknown			
PA4397	*panE*	ketopantoate reductase	-2,27	Biosynthesis of cofactors, prosthetic groups and carrier	Nucleotide biosynthesis and metabolism		
PA4398		two-component sensor	-2,90	Two-component regulatory systems			
PA4399		conserved hypothetical protein	-2,05	Hypothetical, unclassified, unknown			
PA4401		probable glutathione S-transferase	-3,48	Putative enzymes			
PA4402	*argJ*	glutamate N-acetyltransferase	-6,58	Amino acid biosynthesis and metabolism			
PA4403	*secA*	secretion protein SecA	-2,96	Protein secretion/export apparatus			
PA4406	*lpxC*	UDP-3-O-acyl-N-acetylglucosamine deacetylase	-2,10	Cell Wall / LPS / Capsule			
PA4406.1			-5,26	Non-coding RNA gene			
PA4407	*ftsZ*	cell division protein FtsZ	-4,29	Cell division			
PA4409	*ftsQ*	cell division protein FtsQ	-2,90	Cell division			
PA4410	*ddlB*	D-alanine--D-alanine ligase	-6,71	Cell Wall / LPS / Capsule			
PA4411	*murC*	UDP-N-acetylmuramate--alanine ligase	-6,33	Cell Wall / LPS / Capsule			
PA4412	*murG*	UDP-N-acetylglucosamine--N-acetylmuramyl-(pentapeptide) pyrophosphoryl-undecap	-6,33	Cell Wall / LPS / Capsule	Carbon compound catabolism		
PA4413	*ftsW*	cell division protein FtsW	-4,74	Cell division			
PA4414	*murD*	UDP-N-acetylmuramoylalanine--D-glutamate ligase	-6,71	Cell Wall / LPS / Capsule			
PA4415	*mraY*	phospho-N-acetylmuramoyl-pentapeptide-transferase	-2,07	Cell Wall / LPS / Capsule			
PA4416	*murF*	UDP-N-acetylmuramoylalanyl-D-glutamyl-2, 6-diaminopimelate--D-alanyl-D-alanyl liga	-2,60	Cell Wall / LPS / Capsule			
PA4417	*murE*	UDP-N-acetylmuramoylalanyl-D-glutamate-2, 6-diaminopimelate ligase	-2,23	Cell Wall / LPS / Capsule			
PA4424		conserved hypothetical protein	-5,18	Hypothetical, unclassified, unknown			
PA4425	*gmhA*	sedoheptulose 7-phosphate isomerase GmhA	-4,67	Putative enzymes			
PA4426		Lipoprotein	-6,94	Hypothetical, unclassified, unknown			
PA4427	*sspB*	stringent starvation protein B	-3,47	Adaptation, Protection			
PA4428	*sspA*	stringent starvation protein A	-3,58	Adaptation, Protection			
PA4429		probable cytochrome c1 precursor	-4,76	Energy metabolism			
PA4430		probable cytochrome b	-3,83	Energy metabolism			
PA4431		probable iron-sulfur protein	-2,53	Putative enzymes			
PA4433	*rplM*	50S ribosomal protein L13	2,02	Translation, post-translational modification, degradation			
PA4434		probable oxidoreductase	-4,74	Putative enzymes			
PA4435		probable acyl-CoA dehydrogenase	2,71	Putative enzymes			
PA4436		probable transcriptional regulator	2,52	Transcriptional regulators			
PA4437		hypothetical protein	2,82	Hypothetical, unclassified, unknown			
PA4438		conserved hypothetical protein	2,11	Hypothetical, unclassified, unknown			
PA4439	*trpS*	tryptophanyl-tRNA synthetase	-3,72	Translation, post-translational modification, degradation	Amino acid biosynthesis and metabolism		
PA4444	*mltB1*	soluble and membrane-bound lytic transglycosylases	-5,62	Cell Wall / LPS / Capsule			
PA4447	*hisC1*	histidinol-phosphate aminotransferase	-4,07	Amino acid biosynthesis and metabolism			
PA4453		conserved hypothetical protein	-2,18	Hypothetical, unclassified, unknown			
PA4456		probable ATP-binding component of ABC transporter	2,99	Transport of small molecules			
PA4457	*kdsD*	arabinose-5-phosphate isomerase KdsD	3,13	Secreted Factors (toxins, enzymes, alginate)			
PA4461	*lptB*	Lipopolysaccharide export system ATP-binding protein LptB	-2,30	Transport of small molecules			
PA4463		conserved hypothetical protein	-14,93	Hypothetical, unclassified, unknown			
PA4464	*ptsN*	nitrogen regulatory IIA protein	-9,26	Transport of small molecules			
PA4465		conserved hypothetical protein	-5,29	Hypothetical, unclassified, unknown			
PA4467		hypothetical protein	-9,62	Membrane proteins			
PA4468	*sodM*	superoxide dismutase	-22,73	Adaptation, Protection			
PA4469		hypothetical protein	-26,32	Hypothetical, unclassified, unknown			
PA4470	*fumC1*	fumarate hydratase	-23,26	Energy metabolism			
PA4476		hypothetical protein	3,72	Hypothetical, unclassified, unknown			
PA4477	*cafA*	cytoplasmic axial filament protein (Rnase G)	2,09	Cell division			
PA4478		conserved hypothetical protein	2,45	Hypothetical, unclassified, unknown			
PA4479	*mreD*	rod shape-determining protein MreD	7,16	Cell Wall / LPS / Capsule	Cell division		
PA4480	*mreC*	rod shape-determining protein MreC	7,74	Cell Wall / LPS / Capsule	Cell division		
PA4481	*mreB*	rod shape-determining protein MreB	3,41	Cell Wall / LPS / Capsule	Cell division		
PA4482	*gatC*	Glu-tRNA(Gln) amidotransferase subunit C	-2,46	Translation, post-translational modification, degradation			
PA4483	*gatA*	Glu-tRNA(Gln) amidotransferase subunit A	-3,88	Translation, post-translational modification, degradation			
PA4484	*gatB*	Glu-tRNA(Gln) amidotransferase subunit B	-4,27	Translation, post-translational modification, degradation			
PA4487	*magF*	MagF	-4,76	Hypothetical, unclassified, unknown			
PA4491	*magB*	MagB	-2,34	Hypothetical, unclassified, unknown			x
PA4493	*roxR*	RoxR	-4,88	Two-component regulatory systems	Transcriptional regulators		
PA4495		hypothetical protein	-5,95	Hypothetical, unclassified, unknown			
PA4496	*dppA1*	probable binding protein component of ABC transporter	-9,26	Transport of small molecules			
PA4497	*dppA2*	probable binding protein component of ABC transporter	-12,50	Transport of small molecules			
PA4502	*dppA4*	probable binding protein component of ABC transporter	-2,90	Transport of small molecules			
PA4504	*dppC*	dipeptide ABC transporter permease DppC	-2,23	Transport of small molecules	Membrane proteins		
PA4505	*dppD*	dipeptide ABC transporter ATP-binding protein DppD	-4,24	Transport of small molecules			
PA4506	*dppF*	dipeptide ABC transporter ATP-binding protein DppF	-5,43	Transport of small molecules			
PA4514	*piuA*	probable outer membrane receptor for iron transport	-3,17	Transport of small molecules			
PA4515	*piuC*	conserved hypothetical protein	-4,31	Hypothetical, unclassified, unknown			
PA4516		hypothetical protein	-9,71	Hypothetical, unclassified, unknown			
PA4517		conserved hypothetical protein	-2,05	Hypothetical, unclassified, unknown			
PA4520		probable chemotaxis transducer	22,29	Adaptation, Protection	Chemotaxis		
PA4523		hypothetical protein	-2,13	Hypothetical, unclassified, unknown			
PA4524.1		tRNA-Thr	-8,20	Non-coding RNA gene			
PA4525	*pilA*	type 4 fimbrial precursor PilA	-8,70	Motility and Attachment			x
PA4526	*pilB*	type 4 fimbrial biogenesis protein PilB	-5,24	Motility and Attachment			x
PA4528	*pilD*	type 4 prepilin peptidase PilD	-2,07	Secreted Factors (toxins, enzymes, alginate)	Protein secretion/export apparatus	Motility and Attachment	x
PA4529	*coaE*	dephosphocoenzyme A kinase	-2,70	Biosynthesis of cofactors, prosthetic groups and carrier			
PA4530		conserved hypothetical protein	-4,74	Hypothetical, unclassified, unknown			
PA4535		hypothetical protein	-2,30	Hypothetical, unclassified, unknown			
PA4536		hypothetical protein	-4,37	Hypothetical, unclassified, unknown			
PA4537		hypothetical protein	-3,15	Hypothetical, unclassified, unknown			
PA4538	*ndh*	NADH dehydrogenase	-3,30	Energy metabolism			
PA4539		hypothetical protein	3,28	Hypothetical, unclassified, unknown			
PA4540		hypothetical protein	2,79	Hypothetical, unclassified, unknown			
PA4541	*lepA*	Pseudomonas aeruginosa-derived large extracellular protease, LepA	3,83	Membrane proteins	Secreted Factors (toxins, enzymes, alginate)		x
PA4541.1		tRNA-Lys	4,51	Non-coding RNA gene			
PA4541.2		tRNA-Pro	4,11	Non-coding RNA gene			
PA4541.3		tRNA-Asn	3,95	Non-coding RNA gene			
PA4542	*clpB*	ClpB protein	3,10	Translation, post-translational modification, degradation			
PA4543		conserved hypothetical protein	2,07	Hypothetical, unclassified, unknown			
PA4544	*rluD*	pseudouridine synthase	2,60	Transcription, RNA processing and degradation			
PA4546	*pilS*	two-component sensor PilS	-3,44	Two-component regulatory systems			x
PA4547	*pilR*	two-component response regulator PilR	-10,31	Two-component regulatory systems	Transcriptional regulators		x
PA4549	*fimT*	type 4 fimbrial biogenesis protein FimT	5,43	Motility and Attachment			x
PA4550	*fimU*	type 4 fimbrial biogenesis protein FimU	-4,29	Motility and Attachment			x
PA4551	*pilV*	type 4 fimbrial biogenesis protein PilV	-4,63	Motility and Attachment			x
PA4552	*pilW*	type 4 fimbrial biogenesis protein PilW	-9,01	Motility and Attachment			x
PA4553	*pilX*	type 4 fimbrial biogenesis protein PilX	-17,86	Motility and Attachment			x
PA4554	*pilY1*	type 4 fimbrial biogenesis protein PilY1	-3,57	Motility and Attachment			x
PA4555	*pilY2*	type 4 fimbrial biogenesis protein PilY2	-6,33	Motility and Attachment			x
PA4556	*pilE*	type 4 fimbrial biogenesis protein PilE	-11,36	Motility and Attachment			x
PA4557	*lytB*	LytB protein	-2,42	Adaptation, Protection			
PA4558		probable peptidyl-prolyl cis-trans isomerase, FkbP-type	-2,57	Translation, post-translational modification, degradation	Chaperones & heat shock proteins		
PA4562		conserved hypothetical protein	2,96	Hypothetical, unclassified, unknown			
PA4564		conserved hypothetical protein	5,23	Hypothetical, unclassified, unknown			
PA4565	*proB*	glutamate 5-kinase	8,30	Amino acid biosynthesis and metabolism			x
PA4566	*obg*	GTP-binding protein Obg	12,48	Adaptation, Protection			
PA4567	*rpmA*	50S ribosomal protein L27	3,99	Translation, post-translational modification, degradation			
PA4568	*rplU*	50S ribosomal protein L21	5,00	Translation, post-translational modification, degradation			
PA4570		hypothetical protein	-8,13	Hypothetical, unclassified, unknown			
PA4571		probable cytochrome c	-2,65	Energy metabolism			
PA4574		conserved hypothetical protein	4,63	Hypothetical, unclassified, unknown			
PA4575		hypothetical protein	2,67	Hypothetical, unclassified, unknown			
PA4576		probable ATP-dependent protease	-2,44	Putative enzymes			
PA4578		hypothetical protein	-5,10	Hypothetical, unclassified, unknown			
PA4579		hypothetical protein	-3,61	Hypothetical, unclassified, unknown			
PA4580		conserved hypothetical protein	3,43	Hypothetical, unclassified, unknown			
PA4581	*rtcR*	transcriptional regulator RtcR	3,90	Transcriptional regulators			
PA4581.1		tRNA-Arg	7,71	Non-coding RNA gene			
PA4582		conserved hypothetical protein	66,98	Hypothetical, unclassified, unknown			
PA4583		conserved hypothetical protein	31,43	Hypothetical, unclassified, unknown			
PA4584		conserved hypothetical protein	22,78	Hypothetical, unclassified, unknown			
PA4585	*rtcA*	RNA 3'-terminal phosphate cyclase	18,18	Transcription, RNA processing and degradation			
PA4586		Lipoprotein	72,26	Membrane proteins			
PA4587	*ccpR*	cytochrome c551 peroxidase precursor	-2,90	Energy metabolism			
PA4589		probable outer membrane protein precursor	-3,73	Membrane proteins	Transport of small molecules		
PA4590	*pra*	protein activator	-13,70	Transport of small molecules	Carbon compound catabolism		
PA4595		probable ATP-binding component of ABC transporter	-3,41	Transport of small molecules			
PA4596	*esrC*	EsrC	13,23	Transcriptional regulators			
PA4597	*oprJ*	Multidrug efflux outer membrane protein OprJ precursor	2,73	Transport of small molecules	Antibiotic resistance and susceptibility	Membrane proteins	
PA4598	*mexD*	Resistance-Nodulation-Cell Division (RND) multidrug efflux transporter MexD	3,08	Transport of small molecules	Antibiotic resistance and susceptibility	Membrane proteins	
PA4599	*mexC*	Resistance-Nodulation-Cell Division (RND) multidrug efflux membrane fusion protein M	10,00	Transport of small molecules	Antibiotic resistance and susceptibility		
PA4600	*nfxB*	transcriptional regulator NfxB	-3,33	Transcriptional regulators			
PA4601	*morA*	motility regulator	-5,56	Membrane proteins			
PA4604		conserved hypothetical protein	-2,20	Hypothetical, unclassified, unknown			
PA4605		conserved hypothetical protein	-2,11	Hypothetical, unclassified, unknown			
PA4607		hypothetical protein	-29,41	Hypothetical, unclassified, unknown			
PA4608	*mapZ*	MapZ	-2,71	Hypothetical, unclassified, unknown			
PA4610		hypothetical protein	4,56	Hypothetical, unclassified, unknown			
PA4611		hypothetical protein	-10,10	Hypothetical, unclassified, unknown			
PA4614	*mscL*	conductance mechanosensitive channel	-2,56	Adaptation, Protection	Transport of small molecules	Membrane proteins	
PA4615	*fprB*	FprB	-2,58	Putative enzymes			
PA4621		probable oxidoreductase	3,51	Putative enzymes			
PA4623		hypothetical protein	5,19	Hypothetical, unclassified, unknown			
PA4628	*lysP*	lysine-specific permease	-2,35	Transport of small molecules	Membrane proteins		
PA4629		hypothetical protein	18,80	Hypothetical, unclassified, unknown			
PA4630		hypothetical protein	5,87	Hypothetical, unclassified, unknown			
PA4632		Lipoprotein	-2,35	Hypothetical, unclassified, unknown			
PA4633		probable chemotaxis transducer	2,44	Adaptation, Protection	Chemotaxis		
PA4634		hypothetical protein	6,34	Hypothetical, unclassified, unknown			
PA4635		conserved hypothetical protein	18,75	Hypothetical, unclassified, unknown			
PA4637		hypothetical protein	11,08	Hypothetical, unclassified, unknown			
PA4638		hypothetical protein	8,45	Hypothetical, unclassified, unknown			
PA4639		Lipoprotein	2,18	Hypothetical, unclassified, unknown			
PA4644		hypothetical protein	-2,30	Hypothetical, unclassified, unknown			
PA4647	*uraA*	uracil permease	-2,16	Transport of small molecules	Membrane proteins		
PA4648	*cupE1*	Pilin subunit CupE1	-28,57	Motility and Attachment	Cell Wall / LPS / Capsule		
PA4649	*cupE2*	Pilin subunit CupE2	-25,00	Motility and Attachment	Cell Wall / LPS / Capsule		
PA4650	*cupE3*	Pilin subunit CupE3	-17,24	Motility and Attachment	Cell Wall / LPS / Capsule		
PA4651	*cupE4*	Pilin assembly chaperone CupE4	-52,63	Motility and Attachment	Cell Wall / LPS / Capsule	Chaperones & heat shock proteins	
PA4652	*cupE5*	Fimbrial usher protein CupE5	-9,17	Motility and Attachment	Cell Wall / LPS / Capsule		
PA4653	*cupE6*	Adhesin-like protein CupE6	-28,57	Motility and Attachment	Cell Wall / LPS / Capsule		
PA4654		probable major facilitator superfamily (MFS) transporter	10,58	Membrane proteins	Transport of small molecules		
PA4655	*hemH*	ferrochelatase	2,17	Biosynthesis of cofactors, prosthetic groups and carrier			
PA4656		conserved hypothetical protein	3,20	Hypothetical, unclassified, unknown			
PA4657		hypothetical protein	-2,46	Hypothetical, unclassified, unknown			
PA4660	*phr*	deoxyribodipyrimidine photolyase	-2,06	DNA replication, recombination, modification and repair			
PA4661	*pagL*	Lipid A 3-O-deacylase	-3,45	Hypothetical, unclassified, unknown			
PA4662	*murI*	glutamate racemase	-2,03	Cell Wall / LPS / Capsule			
PA4663	*moeB*	molybdopterin biosynthesis MoeB protein	-2,99	Biosynthesis of cofactors, prosthetic groups and carrier			
PA4664	*prmC*	S-adenosylmethionine-dependent methyltransferase, PrmC	-2,26	Biosynthesis of cofactors, prosthetic groups and carrier			
PA4667		Lipoprotein	-3,05	Hypothetical, unclassified, unknown			
PA4668	*lolB*	lipoprotein localization protein LolB	-6,80	Cell Wall / LPS / Capsule			
PA4669.1		tRNA-Gln	2,12	Non-coding RNA gene			
PA4673.1		tRNA-Met	2,93	Non-coding RNA gene			
PA4674		Antitoxin HigA	6,87	Hypothetical, unclassified, unknown			
PA4675	*chtA*	ChtA	-5,43	Transport of small molecules			
PA4686		hypothetical protein	-3,82	Hypothetical, unclassified, unknown			
PA4687	*hitA*	ferric iron-binding periplasmic protein HitA	-8,00	Transport of small molecules			
PA4688	*hitB*	iron (III)-transport system permease HitB	-5,56	Transport of small molecules	Membrane proteins		
PA4690.3		tRNA-Ala	7,83	Non-coding RNA gene			
PA4690.4		tRNA-Ile	11,06	Non-coding RNA gene			
PA4693	*pssA*	phosphatidylserine synthase	-3,14	Fatty acid and phospholipid metabolism			
PA4694	*ilvC*	ketol-acid reductoisomerase	-3,40	Amino acid biosynthesis and metabolism	Biosynthesis of cofactors, prosthetic groups and carrier		
PA4695	*ilvH*	acetolactate synthase isozyme III small subunit	-2,21	Amino acid biosynthesis and metabolism	Biosynthesis of cofactors, prosthetic groups and carrier		
PA4697		hypothetical protein	-2,81	Hypothetical, unclassified, unknown			
PA4700	*mrcB*	penicillin-binding protein 1B	8,71	Cell Wall / LPS / Capsule			
PA4701		conserved hypothetical protein	-5,21	Hypothetical, unclassified, unknown			
PA4702		hypothetical protein	-7,94	Hypothetical, unclassified, unknown			
PA4703		hypothetical protein	-4,33	Hypothetical, unclassified, unknown			
PA4704.2	*prrF2*	sRNA	-7,75	Non-coding RNA gene			
PA4708	*phuT*	Heme-transport protein, PhuT	-2,11	Transport of small molecules			
PA4710	*phuR*	Heme/Hemoglobin uptake outer membrane receptor PhuR precursor	-10,53	Transport of small molecules			
PA4712		hypothetical protein	3,10	Hypothetical, unclassified, unknown			
PA4713		hypothetical protein	4,72	Hypothetical, unclassified, unknown			
PA4714		conserved hypothetical protein	-2,72	Hypothetical, unclassified, unknown			
PA4715		probable aminotransferase	-2,46	Putative enzymes			
PA4716		hypothetical protein	3,25	Hypothetical, unclassified, unknown			
PA4717		conserved hypothetical protein	3,37	Hypothetical, unclassified, unknown			
PA4718		Lipoprotein	4,78	Hypothetical, unclassified, unknown			
PA4720	*trmA*	tRNA (uracil-5-)-methyltransferase	-2,90	Transcription, RNA processing and degradation			
PA4724		probable aminoacyl-transfer RNA synthetase (class I)	5,78	Putative enzymes			
PA4724.1		conserved hypothetical protein	6,85	Hypothetical, unclassified, unknown			
PA4726.11	*crcZ*	CrcZ	-2,92	Non-coding RNA gene	Transcriptional regulators		
PA4729	*panB*	3-methyl-2-oxobutanoate hydroxymethyltransferase	-2,51	Biosynthesis of cofactors, prosthetic groups and carrier			
PA4730	*panC*	pantoate--beta-alanine ligase	-3,68	Biosynthesis of cofactors, prosthetic groups and carrier			
PA4731	*panD*	aspartate 1-decarboxylase precursor	-2,02	Amino acid biosynthesis and metabolism	Biosynthesis of cofactors, prosthetic groups and carrier		
PA4732	*pgi*	glucose-6-phosphate isomerase	-3,29	Energy metabolism	Carbon compound catabolism		
PA4736		hypothetical protein	-2,11	Hypothetical, unclassified, unknown			
PA4740	*pnp*	polyribonucleotide nucleotidyltransferase	-4,65	Transcription, RNA processing and degradation			
PA4741	*rpsO*	30S ribosomal protein S15	3,04	Translation, post-translational modification, degradation			
PA4742	*truB*	tRNA pseudouridine 55 synthase	3,64	Translation, post-translational modification, degradation	Transcription, RNA processing and degradation		

PA4743	rbfA	ribosome-binding factor A	5,41	Translation, post-translational modification, degradation	Adaptation, Protection		
PA4744	infB	translation initiation factor IF-2	2,13	Translation, post-translational modification, degradation			
PA4745	nusA	N utilization substance protein A	2,76	Transcription, RNA processing and degradation			
PA4746		conserved hypothetical protein	5,66	Hypothetical, unclassified, unknown			
PA4747	secG	secretion protein SecG	3,28	Membrane proteins	Protein secretion/export apparatus		
PA4748	tpiA	triosephosphate isomerase	5,11	Energy metabolism	Central intermediary metabolism		
PA4754		hypothetical protein	-2,00	Membrane proteins			
PA4755	greA	transcription elongation factor GreA	-2,95	Transcription, RNA processing and degradation			
PA4756	carB	carbamoylphosphate synthetase large subunit	-5,24	Amino acid biosynthesis and metabolism	Nucleotide biosynthesis and metabolism		
PA4757		conserved hypothetical protein	-3,77	Membrane proteins			
PA4758	carA	carbamoyl-phosphate synthase small chain	-2,38	Amino acid biosynthesis and metabolism	Nucleotide biosynthesis and metabolism		
PA4762	grpE	heat shock protein GrpE	2,10	DNA replication, recombination, modification and repair	Chaperones & heat shock proteins		
PA4763	recN	DNA repair protein RecN	7,31	DNA replication, recombination, modification and repair			
PA4766		conserved hypothetical protein	3,18	Hypothetical, unclassified, unknown			
PA4769		probable transcriptional regulator	2,06	Transcriptional regulators			
PA4770	lldP	L-lactate permease	11,30	Transport of small molecules			
PA4778	cueR	CueR	-3,18	Transcriptional regulators			
PA4779		hypothetical protein	2,22	Membrane proteins			
PA4781		cyclic di-GMP phosphodiesterase	-3,36	Cell Wall / LPS / Capsule	Transcriptional regulators	Motility and Attachment	x
PA4782		Lipoprotein	4,72	Hypothetical, unclassified, unknown			
PA4783		conserved hypothetical protein	8,87	Membrane proteins			
PA4785		probable acyl-CoA thiolase	5,40	Putative enzymes			
PA4786		probable short-chain dehydrogenase	-2,10	Putative enzymes			
PA4793		Lipoprotein	-3,51	Hypothetical, unclassified, unknown			
PA4794		hypothetical protein	-3,46	Hypothetical, unclassified, unknown			
PA4795		hypothetical protein	-6,06	Hypothetical, unclassified, unknown			
PA4796		hypothetical protein	-7,09	Hypothetical, unclassified, unknown			
PA4797		probable transposase	2,05	Relative to phage, transposon, or plasmid			
PA4798		hypothetical protein	-4,83	Hypothetical, unclassified, unknown			
PA4799		hypothetical protein	6,33	Hypothetical, unclassified, unknown			
PA4800		hypothetical protein	6,87	Hypothetical, unclassified, unknown			
PA4801		hypothetical protein	2,51	Hypothetical, unclassified, unknown			
PA4804		POTRA-like domain-containing usher, CupB3	6,82	Membrane proteins	Transport of small molecules	Protein secretion/export apparatus	
PA4806		probable transcriptional regulator	3,15	Transcriptional regulators			
PA4807	selB	selenocysteine-specific elongation factor	-2,15	Translation, post-translational modification, degradation			
PA4809	fdhE	FdhE protein	-5,38	Energy metabolism			
PA4810	fdnI	nitrate-inducible formate dehydrogenase, gamma subunit	-3,30	Energy metabolism			
PA4811	fdnH	nitrate-inducible formate dehydrogenase, beta subunit	-3,98	Energy metabolism			
PA4812	fdnG	formate dehydrogenase-O, major subunit	-2,63	Energy metabolism			
PA4813	lipC	lipase LipC	6,38	Fatty acid and phospholipid metabolism			
PA4814	fadH2	2,4-dienoyl-CoA reductase FadH2	13,21	Fatty acid and phospholipid metabolism			
PA4815		hypothetical protein	2,45	Membrane proteins			
PA4816		hypothetical protein	11,05	Hypothetical, unclassified, unknown			
PA4817		hypothetical protein	15,53	Hypothetical, unclassified, unknown			
PA4818		conserved hypothetical protein	5,75	Membrane proteins			
PA4819		probable glycosyl transferase	5,93	Putative enzymes			
PA4820		hypothetical protein	12,02	Hypothetical, unclassified, unknown			
PA4821		probable transporter	3,81	Membrane proteins	Transport of small molecules		
PA4822		hypothetical protein	9,30	Membrane proteins			
PA4823		hypothetical protein	14,66	Hypothetical, unclassified, unknown			
PA4824		hypothetical protein	13,55	Hypothetical, unclassified, unknown			
PA4825	mgtA	Mg(2+) transport ATPase, P-type 2	7,10	Transport of small molecules			
PA4826		hypothetical protein	4,66	Hypothetical, unclassified, unknown			
PA4834	cntI	CntI	2,45	Membrane proteins			
PA4835	cntM	CntM	2,96	Membrane proteins			
PA4837	cntO	CntO	2,17	Membrane proteins	Transport of small molecules		
PA4843	gcbA	GcbA	3,15	Two-component regulatory systems	Transcriptional regulators		
PA4844	ctpL	CtpL	5,21	Adaptation, Protection	Chemotaxis		
PA4845	dipZ	thiol:disulfide interchange protein DipZ	3,33	Translation, post-translational modification, degradation	Chaperones & heat shock proteins		
PA4849		hypothetical protein	2,87	Membrane proteins			
PA4852		conserved hypothetical protein	4,49	Hypothetical, unclassified, unknown			
PA4853	fis	DNA-binding protein Fis	2,89	DNA replication, recombination, modification and repair	Transcriptional regulators	Transcription, RNA processing and degradation	
PA4855	purD	phosphoribosylamine–glycine ligase	-2,02	Nucleotide biosynthesis and metabolism			x
PA4858		conserved hypothetical protein	6,94	Hypothetical, unclassified, unknown			
PA4859		probable permease of ABC transporter	6,53	Transport of small molecules	Membrane proteins		
PA4860		probable permease of ABC transporter	5,04	Transport of small molecules	Membrane proteins		
PA4861		probable ATP-binding component of ABC transporter	2,17	Transport of small molecules			
PA4863		hypothetical protein	-2,98	Hypothetical, unclassified, unknown			
PA4864	ureD	urease accessory protein	-5,46	Central intermediary metabolism			
PA4865	ureA	urease gamma subunit	-10,42	Central intermediary metabolism			
PA4866		conserved hypothetical protein	-10,99	Hypothetical, unclassified, unknown			
PA4867	ureB	urease beta subunit	-13,89	Central intermediary metabolism			
PA4868	ureC	urease alpha subunit	-16,67	Central intermediary metabolism			
PA4869		hypothetical protein	-3,38	Hypothetical, unclassified, unknown			
PA4870		conserved hypothetical protein	4,50	Hypothetical, unclassified, unknown			
PA4873		probable heat-shock protein	10,26	Chaperones & heat shock proteins			
PA4874		conserved hypothetical protein	-3,37	Hypothetical, unclassified, unknown			
PA4875		hypothetical protein	-2,99	Hypothetical, unclassified, unknown			
PA4876	osmE	osmotically inducible lipoprotein OsmE	-2,00	Membrane proteins	Adaptation, Protection		
PA4877		hypothetical protein	2,54	Hypothetical, unclassified, unknown			
PA4879		conserved hypothetical protein	3,57	Hypothetical, unclassified, unknown			
PA4881		hypothetical protein	24,55	Hypothetical, unclassified, unknown			
PA4882		hypothetical protein	5,02	Hypothetical, unclassified, unknown			
PA4883		hypothetical protein	10,06	Hypothetical, unclassified, unknown			
PA4884		hypothetical protein	15,58	Hypothetical, unclassified, unknown			
PA4887		probable major facilitator superfamily (MFS) transporter	3,92	Membrane proteins	Transport of small molecules		x
PA4889		probable oxidoreductase	2,38	Putative enzymes			
PA4893	ureG	urease accessory protein UreG	-4,02	Biosynthesis of cofactors, prosthetic groups and carrier			
PA4894		hypothetical protein	-6,76	Membrane proteins			
PA4898	opdK	histidine porin OpdK	8,42	Membrane proteins	Transport of small molecules		
PA4900		probable major facilitator superfamily (MFS) transporter	5,30	Membrane proteins	Transport of small molecules		
PA4901	mdlC	benzoylformate decarboxylase	5,34	Carbon compound catabolism			
PA4903		probable major facilitator superfamily (MFS) transporter	6,34	Membrane proteins	Transport of small molecules		
PA4904	vanA	vanillate O-demethylase oxygenase subunit	5,10	Carbon compound catabolism			
PA4907		probable short-chain dehydrogenase	-7,09	Putative enzymes			
PA4908		hypothetical protein	2,24	Hypothetical, unclassified, unknown			
PA4911		probable permease of ABC branched-chain amino acid transporter	3,38	Transport of small molecules	Membrane proteins		
PA4912		branched chain amino acid ABC transporter membrane protein	6,79	Transport of small molecules	Membrane proteins		
PA4914	amaR	transcriptional regulator, AmaR	-2,93	Transcriptional regulators			
PA4915		probable chemotaxis transducer	-4,63	Adaptation, Protection	Chemotaxis		
PA4916	nrtR	Nudix-related transcriptional regulator NrtR	3,90	Hypothetical, unclassified, unknown			
PA4917	nadD2	nicotinate mononucleotide adenylyltransferase NadD2	5,03	Hypothetical, unclassified, unknown			
PA4918	pcnA	nicotinamidase, PcnA	55,24	Hypothetical, unclassified, unknown			
PA4919	pncB1	nicotinate phosphoribosyltransferase	26,99	Biosynthesis of cofactors, prosthetic groups and carrier			
PA4920	nadE	NH3-dependent NAD synthetase	3,00	Amino acid biosynthesis and metabolism	Biosynthesis of cofactors, prosthetic groups and carrier		
PA4921	choE	cholinesterase, ChoE	6,02	Hypothetical, unclassified, unknown			
PA4922	azu	azurin precursor	-12,35	Energy metabolism			
PA4924		Lipoprotein	4,62	Membrane proteins			
PA4925		conserved hypothetical protein	-11,63	Hypothetical, unclassified, unknown			
PA4926		conserved hypothetical protein	-3,39	Hypothetical, unclassified, unknown			
PA4928		conserved hypothetical protein	2,63	Hypothetical, unclassified, unknown			
PA4929		hypothetical protein	-4,41	Membrane proteins			
PA4930	alr	biosynthetic alanine racemase	3,79	Amino acid biosynthesis and metabolism			
PA4931	dnaB	replicative DNA helicase	3,87	DNA replication, recombination, modification and repair			
PA4932	rplI	50S ribosomal protein L9	9,27	Translation, post-translational modification, degradation			
PA4933		hypothetical protein	9,20	Membrane proteins			
PA4934	rpsR	30S ribosomal protein S18	8,60	Translation, post-translational modification, degradation			
PA4935	rpsF	30S ribosomal protein S6	8,34	Translation, post-translational modification, degradation			
PA4936		probable rRNA methylase	-2,20	Transcription, RNA processing and degradation			
PA4937.1		tRNA-Leu	2,89	Non-coding RNA gene			
PA4939		conserved hypothetical protein	2,84	Hypothetical, unclassified, unknown			
PA4940		conserved hypothetical protein	3,96	Membrane proteins			
PA4943		probable GTP-binding protein	2,28	Putative enzymes			
PA4945	miaA	delta 2-isopentenylpyrophosphate transferase	2,01	Translation, post-translational modification, degradation	Adaptation, Protection		
PA4946	mutL	DNA mismatch repair protein MutL	-3,03	DNA replication, recombination, modification and repair			
PA4948		conserved hypothetical protein	4,75	Hypothetical, unclassified, unknown			
PA4949		conserved hypothetical protein	3,05	Hypothetical, unclassified, unknown			
PA4953	motB	chemotaxis protein MotB	4,07	Adaptation, Protection	Chemotaxis	Membrane proteins	x
PA4954	motA	chemotaxis protein MotA	3,88	Adaptation, Protection	Chemotaxis		x
PA4957	psd	phosphatidylserine decarboxylase	-3,97	Fatty acid and phospholipid metabolism	Amino acid biosynthesis and metabolism		
PA4958	fimW	FimW	-7,75	Hypothetical, unclassified, unknown			
PA4959	fimX	FimX	-3,80	Motility and Attachment			
PA4960		probable phosphoserine phosphatase	2,77	Amino acid biosynthesis and metabolism			
PA4961		hypothetical protein	-2,10	Membrane proteins			
PA4964	parC	topoisomerase IV subunit A	-3,38	DNA replication, recombination, modification and repair			
PA4966		hypothetical protein	-2,29	Hypothetical, unclassified, unknown			
PA4967	parE	topoisomerase IV subunit B	-3,21	DNA replication, recombination, modification and repair			
PA4970		conserved hypothetical protein	4,14	Hypothetical, unclassified, unknown			
PA4971	aspP	adenosine diphosphate sugar pyrophosphatase	4,50	Energy metabolism			
PA4972		Liporprotein	2,36	Hypothetical, unclassified, unknown			
PA4974		probable outer membrane protein precursor	-3,51	Protein secretion/export apparatus			
PA4975		NAD(P)H quinone oxidoreductase	-3,48	Energy metabolism			
PA4976	aruH	Arginine:Pyruvate Transaminas, AruH	-3,22	Amino acid biosynthesis and metabolism			
PA4977	aruI	2-ketoarginine decarboxylase, AruI	2,24	Amino acid biosynthesis and metabolism			
PA4978		hypothetical protein	3,67	Putative enzymes			
PA4979		probable acyl-CoA dehydrogenase	2,53	Putative enzymes			
PA4980		probable enoyl-CoA hydratase/isomerase	2,09	Putative enzymes			
PA4981	lysP	lysine-specific permease	2,23	Membrane proteins	Transport of small molecules		
PA4982		probable two-component sensor	7,62	Two-component regulatory systems			
PA4983		probable two-component response regulator	2,99	Two-component regulatory systems	Transcriptional regulators		
PA4984		probable transcriptional regulator	2,27	Transcriptional regulators			
PA4985		Uncharacterized protein	10,89	Hypothetical, unclassified, unknown			
PA4986		probable oxidoreductase	2,29	Putative enzymes			
PA4989		probable transcriptional regulator	5,61	Transcriptional regulators			
PA4990		SMR multidrug efflux transporter	4,09	Membrane proteins	Antibiotic resistance and susceptibility	Transport of small molecules	
PA4994		probable acyl-CoA dehydrogenase	5,95	Putative enzymes			
PA4995		probable acyl-CoA dehydrogenase	8,11	Putative enzymes			
PA4999	waaL	O-antigen ligase, WaaL	-2,14	Membrane proteins			
PA5001	ssg	cell surface-sugar biosynthetic glycosyltransferase, Ssg	-2,44	Hypothetical, unclassified, unknown			
PA5007	wapG	WapG	-2,96	Putative enzymes			
PA5008	wapP	WapP	-3,06	Putative enzymes			
PA5009	waaP	lipopolysaccharide kinase WaaP	-2,32	Cell Wall / LPS / Capsule			
PA5013	ilvE	branched-chain amino acid transferase	-3,45	Amino acid biosynthesis and metabolism			
PA5015	aceE	pyruvate dehydrogenase	-5,49	Energy metabolism	Amino acid biosynthesis and metabolism		
PA5016	aceF	dihydrolipoamide acetyltransferase	-4,27	Energy metabolism	Carbon compound catabolism		
PA5017	dipA	DipA	2,39	Hypothetical, unclassified, unknown			x
PA5018	msrA	peptide methionine sulfoxide reductase	-4,85	Translation, post-translational modification, degradation			x
PA5019		conserved hypothetical protein	4,03	Hypothetical, unclassified, unknown			
PA5021		probable sodium/hydrogen antiporter	2,18	Membrane proteins	Transport of small molecules		
PA5022		conserved hypothetical protein	-3,02	Hypothetical, unclassified, unknown			
PA5023		conserved hypothetical protein	-2,05	Hypothetical, unclassified, unknown			
PA5025	metY	homocysteine synthase	-6,10	Amino acid biosynthesis and metabolism			
PA5026		hypothetical protein	-2,07	Hypothetical, unclassified, unknown			
PA5027	uspO	Universal stress protein	-13,89	Hypothetical, unclassified, unknown			
PA5028		conserved hypothetical protein	-2,15	Hypothetical, unclassified, unknown			
PA5029		probable transcriptional regulator	2,25	Transcriptional regulators			
PA5031		probable short chain dehydrogenase	10,05	Putative enzymes			
PA5032		probable transcriptional regulator	6,73	Transcriptional regulators			
PA5033		hypothetical protein	-2,87	Hypothetical, unclassified, unknown			
PA5040	pilQ	Type 4 fimbrial biogenesis outer membrane protein PilQ precursor	-4,93	Motility and Attachment			x
PA5041	pilP	type 4 fimbrial biogenesis protein PilP	-2,49	Motility and Attachment			x
PA5047		Lipoprotein	4,88	Hypothetical, unclassified, unknown			
PA5048		probable nuclease	6,08	Putative enzymes			
PA5049	rpmE	50S ribosomal protein L31	3,86	Translation, post-translational modification, degradation			
PA5052		hypothetical protein	-2,10	Hypothetical, unclassified, unknown			
PA5056	phaC1	poly(3-hydroxyalkanoic acid) synthase 1	-6,37	Central intermediary metabolism			
PA5057	phaD	poly(3-hydroxyalkanoic acid) depolymerase	-8,00	Carbon compound catabolism			
PA5058	phaC2	poly(3-hydroxyalkanoic acid) synthase 2	-29,41	Central intermediary metabolism			
PA5059		probable transcriptional regulator	-41,67	Transcriptional regulators			
PA5063	ubiE	ubiquinone biosynthesis methyltransferase UbiE	-2,09	Energy metabolism	Biosynthesis of cofactors, prosthetic groups and carrier		
PA5064		hypothetical protein	-2,87	Hypothetical, unclassified, unknown			
PA5065	ubiB	ubiquinone biosynthetic protein UbiB	-4,83	Biosynthesis of cofactors, prosthetic groups and carrier	Putative enzymes		
PA5068	tatA	translocation protein TatA	-2,23	Protein secretion/export apparatus			
PA5069	tatB	translocation protein TatB	-2,54	Protein secretion/export apparatus			
PA5070	tatC	transport protein TatC	-2,70	Membrane proteins	Transport of small molecules		
PA5072	mcpK	McpK	27,03	Adaptation, Protection	Chemotaxis		
PA5073		hypothetical protein	3,75	Hypothetical, unclassified, unknown			
PA5074		probable ATP-binding component of ABC transporter	-3,38	Transport of small molecules			
PA5075		probable permease of ABC transporter	-2,56	Transport of small molecules	Membrane proteins		
PA5079		conserved hypothetical protein	-2,18	Hypothetical, unclassified, unknown			
PA5082	dguC	DguC	2,17	Transport of small molecules			
PA5083	dguB	Rid2 subfamily protein	5,18	Hypothetical, unclassified, unknown			
PA5084	dguA	DguA	11,27	Putative enzymes			
PA5085	dguR	DguR	2,53	Transcriptional regulators			
PA5086	tli5b1	type VI secretion lipase immunity protein, Tli5b1	4,55	Hypothetical, unclassified, unknown			
PA5088	tli5b3	type VI secretion lipase immunity protein, Tli5b3	-2,45	Hypothetical, unclassified, unknown			
PA5089	tle5b	type VI secretion phospholipase D effector Tle5b	-2,36	Secreted Factors (toxins, enzymes, alginate)			
PA5090	vgrG5	VgrG5	-2,04	Hypothetical, unclassified, unknown			
PA5091	hutG	N-formylglutamate amidohydrolase	-3,66	Amino acid biosynthesis and metabolism			
PA5092	hutI	imidazolone-5-propionate hydrolase HutI	-2,40	Amino acid biosynthesis and metabolism			
PA5094		probable ATP-binding component of ABC transporter	-2,07	Transport of small molecules			
PA5099		probable transporter	2,04	Transport of small molecules	Membrane proteins		
PA5101		hypothetical protein	-4,33	Hypothetical, unclassified, unknown			
PA5102		hypothetical protein	3,55	Membrane proteins			
PA5105	hutC	histidine utilization repressor HutC	2,36	Transcriptional regulators			
PA5107	blc	outer membrane lipoprotein Blc	6,97	Membrane proteins			
PA5108		Lipoprotein	7,73	Hypothetical, unclassified, unknown			
PA5109		hypothetical protein	-2,07	Hypothetical, unclassified, unknown			
PA5112	estA	esterase EstA	-2,68	Fatty acid and phospholipid metabolism	Secreted Factors (toxins, enzymes, alginate)	Protein secretion/export apparatus	x
PA5114		hypothetical protein	4,13	Membrane proteins			
PA5118	thiI	thiazole biosynthesis protein ThiI	6,19	Biosynthesis of cofactors, prosthetic groups and carrier			
PA5119	glnA	glutamine synthetase	-5,85	Amino acid biosynthesis and metabolism			
PA5121		hypothetical protein	4,03	Membrane proteins			
PA5122		hypothetical protein	-2,02	Hypothetical, unclassified, unknown			
PA5123		hypothetical protein	-2,62	Hypothetical, unclassified, unknown			
PA5125	ntrC	two-component response regulator NtrC	-4,88	Two-component regulatory systems	Transcriptional regulators		
PA5128	secB	secretion protein SecB	-3,66	Protein secretion/export apparatus			
PA5140	hisF1	imidazoleglycerol-phosphate synthase, cyclase subunit	-3,65	Amino acid biosynthesis and metabolism			
PA5141	hisA	phosphoribosylformimino-5-aminoimidazole carboxamide	-3,08	Amino acid biosynthesis and metabolism			
PA5142	hisH1	glutamine amidotransferase	-2,74	Amino acid biosynthesis and metabolism			
PA5144		hypothetical protein	2,04	Hypothetical, unclassified, unknown			
PA5147	mutY	A / G specific adenine glycosylase	-2,31	DNA replication, recombination, modification and repair			
PA5148		conserved hypothetical protein	-2,28	Hypothetical, unclassified, unknown			
PA5149.1		tRNA-Phe	4,16	Non-coding RNA gene			
PA5151		hypothetical protein	9,38	Membrane proteins			
PA5153		amino acid (lysine/arginine/ornithine/histidine/octopine) ABC transporter periplasmic	-3,72	Transport of small molecules			
PA5154		probable permease of ABC transporter	-2,16	Transport of small molecules	Membrane proteins		
PA5155		amino acid (lysine/arginine/ornithine/histidine/octopine) ABC transporter membrane	-2,08	Transport of small molecules	Membrane proteins		
PA5156		hypothetical protein	7,42	Hypothetical, unclassified, unknown			
PA5157		probable transcriptional regulator	2,58	Transcriptional regulators			
PA5159		multidrug resistance protein	-3,10	Transport of small molecules	Antibiotic resistance and susceptibility		
PA5161	rmlB	dTDP-D-glucose 4,6-dehydratase	-2,35	Cell Wall / LPS / Capsule			
PA5162	rmlD	dTDP-4-dehydrorhamnose reductase	-5,13	Cell Wall / LPS / Capsule			
PA5163	rmlA	glucose-1-phosphate thymidylyltransferase	-9,35	Cell Wall / LPS / Capsule			
PA5164	rmlC	dTDP-4-dehydrorhamnose 3,5-epimerase	-12,82	Cell Wall / LPS / Capsule			
PA5166	dctD	DctD	-3,18	Two-component regulatory systems	Transcriptional regulators	Transport of small molecules	
PA5170	arcD	arginine/ornithine antiporter	-9,01	Amino acid biosynthesis and metabolism	Membrane proteins	Transport of small molecules	
PA5171	arcA	arginine deiminase	-29,41	Amino acid biosynthesis and metabolism			
PA5172	arcB	ornithine carbamoyltransferase, catabolic	-45,45	Amino acid biosynthesis and metabolism			
PA5173	arcC	carbamate kinase	-35,71	Amino acid biosynthesis and metabolism			
PA5175	cysQ	3,5-bisphosphate nucleotidase CysQ	3,91	Putative enzymes			
PA5176		conserved hypothetical protein	3,74	Hypothetical, unclassified, unknown			
PA5181.1	P34	P34	29,05	Non-coding RNA gene			
PA5189		probable transcriptional regulator	2,34	Transcriptional regulators			
PA5191		hypothetical protein	-2,65	Hypothetical, unclassified, unknown			
PA5195		probable heat shock protein	2,11	Chaperones & heat shock proteins			
PA5196		hypothetical protein	-2,44	Hypothetical, unclassified, unknown			
PA5197	rimK	ribosomal protein S6 modification protein	-2,30	Translation, post-translational modification, degradation	Transcription, RNA processing and degradation		
PA5203	gshA	glutamate--cysteine ligase	-6,10	Amino acid biosynthesis and metabolism	Biosynthesis of cofactors, prosthetic groups and carrier		
PA5205		conserved hypothetical protein	-2,43	Membrane proteins			
PA5206	argE	acetylornithine deacetylase	-2,03	Amino acid biosynthesis and metabolism			
PA5210		probable secretion pathway ATPase	-3,68	Protein secretion/export apparatus			
PA5213	gcvP1	glycine cleavage system protein P1	-4,83	Amino acid biosynthesis and metabolism	Central intermediary metabolism		
PA5214	gcvH1	glycine cleavage system protein H1	-2,44	Amino acid biosynthesis and metabolism	Central intermediary metabolism		
PA5217		probable binding protein component of ABC iron transporter	-9,71	Transport of small molecules			
PA5220		hypothetical protein	-10,10	Hypothetical, unclassified, unknown			
PA5221	ubiI	probable FAD-dependent monooxygenase	-2,84	Putative enzymes			
PA5222		hypothetical protein	-2,82	Hypothetical, unclassified, unknown			
PA5223	ubiH	ubiH protein	-4,39	Energy metabolism	Biosynthesis of cofactors, prosthetic groups and carrier		
PA5224	pepP	aminopeptidase P	-3,33	Translation, post-translational modification, degradation			
PA5228		conserved hypothetical protein	2,74	Hypothetical, unclassified, unknown			
PA5230		probable permease of ABC transporter	-6,94	Membrane proteins	Transport of small molecules		
PA5231		probable ATP-binding/permease fusion ABC transporter	-4,63	Membrane proteins	Transport of small molecules		
PA5232		conserved hypothetical protein	-3,13	Hypothetical, unclassified, unknown			
PA5233		hypothetical protein	-2,13	Hypothetical, unclassified, unknown			
PA5235	glpT	glycerol-3-phosphate transporter	6,04	Membrane proteins	Transport of small molecules		
PA5238		probable O-antigen acetylase	3,81	Membrane proteins	Cell Wall / LPS / Capsule	Putative enzymes	
PA5240	trxA	thioredoxin	-3,26	Nucleotide biosynthesis and metabolism	Energy metabolism	Translation, post-translational modification, degradation	
PA5242	ppk	polyphosphate kinase	-2,69	Nucleotide biosynthesis and metabolism	Adaptation, Protection		
PA5243	hemB	delta-aminolevulinic acid dehydratase	-3,02	Biosynthesis of cofactors, prosthetic groups and carrier			
PA5245		conserved hypothetical protein	-2,79	Hypothetical, unclassified, unknown			
PA5246		conserved hypothetical protein	2,37	Hypothetical, unclassified, unknown			
PA5253	algP	alginate regulatory protein AlgP	-9,90	Transcriptional regulators			x
PA5255	algQ	Alginate regulatory protein AlgQ	-3,92	Transcriptional regulators			x
PA5259	hemD	uroporphyrinogen-III synthetase	4,29	Biosynthesis of cofactors, prosthetic groups and carrier			
PA5260	hemC	porphobilinogen deaminase	5,30	Biosynthesis of cofactors, prosthetic groups and carrier			
PA5261	algR	alginate biosynthesis regulatory protein AlgR	2,03	Two-component regulatory systems	Transcriptional regulators	Secreted Factors (toxins, enzymes, alginate)	x
PA5262	fimS	FimS	3,06	Two-component regulatory systems	Motility and Attachment		x
PA5264		hypothetical protein	2,98	Membrane proteins			
PA5266	vgrG6	VgrG6	-2,90	Hypothetical, unclassified, unknown			
PA5267	hcpB	secreted protein Hcp	-3,36	Secreted Factors (toxins, enzymes, alginate)			

PA5270		hypothetical protein	-2,18	Hypothetical, unclassified, unknown		
PA5271		hypothetical protein	-14,93	Hypothetical, unclassified, unknown		
PA5272	*cyaA*	adenylate cyclase	2,12	Adaptation, Protection		x
PA5277	*lysA*	diaminopimelate decarboxylase	-3,10	Amino acid biosynthesis and metabolism		
PA5278	*dapF*	diaminopimelate epimerase	-4,05	Amino acid biosynthesis and metabolism		
PA5281		probable hydrolase	-3,07	Putative enzymes		
PA5282		probable major facilitator superfamily (MFS) transporter	7,37	Membrane proteins	Transport of small molecules	
PA5284		hypothetical protein	2,64	Hypothetical, unclassified, unknown		
PA5288	*glnK*	nitrogen regulatory protein P-II 2	-4,33	Antibiotic resistance and susceptibility	Central intermediary metabolism	
PA5290		conserved hypothetical protein	2,11	Hypothetical, unclassified, unknown		
PA5291	*betT2*	BetT2	-4,15	Membrane proteins	Transport of small molecules	
PA5293		probable transcriptional regulator	3,83	Transcriptional regulators		
PA5294		putative multidrug efflux pump	5,20	Hypothetical, unclassified, unknown		
PA5296	*rep*	ATP-dependent DNA helicase Rep	3,20	DNA replication, recombination, modification and repair		
PA5299		hypothetical protein	2,97	Hypothetical, unclassified, unknown		
PA5302	*dadX*	catabolic alanine racemase	-4,95	Amino acid biosynthesis and metabolism		
PA5303		conserved hypothetical protein	-3,95	Hypothetical, unclassified, unknown		
PA5305		conserved hypothetical protein	-2,40	Hypothetical, unclassified, unknown		
PA5307		hypothetical protein	2,69	Hypothetical, unclassified, unknown		
PA5308	*lrp*	leucine-responsive regulatory protein	-2,96	Transcriptional regulators	Central intermediary metabolism	
PA5310		Lipoprotein	-2,79	Hypothetical, unclassified, unknown		
PA5311		probable major facilitator superfamily (MFS) transporter	16,34	Membrane proteins	Transport of small molecules	
PA5313	*gabT2*	Transaminase	2,45	Carbon compound catabolism	Putative enzymes	
PA5314		hypothetical protein	2,17	Hypothetical, unclassified, unknown		
PA5315	*rpmG*	50S ribosomal protein L33	7,46	Translation, post-translational modification, degradation		
PA5316	*rpmB*	50S ribosomal protein L28	8,85	Translation, post-translational modification, degradation		
PA5316.1			3,28	Non-coding RNA gene		
PA5317	*dppA5*	probable binding protein component of ABC dipeptide transporter	-6,49	Transport of small molecules		
PA5318		hypothetical protein	-7,52	Hypothetical, unclassified, unknown		
PA5320	*coaC*	Phosphopantothenoylcysteine synthase/(R)-4'-phospho-N-pantothenoylcysteine decar	-3,64	DNA replication, recombination, modification and repair	Biosynthesis of cofactors, prosthetic groups and carrier	
PA5321	*dut*	deoxyuridine 5'-triphosphate nucleotidohydrolase	-6,54	Nucleotide biosynthesis and metabolism		
PA5323	*argB*	acetylglutamate kinase	-2,19	Amino acid biosynthesis and metabolism		
PA5324	*sphR*	Sphingosine-responsive Regulator, SphR	4,86	Adaptation, Protection	Transcriptional regulators	
PA5325	*sphA*	SphA	5,93	Adaptation, Protection		
PA5326	*sphD*	SphD	2,36	Hypothetical, unclassified, unknown		
PA5327	*sphC*	SphC	30,77	Putative enzymes		x
PA5328	*sphB*	SphB	44,12	Energy metabolism		
PA5329		conserved hypothetical protein	-5,68	Hypothetical, unclassified, unknown		
PA5332	*crc*	catabolite repression control protein	2,78	Energy metabolism	Carbon compound catabolism	
PA5335		conserved hypothetical protein	3,33	Hypothetical, unclassified, unknown		
PA5338	*spoT*	guanosine-3',5'-bis(diphosphate) 3'-pyrophosphohydrolase	-2,05	Adaptation, Protection	Nucleotide biosynthesis and metabolism	x
PA5339		conserved hypothetical protein	-6,45	Hypothetical, unclassified, unknown		
PA5340		Lipoprotein	-5,78	Hypothetical, unclassified, unknown		
PA5341		hypothetical protein	3,59	Hypothetical, unclassified, unknown		
PA5344	*oxyR*	OxyR	-3,80	Transcriptional regulators		x
PA5345	*recG*	ATP-dependent DNA helicase RecG	-5,99	DNA replication, recombination, modification and repair	Transcriptional regulators	
PA5346	*sadB*	SadB	-4,69	Cell Wall / LPS / Capsule		
PA5349	*rubB*	rubredoxin reductase	-2,70	Carbon compound catabolism		
PA5350	*rubA2*	Rubredoxin 2	-2,16	Carbon compound catabolism		
PA5352		conserved hypothetical protein	4,20	Hypothetical, unclassified, unknown		
PA5353	*glcF*	glycolate oxidase subunit GlcF	5,56	Carbon compound catabolism	Central intermediary metabolism	
PA5354	*glcE*	glycolate oxidase subunit GlcE	6,12	Carbon compound catabolism	Central intermediary metabolism	
PA5355	*glcD*	glycolate oxidase subunit GlcD	4,47	Carbon compound catabolism	Central intermediary metabolism	
PA5359		hypothetical protein	-5,08	Hypothetical, unclassified, unknown		
PA5362		conserved hypothetical protein	-4,57	Hypothetical, unclassified, unknown		
PA5364		probable two-component response regulator	-2,24	Two-component regulatory systems	Transcriptional regulators	
PA5368	*pstC*	membrane protein component of ABC phosphate transporter	2,58	Transport of small molecules	Membrane proteins	
PA5369.3		tRNA-Ala	8,57	Non-coding RNA gene		
PA5369.4		tRNA-Ile	12,28	Non-coding RNA gene		
PA5370		probable major facilitator superfamily (MFS) transporter	2,74	Transport of small molecules	Membrane proteins	
PA5375	*betT1*	BetT1	4,03	Transport of small molecules	Membrane proteins	
PA5376	*cbcV*	CbcV	-4,65	Transport of small molecules		
PA5377	*cbcW*	CbcW	-3,53	Transport of small molecules	Membrane proteins	
PA5379	*sdaB*	L-serine dehydratase	3,72	Amino acid biosynthesis and metabolism		
PA5384	*hocS*	HocS	4,25	Carbon compound catabolism	Central intermediary metabolism	
PA5385	*cdhB*	CdhB, Carnitine dehydrogenase-related gene B	3,75	Carbon compound catabolism		
PA5386	*cdhA*	CdhA, Carnitine dehydrogenase	6,44	Carbon compound catabolism	Putative enzymes	
PA5387	*cdhC*	CdhC, Carnitine dehydrogenase-related gene C	7,58	Carbon compound catabolism		
PA5388	*caiX*	CaiX	8,12	Carbon compound catabolism	Transport of small molecules	
PA5390		probable peptidic bond hydrolase	2,02	Putative enzymes		
PA5393		conserved hypothetical protein	4,00	Hypothetical, unclassified, unknown		
PA5394	*cls*	cardiolipin synthase	2,08	Fatty acid and phospholipid metabolism		
PA5395		conserved hypothetical protein	-4,00	Hypothetical, unclassified, unknown		
PA5403		probable transcriptional regulator	2,67	Transcriptional regulators		
PA5406		hypothetical protein	3,08	Hypothetical, unclassified, unknown		
PA5407		hypothetical protein	4,97	Hypothetical, unclassified, unknown		
PA5408		hypothetical protein	-2,89	Hypothetical, unclassified, unknown		
PA5411	*gbcB*	GbcB	2,86	Amino acid biosynthesis and metabolism		
PA5412		hypothetical protein	2,80	Hypothetical, unclassified, unknown		
PA5413	*ltaA*	low specificity l-threonine aldolase	-7,04	Amino acid biosynthesis and metabolism		
PA5415	*glyA1*	serine hydroxymethyltransferase	-7,04	Amino acid biosynthesis and metabolism		
PA5416	*soxB*	sarcosine oxidase beta subunit	-2,26	Amino acid biosynthesis and metabolism		
PA5418	*soxA*	sarcosine oxidase alpha subunit	-2,81	Carbon compound catabolism		
PA5419	*soxG*	sarcosine oxidase gamma subunit	-2,09	Amino acid biosynthesis and metabolism		
PA5420	*purU2*	formyltetrahydrofolate deformylase	-2,31	Nucleotide biosynthesis and metabolism		
PA5423		hypothetical protein	7,52	Hypothetical, unclassified, unknown		
PA5426	*purE*	phosphoribosylaminoimidazole carboxylase, catalytic subunit	2,43	Nucleotide biosynthesis and metabolism		
PA5427	*adhA*	alcohol dehydrogenase	-15,38	Energy metabolism	Carbon compound catabolism	
PA5428		probable transcriptional regulator	2,06	Transcriptional regulators		
PA5432		probable acetyltransferase	2,26	Putative enzymes		
PA5435		probable transcarboxylase subunit	-3,14	Central intermediary metabolism		
PA5436		probable biotin carboxylase subunit of a transcarboxylase	-2,03	Central intermediary metabolism		
PA5437		probable transcriptional regulator	3,25	Transcriptional regulators		x
PA5442		conserved hypothetical protein	7,21	Membrane proteins		
PA5445		probable coenzyme A transferase	7,73	Putative enzymes		
PA5446		hypothetical protein	-5,85	Hypothetical, unclassified, unknown		
PA5447	*wbpZ*	glycosyltransferase WbpZ	-2,50	Cell Wall / LPS / Capsule		
PA5448	*wbpY*	glycosyltransferase WbpY	-3,46	Cell Wall / LPS / Capsule		
PA5449	*wbpX*	glycosyltransferase WbpX	-3,39	Cell Wall / LPS / Capsule		x
PA5450	*wzt*	ABC subunit of A-band LPS efflux transporter	-2,77	Cell Wall / LPS / Capsule	Transport of small molecules	
PA5452	*wbpW*	phosphomannose isomerase/GDP-mannose WbpW	-2,02	Cell Wall / LPS / Capsule		
PA5453	*gmd*	GDP-mannose 4,6-dehydratase	-2,56	Cell Wall / LPS / Capsule		
PA5455		putative glycosyltransferase	-2,42	Cell Wall / LPS / Capsule		
PA5456		putative glycosyltransferase	-6,33	Cell Wall / LPS / Capsule		
PA5457		methyltransferase	-4,81	Cell Wall / LPS / Capsule		
PA5458		putative acetyltransferase	-2,90	Cell Wall / LPS / Capsule	Membrane proteins	
PA5459		putative methyltransferase	-9,43	Cell Wall / LPS / Capsule		
PA5460		hypothetical protein	-8,70	Hypothetical, unclassified, unknown		
PA5461		hypothetical protein	-7,63	Hypothetical, unclassified, unknown		
PA5464		hypothetical protein	2,44	Hypothetical, unclassified, unknown		
PA5465		hypothetical protein	16,32	Hypothetical, unclassified, unknown		
PA5468		probable citrate transporter	5,68	Transport of small molecules		
PA5469		conserved hypothetical protein	13,16	Membrane proteins		
PA5470		probable peptide chain release factor	28,78	Translation, post-translational modification, degradation		
PA5471	*armZ*	ArmZ	19,12	Antibiotic resistance and susceptibility		
PA5471.1		PA5471 leader peptide	4,53	Hypothetical, unclassified, unknown		
PA5473		conserved hypothetical protein	10,74	Membrane proteins		
PA5475		hypothetical protein	-12,99	Hypothetical, unclassified, unknown		
PA5477		hypothetical protein	3,38	Membrane proteins		
PA5478		conserved hypothetical protein	2,37	Membrane proteins		
PA5480		hypothetical protein	7,26	Hypothetical, unclassified, unknown		
PA5483	*algB*	two-component response regulator AlgB	3,70	Two-component regulatory systems	Transcriptional regulators	
PA5486		conserved hypothetical protein	5,73	Hypothetical, unclassified, unknown		
PA5496	*nrdJb*	class II (cobalamin-dependent) ribonucleotide-diphosphate reductase subunit, NrdJb	-13,51	Nucleotide biosynthesis and metabolism		
PA5497	*nrdJa*	class II (cobalamin-dependent) ribonucleotide-diphosphate reductase subunit, NrdJa	-4,93	Nucleotide biosynthesis and metabolism		
PA5503		probable ATP-binding component of ABC transporter	6,72	Transport of small molecules		
PA5504		D-methionine ABC transporter membrane protein	4,51	Transport of small molecules	Membrane proteins	
PA5505		probable TonB-dependent receptor	-2,21	Transport of small molecules	Membrane proteins	
PA5506		hypothetical protein	2,57	Hypothetical, unclassified, unknown		
PA5509		hypothetical protein	2,85	Hypothetical, unclassified, unknown		
PA5512	*mifS*	MifS	3,88	Two-component regulatory systems	Cell Wall / LPS / Capsule	
PA5514		probable beta-lactamase	4,60	Antibiotic resistance and susceptibility	Putative enzymes	
PA5517		conserved hypothetical protein	3,85	Hypothetical, unclassified, unknown		
PA5518		probable potassium efflux transporter	6,06	Transport of small molecules	Membrane proteins	
PA5519		conserved hypothetical protein	2,42	Hypothetical, unclassified, unknown		
PA5520		hypothetical protein	-2,02	Hypothetical, unclassified, unknown		
PA5522	*pauA6*	Glutamylpolyamine synthetase	6,36	Putative enzymes	Carbon compound catabolism	
PA5523		probable aminotransferase	14,01	Putative enzymes		
PA5524		probable short-chain dehydrogenase	2,38	Putative enzymes		
PA5526		Lipoprotein	6,29	Hypothetical, unclassified, unknown		
PA5530		C5-dicarboxylate transporter	16,56	Membrane proteins	Transport of small molecules	
PA5533		hypothetical protein	3,10	Hypothetical, unclassified, unknown		
PA5534		hypothetical protein	4,34	Hypothetical, unclassified, unknown		
PA5535		conserved hypothetical protein	3,22	Hypothetical, unclassified, unknown		
PA5536	*dksA2*	DksA2	26,94	Adaptation, Protection	Transcriptional regulators	
PA5538	*amiA*	N-acetylmuramoyl-L-alanine amidase	30,15	Cell Wall / LPS / Capsule		
PA5539		RidA subfamily protein	6,32	Hypothetical, unclassified, unknown		
PA5540		hypothetical protein	4,49	Hypothetical, unclassified, unknown		
PA5541	*pyrQ*	dihydroorotase	4,32	Nucleotide biosynthesis and metabolism		
PA5546		conserved hypothetical protein	-2,31	Putative enzymes		
PA5548		probable major facilitator superfamily (MFS) transporter	3,01	Transport of small molecules	Membrane proteins	
PA5549	*glmS*	glucosamine--fructose-6-phosphate aminotransferase	-6,58	Central intermediary metabolism		
PA5552	*glmU*	glucosamine-1-phosphate acetyltransferase/N-acetylglucosamine-1-phosphate uridyltr	-2,54	Cell Wall / LPS / Capsule		
PA5553	*atpC*	ATP synthase epsilon chain	-2,46	Energy metabolism		
PA5554	*atpD*	ATP synthase beta chain	-2,89	Energy metabolism		
PA5555	*atpG*	ATP synthase gamma chain	-2,88	Energy metabolism		
PA5556	*atpA*	ATP synthase alpha chain	-2,46	Energy metabolism		
PA5557	*atpH*	ATP synthase delta chain	-2,39	Energy metabolism		
PA5558	*atpF*	ATP synthase B chain	-2,23	Energy metabolism		
PA5559	*atpE*	atp synthase C chain	-2,12	Energy metabolism		
PA5561	*atpI*	ATP synthase protein I	2,78	Energy metabolism	Membrane proteins	
PA5563	*soj*	chromosome partitioning protein Soj	2,67	Cell division	Central intermediary metabolism	
PA5565	*gidA*	glucose-inhibited division protein A	2,16	Cell division	Central intermediary metabolism	
PA5566		hypothetical protein	4,92	Hypothetical, unclassified, unknown		

Related to iron

PA number	Gene name	Product name	Fold-change	Regulators
**Pyoverdine**				
PA2427	*pvdY*	Pyoverdine biosynthesis protein	-6,13	
PA2426	*pvdS*	sigma factor PvdS	-33,33	PvdS/Fur/OxyR/PA2206/AlgR/PirR/CysB/CzcR/AmgR
PA2425	*pvdG*	Pyoverdine biosynthesis protein	-6,66	PvdS
PA2424	*pvdL*	Non ribosomal peptide synthetase (NRPS)	-6,53	PvdS
PA2413	*pvdH*	L-2,4-diaminobutyrate:2-ketoglutarate 4-aminotransferase, PvdH	-3,84	
PA2412	*mbtH*	NRPS assist	-5,26	
PA2411		probable thioesterase	-5,10	
PA2410	*fpvF*	Fe-pyoverdine utilisation in periplasm	-4,65	
PA2409	*fpvE*	Fe-pyoverdine utilisation in periplasm	-5,98	
PA2408	*fpvD*	Fe-pyoverdine utilisation in periplasm	-5,46	
PA2407	*fpvC*	Fe-pyoverdine utilisation in periplasm	-5,71	
PA2406	*fpvK*	Fe-pyoverdine utilisation in periplasm	-5,12	
PA2405	*fpvJ*	Fe-pyoverdine utilisation in periplasm	-5,52	
PA2404	*fpvH*	Fe-pyoverdine utilisation in periplasm	-4,95	
PA2403	*fpvG*	Fe-pyoverdine utilisation in periplasm	-2,89	PvdS/SigX/FpvI
PA2402	*pvdI*	probable non-ribosomal peptide synthetase (NRPS)	-2,07	PvdS/SigX/FpvI
PA2401	*pvdJ*	NRPS	Unchanged	
PA2399	*pvdD*	NRPS	Unchanged	
PA2398	*fpvA*	ferripyoverdine transporter	-8,13	FpvI/SigX
PA2397	*pvdE*	pyoverdine biosynthesis protein PvdE	-13,51	PvdS
PA2396	*pvdF*	pyoverdine synthetase F	-5,46	PvdS
PA2395	*pvdO*	Pyoverdine chromophore maturation	-8,47	PvdS
PA2394	*pvdN*	Pyoverdine chromophore maturation	-14,49	PvdS
PA2393	*pvdM*	putative dipeptidase	-12,34	PvdS
PA2392	*pvdP*	Pyoverdine chromophore maturation	-3,25	PvdS
PA2391	*opmQ*	RND efflux OMP/recycling of apoyoverdine	-8,69	
PA2390	*pvdT*	RND efflux/recycling of apoyoverdine	-4,60	
PA2389	*pvdR*	RND efflux/recycling of apoyoverdine	-2,36	
PA2388	*fpvR*	Anti-sigma factor	Unchanged	Fur/FpvI/SigX
PA2387	*fpvI*	ECF sigma	Unchanged	Fur/FpvI
PA2386	*pvdA*	L-ornithine N5-oxygenase	-13,15	PvdS/FpvI
PA2385	*pvdQ*	Acylase	-8,47	
PA2384		Fur-like regulator	-4,13	
**Pyochelin**				
PA4231	*pchA*	salicylate biosynthesis isochorismate synthase	-10,52	PchR
PA4230	*pchB*	salicylate biosynthesis protein PchB	-12,50	PchR
PA4229	*pchC*	pyochelin biosynthetic protein PchC	-11,76	PchR
PA4228	*pchD*	pyochelin biosynthesis protein PchD	-10,20	PchR
PA4227	*pchR*	transcriptional regulator PchR	-4,18	Fur/PchR
PA4226	*pchE*	dihydroaeruginoic acid synthetase	-5,71	Fur/PchR
PA4225	*pchF*	pyochelin synthetase	-4,06	Fur/PchR
PA4224	*pchG*	pyochelin biosynthetic protein PchG	-3,27	Fur/PchR
PA4223	*pchH*	probable ATP-binding component of ABC transporter	-2,43	Fur/PchR
PA4222	*pchI*		Unchanged	Fur/PchR
PA4221	*fptA*	Fe(III)-pyochelin outer membrane receptor precursor	-6,49	Fur/PchR
PA4220	*fptB*	hypothetical protein	-6,99	Fur/PchR
**Xenosiderophores**				
PA0149		ECF sigma	Unchanged	Fur
PA0151		TonB dependent transporter	4,12	
PA0192		TonB dependent transporter	6,90	
PA0434		TonB dependent transporter	-2,23	
PA0470	*fiuA*	TonB dependent transporter for ferrichrome	-3,59	
PA0471	*fiuR*	Anti-sigma	-9,34	
PA0472	*fiuI*	ECF sigma	-8,69	Fur
PA0931	*pirA*	ferric enterobactin receptor PirA	-2,92	Fur/PirR
PA1322	*pfuA*	TonB dependent transporter	Unchanged	Fur
PA1363		ECF sigma factor	-3,24	Fur
PA1365	*aleB*	TonB dependent transporter	-2,85	Fur
PA1910	*femA*	TonB dependent mycobactin transporter	Unchanged	Fur
PA2050		ECF sigma	9,11	Fur
PA2057	*sppR*	TonB dependent transporter	7,31	PA2050
PA2058	*sppA*	ABC transporter substrate-binding protein, SppA	13,08	PA2050
PA2059	*sppB*	ABC transporter permease, SppB	23,43	PA2050
PA2060	*sppC*	ABC transporter permease, SppC	8,45	PA2050
PA2061	*sppD*	ABC transporter ATP-binding protein, SppD	3,11	PA2050
PA2289		TonB dependent transporter	-6,41	Fur
PA2466	*foxA*	TonB ferrioxamine dependent transporter	-3,06	Fur
PA2467	*foxR*	Anti-sigma factor FoxR	-3,80	
PA2468	*foxI*	ECF sigma factor FoxI	-4,34	Fur
PA2686	*pfeR*	two-component response regulator PfeR	-3,30	PfeR
PA2687	*pfeS*	Anti sigma	Unchanged	Fur
PA2688	*pfeA*	TonB dependent enterobactin transporter	Unchanged	Fur
PA3531	*bfrB*	Bacterioferritin	10,20	PrrF
PA3899	*fecI*	ECF sigma	-2,25	Fur
PA3900	*fecR*	FecR	-3,84	Fur
PA3901	*fecA*	Fe(III) dicitrate tTonB-dependent ransport protein FecA	-2,60	Fur
PA4168	*fpvB*	second ferric pyoverdine transporter FpvB	-4,06	Fur
PA4514	*piuA*	TonB-dependent tra,sporter	-3,17	Fur
PA4515	*piuC*	conserved hypothetical protein	-4,31	Fur
PA4675	*chtA*	TonB-dependent aerobactin transporter	-5,43	Fur
PA4704,2	*prrF2*	sRNA	-7,70	Fur
PA4896		ECF sigma	Unchanged	Fur
PA4897	*optI*	TonB dependent transporter	Unchanged	Fur
**Heme uptake**				
PA1300	*hxuI*	ECF sigma factor	4,41	Fur
PA1301	*hxuR*	probable transmembrane sensor	4,55	Fur
PA1302	*hxuA*	TonB-dependent transporter	5,94	Fur
PA3407	*hasAp*	heme acquisition protein HasAp	-5,31	HasAp/HasR
PA3408	*hasR*	TonB-dependent transporter	Unchanged	Fur
PA4708	*phuT*	Heme-transport protein, PhuT	-2,10	
PA4710	*phuR*	TonB-dependent transporter	-10,52	Fur

Related to QS

PA number	Gene name	Product name	Fold-change	Schuster 2003	Asfahl & Schuster 2018	Gilbert 2009	Groleau 2020	Bredenbruch 2006
PA0007		cytochrome c	-2,2	LasR/RhlR				
PA0026	*plcB*	phospholipase C, PlcB	-6	LasR/RhlR	LasR/RhlR	LasR		
PA0027		hypothetical protein	-7,5	LasR/RhlR	LasR/RhlR			
PA0028			-5,7	LasR/RhlR				
PA0051	*phzH*	potential phenazine-modifying enzyme	-8,4	LasR/RhlR				
PA0052		hypothetical protein	-6,8	LasR/RhlR	LasR/RhlR			
PA0059	*osmC*	Osmotically induced protein	-2,6	LasR/RhlR				
PA0109			-2,4	LasR/RhlR				
PA0122	*rahU*	Hemolysin	-26,3	LasR/RhlR		LasR		
PA0143	*nuh*	purine nucleosidase Nuh	-5,9	LasR/RhlR	LasR/RhlR	LasR		
PA0178		probable two-component sensor	-10,3		LasR/RhlR			
PA0200			-3,8					MvfR
PA0524	*norB*	nitric-oxide reductase subunit B	-3,5		LasR/RhlR			
PA0572	*impA*	hypothetical protein	-27	LasR/RhlR	LasR/RhlR			
PA0996	*pqsA*	HHQ biosynthesis	-12,5	LasR/RhlR		LasR		
PA0997	*pqsB*	HHQ biosynthesis	-21,7	LasR/RhlR				
PA0998	*pqsC*	HHQ biosynthesis	-21,7	LasR/RhlR				
PA0999	*pqsD*	HHQ biosynthesis	-13,3	LasR/RhlR				
PA1000	*pqsE*	Quinolone signal response protein	-7,7	LasR/RhlR				
PA1001	*phnA*	Anthanilate kinase	-6,4	LasR/RhlR				
PA1002	*phnB*	Anthanilate kinase	-9,2	LasR/RhlR				
PA1003	*mvfR*	PQS regulator	-4,7	LasR/RhlR		LasR		
PA1130	*rhlC*	rhamnosyltransferase 2	-10,8	LasR/RhlR				
PA1174	*napA*	Periplasmic nitrate reductase	-7,1					MvfR
PA1215		AMP binding	-20	LasR/RhlR				
PA1216		SAM methyl transferase	-41,6	LasR/RhlR				
PA1217		Isopropylmalate synthase	-35,7	LasR/RhlR				
PA1218		Phytanoyl CoA dioxygenase	-34,4	LasR/RhlR				
PA1245	*aprX*	T1SS	-3,9	LasR/RhlR		LasR		MvfR
PA1246	*aprD*	alkaline protease secretion protein AprD	-4,1	LasR/RhlR	LasR/RhlR			
PA1248	*aprF*	Alkaline protease secretion outer membrane protein AprF precursor	-10,5	LasR/RhlR	LasR/RhlR			MvfR
PA1249	*aprA*	alkaline metalloproteinase precursor	-45,4	LasR/RhlR	LasR/RhlR			MvfR
PA1250	*aprI*	alkaline proteinase inhibitor AprI	-4,1	LasR/RhlR	LasR/RhlR			
PA1430	*lasR*	transcriptional regulator LasR	-4,3		LasR/RhlR			
PA1431	*rsaL*	regulatory protein RsaL	-5	LasR/MvfR	LasR/RhlR	LasR		
PA1663	*sfa2*	Sfa2	-3,2	LasR/RhlR	LasR/RhlR			
PA1664	*orfX*		-3,2	LasR/RhlR				
PA1665	*fha2*		-5,2	LasR/RhlR				
PA1666	*lip2*		-5	LasR/RhlR				
PA1667	*hsiJ2*		-3,8	LasR/RhlR				
PA1668	*dotU2*	DotU2	-2,6	LasR/RhlR	LasR/RhlR			
PA1784		hypothetical protein	-29,4	LasR/RhlR	LasR/RhlR			
PA1869	*acp1*	Acyl carrier protein	-6,4	LasR/RhlR	LasR/RhlR			
PA1871	*lasA*	LasA protease precursor	-62,5	LasR/RhlR	LasR/RhlR			
PA1893		hypothetical protein	-12,2	LasR/RhlR	LasR/RhlR			
PA1894		hypothetical protein	-16,9	LasR/RhlR	LasR/RhlR			
PA1895		hypothetical protein	-16,1	LasR/RhlR	LasR/RhlR			
PA1896		hypothetical protein	-11,6	LasR/RhlR	LasR/RhlR			
PA1897		hypothetical protein	-10,5	LasR/RhlR	LasR/RhlR			
PA1899	*phzA2*	probable phenazine biosynthesis protein	-16,4				RhlR/MvfR	
PA1900	*phzB2*	probable phenazine biosynthesis protein	-37				RhlR/MvfR	
PA1901	*phzC2*	phenazine biosynthesis protein PhzC	-16,4				RhlR/MvfR	
PA1902	*phzD2*	phenazine biosynthesis protein PhzD	-17,2				RhlR/MvfR	
PA1903	*phzE2*	phenazine biosynthesis protein PhzE	-19,2				RhlR/MvfR	
PA1904	*phzF2*	probable phenazine biosynthesis protein	-21,3				RhlR/MvfR	
PA1905	*phzG2*	probable pyridoxamine 5'-phosphate oxidase	-23,8				RhlR/MvfR	
PA1914	*hvn*	Secreted NADase	-333,3	LasR/RhlR		LasR		
PA2030		hypothetical protein	-10,6	LasR/RhlR				
PA2031		hypothetical protein	-8,4	LasR/RhlR				
PA2066		Rossman fold	-21,2	LasR/RhlR				
PA2067		Hydrolase	-26,7	LasR/RhlR				
PA2068		MFS transporter	-33,3	LasR/RhlR				
PA2069		Carbamoyl transferase	-38,4	LasR/RhlR				
PA2080	*kynU*	kynureninase KynU	-5,4		LasR/RhlR	LasR		
PA2193	*hcnA*	hydrogen cyanide synthase HcnA	-8,3	LasR/RhlR	LasR/RhlR			
PA2194	*hcnB*	hydrogen cyanide synthase HcnB	-9,3	LasR/RhlR	LasR/RhlR			
PA2195	*hcnC*	hydrogen cyanide synthase HcnC	-12,2	LasR/RhlR	LasR/RhlR			
PA2300	*chiC*	Chitinase	-18,8	LasR/RhlR				
PA2301		hypothetical protein	-10,4		LasR/RhlR			
PA2302	*ambE*	AmbE	-14,5	LasR/RhlR	LasR/RhlR			
PA2303	*ambD*	AmbD	-16,1	LasR/RhlR	LasR/RhlR			
PA2304	*ambC*	AmbC	-11,2	LasR/RhlR	LasR/RhlR			
PA2305	*ambB*	AmbB	-3,9	LasR/RhlR	LasR/RhlR	LasR		
PA2327		Permease of ABC	-3	LasR/RhlR				
PA2328		TAT signal	-13,1	LasR/RhlR				
PA2329		ATP of ABC	-9,9	LasR/RhlR				
PA2330		Acyl CoA dehydrogenase	-10,4	LasR/RhlR				
PA2331		Peroxyredoxin	-10,8	LasR/RhlR				
PA2384		Fur-like regulator	-4,1					MvfR
PA2386	*pvdA*	Pyoverdine biosynthesis	-13,1					MvfR
PA2402	*pvdI*	Pyoverdine biosynthesis	-2					MvfR
PA2405	*fpvJ*	Pyoverdine utilisation	-5,5					MvfR
PA2412	*mbtH*	Pyoverdine biosynthesis	-5,3					MvfR
PA2423		hypothetical protein	-4,5	LasR/RhlR	LasR/RhlR			
PA2427		hypothetical protein	-6,1					MvfR
PA2570	*lecA*	LecA lectin	-4,3	LasR/RhlR				
PA2587	*pqsH*	probable FAD-dependent monooxygenase	-7	LasR/RhlR	LasR/RhlR	LasR		
PA2588	*cdpR*	Transcriptional regulator	-8,3	LasR/RhlR	LasR/RhlR	LasR		
PA2592		probable periplasmic spermidine/putrescine-binding protein	-3,3	LasR/RhlR	LasR/RhlR	LasR		
PA2607		conserved hypothetical protein	-8,6		LasR/RhlR			
PA2608		conserved hypothetical protein	-11,4		LasR/RhlR			
PA2939	*paaP*	probable aminopeptidase	-50	LasR/RhlR	LasR/RhlR			
PA2949		esterase	-3,1		LasR/RhlR			
PA3095	*xcpZ*	general secretion pathway protein M	-3,2				RhlR	
PA3096	*xcpY*	general secretion pathway protein L	-3,1				RhlR	
PA3097	*xcpX*	general secretion pathway protein K	-2				RhlR	
PA3098	*xcpW*	general secretion pathway protein J	-5,3				RhlR	
PA3099	*xcpV*	general secretion pathway protein I	-9,2				RhlR	
PA3100	*xcpU*	General secretion pathway outer membrane protein H precursor	-15,8				RhlR	
PA3101	*xcpT*	general secretion pathway protein G	-17,2				RhlR	
PA3102	*xcpS*	general secretion pathway protein F	-3,5				RhlR	
PA3103	*xcpR*	general secretion pathway protein E	-3,8				LasR/RhlR	
PA3105	*xcpQ*	general secretion pathway protein D	-2,5	LasR/RhlR				
PA3305.1	*phrS*	PhrS	-2,7				MvfR	
PA3326	*clpP2*	ClpP2	-5,8	LasR/RhlR	LasR/RhlR	LasR		
PA3327		probable non-ribosomal peptide synthetase	-4	LasR/RhlR	LasR/RhlR			
PA3328		probable FAD-dependent monooxygenase	-3,5	LasR/RhlR	LasR/RhlR			
PA3329		hypothetical protein	-6	LasR/RhlR	LasR/RhlR			
PA3330		probable short chain dehydrogenase	-8,8	LasR/RhlR	LasR/RhlR			
PA3331		cytochrome P450	-9,5	LasR/RhlR	LasR/RhlR			
PA3332		conserved hypothetical protein	-9,9	LasR/RhlR	LasR/RhlR			
PA3333	*fabH2*	3-oxoacyl-[acyl-carrier-protein] synthase III	-8,4	LasR/RhlR	LasR/RhlR			
PA3336		probable major facilitator superfamily (MFS) transporter	-3,5	LasR/RhlR	LasR/RhlR			
PA3361	*lecB*	fucose-binding lectin PA-IIL	-10,5	LasR/RhlR			RhlR	
PA3476	*rhlI*	autoinducer synthesis protein RhlI	-2,1	LasR/RhlR	LasR/RhlR			
PA3477	*rhlR*	transcriptional regulator RhlR	-13,3	LasR/RhlR	LasR/RhlR	LasR		
PA3478	*rhlB*	rhamnosyltransferase chain B	-22,2	LasR/RhlR				
PA3479	*rhlA*	rhamnosyltransferase chain A	-15,8	LasR/RhlR	LasR/RhlR	LasR		
PA3520		Periplasmic binding protein	-5,3	LasR/RhlR				
PA3535	*eprS*	probable serine protease	-5,3	LasR/RhlR	LasR/RhlR	LasR		
PA3615		hypothetical protein	-7,7		LasR/RhlR			
PA3724	*lasB*	elastase LasB	-40	LasR/RhlR		LasR		
PA4078		NRPS	-19,2	LasR/RhlR				
PA4117	*bphP*	bacterial phytochrome, BphP	-5	LasR/RhlR	LasR/RhlR	LasR		
PA4141			-12,7					MvfR
PA4190	*pqsL*	probable FAD-dependent monooxygenase	-9,5	LasR/RhlR	LasR/RhlR			
PA4206	*mexH*	RND efflux	-2,1	LasR/RhlR				MvfR
PA4207	*mexI*	RND efflux	-4,4	LasR/RhlR				
PA4208	*opmD*	OMP	-22,7	LasR/RhlR				
PA4209	*phzM*	probable phenazine-specific methyltransferase	-2,4	LasR/RhlR			RhlR	
PA4210	*phzA1*	probable phenazine biosynthesis protein	-9,5	LasR/RhlR			RhlR	
PA4211	*phzB1*	probable phenazine biosynthesis protein	-6,4	LasR/RhlR			RhlR	
PA4212	*phzC1*	phenazine biosynthesis protein PhzC	-13,1	LasR/RhlR			RhlR	
PA4213	*phzD1*	phenazine biosynthesis protein PhzD	-19,6	LasR/RhlR			RhlR	
PA4214	*phzE1*	phenazine biosynthesis protein PhzE	-19,6	LasR/RhlR			RhlR	
PA4215	*phzF1*	probable phenazine biosynthesis protein	-21,7	LasR/RhlR			RhlR	
PA4216	*phzG1*	probable pyridoxamine 5'-phosphate oxidase	-23,2	LasR/RhlR			RhlR	
PA4217	*phzS*	flavin-containing monooxygenase	-6,6	LasR/RhlR			RhlR	MvfR
PA4218	*ampP*	FptX protein	-4,9					MvfR
PA4220	*fptB*	Pyochelin	-7					MvfR
PA4221	*fptA*	Fe-PCH transporter	-6,5					MvfR
PA4223	*pchH*	Pyochelin	-2,4					MvfR
PA4224	*pchG*	Pyochelin	-3,3					MvfR
PA4225	*pchF*	Pyochelin	-4					MvfR
PA4226	*pchE*	Pyochelin	-5,7					MvfR
PA4228	*pchD*	Pyochelin	-10,2					MvfR
PA4229	*pchC*	Pyochelin	-11,7					MvfR
PA4230	*pchB*	Pyochelin	-12,5					MvfR
PA4231	*pchA*	Pyochelin	-10,5					MvfR
PA4236	*katA*	Catalase	-5,6					MvfR
PA4306	*flp*	Type IVb pilin, Flp	-62,5	LasR/RhlR		LasR		
PA4467		Zn permease	-9,6					MvfR
PA4468	*sodA*	Superoxide dismutase Mn factored	-22,7					MvfR
PA4469		hypothetical protein	-26,3					MvfR
PA4470	*fumC1*	Fumarate hydratase	-23,2					MvfR
PA4590	*pra*	Protein activator	-13,7	LasR/RhlR				
PA4648	*cupE1*	Pilin subunit	-28,5					
PA4649	*cupE2*	Pilin subunit	-25	LasR/RhlR				
PA4650	*cupE3*	Pilin subunit	-17,2	LasR/RhlR				
PA4651	*cupE4*	Chaperone	-52,6	LasR/RhlR				
PA4652	*cupE5*	Usher	-9,1	LasR/RhlR				
PA4653	*cupE6*	Fimbrial adhesin	-28,6	LasR/RhlR				
PA4778	*cueR*	CueR	-3,2	LasR/RhlR	LasR/RhlR	LasR		
PA4869		hypothetical protein	-3,4	LasR/RhlR	LasR/RhlR			
PA5170	*arcD*	Arg/Orn antiporter	-9					MvfR
PA5220		hypothetical protein	-10	LasR/RhlR				
PA5255	*algQ*	Alginate regulatory protein AlgQ	-3,9		LasR/RhlR			
PA5460		hypothetical protein	-8,7					MvfR

Related to virulence factors

PA number	Gene name	Product name	Fold-change	Regulation	
PA0026	*plcB*	phospholipase C, PlcB	-6	QS	
PA0051	*phzH*	potential phenazine-modifying enzyme	-8,4	QS	
PA0122	*rahU*	Hemolysin	-26,3	QS	
PA0996	*pqsA*	HHQ biosynthesis	-12,5	QS	
PA0997	*pqsB*	HHQ biosynthesis	-21,7	QS	
PA0998	*pqsC*	HHQ biosynthesis	-21,7	QS	
PA0999	*pqsD*	HHQ biosynthesis	-13,3	QS	
PA1000	*pqsE*	Quinolone signal response protein	-7,7	QS	
PA1001	*phnA*	Anthanilate kinase	-6,4	QS	
PA1002	*phnB*	Anthanilate kinase	-9,2	QS	
PA1003	*mvfR*	PQS regulator	-4,7	QS	
PA1130	*rhlC*	rhamnosyltransferase 2	-10,8	QS	
PA1248	*aprF*	Alkaline protease secretion outer membrane protein AprF precursor	-10,5	QS	
PA1249	*aprA*	alkaline metalloproteinase precursor	-45,4	QS	
PA1250	*aprI*	alkaline proteinase inhibitor AprI	-4,1	QS	
PA1430	*lasR*	transcriptional regulator LasR	-4,3	QS	
PA1431	*rsaL*	regulatory protein RsaL	-5	QS	
PA1871	*lasA*	LasA protease precursor	-62,5	QS	
PA1899	*phzA2*	probable phenazine biosynthesis protein	-16,4	QS	
PA1900	*phzB2*	probable phenazine biosynthesis protein	-37	QS	
PA1901	*phzC2*	phenazine biosynthesis protein PhzC	-16,4	QS	
PA1902	*phzD2*	phenazine biosynthesis protein PhzD	-17,2	QS	
PA1903	*phzE2*	phenazine biosynthesis protein PhzE	-19,2	QS	
PA1904	*phzF2*	probable phenazine biosynthesis protein	-21,3	QS	
PA1905	*phzG2*	probable pyridoxamine 5'-phosphate oxidase	-23,8	QS	
PA1914	*hvn*	Secreted NADase	-333,3	QS	
PA2080	*kynU*	kynureninase KynU	-5,4	QS	
PA2193	*hcnA*	hydrogen cyanide synthase HcnA	-8,3	QS	
PA2194	*hcnB*	hydrogen cyanide synthase HcnB	-9,3	QS	
PA2195	*hcnC*	hydrogen cyanide synthase HcnC	-12,2	QS	
PA2300	*chiC*	Chitinase	-18,8	QS	
PA2302	*ambE*	AmbE	-14,5	QS	
PA2303	*ambD*	AmbD	-16,1	QS	
PA2304	*ambC*	AmbC	-11,2	QS	
PA2305	*ambB*	AmbB	-3,9	QS	
PA2386	*pvdA*	Pyoverdine biosynthesis	-13,1	QS	Iron
PA2402	*pvdI*	Pyoverdine biosynthesis	-2	QS	Iron
PA2405	*fpvJ*	Pyoverdine utilisation	-5,5	QS	Iron
PA2412	*mbtH*	Pyoverdine biosynthesis	-5,3	QS	Iron
PA2570	*lecA*	LecA lectin	-4,3	QS	
PA2587	*pqsH*	probable FAD-dependent monooxygenase	-7	QS	
PA3361	*lecB*	fucose-binding lectin PA-IIL	-10,5	QS	
PA3476	*rhlI*	autoinducer synthesis protein RhlI	-2,1	QS	
PA3477	*rhlR*	transcriptional regulator RhlR	-13,3	QS	
PA3478	*rhlB*	rhamnosyltransferase chain B	-22,2	QS	
PA3479	*rhlA*	rhamnosyltransferase chain A	-15,8	QS	
PA3724	*lasB*	elastase LasB	-40	QS	
PA4175	*piv (prpL)*	Protease IV	-29,41		Iron
PA4206	*mexH*	RND efflux	-2,1	QS	
PA4207	*mexI*	RND efflux	-4,4	QS	
PA4208	*opmD*	OMP	-22,7	QS	
PA4209	*phzM*	probable phenazine-specific methyltransferase	-2,4	QS	
PA4210	*phzA1*	probable phenazine biosynthesis protein	-9,5	QS	
PA4211	*phzB1*	probable phenazine biosynthesis protein	-6,4	QS	
PA4212	*phzC1*	phenazine biosynthesis protein PhzC	-13,1	QS	
PA4213	*phzD1*	phenazine biosynthesis protein PhzD	-19,6	QS	
PA4214	*phzE1*	phenazine biosynthesis protein PhzE	-19,6	QS	
PA4215	*phzF1*	probable phenazine biosynthesis protein	-21,7	QS	
PA4216	*phzG1*	probable pyridoxamine 5'-phosphate oxidase	-23,2	QS	
PA4217	*phzS*	flavin-containing monooxygenase	-6,6	QS	
PA4220	*fptB*	Pyochelin	-7	QS	Iron
PA4221	*fptA*	Fe-PCH transporter	-6,5	QS	Iron
PA4223	*pchH*	Pyochelin	-2,4	QS	Iron
PA4224	*pchG*	Pyochelin	-3,3	QS	Iron
PA4225	*pchF*	Pyochelin	-4	QS	Iron
PA4226	*pchE*	Pyochelin	-5,7	QS	Iron
PA4228	*pchD*	Pyochelin	-10,2	QS	Iron
PA4229	*pchC*	Pyochelin	-11,7	QS	Iron
PA4230	*pchB*	Pyochelin	-12,5	QS	Iron
PA4231	*pchA*	Pyochelin	-10,5	QS	Iron
**Secretion**					
PA1657	*hsiB2*	T6SS	-2,28	QS	
PA1658	*hsiC2*	T6SS	-3,37	QS	
PA1659	*hsiF2*	T6SS	-3,54	QS	
PA1660	*hsiG2*	T6SS	-4,65	QS	
PA1661	*hsiH2*	T6SS	-3,3	QS	
PA1662	*clpV2*	T6SS	-2,99	QS	
PA1663	*sfa2*	T6SS	-3,23	QS	
PA1664	*orfX*	T6SS	-3,61	QS	
PA1665	*fha2*	T6SS	-5,23	QS	
PA1666	*lip2*	T6SS	-5	QS	
PA1667	*hsiJ2*	T6SS	-3,84	QS	
PA1668	*dotU2*	T6SS	-2,62	QS	
PA3095	*xcpZ*	T2SS	-3,2	QS	
PA3096	*xcpY*	T2SS	-3,1	QS	
PA3097	*xcpX*	T2SS	-2	QS	
PA3098	*xcpW*	T2SS	-5,3	QS	
PA3099	*xcpV*	T2SS	-9,2	QS	
PA3100	*xcpU*	T2SS	-15,8	QS	
PA3101	*xcpT*	T2SS	-17,2	QS	
PA3102	*xcpS*	T2SS	-3,5	QS	
PA3103	*xcpR*	T2SS	-3,8	QS	
PA3105	*xcpQ*	T2SS	-2,5	QS	
PA2360	*hsiA3*	T6SS	-11,49	QS	Iron
PA2361	*icmF3*	T6SS	-2,61		Iron
PA2363	*hsiJ3*	T6SS	-3,84		Iron
PA2365	*hsiB3*	T6SS	-35,71		Iron
PA2366	*hsiC3*	T6SS	-47,61		Iron
PA2367	*hcp3*	T6SS	-55,55		Iron
PA2368	*hsiF3*	T6SS	-66,66		Iron
PA2369	*hsiG3*	T6SS	-32,25		Iron
PA2370	*hsiH3*	T6SS	-41,66		Iron
PA2371	*clpV3*	T6SS	-25,64		Iron
PA2372		T6SS	-17,24		Iron
PA2373	*vgrG3*	T6SS	-7,46		Iron
PA2374	*tseF*	T6SS	-7,57		Iron

Supplementary Table S2. Strains used in this study.

Strains	Relevant characteristics	Sources
***Pseudomonas aeruginosa* H103**	Wild-type strain; prototroph derivative of PAO1	**Hancock and Carey, 1979**
Pseudomonas aeruginosa* PAO1 Δ*pqsA* CTX-*lux::pqsA	*pqsA* mutant containing a copy of the *pqsA* promoter fused to the CTX-*luxCDABE* cassette, Tc^R	**Fletcher et al., 2007**
***Escherichia coli* (pSB401)**	N-acyl homoserine lactones bioluminescent reporter harboring pSB401 that contains a fusion of *luxRI'::luxCDABE*, Tc^R	**Winson et al., 1998**

Tc^R, Tetracycline resistance

Supplementary Table S3. Primers used in this study.

PA number	Gene name	Primer name	Sequence (5' - 3')
**PA0668.1**	16SrRNA	16S-F	AACCTGGGAACTGCATCCAA
		16S-R	CTTCGCCACTGGTGTTCCTT
**PA0044**	*exoT*	exoT-F	TCGAGGCTTCCCGTACCCA
		exoT-R	CAGGGCGACCTTGTCCATT
**PA0409**	*pilH*	pilH-F	GTGATCATCGTCACCACCAA
		pilH-R	TTTCAGCAGGGTCTCTTCGT
**PA0612**	*ptrB*	ptrB-F	GCCAACGAACTGGTCCTG
		ptrB-R	GCAGTCCTCGCAGTCTTCC
**PA0652**	*vfr*	vfr-F	CGAAACGCTGTTCTTCATCA
		vfr-R	GAAATCACCGCTGTTGAGGT
**PA0996**	*pqsA*	pqsA-F	CGGAGTTGCTGGCATTGC
		pqsA-R	CTGTTGCCCATGCCATAGC
**PA1003**	*pqsR*	pqsR-F	AACCTGGAAATCGACCTGTG
		pqsR-R	TGAAATCGTCGAGCAGTACG
**PA1148**	*toxA*	toxA-F	ACGAGAAGCCTTCGAACATC
		toxA-R	TGACGAAGAAGGTGGCATC
**PA1430**	*lasR*	lasR-F	CGGTTTTCTTGAGCTGGAAC
		lasR-R	GCCGAACAGGATCTTCGAG
**PA1713**	*exsA*	exsA-F	ATCGAGGAGTTGCTGATGCT
		exsA-R	TCCATGAATAGCTGCAGACG
**PA1898**	*qscR*	qscR-F	AGGGGAATGGAAAAGCAGAT
		qscR-R	GGGTGTATTCCAGGAGACCA
**PA1899**	*phzA2*	phzA2-F	GGTTTACCGACAACCTGGAATTGC
		phzA2-R	AACAGGCTGTGCCGCTGTAACC
**PA2523**	*czcR*	czcR-F	ACTTCATGGTCAAGCCGTTC
		czcR-R	ACTCCTTGGTCGTCAGGTTG
**PA2570**	*lecA*	lecA-F	ATCCGGGCGATGTCATTACC
		lecA-R	CAAAACGCATCGTGGCAGAT
**PA2896**	*sbrI*	sbrI-F	GGCAAGGTCTCTACCGTTTTC
		sbrI-R	AGGTTTCCTGGTAGATCTCGTC
**PA3006**	*psrA*	psrA-F	CGATTTCGGCGTGAACACTT
		psrA-R	CGAAGAACGGCACCATCAG
**PA3305.1**	*phrS*	phrS-F	CAACTGGAGGCCATCAACAT
		phrS-R	CCTTGCGTGCTCTGTGTATC
**PA3476**	*rhlI*	rhlI-F	GGCTGGGACTTGGCGTAG
		rhlI-R	GGCTGGGACTTGGCGTAG
**PA3477**	*rhlR*	rhlR-F	CTGGGCTTCGATTACTACGC
		rhlR-R	CCCGTAGTTCTGCATCTGGT
**PA3479**	*rhlA*	rhlA-F	AACATTTCAACGTGGTGCTG
		rhlA-R	ATTTCCACCTCGTCGTCCTT
**PA3621.1**	*rsmZ*	rsmZ-F	CGTACAGGGAACACGCAAC
		rsmZ-R	TTCAGTCCCTCGTCATCATC
**PA3622**	*rpoS*	rpoS-F	CACTTCCTTCTCTTCCAAACAACAC
		rpoS-R	AGCTGCGTTGCGTCCAA
**PA3724**	*lasB*	lasB-F	AACCGTGCGTTCTACCTGTT
		lasB-R	TGGTGGCGGTCCAGTAGTA

**PA4210**	*phzA1*	phzA1-F	AACCACTACATCCATTCCTTCG
		phzA1-R	CGGCTATTCCCAATGCAC
**PA4525**	*pilA*	pilA-F	ACCCGCTGAAGACCACTGTT
		pilA-R	CGGTCGCAGTAGAAGCAGTAGTAC
**PA5272**	*cyaA*	cyaA-F	CGCTTCAAGGAGCAGGTATT
		cyaA-R	GAGATGGCGATAGACCAGGA
**PA5338**	*spoT*	spoT-F	CTCCGAGTCGATCAACCTCG
		spoT-R	TGCGTTCCTGGGGAATCTTC

SUPPLEMENTARY REFERENCES

Hancock RE, Carey AM. 1979. Outer membrane of *Pseudomonas aeruginosa*: heat- 2-mercaptoethanol-modifiable proteins. J Bacteriol 140:902-10.

Fletcher MP, Diggle SP, Camara M, Williams P. 2007. Biosensor-based assays for PQS, HHQ and related 2-alkyl-4-quinolone quorum sensing signal molecules. Nat Protoc 2:1254-62.

Winson MK, Swift S, Fish L, Throup JP, Jørgensen F, Chhabra SR, Bycroft BW, Williams P, Stewart GS. 1998. Construction and analysis of *luxCDABE*-based plasmid sensors for investigating *N*-acyl homoserine lactone-mediated quorum sensing. FEMS Microbiol Lett 163: 185–192.
